# Unveiling the Antioxidative Potential of Galangin: Complete and Detailed Mechanistic Insights through Density Functional Theory Studies

**DOI:** 10.1021/acs.joc.4c00611

**Published:** 2024-06-11

**Authors:** Maciej Spiegel

**Affiliations:** Department of Organic Chemistry and Pharmaceutical Technology, Faculty of Pharmacy, Wroclaw Medical University, Borowska 211A, 50-556 Wroclaw, Poland

## Abstract

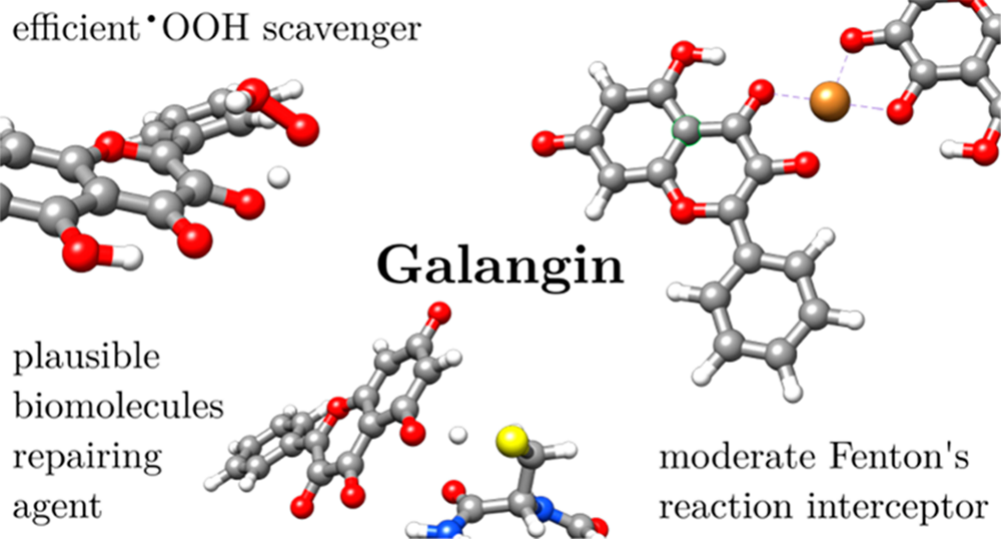

A comprehensive quantum mechanical investigation delved into the antioxidative activity of galangin (**Glg**). Thermochemical and kinetic data were used to assess antiradical, chelating, and renewal potential under physiological conditions. A brief comparison with reference antioxidants and other flavonoids characterized **Glg** as a moderate antioxidative agent. The substance showed significantly lower performance in lipid compared to aqueous solvent—the reaction rates for scavenging ^•^OOH in both media were established at 3.77 × 10^3^ M^–1^ s^–1^ and 6.21 × 10^4^ M^–1^ s^–1^, respectively, accounting for the molar fraction of both interacting molecules at the given pH. The impact of pH value on the kinetics was assessed. Although efficient at chelating Cu(II) ions, the formed complexes can still undergo the Fenton reaction. On the other hand, they persistently scavenge ^•^OH *in statu nascendi*. The flavonoid effectively repairs oxidatively damaged biomolecules except model lipid acids. All **Glg** radicals are readily restored by physiologically prevailing O_2_^•–^. Given this, the polyphenol is expected to participate in antiradical and regenerating activities multiple times, amplifying its antioxidative potential.

## Introduction

Oxidative stress, characterized by an imbalance between the generation of reactive oxygen species (ROS) and the defensive capabilities of cellular antioxidants underpins various physiological processes. While ROS are integral to cellular metabolism and signaling,^1,2^ their excessive production can lead to cellular damage and malfunctions.^3^ This disturbance in redox homeostasis has been implicated in numerous ailments, from neurodegenerative disorders^4,5^ to cardiovascular diseases^6^ and cancer.^7^

Antioxidants, including vitamins (e.g., vitamin C and E),^8^ minerals (e.g., selenium),^8^ and phytochemicals (e.g., polyphenols),^9^ play a pivotal role these processes in a multiple ways. Most of them are capable donating electrons or hydrogen atoms, effectively scavenging free radicals like hydroperoxide radicals that contribute to protein and membrane damage.^10^ Others exhibit the ability to chelate metals participating in Fenton’s process and promptly intercept hydroxyl radicals formed during the reaction. Additionally, some antioxidants have been observed to interfere with enzymes producing oxidative species *in vivo* as a byproduct.^1,2^ Numerous studies^11^ underscore the potential health benefits of a diet rich in antioxidants, emphasizing their role in preventing or ameliorating diseases associated with oxidative stress.

**Figure 1:**
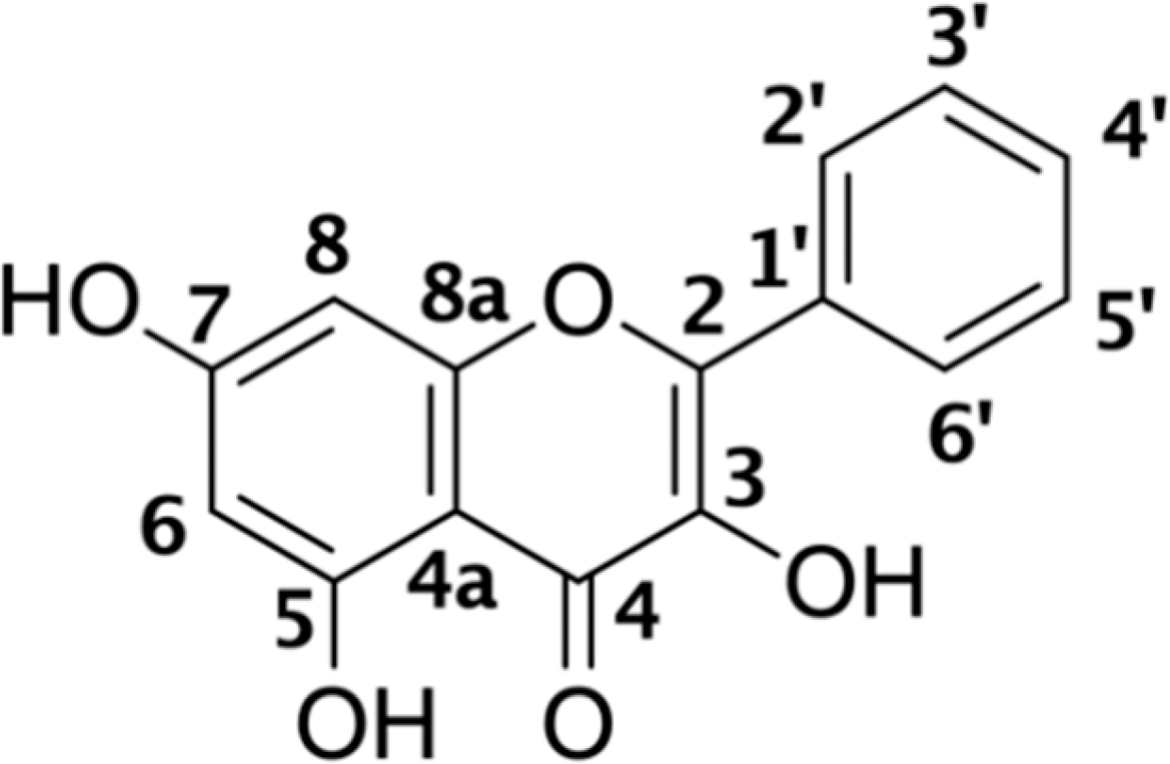
Molecular structure of galangin (**Glg**) with site numbering.

Galangin (referred to as **Glg** and illustrated in Figure 1) is a natural flavonoid present in various plant sources, including *Alpinia officinarum* and *Helichrysum aureonitens*, as well as in propolis.^12^ Alongside pinocembrin and chrysin, it ranks among the most abundant flavonoids discovered in honey.^13^ This bioactive polyphenol has recently garnered attention for its robust antioxidant and anti-inflammatory properties. Research indicates that **Glg** demonstrates protective effects against cellular damage induced by oxidative stress by modulating intracellular communication and boosting the activity of endogenous antioxidant enzymes. Furthermore, galangin exhibits promise in diverse therapeutic applications, spanning from neuroprotection to anticancer effects.^14−18^

In recent years, computational studies on antioxidants have emerged as indispensable tools for unravelling the intricate molecular mechanisms. Through the application of advanced computational tools, such as quantum chemical calculations, valuable insights into the thermodynamics and kinetics of antioxidant reactions, facilitating the identification of crucial structural features that augment their scavenging capabilities can be distinguished.^10,19^ Moreover, computational approaches play a significant role in the rational design of novel antioxidant compounds with optimized properties, providing guidance for the development of potential therapeutic agents.^20−22^

In this research paper, the primary focus lies in utilizing quantum mechanics to thoroughly investigate the antioxidative activity of **Glg**. Even though some other theoretical works have been already conducted in the topic^23−26^ this is the first one to be so comprehensive. Building upon previous research, it can be hypothesized that the substance, given its established antioxidant properties, will showcase an ability to effectively neutralize and scavenge reactive radical species, thereby mitigating oxidative stress. The computational framework employed allows for a exploration of the molecular interactions, structural determinants, and electronic features that govern this efficacy, providing insights into the complete antioxidative activity of this flavonoid.

## Results and Discussion

### Antioxidative Activity Type I

Understanding reaction mechanisms is crucial for rationalizing the reactivity of chemical compounds, especially in the context of antiradical activity. Three primary pathways (reactions 1, 2 and 3) govern the antiradical activity of a substance:

- single electron transfer (SET):
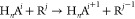
1
- formal hydrogen atom transfer (*f*-HAT):
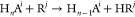
2
- radical adduct formation (RAF):
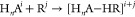
3

The evaluation of free radical scavenging activity focuses on the reactions of **Glg** species with the hydroperoxyl radical ^•^OOH. While the hydroxyl radical, ^•^OH, is widely recognized as the primary initiator of oxidative damage, its high reactivity results in swift reactions with molecules in its proximity before an antioxidant can effectively intercept it. The extended half-lives of peroxyl species, including ^•^OOH, offer antioxidants a window of opportunity to successfully intercept them.^2^ This characteristic not only aids in exploring trends in radical scavenging efficiency but also underscores the crucial role of peroxyradicals as essential reaction partners for polyphenolic antioxidants.^27^ Additionally, ^•^OOH has been proposed to play a pivotal role in the toxic side effects associated with aerobic respiration.^28^

#### Thermochemistry

**Figure 2:**
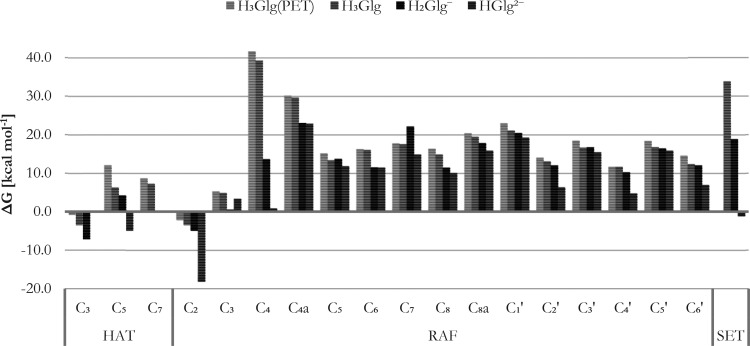
Distribution of Gibbs free energies of reaction (Δ*G*, in kcal mol^–1^, at 298.15 K) for the modeled pathways.

As evidenced from the thermochemical data presented in Figure 2 and detailed in Table S1, in the lipid solution (**H**_**3**_**Glg**^**PET**^), only two chemical pathways were identified as exergonic: *f*-HAT from the phenolic hydroxyl group at C_3_ (−0.8 kcal mol^–1^) and RAF at C_2_ (−2.2 kcal mol^–1^). For **H**_**3**_**Glg**, both *f*-HAT from the C_3_ hydroxyl group and RAF at C_2_ are equally feasible, with esteemed Gibbs free energies of −3.5 kcal mol^–1^. As subsequent deprotonation occurs, these values decrease further, reaching −7.1 kcal mol^–1^ for *f*-HAT and −4.9 kcal mol^–1^ for RAF in **H**_**2**_**Glg**^**–**^. In the case of **HGlg**^**2–**^, which lacks the C_3_ hydroxyl group, a Δ*G* value of −4.9 kcal mol^–1^ is obtained. The pronounced feasibility of hydrogen atom transfer from C_3_, compared to other hydrogen-donating sites, can be attributed to the greater degree of spin density delocalization compared to instances of C_5_ or C_7_ radicals.^29^ Considering the acidic nature of these residues, exergonicity is also somewhat influenced by the polarity of the solvent and subsequent deprotonation events.

A particularly unique aspect is the plausible thermochemistry of the radical adduct formation route involving position C_2_. The Gibbs free energies remain constantly negative, with an exceptionally low value observed for **HGlg**^**2–**^ (−18.2 kcal mol^–1^), nearly four times lower than for **H**_**2**_**Glg**^**–**^. This intriguing property is noteworthy, especially when contrasted with the sizably endergonic nature of nearly all other RAF pathways. This suggests that the ability to intercept hydroperoxyl radical could be a subject of debate from the thermochemical standpoint, highlighting the unique characteristics of the discussed route.

**Figure 3:**
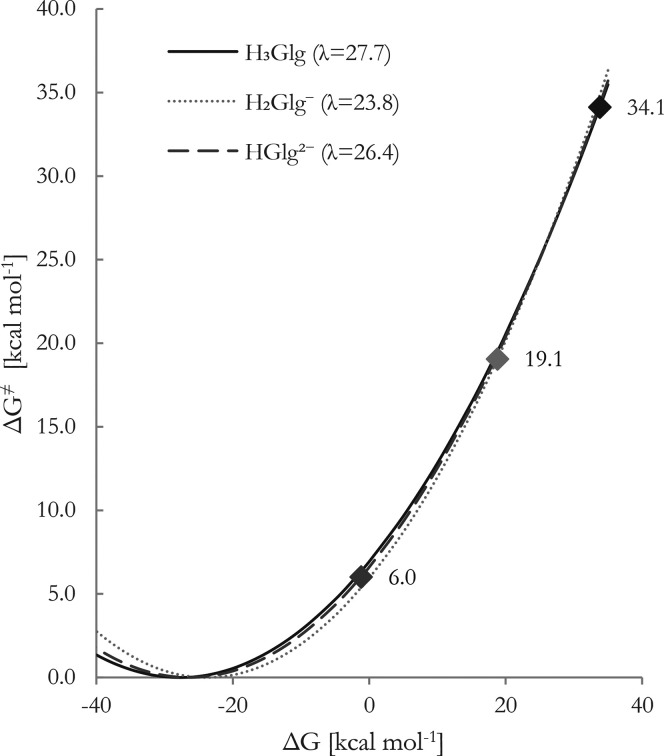
Gibbs free energies of activation (Δ*G*^⧧^) as a function of Gibbs free energies of reaction (Δ*G*). λ represents reorganization energies for the given species. The squares correspond to the pair of values. All values are in kcal mol^–1^, at 298.15 K.

Last but not least, the Δ*G* values of 33.8 kcal mol^–1^ and 18.8 kcal mol^–1^, associated with the SET mechanism from **H**_**3**_**Glg** and **H**_**2**_**Glg**^**–**^ species, respectively, may initially suggest an unfavorable nature of the process. However, caution should be exercised in dismissing these values outright. Electron transfer pathways may play a significant role in overall antiradical activity, potentially surpassing other channels. The efficacy of the mechanism hinges strongly on the established reorganization energies. To systematically explore this relationship, Marcus theory has been applied, calculating activation energies as a function of established reorganization energies and free energies, graphically represented in the Marcus parabola depicted in Figure 3.

The obtained high reorganization energies suggest a widespread of the parabola’s arms, indicating substantial structural changes during SET reactions. Additionally, the λ values imply that activation energies change more gradually as Δ*G* varies, suggesting a less pronounced impact on Δ*G*‡ is expected. The parabola’s apex is approximately −25.0 kcal mol^–1^, and all computed Δ*G* values reside on the descending arm, with the lowest datapoint at 6.0 kcal mol^–1^ (**HGlg**^**2–**^). These findings support the assertion that **Glg** species likely do not act as electron donors to ^•^OOH, disregarding the potential significance of the SET pathway in overall antiradical activity, except for **HGlg**^**2–**^. The outcomes also further stress the control of deprotonation on the electron-related mechanisms.

#### Kinetics

Not all pathways identified as endergonic were excluded from the kinetic calculations. While it is not expected that the experimentally observed products will result from these reactions, their significance may still be valid. This is especially true if subsequent processes are sufficiently exergonic, providing a driving force, and if the initial step itself is associated with a low activation energy. An example of this scenario can be the formation of radical-ionic species, as they are prone to engaging in rapid protonation/deprotonation equilibria. In the complex nature of the physiological environment with a diverse array of reacting substances, such situations may easily occur.^1,30^ Consequently, the kinetic analysis encompasses pathways labeled with positive, albeit low (<10.0 kcal mol^–1^), values of Δ*G*, recognizing their potential relevance in the overall reaction network. In contrast, electron-related processes adhere to Marcus theory, making them all worth investigating.^31−33^

Yet, before delving into the kinetic considerations, another crucial aspect must be addressed regarding the acid–base equilibrium of the hydroperoxyl radical. The ^•^OOH/O_2_^•–^ radical pair exists as part of an acid–base equilibrium with a p*K*_a_ of 4.8. In an aqueous solution at pH = 7.4, the molar fraction of ^•^OOH is only 0.0025. Its counterpart, superoxide anion radical, is abundant and carries a negative charge, making its electronic structure disinclined to acquire an additional electron. As a nucleophile and mild reducing agent, it possesses minimal impact on biological targets.^34,35^ Thereby, its protonated form is considered a primary contributor to oxidative damage,^36^ despite its significantly lower molar fraction. Consequently, to accurately replicate data under these conditions, this aspect must be taken into consideration. The rate coefficients must be so corrected following the given eq 4:
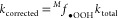
4

**Figure 4:**
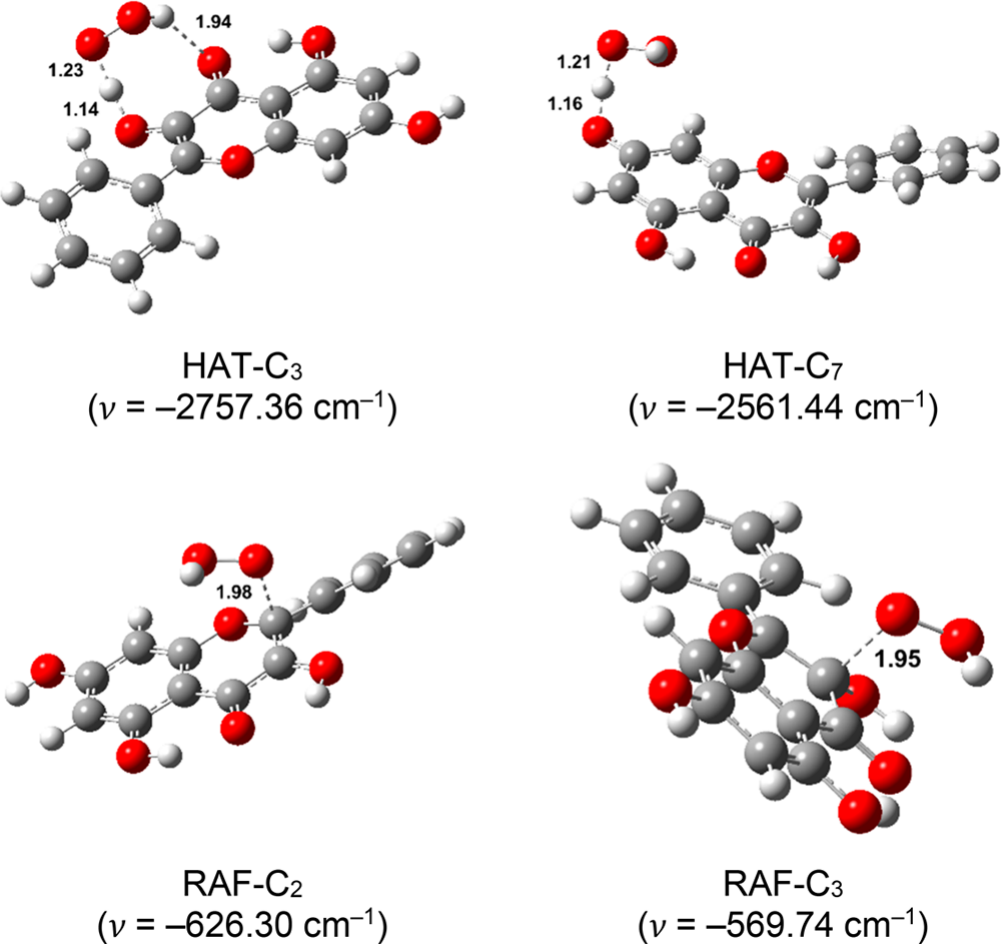
Optimized geometries of the transition states in lipid solution. Distances are reported in angstroms.

**Figure 5:**
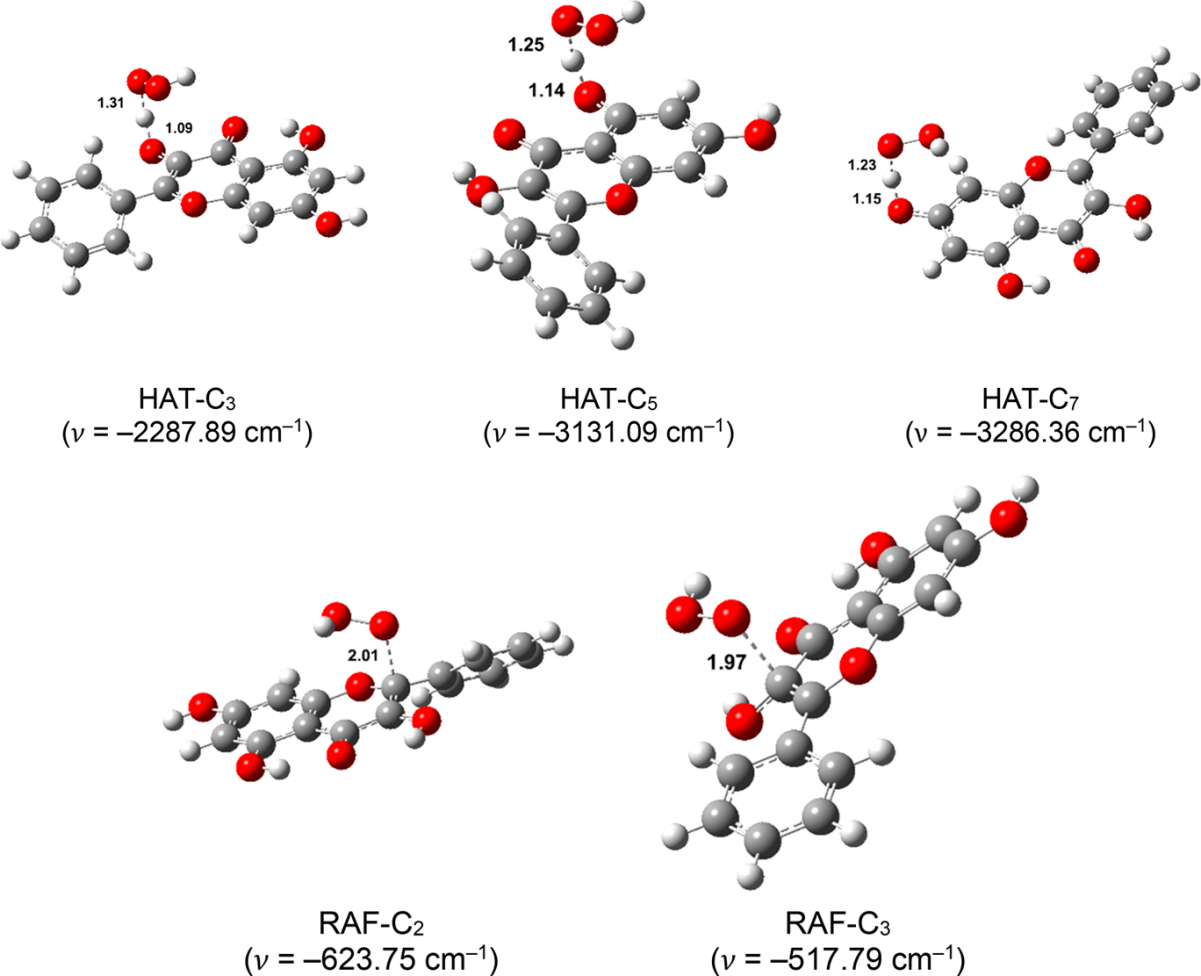
Optimized geometries of the transition states of neutral species in aqueous solution. Distances are reported in angstroms.

**Figure 6:**
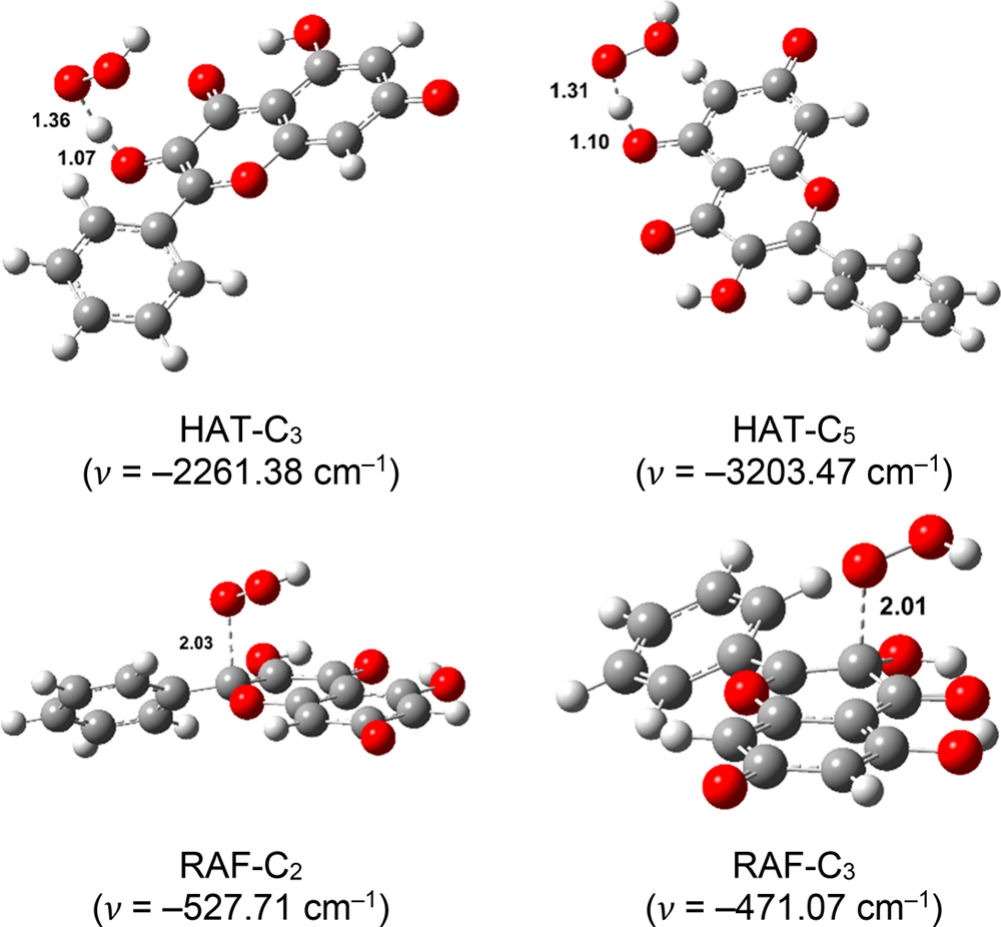
Optimized geometries of the transition states of anionic species in aqueous solution. Distances are reported in angstroms.

**Figure 7:**
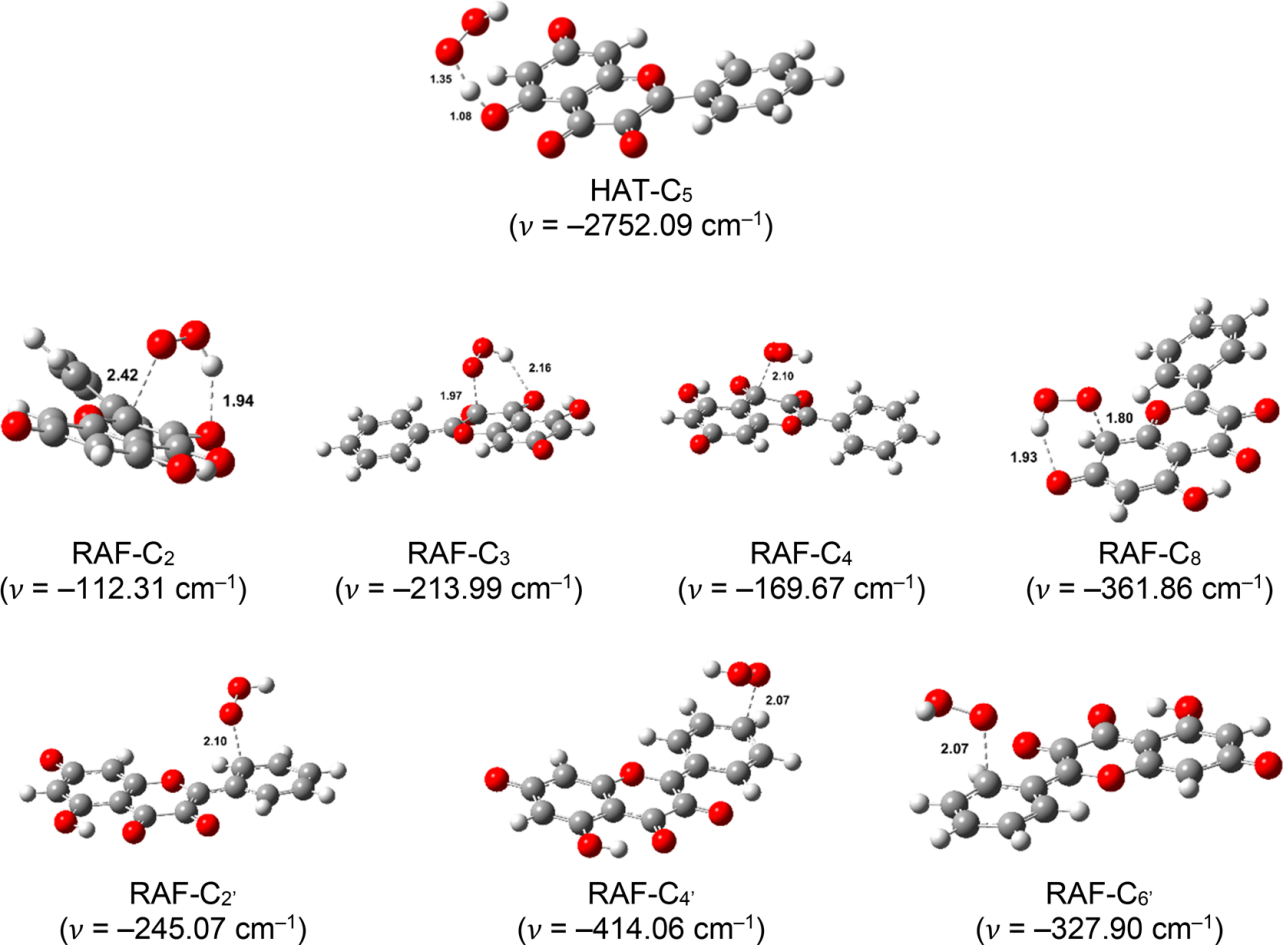
Optimized geometries of the transition states of dianionic species in aqueous solution. Distances are reported in angstrom.

The exploration of viable mechanisms is elucidated through the determination of rate constants and branching ratios. The pertinent transition state structures are depicted in Figures 4–7, accompanied by the corresponding thermochemical data detailed in Table 1.

**Table 1:** Gibbs Free Energies of Activation (Δ*G*^*≠*^, kcal mol^–1^), Rate Constants (*k*, M^–1^ s^–1^), and Branching Ratios (Γ, %) of the Reactions between Galangin Species and the Hydroperoxyl Radical in Lipid and Aqueous Solution

	H_3_Glg^PET^			H_3_Glg			H_2_Glg^–^			HGlg^2–^		
	Δ*G*^*≠*^	*k*	Γ	Δ*G*^*≠*^	*k*	Γ	Δ*G*^*≠*^	*k*	Γ	Δ*G*^*≠*^	*k*	Γ
*f*-HAT												
C_3_	16.7	3.77 × 10^3^	99.89	15.4	6.42 × 10^3^	99.30	13.8	5.04 × 10^4^	98.64			
C_5_				24.1	1.28 × 10^–1^	0.00	22.3	2.88 × 10^0^	0.01	22.4	1.08 × 10^1^	0.00
C_7_	19.8	1.73 × 10^0^	0.05	22.0	6.95 × 10^–1^	0.01						
RAF												
C_2_	17.8	8.04 × 10^–1^	0.02	15.6	3.33 × 10^1^	0.52	14.2	3.27 × 10^2^	0.64	2.3	2.50 × 10^9^	51.68
C_3_	17.3	1.75 × 10^0^	0.05	16.2	1.13 × 10^1^	0.18	14.1	3.64 × 10^2^	0.71	3.5	1.83 × 10^9^	37.92
C_4_										5.9	2.74 × 10^8^	5.68
C_8_										12.3	6.59 × 10^3^	0.00
C_2′_										15.0	7.11 × 10^1^	0.00
C_4′_										14.0	4.38 × 10^2^	0.00
C_6′_										14.6	1.42 × 10^2^	0.00
SET				34.1	5.89 × 10^–13^	0.00	19.1	6.51 × 10^–2^	0.00	6.0	2.28 × 10^8^	4.72
*k*_total_	3.77 × 10^3^			6.46 × 10^3^			5.11 × 10^4^			4.83 × 10^9^		
*k*_corrected_				8.81 × 10^0^			5.78 × 10^1^			6.21 × 10^4^		
*k*_overall_				6.21 × 10^4^								

The provided kinetic and branching ratios for the reactions in lipid media underscore the significance of the hydrogen atom transfer mechanism. To be more precise, the observed reactivity is predominantly associated with the hydroxyl group at C_3_. The notably high reaction rate constant of 3.77 × 10^3^ M^–1^ s^–1^ results in a nearly unary branching ratio, emphasizing its prevalence in scavenging the ^•^OOH radical. In contrast, the contribution of the remaining pathways, including *f*-HAT from C_7_ and RAFs at C_2_ and C_3_, to the overall activity in lipids is not greater than 0.12%. Thus, at least in this medium, the hydroxyl moiety is identified as the only one responsible for the antioxidant behavior of the investigated compound.

In an aqueous solution at physiological pH, the chemistry involved in the peroxyl radical scavenging activity of **Glg** becomes more complex. According to the calculated overall rate constants, **Glg** is predicted to react with ^•^OOH at a rate of around 6.21 × 10^4^ M^–1^ s^–1^. This is the sum of individual contributions from **H**_**3**_**Glg** (6.46 × 10^3^ M^–1^ s^–1^), **H**_**2**_**Glg**^**–**^ (5.11 × 10^4^ M^–1^ s^–1^) and **HGlg**^**2–**^ (4.83 × 10^9^ M^–1^ s^–1^). Nonetheless, while the *k*_total_ values are generally plausible, with none dropping below 10^3^ M^–1^ s^–1^, the small fraction of ^•^OOH present at this pH (∼0.25%) and the varying molar fraction of each species notably interfere with the final outcome.

The acid–base equilibria of the investigated **Glg** species exert a significant influence on the kinetics of their reactions with peroxyl radicals, thereby impacting their capability as hydroperoxyl radical scavengers. Evidently, the anti-^•^OOH activity increases with the degree of deprotonation, particularly pronounced for the single electron transfer mechanism, as expected. Furthermore, in the case of **HGlg**^**2–**^, rate constants of some RAF pathways, e.g., at C_2_ and C_4_, reach magnitudes beyond 9, being limited solely by diffusion. This rapid shift in the reaction rates underscores the consequences of considering even those species seemingly present in negligible populations under the studied conditions.

In comparison, when reacting with ^•^OOH, **H**_**3**_**Glg**^**PET**^ is approximately 140 times less efficient as an antioxidant than α-tocopherol.^37^ However, its capability to scavenge hydroperoxyl radicals in this medium is notably better than apigenin^38^ (6500 times greater rate constant) and quite similar to that of scutellarein^39^ (around 4 times greater). Shifting to a water solvent, while **Glg** (*k*_overall_ = 6.21 × 10^4)^ is notably less efficient as a scavenger than ascorbate (*k*_overall_ = 1.00 × 10^8^),^40^ but comparable to Trolox (*k*_overall_ = 8.96 × 10^4^)^41^ or already mentioned scutellarein and its glycoside — scutellarin.^42^ In both media, **Glg** exhibits much better antiradical activity than pinocembrin,^43^ other closely related flavonoid.

At this point, it is worth delving into a small discussion on the role of the hydroxyl group at C_3_ and the double bond linking C_2_ and C_3_ atoms. **Glg**, in contrast to pinocembrin, is able to exhibit its antioxidative potential in both media, regardless of the state. With the branching ratio consistently indicating the C_3_ hydroxyl group as literally the only active site, its role is undeniable and may be considered essential for good antiradical activity. Probably equally important is the presence of the C_2_=C_3_ bond, allowing for resonance with the side ring, as evidenced by Zheng et al.^44^ However, the lack of kinetic data on chrysin prevents us from making conclusive judgments. That compound is still to be studied in further submissions, just like pinobanksin, a flavonol having only a C_3_ hydroxyl group.

Only now can this assumption be made, considering that while **Glg** has an electron-withdrawing hydroxyl group, its resonance character interferes sufficiently with the conjugated systems founded on the aforementioned double bond. Thus, the single electron transfer reaction rate is over 2583 times greater than the one exhibited by pinocembrin, which is devoid of both of these features.

#### Impact of pH on Reaction Rates

**Figure 8:**
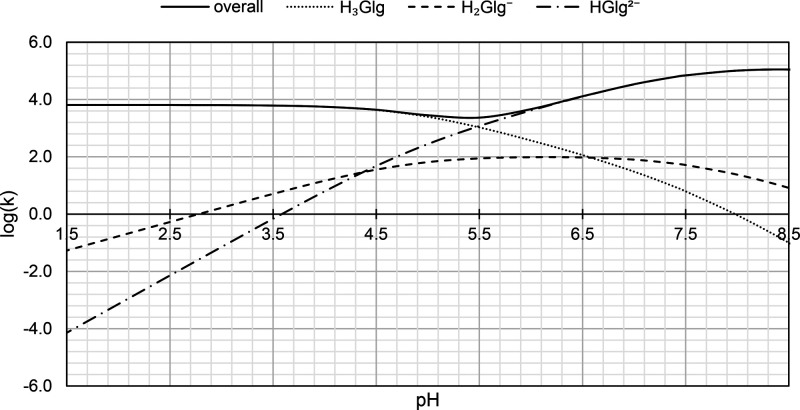
Dependence of kinetics on pH for the reactions between galangin species and hydroperoxyl radicals in aqueous solution.

Continuing the elucidation on the topic, a graph depicting the impact of pH on the overall and species-specific total rate constants is provided (Figure 8). It encompasses the pH range of 1.5 to 8.5, corresponding to the acidity found in the stomach and the slight alkalinity present in the small intestine.

The observed sum of reaction rates is primarily constituted of two forms — **H**_**3**_**Glg**, for pH values lower than around 5.0, and **HGlg**^**2–**^ for the remainder. The **H**_**2**_**Glg**^**–**^ appears to be of less significance. Generally, the overall log(*k*) value remains stable at the outset in the most acidic environments. Starting from pH ∼ 3.5, it slightly drops, and a basin can be clearly observed between the pH values of 4.5 and 6, with the minimum at around 5, associated with log(*k*) = 3.52. Thereafter, a relatively quick increase in anti-^•^OOH activity is observed, resulting from the growing concentration of **HGlg**^**2–**^ and its particular feasibility to intercept the radical.

### Antioxidative Activity Type II

Given the expectation of hydration for charged species in aqueous solutions, the computational analysis of “*free*” Cu(II) ions involved the incorporation of four water molecules within the coordination sphere, arranged in a nearly square-planar configuration.^79^ This arrangement, deemed the most probable in aqueous environments, improves the model’s accuracy in representing copper ions under physiological conditions compared to unhydrated forms. To ensure consistency, the Cu(I) models also included the same number of water molecules, despite experimental evidence supporting a linear two-coordinate structure.^80,81^ Consequently, Cu(I) is coordinated to only two water molecules, while the remaining two reside in the solvation sphere.

#### Pro-oxidant Effects

A crucial aspect of antioxidant protection, especially in the presence of metal ions, is the reductive capability of antioxidants, particularly their deprotonated species. The monoanions of antioxidants can act as nucleophilic agents, leading to the reduction of Cu(II) to Cu(I) and thereby accelerating Fenton reactions, resulting in the generation of hydroxyl radicals.^45^ This potential pro-oxidant effect was explored in this study for **Glg** and its corresponding species, illustrated by the reaction 5:
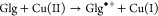
5

To provide context for the calculated data, the Cu(II) reductive activity of the investigated species was compared to that of the superoxide anion radical and the ascorbate. O_2_^•–^ and Asc^–^ are known reducing species at physiological levels; even at an experimental level, a mixture of copper-ascorbate is used to induce redox conditions, and O_2_^•–^ is the primary reducing species in Fenton reactions. The reaction, 6, is depicted as
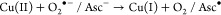
6

**Figure 9:**
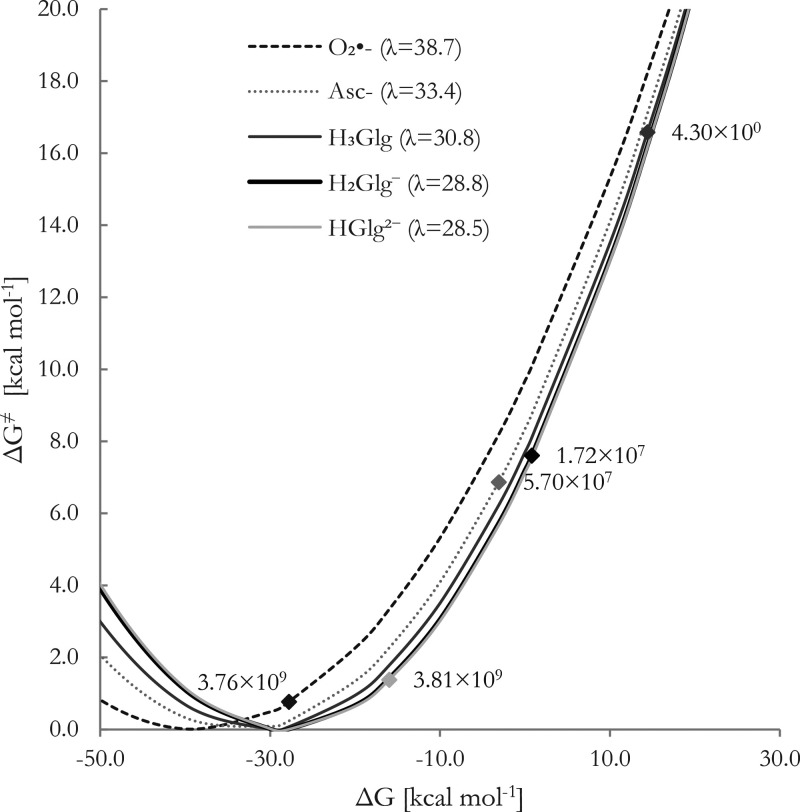
Gibbs free energies of reaction (Δ*G*, kcal mol^–1^), reorganization energies (λ, kcal mol^–1^), reaction barriers (Δ*G*^*≠*^, kcal mol^–1^), and rate constants (M^–1^ s^–1^) for the Cu(II) reduction by **Glg** species, O_2_^•–^, and Asc^–^, at 298.15 K and pH = 7.4.

In Figure 9, Marcus parabolas representing the reduction of free Cu(II) are displayed. The rate constants were determined by considering the molar fractions of **Glg**, O_2_^•–^ and Asc^–^ at the relevant pH. Considering that the experimental measurement for the reduction of Cu(II) to Cu(I) by O_2_^•–^ is reported as 8.1 × 10^9^ M^–1^ s^–1^, see ref (36), it can be inferred that the calculated rate constant for that reaction is only 2.15 times lower than the experimentally measured value. This suggests that the calculated values align closely with the experimental findings, supporting the kinetic data reported and discussed in this section.

At physiological pH, the dianionic species are expected to reduce Cu(II) to Cu(I) at significant rates (*k* = 3.81 × 10^9^ M^–1^ s^–1^). The monoanionic form exhibits a slightly lower reduction potential (*k* = 1.72 × 10^7^ M^–1^ s^–1^), which, although still substantial, is nearly 220 times and 3.4 times lower than those associated with the corresponding reductions mediated by O_2_^•–^ and Asc^–^, respectively. Consequently, **H**_**2**_**Glg**^**–**^ is not expected to exhibit significantly greater pro-oxidant behavior than the reference physiological antioxidants. Additionally, among all the species, the neutral form appears to be even less prone to undergo this process, with a reaction rate estimated to be 4.30 × 10^0^ M^–1^ s^–1^.

#### ^•^OH-Inactivating Ligand Behavior

The possibility that **Glg** behave as a ^•^OH-Inactivating Ligand (OIL) in the presence of redox metal ions was also explored. Such activity can be exhibited in two different ways:

- OIL-1: by sequestering metal ions from reductants.
- OIL-2: by deactivating ^•^OH as they are formed through Fenton-like reactions.

In both cases, OIL molecules should act as metal chelating agents. When they behave as OIL–1 agents, the metal (Cu(II)) is protected by the antioxidant in the complex formed. Thus, initially, the Fenton reactions that originate hydroxyl radicals are inhibited. Furthermore, the antioxidant behaves like OIL–2, when once the hydroxyl radical is formed by Fenton reactions, the complex of **Glg** with Cu(II) immediately reacts with the radical, thus acting as an immediate target and protecting other molecules of great interest such as proteins or even DNA.

Considering this, chelation was the first aspect explored here since it represents a necessary and crucial step in both cases. Zhao et al.^46^ have spectroscopically evaluated that two species of complexes (mono– and bis−) are produced between **Glg** with Cu(II). Furthermore, Kasprzak et al. through their literature review,^47^ have reported that most of the complexes formed between the given transition metal and studied flavonoid are of bidentate character. Therefore, the chemical routes encompassing O atoms of C_3_C_4_ and C_4_C_5_ motifs leading to the direct Cu(II) chelation into mono- and bis– complexes were studied following the general reaction schemes for monodentate (reaction 7):
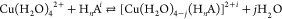
7and bidentate (reaction 8) pathway:
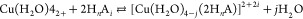
8

As evidenced from the exergonic character of the reaction (Table 2), all the sites exhibit thermochemical possibility to form Cu complexes. The C_3_C_4_ motif is the most favorable site in nearly all the cases, which in the instances of dianionic form of **Glg**, **[Cu(HGlg)**_**2**_**]** and **[Cu(HGlg)**_**2**_**]**^**2–**^, is associated with a unitary molar fraction. An exception to this pattern, however, can be found in **[Cu(H**_**2**_**Glg)**_**2**_**]** where chelation is thermodynamically preferred at the C_4_C_5_ site (0.85 vs. 0.15).

**Table 2:**
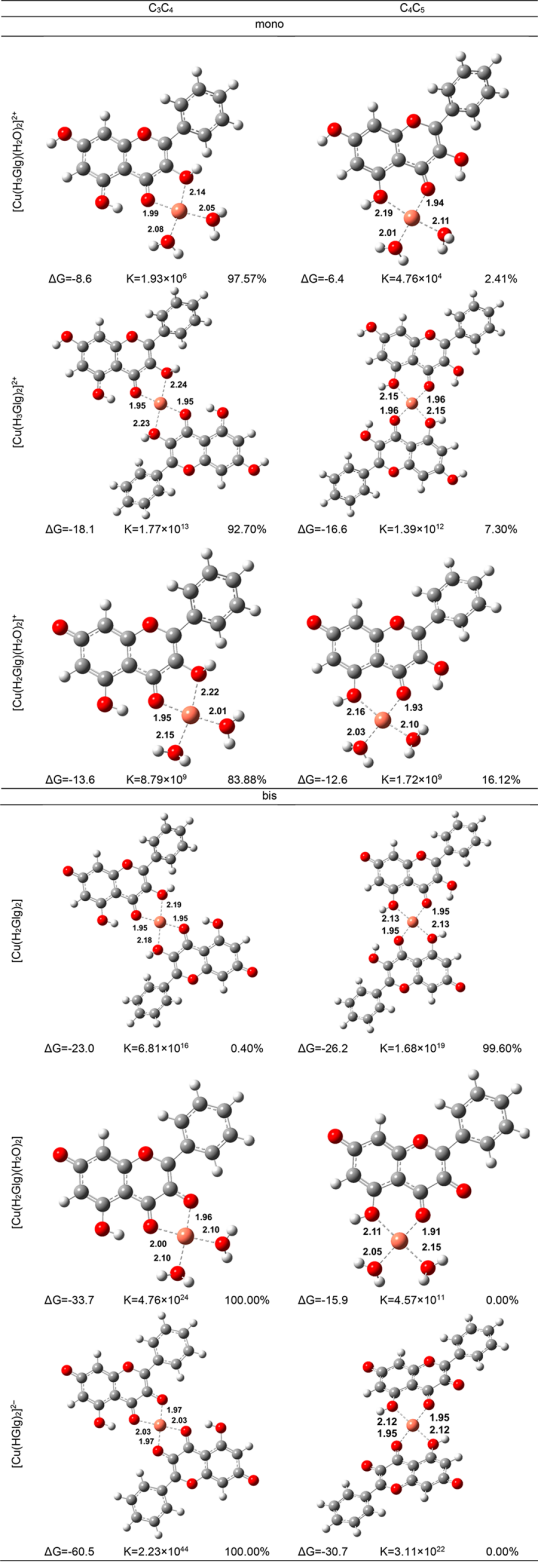
Gibbs Free Energies of Complexation (in kcal mol^–1^), Apparent Equilibrium Constants, and Maxwell–Boltzmann Distribution (%) at pH = 7.4

#### OIL-1

Similar to the previous antioxidative activities, the OIL-1 type properties were explored for forms with non-negligible populations (the molar fraction greater than 0.1%), consistent with previous papers^48−50^.. Gibbs free energies of reaction and reaction rates for the reduction of the complexes with O_2_^•–^ and Asc^–^, along with the same reaction for the reference Cu(H_2_O)_4_^2+^ are given in Table 3.

**Table 3:** Energies of Reactions (Δ*E*, kcal mol^–1^), Gibbs Free Energies of Reaction (Δ*G*, kcal mol^–1^), Reorganization Energies (λ, kcal mol^–1^), Activation Energies (Δ*G*^≠^, kcal mol^–1^), and Rate Constants (*k*, M^–1^ s^–1^), for the Reactions of the Complexes with the Reductants O_2_^•–^ and Asc^–^, in Aqueous Solution at 298.15 K and pH = 7.4^a^

		O_2_^•–^					Asc^–^				
		Δ*E*	Δ*G*	λ	Δ*G*^*≠*^	*k*	Δ*E*	Δ*G*	λ	Δ*G*^*≠*^	*k*
Cu(H_2_O)_4_^2+^		1.6	–27.8	38.7	0.8	3.76 × 10^9^	22.8	–3.1	33.4	6.9	5.70 × 10^7^
mono											
[Cu(H_3_Glg)(H_2_O)_2_]^2+^	C_3_C_4_	–1.3	–28.3	35.7	0.4	↑ 4.07 × 10^9^	19.9	–3.7	29.8	5.7	↑ 3.63 × 10^8^
	C_4_C_5_	–1.6	–27.8	34.8	0.4	↑ 4.05 × 10^9^	19.6	–3.2	28.9	5.7	↑ 3.47 × 10^8^
[Cu(H_2_Glg)(H_2_O)_2_]^+^	C_3_C_4_	–0.8	–26.3	34.3	0.5	↑ 3.98 × 10^9^	20.4	–1.7	28.5	6.3	↑ 1.45 × 10^8^
	C_4_C_5_	1.9	–25.2	35.8	0.8	↑ 4.06 × 10^9^	23.2	–0.6	29.9	7.2	↓ 3.29 × 10^7^
[Cu(HGlg)(H_2_O)_2_]	C_3_C_4_	15.3	–18.5	42.5	3.4	↓ 3.33 × 10^9^	36.5	6.2	36.7	12.5	↓ 4.19 × 10^3^
bis											
[Cu(H_3_Glg)_2_]^2+^	C_3_C_4_	–6.3	–26.8	29.0	0.0	↑ 4.35 × 10^9^	14.9	–2.1	22.8	4.7	↑ 1.43 × 10^9^
	C_4_C_5_	–3.8	–26.2	30.9	0.2	↑ 4.28 × 10^9^	17.4	–1.5	24.8	5.5	↑ 5.34 × 10^8^
[Cu(H_2_Glg)_2_]	C_4_C_5_	2.2	–19.1	29.9	1.0	↑ 4.31 × 10^9^	23.4	5.5	23.7	9.0	↓ 1.51 × 10^6^
[Cu(HGlg)_2_]^2–^	C_3_C_4_	26.8	–1.7	37.1	8.4	↓ 4.12 × 10^6^	48.0	22.9	31.0	23.4	↓ 4.15 × 10^–5^

a Arrows up (↑) and down (↓) indicate whether the reaction rate is greater or lower, respectively, than the reference reaction with the “free” ion.

The methodology applied was verified by reference to the experimental reaction rate between copper and the superoxide radical, measured by Butler et al.^51^ as (8.1 ± 0.5) × 10^9^ M^–1^ s^–1^ at pH = 7.0 and by Brigelius et al.^52^ as (2.7 ± 0.2) × 10^9^ M^–1^ s^–1^ at pH = 7.8. The established rate of 3.76 × 10^9^ M^–1^ s^–1^ at pH 7.4 lies in between the values reported by the two studies, implying that the data presented are accurate and reliable.

The outcomes underline several important aspects in terms of OIL-1 activity. First, the superoxide radical appears as a particularly strong reductant, as only dianionic species — **[Cu(HGlg)**_**2**_**]**^**2–**^, and to a very tiny extent also **[Cu(H**_**2**_**Glg)(H**_**2**_**O)**_**2**_**]** — yielded *k* values lower than the “free ion”. These are 4.12 × 10^6^ M^–1^ s^–1^ and 3.33 × 10^9^ M^–1^ s^–1^, respectively, vs 3.76 × 10^9^ M^–1^ s^–1^. For the remainder, the reaction seems to undergo even more readily, and hence the pro-oxidative character, at least in terms of this kind of activity, is evident.

When it comes to Asc^–^, much more plausible results are obtained. In this case, both dianionic species significantly lower the corresponding reaction rates, with **[Cu(HGlg)**_**2**_**]**^**2–**^ literally inhibiting the process. **[Cu(H**_**2**_**Glg)(H**_**2**_**O)**_**2**_**]**^**+**^ and **[Cu(H**_**2**_**Glg)**_**2**_**]** have only slightly altered the reaction rate, approximately 38-fold in the former and around 2-fold in the latter case.

Concluding, the data prove that only bis-complex built upon dianionic species is capable of strongly alleviating the process of Cu(II) reduction in either case, while its monocomplex only does so in the instance of ascorbate. Yet, these forms can be considered as good OIL-1 agents.

#### OIL-2

To further enhance the research outcomes, the possibility of ligand-chelated copper being involved in the scavenging of hydroxyl radical was studied. This part involved three mechanisms already discussed in terms of antiradical activity, that may contribute to this desired behavior, namely, single electron transfer, formal hydrogen atom transfer, and radical adduct formation.

To begin with, the SET mechanism was examined with the results provided in Table 4. Although hydroxyl radicals are generally recognized to react rapidly, at the diffusion limit with any structure in their vicinity, the rate constant of electron transfer from neutral species does not reflect this. For the electron-related processes, which are strongly based on the Marcus theory, it can be asserted that the corresponding data points would fall close to or even within the inverted region of the parabola, especially when considering **[Cu(H**_**3**_**Glg)**_**2**_**]**^**2+**^, for which the reaction rates are particularly low. On the other hand, the ionic forms scavenge the radical with a magnitude of 9. This is not unexpected, as it has been evidenced several times that deprotonation of flavonoids enhances the propensity of electron-related mechanisms. Apparently, this trait can be also observed in the metal complexes they make. Looking at the neutral forms, two other observations emerge: firstly, greater reaction rates are observed if the Cu(II) is coordinated by the C_4_C_5_ motif rather than C_3_C_4_ (1.18 × 10^7^ M^–1^ s^–1^ vs 1.32 × 10^5^ for **[Cu(H**_**3**_**Glg)(H**_**2**_**O)**_**2**_**]**^**2+**^; 1.61 × 10^1^ vs 1.07 × 10^–3^ for **[Cu(H**_**3**_**Glg)**_**2**_**]**^**2+**^), and secondly, mono–complexes react much faster than bis–complexes.

**Table 4:** Energies of Reactions (Δ*E*, kcal mol^–1^), Gibbs Free Energies of Reaction (Δ*G*, kcal mol^–1^), Reorganization Energies (λ, kcal mol^–1^), Activation Energies (Δ*G*^≠^, kcal mol^–1^), and Rate Constants (*k*, M^–1^ s^–1^), for the SET Pathways of the Complexes with the ^•^OH, in Aqueous Solution at 298.15 K and pH = 7.4

		Δ*E*	Δ*G*	λ	Δ*G*^*≠*^	*k*
mono						
[Cu(H_3_Glg)(H_2_O)_2_]^2+^	C_3_C_4_	15.2	10.0	15.1	10.5	1.32 × 10^5^
	C_4_C_5_	10.7	7.0	13.6	7.8	1.18 × 10^7^
[Cu(H_2_Glg)(H_2_O)_2_]^+^	C_3_C_4_	–3.8	–5.1	11.2	0.8	4.15 × 10^9^
	C_4_C_5_	–5.2	–7.1	11.8	0.5	4.27 × 10^9^
[Cu(HGlg)(H_2_O)_2_]	C_3_C_4_	–14.0	–16.8	12.7	0.3	4.16 × 10^9^
bis						
[Cu(H_3_Glg)_2_]^2+^	C_3_C_4_	18.4	19.0	9.3	21.5	1.07 × 10^–3^
	C_4_C_5_	14.4	15.0	9.3	15.8	1.61 × 10^1^
[Cu(H_2_Glg)_2_]	C_4_C_5_	–1.1	–0.6	9.4	2.1	4.49 × 10^9^
[Cu(HGlg)_2_]^2–^	C_3_C_4_	–13.6	–12.8	9.1	0.4	4.57 × 10^9^

**Figure 10:**
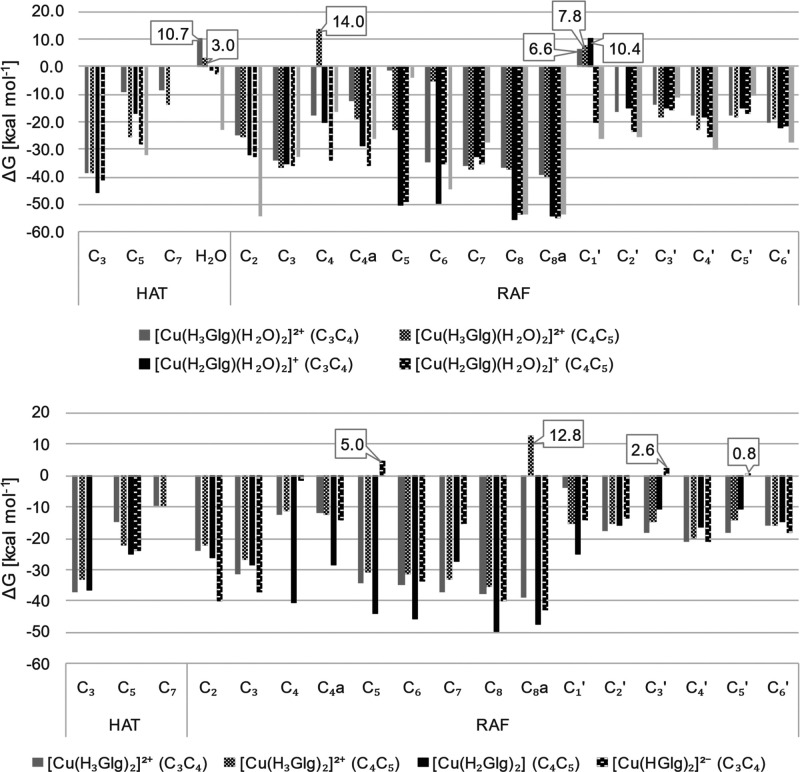
Gibbs free energies of reaction (Δ*G*, kcal mol^–1^) for *f*-HAT RAF pathways of the mono- (upper) and bis- (lower) complexes with the ^•^OH, in aqueous solution at 298.15 K and pH = 7.4.

The compiled data on *f*-HAT and RAF feasibility are plotted on Figure 10. It may be expected that ^•^OH reactions obey the Bell-Evans–Polanyi principle,^53,54^ which states that the most exergonic processes have the lowest activation energies and are thus kinetically favored, implicitly assumed to be followed. While such a statement must be primarily verified, the already conducted research regarding OIL-2 activity verifies its applicability. Therefore, the data presented herein are not associated with explicit kinetic rate constants because if the reaction is going to take place, it is are likely to undergo with *k* beyond the diffusion limit, preventing the rationalization of the results. Instead, only Gibbs free energies have been considered.

While almost all the values are exergonic, there are several for which Δ*G* is more or less positive.

Initially, *f*-HAT pathways from the aromatic hydroxyls and water molecules within the coordination sphere of Cu(II) were examined. The outcomes reveal an overall thermodynamically favorable character of the mechanism for almost all reactions are exergonic. The only exception is *f*-HAT from the coordinating water molecule in **[Cu(H**_**3**_**Glg)(H**_**2**_**O)**_**2**_**]**^**2+**^, where Δ*G* has been found slightly positive (3.0 kcal mol-1). Site C_3_ stands out as particularly promising, consistently associated with the lowest Δ*G* values, not exceeding but also they are not greater than −33.0 kcal mol^–1^ (as seen in the instance of the complex with the C_4_C_5_ motif of **[Cu(H**_**3**_**Glg)**_**2**_**]**^**2+**^). The trend shifts to C_5_, C_7_ sites,and finally to the coordinating water molecule.

Additionally, an observation regarding the impact of coordination on the exergonic character of the reaction can be made. The electron-withdrawing nature of the metal center leads to a reduction of Gibbs free energies of reaction. For instance, in **[Cu(H**_**3**_**Glg)(H**_**2**_**O)**_**2**_**]**^**2+**^, the Δ*G* is −9.4 kcal mol^–1^ for the C_5_ hydroxyl when Cu(II) is chelated by the C_3_C_4_ motif. However, if C_4_C_5_ participates in a formation of the complex, Δ*G* drops to −25.5 kcal mol^–1^. This observation is similarly applicable to **[Cu(H**_**2**_**Glg)(H**_**2**_**O)**_**2**_**]**^**+**^. Conversely, the effect is less pronounced for the bis complex, **[Cu(H**_**3**_**Glg)**_**2**_**]**^**2+**^ , with a difference of around 4.1 kcal mol^–1^. Furthemore, similar to the previous remarks, deprotonation renders the process, from the thermochemical standpoint, more favorable.

An examination of the RAF mechanism involving all the possible sites on the aromatic rings is to be briefly presented. One can see that, similarly to *f*-HAT, most of the reactions are strongly exergonic, with several exceptions listed for which Δ*G* is positive. Visibly, the formation of radical adducts at the B-ring is less preferable than in the AC system, which participates in chelate formation.

Ultimately, it appears that all of the complexes are capable of efficiently scavenging OH radicals. Such chemical properties are relevant not only by considering that not all the species can intercept their formation, so they will at least be able to scavenge them, but also to indicate that some species, namely the dianionic, can “double” their antioxidative effects, either preventing OH radicals’ formation or scavenging those already produced. Most importantly, the outcomes are strongly supported by the experimental data.^55^

### Antioxidative Activity Type III

Aside from the presented behaviors, antioxidants may also interfere with biological targets and repair them following hydrogen atom or electron transfer reactions. Three different kinds of biological targets were considered here, namely, lipids, proteins, and DNA. The models used to represent them are proposed and described in detail in the CADMA-Chem protocol^56^ and consist of:

(1) 9:2Δ^3,6^ carboxyl acid (LM), representing a simplified model of linoleic acid, which maintains its most important chemical feature (two allylic H atoms);
(2) N-formylated amides of amino acids, used to represent residues in proteins that are particularly susceptible to oxidative stress, such as leucine (NF-Leu), cysteine (NF-Cys), methionine (NF-Met), tyrosine (NF-Tyr), histidine (NF-His), and tryptophan (NF-Trp).^57^
(3) 2′-deoxyguanosine (2dG) as the most easily oxidized of the nucleobases^58^ — if a chemical agent oxidizes 2dG, then it can also cause oxidative damage to DNA. In contrast, if such an agent does not oxidize 2dG, then it is expected to be innocuous to DNA.

Thermochemical and kinetic data associated with these processes are presented in Table 5, complemented by structures of the corresponding transition states depicted in Supporting Information, Figures S3–S5.

**Table 5:** Gibbs Free Energies of Reaction (Δ*G*, kcal mol^–1^), Activation Energies (Δ*G*^≠^, kcal mol^–1^), and Rate Constants (*k*, M^–1^ s^–1^) for the Regeneration Pathways of the **Glg** Species with the Relevant Biomolecules, at 298.15 K and pH = 7.4

	H_3_Glg			H_2_Glg^–^			HGlg^2–^		
	Δ*G*	Δ*G*^*≠*^	*k*_*i*_	Δ*G*	Δ*G*^*≠*^	*k*_*i*_	Δ*G*	Δ*G*^*≠*^	*k*_i_
LM^•^									
C_3_	11.4								
C_5_	24.3								
C_7_	20.8								
*k*_total_									
*k*_overall_									
2dG^•^									
C_3_	–10.9	19.5	1.70 × 10^1^	–14.2	18.3	1.56 × 10^2^			
C_5_	–1.1	28.0	1.33 × 10^–4^	–3.0	27.3	5.62 × 10^–4^	–12.3	27.4	2.36 × 10^–4^
C_7_	–0.2	22.9	1.18 × 10^–2^						
*k*_total_	1.70 × 10^1^			1.56 × 10^2^			2.36 × 10^–4^		
*k*_overall_	9.23 × 10^0^			7.04 × 10^1^			1.21 × 10^–6^		
[2dG-H]^•+^									
	3.2	6.8	6.15 × 10^7^	–11.7	0.3	3.71 × 10^9^	–31.9	2.2	3.62 × 10^9^
*k*_overall_	3.34 × 10^7^			1.67 × 10^9^			1.86 × 10^7^		
NF-Cys^•^									
C_3_	–0.7	19.3	9.69 × 10^0^	–4.1	17.5	4.41 × 10^1^			
C_5_	9.0	26.7	2.07 × 10^–3^	7.2	18.7	5.48 × 10^–1^	–2.1	5.1	7.52 × 10^8^
C_7_	9.9	30.8	3.00 × 10^–10^						
*k*_total_	9.69 × 10^0^			4.46 × 10^1^			7.52 × 10^8^		
*k*_overall_	5.27 × 10^0^			2.01 × 10^1^			3.86 × 10^6^		
NF-His^•^									
C_3_	–5.9	22.3	1.15 × 10^–1^	–9.3	20.9	1.31 × 10^1^			
C_5_	3.8	30.5	3.02 × 10^–7^	2.0	28.9	5.36 × 10^–6^	–7.3	19.4	9.29 × 10^0^
C_7_	4.7	30.1	1.19 × 10^–7^						
*k*_total_	1.15 × 10^–1^			1.31 × 10^0^			9.29 × 10^0^		
*k*_overall_	6.25 × 10^–2^			5.93 × 10^–1^			4.72 × 10^–2^		
NF-Leu^•^									
C_3_	–9.2	17.9	4.18 × 10^1^	–12.6	17.7	9.15 × 10^1^			
C_5_	0.5	26.0	8.90 × 10^–5^	–1.3	29.7	7.11 × 10^–7^	–10.6	26.4	4.99 × 10^–4^
C_7_	1.4	24.4	1.75 × 10^–4^						
*k*_total_	4.18 × 10^1^			9.15 × 10^1^			4.99 × 10^–4^		
*k*_overall_	2.27 × 10^1^			4.13 × 10^1^			2.56 × 10^–6^		
NF-Met^•^									
C_3_	–7.9	16.3	9.61 × 10^1^	–11.3	16.6	7.29 × 10^1^			
C_5_	1.8	31.0	3.36 × 10^–9^	0.0	31.3	9.81 × 10^–8^	–9.4	31.8	3.29 × 10^–7^
C_7_	2.7	18.5	7.06 × 10^–1^						
*k*_total_	9.68 × 10^1^			7.29 × 10^1^			3.29 × 10^–7^		
*k*_overall_	5.26 × 10^1^			3.29 × 10^1^			1.69 × 10^–9^		
[NF-Trp-H]^•+^									
	11.1	11.8	1.35 × 10^4^	–3.9	1.9	3.66 × 10^9^	–23.9	0.7	3.71 × 10^9^
*k*_overall_	7.34 × 10^3^			1.65 × 10^9^			1.90 × 10^7^		
NF-Tyr^•^									
C_3_	–2.5	15.1	3.76 × 10^3^	–5.9	16.3	2.52 × 10^2^			
C_5_	7.2	23.8	7.68 × 10^–4^	5.4	21.0	8.78 × 10^–2^	–3.9	2.5^a^	1.77 × 10^9^
C_7_	8.2	18.7	1.45 × 10^–1^						
*k*_tot_	3.76 × 10^3^			2.52 × 10^2^			1.77 × 10^9^		
*k*_overall_	2.04 × 10^3^			1.02 × 10^2^			9.05 × 10^6^		
[NF-Tyr-H]^•+^									
	1.3	5.5	5.18 × 10^8^	–13.6	0.0	3.71 × 10^9^	–33.6	3.6	2.92 × 10^9^
*k*_overall_	2.81 × 10^8^			1.67 × 10^9^			1.50 × 10^7^		

a The value is not fully converged. The transition state appears not to exist or to be below the displacement potential, suggesting that the system is not bound. An ansatz for the transition state has been chosen as the maximum from the full IRC scan obtained through frequency calculations.

The results indicate that from a thermochemical standpoint, **Glg** is unlikely to restore oxidatively damaged lipids. As evidenced for the model linoleic acid, the Gibbs free energies of hydrogen transfer are above the imposed threshold, assessed at 11.4 kcal mol^–^1 for C_3_, 24.3 kcal mol^–1^ for C_5_, and 20.8 kcal mol^–1^ for C_7_ hydroxyl. On the other hand, **Glg** exhibits its type III antioxidative activity broadly when interacting with amino acids and nucleobases. All reactions were found to be below 10 kcal mol^–1^ and thus have all been studied from a kinetic standpoint.

From the output, it can be deduced that the relative activity of hydroxyl groups in hydrogen atom transfer is constituted by C_3_ > C_7_ > C_5_, at least concerning Gibbs free energies of reactions. Without delving into activation energies and reaction rates, the incorrect assumption of such a pattern being representative of reactivity may be constructed. However, one can actually observe that while *f–*HAT from C_5_ exhibits greater exergonic character than C_7_, the activation energies are clearly higher for the former than the latter, impacting reaction rates significantly.

Another observation worth underlining is the ambiguous effect of deprotonation: in the previous paper on pinocembrin,^43^ it has been evidenced to enhance activity in all the examples studied. Here, however, it is vague, and for example, while NF-Cys^•^ is restored moderately by hydrogen transfer from C_5_ of **H**_**3**_**Glg** (*k* = 2.07 × 10^–3^ M^–1^ s^–1^) or **H**_**2**_**Glg**^**–**^ (*k* = 5.48 × 10^–1^ M^–1^ s^–1^), in the case of **HGlg**^**2–**^, the rate of the process extremely increases, up to 7.52 × 10^8^ M^–1^ s^–1^. Similar situations are evident for NF-His^•^ or NF-Tyr^•^. On the other hand, similarly dramatic shifts are not found for 2dG^•^, NF-Leu^•^, or NF-Met^•^. Position C_5_ has been chosen for reference due to the particularly strong bond between hydrogen and oxygen of the hydroxyl residues, further stabilized by the electron density of the carbonyl group at C_4_.^29^ When it comes to electron-related processes, the aforementioned trend is not visible at all, and the observed increasing propensity of the electron transfer reaction follows the subsequent deprotonations.

#### Regeneration

Antioxidants typically lose their scavenging ability after neutralizing a free radical. However, in biological systems, they can be regenerated with the help of other antioxidants like glutathione, vitamin C, or vitamin E. Yet, in an oxidatively stressed environment, their concentrations may become depleted. The superoxide anion radical, abundant at physiological pH, might play a role in mediating this renewal process. It could follow pathways of reduction of cation radical (reaction 9) or radical (reaction 10), and subsequent protonation (reaction 11), as follows:
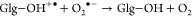
9
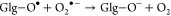
10
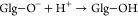
11Table 6 sheds light on the regeneration dynamics of **Glg** species, offering insights into the energetics and kinetics of their interactions with hydroperoxyl radicals. Remarkably, irrespective of the protonation state and the involved residues, the regeneration process proves feasible, with consistently negative Gibbs free energies. While viability decreases gradually with each successive deprotonation step, the process remains plausible, with none of the processes being endergonic.

**Table 6:** Gibbs Free Energies of Reactions (Δ*G*, kcal mol^–1^), Gibbs Free Energies of Activation (Δ*G*^≠^, kcal mol^–1^), Rate Constants (*k*, M^–1^ S^–1^), and Gibbs Free Energies of Protonation (Δ*G*^+^, kcal mol^–1^) at 298.15 K for the Regeneration Process

	Δ*G*	Δ*G*^*≠*^	*k*	Δ*G*^+^
**H**_**3**_**Glg**				
C_3_	–20.1	1.3	4.00 × 10^9^	–32.6
C_5_	–29.6	0.8	3.96 × 10^9^	–32.8
C_7_	–34.2	0.7	3.98 × 10^9^	–29.2
SET	–49.2	3.6	3.98 × 10^9^	
**H**_**2**_**Glg**^**–**^				
C_3_	–13.9	0.7	3.45 × 10^9^	–35.1
C_5_	–21.3	0.1	3.83 × 10^9^	–39.1
SET	–34.2	1.0	3.98 × 10^9^	
**HGlg**^**2–**^				
C_5_	–7.5	3.6	3.09 × 10^9^	–43.8
SET	–14.2	1.6	3.98 × 10^9^	

Furthermore, the calculated activation energies, capped at 3.6 kcal mol^–1^ (notably in the cases of C_5_ of **HGlg**^**2–**^ and SET from **H**_**3**_**Glg**^**–**^), suggest rapid reactions limited by diffusion. This implies that, if left not intercepted by the surrounding environmental factors, these reactions could perpetuate a self-sustaining cycle of regeneration and scavenging activity. The significance of this is further underscored by the consistently strongly negative energies of protonation from the solvent for all species.

As a consequence of all the mentioned, the dianionic species is a particularly efficient agent when it comes to scavenging radicals and recovering damaged biomolecules.

## Conclusions

Based on the results obtained, the following conclusions regarding the antioxidative activity of galangin can be drawn:

- Under physiological conditions, **Glg** is primarily present in its neutral and monoionic forms. Although the dianion is present to a minor extent, it should not be disregarded. Kinetic data suggests that even species present in small fractions may play a pivotal role in accurately examining antioxidative activity.
- According to the eH-DAMA analysis, the neutral species appears to possess similar antiradical activity in pentylethanoate and water. However, its potential might be revealed as deprotonation occurs. These preliminary outcomes are consistent with more detailed analyses provided in the following section, suggesting that eH-DAMA could be considered a useful tool for preliminary assessment and eventual assertions prior to regular study.
- The overall reaction rates have been established 3.77 × 10^3^ M^–1^ s^–1^ in lipid medium and 6.21 × 10^4^ M^–1^ s^–1^ in water, highlighting moderate antiradical activity toward OOH radical. It is worth mentioning that the log(*k*_*overall*_) value falls within 3.5 and 5 across the entire range of physiologically relevant pH implicating stability of this characteristic.
- Two of the three present forms of **Glg** readily reduce Cu(II) to Cu(I) before chelating it. **HGlg**^**2–**^ demonstrates stronger reductive capacity than O_2_^•–^ and Asc^–^, while **H**_**2**_**Glg**^**–**^ exhibits a similar potential to ascorbate. In contrast, the neutral form shows negligible harmful activity.
- When complexed by the antioxidant, Cu(II) is less prone to undergo reduction, particularly in the instances of **[Cu(HGlg)**_**2**_**]**^**2–**^ and **[Cu(H**_**2**_**Glg)(H**_**2**_**O)**_**2**_**]** when it comes to O_2_^•–^. Additionally, in addition to the aforementioned, **[Cu(H**_**2**_**Glg)(H**_**2**_**O)**_**2**_**]**^**+**^ and **[Cu(H**_**2**_**Glg)**_**2**_**]** exhibit this property also when considering Asc^–^ and the C_4_C_5_ chelation site.
- Hydroxyl radicals formed during the Fenton’s reaction on the already complexed copper are efficiently neutralized by most of the chelates, as evidenced by the plausible thermodynamics of these processes.
- Galangin can be labeled as an efficient repair agent, especially concerning [2dG-H]^•+^, NF-Cys, [NF-Trp-H]^•+^, NF-Tyr^•^ or [NF-Tyr-H]^•+^. It can marginally restore other amino acids but ultimately is not capable of fixing oxidatively damaged lipids.
- The reduction of ″*used*″ antioxidant by O_2_^–•^, prevalent at physiological pH, renders **Glg** a particularly potent AOX-I and AOX-III agent.

In summary, this research not only contributes to understanding galangin antioxidant activity but also underscores crucial aspects contributing to overall efficacy. Further experimental validations and applications of the methodology can enhance knowledge of such systems, elucidating the beneficial activity of food products.

## Computational Methods

The low-energy ground-state conformer of neutral galangin was systematically generated using a robust conformer search procedure that combines metadynamic sampling and z-matrix genetic crossing, specifically the iMTD-GC method implemented in the CREST driver program.^59^

Electronic structure calculations in this study were performed using the Gaussian 16 (rev. C.01) software package.^60^ For geometry optimizations and frequency calculations, the density functional theory (DFT) approach was employed, depending on the specific type of antioxidant activity investigated. Further details can be found in the subsequent paragraphs and Supporting Information.

Solvation effects were integrated into the study using the universal solvation model based on solute electron density (SMD),^61^ with pentyl ethanoate (ε = 4.73^62^) and water (ε = 78.35^62^) selected to replicate physiological conditions in cellular environments. The selection of SMD was based on its demonstrated suitability for simulating solvents with diverse characteristics and media, whether charged or noncharged.^61^ Notably, SMD has proven effective in mixed models and has been successfully applied for geometry optimization and vibrational calculations in solution settings. Empirical validation for a wide range of solutes and liquid environments further supports its appropriateness.^63^

Unrestricted calculations were specifically employed for open-shell systems in this study. In all instances, deviations in spin values from the ideal were negligible after the elimination of the initial spin contamination. The identification of local minima depended on the absence of imaginary frequencies, while transition states were discerned through the presence of a single frequency precisely corresponding to the anticipated motion along the reaction coordinate. Additionally, the accuracy of the identified structures was confirmed through Intrinsic Reaction Coordinate (IRC) computations^64,65^ providing assurance that the calculated transition states appropriately connected with the reactants and products of the intended reaction. This reinforces the reliability of the theoretical predictions.

The detailed computational methodology encompassing acid–base equilibria, thermochemistry and kinetics is presented in the Supporting Information. The discussion on the dissociation constants, molar fractions (Figure S1) and relative reactivity (Figure S2) is given there too.

### Antioxidant Activity Type I

Antiradical activity was investigated using the hybrid meta exchange-correlation functional M05-2X. This choice was based on its proficiency in addressing noncovalent interactions, kinetics, and thermochemistry, as evidenced by extensive validation against barrier heights, conformational energy, and bond dissociation energies.^66^ Furthermore, M05-2X has demonstrated efficacy in modeling open-shell systems, particularly in estimating energies associated with reactions involving free radicals.^67^ It stands out as one of the top-performing DFT approximations, alongside LC-xPBE, M06-2X, BMK, B2PLYP, and MN12SX, based on a benchmark study assessing rate constant calculations for radical molecule reactions in aqueous solutions.^68^ An additional reason for choosing M05-2X over M06-2X, which was also tested by us in a broader scope,^69^ was previously mentioned in the original QM-ORSA article^40^ where the authors referred to the papers of Biczysko et al.^70^ and Mangiatordi et al.^71^ indicating that the newer version “[ . . .] *does not necessarily provide excellent results for harmonic vibrations*, *and also that it may produce high frequencies for proton transfer reactions*.“

Regarding the choice of basis set, Pople’s 6-311+G(d,p)^72,73^ has been selected due to its well-balanced compromise between computational resources uptake. Galano et al.^40,68^ thoroughly assessed a similar combination, akin to the one utilized in this study, to evaluate the antiradical activity of several antioxidants operating through electron transfer or hydrogen transfer mechanisms, including polyphenolic antioxidants. Their findings underscored the adequacy of both experimental and computational data, further validating the results presented herein.

### Antioxidant Activity Type II

The computations were performed using the M05 functional^66^ chosen for its parametrization that includes both metals and nonmetals, in contrast to M05-2X. Furthermore, M05 performs well not only for main-group thermochemistry but also for interactions with transition metals. Pople’s 6-311+G(d,p) basis set was selected for s- and p-block elements, and the SDD valence basis set and pseudopotentials^74,75^ were used for metal centers to avoid the need to describe relativistic effects in deep core electrons.

The ability to chelate the ion, as denoted by the apparent equilibrium constant (*K*^app^), was evaluated using the eq 12:
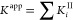
12where *K*_*i*_^*II*^ represents each reaction pathway contributing to the chelation process. The *K*_*i*_^*II*^ values were estimated following eqs 13 and 14:
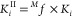
13where
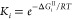
14Here, Δ*G*_*i*_^II^ represents the Gibbs free energy of the reaction, and *R* and *T* are the gas constant and temperature, respectively.

### Antioxidant Activity Type III

The studies in this section were conducted using the same level of theory as presented in AOX-I, with the inclusion of Grimme’s dispersion correction.^76^ This correction is recommended, particularly for Minnesota functionals, as they may not accurately recover the correct asymptotic behavior of London dispersion in the long intermolecular distance regime, such as for large molecules and liquids.^77^

## Data Availability

The data underlying this study are available in the published article and its Supporting Information. The preprint version of the manuscript can be accessed on the *ChemRxiv* server.^78^

## Supporting Information Available

Details on computational methods used to assess dissociation constants and perform thermochemical and kinetic computations; Results of acid–base equilibria investigations encompassing p*K*_a_ values and molar fractions, as well as appendix information regarding relative reactivity compared to other antioxidants; Figures of the transition states related to type III antioxidant activity; Cartesian coordinates, electronic, and thermochemical data of all the studied systems (PDF)
