# Supplementary material for: Unveiling the Antioxidative Potential of Galangin: Complete and Detailed Mechanistic Insights through Density Functional Theory Studies
Source: J Org Chem. 2024 Jun 11;89(12):8676–90. doi: 10.1021/acs.joc.4c00611 (PMC11197094; doi:10.1021/acs.joc.4c00611)
Supplement: Supplementary file 1 — jo4c00611_si_001.pdf [file jo4c00611_si_001.pdf]

Supporting Information

Unveiling the Antioxidative Potential of Galangin: Complete and Detailed Mechanistic Insights Through Density Functional Theory Studies

Maciej Spiegel

maciej.spiegel@umw.edu.pl

Department of Organic Chemistry and Pharmaceutical Technology,
Faculty of Pharmacy, Wroclaw Medical University, Borowska 211A, 50-556 Wroclaw, Poland

---

COMPUTATIONAL METHODS **S2**

DISSOCIATION CONSTANTS S2

THERMOCHEMISTRY S2

KINETICS S2

ACID–BASE EQUILIBRIA **S4**

RELATIVE REACTIVITY **S5**

REFERENCES **S6**

FIGURES **S9**

CARTESIAN COORDINATES & ENERGIES **S12**

Computational Methods

Dissociation Constants

To determine the acid constants for the substances under investigation, a parameter-fitted approach outlined by Galano et al.[1] was employed. This method involves calculating pK_a values using a linear fitting expression (**Equation S1**):

$$pK_a = m\Delta G_{BA} + C_0 \qquad \text{(S1)}$$

Here, ΔG_{BA} represents the Gibbs free energy difference between the conjugated base and the corresponding acid. The parameters m and C_0 are variable and contingent upon the specific substituents and the computational level employed. Only acid-base species with molar fractions (${}^{M}f$) exceeding 0.1% were considered for inclusion in the study. Although the original methodology was developed on Gaussian in version 09 for which default integration grid was “Fine”, while for the used here Gaussian in version 16 it is “UltraFine”.

Thermochemistry

The assessment of the thermodynamic feasibility of various processes involved analysing the Gibbs free energies of reaction (**Table S1**). The energies of solvated electron and proton were obtained from the study by Marković et al.[2]

Relative energies, incorporating thermodynamic corrections at 298.15 K, were calculated with respect to the sum of the isolated reactants, all referenced to the 1 M standard state. Additionally, solvent cage effects, accounting for entropy loss due to liquid-phase effects, were taken into consideration. This correction, following the approach proposed by Okuno[3], integrated the free volume theory by Benson[4], both to reduce over-penalizing entropy losses in solution. For a bimolecular reaction leading to a single product, the application of this correction at 298 K resulted in a reduction of 2.55 kcal mol^{-1} in the Gibbs free energy in solution compared to the same reaction in the gas phase. Neglecting both the standard state and solvent effects simultaneously in the calculation of reaction barriers would result in a substantial underestimation of rate constants, approximately by a factor of 1800, for bimolecular reactions at room temperature.[5] This highlights the significant influence of solvent effects on both the thermodynamics and kinetics of reactions in the solution phase.

	H_3Glg^{PET}	H_3Glg	H_2Glg^-	$HGlg^{2-}$
***f*-HAT**				
C_3	-0.8	-3.5	-7.1	
C_5	12.0	6.2	4.2	-4.9
C_7	8.6	7.2		
**RAF**				
C_2	-2.2	-3.5	-4.9	-18.2
C_3	5.2	4.8	0.5	3.3
C_4	41.7	39.3	13.6	0.8
C_{4a}	30.1	29.6	23.1	22.9
C_5	15.1	13.3	13.7	11.8
C_6	16.2	16.0	11.5	11.4
C_7	17.7	17.5	22.1	14.8
C_8	16.3	14.8	11.4	10.0
C_{8a}	20.3	19.4	17.8	15.8
$C_{1'}$	22.9	21.0	20.4	19.2
$C_{2'}$	14.0	13.0	12.0	6.3
$C_{3'}$	18.4	16.6	16.7	15.4
$C_{4'}$	11.6	11.6	10.2	4.7
$C_{5'}$	18.3	16.7	16.4	15.8
$C_{6'}$	14.5	12.3	12.0	6.9
**SET**		33.8	18.8	-1.2

Table S1. Gibbs free energies of reaction (ΔG, in kcal mol^{-1}, at 298.15 K) for the modelled pathways.

Kinetics

Kinetic data were obtained using the QM-ORSA protocol, a validated method designed for calculating rate constants in solution, demonstrating uncertainties comparable to experimental measurements.[5–7] Detailed information on the computational procedures can be found in the respective references.

The conventional Transition State Theory (TST)[8–10] was employed for rate constant calculations, utilizing harmonic vibrational frequencies and non-symmetrical, unidimensional Eckart tunnelling corrections.[11] The TST rate constant (k_{TST}, **Equation S2**) is expressed as:

$$k_{TST} = \sigma\kappa(T)\frac{k_bT}{h}e^{-\left(\frac{\Delta G^{\neq}}{RT}\right)} \qquad \text{(S2)}$$

Here, $\Delta G^{\neq}$ represents the Gibbs activation energy, σ is the reaction path degeneracy (also known as reaction symmetry number)[12,13], $\kappa(T)$ is the tunneling correction[14], T is the temperature, and k_b, h and R are the Boltzmann, Planck, and ideal gas constants, respectively. σ signifies the number of equivalent reaction paths, and for the studied reactions, $\sigma = 1$ due to the absence of rotational symmetry in the transition state geometries.[8,10,15]

For Single Electron Transfer (SET) reactions, activation energies were determined using Marcus theory.[16] The formula for calculating $\Delta G^{\neq}$ is given by **Equation S3**:

$$\Delta G^{\neq} = \frac{\lambda}{4}\left(1 + \frac{\Delta G_{SET}}{\lambda}\right)^2 \qquad \text{(S3)}$$

Here, ΔG_{SET} represents the free energy of the reaction, and λ corresponds to the reorganization energy. λ values were determined as the sum of internal (λ_i, $\lambda_i \approx \Delta E_{SET} - \Delta G_{SET}$ where ΔE_{SET} is the non-adiabatic energy difference between reactants and vertical products), and solvent (λ_o) reorganization energies.[17] The λ_o values were obtained from the 2-sphere model of Marcus (**Equation S4**):

$$\lambda_o = \Delta q^2\left(\frac{1}{\varepsilon_\infty} - \frac{1}{\varepsilon_0}\right)\left(\frac{1}{2r_A} + \frac{1}{2r_D} - \frac{1}{r_{AD}}\right) \qquad \text{(S4)}$$

where Δq is the amount of charge transferred, r_A, r_D, and r_{AD} are the effective radii of the acceptor (A), donor (D) and reactant complex (AD), and ε_∞ and ε_0 are the static and optical dielectric constants of the solvent, respectively. For the studied here reactions in which an electron complex is not formed, the r_{AD} value is approximated as the sum of the reactant radii.

Because some rate constants tend to approach the diffusion limit, a correction using the Collins–Kimball theory[18] was applied to yield realistic data. The apparent rate constant (k_{app}) in the solvents at 298.15K is given by **Equation S5**:

$$k_{app} = \frac{k_D k_{TST}}{k_D + k_{TST}} \qquad \text{(S5)}$$

Here k_D represent the steady-state Smoluchowski rate constant for an irreversible bimolecular diffusion-controlled reaction (**Equation S6**)[19]:

$$k_D = 4\pi R_{AB} D_{AB} N_A \qquad \text{(S6)}$$

where R_{AB} is the reaction distance, D_{AB} is the mutual diffusion coefficient of the reactants A and B computed as a sum of D_A and D_B, estimated using the Stokes-Einstein formulation (**Equation S7**):

$$D = \frac{k_BT}{6\pi\eta a} \qquad \text{(S7)}$$

with η representing the viscosity of the solvent ($\eta_{pentyl\ ethanoate}$ = 8.62 × 10^{-4} Pa s; η_{H_2O} = 8.91 × 10^{-4} Pa s) and a is the radius of the solute.

The total rate coefficients for the reactions (k_{total}) were determined by summing the contributions from each reaction path (i) as given by **Equation S8**:

$$k_{total} = \sum_i^n k_i \qquad \text{(S8)}$$

The overall rate coefficients ($k_{overall}$) were calculated by considering the molar fractions (${}^{M}f$) of the acid–base species involved in each chemical route at the pH of interest (**Equation S9**):

$$k_{overall} = {}^{M}f k_{total} + {}^{M}f^{-} k_{total}^{-} + {}^{M}f^{2-} k_{total}^{2-} \qquad \text{(S9)}$$

The molar fractions are computed from the pK_a values of the reactants following the **Equations S10-S12**:

$${}^{m}f^{2-} = \frac{1}{1+\beta_1[H^+]+\beta_2[H^+]^2} \qquad \text{(S10)}$$

$${}^{m}f^{-} = \beta_1[H^+]({}^{m}f^{2-}) \qquad \text{(S11)}$$

$${}^{m}f = \beta_2[H^+]^2({}^{m}f^{2-}) \qquad \text{(S12)}$$

where $\beta_1 = 10^{\mathrm{p}K_{a2}}$ and $\beta_2 = 10^{\mathrm{p}K_{a2}+\mathrm{p}K_{a1}}$.

The percent contributions of each reaction mechanism (Γ) are then estimated using the **Equation S13**:

$$\Gamma_i = 100 \times \frac{k_i}{k_{total}} \qquad \text{(S13)}$$

This approach provides a quantitative breakdown of the contribution of each mechanism to the total reaction rate, facilitating a more detailed analysis of the reaction network.

Acid–Base Equilibria

In aqueous solutions, the equilibrium between neutral and charged species in molecules with acid–base characteristics, governed by the pK_a-pH relationship, plays a crucial role. This equilibrium significantly influences the antioxidant activity of the substances.[20]

Currently, a complete set of experimental pK_a values for **Glg** is not available. In a prior study, Jovanovic et al.[21] determined spectrophotometrically pK_{a1} and pK_{a2} to be 6.8 and 9.4, respectively. Due to the absence of comprehensive experimental data, reliance is placed on the theoretical estimates presented in this paper. The proposed methodology[1], however, has been previously validated as reliable for polyphenolic compounds, producing results closely aligned with experimentally measured values. Further validation is provided here — specifically, the given procedure yields the first dissociation constant corresponding to the deprotonation of the phenolic OH located at C_7 (pK_{a1}=7.48), followed by C_3 (pK_{a2}=9.34), and finally the C_5 group (pK_{a3}=12.07). While the first estimated value is 10% higher than reported in the experimental study, the second one is nearly identical.

The hydroxyl group at C_7 exhibits the highest propensity for deprotonation, consistent with previous observations.[22–25] Additionally, the intramolecular hydrogen bonding with the carbonyl residue at C_4, involving the acidic hydrogens of C_3 and C_5 groups, stabilizes the system *via* electron density delocalization, reducing the tendency for dissociation of the corresponding hydroxyl groups[26]. Therefore, the proposed deprotonation pathway is deemed reasonable.

As indicated by the graph depicting molar fraction as a function of pH (**Figure S1**), the species with the largest population at pH=7.4 comprises the neutral form (${}^{M}f = 54.37\%$) and the product of the first deprotonation (${}^{M}f^{-} = 45.11\%$). Although the dianion is predicted to exist only to a minor extent under the same conditions, its population is not negligible $({}^{M}f^{2-} = 0.51\%)$ and should be duly considered.

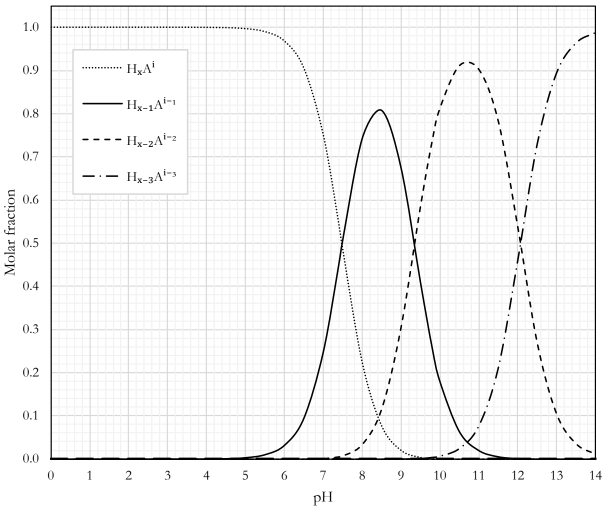

Figure S1. Molar fractions of galangin species plotted as a function of pH.

It's noteworthy that the original paper by Galano et al.[1] utilized Gaussian09, where the default integration grid is "Fine.". In our study, we employed the newer iteration of the software, Gaussian16, where the default integration grid is now "UltraFine." Although such version was also used by the protocol's authors in other papers[27 28], to further ensure the reliability of the methodology, the energies were recomputed with the "Fine" grid imposed.

The resulting pK_a values were only slightly altered, measuring 7.51, 9.38, and 12.11. Consequently, the molar fractions shifted from 54.37%, 45.11%, and 0.51% to 56.16%, 43.39%, and 0.46%. This indicates a minimal impact of the integration grid change, allowing us to confidently rely on the original results.

Relative Reactivity

The ionization potential (IP) and bond dissociation energy (BDE) were systematically computed using the ΔSCF framework to construct the electron and hydrogen-donating ability map for antioxidants, known as eH-DAMA.[29] eH-DAMA visually represents the likelihood of molecules as hydrogen and electron donors, reflecting their capabilities in hydrogen atom transfer and electron transfer mechanisms. The most effective radical scavengers are anticipated to be located in the bottom-left quarter. This approach provides a comprehensive exploration of the molecule's reactivity for comparison purposes.

Figure S2 illustrates two eH-DAMA maps, one for a nonpolar environment (upper, for pentyl ethanoate) and the other for a polar environment (lower, for water). All undissociated hydroxyl groups within the molecule, potentially acting as hydrogen donors ($H^{\bullet}$), were considered when plotting the maps. These maps include the dominant acid–base species of **Glg**, reference substances (α-tocopherol, Trolox, ascorbic acid), various flavonoids (isorhamnetin, scutellarein, apigenin, pinocembrin)[22–25] marked for comparative purposes, and the $H_2O_2/O_2^{\bullet-}$ pair representing the potential oxidant target,

The lowest BDE and IP values for the galangin species were estimated as (91.9 kcal mol^{-1}, 5.9 eV) in pentyl ethanoate (**H_3Glg^{PET}**), and (91.3 kcal mol^{-1}, 5.0 eV), (88.0 kcal mol^{-1}, 4.4 eV), (90.6 kcal mol^{-1}, 3.5 eV) for the neutral (**H_3Glg**), monoanionic (**H_2Glg^-**), and dianionic (**$HGlg^{2-}$**) species, respectively. Unfortunately, comparative data in the literature are sparse. In our previous paper[26] employing the B3LYP/6-31+G(d,p)/PCM level of theory, **H_3Glg** was associated with a slightly different BDE value of 86.9 kcal mol^{-1}. However, the IP outputs remains coherent. Other study, by Lewandowski et al.[30] , reported intrinsic reactivity indices of 78.7 kcal mol^{-1} and 4.83 eV, obtained through computations under the B3LYP/6-311++G(d,p)/PCM regime. The observed inconsistency is not surprising, given B3LYP's known limitations in such studies, in contrast to the level of theory chosen here[31,32]. Furthermore, determining IP is highly reliant on the functional and basis set, emphasizing the need for methodological consistency for accurate and meaningful comparisons in reactivity studies.[32] Very recently, a benchmark

on these two reactivity indices, with reference energy values computed at 'golden standard' DLPNO-CCSD(T)/CBS[DZ-TZ,def2]/CPCM, was conducted for a set of polyphenolic compounds, including quercetin, which, like galangin, belongs to the group of flavonols.[33] The authors reported that the mean absolute deviation of BDE and IP energies for the level of theory used in this paper is the closest to the reference. This further confirms that M052X/6-311+G(d,p) chosen is trustworthy, furthermore validating the output presented in this work.

In a nonpolar medium, **H_3Glg^{PET}** shows promise for deactivating free radicals through electron transfer compared to pinocembrin, apigenin, and isorhamnetin. However, based on BDE, all other reductants, except pinocembrin, are projected to undergo hydrogen atom transfer more readily. Notably, the BDE value is on the margin of the box drawn by the H_2O_2/•OOH pair, suggesting that the hydrogen atom transfer process might not be particularly effective

Transitioning to a polar environment, the substance's activity improves significantly, observed in a simultaneous decrease in BDE and IP values. In this environment, any **Glg** species demonstrates the capability to reduce the reference oxidant. Also, consecutive deprotonations significantly lower the IP value. Interestingly, the pattern is similar to that of pinocembrin, but the additional hydroxyl group at C_3 and a double bond between C_2 and C_3 influence the reactivity indices, causing a notable shift towards the zone corresponding to more active antioxidants. While neutral and monoanionic species may not be more effective antiradical agents than Trolox anion and ascorbate, the dianionic form is likely to exhibit an outstanding propensity for electron transfer, beating any other tested flavonoid insofar.

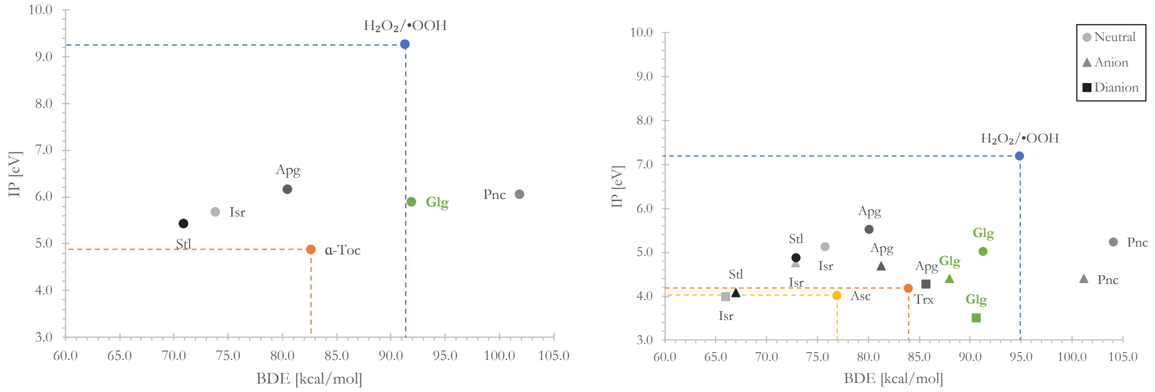

Figure S2. Electron and Hydrogen Donating Ability Maps for Antioxidants in pentyl ethanoate (left) and water (right).

References

(1) Galano, A.; Pérez-González, A.; Castañeda-Arriaga, R.; Muñoz-Rugeles, L.; Mendoza-Sarmiento, G.; Romero-Silva, A.; Ibarra-Escutia, A.; Rebollar-Zepeda, A. M.; León-Carmona, J. R.; Hernández-Olivares, M. A.; Alvarez-Idaboy, J. R. Empirically Fitted Parameters for Calculating p *K* a Values with Small Deviations from Experiments Using a Simple Computational Strategy. *J. Chem. Inf. Model.* **2016**, *56* (9), 1714–1724. https://doi.org/10.1021/acs.jcim.6b00310.

(2) Marković, Z.; Tošović, J.; Milenković, D.; Marković, S. Revisiting the Solvation Enthalpies and Free Energies of the Proton and Electron in Various Solvents. *Computational and Theoretical Chemistry* **2016**, *1077*, 11–17. https://doi.org/10.1016/j.comptc.2015.09.007.

(3) Okuno, Y. Theoretical Investigation of the Mechanism of the Baeyer-Villiger Reaction in Nonpolar Solvents. *Chemistry A European J* **1997**, *3* (2), 212–218. https://doi.org/10.1002/chem.19970030208.

(4) Benson, S. W. *The Foundations of Chemical Kinetics*; Advanced Chemistry Series; McGraw-Hill, 1960.

(5) Galano, A.; Alvarez-Idaboy, J. R. A Computational Methodology for Accurate Predictions of Rate Constants in Solution: Application to the Assessment of Primary Antioxidant Activity. *J Comput Chem* **2013**, *34* (28), 2430–2445. https://doi.org/10.1002/jcc.23409.

(6) Galano, A.; Mazzone, G.; Alvarez-Diduk, R.; Marino, T.; Alvarez-Idaboy, J. R.; Russo, N. Food Antioxidants: Chemical Insights at the Molecular Level. *Annu. Rev. Food Sci. Technol.* **2016**, *7* (1), 335–352. https://doi.org/10.1146/annurev-food-041715-033206.
(7) Galano, A.; Alvarez-Idaboy, J. R. Computational Strategies for Predicting Free Radical Scavengers' Protection against Oxidative Stress: Where Are We and What Might Follow? *Int J of Quantum Chemistry* **2019**, *119* (2), e25665. https://doi.org/10.1002/qua.25665.
(8) Evans, M. G.; Polanyi, M. Some Applications of the Transition State Method to the Calculation of Reaction Velocities, Especially in Solution. *Trans. Faraday Soc.* **1935**, *31*, 875. https://doi.org/10.1039/tf9353100875.
(9) Truhlar, D. G.; Garrett, B. C.; Klippenstein, S. J. Current Status of Transition-State Theory. *J. Phys. Chem.* **1996**, *100* (31), 12771–12800. https://doi.org/10.1021/jp953748q.
(10) Eyring, H. The Activated Complex in Chemical Reactions. *The Journal of Chemical Physics* **1935**, *3* (2), 107–115. https://doi.org/10.1063/1.1749604.
(11) Eckart, C. The Penetration of a Potential Barrier by Electrons. *Phys. Rev.* **1930**, *35* (11), 1303–1309. https://doi.org/10.1103/PhysRev.35.1303.
(12) Pollak, E.; Pechukas, P. Symmetry Numbers, Not Statistical Factors, Should Be Used in Absolute Rate Theory and in Broensted Relations. *J. Am. Chem. Soc.* **1978**, *100* (10), 2984–2991. https://doi.org/10.1021/ja00478a009.
(13) Laidler, K. J. *Chemical Kinetics*, 3. ed., [Nachdr.].; HarperCollins New York, N.Y.: New York, N.Y., 1998.
(14) Kuppermann, A.; Truhlar, D. G. Exact Tunneling Calculations. *J. Am. Chem. Soc.* **1971**, *93* (8), 1840–1851. https://doi.org/10.1021/ja00737a002.
(15) Truhlar, D. G.; Hase, W. L.; Hynes, J. T. Current Status of Transition-State Theory. *J. Phys. Chem.* **1983**, *87* (15), 2664–2682. https://doi.org/10.1021/j100238a003.
(16) Marcus, R. A. Electron Transfer Reactions in Chemistry. Theory and Experiment. *Rev. Mod. Phys.* **1993**, *65* (3), 599–610. https://doi.org/10.1103/RevModPhys.65.599.
(17) Marcus, R. A.; Sutin, N. Electron Transfers in Chemistry and Biology. *Biochimica et Biophysica Acta (BBA) - Reviews on Bioenergetics* **1985**, *811* (3), 265–322. https://doi.org/10.1016/0304-4173(85)90014-X.
(18) Collins, F. C.; Kimball, G. E. Diffusion-Controlled Reaction Rates. *Journal of Colloid Science* **1949**, *4* (4), 425–437. https://doi.org/10.1016/0095-8522(49)90023-9.
(19) Smoluchowski, M. V. Versuch Einer Mathematischen Theorie Der Koagulationskinetik Kolloider Lösungen. *Zeitschrift für Physikalische Chemie* **1918**, *92U* (1), 129–168. https://doi.org/10.1515/zpch-1918-9209.
(20) Spiegel, M.; Cel, K.; Sroka, Z. The Mechanistic Insights into the Role of pH and Solvent on Antiradical and Prooxidant Properties of Polyphenols — Nine Compounds Case Study. *Food Chemistry* **2023**, *407*, 134677. https://doi.org/10.1016/j.foodchem.2022.134677.
(21) Jovanovic, S. V.; Steenken, S.; Tosic, M.; Marjanovic, B.; Simic, M. G. Flavonoids as Antioxidants. *J. Am. Chem. Soc.* **1994**, *116* (11), 4846–4851. https://doi.org/10.1021/ja00090a032.
(22) Spiegel, M. Theoretical Insights into the Oxidative Stress-Relieving Properties of Pinocembrin—An Isolated Flavonoid from Honey and Propolis. *J. Phys. Chem. B* **2023**, *127* (41), 8769–8779. https://doi.org/10.1021/acs.jpcb.3c03545.
(23) Spiegel, M.; Marino, T.; Prejanò, M.; Russo, N. On the Scavenging Ability of Scutellarein against the OOH Radical in Water and Lipid-like Environments: A Theoretical Study. *Antioxidants* **2022**, *11* (2), 224. https://doi.org/10.3390/antiox11020224.
(24) Spiegel, M.; Ciardullo, G.; Marino, T.; Russo, N. Computational Investigation on the Antioxidant Activities and on the Mpro SARS-CoV-2 Non-Covalent Inhibition of Isorhamnetin. *Front. Chem.* **2023**, *11*, 1122880. https://doi.org/10.3389/fchem.2023.1122880.
(25) Spiegel, M.; Sroka, Z. Quantum-Mechanical Characteristics of Apigenin: Antiradical, Metal Chelation and Inhibitory Properties in Physiologically Relevant Media. *Fitoterapia* **2023**, *164* (October 2022), 105352. https://doi.org/10.1016/j.fitote.2022.105352.
(26) Spiegel, M.; Andruniów, T.; Sroka, Z. Flavones' and Flavonols' Antiradical Structure–Activity Relationship—A Quantum Chemical Study. *Antioxidants* **2020**, *9* (6), 461. https://doi.org/10.3390/antiox9060461.
(27) Pérez-González, A.; Castañeda-Arriaga, R.; Guzmán-López, E. G.; Hernández-Ayala, L. F.; Galano, A. Chalcone Derivatives with a High Potential as Multifunctional Antioxidant Neuroprotectors. *ACS Omega* **2022**, *7* (43), 38254–38268. https://doi.org/10.1021/acsomega.2c05518.

(28) Hernández-Ayala, L. F.; Guzmán-López, E. G.; Galano, A. Quinoline Derivatives: Promising Antioxidants with Neuroprotective Potential. *Antioxidants* **2023**, *12* (10), 1853. https://doi.org/10.3390/antiox12101853.

(29) Guzman-Lopez, E.; Reina, M.; Perez-Gonzalez, A.; Francisco-Marquez, M.; Hernandez-Ayala, L.; Castañeda-Arriaga, R.; Galano, A. CADMA-Chem: A Computational Protocol Based on Chemical Properties Aimed to Design Multifunctional Antioxidants. *IJMS* **2022**, *23* (21), 13246. https://doi.org/10.3390/ijms232113246.

(30) Lewandowski, W.; Lewandowska, H.; Golonko, A.; Świderski, G.; Świsłocka, R.; Kalinowska, M. Correlations between Molecular Structure and Biological Activity in "Logical Series" of Dietary Chromone Derivatives. *PLoS ONE* **2020**, *15* (8), e0229477. https://doi.org/10.1371/journal.pone.0229477.

(31) De Souza, G. L. C.; Peterson, K. A. Benchmarking Antioxidant-Related Properties for Gallic Acid through the Use of DFT, MP2, CCSD, and CCSD(T) Approaches. *J. Phys. Chem. A* **2021**, *125* (1), 198–208. https://doi.org/10.1021/acs.jpca.0c09116.

(32) Spiegel, M.; Gamian, A.; Sroka, Z. A Statistically Supported Antioxidant Activity DFT Benchmark—The Effects of Hartree–Fock Exchange and Basis Set Selection on Accuracy and Resources Uptake. *Molecules* **2021**, *26* (16), 5058. https://doi.org/10.3390/molecules26165058.

(33) Mendes, R. A.; Da Mata, V. A. S.; Brown, A.; De Souza, G. L. C. A Density Functional Theory Benchmark on Antioxidant-Related Properties of Polyphenols. *Phys. Chem. Chem. Phys.* **2024**, 10.1039.D3CP04412B. https://doi.org/10.1039/D3CP04412B.

Figures

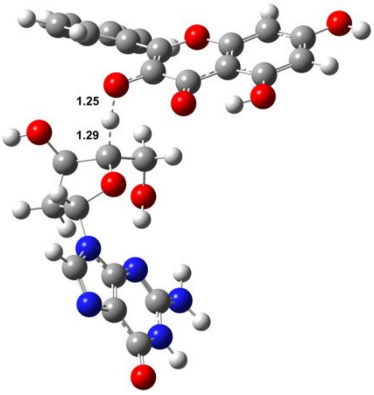

2dG (C_3)

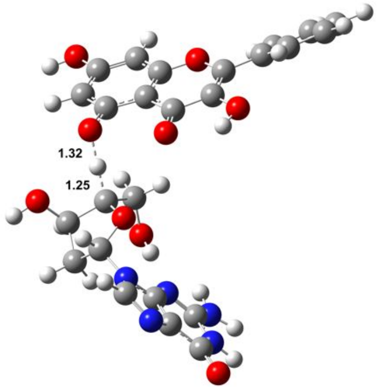

2dG (C_5)

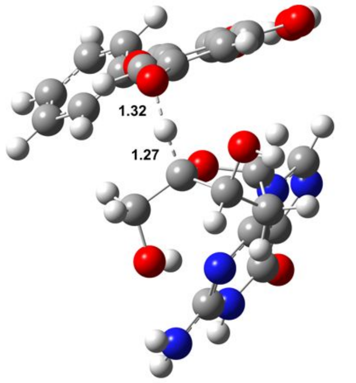

2dG (C_7)

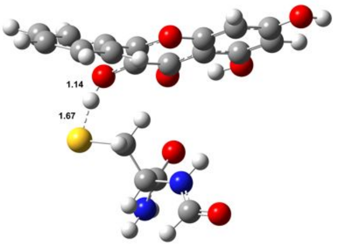

NF-Cys (C_3)

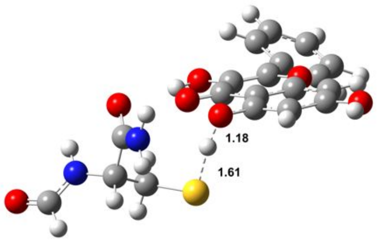

NF-Cys (C_5)

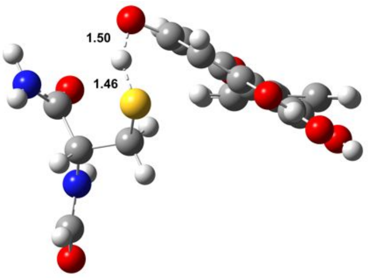

NF-Cys (C_7)

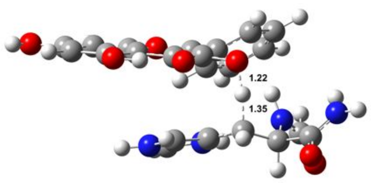

NF-His (C_3)

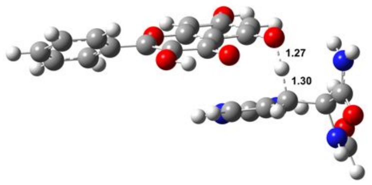

NF-His (C_5)

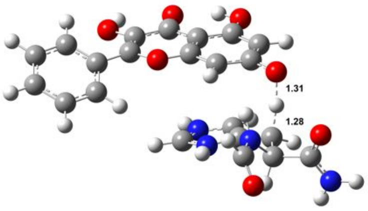

NF-His (C_7)

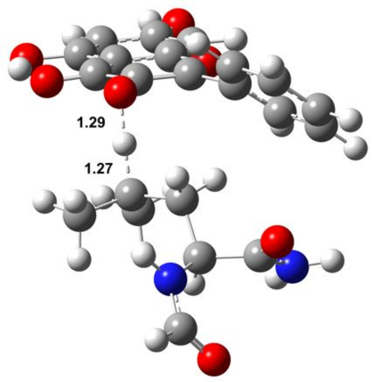

NF-Leu (C_3)

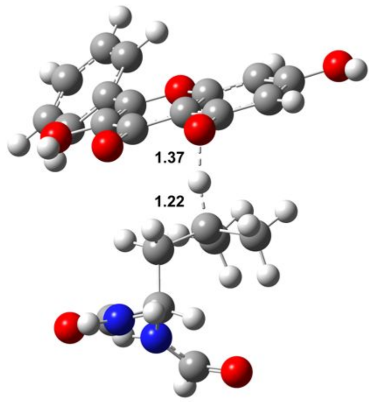

NF-Leu (C_5)

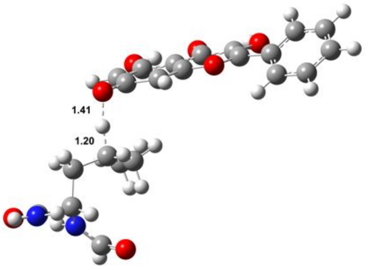

NF-Leu (C_7)

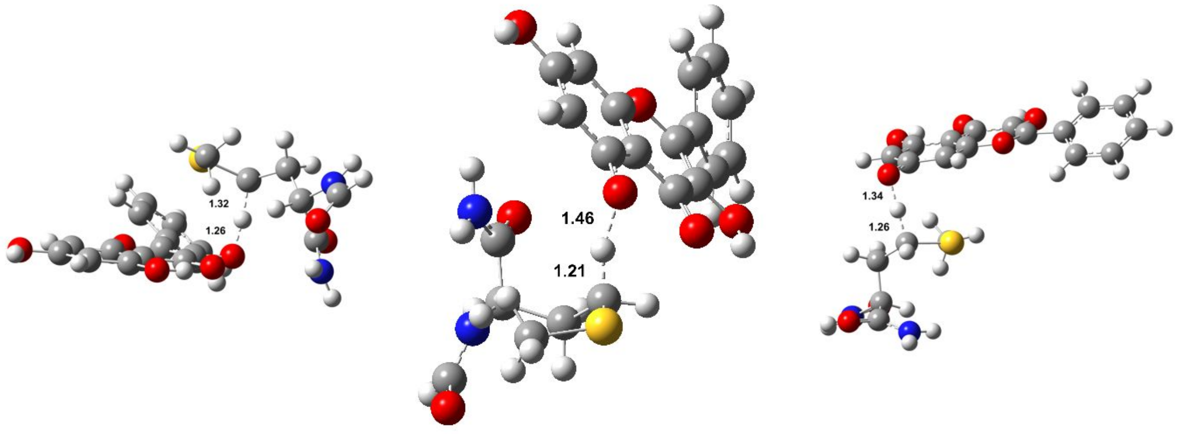

NF-Met (C_3) NF-Met (C_5) NF-Met (C_7)

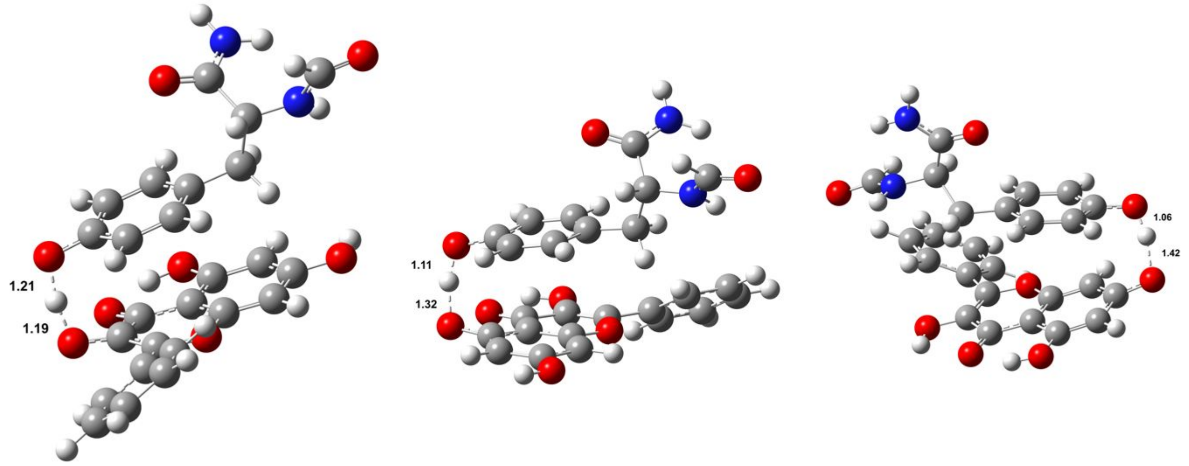

NF-Tyr (C_3) NF-Tyr (C_5) NF-Tyr (C_7)

Figure S3. Optimized geometries of transition states for repair mechanisms by **H_3Glg**, with distances reported in angstroms

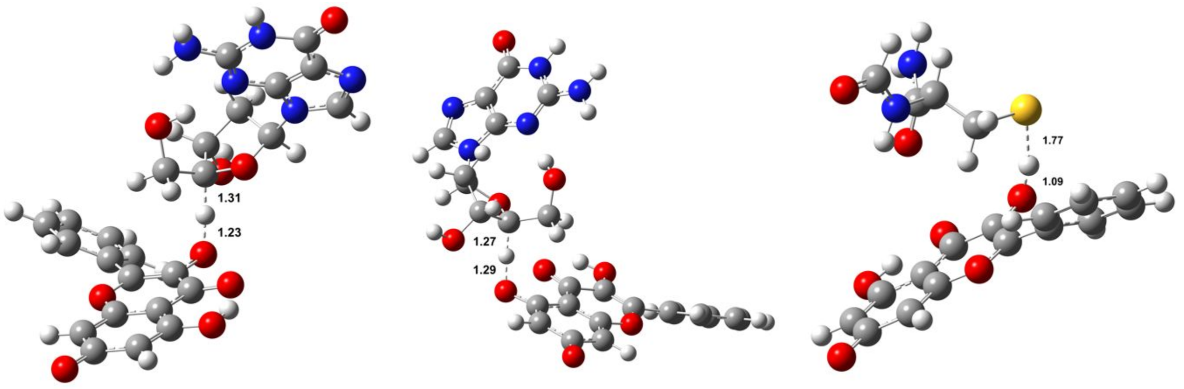

2dG (C_3) 2dG (C_5) NF-Cys (C_3)

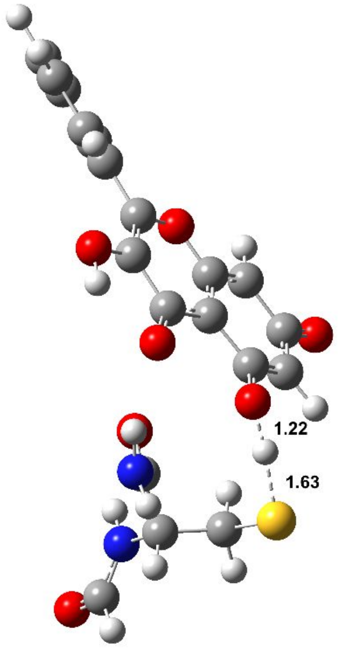

NF-Cys (C_5)

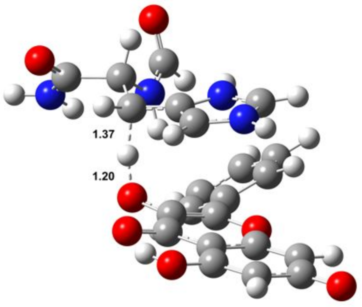

NF-His (C_3)

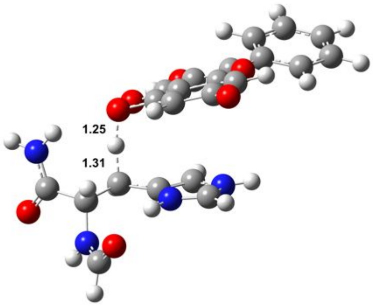

NF-His (C_5)

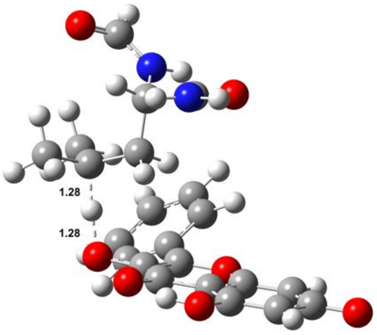

NF-Leu (C_3)

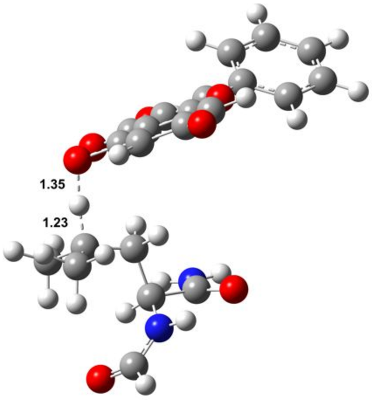

NF-Leu (C_5)

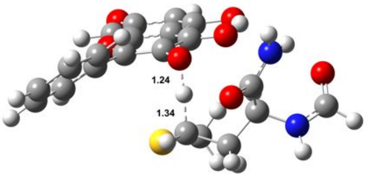

NF-Met (C_3)

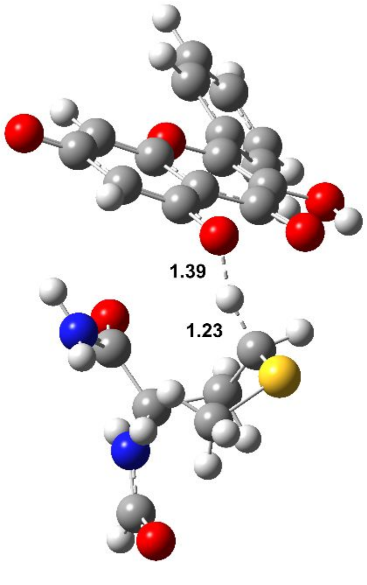

NF-Met (C_5)

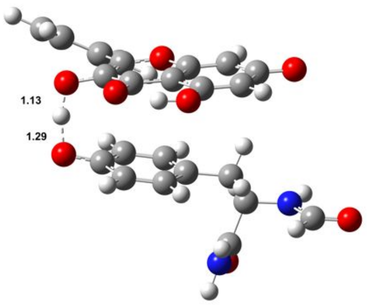

NF-Tyr (C_3)

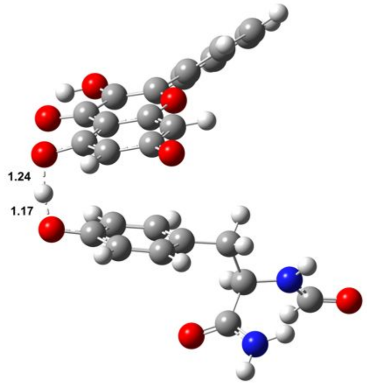

NF-Tyr (C_5)

Figure S4. Optimized geometries of transition states for repair mechanisms by $\mathbf{H_2Glg^-}$, with distances

reported in angstroms

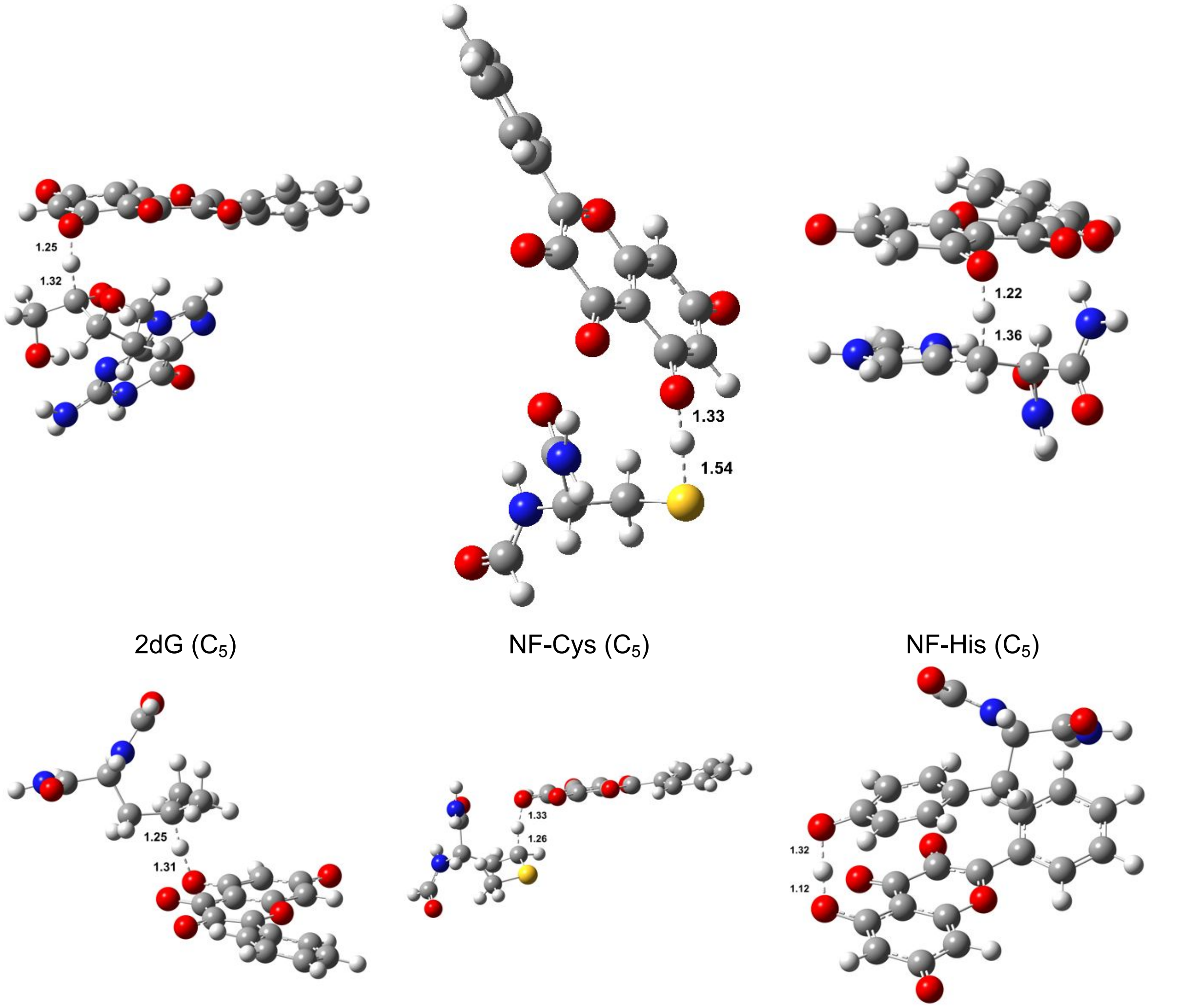

2dG (C_5) NF-Cys (C_5) NF-His (C_5)

NF-Leu (C_5) NF-Met (C_5) NF-Tyr (C_5)

Figure S5. Optimized geometries of transition states for repair mechanisms by **HGlg^{2-}**, with distances reported in angstroms

Cartesian coordinates & energies

==> ./AOX-III/water <==

O	-0.214984	-0.882345	0.078474
O	-1.377530	2.536020	-0.180165
O	3.717630	1.846734	-0.106688
O	1.275281	2.870147	-0.182346
O	4.008800	-2.882269	0.199249
C	1.704527	0.542636	-0.018467
C	1.132629	-0.732770	0.061681
C	-1.055395	0.187883	0.013092
C	0.856470	1.695258	-0.092133
C	-0.567812	1.447654	-0.074138
C	-2.468374	-0.207550	0.014956
C	3.117537	0.639254	-0.028370
C	1.892034	-1.886906	0.133633
C	3.272080	-1.745567	0.124060
C	3.895628	-0.495470	0.042202
C	-2.838958	-1.414217	-0.586952
C	-3.440704	0.591243	0.622212
C	-4.169693	-1.806172	-0.594295
C	-4.769091	0.187179	0.617418
C	-5.137222	-1.006969	0.006867
H	1.425029	-2.858181	0.197310
H	4.974452	-0.416877	0.033528
H	-2.088293	-2.034610	-1.055791
H	-3.158408	1.510925	1.111740
H	-4.451157	-2.735419	-1.070250
H	-5.516115	0.804706	1.096825
H	-6.173638	-1.315813	0.002474
H	-0.792836	3.305492	-0.270850
H	3.015857	2.529887	-0.156877

H	4.949597	-2.669329	0.193101

SCF Energy: -953.942936373
Sum of electronic and zero-point Energies= -953.720358
Sum of electronic and thermal Energies= -953.704563
Sum of electronic and thermal Enthalpies= -953.703618
Sum of electronic and thermal Free Energies= -953.763711

==> ./AOX-III/water/His/C5 <==

O	-2.881012	0.839503	0.503168
O	-2.869479	-2.717421	-0.107756
O	1.513618	-0.590368	1.777113
O	-0.400585	-2.348344	0.703860
O	0.270309	3.979976	1.885558
C	-0.705860	0.001791	1.091169
C	-1.615246	1.049066	0.934359
C	-3.322772	-0.404931	0.174095
C	-1.111837	-1.333433	0.707715
C	-2.489774	-1.467061	0.261729
C	-4.713310	-0.406357	-0.293256
C	0.603928	0.338363	1.570734
C	-1.296634	2.378398	1.200406
C	-0.017772	2.677060	1.642069
C	0.929277	1.671457	1.826079
C	-5.210412	0.717981	-0.960451
C	-5.550269	-1.502650	-0.067554
C	-6.523111	0.735817	-1.408803
C	-6.864827	-1.473226	-0.513297
C	-7.352826	-0.359019	-1.187305
H	-2.036702	3.152702	1.059726
H	1.925560	1.907501	2.176990
H	-4.567163	1.568627	-1.135341
H	-5.183066	-2.363719	0.469473
H	-6.898270	1.604642	-1.931874
H	-7.509709	-2.320984	-0.327370
H	-8.376549	-0.341838	-1.535405
H	-2.077697	-3.272959	-0.005108
H	2.102441	-0.818164	0.674568
O	4.872286	-3.232146	-0.281722
N	2.824261	1.658923	-0.948639
N	4.990399	-0.806648	-1.389915
N	0.874809	2.057667	-1.757328
N	4.060626	-2.450805	1.679060
C	2.670229	-0.810716	-0.493796
C	4.162256	-0.938910	-0.206841
C	2.173021	0.436361	-1.020966
C	4.410275	-2.319822	0.401645
C	0.925186	0.710683	-1.535066
C	2.029004	2.614695	-1.397914
H	2.218416	-1.679299	-0.968217
H	4.468005	-0.174040	0.506429
H	3.808592	1.785755	-0.693491
H	0.105962	0.052140	-1.760471
H	2.276223	3.658444	-1.477560
H	3.678837	-1.677091	2.201006
H	4.131753	-3.355394	2.119973
H	0.081898	2.569077	-2.124731
C	5.693715	0.291370	-1.668604
H	5.157089	-1.641624	-1.935088
O	5.617874	1.348608	-1.032479
H	6.353736	0.185444	-2.531621
H	1.172436	4.066858	2.217068

SCF Energy: -1596.11394406
Sum of electronic and zero-point Energies= -1595.709058
Sum of electronic and thermal Energies= -1595.680193
Sum of electronic and thermal Enthalpies= -1595.679249
Sum of electronic and thermal Free Energies= -1595.771678

==> ./AOX-III/water/His/C7 <==

O	-1.812495	0.242193	-0.760721
O	-4.895254	-1.160399	0.498422
O	-0.998189	-4.447962	-0.161956
O	-3.353943	-3.337084	0.321903
O	2.462213	-1.516378	-1.475299
C	-1.478943	-2.112262	-0.463761
C	-1.004365	-0.844332	-0.816034
C	-3.095580	0.155673	-0.324492
C	-2.842808	-2.253038	-0.027297
C	-3.626659	-1.042303	0.030236
C	-3.755112	1.463578	-0.260421
C	-0.575972	-3.208782	-0.497932
C	0.303398	-0.629197	-1.209037
C	1.185922	-1.719503	-1.198702
C	0.740472	-3.012676	-0.842424
C	-2.984619	2.598520	0.016308
C	-5.128505	1.595294	-0.483014
C	-3.587106	3.846045	0.083348
C	-5.720835	2.849370	-0.422626
C	-4.955340	3.974288	-0.135368
H	0.646703	0.360166	-1.475240
H	1.439263	-3.837042	-0.833740
H	-1.921650	2.500404	0.188491
H	-5.725224	0.728366	-0.721589
H	-2.988634	4.718427	0.306758
H	-6.781936	2.947026	-0.605879
H	-5.422304	4.948436	-0.085787
H	-5.026490	-2.098384	0.711993

H	-1.943964	-4.390748	0.088425
H	2.981745	-0.975475	-0.406712
O	5.734972	0.232185	-1.339634
N	1.298004	0.993034	1.352797
N	3.454477	1.602178	-0.628957
N	0.032666	-0.459483	2.295810
N	6.370633	0.031542	0.822816
C	3.284163	-0.427201	0.707967
C	4.124095	0.790409	0.366006
C	1.999903	-0.203949	1.334816
C	5.498395	0.331816	-0.138148
C	1.173987	-1.122272	1.943331
C	0.119194	0.816014	1.927089
H	3.837727	-1.228932	1.189357
H	4.272771	1.393899	1.264698
H	1.620702	1.871990	0.963838
H	1.328805	-2.165481	2.150382
H	-0.631535	1.570749	2.082462
H	6.131457	0.143212	1.795441
H	7.275260	-0.341084	0.577717
H	-0.772080	-0.868668	2.755875
C	3.923617	2.825464	-0.925422
H	2.854619	1.147895	-1.305333
O	4.829496	3.371591	-0.302587
H	3.416094	3.305446	-1.765982

SCF Energy: -1596.11482554
Sum of electronic and zero-point Energies= -1595.709842
Sum of electronic and thermal Energies= -1595.681067
Sum of electronic and thermal Enthalpies= -1595.680123
Sum of electronic and thermal Free Energies= -1595.772254

==> ./AOX-III/water/His/C3 <==

O	1.746315	1.528202	-0.349781
O	-0.657653	-0.786430	-1.675405
O	4.145241	-2.575743	-1.005489
O	1.598013	-2.327250	-1.615624
O	6.247648	1.245697	0.866861
C	2.879519	-0.556823	-0.722332
C	2.878845	0.767667	-0.270426
C	0.594611	1.028229	-0.834063
C	1.670436	-1.145023	-1.239153
C	0.492799	-0.274917	-1.271222
C	-0.501133	1.998431	-0.812510
C	4.085063	-1.289180	-0.605547
C	3.992781	1.381575	0.261878
C	5.159293	0.628357	0.346654
C	5.217303	-0.699919	-0.078929
C	-0.607217	2.887360	0.262903
C	-1.430990	2.051477	-1.856914
C	-1.653298	3.797105	0.306177
C	-2.465733	2.975623	-1.811163
C	-2.583377	3.841680	-0.728333
H	3.959734	2.407924	0.595050
H	6.135316	-1.266176	0.003476
H	0.115258	2.849270	1.066780
H	-1.331330	1.386494	-2.701766
H	-1.741913	4.472432	1.145823
H	-3.177764	3.021061	-2.623521
H	-3.396375	4.554060	-0.692879
H	-1.185447	-1.338002	-0.719111
H	3.255667	-2.828048	-1.335098
O	-3.878381	-3.556749	-0.079204
N	-0.831184	0.007501	2.043941
N	-3.271064	0.001396	0.278463
N	1.140214	-0.689526	2.520517
N	-4.604437	-1.809681	-1.315183
C	-1.623665	-1.796911	0.468207
C	-3.084921	-1.407898	0.564500
C	-0.677479	-1.201195	1.378254
C	-3.904342	-2.349020	-0.322677
C	0.591950	-1.630464	1.691681
C	0.272638	0.296565	2.715862
H	-1.459445	-2.861736	0.341549
H	-3.442163	-1.593552	1.581115
H	-1.643854	0.611889	2.004900
H	1.114543	-2.523148	1.398340
H	0.433637	1.169325	3.323540
H	-4.597719	-0.818868	-1.492624
H	-5.156268	-2.412388	-1.906806
H	2.070363	-0.720364	2.919944
C	-4.274355	0.719655	0.814984
H	-2.670316	0.452484	-0.401226
O	-5.102791	0.262281	1.595494
H	-4.283569	1.764443	0.492267
H	7.007448	0.650778	0.864263

SCF Energy: -1596.12828001
Sum of electronic and zero-point Energies= -1595.723316
Sum of electronic and thermal Energies= -1595.694700
Sum of electronic and thermal Enthalpies= -1595.693756
Sum of electronic and thermal Free Energies= -1595.784720

==> ./AOX-III/water/Cys/C5 <==

O	3.145594	0.787057	-0.005661
O	1.549293	-2.402394	-0.519200
O	-1.470070	1.857440	-0.615485
O	-0.511050	-0.779411	-0.720383

O	1.748930	5.222935	0.209378
C	0.804651	1.184005	-0.318148
C	2.098811	1.630589	-0.076904
C	2.989812	-0.555431	-0.157797
C	0.575785	-0.232726	-0.502002
C	1.759505	-1.072419	-0.394893
C	4.246888	-1.292990	-0.002511
C	-0.223734	2.179854	-0.375455
C	2.418659	2.981646	0.100378
C	1.404045	3.925622	0.036747
C	0.088506	3.532755	-0.182916
C	5.236161	-0.781917	0.844401
C	4.476639	-2.484280	-0.696691
C	6.432749	-1.464760	1.004733
C	5.680456	-3.156663	-0.535497
C	6.657541	-2.653050	0.316459
H	3.443459	3.272078	0.281346
H	-0.712111	4.259997	-0.214467
H	5.062256	0.139456	1.381842
H	3.728811	-2.872041	-1.371159
H	7.189689	-1.069337	1.667931
H	5.856041	-4.073317	-1.081493
H	7.592206	-3.182601	0.441294
H	-1.985446	1.356084	0.325252
S	-2.667308	0.889331	1.705092
N	-4.765325	1.324554	-1.125007
O	-3.642896	-0.497203	-1.858134
O	-6.512112	-3.651825	0.507623
N	-5.103042	-1.939912	0.025542
C	-4.592864	-0.614102	0.329792
C	-3.310567	-0.716654	1.160313
C	-4.295790	0.086621	-0.989914
C	-6.119372	-2.497952	0.687077
H	-5.354329	-0.072957	0.888077
H	-3.536890	-1.279428	2.067124
H	-2.542930	-1.251905	0.603727
H	-4.586569	-2.505259	-0.637083
H	-6.598181	-1.833616	1.413485
H	-4.528609	1.857742	-1.948407
H	-5.261122	1.773295	-0.371837
H	0.591673	-2.510376	-0.653114
H	0.968752	5.788734	0.154121

SCF Energy: -1768.78330009
Sum of electronic and zero-point Energies= -1768.444740
Sum of electronic and thermal Energies= -1768.417465
Sum of electronic and thermal Enthalpies= -1768.416520
Sum of electronic and thermal Free Energies= -1768.507730

==> ./AOX-III/water/Cys/C3 <==

O	-0.744795	-1.886784	0.680448
O	1.028573	-0.847591	-2.240267
O	-3.944495	0.483739	-2.003430
O	-1.473908	0.049764	-2.816869
O	-5.079925	-1.207672	2.277191
C	-2.286369	-0.684736	-0.725475
C	-1.991067	-1.354040	0.468177
C	0.239029	-1.760486	-0.212439
C	-1.276877	-0.516088	-1.734132
C	0.049865	-1.061043	-1.402481
C	1.504891	-2.329340	0.219356
C	-3.597206	-0.172713	-0.881793
C	-2.907764	-1.536265	1.476278
C	-4.187545	-1.017162	1.279557
C	-4.539931	-0.337222	0.115177
C	1.834836	-2.286247	1.581259
C	2.385224	-2.913931	-0.701190
C	3.047891	-2.797326	2.009004
C	3.585462	-3.443576	-0.256680
C	3.922784	-3.376137	1.091820
H	-2.647707	-2.063980	2.381492
H	-5.539083	0.055710	-0.015166
H	1.153428	-1.829734	2.285200
H	2.118186	-2.973835	-1.745165
H	3.313998	-2.745937	3.055390
H	4.259657	-3.908117	-0.962335
H	4.867176	-3.779215	1.431134
H	1.910044	-0.276779	-1.789933
H	-3.162808	0.506994	-2.595706
H	-5.933825	-0.822900	2.043778
S	3.008607	0.759015	-1.075079
N	1.222260	4.016426	-1.662015
O	-0.253813	2.379810	-1.157306
O	0.421533	4.150266	3.418540
N	0.761381	2.990288	1.498165
C	1.583862	2.681357	0.339167
C	1.962225	1.203941	0.336164
C	0.776173	3.012292	-0.911633
C	1.106339	3.882566	2.430475
H	2.477117	3.300210	0.390448
H	1.040531	0.612636	0.299889
H	2.476433	0.958880	1.264084
H	-0.119995	2.501835	1.601085
H	2.069157	4.369383	2.246228
H	0.716542	4.277906	-2.494980
H	2.081644	4.490385	-1.437361

SCF Energy: -1768.79702663
Sum of electronic and zero-point Energies= -1768.457269
Sum of electronic and thermal Energies= -1768.430487

Sum of electronic and thermal Enthalpies= -1768.429543
Sum of electronic and thermal Free Energies= -1768.519502

==> ./AOX-III/water/Cys/C7 <==

O	1.817274	0.094459	-0.924343
O	3.830182	1.353003	1.815152
O	-0.632588	3.733840	0.936287
O	1.685864	2.963170	1.919809
O	-2.477712	1.124308	-2.494931
C	0.624661	1.887099	0.100034
C	0.722643	0.881965	-0.887014
C	2.859471	0.240476	-0.047350
C	1.694941	2.087060	1.028721
C	2.830259	1.196591	0.908305
C	3.915153	-0.752293	-0.271452
C	-0.506948	2.717652	0.086569
C	-0.260376	0.628918	-1.825325
C	-1.426876	1.401232	-1.801313
C	-1.527237	2.501628	-0.858199
C	3.570828	-2.014442	-0.766942
C	5.255858	-0.452780	-0.013296
C	4.554941	-2.967702	-0.983603
C	6.234812	-1.410627	-0.240717
C	5.888221	-2.668907	-0.720820
H	-0.156330	-0.193101	-2.517945
H	-2.201782	3.317811	-1.075753
H	2.535892	-2.249777	-0.970138
H	5.533899	0.526768	0.344026
H	4.280198	-3.944323	-1.357650
H	7.271043	-1.170049	-0.047141
H	6.654175	-3.412775	-0.892720
H	3.562512	2.074778	2.406073
H	0.159003	3.739911	1.522914
H	-3.388203	1.190797	-1.310588
S	-3.620870	1.453372	0.109965
N	-4.774476	-1.420376	-1.624870
O	-2.598428	-2.027076	-1.483342
O	-2.904537	-3.720466	3.086239
N	-3.047220	-2.430079	1.225920
C	-3.657541	-1.314375	0.530191
C	-2.846820	-0.039254	0.804504
C	-3.639938	-1.627024	-0.961778
C	-3.461082	-2.842062	2.429029
H	-4.677434	-1.200003	0.892440
H	-1.840009	-0.147994	0.401850
H	-2.795423	0.113935	1.879841
H	-2.190368	-2.813747	0.845633
H	-4.361744	-2.334413	2.789000
H	-4.800516	-1.571791	-2.622517
H	-5.609374	-1.115626	-1.150349

SCF Energy: -1768.78330744
Sum of electronic and zero-point Energies= -1768.441492
Sum of electronic and thermal Energies= -1768.415243
Sum of electronic and thermal Enthalpies= -1768.414298
Sum of electronic and thermal Free Energies= -1768.501268

==> ./AOX-III/water/PA/Leu/C5 <==

O	-2.431971	1.095101	-0.612744
O	-2.346251	-1.020265	2.343678
O	1.639716	2.301977	1.647165
O	-0.120301	0.349927	2.648114
O	0.510048	4.215326	-2.491113
C	-0.424714	1.591077	0.625451
C	-1.255597	1.775947	-0.491480
C	-2.818427	0.190255	0.327034
C	-0.786114	0.631013	1.637293
C	-2.047964	-0.072722	1.401771
C	-4.098511	-0.455565	-0.006748
C	0.782829	2.374551	0.633439
C	-0.976096	2.640522	-1.535839
C	0.211543	3.408577	-1.539810
C	1.079629	3.227509	-0.415906
C	-4.331923	-0.872993	-1.319871
C	-5.085932	-0.636467	0.964202
C	-5.534202	-1.481180	-1.650197
C	-6.287820	-1.246926	0.626157
C	-6.512308	-1.672059	-0.678009
H	-1.677662	2.720268	-2.355006
H	2.006251	3.785682	-0.374020
H	-3.568859	-0.727154	-2.072086
H	-4.927409	-0.277744	1.973167
H	-5.707838	-1.808432	-2.666095
H	-7.051194	-1.378664	1.380457
H	-7.448575	-2.146344	-0.938786
H	-3.089891	-1.561684	2.051626
H	2.461477	1.259159	1.438841
O	1.603054	-2.269622	-2.332805
N	4.032921	-1.490834	-1.357683
N	1.109312	-3.284869	-0.373744
C	3.225480	0.361722	1.080687
C	2.342308	-0.403364	0.114814
C	2.951198	-1.717338	-0.423542
C	4.382184	1.132985	0.486279
C	3.555058	-0.375013	2.358974
C	1.832650	-2.465067	-1.136890
H	2.080732	0.223339	-0.742475
H	1.419886	-0.671360	0.634020
H	3.332415	-2.320217	0.396914
H	5.212462	0.463500	0.245309

H	4.084164	1.654049	-0.424509
H	4.754583	1.864342	1.205520
H	4.304406	-1.148684	2.161990
H	3.974340	0.307336	3.099303
H	2.669943	-0.853693	2.779788
C	5.308966	-1.794459	-1.115338
H	3.802812	-1.075575	-2.248861
H	1.327920	-3.412913	0.601271
H	0.298967	-3.742390	-0.762593
O	5.732785	-2.300129	-0.073815
H	5.975392	-1.547423	-1.946445

SCF Energy: -1488.06149937
Sum of electronic and zero-point Energies= -1487.651428
Sum of electronic and thermal Energies= -1487.621919
Sum of electronic and thermal Enthalpies= -1487.620974
Sum of electronic and thermal Free Energies= -1487.715841

==> ./AOX-III/water/PA/Leu/C3 <==

O	-1.702122	1.600953	-0.645447
O	0.305223	1.045916	2.284063
O	-3.984974	-1.785882	1.880907
O	-1.735574	-0.730173	2.693057
O	-5.712026	-0.253659	-2.238016
C	-2.791126	-0.081198	0.684965
C	-2.744748	0.724012	-0.469891
C	-0.713583	1.712578	0.267406
C	-1.765316	-0.005724	1.667685
C	-0.698366	0.969639	1.420728
C	0.352328	2.624484	-0.159370
C	-3.895377	-0.976081	0.791392
C	-3.695280	0.691683	-1.455020
C	-4.804619	-0.199682	-1.338810
C	-4.862159	-1.033982	-0.175587
C	0.734507	2.632200	-1.504759
C	0.998788	3.464201	0.751558
C	1.769822	3.454276	-1.926820
C	2.024108	4.292983	0.318658
C	2.417485	4.283618	-1.016250
H	-3.607386	1.335619	-2.318736
H	-5.689260	-1.722614	-0.065121
H	0.235005	1.981242	-2.209026
H	0.690812	3.475241	1.786045
H	2.071655	3.446423	-2.965103
H	2.516004	4.948147	1.024455
H	3.222677	4.925314	-1.347427
H	1.288847	0.355701	1.849993
H	-3.193451	-1.603218	2.437152
O	0.857089	-2.513803	-2.438217
N	3.236707	-1.667622	-1.341904
N	0.664446	-4.038839	-0.779352
C	2.208327	-0.372615	1.342091
C	1.423951	-1.068209	0.251448
C	2.198252	-2.189845	-0.478266
C	3.256579	0.629215	0.914927
C	2.617488	-1.252482	2.498967
C	1.186729	-2.943313	-1.329712
H	1.092943	-0.350466	-0.503644
H	0.536710	-1.527821	0.696327
H	2.659693	-2.863778	0.238798
H	4.193107	0.117960	0.671748
H	2.935179	1.202027	0.044603
H	3.472657	1.321511	1.731148
H	3.460042	-1.888111	2.206195
H	2.942744	-0.647457	3.346249
H	1.796378	-1.895886	2.817769
C	4.543456	-1.819859	-1.120212
H	2.947834	-1.137618	-2.151693
H	0.962541	-4.355360	0.129261
H	-0.077748	-4.529557	-1.254541
O	5.034907	-2.427545	-0.166893
H	5.167279	-1.343936	-1.882045

SCF Energy: -1488.08130002
Sum of electronic and zero-point Energies= -1487.671498
Sum of electronic and thermal Energies= -1487.642256
Sum of electronic and thermal Enthalpies= -1487.641312
Sum of electronic and thermal Free Energies= -1487.735024

==> ./AOX-III/water/PA/aIP <==

O	-0.205710	-0.899330	0.090639
O	-1.292639	2.518967	-0.187887
O	3.767510	1.772077	-0.100440
O	1.359575	2.821791	-0.178159
O	3.993673	-2.912962	0.188058
C	1.741975	0.482528	-0.011948
C	1.138345	-0.765228	0.069771
C	-1.024639	0.172022	0.020281
C	0.917261	1.656059	-0.087226
C	-0.504117	1.431276	-0.074278
C	-2.442393	-0.190303	0.017672
C	3.191315	0.559316	-0.023671
C	1.881251	-1.934493	0.138036
C	3.332831	-1.856264	0.125920
C	3.952882	-0.561283	0.042037
C	-2.827959	-1.410537	-0.548425
C	-3.405274	0.649929	0.584808
C	-4.165407	-1.775194	-0.560741

C	-4.740080	0.270270	0.576982
C	-5.123240	-0.936455	0.001307
H	1.399206	-2.898733	0.198931
H	5.031353	-0.497009	0.033199
H	-2.084269	-2.059975	-0.987844
H	-3.112005	1.579954	1.046737
H	-4.460306	-2.713338	-1.009992
H	-5.480861	0.917496	1.025532
H	-6.165585	-1.224461	-0.007097
H	-0.704397	3.288101	-0.271117
H	3.061099	2.453750	-0.149184

SCF Energy: -953.281849790
Sum of electronic and zero-point Energies= -953.072265
Sum of electronic and thermal Energies= -953.056816
Sum of electronic and thermal Enthalpies= -953.055872
Sum of electronic and thermal Free Energies= -953.116042

==> ./AOX-III/water/PA/_HAT/C5 <==

O	-0.151724	-0.840729	0.093880
O	-1.318689	2.552015	-0.160762
O	3.836678	1.748644	-0.126058
O	1.259318	2.935878	-0.176101
O	4.005346	-2.943524	0.196421
C	1.769532	0.588861	-0.018569
C	1.183989	-0.659269	0.067454
C	-1.009420	0.209632	0.027943
C	0.915353	1.744937	-0.084426
C	-0.516210	1.468817	-0.058370
C	-2.417054	-0.200471	0.020682
C	3.254120	0.642269	-0.040963
C	1.927047	-1.849155	0.139534
C	3.376371	-1.834271	0.126755
C	3.979563	-0.577240	0.035292
C	-2.762173	-1.437416	-0.534113
C	-3.412504	0.614144	0.567478
C	-4.088091	-1.844449	-0.553876
C	-4.736272	0.195735	0.548665
C	-5.078123	-1.029201	-0.014117
H	1.401370	-2.791002	0.205734
H	5.059357	-0.506832	0.018764
H	-1.995573	-2.070520	-0.957802
H	-3.153063	1.558464	1.020618
H	-4.348227	-2.797761	-0.992953
H	-5.500711	0.827258	0.979957
H	-6.111104	-1.348965	-0.029604
H	-0.716245	3.313539	-0.239982

SCF Energy: -952.824202088
Sum of electronic and zero-point Energies= -952.627416
Sum of electronic and thermal Energies= -952.612039
Sum of electronic and thermal Enthalpies= -952.611095
Sum of electronic and thermal Free Energies= -952.671628

==> ./AOX-III/water/PA/_HAT/C3 <==

O	-0.230732	-0.854718	0.071050
O	-1.252545	2.529405	-0.241825
O	3.806005	1.757972	-0.060248
O	1.433308	2.864444	-0.124343
O	3.955700	-2.948955	0.151143
C	1.745737	0.532148	-0.006798
C	1.142110	-0.743687	0.051439
C	-1.043553	0.204372	0.006438
C	0.967687	1.705072	-0.070953
C	-0.524664	1.531104	-0.101369
C	-2.452915	-0.147772	0.010932
C	3.177575	0.559246	-0.005996
C	1.843144	-1.908352	0.103933
C	3.281513	-1.875085	0.102354
C	3.914407	-0.590844	0.045430
C	-2.833665	-1.422436	-0.438577
C	-3.434009	0.739396	0.478153
C	-4.168750	-1.789495	-0.441143
C	-4.766357	0.355304	0.482648
C	-5.138630	-0.902435	0.018652
H	1.328149	-2.857325	0.150264
H	4.994673	-0.538336	0.043413
H	-2.083421	-2.109979	-0.800886
H	-3.151406	1.708341	0.856051
H	-4.454496	-2.767639	-0.802385
H	-5.516044	1.039417	0.855287
H	-6.180333	-1.192788	0.019239
H	3.111211	2.453915	-0.098062

SCF Energy: -952.843670294
Sum of electronic and zero-point Energies= -952.645980
Sum of electronic and thermal Energies= -952.630942
Sum of electronic and thermal Enthalpies= -952.629998
Sum of electronic and thermal Free Energies= -952.689595

==> ./AOX-III/water/PA/vIP <==

O	-0.198715	-0.904273	0.082853
O	-1.296914	2.540369	-0.179205
O	3.773516	1.784804	-0.102363

O	1.341823	2.837326	-0.179396
O	4.042869	-2.921001	0.195844
C	1.742806	0.503466	-0.015236
C	1.161068	-0.778719	0.064951
C	-1.024188	0.181536	0.015715
C	0.920012	1.652905	-0.088701
C	-0.514370	1.428987	-0.071302
C	-2.444016	-0.192361	0.015597
C	3.166210	0.567963	-0.024003
C	1.899215	-1.933557	0.133813
C	3.322509	-1.865396	0.128164
C	3.926124	-0.565696	0.045162
C	-2.830557	-1.409233	-0.555600
C	-3.411363	0.636474	0.590707
C	-4.167333	-1.781236	-0.564411
C	-4.746352	0.254100	0.583021
C	-5.128721	-0.950905	0.003340
H	1.403893	-2.892247	0.196400
H	5.005363	-0.490111	0.037829
H	-2.086272	-2.055271	-0.999011
H	-3.120200	1.564672	1.058226
H	-4.458176	-2.719850	-1.015957
H	-5.487510	0.897352	1.037273
H	-6.169818	-1.243672	-0.002445
H	-0.682217	3.287282	-0.269281
H	3.068048	2.464096	-0.152208

SCF Energy: -953.277514924

==> ./AOX-III/water/PA <==

O	-0.198715	-0.904273	0.082853
O	-1.296914	2.540369	-0.179205
O	3.773516	1.784804	-0.102363
O	1.341823	2.837326	-0.179396
O	4.042869	-2.921001	0.195844
C	1.742806	0.503466	-0.015236
C	1.161068	-0.778719	0.064951
C	-1.024188	0.181536	0.015715
C	0.920012	1.652905	-0.088701
C	-0.514370	1.428987	-0.071302
C	-2.444016	-0.192361	0.015597
C	3.166210	0.567963	-0.024003
C	1.899215	-1.933557	0.133813
C	3.322509	-1.865396	0.128164
C	3.926124	-0.565696	0.045162
C	-2.830557	-1.409233	-0.555600
C	-3.411363	0.636474	0.590707
C	-4.167333	-1.781236	-0.564411
C	-4.746352	0.254100	0.583021
C	-5.128721	-0.950905	0.003340
H	1.403893	-2.892247	0.196400
H	5.005363	-0.490111	0.037829
H	-2.086272	-2.055271	-0.999011
H	-3.120200	1.564672	1.058226
H	-4.458176	-2.719850	-1.015957
H	-5.487510	0.897352	1.037273
H	-6.169818	-1.243672	-0.002445
H	-0.682217	3.287282	-0.269281
H	3.068048	2.464096	-0.152208

SCF Energy: -953.481420182

Sum of electronic and zero-point Energies= -953.271643

Sum of electronic and thermal Energies= -953.256245

Sum of electronic and thermal Enthalpies= -953.255301

Sum of electronic and thermal Free Energies= -953.314889

==> ./AOX-III/water/PA/Tyr/C5 <==

O	-2.262060	0.265920	0.944795
O	-4.443108	-0.503446	-1.831121
O	-0.974246	-4.145055	-0.410899
O	-3.088930	-2.722370	-1.653440
O	1.117033	-2.026804	3.225940
C	-1.770217	-1.968059	0.205255
C	-1.541049	-0.878093	1.062033
C	-3.221037	0.422703	-0.015584
C	-2.760383	-1.855469	-0.818188
C	-3.479282	-0.582589	-0.875611
C	-3.891955	1.725851	0.064146
C	-0.925612	-3.118157	0.401142
C	-0.576294	-0.858019	2.059745
C	0.231747	-1.992168	2.302347
C	0.019038	-3.115149	1.439526
C	-3.993523	2.368780	1.302551
C	-4.418849	2.341532	-1.075054
C	-4.626410	3.599880	1.399384
C	-5.046303	3.575963	-0.970081
C	-5.156047	4.206271	0.264699
H	-0.467645	0.029400	2.668160
H	0.601092	-4.013373	1.599436
H	-3.585841	1.900176	2.186822
H	-4.323569	1.867035	-2.039538
H	-4.707344	4.085211	2.362360
H	-5.445236	4.047863	-1.857547
H	-5.648083	5.166201	0.342198
H	-4.433401	-1.372702	-2.270048
H	0.151393	-4.232712	-0.916268
O	4.759845	0.262299	-1.704332
O	1.264587	-4.094093	-1.243917
N	3.771268	3.146240	0.191948
N	5.947423	1.443825	-0.188802
C	2.791335	0.971262	0.647417

C	3.541563	1.833878	-0.385607
C	2.413804	-0.381903	0.122745
C	1.367837	-0.501601	-0.810903
C	3.096955	-1.532107	0.543908
C	4.818993	1.122440	-0.821358
C	0.996201	-1.730488	-1.297205
C	2.719392	-2.776190	0.094673
C	1.644066	-2.905093	-0.826035
H	3.414143	0.869450	1.537113
H	1.886776	1.517652	0.918094
H	2.933217	1.943200	-1.281975
H	0.852604	0.391677	-1.142255
H	3.911322	-1.433721	1.249939
H	0.197685	-1.838068	-2.019368
H	3.214422	-3.676482	0.432192
H	6.801504	0.968141	-0.436227
H	5.975144	2.170856	0.507186
C	4.010377	4.230498	-0.555768
H	3.884280	3.226759	1.195370
O	4.298701	5.335080	-0.098738
H	3.916045	4.056912	-1.633040

SCF Energy: -1676.47550789
Sum of electronic and zero-point Energies= -1676.064052
Sum of electronic and thermal Energies= -1676.033581
Sum of electronic and thermal Enthalpies= -1676.032637
Sum of electronic and thermal Free Energies= -1676.130069

==> ./AOX-III/water/PA/Tyr/C3 <==

O	-1.724747	-0.579394	1.482793
O	-3.467338	-0.677499	-1.651376
O	0.628205	-3.750056	-1.245828
O	-1.604136	-2.647081	-2.024624
O	2.225459	-2.576098	3.035402
C	-0.607483	-2.166341	0.061129
C	-0.676989	-1.443726	1.268971
C	-2.640326	-0.330956	0.537466
C	-1.588619	-2.000045	-0.947435
C	-2.603705	-0.968884	-0.695152
C	-3.611757	0.683824	0.942227
C	0.508134	-3.049350	-0.092088
C	0.236038	-1.564082	2.276779
C	1.351238	-2.455458	2.120138
C	1.446378	-3.189454	0.892603
C	-3.191436	1.710051	1.797620
C	-4.941362	0.639495	0.509576
C	-4.087442	2.688819	2.197958
C	-5.833953	1.615607	0.928302
C	-5.409529	2.643088	1.764766
H	0.129886	-0.988680	3.185520
H	2.282175	-3.860538	0.747461
H	-2.162640	1.746272	2.127648
H	-5.276071	-0.162616	-0.129280
H	-3.755047	3.487481	2.846456
H	-6.863852	1.571835	0.602354
H	-6.108342	3.405237	2.081567
H	-2.957088	0.014649	-2.377602
H	-0.149931	-3.519173	-1.806416
O	3.719276	3.704889	-0.598942
O	-2.156856	0.787406	-3.026892
N	4.425654	1.338232	1.224255
N	3.983473	1.924583	-1.955939
C	2.576863	0.254152	-0.031477
C	3.263255	1.566541	0.366409
C	1.339021	0.461142	-0.858216
C	1.203955	-0.180295	-2.106698
C	0.298523	1.273606	-0.399456
C	3.667319	2.486816	-0.790080
C	0.060595	-0.049990	-2.856676
C	-0.859565	1.415657	-1.140409
C	-1.018135	0.731929	-2.376315
H	3.268335	-0.406341	-0.555348
H	2.301605	-0.243659	0.902431
H	2.575376	2.160484	0.960893
H	2.018748	-0.796559	-2.466787
H	0.382802	1.775947	0.555950
H	-0.054739	-0.562630	-3.801975
H	-1.673740	2.038666	-0.793849
H	4.285372	2.513111	-2.717825
H	3.944335	0.928738	-2.100087
C	5.497868	0.627535	0.863326
H	4.436385	1.766187	2.139666
O	6.478631	0.449412	1.587491
H	5.451261	0.201175	-0.142792

SCF Energy: -1676.48652491
Sum of electronic and zero-point Energies= -1676.073581
Sum of electronic and thermal Energies= -1676.043667
Sum of electronic and thermal Enthalpies= -1676.042723
Sum of electronic and thermal Free Energies= -1676.137522

==> ./AOX-III/water/PA/PA/Cys/C5 <==

O	-2.585695	-0.929956	0.548482
O	-2.648626	1.655297	-1.858158
O	1.581327	-1.404218	-1.830250
O	-0.263343	0.620095	-2.422344
O	0.613807	-4.004977	1.978584
C	-0.498331	-1.030832	-0.709693

C	-1.328665	-1.471304	0.352872
C	-3.065414	0.069744	-0.190752
C	-0.905460	0.067849	-1.510788
C	-2.269567	0.660027	-1.213746
C	-4.404612	0.487888	0.194508
C	0.777154	-1.716266	-0.846354
C	-0.993416	-2.441012	1.247731
C	0.281238	-3.095961	1.142478
C	1.124246	-2.701185	0.073196
C	-4.846422	0.229212	1.501766
C	-5.274493	1.110026	-0.712918
C	-6.120391	0.604518	1.895116
C	-6.551932	1.471018	-0.311111
C	-6.976911	1.226743	0.990888
H	-1.683868	-2.721032	2.030882
H	2.075015	-3.204543	-0.047293
H	-4.182959	-0.249132	2.207391
H	-4.958893	1.287425	-1.728086
H	-6.446010	0.412583	2.908124
H	-7.220041	1.940132	-1.020092
H	-7.972456	1.515788	1.298879
H	2.874416	-1.401388	-1.517074
S	4.382622	-1.311247	-1.220660
N	2.537922	1.638551	-1.744592
O	1.692927	1.294569	0.324785
O	5.452656	3.016572	2.849390
N	4.169728	1.894874	1.349154
C	4.061126	1.165016	0.094583
C	4.307606	-0.332791	0.318234
C	2.656639	1.375461	-0.443531
C	5.299108	2.477617	1.749970
H	4.799787	1.561161	-0.599433
H	3.534139	-0.726581	0.978018
H	5.268881	-0.430306	0.819985
H	3.395383	1.834035	1.998554
H	6.101856	2.454889	1.005570
H	1.619256	1.516885	-2.157085
H	3.346560	1.542422	-2.338323

SCF Energy: -1767.88977783
Sum of electronic and zero-point Energies= -1767.574571
Sum of electronic and thermal Energies= -1767.548483
Sum of electronic and thermal Enthalpies= -1767.547538
Sum of electronic and thermal Free Energies= -1767.635046

==> ./AOX-III/water/PA/PA/His/C5 <==

O	-2.091707	1.162203	-0.439701
O	-1.963074	-2.335331	-1.348017
O	2.395666	0.494487	-1.957189
O	0.399658	-1.461900	-2.245145
O	0.946249	4.720082	-0.502591
C	0.073015	0.713054	-1.343883
C	-0.861060	1.586667	-0.768339
C	-2.482250	-0.154008	-0.580462
C	-0.315705	-0.638635	-1.634170
C	-1.655808	-1.095472	-1.163182
C	-3.843778	-0.355378	-0.084091
C	1.382911	1.269843	-1.576161
C	-0.595294	2.922176	-0.476210
C	0.666662	3.491472	-0.759499
C	1.644510	2.603732	-1.306022
C	-4.654973	0.757049	0.203385
C	-4.377961	-1.635341	0.137178
C	-5.946631	0.592964	0.681346
C	-5.672193	-1.788508	0.619507
C	-6.466621	-0.680794	0.892353
H	-1.374028	3.524444	-0.027555
H	2.647295	2.972378	-1.480954
H	-4.274882	1.755077	0.045807
H	-3.771743	-2.502229	-0.062120
H	-6.550148	1.466615	0.889176
H	-6.058341	-2.785549	0.785598
H	-7.474034	-0.806333	1.265299
H	2.890906	0.052521	-0.929420
O	4.253060	-3.327551	0.029929
N	1.327030	0.787141	1.569987
N	2.439589	-2.257105	1.757279
N	2.193849	2.706404	1.990091
N	2.981588	-2.690670	-1.738488
C	3.191199	-0.346017	0.333007
C	2.522868	-1.712045	0.413176
C	2.626231	0.738199	1.088416
C	3.328698	-2.671907	-0.457457
C	3.165678	1.978363	1.365150
C	1.081143	1.980397	2.087647
H	4.277678	-0.354701	0.342966
H	1.505866	-1.647427	0.030451
H	0.660211	0.011050	1.551990
H	4.144058	2.370996	1.157152
H	0.163872	2.291749	2.553963
H	2.175511	-2.160145	-2.059697
H	3.509233	-3.258841	-2.383904
H	2.273526	3.670867	2.286103
C	1.295309	-2.308067	2.440006
H	3.252080	-2.729240	2.129270
O	0.235558	-1.793813	2.062093
H	1.369570	-2.843504	3.388579

SCF Energy: -1595.20152545
Sum of electronic and zero-point Energies= -1594.821881

Sum of electronic and thermal Energies= -1594.794159
Sum of electronic and thermal Enthalpies= -1594.793215
Sum of electronic and thermal Free Energies= -1594.882168

==> ./AOX-III/water/PA/PA <==

O	-0.209061	-0.852822	0.058814
O	-1.257140	2.626503	-0.117707
O	3.777775	1.775915	-0.081215
O	1.437715	2.848965	-0.124219
O	3.997364	-2.944132	0.141991
C	1.733699	0.520787	-0.014276
C	1.136365	-0.751360	0.046160
C	-1.036110	0.260819	0.016249
C	0.931782	1.692828	-0.063282
C	-0.542500	1.546370	-0.049909
C	-2.444909	-0.137962	0.007753
C	3.160649	0.562774	-0.021635
C	1.867048	-1.921227	0.097387
C	3.285654	-1.873758	0.091883
C	3.907544	-0.581730	0.028700
C	-2.798854	-1.449452	-0.352040
C	-3.471798	0.747903	0.369243
C	-4.126770	-1.851585	-0.361677
C	-4.798401	0.334798	0.362055
C	-5.136939	-0.962813	-0.005864
H	1.358202	-2.874114	0.145385
H	4.987867	-0.519340	0.022127
H	-2.030380	-2.153988	-0.633288
H	-3.222982	1.753498	0.664484
H	-4.372586	-2.865175	-0.649660
H	-5.571479	1.034428	0.651201
H	-6.171360	-1.278430	-0.012943
H	3.050729	2.449332	-0.113106

SCF Energy: -953.010789239
Sum of electronic and zero-point Energies= -952.813954
Sum of electronic and thermal Energies= -952.799072
Sum of electronic and thermal Enthalpies= -952.798127
Sum of electronic and thermal Free Energies= -952.856587

==> ./AOX-III/water/PA/PA/2dG-C4p/C5 <==

O	2.457793	-0.305833	1.352046
O	3.495817	-1.634586	-1.852568
O	-0.687820	-3.802349	0.433911
O	1.638394	-3.420884	-1.106745
O	-0.807935	-1.451996	4.508170
C	1.058963	-2.180812	0.836996
C	1.444585	-1.131020	1.686010
C	3.136894	-0.433348	0.155436
C	1.822209	-2.443923	-0.355007
C	2.888240	-1.465501	-0.721974
C	4.074447	0.673990	-0.041383
C	-0.105221	-2.917660	1.254227
C	0.841461	-0.866862	2.910081
C	-0.237546	-1.653194	3.369788
C	-0.700549	-2.668434	2.476392
C	3.854079	1.895001	0.617196
C	5.198740	0.561224	-0.872821
C	4.720748	2.964511	0.440720
C	6.063565	1.635717	-1.040392
C	5.829931	2.843372	-0.391137
H	1.210901	-0.046652	3.511200
H	-1.585550	-3.233290	2.741285
H	2.994135	2.007877	1.261600
H	5.396824	-0.371920	-1.374111
H	4.525469	3.897750	0.952204
H	6.928332	1.524548	-1.681195
H	6.504109	3.677846	-0.528840
H	-1.515767	-3.161172	-0.243366
O	-1.567523	-1.044634	-0.217966
O	-1.205202	-3.072843	-2.656780
O	-4.439055	-1.213402	-0.464461
O	-1.782565	5.246850	0.584606
N	-0.852377	1.030875	-0.927715
N	0.081468	2.988027	-0.387224
N	-3.189304	1.507567	-0.450311
N	-3.455040	3.726422	0.303022
N	-5.277797	2.323167	0.082370
C	-0.921538	-0.359592	-1.298966
C	-2.190077	-2.135448	-2.222207
C	-1.773723	-0.693984	-2.513631
C	-2.243939	-2.162461	-0.698245
C	-3.586348	-2.274739	-0.027154
C	-1.883907	1.841176	-0.534838
C	0.301898	1.772814	-0.797503
C	-1.288576	3.048860	-0.214656
C	-2.113751	4.108249	0.249114
C	-3.949636	2.493193	-0.022086
H	0.103689	-0.710722	-1.403697
H	-3.159912	-2.384364	-2.649479
H	-2.652838	-0.052122	-2.532892
H	-1.222170	-0.600971	-3.446024
H	-3.453553	-2.243344	1.055501
H	-4.062720	-3.211338	-0.306291
H	1.260658	1.344296	-1.044106
H	-1.153855	-3.021009	-3.617041
H	-3.966119	-0.367350	-0.367507
H	-4.100890	4.434556	0.632660
H	-5.831217	2.993494	0.591903

H -5.629115 1.382109 0.001723

SCF Energy: -1916.12959789
Sum of electronic and zero-point Energies= -1915.693630
Sum of electronic and thermal Energies= -1915.661418
Sum of electronic and thermal Enthalpies= -1915.660474
Sum of electronic and thermal Free Energies= -1915.758621

==> ./AOX-III/water/PA/PA/Met/C5 <==

O	3.290910	1.023413	-0.234341
O	3.278301	-2.284024	1.226050
O	-1.241267	0.415268	1.231967
O	0.725023	-1.566839	1.536869
O	0.132216	4.427770	-0.795096
C	1.067615	0.569992	0.541951
C	2.014823	1.419968	-0.056480
C	3.743751	-0.217075	0.173025
C	1.477908	-0.736023	0.989467
C	2.909638	-1.122829	0.793289
C	5.170233	-0.378770	-0.112865
C	-0.257912	1.120600	0.685480
C	1.729610	2.700629	-0.512352
C	0.431915	3.242377	-0.384341
C	-0.542737	2.398442	0.229148
C	5.936047	0.733500	-0.507234
C	5.818839	-1.622530	-0.023570
C	7.288995	0.607391	-0.786913
C	7.173463	-1.738744	-0.310488
C	7.920138	-0.629216	-0.690082
H	2.520117	3.284106	-0.965303
H	-1.556048	2.764630	0.344851
H	5.471171	1.704182	-0.589385
H	5.254367	-2.492152	0.266017
H	7.852842	1.482530	-1.081793
H	7.646223	-2.709311	-0.236634
H	8.974995	-0.725383	-0.908567
H	-1.936924	-0.148829	0.246128
S	-2.024998	-2.094534	-1.277019
O	-4.261058	1.651659	1.541381
N	-6.078104	0.514913	-0.262221
N	-4.055107	-0.456959	2.336519
C	-3.799428	0.140803	-1.054744
C	-4.819739	-0.139736	0.061335
C	-2.395782	-0.423066	-0.892694
C	-4.345363	0.430477	1.388296
C	-3.443543	-3.044107	-0.682068
H	-3.701832	1.225106	-1.129654
H	-4.233965	-0.217251	-1.991146
H	-5.020452	-1.199946	0.165464
H	-1.677224	0.172116	-1.461096
H	-3.651811	-0.137661	3.204683
H	-4.104811	-1.445995	2.152200
H	-3.567239	-2.910903	0.390327
H	-3.209057	-4.086122	-0.887584
H	-4.346202	-2.766889	-1.221664
C	-6.994194	-0.058977	-1.044371
H	-6.193551	1.485238	-0.005844
O	-6.903235	-1.200474	-1.501491
H	-7.860937	0.576587	-1.244222

SCF Energy: -1846.46940365
Sum of electronic and zero-point Energies= -1846.097542
Sum of electronic and thermal Energies= -1846.068757
Sum of electronic and thermal Enthalpies= -1846.067812
Sum of electronic and thermal Free Energies= -1846.161194

==> ./AOX-III/water/PA/PA/_HAT/C5 <==

O	-0.191291	-0.808097	0.092239
O	-1.271513	2.539377	-0.235432
O	3.907462	1.745530	-0.087079
O	1.353271	2.959507	-0.102919
O	3.942473	-2.959141	0.145690
C	1.793335	0.613409	-0.007844
C	1.185415	-0.669039	0.059735
C	-1.024865	0.228942	0.018385
C	0.979490	1.771384	-0.059930
C	-0.521408	1.555113	-0.092796
C	-2.430005	-0.151258	0.015062
C	3.263561	0.641253	-0.022962
C	1.861349	-1.847615	0.111785
C	3.303368	-1.844051	0.097652
C	3.943627	-0.593376	0.030594
C	-2.782918	-1.438347	-0.420473
C	-3.435020	0.721092	0.457930
C	-4.111035	-1.832362	-0.432696
C	-4.760490	0.312111	0.451087
C	-5.104363	-0.958804	0.001513
H	1.321842	-2.782840	0.166686
H	5.026710	-0.567260	0.017813
H	-2.015847	-2.117076	-0.763924
H	-3.176138	1.701341	0.823158
H	-4.372415	-2.821552	-0.782562
H	-5.527288	0.988209	0.803409
H	-6.140330	-1.268955	-0.006835

SCF Energy: -952.369234032
Sum of electronic and zero-point Energies= -952.184373

Sum of electronic and thermal Energies= -952.169466
Sum of electronic and thermal Enthalpies= -952.168522
Sum of electronic and thermal Free Energies= -952.228145

==> ./AOX-III/water/PA/PA/aIP <==

O	-0.230732	-0.854718	0.071050
O	-1.252545	2.529405	-0.241825
O	3.806005	1.757972	-0.060247
O	1.433308	2.864444	-0.124343
O	3.955699	-2.948956	0.151143
C	1.745737	0.532148	-0.006798
C	1.142110	-0.743687	0.051438
C	-1.043553	0.204372	0.006438
C	0.967687	1.705072	-0.070953
C	-0.524664	1.531104	-0.101369
C	-2.452915	-0.147771	0.010932
C	3.177575	0.559246	-0.005996
C	1.843144	-1.908352	0.103932
C	3.281513	-1.875085	0.102354
C	3.914407	-0.590844	0.045430
C	-2.833665	-1.422436	-0.438577
C	-3.434009	0.739397	0.478152
C	-4.168750	-1.789496	-0.441143
C	-4.766357	0.355304	0.482648
C	-5.138630	-0.902435	0.018652
H	1.328148	-2.857325	0.150263
H	4.994673	-0.538337	0.043413
H	-2.083420	-2.109979	-0.800885
H	-3.151406	1.708341	0.856051
H	-4.454496	-2.767639	-0.802384
H	-5.516044	1.039418	0.855287
H	-6.180332	-1.192788	0.019239
H	3.111211	2.453915	-0.098062

SCF Energy: -952.843670294
Sum of electronic and zero-point Energies= -952.645980
Sum of electronic and thermal Energies= -952.630942
Sum of electronic and thermal Enthalpies= -952.629998
Sum of electronic and thermal Free Energies= -952.689595

==> ./AOX-III/water/PA/PA/Leu/C5 <==

O	3.189202	0.818599	0.162263
O	2.170229	-2.329734	-1.303455
O	-1.128190	1.735963	-1.720383
O	-0.015577	-0.832678	-1.684353
O	1.307938	5.091764	0.490527
C	1.013116	1.115795	-0.790344
C	2.139142	1.614527	-0.116628
C	3.233251	-0.508996	-0.224525
C	0.966773	-0.275856	-1.155315
C	2.182822	-1.104196	-0.889587
C	4.511018	-1.121524	0.142794
C	-0.033880	2.073248	-1.040589
C	2.261835	2.929443	0.319324
C	1.231982	3.867184	0.096061
C	0.079107	3.380726	-0.597189
C	5.577718	-0.311215	0.572216
C	4.719039	-2.510627	0.098947
C	6.797973	-0.865942	0.929827
C	5.943631	-3.056424	0.464175
C	6.992224	-2.242997	0.878319
H	3.164078	3.227045	0.836910
H	-0.737307	4.062204	-0.800694
H	5.452891	0.759951	0.619822
H	3.916807	-3.155301	-0.216751
H	7.601882	-0.216445	1.250256
H	6.074895	-4.129823	0.424866
H	7.944140	-2.673634	1.157841
H	-1.981784	1.179883	-0.895219
O	-4.902360	-3.144961	-0.730339
N	-4.231526	-1.555898	1.411497
N	-6.327360	-1.546520	-1.454799
C	-2.768893	0.821286	0.009327
C	-3.434586	-0.382927	-0.629021
C	-4.637271	-0.935723	0.167043
C	-1.848702	0.567404	1.178779
C	-3.651511	2.036121	0.176803
C	-5.314731	-1.982670	-0.705656
H	-2.711483	-1.190896	-0.757240
H	-3.803747	-0.097920	-1.617018
H	-5.337792	-0.137806	0.398573
H	-2.419307	0.495580	2.109757
H	-1.274063	-0.349629	1.048635
H	-1.155445	1.404874	1.302119
H	-4.336435	1.896508	1.019103
H	-3.044596	2.918189	0.390635
H	-4.243264	2.224147	-0.720163
C	-4.529127	-1.079824	2.621465
H	-3.662281	-2.388115	1.354860
H	-6.631912	-0.587615	-1.407710
H	-6.756558	-2.168998	-2.122174
O	-5.198354	-0.068088	2.842606
H	-4.115204	-1.683066	3.434415

SCF Energy: -1487.59481462
Sum of electronic and zero-point Energies= -1487.197994
Sum of electronic and thermal Energies= -1487.168806

Sum of electronic and thermal Enthalpies= -1487.167861
Sum of electronic and thermal Free Energies= -1487.262810

==> ./AOX-III/water/PA/PA/Tyr/C5 <==

O	-0.929967	-1.842495	0.884482
O	-0.829041	-2.210960	-2.628223
O	-4.527135	0.955095	-0.701326
O	-3.106659	-0.825254	-2.402237
O	-4.037211	-0.041277	3.873539
C	-2.818123	-0.623649	-0.046591
C	-2.103167	-1.134684	1.060855
C	-0.420975	-2.098113	-0.324385
C	-2.470544	-1.039286	-1.361784
C	-1.186026	-1.833333	-1.499559
C	0.914288	-2.668960	-0.290117
C	-3.916099	0.256001	0.266945
C	-2.474897	-0.960478	2.362487
C	-3.650313	-0.200332	2.667915
C	-4.328748	0.411004	1.570936
C	1.381140	-3.251824	0.899386
C	1.780182	-2.574580	-1.391523
C	2.672970	-3.745919	0.975551
C	3.074656	-3.064187	-1.300303
C	3.524125	-3.654059	-0.122639
H	-1.879825	-1.382712	3.160103
H	-5.156792	1.075795	1.780241
H	0.726175	-3.323046	1.755224
H	1.448004	-2.101688	-2.301080
H	3.018016	-4.201807	1.893224
H	3.737108	-2.978145	-2.150752
H	4.534378	-4.034856	-0.058557
H	-3.786226	1.519762	-1.319526
O	4.846403	1.511594	1.903123
O	-2.893512	2.252775	-1.960635
N	3.117636	2.916693	0.362859
N	4.806605	-0.260518	0.500305
C	1.883913	0.802197	0.722364
C	3.162799	1.480221	0.189238
C	0.655036	1.273437	-0.009817
C	-0.340400	2.003556	0.639314
C	0.481857	0.999128	-1.369029
C	4.361858	0.919963	0.935620
C	-1.499889	2.389959	-0.022652
C	-0.668807	1.384582	-2.042734
C	-1.713470	2.036520	-1.365182
H	2.000193	-0.278449	0.613455
H	1.793573	1.025989	1.786983
H	3.267589	1.267096	-0.873796
H	-0.215843	2.253603	1.686729
H	1.251929	0.454216	-1.904750
H	-2.282755	2.919720	0.506490
H	-0.808097	1.124305	-3.084752
H	5.527609	-0.739934	1.017531
H	4.351561	-0.739246	-0.261825
C	2.647598	3.739747	-0.576990
H	3.222724	3.277016	1.302997
O	2.426414	4.940654	-0.399019
H	2.492859	3.266634	-1.551557

SCF Energy: -1676.04231053
Sum of electronic and zero-point Energies= -1675.642220
Sum of electronic and thermal Energies= -1675.612241
Sum of electronic and thermal Enthalpies= -1675.611297
Sum of electronic and thermal Free Energies= -1675.706647

==> ./AOX-III/water/PA/PA/Tyr/C5/IRC <==

O	-0.880589	-1.840889	0.889983
O	-0.781687	-2.212910	-2.620700
O	-4.513546	0.903216	-0.679484
O	-3.052140	-0.831338	-2.407316
O	-3.965577	-0.044654	3.898695
C	-2.792020	-0.646923	-0.042680
C	-2.060051	-1.139712	1.067151
C	-0.375420	-2.102290	-0.318115
C	-2.432210	-1.051813	-1.351917
C	-1.143330	-1.840216	-1.490433
C	0.961638	-2.672702	-0.284799
C	-3.908888	0.243918	0.261910
C	-2.417081	-0.957158	2.368580
C	-3.598510	-0.195846	2.679341
C	-4.291091	0.395820	1.597522
C	1.429748	-3.255414	0.904120
C	1.827746	-2.578867	-1.385988
C	2.722152	-3.748810	0.980396
C	3.122735	-3.067929	-1.295107
C	3.573385	-3.657225	-0.117647
H	-1.812045	-1.368567	3.164419
H	-5.132834	1.041868	1.814811
H	0.775284	-3.326841	1.760390
H	1.494853	-2.107297	-2.295951
H	3.067250	-4.204190	1.898366
H	3.784539	-2.982102	-2.146151
H	4.583794	-4.037657	-0.053707
H	-3.532135	1.787178	-1.522842
O	4.894976	1.513130	1.903550
O	-2.812597	2.313025	-2.005485
N	3.160501	2.914122	0.368615
N	4.848285	-0.265340	0.508967
C	1.930378	0.795660	0.723389
C	3.207669	1.477983	0.193016

C	0.701415	1.268087	-0.008559
C	-0.297785	1.984735	0.647722
C	0.541084	0.998438	-1.369404
C	4.407175	0.917645	0.940479
C	-1.455341	2.373925	-0.018642
C	-0.606469	1.388752	-2.046585
C	-1.626109	2.040876	-1.358983
H	2.044202	-0.283845	0.604007
H	1.837678	1.011320	1.789110
H	3.316412	1.266878	-0.870044
H	-0.179778	2.222525	1.697884
H	1.313958	0.455861	-1.901818
H	-2.245560	2.898780	0.502701
H	-0.748007	1.140802	-3.090313
H	5.572397	-0.742299	1.024148
H	4.393189	-0.745181	-0.252491
C	2.694666	3.739519	-0.571534
H	3.278432	3.275270	1.306897
O	2.479034	4.941137	-0.393924
H	2.536243	3.266411	-1.545604

SCF Energy: -1676.04635648

==> ./AOX-III/water/PA/PA/vIP <==

O	-0.209061	-0.852822	0.058814
O	-1.257140	2.626503	-0.117707
O	3.777775	1.775915	-0.081215
O	1.437715	2.848965	-0.124219
O	3.997364	-2.944132	0.141991
C	1.733699	0.520787	-0.014276
C	1.136365	-0.751360	0.046160
C	-1.036110	0.260819	0.016249
C	0.931782	1.692828	-0.063282
C	-0.542500	1.546370	-0.049909
C	-2.444909	-0.137962	0.007753
C	3.160649	0.562774	-0.021635
C	1.867048	-1.921227	0.097387
C	3.285654	-1.873758	0.091883
C	3.907544	-0.581730	0.028700
C	-2.798854	-1.449452	-0.352040
C	-3.471798	0.747903	0.369243
C	-4.126770	-1.851585	-0.361677
C	-4.798401	0.334798	0.362055
C	-5.136939	-0.962813	-0.005864
H	1.358202	-2.874114	0.145385
H	4.987867	-0.519340	0.022127
H	-2.030380	-2.153988	-0.633288
H	-3.222982	1.753498	0.664484
H	-4.372586	-2.865175	-0.649660
H	-5.571479	1.034428	0.651201
H	-6.171360	-1.278430	-0.012943
H	3.050729	2.449332	-0.113106

SCF Energy: -952.834559653

==> ./AOX-III/water/PA/Met/C3 <==

O	-2.691221	0.516875	-0.309227
O	0.808593	0.923334	-1.051141
O	-0.914971	-3.914304	-0.797944
O	0.550026	-1.789368	-1.165489
O	-5.415481	-3.232032	0.461795
C	-1.732127	-1.668194	-0.581795
C	-2.831500	-0.845606	-0.267837
C	-1.497449	1.099256	-0.577168
C	-0.468291	-1.100298	-0.901306
C	-0.379164	0.362177	-0.872228
C	-1.554397	2.561837	-0.470589
C	-1.948746	-3.075473	-0.517142
C	-4.066609	-1.332676	0.074142
C	-4.279051	-2.742048	0.136117
C	-3.168773	-3.592897	-0.176294
C	-2.409166	3.142000	0.472739
C	-0.784840	3.382418	-1.300853
C	-2.478832	4.522556	0.593908
C	-0.867555	4.762834	-1.180019
C	-1.707989	5.335956	-0.230855
H	-4.875973	-0.653432	0.302149
H	-3.303338	-4.665575	-0.133958
H	-3.005508	2.510748	1.116428
H	-0.139629	2.941002	-2.044556
H	-3.134254	4.963145	1.332625
H	-0.276384	5.391824	-1.831395
H	-1.765071	6.411900	-0.136852
H	1.277520	1.115029	0.079508
H	-0.136130	-3.345656	-1.001643
S	0.206818	0.802900	2.233169
O	3.886121	1.736249	-0.743264
N	4.533797	-1.132667	0.807327
N	3.888217	-0.187304	-1.931332
C	2.953634	0.546575	1.602997
C	3.381964	-0.323562	0.427255
C	1.640721	1.280849	1.356759
C	3.732744	0.515018	-0.807446
C	0.384106	-0.992048	2.408720
H	3.734545	1.280610	1.800694
H	2.879521	-0.099787	2.476492
H	2.581289	-1.006374	0.147700
H	1.712971	2.364918	1.397691
H	4.151681	0.285810	-2.781738
H	3.737765	-1.185708	-1.932930
H	0.729851	-1.434859	1.478334
H	-0.605080	-1.374151	2.650802
H	1.070672	-1.225878	3.218571
C	4.759177	-2.350205	0.309329

H	5.185093	-0.762403	1.484452
O	4.053129	-2.891622	-0.545283
H	5.640561	-2.841607	0.728776

SCF Energy: -1846.96598348
Sum of electronic and zero-point Energies= -1846.581056
Sum of electronic and thermal Energies= -1846.552281
Sum of electronic and thermal Enthalpies= -1846.551337
Sum of electronic and thermal Free Energies= -1846.643718

==> ./AOX-III/water/PA/Met/C5 <==

O	2.816910	0.978013	-0.309316
O	2.570928	-1.866346	1.903606
O	-1.508848	1.430015	1.760196
O	0.299639	-0.693273	2.384730
O	0.076646	4.650102	-1.266193
C	0.687671	1.091833	0.823577
C	1.635859	1.619001	-0.074601
C	3.140273	-0.187711	0.319423
C	0.996547	-0.115030	1.523817
C	2.285206	-0.730495	1.208874
C	4.468079	-0.686629	-0.060338
C	-0.540405	1.835752	0.956561
C	1.463121	2.795153	-0.779546
C	0.276128	3.552483	-0.630629
C	-0.713140	3.016232	0.251473
C	5.455251	0.224087	-0.451912
C	4.759490	-2.053946	-0.053758
C	6.716533	-0.226772	-0.814663
C	6.022727	-2.497300	-0.422799
C	7.004843	-1.587764	-0.800022
H	2.242378	3.130214	-1.450251
H	-1.647591	3.549781	0.369422
H	5.235945	1.282163	-0.462783
H	3.998568	-2.768036	0.221023
H	7.474986	0.485914	-1.108342
H	6.236649	-3.557365	-0.421485
H	7.987783	-1.937299	-1.085163
H	1.806714	-1.996412	2.492737
H	-2.197327	0.288450	1.378168
S	-4.252043	-0.550297	2.318284
O	-1.172829	0.200091	-1.787633
N	-3.133204	-1.764172	-2.299828
N	-2.978288	1.538388	-1.536805
C	-2.734953	-1.555451	0.094532
C	-3.230331	-0.845655	-1.173556
C	-2.765042	-0.806423	1.419079
C	-2.366172	0.357025	-1.517616
C	-5.496956	-0.081609	1.095704
H	-1.695917	-1.839405	-0.083796
H	-3.313416	-2.476888	0.195452
H	-4.268057	-0.546395	-1.091339
H	-2.096632	-1.294578	2.126678
H	-2.435474	2.371721	-1.708736
H	-3.952396	1.625894	-1.296823
H	-5.161678	0.782398	0.526799
H	-6.384218	0.187006	1.664672
H	-5.729702	-0.919874	0.443475
C	-4.109864	-2.623597	-2.597690
H	-2.243364	-1.856261	-2.769085
O	-5.195578	-2.672908	-2.015709
H	-3.868244	-3.290435	-3.429648

SCF Energy: -1846.94221572
Sum of electronic and zero-point Energies= -1846.557137
Sum of electronic and thermal Energies= -1846.528102
Sum of electronic and thermal Enthalpies= -1846.527158
Sum of electronic and thermal Free Energies= -1846.620358

==> ./AOX-III/water/PA/His/C5 <==

O	-2.879974	0.830562	0.545197
O	-2.895993	-2.706927	-0.188937
O	1.515170	-0.686057	1.743384
O	-0.438358	-2.398831	0.628691
O	0.398122	3.879707	1.919626
C	-0.708857	-0.057233	1.091728
C	-1.600290	1.020053	0.969827
C	-3.336468	-0.398869	0.174210
C	-1.128186	-1.360286	0.670731
C	-2.512501	-1.467151	0.220386
C	-4.730409	-0.369774	-0.285356
C	0.610595	0.267733	1.570173
C	-1.262721	2.332900	1.252082
C	0.043853	2.670511	1.687303
C	0.954548	1.580570	1.849185
C	-5.231574	0.794302	-0.877844
C	-5.569287	-1.476706	-0.127060
C	-6.546498	0.841865	-1.318290
C	-6.885440	-1.419053	-0.565980
C	-7.376569	-0.264155	-1.165118
H	-2.005847	3.107596	1.120445
H	1.960085	1.784123	2.194543
H	-4.589588	1.654846	-1.000461
H	-5.201488	-2.370648	0.352545
H	-6.922989	1.743356	-1.781912
H	-7.529579	-2.277329	-0.432585
H	-8.401689	-0.224370	-1.507326
H	-2.099457	-3.259342	-0.097620

H	2.110871	-0.848338	0.652639
O	4.934626	-3.171263	-0.384122
N	2.806284	1.708186	-0.859244
N	5.020834	-0.703396	-1.401676
N	0.838935	2.118501	-1.614805
N	4.102695	-2.473065	1.599527
C	2.692985	-0.781642	-0.523073
C	4.184343	-0.894219	-0.231436
C	2.173934	0.480253	-0.989888
C	4.453597	-2.291317	0.328485
C	0.915621	0.761244	-1.472312
C	1.987369	2.674717	-1.237934
H	2.260290	-1.636315	-1.038270
H	4.471878	-0.151064	0.511949
H	3.788582	1.838858	-0.601026
H	0.103357	0.103224	-1.721451
H	2.214613	3.725722	-1.256499
H	3.693467	-1.727653	2.141811
H	4.182684	-3.392284	2.007264
H	0.028542	2.637310	-1.929086
C	5.707541	0.415806	-1.632517
H	5.203293	-1.513167	-1.978711
O	5.611957	1.445517	-0.955273
H	6.373828	0.354945	-2.495220

SCF Energy: -1595.65500631
Sum of electronic and zero-point Energies= -1595.263003
Sum of electronic and thermal Energies= -1595.234514
Sum of electronic and thermal Enthalpies= -1595.233570
Sum of electronic and thermal Free Energies= -1595.325405

==> ./AOX-III/water/PA/His/C3 <==

O	1.765910	1.552615	-0.354108
O	-0.623706	-0.780864	-1.672797
O	4.172954	-2.556136	-1.007276
O	1.635083	-2.311656	-1.628813
O	6.271716	1.204557	0.922396
C	2.905061	-0.535997	-0.729226
C	2.910230	0.793243	-0.261378
C	0.617822	1.042815	-0.836578
C	1.715653	-1.121894	-1.242019
C	0.526783	-0.259602	-1.271408
C	-0.487429	2.004638	-0.813987
C	4.123581	-1.262004	-0.592008
C	4.006290	1.400791	0.286859
C	5.228835	0.671037	0.413837
C	5.239245	-0.684925	-0.048397
C	-0.600547	2.893569	0.260580
C	-1.419800	2.051115	-1.856344
C	-1.653739	3.795543	0.305088
C	-2.462900	2.966159	-1.809360
C	-2.586359	3.832372	-0.727340
H	3.950683	2.426247	0.623923
H	6.150389	-1.261186	0.041366
H	0.124083	2.863025	1.062825
H	-1.316047	1.387078	-2.701468
H	-1.745776	4.470884	1.144453
H	-3.176533	3.004748	-2.620784
H	-3.405048	4.538189	-0.691081
H	-1.140189	-1.328861	-0.733856
H	3.277559	-2.788261	-1.339733
O	-3.829906	-3.581410	-0.055141
N	-0.795497	0.010472	2.039345
N	-3.243152	-0.017782	0.278195
N	1.193196	-0.657311	2.484893
N	-4.566728	-1.846120	-1.301175
C	-1.582176	-1.802566	0.471619
C	-3.045086	-1.424266	0.572085
C	-0.635151	-1.197006	1.371809
C	-3.861808	-2.375323	-0.306473
C	0.645007	-1.607648	1.666636
C	0.315321	0.316842	2.692334
H	-1.407500	-2.864556	0.337587
H	-3.398782	-1.605695	1.590865
H	-1.616895	0.603381	2.013058
H	1.176575	-2.491822	1.364247
H	0.474128	1.193160	3.295371
H	-4.565224	-0.856362	-1.484646
H	-5.117594	-2.455311	-1.886973
H	2.132584	-0.670657	2.863061
C	-4.251010	0.694861	0.812307
H	-2.641737	0.436886	-0.398553
O	-5.078299	0.233476	1.592178
H	-4.266360	1.739140	0.488210

SCF Energy: -1595.66913868
Sum of electronic and zero-point Energies= -1595.277254
Sum of electronic and thermal Energies= -1595.248972
Sum of electronic and thermal Enthalpies= -1595.248028
Sum of electronic and thermal Free Energies= -1595.338175

==> ./AOX-III/water/PA/Cys/C3 <==

O	-0.846872	-1.849485	0.733743
O	0.911322	-0.885889	-2.229084
O	-4.004273	0.551162	-1.984319
O	-1.559898	0.073772	-2.793511
O	-5.168203	-0.966637	2.323142
C	-2.367367	-0.627947	-0.687508

C	-2.087795	-1.282775	0.530669
C	0.122616	-1.760420	-0.178269
C	-1.379717	-0.494823	-1.692293
C	-0.065232	-1.066955	-1.367372
C	1.379126	-2.372327	0.230726
C	-3.684247	-0.087481	-0.832861
C	-2.989537	-1.416150	1.543553
C	-4.309075	-0.865039	1.394283
C	-4.614311	-0.200785	0.161857
C	1.755967	-2.312149	1.578581
C	2.199104	-3.021196	-0.701073
C	2.958429	-2.869282	1.980239
C	3.388555	-3.595960	-0.282951
C	3.774641	-3.511186	1.051257
H	-2.721128	-1.932960	2.453861
H	-5.601261	0.219825	0.025025
H	1.119580	-1.808135	2.292415
H	1.894397	-3.093557	-1.734258
H	3.261121	-2.805227	3.016008
H	4.015737	-4.109110	-0.998381
H	4.710150	-3.950204	1.370105
H	1.804829	-0.437495	-1.797351
H	-3.205744	0.537061	-2.559529
S	3.026279	0.614086	-1.072052
N	1.488922	3.985112	-1.681494
O	-0.109918	2.456004	-1.219251
O	0.556158	4.131359	3.391351
N	0.869087	2.978770	1.461707
C	1.704673	2.622681	0.324835
C	1.985002	1.123307	0.319833
C	0.956116	3.012965	-0.945268
C	1.248549	3.829939	2.417909
H	2.635302	3.180539	0.409278
H	1.024264	0.598189	0.261185
H	2.457370	0.841754	1.259821
H	-0.045471	2.549245	1.533539
H	2.248231	4.249796	2.269126
H	1.022923	4.280627	-2.526136
H	2.373669	4.398309	-1.436798

SCF Energy: -1768.34021189
Sum of electronic and zero-point Energies= -1768.013222
Sum of electronic and thermal Energies= -1767.986791
Sum of electronic and thermal Enthalpies= -1767.985847
Sum of electronic and thermal Free Energies= -1768.073644

==> ./AOX-III/water/PA/Cys/C5 <==

O	2.572038	0.975003	0.550095
O	2.765048	-1.708317	-1.828076
O	-1.529360	1.163981	-1.880034
O	0.349488	-0.851510	-2.290873
O	-0.738414	3.973480	1.815078
C	0.509092	0.943416	-0.686720
C	1.328734	1.446419	0.312497
C	3.084404	-0.049557	-0.167875
C	0.975829	-0.200904	-1.437051
C	2.325727	-0.640921	-1.128156
C	4.440398	-0.419683	0.245310
C	-0.807114	1.580490	-0.882641
C	0.921851	2.470712	1.166949
C	-0.401856	3.061772	1.020758
C	-1.225121	2.580387	-0.030647
C	4.830998	-0.203860	1.571374
C	5.351749	-0.960989	-0.666065
C	6.112646	-0.540126	1.980660
C	6.634948	-1.286762	-0.248825
C	7.017065	-1.081937	1.072509
H	1.580721	2.823712	1.946554
H	-2.190925	3.039761	-0.188341
H	4.129465	0.215040	2.278569
H	5.067859	-1.107215	-1.696667
H	6.405274	-0.379482	3.009059
H	7.338573	-1.696565	-0.960192
H	8.016966	-1.340833	1.393018
H	-2.735370	1.239943	-1.691618
S	-4.350005	1.205385	-1.474626
N	-2.470551	-1.817270	-1.515560
O	-1.754742	-1.062019	0.491712
O	-5.604400	-2.744588	2.965910
N	-4.281753	-1.683613	1.455590
C	-4.111166	-1.095801	0.134122
C	-4.404233	0.408025	0.168452
C	-2.671259	-1.326015	-0.293224
C	-5.390290	-2.324159	1.825746
H	-4.797401	-1.591071	-0.550012
H	-3.698665	0.888308	0.847438
H	-5.407279	0.532603	0.573244
H	-3.583851	-1.476058	2.159133
H	-6.114935	-2.465455	1.017577
H	-1.533832	-1.750711	-1.896054
H	-3.246091	-1.877614	-2.155799
H	2.023988	-1.965638	-2.405639

SCF Energy: -1768.33702991
Sum of electronic and zero-point Energies= -1768.009833
Sum of electronic and thermal Energies= -1767.983090
Sum of electronic and thermal Enthalpies= -1767.982146
Sum of electronic and thermal Free Energies= -1768.071712

==> ./AOX-III/water/PA/2dG-C4p/C5 <==

O	-4.375483	0.246762	0.219870
O	-2.409900	-2.066291	-1.740355
O	-0.595918	2.820549	-1.276523
O	-0.935175	0.073717	-1.908755
O	-3.873648	4.720941	1.505640
C	-2.421581	1.412309	-0.577470
C	-3.620025	1.378566	0.157656
C	-4.000706	-0.903006	-0.410484
C	-1.983413	0.226748	-1.249023
C	-2.855270	-0.938623	-1.121700
C	-4.940082	-2.007016	-0.177119
C	-1.721209	2.666491	-0.575048
C	-4.126109	2.460199	0.856263
C	-3.439038	3.697342	0.869194
C	-2.209502	3.746472	0.133494
C	-5.682121	-2.034147	1.008654
C	-5.118291	-3.021932	-1.122110
C	-6.575665	-3.068122	1.249211
C	-6.018828	-4.050023	-0.876028
C	-6.746210	-4.079298	0.308885
H	-5.056705	2.351062	1.396194
H	-1.642619	4.668492	0.121830
H	-5.551913	-1.251629	1.742371
H	-4.569563	-2.999302	-2.050926
H	-7.139036	-3.084049	2.172135
H	-6.155321	-4.826774	-1.615949
H	-7.444558	-4.883349	0.497077
H	-1.555966	-1.814597	-2.134759
H	0.407953	2.338205	-0.624108
O	2.031893	0.931739	-0.741390
O	2.098429	4.033548	-0.523170
O	1.587120	0.732503	2.197186
O	6.523612	-3.666511	-0.166833
N	4.225196	0.199098	-1.007751
N	5.752260	-1.202374	-1.847875
N	3.725652	-0.914477	1.100677
N	5.015252	-2.872960	1.343591
N	3.534538	-2.218292	2.991533
C	3.382349	1.370523	-0.948561
C	2.306160	2.975909	0.414678
C	3.666237	2.319034	0.206956
C	1.343611	1.844887	0.069972
C	0.644042	1.144929	1.210901
C	4.380758	-0.790967	-0.073154
C	5.071295	-0.112530	-2.048892
C	5.328992	-1.644746	-0.609611
C	5.705486	-2.795560	0.133401
C	4.078306	-1.981630	1.787688
H	3.464037	1.860931	-1.916000
H	2.153005	3.331315	1.431641
H	3.947193	1.752483	1.092918
H	4.443035	3.040653	-0.034887
H	0.090304	0.289612	0.820711
H	-0.047871	1.833696	1.692222
H	5.127827	0.522067	-2.918151
H	2.767312	4.706687	-0.358824
H	2.253444	0.159511	1.772837
H	5.240309	-3.671050	1.926605
H	3.651628	-3.116937	3.431210
H	2.744647	-1.654506	3.262739

SCF Energy: -1916.59756604
Sum of electronic and zero-point Energies= -1916.148898
Sum of electronic and thermal Energies= -1916.116037
Sum of electronic and thermal Enthalpies= -1916.115093
Sum of electronic and thermal Free Energies= -1916.217218

==> ./AOX-III/water/PA/2dG-C4p/C3 <==

O	4.112524	0.085783	0.383029
O	1.482471	-1.115336	-1.753074
O	2.272073	3.975629	-1.747421
O	1.336972	1.571780	-2.193105
O	5.863014	4.348334	1.295780
C	3.146190	1.983402	-0.734212
C	4.087695	1.441309	0.161896
C	3.254125	-0.750677	-0.238928
C	2.224349	1.141865	-1.415301
C	2.336476	-0.294005	-1.149217
C	3.381961	-2.135452	0.226517
C	3.167170	3.398388	-0.901267
C	5.002876	2.195266	0.846798
C	5.025002	3.612152	0.670533
C	4.068262	4.180970	-0.231713
C	3.731438	-2.364694	1.562576
C	3.159805	-3.221933	-0.625806
C	3.836829	-3.662133	2.041645
C	3.278723	-4.516880	-0.140283
C	3.609576	-4.740646	1.192266
H	5.701621	1.723469	1.523170
H	4.061493	5.252603	-0.380091
H	3.903655	-1.527520	2.224044
H	2.915574	-3.053573	-1.662819
H	4.094992	-3.831029	3.077999
H	3.116012	-5.352783	-0.806675
H	3.695165	-5.751773	1.566411
H	0.458068	-1.223966	-1.081765
H	1.713387	3.251115	-2.110603
O	-1.578380	-0.454786	-0.933106
O	-0.557787	-3.378209	-1.333522
O	-1.117757	-0.634948	1.976809

O	-7.322626	2.154097	0.339519
N	-3.894355	-0.481717	-1.155838
N	-5.826333	0.467404	-1.762676
N	-3.735313	0.287453	1.148235
N	-5.594862	1.618189	1.723278
N	-3.938475	1.163232	3.269088
C	-2.698270	-1.279589	-1.290920
C	-1.098541	-2.659282	-0.222338
C	-2.607355	-2.480458	-0.360095
C	-0.596490	-1.225154	-0.299067
C	-0.114211	-0.562159	0.965366
C	-4.345365	0.192013	-0.051774
C	-4.826115	-0.269604	-2.148238
C	-5.539657	0.769739	-0.445311
C	-6.254661	1.559895	0.494700
C	-4.404240	1.014432	2.019507
H	-2.625250	-1.561136	-2.339094
H	-0.807032	-3.130979	0.714292
H	-3.042201	-2.233776	0.606991
H	-3.094330	-3.360062	-0.774442
H	0.150105	0.476116	0.752115
H	0.762145	-1.086671	1.341808
H	-4.691942	-0.699876	-3.127167
H	-0.930326	-4.266550	-1.318366
H	-1.951728	-0.268344	1.624825
H	-6.051952	2.166529	2.442812
H	-4.331404	1.865511	3.875018
H	-2.998158	0.851403	3.453637

SCF Energy: -1916.61092616
Sum of electronic and zero-point Energies= -1916.162468
Sum of electronic and thermal Energies= -1916.129766
Sum of electronic and thermal Enthalpies= -1916.128821
Sum of electronic and thermal Free Energies= -1916.231583

==> ./AOX-III/water/Met/C3 <==

O	2.625400	0.808320	0.225224
O	-0.856502	0.742448	1.140044
O	1.518620	-3.817573	0.796726
O	-0.234304	-1.913763	1.165042
O	5.881433	-2.464275	-0.505348
C	1.998246	-1.481565	0.538369
C	2.950828	-0.512306	0.206182
C	1.377891	1.226156	0.546737
C	0.659458	-1.087469	0.892017
C	0.378988	0.346084	0.888906
C	1.230980	2.680504	0.432372
C	2.398688	-2.839636	0.495567
C	4.250085	-0.825889	-0.144816
C	4.601293	-2.169564	-0.164828
C	3.689440	-3.181189	0.149151
C	1.948744	3.358595	-0.558861
C	0.398766	3.397092	1.297672
C	1.820207	4.733496	-0.692087
C	0.285454	4.773920	1.164783
C	0.988403	5.443498	0.168179
H	4.962895	-0.053333	-0.390953
H	3.988178	-4.220488	0.122452
H	2.591557	2.806369	-1.229937
H	-0.139553	2.881729	2.077812
H	2.368264	5.250324	-1.467776
H	-0.351173	5.324793	1.843354
H	0.891306	6.515674	0.064532
H	-1.386438	0.995609	0.029876
H	0.660226	-3.384281	1.007282
H	6.027883	-3.417531	-0.475139
S	-0.439326	0.734435	-2.185689
O	-3.923169	1.224431	1.195450
N	-4.599426	-1.321924	-0.805937
N	-3.693443	-0.877198	1.995554
C	-3.152512	0.548431	-1.403933
C	-3.437251	-0.548800	-0.384867
C	-1.800726	1.222578	-1.204686
C	-3.696023	0.027004	1.014557
C	-0.654238	-1.051183	-2.385569
H	-3.930361	1.306624	-1.326593
H	-3.220588	0.110215	-2.399611
H	-2.598574	-1.239543	-0.311994
H	-1.836996	2.309652	-1.212640
H	-3.892351	-0.587903	2.940723
H	-3.498254	-1.847049	1.794880
H	-0.636293	-1.552494	-1.421572
H	0.188227	-1.387418	-2.985330
H	-1.580970	-1.269700	-2.910985
C	-4.746365	-2.619600	-0.530050
H	-5.321506	-0.860317	-1.340355
O	-3.948498	-3.281966	0.137976
H	-5.652155	-3.059958	-0.954024

SCF Energy: -1847.42749939
Sum of electronic and zero-point Energies= -1847.029760
Sum of electronic and thermal Energies= -1847.000588
Sum of electronic and thermal Enthalpies= -1846.999644
Sum of electronic and thermal Free Energies= -1847.092980

==> ./AOX-III/water/Met/C7 <==

O	-2.510783	-0.286545	-0.796556
O	-4.623699	1.639559	1.417598

O	-0.372184	3.975327	-0.286531
O	-2.664222	3.378344	0.907552
O	1.634946	0.535916	-2.825833
C	-1.496554	1.859834	-0.495146
C	-1.495441	0.576507	-1.047553
C	-3.555392	0.050977	0.007489
C	-2.579798	2.259309	0.353993
C	-3.615899	1.277375	0.580590
C	-4.514625	-1.046551	0.174746
C	-0.404058	2.721840	-0.798837
C	-0.473353	0.119277	-1.866256
C	0.606087	0.977228	-2.128748
C	0.626399	2.287280	-1.592036
C	-4.053796	-2.366613	0.132469
C	-5.877304	-0.795150	0.356707
C	-4.944723	-3.418720	0.286414
C	-6.762814	-1.854550	0.502555
C	-6.300248	-3.165666	0.472471
H	-0.516284	-0.867719	-2.301127
H	1.466821	2.934851	-1.799103
H	-3.000925	-2.564978	-0.010193
H	-6.245504	0.219125	0.364908
H	-4.580342	-4.436434	0.261513
H	-7.817066	-1.653651	0.634401
H	-6.993646	-3.987275	0.589807
H	-4.418471	2.538656	1.720956
H	-1.170952	4.096527	0.267378
H	2.272443	-0.282378	-1.966306
S	1.512687	-1.536785	-0.062565
O	6.457423	-1.575196	-0.752218
N	5.703254	0.317326	1.128236
N	5.107023	-3.025928	0.336781
C	3.846934	0.001706	-0.450099
C	4.742098	-0.645877	0.625348
C	2.824796	-0.941188	-1.047217
C	5.518916	-1.801429	0.015248
C	1.190549	-0.220897	1.136253
H	4.490498	0.354232	-1.257311
H	3.362985	0.871230	-0.005694
H	4.139912	-0.996043	1.461235
H	3.245914	-1.787690	-1.588916
H	5.558584	-3.826555	-0.079869
H	4.355522	-3.169805	0.991676
H	1.979136	-0.193358	1.884359
H	0.244678	-0.475107	1.609154
H	1.106488	0.738193	0.632876
C	5.391889	1.200482	2.079806
H	6.592605	0.397806	0.655822
O	4.304353	1.244592	2.657554
H	6.204017	1.892129	2.318639

SCF Energy: -1847.42374399

Sum of electronic and zero-point Energies= -1847.025904
Sum of electronic and thermal Energies= -1846.996698
Sum of electronic and thermal Enthalpies= -1846.995754
Sum of electronic and thermal Free Energies= -1847.089573

==> ./AOX-III/water/Met/C5 <==

O	-2.843019	0.954206	0.300895
O	-2.528543	-1.910304	-1.870336
O	1.488853	1.452754	-1.718179
O	-0.264719	-0.691597	-2.340565
O	-0.244474	4.695313	1.261583
C	-0.705610	1.101543	-0.804027
C	-1.675620	1.606273	0.070507
C	-3.138299	-0.222260	-0.316297
C	-0.985893	-0.131897	-1.499291
C	-2.262995	-0.763472	-1.190696
C	-4.461765	-0.738322	0.052236
C	0.515505	1.849486	-0.943291
C	-1.531320	2.800469	0.766094
C	-0.362134	3.520473	0.585083
C	0.656335	3.051381	-0.239530
C	-5.470748	0.162621	0.408778
C	-4.726013	-2.110810	0.067667
C	-6.729278	-0.304617	0.759010
C	-5.986611	-2.570016	0.425428
C	-6.991090	-1.671061	0.767279
H	-2.315274	3.150174	1.421363
H	1.573282	3.615307	-0.351624
H	-5.270850	1.224590	0.401148
H	-3.947212	-2.815215	-0.180629
H	-7.506194	0.398919	1.024863
H	-6.181381	-3.633540	0.442248
H	-7.972215	-2.033239	1.042680
H	-1.765271	-2.045009	-2.458255
H	2.232403	0.248625	-1.348044
H	0.596567	5.118128	1.050762
S	4.262734	-0.518831	-2.323951
O	1.182707	0.172357	1.734274
N	3.138901	-1.789287	2.290231
N	2.988345	1.511931	1.490348
C	2.784044	-1.590504	-0.111675
C	3.252979	-0.875052	1.162947
C	2.789183	-0.817947	-1.424281
C	2.379723	0.328383	1.481072
C	5.535906	-0.156089	-1.099838
H	1.752502	-1.904035	0.058850
H	3.388334	-2.493564	-0.220781
H	4.291498	-0.573211	1.098568
H	2.123375	-1.300791	-2.138150

H	2.446442	2.345101	1.664652
H	3.967454	1.597934	1.270862
H	5.243452	0.699461	-0.495082
H	6.430306	0.091801	-1.666570
H	5.728319	-1.030988	-0.482591
C	4.113307	-2.645801	2.605515
H	2.240391	-1.885636	2.741825
O	5.208205	-2.692292	2.041414
H	3.859878	-3.312204	3.434249

SCF Energy: -1847.40515090
Sum of electronic and zero-point Energies= -1847.006648
Sum of electronic and thermal Energies= -1846.977395
Sum of electronic and thermal Enthalpies= -1846.976451
Sum of electronic and thermal Free Energies= -1847.069614

==> ./AOX-III/water/_HAT/C5 <==

O	-0.164852	-0.815829	0.094175
O	-1.398819	2.555842	-0.156283
O	3.785415	1.809548	-0.132466
O	1.193047	2.975964	-0.178295
O	3.987578	-2.899596	0.197836
C	1.722709	0.640552	-0.020523
C	1.159868	-0.615530	0.067052
C	-1.042615	0.222261	0.029231
C	0.852020	1.789106	-0.085400
C	-0.574358	1.490793	-0.056086
C	-2.441202	-0.215749	0.021455
C	3.187390	0.726244	-0.047624
C	1.920807	-1.804075	0.140077
C	3.315946	-1.724998	0.121301
C	3.941973	-0.505602	0.028162
C	-2.764196	-1.448997	-0.554159
C	-3.448498	0.570484	0.587148
C	-4.082199	-1.880558	-0.575667
C	-4.763977	0.126944	0.567014
C	-5.084615	-1.094095	-0.016391
H	1.414866	-2.755619	0.208879
H	5.020822	-0.429105	0.008421
H	-1.987511	-2.059214	-0.992959
H	-3.204027	1.511630	1.055307
H	-4.326598	-2.830287	-1.031160
H	-5.538449	0.735430	1.013161
H	-6.111343	-1.433305	-0.033101
H	-0.820402	3.334414	-0.237594
H	4.939369	-2.742051	0.175774

SCF Energy: -953.282992437
Sum of electronic and zero-point Energies= -953.073300
Sum of electronic and thermal Energies= -953.057597
Sum of electronic and thermal Enthalpies= -953.056653
Sum of electronic and thermal Free Energies= -953.117523

==> ./AOX-III/water/_HAT/C7 <==

O	-0.205710	-0.899330	0.090639
O	-1.292639	2.518967	-0.187887
O	3.767510	1.772077	-0.100440
O	1.359575	2.821791	-0.178159
O	3.993673	-2.912962	0.188058
C	1.741975	0.482528	-0.011948
C	1.138345	-0.765228	0.069772
C	-1.024639	0.172022	0.020281
C	0.917261	1.656059	-0.087226
C	-0.504117	1.431276	-0.074277
C	-2.442393	-0.190303	0.017672
C	3.191315	0.559316	-0.023671
C	1.881251	-1.934493	0.138036
C	3.332831	-1.856264	0.125920
C	3.952882	-0.561283	0.042036
C	-2.827959	-1.410537	-0.548425
C	-3.405274	0.649929	0.584808
C	-4.165407	-1.775194	-0.560741
C	-4.740080	0.270270	0.576982
C	-5.123240	-0.936455	0.001307
H	1.399206	-2.898733	0.198931
H	5.031353	-0.497009	0.033199
H	-2.084269	-2.059976	-0.987844
H	-3.112005	1.579954	1.046737
H	-4.460306	-2.713339	-1.009992
H	-5.480861	0.917496	1.025532
H	-6.165585	-1.224462	-0.007097
H	-0.704397	3.288101	-0.271117
H	3.061099	2.453750	-0.149184

SCF Energy: -953.281849790
Sum of electronic and zero-point Energies= -953.072265
Sum of electronic and thermal Energies= -953.056816
Sum of electronic and thermal Enthalpies= -953.055872
Sum of electronic and thermal Free Energies= -953.116042

==> ./AOX-III/water/_HAT/C3 <==

O	-0.245838	-0.825636	0.068647
O	-1.327686	2.540103	-0.232727

O	3.763799	1.816920	-0.064094
O	1.356770	2.900105	-0.117228
O	3.922479	-2.914389	0.144838
C	1.713979	0.574449	-0.007983
C	1.117246	-0.691958	0.049334
C	-1.081345	0.216097	0.007416
C	0.902693	1.752872	-0.067866
C	-0.586984	1.552768	-0.095685
C	-2.480426	-0.163479	0.012427
C	3.129954	0.632847	-0.011091
C	1.838396	-1.859339	0.101040
C	3.230795	-1.755514	0.093980
C	3.882886	-0.525331	0.038433
C	-2.834023	-1.449825	-0.427816
C	-3.479026	0.710875	0.468203
C	-4.161638	-1.840423	-0.433687
C	-4.803297	0.301829	0.470473
C	-5.149038	-0.966552	0.014769
H	1.347353	-2.819647	0.146442
H	4.963165	-0.471584	0.032923
H	-2.069553	-2.125828	-0.781762
H	-3.215973	1.687578	0.839948
H	-4.428566	-2.826098	-0.788434
H	-5.567557	0.974212	0.834358
H	-6.185181	-1.276003	0.012920
H	3.087236	2.525907	-0.098348
H	4.871835	-2.741275	0.134801

SCF Energy: -953.300220124
Sum of electronic and zero-point Energies= -953.089409
Sum of electronic and thermal Energies= -953.074098
Sum of electronic and thermal Enthalpies= -953.073153
Sum of electronic and thermal Free Energies= -953.133006

==> ./AOX-III/water/Tyr/C5 <==

O	1.162429	1.030946	1.299455
O	3.070354	2.354985	-1.463441
O	3.727154	-2.691262	-0.281019
O	4.008450	-0.086645	-1.346992
O	0.930842	-3.048127	3.525470
C	2.585331	-0.728784	0.477732
C	1.613761	-0.244071	1.357716
C	1.627839	1.907883	0.368237
C	3.142820	0.178693	-0.500525
C	2.595173	1.528988	-0.495564
C	0.938562	3.201734	0.419023
C	2.954221	-2.122480	0.591588
C	1.046547	-1.020702	2.361701
C	1.491361	-2.332730	2.516458
C	2.422520	-2.881542	1.654040
C	-0.393915	3.244126	0.843379
C	1.587538	4.386821	0.061452
C	-1.069831	4.454718	0.893010
C	0.905377	5.594723	0.121013
C	-0.422964	5.632240	0.531094
H	0.300220	-0.600269	3.019946
H	2.716103	-3.919098	1.744664
H	-0.898561	2.330841	1.124718
H	2.622759	4.368017	-0.242382
H	-2.101909	4.478693	1.214803
H	1.416214	6.508877	-0.148543
H	-0.950774	6.575210	0.573288
H	3.701017	1.820028	-1.975959
H	3.046346	-2.752187	-1.415052
O	-3.219753	-1.605358	-2.449100
O	2.331732	-2.618535	-2.252379
N	-4.549570	-0.300226	0.637455
N	-4.645940	0.111327	-2.097399
C	-2.172980	-0.299234	0.142273
C	-3.538224	-0.936976	-0.184459
C	-1.022030	-0.956268	-0.556633
C	-0.604960	-2.241960	-0.170534
C	-0.350623	-0.294127	-1.605658
C	-3.806113	-0.836020	-1.682902
C	0.480026	-2.834752	-0.770414
C	0.738718	-0.865738	-2.210516
C	1.207310	-2.129586	-1.764375
H	-2.213759	0.759918	-0.114447
H	-2.031604	-0.389966	1.220639
H	-3.513287	-2.000147	0.050403
H	-1.145232	-2.760467	0.611351
H	-0.693035	0.685957	-1.912369
H	0.812704	-3.825243	-0.493291
H	1.292783	-0.356487	-2.987427
H	-4.825553	0.209799	-3.085007
H	-5.139102	0.701307	-1.447626
C	-5.690651	-0.909854	0.982561
H	-4.445341	0.680108	0.870685
O	-6.599533	-0.368651	1.609151
H	-5.748923	-1.956344	0.665595
H	1.285213	-3.945701	3.538285

SCF Energy: -1676.93679424
Sum of electronic and zero-point Energies= -1676.512148
Sum of electronic and thermal Energies= -1676.482348
Sum of electronic and thermal Enthalpies= -1676.481404
Sum of electronic and thermal Free Energies= -1676.574494

==> ./AOX-III/water/Tyr/C3 <==

O	1.858982	0.008461	1.555588
O	3.589914	0.896492	-1.456998
O	-0.209016	4.033698	-0.018225
O	2.003077	3.088413	-1.079510
O	-2.048442	1.566757	3.592484
C	0.901235	2.047344	0.732136
C	0.884980	0.964599	1.617451
C	2.754697	-0.025062	0.556762
C	1.904599	2.130396	-0.292424
C	2.797357	0.968378	-0.414834
C	3.613863	-1.203020	0.603590
C	-0.151223	2.989923	0.829893
C	-0.074233	0.803527	2.594147
C	-1.090556	1.752918	2.652712
C	-1.141575	2.841598	1.781437
C	3.093507	-2.395641	1.122803
C	4.938127	-1.156533	0.154120
C	3.885852	-3.531375	1.172346
C	5.726886	-2.295391	0.222138
C	5.202772	-3.482845	0.723370
H	-0.054665	-0.043175	3.263868
H	-1.947988	3.560101	1.841527
H	2.068662	-2.431300	1.464861
H	5.349351	-0.232970	-0.222430
H	3.477505	-4.454219	1.560030
H	6.753591	-2.254771	-0.113874
H	5.820717	-4.369310	0.766966
H	2.895019	0.613549	-2.378349
H	0.568707	3.975877	-0.616373
O	-3.362370	-1.294530	-2.707728
O	1.981693	0.340431	-3.126267
N	-4.610295	-1.414916	0.660770
N	-5.237132	-0.380259	-1.838637
C	-2.568334	-0.215441	0.126692
C	-3.553940	-1.309144	-0.329736
C	-1.370400	-0.087149	-0.765823
C	-1.265898	0.985797	-1.674450
C	-0.345646	-1.035147	-0.704564
C	-4.061000	-0.998697	-1.734658
C	-0.162028	1.128161	-2.478159
C	0.770418	-0.913158	-1.508828
C	0.894765	0.187101	-2.401923
H	-3.100975	0.735423	0.181081
H	-2.241811	-0.486900	1.131931
H	-3.033188	-2.263182	-0.396483
H	-2.069058	1.710776	-1.721017
H	-0.426370	-1.864456	-0.012510
H	-0.062202	1.960249	-3.161673
H	1.569075	-1.643018	-1.482122
H	-5.590421	-0.146092	-2.753727
H	-5.803052	-0.185724	-1.028859
C	-5.357923	-2.515946	0.804882
H	-4.872317	-0.590627	1.187892
O	-6.310320	-2.607863	1.577111
H	-5.040829	-3.353711	0.174598
H	-2.713720	2.263679	3.533616

SCF Energy: -1676.94900116

Sum of electronic and zero-point Energies= -1676.524177

Sum of electronic and thermal Energies= -1676.493784

Sum of electronic and thermal Enthalpies= -1676.492840

Sum of electronic and thermal Free Energies= -1676.588318

==> ./AOX-III/water/Tyr/C7 <==

O	-0.858122	-1.668979	0.719504
O	1.062740	-2.446914	-2.236851
O	-3.645162	-0.559287	-3.012206
O	-1.224137	-1.582620	-3.307513
O	-5.008094	0.368216	1.400860
C	-2.194003	-1.131909	-1.187435
C	-2.015909	-1.208162	0.193959
C	0.184715	-2.038916	-0.065968
C	-1.149595	-1.578401	-2.057648
C	0.062884	-2.036355	-1.418492
C	1.375287	-2.397685	0.710672
C	-3.437620	-0.615494	-1.679605
C	-2.980606	-0.776996	1.089682
C	-4.176869	-0.185929	0.582319
C	-4.396800	-0.153949	-0.822443
C	1.222273	-2.959216	1.982385
C	2.656729	-2.132582	0.218456
C	2.340740	-3.268647	2.742182
C	3.770235	-2.434867	0.991105
C	3.615780	-3.007056	2.249327
H	-2.836319	-0.892958	2.153675
H	-5.309801	0.278843	-1.205963
H	0.232236	-3.160218	2.366593
H	2.782607	-1.677025	-0.752462
H	2.216952	-3.712567	3.720314
H	4.758518	-2.216347	0.609661
H	4.485724	-3.245237	2.846039
H	0.730895	-2.354916	-3.145191
H	-2.848922	-0.914097	-3.462738
H	-4.231623	1.301263	2.134346
O	1.344369	4.113096	0.176406
O	-3.431312	1.830182	2.594837
N	3.498308	1.485864	-1.034345
N	3.437144	4.181697	-0.666791
C	1.071470	1.423237	-0.746623
C	2.400773	2.011198	-0.247754
C	-0.103014	1.612059	0.163436

C	-1.323758	2.081313	-0.352815
C	-0.024099	1.262145	1.528460
C	2.352416	3.537425	-0.240101
C	-2.437691	2.180702	0.445977
C	-1.124972	1.347841	2.339798
C	-2.366466	1.779116	1.804729
H	0.846819	1.824202	-1.735500
H	1.240136	0.347995	-0.855633
H	2.574817	1.716873	0.788391
H	-1.378196	2.369398	-1.394330
H	0.908203	0.893150	1.935345
H	-3.379368	2.548582	0.062134
H	-1.088358	1.057784	3.380713
H	3.452783	5.189292	-0.631951
H	4.251651	3.683295	-0.985128
C	4.509488	0.781221	-0.510857
H	3.440350	1.547475	-2.043921
O	5.400560	0.257239	-1.176830
H	4.487666	0.714245	0.581442

SCF Energy: -1676.94516250

Sum of electronic and zero-point Energies= -1676.519597

Sum of electronic and thermal Energies= -1676.489360

Sum of electronic and thermal Enthalpies= -1676.488416

Sum of electronic and thermal Free Energies= -1676.582479

==> ./AOX-III/water/2dG-C4p/C5 <==

O	-4.358610	0.202731	0.227160
O	-2.413395	-2.108366	-1.747625
O	-0.616796	2.784477	-1.309739
O	-0.935414	0.049365	-1.919468
O	-3.899131	4.640518	1.561536
C	-2.423771	1.379379	-0.583039
C	-3.607093	1.326421	0.157059
C	-3.988931	-0.942990	-0.408283
C	-1.978516	0.182121	-1.263659
C	-2.846198	-0.978865	-1.130054
C	-4.925765	-2.046956	-0.170923
C	-1.721260	2.625612	-0.598023
C	-4.106877	2.412344	0.871599
C	-3.396781	3.598709	0.850331
C	-2.206951	3.709214	0.127385
C	-5.650983	-2.080140	1.024958
C	-5.117236	-3.053301	-1.122147
C	-6.542920	-3.114123	1.269351
C	-6.017214	-4.080599	-0.871976
C	-6.727937	-4.116938	0.322741
H	-5.026832	2.317543	1.429718
H	-1.652232	4.638218	0.115421
H	-5.508621	-1.303602	1.762781
H	-4.580692	-3.024537	-2.057809
H	-7.093606	-3.136850	2.199640
H	-6.165712	-4.851382	-1.615668
H	-7.425314	-4.921043	0.514154
H	-1.560341	-1.875000	-2.152571
H	0.424684	2.309738	-0.661153
O	2.031795	0.898307	-0.759464
O	2.103168	4.003059	-0.676194
O	1.638061	0.814611	2.187048
O	6.510276	-3.692003	-0.093018
N	4.218104	0.152057	-1.041046
N	5.713183	-1.296321	-1.858372
N	3.769174	-0.862730	1.127744
N	5.049823	-2.820093	1.421228
N	3.621819	-2.081178	3.079207
C	3.379121	1.326296	-1.013466
C	2.331822	2.988361	0.303089
C	3.686486	2.320800	0.096249
C	1.361553	1.844486	0.024686
C	0.678382	1.192696	1.204885
C	4.393286	-0.796434	-0.067426
C	5.033309	-0.212058	-2.089981
C	5.321397	-1.679787	-0.590467
C	5.708320	-2.801086	0.191238
C	4.132812	-1.901830	1.851363
H	3.439247	1.775869	-2.002029
H	2.198958	3.387373	1.306634
H	3.983535	1.790636	0.999373
H	4.458860	3.030603	-0.190454
H	0.112753	0.326733	0.856738
H	-0.000923	1.903623	1.671639
H	5.070345	0.382784	-2.987838
H	2.780296	4.678855	-0.564551
H	2.298747	0.227494	1.772683
H	5.283144	-3.595465	2.031113
H	3.745823	-2.961462	3.552959
H	2.843470	-1.500212	3.347455
H	-3.318274	5.408350	1.495606

SCF Energy: -1917.05867941

Sum of electronic and zero-point Energies= -1916.596999

Sum of electronic and thermal Energies= -1916.563909

Sum of electronic and thermal Enthalpies= -1916.562965

Sum of electronic and thermal Free Energies= -1916.664882

==> ./AOX-III/water/2dG-C4p/C3 <==

O	-4.085481	-0.045157	0.362673
O	-1.444186	1.164312	-1.752887

O	-2.269445	-3.928936	-1.780522
O	-1.310061	-1.524588	-2.199530
O	-5.885311	-4.260577	1.278077
C	-3.131909	-1.938775	-0.758098
C	-4.061187	-1.389109	0.130786
C	-3.224124	0.795733	-0.248131
C	-2.189726	-1.085922	-1.435014
C	-2.299961	0.343738	-1.158917
C	-3.356714	2.175558	0.224614
C	-3.149181	-3.342072	-0.943088
C	-4.987903	-2.149537	0.814728
C	-4.973169	-3.522673	0.598114
C	-4.064940	-4.127748	-0.273174
C	-3.735122	2.394818	1.554787
C	-3.111145	3.267118	-0.615213
C	-3.845936	3.688626	2.041025
C	-3.236889	4.558235	-0.122185
C	-3.596240	4.772326	1.204514
H	-5.691806	-1.693958	1.494831
H	-4.072971	-5.199157	-0.421608
H	-3.924464	1.553465	2.206047
H	-2.844855	3.106139	-1.647800
H	-4.125915	3.850924	3.072668
H	-3.056818	5.398816	-0.777990
H	-3.686396	5.780870	1.584441
H	-0.404810	1.266519	-1.061769
H	-1.694857	-3.220381	-2.147340
O	1.605751	0.472826	-0.922588
O	0.615200	3.409749	-1.306475
O	1.144639	0.643608	1.985264
O	7.315026	-2.212458	0.334139
N	3.921360	0.476647	-1.144672
N	5.844573	-0.487655	-1.755039
N	3.746923	-0.312973	1.151563
N	5.588651	-1.672044	1.717872
N	3.932127	-1.215348	3.262745
C	2.734275	1.288066	-1.275990
C	1.148684	2.678915	-0.200216
C	2.655345	2.484416	-0.338530
C	0.630534	1.249935	-0.287230
C	0.140779	0.585421	0.974595
C	4.361750	-0.212311	-0.045637
C	4.853686	0.263850	-2.136463
C	5.550694	-0.799410	-0.441474
C	6.253510	-1.607734	0.492290
C	4.404386	-1.057410	2.017008
H	2.664235	1.575870	-2.322664
H	0.861895	3.146974	0.739623
H	3.087215	2.227696	0.627290
H	3.151472	3.361365	-0.747590
H	-0.134163	-0.448955	0.755607
H	-0.730599	1.117475	1.352268
H	4.727136	0.705079	-3.111519
H	0.997136	4.293956	-1.286571
H	1.974745	0.268679	1.632401
H	6.036683	-2.233907	2.432641
H	4.318592	-1.923943	3.865359
H	2.995942	-0.892567	3.449197
H	-5.788887	-5.195916	1.061376

SCF Energy: -1917.07178438
Sum of electronic and zero-point Energies= -1916.610065
Sum of electronic and thermal Energies= -1916.577152
Sum of electronic and thermal Enthalpies= -1916.576208
Sum of electronic and thermal Free Energies= -1916.678357

==> ./AOX-III/water/2dG-C4p/C7 <==

O	-2.087073	-1.316699	-1.114732
O	-3.912860	-0.800330	1.962271
O	0.487296	-3.439580	2.335534
O	-1.801255	-2.195730	2.823191
O	2.174484	-3.030271	-2.048700
C	-0.850098	-2.355574	0.654033
C	-0.987604	-1.989217	-0.689539
C	-3.064712	-0.927594	-0.255154
C	-1.858416	-1.956398	1.598106
C	-2.978062	-1.215561	1.068077
C	-4.129422	-0.169990	-0.921156
C	0.317022	-3.063929	1.046640
C	-0.011320	-2.255453	-1.632709
C	1.155994	-2.898028	-1.205300
C	1.297796	-3.343552	0.126172
C	-3.814870	0.599504	-2.046358
C	-5.449392	-0.220834	-0.464279
C	-4.807321	1.319465	-2.694783
C	-6.437530	0.496954	-1.124457
C	-6.119937	1.270461	-2.235655
H	-0.112120	-1.907856	-2.650503
H	2.207745	-3.841921	0.429766
H	-2.795757	0.639837	-2.403307
H	-5.705695	-0.830860	0.388101
H	-4.555840	1.919049	-3.558753
H	-7.458411	0.446724	-0.771847
H	-6.892765	1.830123	-2.744686
H	-3.608793	-1.091870	2.836710
H	-0.277867	-3.111489	2.852021
H	2.896334	-1.936892	-1.886419
O	2.153719	-0.429619	-0.641909
O	4.738099	-2.169773	-0.330231
O	3.520027	1.437776	-2.405257
O	-0.821045	4.258997	2.362008
N	1.791807	0.638104	1.369618

N 0.196141 1.373754 2.752054
N 2.059770 2.581326 -0.070633
N 0.646096 4.337085 0.621803
N 2.055099 4.597575 -1.189726
C 2.632228 -0.325969 0.711573
C 4.478724 -0.808850 -0.675072
C 4.103980 0.018992 0.554853
C 3.193908 -0.766719 -1.498497
C 3.152882 0.099658 -2.734286
C 1.516152 1.920713 0.973719
C 0.946299 0.363430 2.423763
C 0.540924 2.363137 1.850003
C 0.037348 3.681866 1.692827
C 1.589584 3.803837 -0.212410
H 2.489357 -1.272542 1.227001
H 5.315751 -0.389775 -1.229706
H 4.219151 1.079705 0.339506
H 4.688181 -0.252711 1.430703
H 2.149452 0.061871 -3.161482
H 3.871462 -0.266296 -3.463337
H 0.960915 -0.602709 2.902409
H 5.530242 -2.190612 0.217920
H 2.959794 1.755791 -1.673781
H 0.334149 5.287230 0.457336
H 1.567131 5.446856 -1.424861
H 2.639113 4.173171 -1.892679

SCF Energy: -1917.06699938
Sum of electronic and zero-point Energies= -1916.605212
Sum of electronic and thermal Energies= -1916.572392
Sum of electronic and thermal Enthalpies= -1916.571448
Sum of electronic and thermal Free Energies= -1916.672920

==> ./AOX-III/water/vIP <==
O -0.214984 -0.882345 0.078474
O -1.377530 2.536020 -0.180165
O 3.717630 1.846734 -0.106688
O 1.275281 2.870147 -0.182346
O 4.008800 -2.882269 0.199249
C 1.704527 0.542636 -0.018467
C 1.132629 -0.732770 0.061681
C -1.055395 0.187883 0.013092
C 0.856470 1.695258 -0.092133
C -0.567812 1.447654 -0.074138
C -2.468374 -0.207550 0.014956
C 3.117537 0.639254 -0.028370
C 1.892034 -1.886906 0.133633
C 3.272080 -1.745567 0.124060
C 3.895628 -0.495470 0.042202
C -2.838958 -1.414217 -0.586952
C -3.440704 0.591243 0.622212
C -4.169693 -1.806172 -0.594295
C -4.769091 0.187179 0.617418
C -5.137222 -1.006969 0.006867
H 1.425029 -2.858181 0.197310
H 4.974452 -0.416877 0.033528
H -2.088293 -2.034610 -1.055791
H -3.158408 1.510925 1.111740
H -4.451157 -2.735419 -1.070250
H -5.516115 0.804706 1.096825
H -6.173638 -1.315813 0.002474
H -0.792836 3.305492 -0.270850
H 3.015857 2.529887 -0.156877
H 4.949597 -2.669329 0.193101

SCF Energy: -953.708822122

==> ./AOX-III/water/aIP <==
O -0.238268 -0.850936 0.080262
O -1.327422 2.507436 -0.171133
O 3.760415 1.814540 -0.073364
O 1.283557 2.890745 -0.106651
O 3.941086 -2.908543 0.118813
C 1.720994 0.559752 -0.002048
C 1.131119 -0.712224 0.055550
C -1.082790 0.162905 0.029146
C 0.901462 1.717364 -0.049737
C -0.555860 1.472544 -0.053633
C -2.472599 -0.202478 0.019776
C 3.137053 0.632158 -0.019030
C 1.853463 -1.870960 0.097245
C 3.253232 -1.756191 0.078251
C 3.896295 -0.523832 0.019746
C -2.811706 -1.519432 -0.351952
C -3.483630 0.705424 0.389076
C -4.135382 -1.906422 -0.374649
C -4.803539 0.295051 0.377748
C -5.132208 -1.001541 -0.009335
H 1.371398 -2.835821 0.140876
H 4.976013 -0.464058 0.003617
H -2.036511 -2.212436 -0.642449
H -3.238677 1.702985 0.713447
H -4.397181 -2.910062 -0.677329
H -5.579354 0.985172 0.676219
H -6.168219 -1.310603 -0.023978
H -0.769465 3.313248 -0.209154
H 3.099743 2.533355 -0.100339
H 4.891882 -2.740442 0.098742

SCF Energy: -953.719982375

Sum of electronic and zero-point Energies= -953.496623
Sum of electronic and thermal Energies= -953.480973
Sum of electronic and thermal Enthalpies= -953.480029
Sum of electronic and thermal Free Energies= -953.540960

==> ./AOX-III/water/Leu/C3 <==

O	-2.269023	1.437919	0.057947
O	-0.295821	-0.892528	1.954188
O	-4.875478	-2.581434	0.267166
O	-2.503512	-2.399341	1.365930
O	-6.458207	1.269924	-2.009561
C	-3.516945	-0.604683	0.217549
C	-3.389120	0.714396	-0.230613
C	-1.259291	0.912557	0.782738
C	-2.459405	-1.215910	0.981704
C	-1.304907	-0.369682	1.276985
C	-0.106435	1.809838	0.885828
C	-4.699449	-1.303390	-0.126898
C	-4.361125	1.354218	-0.972821
C	-5.508576	0.632760	-1.280726
C	-5.688596	-0.688902	-0.867774
C	0.260261	2.560803	-0.236053
C	0.655675	1.884141	2.054861
C	1.403975	3.345676	-0.198848
C	1.789043	2.683777	2.087699
C	2.173899	3.402008	0.959264
H	-4.231650	2.374262	-1.301977
H	-6.589836	-1.229237	-1.124746
H	-0.330975	2.502171	-1.139420
H	0.360436	1.322220	2.927891
H	1.698196	3.906704	-1.075227
H	2.376497	2.741670	2.993613
H	3.067983	4.010161	0.984447
H	0.604990	-1.313811	1.128720
H	-4.068565	-2.851605	0.759640
H	-7.203584	0.679394	-2.172499
O	5.035595	1.119933	-0.013057
N	4.640235	-1.667001	-0.072670
N	3.862673	1.266981	-1.939120
C	1.414009	-1.666410	0.219995
C	2.466139	-0.577442	0.314642
C	3.662951	-0.760302	-0.639750
C	1.831374	-3.056203	0.635610
C	0.563436	-1.612678	-1.024274
C	4.273598	0.623817	-0.845048
H	2.854587	-0.501666	1.333217
H	1.993976	0.374131	0.071044
H	3.329320	-1.144326	-1.604421
H	2.466709	-3.501847	-0.137109
H	2.389189	-3.043648	1.572815
H	0.957229	-3.697997	0.751882
H	1.148877	-1.941799	-1.890327
H	-0.296040	-2.279727	-0.937132
H	0.211540	-0.598811	-1.225561
C	5.777936	-1.940470	-0.710456
H	4.490645	-2.053488	0.846894
H	3.229505	0.833279	-2.591226
H	4.114402	2.234900	-2.068721
O	6.065314	-1.475607	-1.816203
H	6.444586	-2.619227	-0.172216

SCF Energy: -1488.54355885
Sum of electronic and zero-point Energies= -1488.120427
Sum of electronic and thermal Energies= -1488.090973
Sum of electronic and thermal Enthalpies= -1488.090029
Sum of electronic and thermal Free Energies= -1488.183497

==> ./AOX-III/water/Leu/C7 <==

O	2.740448	-0.333681	0.669186
O	5.291839	1.339837	-1.267775
O	1.032455	4.049259	-0.284652
O	3.410303	3.234335	-1.114547
O	-1.517297	0.928024	2.167545
C	1.945146	1.867861	0.159478
C	1.767288	0.608501	0.743060
C	3.912278	-0.102162	0.022689
C	3.170459	2.151233	-0.540565
C	4.148861	1.091936	-0.577218
C	4.803354	-1.266666	0.038952
C	0.898800	2.822641	0.270511
C	0.611635	0.264182	1.420759
C	-0.404934	1.224835	1.519971
C	-0.259067	2.504553	0.937170
C	4.245946	-2.549620	0.056526
C	6.192568	-1.114386	0.056990
C	5.070978	-3.664399	0.074396
C	7.010285	-2.235982	0.083627
C	6.453873	-3.510506	0.087295
H	0.494984	-0.714127	1.863162
H	-1.062305	3.223191	1.017945
H	3.172159	-2.670480	0.044662
H	6.629862	-0.127903	0.070059
H	4.634364	-4.653566	0.077837
H	8.084144	-2.112107	0.106687
H	7.095243	-4.381124	0.103551
H	5.204699	2.232775	-1.637747
H	1.904776	4.089270	-0.728325
H	-2.391248	0.314205	1.249042

O	-7.489187	0.377363	0.366897
N	-5.537414	-0.214306	-1.480780
N	-7.146063	-1.637797	1.334092
C	-2.967317	-0.250971	0.358174
C	-4.419377	0.000149	0.731062
C	-5.440427	-0.760012	-0.142601
C	-2.451557	0.428473	-0.889040
C	-2.514643	-1.688219	0.490908
C	-6.797630	-0.630038	0.534680
H	-4.647984	1.066075	0.669837
H	-4.572280	-0.320608	1.763832
H	-5.167334	-1.808644	-0.223276
H	-2.761095	-0.122839	-1.781275
H	-2.811173	1.454499	-0.968350
H	-1.357769	0.435250	-0.890214
H	-2.898302	-2.282771	-0.343815
H	-1.425089	-1.747433	0.459806
H	-2.867001	-2.131944	1.422807
C	-5.157618	-0.860457	-2.584406
H	-5.926640	0.712282	-1.580024
H	-6.554415	-2.446566	1.435602
H	-7.995863	-1.575401	1.873866
O	-4.667551	-1.991468	-2.610998
H	-5.318888	-0.282350	-3.498615

SCF Energy: -1488.53132438
Sum of electronic and zero-point Energies= -1488.108407
Sum of electronic and thermal Energies= -1488.078914
Sum of electronic and thermal Enthalpies= -1488.077970
Sum of electronic and thermal Free Energies= -1488.173083

==> ./AOX-III/water/Leu/C5 <==

O	3.162457	0.814856	0.191883
O	2.020704	-2.252585	-1.330245
O	-1.121489	1.906462	-1.661377
O	-0.015948	-0.675029	-1.817381
O	1.443018	5.115786	0.695612
C	0.995599	1.231539	-0.763079
C	2.130229	1.651667	-0.066990
C	3.142596	-0.485623	-0.210403
C	0.919638	-0.145160	-1.200381
C	2.074392	-0.971811	-0.881501
C	4.374541	-1.203235	0.136359
C	-0.035256	2.203494	-0.981260
C	2.288424	2.947996	0.419051
C	1.270664	3.860357	0.208011
C	0.109455	3.495167	-0.475428
C	5.579413	-0.495729	0.204012
C	4.362111	-2.573443	0.411777
C	6.756371	-1.155797	0.526114
C	5.543685	-3.224528	0.740294
C	6.742050	-2.521040	0.793949
H	3.187576	3.221693	0.951532
H	-0.685164	4.211911	-0.636575
H	5.593925	0.564222	-0.006016
H	3.434182	-3.123526	0.386287
H	7.685108	-0.603568	0.567303
H	5.525361	-4.283223	0.959487
H	7.660300	-3.033452	1.046767
H	1.174428	-2.327368	-1.803904
H	-1.995518	1.260560	-0.829887
H	0.673924	5.662198	0.492886
O	-4.837407	-3.099381	-0.905494
N	-4.087957	-1.691709	1.318793
N	-6.290170	-1.459339	-1.462364
C	-2.729103	0.843422	0.052798
C	-3.397424	-0.328430	-0.643828
C	-4.555225	-0.967267	0.155286
C	-1.775267	0.523266	1.179254
C	-3.630289	2.024249	0.334284
C	-5.254887	-1.945875	-0.778273
H	-2.664107	-1.107889	-0.859489
H	-3.811164	0.020590	-1.592465
H	-5.254880	-0.204881	0.488857
H	-2.325539	0.363312	2.111044
H	-1.181041	-0.365279	0.965954
H	-1.102151	1.366948	1.358361
H	-4.283565	1.806844	1.184719
H	-3.034949	2.901863	0.593508
H	-4.253992	2.262118	-0.528231
C	-4.320627	-1.321344	2.579086
H	-3.543973	-2.527613	1.160285
H	-6.598558	-0.509423	-1.331916
H	-6.736009	-2.031524	-2.163067
O	-4.961997	-0.325019	2.919496
H	-3.878547	-1.999142	3.314812

SCF Energy: -1488.52859573
Sum of electronic and zero-point Energies= -1488.105707
Sum of electronic and thermal Energies= -1488.075961
Sum of electronic and thermal Enthalpies= -1488.075017
Sum of electronic and thermal Free Energies= -1488.170605

==> ./AOX-III/pet/LM <==

O	2.403812	1.274062	0.139474
O	-0.626837	0.410598	1.849220
O	2.940451	-3.289716	1.503237
O	0.767939	-1.921242	2.031883

O	6.331861	-1.019571	-0.921736
C	2.606909	-0.998015	0.880214
C	3.126970	0.122247	0.223813
C	1.169507	1.382466	0.675677
C	1.294592	-0.945981	1.467032
C	0.574402	0.331764	1.358161
C	0.551139	2.677876	0.402706
C	3.396871	-2.172856	0.900397
C	4.370172	0.132256	-0.375337
C	5.114910	-1.040466	-0.323947
C	4.642250	-2.194184	0.305199
C	0.861136	3.335374	-0.794822
C	-0.328686	3.275140	1.312803
C	0.282044	4.561444	-1.084098
C	-0.892719	4.508357	1.019575
C	-0.595855	5.150093	-0.178658
H	4.742551	1.014397	-0.874118
H	5.238968	-3.096159	0.326503
H	1.539702	2.876533	-1.499996
H	-0.549796	2.786841	2.248765
H	0.515455	5.057769	-2.015911
H	-1.562643	4.971056	1.731041
H	-1.043597	6.108321	-0.404396
H	-1.492211	-0.006230	0.858764
H	2.040725	-3.093233	1.847341
H	6.766727	-1.875820	-0.828819
O	-7.018212	-0.299691	-0.804622
O	-7.355009	-2.470307	-0.404613
C	-2.066346	-0.331539	-0.195388
C	1.046480	-1.214461	-2.318542
C	-0.994125	-1.063704	-0.858708
C	-0.071734	-0.482876	-1.651407
C	-3.252075	-1.082899	0.249548
C	-4.470157	-0.546276	0.353022
C	-5.657900	-1.314790	0.852943
C	0.871917	-1.224111	-3.841634
C	-6.749200	-1.447420	-0.170961
H	-2.286166	0.642651	-0.631372
H	1.991719	-0.719629	-2.079507
H	1.099331	-2.234630	-1.934942
H	-0.903136	-2.123676	-0.635016
H	-0.165824	0.578264	-1.871360
H	-3.097889	-2.120820	0.531571
H	-4.628226	0.490059	0.075700
H	-6.110543	-0.791708	1.701861
H	-5.378624	-2.315201	1.172946
H	0.812025	-0.205721	-4.228060
H	1.715029	-1.721893	-4.320577
H	-0.042526	-1.748521	-4.120043
H	-7.736103	-0.450347	-1.438659

SCF Energy: -1455.18164415

Sum of electronic and zero-point Energies= -1454.759267

Sum of electronic and thermal Energies= -1454.730015

Sum of electronic and thermal Enthalpies= -1454.729071

Sum of electronic and thermal Free Energies= -1454.824556

==> ./AOX-III/pet/_BDE/c5 <==

O	-0.163710	-0.811729	0.062971
O	-1.375276	2.569849	-0.127931
O	3.803151	1.803701	-0.073428
O	1.178150	2.980990	-0.162518
O	3.974604	-2.909477	0.154185
C	1.731257	0.645699	-0.019331
C	1.162396	-0.608356	0.045655
C	-1.047766	0.226261	0.016097
C	0.857828	1.799618	-0.079730
C	-0.576737	1.493130	-0.052656
C	-2.445473	-0.216858	0.015425
C	3.199555	0.732354	-0.025326
C	1.919821	-1.801000	0.102723
C	3.314485	-1.731641	0.095574
C	3.945653	-0.511930	0.031949
C	-2.756071	-1.510027	-0.419859
C	-3.473368	0.626180	0.449906
C	-4.072661	-1.945192	-0.431321
C	-4.787351	0.179311	0.440756
C	-5.092349	-1.102558	-0.001809
H	1.410928	-2.752369	0.151568
H	5.024705	-0.429240	0.024800
H	-1.969610	-2.169664	-0.756587
H	-3.246370	1.620571	0.800226
H	-4.302259	-2.943767	-0.777574
H	-5.574532	0.836661	0.784149
H	-6.118534	-1.444283	-0.010567
H	-0.761573	3.325240	-0.193424
H	4.926573	-2.759768	0.142863

SCF Energy: -953.277244792

Sum of electronic and zero-point Energies= -953.066843

Sum of electronic and thermal Energies= -953.051144

Sum of electronic and thermal Enthalpies= -953.050200

Sum of electronic and thermal Free Energies= -953.111366

==> ./AOX-III/pet/_BDE/c7 <==

O	-0.207673	-0.895549	0.077398
O	-1.264908	2.537708	-0.146543

O	3.767708	1.773020	-0.092954
O	1.357153	2.817726	-0.146736
O	3.988948	-2.912237	0.156626
C	1.749626	0.478619	-0.008279
C	1.138485	-0.770262	0.060097
C	-1.029021	0.177752	0.018599
C	0.922449	1.653340	-0.068131
C	-0.502883	1.434567	-0.055556
C	-2.446409	-0.188612	0.013540
C	3.195694	0.564703	-0.023532
C	1.878718	-1.938402	0.117324
C	3.329949	-1.861778	0.105597
C	3.952299	-0.561117	0.033249
C	-2.818455	-1.463580	-0.429639
C	-3.429916	0.702536	0.456059
C	-4.153943	-1.832838	-0.442232
C	-4.763176	0.319029	0.448645
C	-5.129375	-0.943473	-0.003111
H	1.401162	-2.905362	0.170320
H	5.030963	-0.502875	0.023715
H	-2.065262	-2.158003	-0.771898
H	-3.154906	1.681430	0.814642
H	-4.433764	-2.815942	-0.795147
H	-5.517065	1.010118	0.799804
H	-6.170947	-1.234886	-0.011336
H	-0.646741	3.284972	-0.215088
H	3.062473	2.452284	-0.133106

SCF Energy: -953.284415614
Sum of electronic and zero-point Energies= -953.073525
Sum of electronic and thermal Energies= -953.058263
Sum of electronic and thermal Enthalpies= -953.057318
Sum of electronic and thermal Free Energies= -953.117032

==> ./AOX-III/pet/_BDE/c3 <==

O	-0.246537	-0.815521	0.055219
O	-1.316621	2.564576	-0.157712
O	3.756808	1.806807	-0.053074
O	1.390228	2.896252	-0.074778
O	3.909344	-2.921890	0.102136
C	1.715374	0.571266	-0.004785
C	1.112492	-0.690432	0.038807
C	-1.084826	0.233509	0.008976
C	0.914277	1.764770	-0.043164
C	-0.585095	1.577683	-0.064241
C	-2.481916	-0.154996	0.008048
C	3.131196	0.630747	-0.012749
C	1.832983	-1.861362	0.075043
C	3.225809	-1.762394	0.065318
C	3.880488	-0.533729	0.020639
C	-2.815112	-1.489989	-0.285868
C	-3.510336	0.753958	0.309943
C	-4.137267	-1.896050	-0.292580
C	-4.829953	0.330712	0.309634
C	-5.149798	-0.988050	0.005736
H	1.342484	-2.822268	0.111169
H	4.960654	-0.472164	0.011857
H	-2.036182	-2.199106	-0.521877
H	-3.272760	1.776333	0.549710
H	-4.380139	-2.922331	-0.531369
H	-5.612810	1.036551	0.550629
H	-6.182770	-1.308588	0.002764
H	3.067708	2.508546	-0.070728
H	4.858131	-2.752625	0.090973

SCF Energy: -953.298855839
Sum of electronic and zero-point Energies= -953.087495
Sum of electronic and thermal Energies= -953.072096
Sum of electronic and thermal Enthalpies= -953.071152
Sum of electronic and thermal Free Energies= -953.131930

==> ./AOX-III/pet <==

O	-0.214863	-0.875288	0.071186
O	-1.346965	2.553138	-0.138648
O	3.707510	1.844302	-0.091544
O	1.277660	2.867781	-0.144133
O	4.000253	-2.881635	0.152361
C	1.706830	0.540265	-0.011614
C	1.129790	-0.732955	0.055244
C	-1.058902	0.193320	0.016384
C	0.862373	1.694825	-0.068935
C	-0.565844	1.453604	-0.054267
C	-2.470571	-0.207271	0.013817
C	3.119508	0.644337	-0.025208
C	1.890508	-1.887385	0.110623
C	3.271966	-1.746286	0.097503
C	3.895763	-0.495285	0.030714
C	-2.817751	-1.489539	-0.426084
C	-3.473857	0.662144	0.453787
C	-4.145972	-1.887508	-0.437998
C	-4.799810	0.252024	0.445594
C	-5.141003	-1.018669	-0.002762
H	1.427835	-2.861138	0.163286
H	4.974248	-0.408575	0.021634
H	-2.049365	-2.169103	-0.764188
H	-3.218518	1.647850	0.808882
H	-4.403896	-2.877965	-0.787622
H	-5.567754	0.929144	0.794088

H	-6.176315	-1.331793	-0.010811
H	-0.734187	3.303417	-0.207252
H	3.000007	2.522938	-0.129766
H	4.941002	-2.674893	0.137195

SCF Energy: -953.944373578
Sum of electronic and zero-point Energies= -953.719896
Sum of electronic and thermal Energies= -953.704431
Sum of electronic and thermal Enthalpies= -953.703487
Sum of electronic and thermal Free Energies= -953.762808

==> ./AOX-I/pet/aIP <==

O	-0.240125	-0.847410	0.073392
O	-1.292712	2.541471	-0.082499
O	3.753916	1.806793	-0.086127
O	1.281933	2.886046	-0.080435
O	3.930625	-2.907669	0.095151
C	1.721558	0.554315	-0.003926
C	1.127145	-0.716760	0.049576
C	-1.091430	0.170094	0.036829
C	0.906421	1.710563	-0.029438
C	-0.552394	1.475211	-0.013809
C	-2.475340	-0.197373	0.015814
C	3.139702	0.632407	-0.031868
C	1.851177	-1.873467	0.082118
C	3.254902	-1.757594	0.058575
C	3.898039	-0.525792	-0.001176
C	-2.804206	-1.548609	-0.242558
C	-3.506963	0.736423	0.259368
C	-4.122519	-1.943040	-0.270192
C	-4.822279	0.319724	0.240670
C	-5.133722	-1.011533	-0.027497
H	1.376777	-2.842374	0.122315
H	4.977216	-0.455980	-0.024628
H	-2.022774	-2.267022	-0.437225
H	-3.284634	1.765411	0.484771
H	-4.372213	-2.972879	-0.481173
H	-5.610628	1.031168	0.439077
H	-6.167801	-1.326896	-0.046153
H	-0.684664	3.313748	-0.118463
H	3.095466	2.526321	-0.102563
H	4.883613	-2.752761	0.073281

SCF Energy: -953.701936212
Sum of electronic and zero-point Energies= -953.477414
Sum of electronic and thermal Energies= -953.461955
Sum of electronic and thermal Enthalpies= -953.461010
Sum of electronic and thermal Free Energies= -953.521056

==> ./AOX-I/pet/HAT/C3/TS <==

O	-0.052194	-1.260259	0.100789
O	-1.410629	1.851241	-0.979841
O	3.706857	1.645971	-0.443454
O	1.245358	2.508365	-0.617222
O	4.273474	-2.952328	0.528709
C	1.779857	0.251686	-0.202860
C	1.290613	-1.033266	0.057862
C	-0.953034	-0.301630	-0.164184
C	0.869911	1.330633	-0.455191
C	-0.554232	0.983420	-0.514617
C	-2.329192	-0.781321	-0.098710
C	3.184882	0.441470	-0.205218
C	2.110412	-2.111418	0.305702
C	3.486033	-1.887350	0.286453
C	4.029360	-0.627210	0.034412
C	-2.589028	-2.138753	-0.329629
C	-3.383752	0.080560	0.227370
C	-3.886271	-2.618601	-0.258596
C	-4.675679	-0.413621	0.312220
C	-4.931305	-1.757903	0.062712
H	1.706031	-3.090650	0.512548
H	5.100006	-0.471365	0.027739
H	-1.777936	-2.807788	-0.577723
H	-3.191930	1.121807	0.431812
H	-4.082798	-3.663960	-0.452696
H	-5.484837	0.253371	0.575448
H	-5.943245	-2.135276	0.122018
H	2.962380	2.272546	-0.577040
H	5.202357	-2.697243	0.496312
H	-1.448887	2.804521	-0.355165
O	-1.495690	3.610881	0.570265
O	-0.521258	3.175493	1.410502
H	0.300063	3.278963	0.892408

SCF Energy: -1104.85829883
Sum of electronic and zero-point Energies= -1104.621630
Sum of electronic and thermal Energies= -1104.603515
Sum of electronic and thermal Enthalpies= -1104.602571
Sum of electronic and thermal Free Energies= -1104.669037

==> ./AOX-I/pet/HAT/C3 <==

O	-0.246527	-0.814905	0.054257
O	-1.315588	2.565851	-0.152678

O	3.757687	1.806265	-0.052477
O	1.390940	2.896312	-0.072992
O	3.908818	-2.922408	0.099765
C	1.715817	0.571328	-0.004801
C	1.112550	-0.690150	0.038067
C	-1.085128	0.234079	0.009244
C	0.914863	1.764905	-0.041969
C	-0.584752	1.578276	-0.062013
C	-2.482313	-0.154633	0.008070
C	3.131614	0.630378	-0.012882
C	1.832814	-1.861278	0.073460
C	3.225626	-1.762687	0.063701
C	3.880627	-0.534237	0.019702
C	-2.814844	-1.491621	-0.277895
C	-3.512092	0.755730	0.301460
C	-4.136802	-1.898238	-0.284960
C	-4.831559	0.332010	0.300610
C	-5.150405	-0.988764	0.004808
H	1.342395	-2.822216	0.108958
H	4.960784	-0.473030	0.010859
H	-2.035411	-2.202310	-0.507167
H	-3.275908	1.779851	0.534830
H	-4.378683	-2.926198	-0.517381
H	-5.615248	1.039204	0.534788
H	-6.183245	-1.309637	0.001465
H	3.068950	2.508160	-0.069476
H	4.857626	-2.753332	0.088516

SCF Energy: -953.295775200
Sum of electronic and zero-point Energies= -953.084406
Sum of electronic and thermal Energies= -953.069004
Sum of electronic and thermal Enthalpies= -953.068060
Sum of electronic and thermal Free Energies= -953.128871

==> ./AOX-I/pet/HAT/C7/TS <==

O	-0.460439	-0.565587	-0.278570
O	-2.202374	2.527110	0.355364
O	2.867744	2.858616	-0.070462
O	0.303580	3.357243	0.265656
O	4.059357	-1.611921	-0.910307
C	1.160090	1.184177	-0.155316
C	0.823280	-0.152981	-0.340918
C	-1.483517	0.294253	-0.043390
C	0.116059	2.137530	0.100740
C	-1.231268	1.618413	0.145048
C	-2.791634	-0.366251	-0.055137
C	2.535688	1.575367	-0.239002
C	1.785795	-1.120681	-0.600222
C	3.140480	-0.716325	-0.670413
C	3.504643	0.636783	-0.480964
C	-2.939807	-1.573137	-0.748916
C	-3.887539	0.176330	0.624107
C	-4.166926	-2.217023	-0.771829
C	-5.109554	-0.480683	0.601896
C	-5.254822	-1.673355	-0.096847
H	1.509526	-2.150100	-0.768941
H	4.545426	0.919868	-0.539249
H	-2.098446	-2.000465	-1.274560
H	-3.784289	1.097462	1.174882
H	-4.273711	-3.144613	-1.317305
H	-5.949892	-0.058191	1.135489
H	-6.210904	-2.178798	-0.114001
H	-1.753416	3.386289	0.421602
H	2.047147	3.371894	0.086967
H	4.084530	-2.393331	-0.047086
O	4.025924	-2.948317	1.024628
O	2.851348	-2.485627	1.540792
H	3.117107	-1.836664	2.212364

SCF Energy: -1104.85001914
Sum of electronic and zero-point Energies= -1104.614556
Sum of electronic and thermal Energies= -1104.595970
Sum of electronic and thermal Enthalpies= -1104.595026
Sum of electronic and thermal Free Energies= -1104.664129

==> ./AOX-I/pet/HAT/C7 <==

O	-0.207441	-0.895629	0.075525
O	-1.264955	2.537694	-0.145156
O	3.768625	1.772846	-0.092299
O	1.356815	2.817935	-0.146101
O	3.989407	-2.912164	0.157264
C	1.750014	0.478663	-0.009042
C	1.138648	-0.770244	0.058888
C	-1.029100	0.177674	0.017919
C	0.922525	1.653388	-0.068192
C	-0.502893	1.434480	-0.055667
C	-2.446687	-0.188710	0.013324
C	3.196050	0.564755	-0.023468
C	1.878984	-1.938261	0.116097
C	3.330263	-1.861768	0.105548
C	3.952620	-0.560999	0.033478
C	-2.819036	-1.464416	-0.427486
C	-3.430429	0.703218	0.453878
C	-4.154553	-1.833595	-0.439737
C	-4.763736	0.319784	0.446932
C	-5.130032	-0.943442	-0.002520
H	1.401900	-2.905481	0.168687
H	5.031312	-0.503155	0.024712

H	-2.065955	-2.159750	-0.768060
H	-3.155862	1.682944	0.810450
H	-4.434340	-2.817348	-0.790824
H	-5.517641	1.011592	0.796594
H	-6.171604	-1.234764	-0.010517
H	-0.646899	3.285110	-0.212539
H	3.063640	2.452225	-0.132087

SCF Energy: -953.281241518
Sum of electronic and zero-point Energies= -953.070340
Sum of electronic and thermal Energies= -953.055081
Sum of electronic and thermal Enthalpies= -953.054137
Sum of electronic and thermal Free Energies= -953.113822

==> ./AOX-I/pet/HAT/C5/TS <==

O	0.695255	1.014072	0.034673
O	1.447498	-2.446523	-0.635607
O	-3.550163	-0.997754	-0.868795
O	-1.140325	-2.464207	-0.864319
O	-3.167374	3.597547	0.160590
C	-1.371236	-0.121757	-0.409125
C	-0.646240	1.025385	-0.102805
C	1.424167	-0.126245	-0.129801
C	-0.655982	-1.370390	-0.588284
C	0.795965	-1.285754	-0.434397
C	2.865138	0.098904	0.029235
C	-2.795658	0.006747	-0.530443
C	-1.247333	2.270829	0.087336
C	-2.626799	2.374898	-0.028965
C	-3.400056	1.257960	-0.321593
C	3.380466	1.388776	-0.143607
C	3.733816	-0.946047	0.361436
C	4.739357	1.623983	0.000950
C	5.091520	-0.699362	0.510684
C	5.599557	0.581486	0.328475
H	-0.643231	3.135340	0.321365
H	-4.476060	1.324956	-0.418370
H	2.718845	2.203708	-0.398046
H	3.350608	-1.942747	0.510415
H	5.126675	2.623536	-0.142791
H	5.753373	-1.513456	0.773162
H	6.659063	0.767151	0.442532
H	0.746974	-3.089562	-0.847433
H	-4.123063	3.563266	0.042316
H	-3.527611	-1.816830	-0.043997
O	-3.637915	-2.298269	1.063003
O	-2.830183	-1.495524	1.810659
H	-3.440028	-1.006581	2.384648

SCF Energy: -1104.83879253
Sum of electronic and zero-point Energies= -1104.603590
Sum of electronic and thermal Energies= -1104.584737
Sum of electronic and thermal Enthalpies= -1104.583793
Sum of electronic and thermal Free Energies= -1104.652802

==> ./AOX-I/pet/HAT/C5 <==

O	-0.163702	-0.811329	0.061428
O	-1.374484	2.570764	-0.125981
O	3.803775	1.803306	-0.072542
O	1.178729	2.981179	-0.160121
O	3.974371	-2.909792	0.152630
C	1.731540	0.645769	-0.019383
C	1.162455	-0.608172	0.044571
C	-1.047974	0.226718	0.015574
C	0.858240	1.799752	-0.078727
C	-0.576489	1.493536	-0.052109
C	-2.445814	-0.216560	0.015156
C	3.199848	0.732125	-0.025047
C	1.919814	-1.800977	0.101071
C	3.314397	-1.731869	0.094496
C	3.945758	-0.512238	0.031804
C	-2.755731	-1.512204	-0.413380
C	-3.474952	0.628377	0.443191
C	-4.072168	-1.947806	-0.424714
C	-4.788795	0.181056	0.434246
C	-5.092860	-1.103223	-0.001700
H	1.410931	-2.752357	0.149228
H	5.024794	-0.429687	0.025009
H	-1.968756	-2.173793	-0.744831
H	-3.249414	1.624812	0.788399
H	-4.300861	-2.948378	-0.765714
H	-5.576708	0.840093	0.772649
H	-6.118923	-1.445238	-0.010334
H	-0.760560	3.326070	-0.190243
H	4.926378	-2.760256	0.142224

SCF Energy: -953.274143360
Sum of electronic and zero-point Energies= -953.063749
Sum of electronic and thermal Energies= -953.048043
Sum of electronic and thermal Enthalpies= -953.047099
Sum of electronic and thermal Free Energies= -953.108367

==> ./AOX-I/pet <==

O	-0.214759	-0.875135	0.069170

O	-1.346650	2.553377	-0.137720
O	3.708279	1.843965	-0.090211
O	1.277980	2.867897	-0.143060
O	4.000098	-2.881838	0.151896
C	1.707134	0.540315	-0.012099
C	1.129938	-0.732810	0.054026
C	-1.058992	0.193451	0.015596
C	0.862653	1.694939	-0.068765
C	-0.565637	1.453729	-0.054431
C	-2.470873	-0.207108	0.013493
C	3.119873	0.644185	-0.024776
C	1.890636	-1.887340	0.109329
C	3.272034	-1.746413	0.097046
C	3.895974	-0.495491	0.031091
C	-2.818101	-1.490659	-0.422711
C	-3.474664	0.663409	0.450306
C	-4.146285	-1.888764	-0.434117
C	-4.800586	0.253131	0.442718
C	-5.141602	-1.018822	-0.001970
H	1.428164	-2.861185	0.161471
H	4.974436	-0.408860	0.022714
H	-2.049746	-2.171410	-0.758345
H	-3.220037	1.650310	0.802439
H	-4.403975	-2.880253	-0.780930
H	-5.568747	0.931197	0.788846
H	-6.176871	-1.332019	-0.009583
H	-0.733972	3.303838	-0.204875
H	3.001267	2.523027	-0.128269
H	4.940886	-2.675283	0.137530

SCF Energy: -953.941098764
Sum of electronic and zero-point Energies= -953.716611
Sum of electronic and thermal Energies= -953.701147
Sum of electronic and thermal Enthalpies= -953.700203
Sum of electronic and thermal Free Energies= -953.759510

==> ./AOX-I/pet/RAF/C4a <==

O	-0.485751	0.987065	0.074745
O	-1.413754	-2.394453	-0.806219
O	3.611424	-1.517139	-0.318612
O	1.234153	-2.577260	-0.696175
O	3.568397	3.226650	-0.601767
C	1.539602	-0.358467	0.198686
C	0.869454	0.933161	-0.039296
C	-1.261567	-0.100213	-0.163790
C	0.731351	-1.492641	-0.382852
C	-0.687680	-1.296231	-0.471411
C	-2.691555	0.206343	-0.060608
C	2.970415	-0.343198	-0.234177
C	1.548709	2.095605	-0.247329
C	2.939056	2.050279	-0.409655
C	3.636980	0.827021	-0.442859
C	-3.086269	1.366396	0.619829
C	-3.671678	-0.616557	-0.629073
C	-4.429318	1.686730	0.738362
C	-5.013398	-0.280787	-0.513014
C	-5.398328	0.865400	0.171801
H	1.020396	3.035046	-0.326584
H	4.693677	0.798902	-0.678645
H	-2.341020	2.009797	1.062813
H	-3.389688	-1.505824	-1.167355
H	-4.719254	2.580144	1.274508
H	-5.760296	-0.920827	-0.962663
H	-6.446075	1.118468	0.262551
H	-0.776782	-3.116358	-0.914861
H	2.936745	-2.225314	-0.376839
H	4.513755	3.084315	-0.723387
O	1.473951	-0.500690	1.697494
O	1.940805	-1.787206	2.078179
H	2.860087	-1.611123	2.321631

SCF Energy: -1104.84116962
Sum of electronic and zero-point Energies= -1104.599901
Sum of electronic and thermal Energies= -1104.581281
Sum of electronic and thermal Enthalpies= -1104.580337
Sum of electronic and thermal Free Energies= -1104.647698

==> ./AOX-I/pet/RAF/C4a/TS <==

O	-0.482402	0.987952	0.136356
O	-1.456693	-2.326455	-0.926689
O	3.571796	-1.471160	-0.578719
O	1.198223	-2.525570	-0.910295
O	3.590724	3.255316	-0.168792
C	1.528411	-0.351863	0.035738
C	0.868267	0.936321	0.020196
C	-1.267811	-0.084957	-0.142773
C	0.710116	-1.458783	-0.515473
C	-0.714063	-1.265567	-0.522957
C	-2.696853	0.223564	-0.036806
C	2.944275	-0.316168	-0.341558
C	1.560145	2.114665	0.005144
C	2.949251	2.072470	-0.160459
C	3.635804	0.865885	-0.364113
C	-3.122860	1.552023	-0.152401
C	-3.640768	-0.782058	0.197490
C	-4.469985	1.863747	-0.052058
C	-4.985892	-0.459221	0.304402
C	-5.405324	0.859941	0.176028

H	1.046257	3.061820	0.081744
H	4.697518	0.857407	-0.575654
H	-2.400502	2.335730	-0.326633
H	-3.323927	-1.807025	0.306259
H	-4.789756	2.892172	-0.150870
H	-5.707485	-1.242450	0.492477
H	-6.455695	1.105584	0.256486
H	-0.826299	-3.028433	-1.147696
H	2.889698	-2.171000	-0.679246
H	4.536367	3.123969	-0.302038
O	1.521123	-0.692135	1.689031
O	2.055665	-1.972111	1.915881
H	2.990069	-1.794477	2.088595

SCF Energy: -1104.83990332
Sum of electronic and zero-point Energies= -1104.599387
Sum of electronic and thermal Energies= -1104.581048
Sum of electronic and thermal Enthalpies= -1104.580104
Sum of electronic and thermal Free Energies= -1104.646690

==> ./AOX-I/pet/RAF/C6 <==

O	-0.746858	-0.813328	-0.072080
O	-2.083961	2.540683	-0.089753
O	3.009697	2.124650	-0.400514
O	0.516991	3.009888	-0.260707
O	3.567818	-2.617761	-0.411112
C	1.086936	0.710945	-0.185555
C	0.574907	-0.587197	-0.164833
C	-1.655088	0.197606	-0.026090
C	0.167703	1.815125	-0.182445
C	-1.235415	1.488959	-0.088271
C	-3.036837	-0.287042	0.058272
C	2.467744	0.909667	-0.273177
C	1.413606	-1.723656	-0.237839
C	2.755049	-1.550745	-0.302674
C	3.426532	-0.213945	-0.237788
C	-3.338495	-1.580792	-0.382611
C	-4.056879	0.512483	0.584714
C	-4.637878	-2.058772	-0.308668
C	-5.353327	0.022703	0.660464
C	-5.649563	-1.259232	0.212563
H	0.983859	-2.715032	-0.244624
H	-2.557641	-2.207607	-0.787740
H	-3.837214	1.506125	0.941385
H	-4.860291	-3.057574	-0.658877
H	-6.133569	0.646475	1.074987
H	-6.662348	-1.634468	0.271184
H	-1.523049	3.328974	-0.172998
H	2.273691	2.775227	-0.384664
H	4.468417	-2.359192	-0.171089
H	4.176182	-0.107595	-1.029599
O	4.165775	-0.105215	1.028028
O	5.329278	-0.923310	0.914284
H	6.028071	-0.276831	0.740491

SCF Energy: -1104.86550263
Sum of electronic and zero-point Energies= -1104.622589
Sum of electronic and thermal Energies= -1104.604339
Sum of electronic and thermal Enthalpies= -1104.603394
Sum of electronic and thermal Free Energies= -1104.669733

==> ./AOX-I/pet/RAF/C5/TS <==

O	-0.702107	0.935796	-0.160492
O	-1.639142	-2.551324	-0.138404
O	3.342842	-1.521974	-0.737532
O	0.991305	-2.716961	-0.356658
O	3.347658	3.227464	-0.542963
C	1.272968	-0.366131	-0.335074
C	0.637132	0.856558	-0.257052
C	-1.490712	-0.175450	-0.120260
C	0.508614	-1.568556	-0.303223
C	-0.927712	-1.408318	-0.193478
C	-2.913316	0.149410	0.027940
C	2.720553	-0.394592	-0.339656
C	1.334376	2.073142	-0.302966
C	2.712192	2.037226	-0.454985
C	3.409885	0.834578	-0.532220
C	-3.279666	1.381353	0.581957
C	-3.909933	-0.741261	-0.383925
C	-4.618601	1.708622	0.731440
C	-5.247522	-0.401504	-0.236330
C	-5.606639	0.819056	0.323071
H	0.805400	3.011785	-0.234525
H	4.485215	0.810170	-0.646726
H	-2.517456	2.076645	0.901947
H	-3.643386	-1.688078	-0.825596
H	-4.890339	2.659776	1.168586
H	-6.010810	-1.094216	-0.563610
H	-6.650704	1.076935	0.438942
H	-0.984304	-3.268485	-0.174991
H	2.692990	-2.251968	-0.650185
H	4.300767	3.094677	-0.587124
O	2.948936	-0.502763	1.505710
O	4.266569	-0.811486	1.744619
H	4.354436	-1.722052	1.427608

SCF Energy: -1104.84841939

Sum of electronic and zero-point Energies= -1104.607909
Sum of electronic and thermal Energies= -1104.589605
Sum of electronic and thermal Enthalpies= -1104.588661
Sum of electronic and thermal Free Energies= -1104.655258

==> ./AOX-I/pet/RAF/C5 <==

O	-0.716195	0.931157	-0.087543
O	-1.630903	-2.556194	-0.120372
O	3.308232	-1.377595	-0.935042
O	0.993227	-2.709258	-0.277557
O	3.271352	3.299212	-0.545614
C	1.269014	-0.356288	-0.154831
C	0.622195	0.848703	-0.130509
C	-1.505037	-0.181357	-0.070738
C	0.514481	-1.563860	-0.193286
C	-0.929639	-1.408850	-0.132072
C	-2.932714	0.138270	0.031236
C	2.769286	-0.397667	-0.107321
C	1.306707	2.087054	-0.200032
C	2.697393	2.080082	-0.380501
C	3.414235	0.914659	-0.408682
C	-3.322276	1.368418	0.573139
C	-3.911784	-0.756968	-0.411561
C	-4.666791	1.690133	0.678717
C	-5.254930	-0.422915	-0.307448
C	-5.637382	0.796291	0.239230
H	0.755053	3.014212	-0.172594
H	4.483556	0.909066	-0.565854
H	-2.574576	2.067420	0.918413
H	-3.626905	-1.703028	-0.843385
H	-4.956548	2.640187	1.106626
H	-6.004134	-1.119339	-0.658585
H	-6.685721	1.049741	0.321206
H	-0.965035	-3.264615	-0.160093
H	2.732523	-2.158307	-0.845006
H	4.228623	3.207662	-0.600117
O	3.021413	-0.753847	1.277995
O	4.424394	-0.830686	1.473525
H	4.644462	-1.679551	1.064211

SCF Energy: -1104.86683143
Sum of electronic and zero-point Energies= -1104.624684
Sum of electronic and thermal Energies= -1104.606471
Sum of electronic and thermal Enthalpies= -1104.605526
Sum of electronic and thermal Free Energies= -1104.671547

==> ./AOX-I/pet/RAF/C1p <==

O	-0.050841	-0.806327	0.231603
O	1.019749	2.624930	-0.129932
O	-4.023560	1.789723	-0.331193
O	-1.615125	2.878516	-0.309763
O	-4.213459	-2.916871	0.190791
C	-1.996875	0.551691	-0.055397
C	-1.396163	-0.700222	0.127527
C	0.754889	0.280208	0.150317
C	-1.180730	1.724545	-0.142546
C	0.253364	1.520030	-0.029911
C	2.232780	-0.054072	0.270546
C	-3.409417	0.614273	-0.155621
C	-2.130164	-1.869710	0.210364
C	-3.511044	-1.767865	0.108157
C	-4.159343	-0.540598	-0.072696
C	2.466897	-1.505468	0.546952
C	2.965148	0.445239	-0.938653
C	3.303597	-2.260258	-0.207806
C	3.781881	-0.358503	-1.668344
C	3.974492	-1.715989	-1.326604
H	1.968748	-1.917321	1.412915
H	2.816081	1.482702	-1.200836
H	3.467284	-3.296899	0.054982
H	4.293802	0.048705	-2.530443
H	4.634792	-2.333997	-1.916224
H	0.402809	3.362843	-0.262098
H	-3.336223	2.487374	-0.368753
H	-5.156579	-2.738010	0.109462
H	-5.237373	-0.484766	-0.148443
H	-1.647372	-2.825166	0.349099
O	2.605642	0.724272	1.458459
O	3.991988	0.551520	1.693426
H	4.383009	1.268627	1.175505

SCF Energy: -1104.85312825
Sum of electronic and zero-point Energies= -1104.612209
Sum of electronic and thermal Energies= -1104.593787
Sum of electronic and thermal Enthalpies= -1104.592843
Sum of electronic and thermal Free Energies= -1104.659183

==> ./AOX-I/pet/RAF/C6p <==

O	0.319389	-1.068026	-0.044054
O	-1.150929	2.188893	-0.614200
O	3.913694	2.076134	-0.134666
O	1.397517	2.801722	-0.439420
O	4.714814	-2.556711	0.469472
C	2.072314	0.554538	-0.099717

C	1.636080	-0.769604	0.020178
C	-0.646754	-0.115239	-0.227256
C	1.111118	1.594218	-0.308053
C	-0.278016	1.195029	-0.377505
C	-1.975166	-0.663039	-0.263509
C	3.460394	0.822461	-0.022074
C	2.515526	-1.820178	0.211166
C	3.870327	-1.519950	0.282625
C	4.353643	-0.212569	0.168890
C	-2.168231	-2.035950	-0.316199
C	-3.155954	0.260883	-0.155625
C	-3.440698	-2.591664	-0.365695
C	-4.479069	-0.418168	-0.302115
C	-4.598716	-1.760057	-0.377000
H	-1.309390	-2.689561	-0.342788
H	-3.551447	-3.664875	-0.413883
H	-5.575944	-2.217317	-0.456233
H	-0.621489	3.003319	-0.644296
H	3.139436	2.663464	-0.269578
H	5.624454	-2.242060	0.507359
H	5.413000	-0.001995	0.228239
H	2.162874	-2.836021	0.304671
H	-5.351783	0.220274	-0.327637
H	-3.077978	1.092840	-0.853083
O	-3.031162	0.837703	1.177175
O	-3.900222	1.960425	1.243332
H	-4.617328	1.626560	1.797465

SCF Energy: -1104.86675839
Sum of electronic and zero-point Energies= -1104.625148
Sum of electronic and thermal Energies= -1104.606696
Sum of electronic and thermal Enthalpies= -1104.605752
Sum of electronic and thermal Free Energies= -1104.672520

==> ./AOX-I/pet/RAF/C2 <==

O	-0.070104	-0.932481	-0.145179
O	-1.366242	2.406846	0.087550
O	3.712897	2.012935	-0.300409
O	1.227483	2.921155	-0.333748
O	4.262958	-2.691037	-0.190679
C	1.789446	0.604882	-0.171010
C	1.280347	-0.702458	-0.126667
C	-0.940082	0.037469	0.404945
C	0.883959	1.727461	-0.172948
C	-0.489794	1.424077	0.051967
C	-2.342609	-0.262852	-0.070747
C	3.186106	0.784857	-0.235882
C	2.104442	-1.803267	-0.133154
C	3.485156	-1.588881	-0.186337
C	4.034871	-0.312464	-0.234022
C	-2.547926	-0.853375	-1.312548
C	-3.426947	0.124993	0.710095
C	-3.844004	-1.068875	-1.767495
C	-4.718701	-0.093382	0.251579
C	-4.929282	-0.690543	-0.987102
H	1.700212	-2.804286	-0.110495
H	5.105442	-0.162013	-0.277499
H	-1.703034	-1.148498	-1.918021
H	-3.260175	0.584860	1.673910
H	-4.002665	-1.533289	-2.731334
H	-5.560961	0.201308	0.862719
H	-5.937061	-0.860187	-1.341822
H	-0.870423	3.230312	-0.080365
H	2.975507	2.657353	-0.337049
H	5.193063	-2.442031	-0.227096
O	-0.816357	0.025137	1.823762
O	-1.245521	-1.234260	2.318350
H	-0.430682	-1.755749	2.285818

SCF Energy: -1104.89536496
Sum of electronic and zero-point Energies= -1104.652379
Sum of electronic and thermal Energies= -1104.634303
Sum of electronic and thermal Enthalpies= -1104.633358
Sum of electronic and thermal Free Energies= -1104.699159

==> ./AOX-I/pet/RAF/C2/TS <==

O	-0.152952	-0.930947	-0.018184
O	-1.346334	2.431869	-0.448757
O	3.719309	1.834343	-0.556267
O	1.270131	2.821792	-0.612789
O	4.099544	-2.850403	0.043572
C	1.746721	0.515703	-0.280233
C	1.194469	-0.758681	-0.084406
C	-1.010380	0.115930	0.017219
C	0.885010	1.651118	-0.406736
C	-0.528589	1.391032	-0.305366
C	-2.420906	-0.283692	-0.157028
C	3.155055	0.636744	-0.366048
C	1.974943	-1.890046	0.019605
C	3.355467	-1.731730	-0.066441
C	3.954945	-0.483870	-0.256405
C	-2.716946	-1.498966	-0.773912
C	-3.454208	0.540799	0.291675
C	-4.040124	-1.880369	-0.951654
C	-4.773127	0.148366	0.115583
C	-5.069516	-1.058791	-0.508554
H	1.533584	-2.862543	0.176055
H	5.029981	-0.383621	-0.324215

H	-1.917844	-2.139442	-1.117698
H	-3.228255	1.473040	0.785882
H	-4.264832	-2.820808	-1.436135
H	-5.571182	0.786079	0.470558
H	-6.099665	-1.358965	-0.645665
H	-0.773952	3.206631	-0.590122
H	3.002120	2.498898	-0.631380
H	5.036573	-2.636871	-0.025439
O	-1.076836	0.449906	1.965903
O	0.133048	0.095947	2.491510
H	0.569298	0.936256	2.694451

SCF Energy: -1104.86112180
Sum of electronic and zero-point Energies= -1104.620340
Sum of electronic and thermal Energies= -1104.602075
Sum of electronic and thermal Enthalpies= -1104.601131
Sum of electronic and thermal Free Energies= -1104.667241

==> ./AOX-I/pet/RAF/C8 <==

O	-0.343904	-0.526689	-0.121227
O	-1.869542	2.728507	0.128224
O	3.252720	2.629260	-0.029217
O	0.695797	3.347216	0.130502
O	4.088006	-2.043461	-0.509801
C	1.402240	1.087449	-0.040150
C	0.959614	-0.212174	-0.119633
C	-1.314922	0.415627	-0.031510
C	0.418023	2.136364	0.059980
C	-0.966816	1.727998	0.059913
C	-2.665931	-0.154781	-0.074367
C	2.794524	1.370438	-0.109969
C	1.853429	-1.392158	-0.189394
C	3.270569	-0.970055	-0.357770
C	3.708655	0.316337	-0.278354
C	-2.846620	-1.446695	-0.582771
C	-3.774848	0.557240	0.395563
C	-4.113410	-2.007330	-0.631566
C	-5.038239	-0.015514	0.347756
C	-5.213527	-1.294004	-0.167632
H	-1.997635	-2.009524	-0.941525
H	-3.652037	1.547015	0.804010
H	-4.240756	-3.004048	-1.031439
H	-5.887707	0.541588	0.718754
H	-6.201027	-1.733781	-0.204956
H	-1.350406	3.549064	0.157569
H	2.479858	3.223360	0.056851
H	5.010634	-1.764051	-0.545952
H	4.763793	0.548123	-0.353942
H	1.538288	-2.087472	-0.972005
O	1.711900	-2.126198	1.066922
O	1.939401	-3.504291	0.813672
H	2.905519	-3.557485	0.799446

SCF Energy: -1104.86542540
Sum of electronic and zero-point Energies= -1104.622337
Sum of electronic and thermal Energies= -1104.604061
Sum of electronic and thermal Enthalpies= -1104.603117
Sum of electronic and thermal Free Energies= -1104.669631

==> ./AOX-I/pet/RAF/C5p <==

O	0.459961	-0.893522	-0.071203
O	-0.692809	2.512259	-0.457183
O	4.347035	1.878455	0.087069
O	1.920393	2.866785	-0.228317
O	4.670385	-2.834494	0.478192
C	2.362738	0.548998	0.002498
C	1.797989	-0.731186	0.044157
C	-0.384460	0.163466	-0.229476
C	1.514814	1.690674	-0.164570
C	0.093543	1.428857	-0.279402
C	-1.787365	-0.256479	-0.361192
C	3.768759	0.672694	0.125613
C	2.563213	-1.872709	0.203776
C	3.937395	-1.711858	0.323233
C	4.549265	-0.453836	0.286537
C	-2.064280	-1.546354	-0.893125
C	-2.804142	0.569491	0.022283
C	-3.399514	-1.970693	-1.053756
C	-4.234429	0.170975	-0.097403
C	-4.444214	-1.176145	-0.699701
H	-1.252956	-2.194538	-1.182849
H	-2.606692	1.540232	0.448137
H	-3.586080	-2.949170	-1.476047
H	-5.463835	-1.505175	-0.845499
H	-0.089012	3.272249	-0.487414
H	3.640292	2.547199	-0.035750
H	5.605158	-2.614454	0.554292
H	5.622210	-0.351669	0.381676
H	2.109438	-2.851565	0.236856
H	-4.791648	0.935847	-0.649486
O	-4.743787	0.224953	1.267223
O	-6.165079	0.207505	1.194748
H	-6.371551	-0.679858	1.515021

SCF Energy: -1104.86013900
Sum of electronic and zero-point Energies= -1104.618710

Sum of electronic and thermal Energies= -1104.600186
Sum of electronic and thermal Enthalpies= -1104.599242
Sum of electronic and thermal Free Energies= -1104.666493

==> ./AOX-I/pet/RAF/C2p <==

O	-0.027981	0.536709	0.294457
O	-0.707623	-2.973920	-0.251344
O	4.198615	-1.612866	-0.272614
O	1.919064	-2.938020	-0.364609
O	3.892832	3.061123	0.466813
C	2.053223	-0.597626	0.000720
C	1.320025	0.574089	0.220832
C	-0.744220	-0.616421	0.137026
C	1.360834	-1.839948	-0.157602
C	-0.080729	-1.794192	-0.080115
C	-2.164159	-0.406155	0.228761
C	3.465360	-0.513192	-0.062187
C	1.928604	1.806437	0.377912
C	3.315159	1.850712	0.311639
C	4.091581	0.706588	0.095305
C	-2.635734	1.022041	0.186945
C	-3.082779	-1.439621	0.320896
C	-4.093125	1.199109	0.457266
C	-4.443911	-1.184672	0.465435
C	-4.935987	0.149168	0.555522
H	-2.040858	1.648515	0.852658
H	-2.742689	-2.461663	0.311783
H	-4.454633	2.214747	0.544051
H	-5.131772	-2.014191	0.537444
H	-5.993335	0.311472	0.716962
H	-0.000229	-3.622803	-0.407756
H	3.582129	-2.370760	-0.362851
H	4.850529	2.983744	0.398286
H	5.171110	0.764003	0.047437
H	1.346888	2.701068	0.540777
O	-2.322345	1.460174	-1.168191
O	-2.390774	2.880649	-1.185749
H	-3.206284	3.037178	-1.679024

SCF Energy: -1104.86719936
Sum of electronic and zero-point Energies= -1104.625686
Sum of electronic and thermal Energies= -1104.607181
Sum of electronic and thermal Enthalpies= -1104.606237
Sum of electronic and thermal Free Energies= -1104.673324

==> ./AOX-I/pet/RAF/C7 <==

O	-0.512149	-0.647505	-0.043285
O	-2.183680	2.541463	0.032757
O	2.901194	2.723258	-0.081974
O	0.333391	3.301393	-0.001823
O	3.716508	-1.647967	-1.273224
C	1.153274	1.072719	-0.055397
C	0.789897	-0.273537	-0.073869
C	-1.518435	0.258352	0.001801
C	0.129733	2.069303	-0.006208
C	-1.233608	1.589975	0.018721
C	-2.845262	-0.362948	-0.005205
C	2.569195	1.420753	-0.088753
C	1.716584	-1.289626	-0.125013
C	3.177225	-0.980950	-0.149657
C	3.513913	0.467462	-0.125693
C	-2.995528	-1.643719	-0.549575
C	-3.957859	0.291114	0.534214
C	-4.241074	-2.251587	-0.565872
C	-5.198624	-0.329965	0.521388
C	-5.345603	-1.597102	-0.030661
H	1.406720	-2.322543	-0.143241
H	-2.140612	-2.156077	-0.966115
H	-3.852848	1.271150	0.971517
H	-4.349491	-3.237575	-0.996623
H	-6.052338	0.178641	0.947911
H	-6.316216	-2.074238	-0.041778
H	-1.706381	3.388620	0.013134
H	2.077571	3.252718	-0.052802
H	4.494142	-1.162744	-1.571994
H	4.562538	0.734339	-0.131842
O	3.808615	-1.512943	1.030976
O	3.609892	-2.918570	1.062552
H	4.203773	-3.226785	0.362691

SCF Energy: -1104.86061449
Sum of electronic and zero-point Energies= -1104.619373
Sum of electronic and thermal Energies= -1104.600901
Sum of electronic and thermal Enthalpies= -1104.599956
Sum of electronic and thermal Free Energies= -1104.667401

==> ./AOX-I/pet/RAF/C4p <==

O	-0.456406	-0.782378	0.100994
O	0.391792	2.738532	0.073876
O	-4.564518	1.642210	-0.171213
O	-2.215787	2.847124	-0.066898
O	-4.504077	-3.096704	-0.063045
C	-2.476428	0.489383	-0.033732
C	-1.805453	-0.737082	0.028748
C	0.330162	0.341804	0.117958

C	-1.718248	1.702442	-0.017946
C	-0.280884	1.575212	0.060710
C	1.722842	0.048348	0.198696
C	-3.890090	0.488405	-0.109826
C	-2.478845	-1.944415	0.019853
C	-3.866305	-1.907508	-0.055530
C	-4.580917	-0.707145	-0.120847
C	2.151472	-1.323755	0.252188
C	2.732400	1.069852	0.214305
C	3.453185	-1.658234	0.332738
C	4.042175	0.759526	0.303026
C	4.539969	-0.642428	0.398916
H	1.406804	-2.104022	0.224702
H	2.440306	2.104115	0.152015
H	3.753854	-2.696782	0.361499
H	4.781299	1.549440	0.321630
H	5.122740	-0.777288	1.317366
H	-0.289637	3.431945	0.023420
H	-3.912022	2.373790	-0.150831
H	-5.456595	-2.963497	-0.118550
H	-5.661506	-0.700983	-0.180379
H	-1.948069	-2.883002	0.068707
O	5.433341	-0.967018	-0.694987
O	6.651339	-0.264438	-0.476176
H	6.627143	0.401238	-1.175529

SCF Energy: -1104.86944448
Sum of electronic and zero-point Energies= -1104.627981
Sum of electronic and thermal Energies= -1104.609345
Sum of electronic and thermal Enthalpies= -1104.608401
Sum of electronic and thermal Free Energies= -1104.677191

==> ./AOX-I/pet/RAF/C3 <==

O	0.212655	1.034849	0.411859
O	1.357324	-2.316726	-0.341560
O	-3.598981	-1.379474	-1.200511
O	-1.130849	-2.418557	-1.152387
O	-4.008462	3.007184	0.521854
C	-1.664490	-0.265824	-0.339568
C	-1.122310	0.911280	0.205450
C	1.091055	0.031728	0.109165
C	-0.826974	-1.406815	-0.527280
C	0.538047	-1.353717	0.182652
C	2.443186	0.446984	-0.065829
C	-3.045995	-0.295500	-0.647680
C	-1.905398	2.003991	0.500745
C	-3.270879	1.921947	0.221929
C	-3.849625	0.790857	-0.354048
C	2.720581	1.824245	-0.229752
C	3.528882	-0.457581	-0.061016
C	4.019745	2.267104	-0.392527
C	4.822589	0.004951	-0.223247
C	5.080400	1.363386	-0.392409
H	-1.476576	2.906618	0.909109
H	-4.906725	0.751797	-0.580871
H	1.907602	2.534323	-0.235507
H	3.352611	-1.510390	0.080088
H	4.208829	3.324297	-0.521806
H	5.640771	-0.702515	-0.211288
H	6.095319	1.714146	-0.519207
H	0.778610	-3.001503	-0.711158
H	-2.896616	-2.031605	-1.380879
H	-4.930937	2.862774	0.282779
O	0.317684	-1.544308	1.593746
O	-0.385969	-2.771442	1.772829
H	-1.232980	-2.462350	2.122593

SCF Energy: -1104.88225392
Sum of electronic and zero-point Energies= -1104.640010
Sum of electronic and thermal Energies= -1104.621691
Sum of electronic and thermal Enthalpies= -1104.620747
Sum of electronic and thermal Free Energies= -1104.687381

==> ./AOX-I/pet/RAF/C3/TS <==

O	0.173903	1.119386	0.153344
O	1.349307	-2.128002	-0.816761
O	-3.737666	-1.506197	-0.703136
O	-1.282743	-2.506856	-0.867066
O	-4.052010	3.057171	0.520268
C	-1.742002	-0.259226	-0.278872
C	-1.175343	0.972157	0.071779
C	1.042169	0.120707	-0.130502
C	-0.891504	-1.382447	-0.523872
C	0.553793	-1.182185	-0.307393
C	2.430417	0.535979	-0.130561
C	-3.150678	-0.352353	-0.367729
C	-1.939662	2.086204	0.343708
C	-3.323784	1.955572	0.252701
C	-3.937223	0.752873	-0.101023
C	2.737803	1.894625	-0.308007
C	3.472425	-0.382200	0.070167
C	4.056076	2.317586	-0.303482
C	4.787407	0.055587	0.081010
C	5.085467	1.400723	-0.110361
H	-1.484721	3.026960	0.614634
H	-5.014323	0.672549	-0.166157
H	1.942334	2.609455	-0.458980
H	3.251037	-1.423991	0.236184

H	4.282054	3.364833	-0.450855
H	5.583115	-0.657937	0.245735
H	6.114257	1.734300	-0.104717
H	0.815894	-2.936603	-0.880869
H	-3.037856	-2.169608	-0.864755
H	-4.992541	2.866231	0.433808
O	0.346244	-1.762281	1.538401
O	0.340994	-3.137452	1.480313
H	-0.595644	-3.373959	1.407706

SCF Energy: -1104.86174793
Sum of electronic and zero-point Energies= -1104.621000
Sum of electronic and thermal Energies= -1104.602694
Sum of electronic and thermal Enthalpies= -1104.601750
Sum of electronic and thermal Free Energies= -1104.668034

==> ./AOX-I/pet/RAF/C8a <==

O	-0.259765	-0.693143	-0.336269
O	-1.476178	2.648607	0.421567
O	3.550912	2.085216	-0.287391
O	1.154340	3.051716	0.226202
O	3.926921	-2.589752	-0.988460
C	1.629431	0.748147	0.019213
C	1.039001	-0.601398	0.203930
C	-1.127750	0.344396	-0.099398
C	0.759340	1.868992	0.164748
C	-0.666815	1.585283	0.183560
C	-2.530633	-0.066420	-0.217712
C	2.968525	0.893179	-0.240016
C	1.863258	-1.710619	-0.350367
C	3.198285	-1.507852	-0.617549
C	3.782623	-0.242201	-0.523531
C	-2.858315	-1.411452	-0.011317
C	-3.542822	0.842633	-0.545919
C	-4.174513	-1.835110	-0.119547
C	-4.855486	0.407928	-0.661134
C	-5.176820	-0.927789	-0.445329
H	-2.078598	-2.113776	0.244031
H	-3.302689	1.879690	-0.718011
H	-4.417680	-2.875074	0.051621
H	-5.629853	1.116493	-0.922177
H	-6.202675	-1.259992	-0.532018
H	-0.885931	3.410543	0.526058
H	2.858979	2.755564	-0.077908
H	4.815193	-2.323497	-1.248318
H	4.834777	-0.089282	-0.722016
H	1.417020	-2.687770	-0.455008
O	0.942337	-0.725419	1.641381
O	0.364630	-1.986938	1.959037
H	1.122029	-2.461498	2.327408

SCF Energy: -1104.85750816
Sum of electronic and zero-point Energies= -1104.616284
Sum of electronic and thermal Energies= -1104.597900
Sum of electronic and thermal Enthalpies= -1104.596956
Sum of electronic and thermal Free Energies= -1104.663186

==> ./AOX-I/pet/RAF/C4 <==

O	-0.402293	-1.135794	0.328922
O	-1.389305	2.303003	-0.229941
O	3.764110	1.197878	-0.312527
O	1.248638	1.742774	1.426239
O	3.595569	-3.500813	0.042741
C	1.617956	0.120021	0.006441
C	0.936428	-1.096613	0.175110
C	-1.180429	-0.011996	0.118621
C	0.851647	1.394648	0.183978
C	-0.631659	1.202758	0.009215
C	-2.609837	-0.352150	0.026357
C	3.027151	0.087150	-0.148511
C	1.596588	-2.307933	0.181261
C	2.979652	-2.303850	0.029393
C	3.696636	-1.120558	-0.146861
C	-2.980354	-1.615546	-0.444924
C	-3.605522	0.547137	0.417776
C	-4.320271	-1.964621	-0.538388
C	-4.943885	0.189320	0.325799
C	-5.307134	-1.063543	-0.154789
H	1.051522	-3.233014	0.293195
H	4.769512	-1.128154	-0.284820
H	-2.218115	-2.322070	-0.740142
H	-3.334376	1.518612	0.800078
H	-4.592850	-2.943154	-0.910386
H	-5.704834	0.892344	0.636965
H	-6.351253	-1.337354	-0.225586
H	-0.819496	3.079757	-0.298651
H	3.178456	1.945421	-0.494113
H	4.546080	-3.389971	-0.071948
O	1.354286	2.348756	-0.796595
O	0.974067	3.652345	-0.375330
H	1.552978	3.827040	0.384474

SCF Energy: -1104.82477932
Sum of electronic and zero-point Energies= -1104.582547
Sum of electronic and thermal Energies= -1104.564367

Sum of electronic and thermal Enthalpies= -1104.563423
Sum of electronic and thermal Free Energies= -1104.629123

==> ./AOX-I/pet/RAF/C3p <==

O	0.201212	0.572560	-0.048148
O	-0.146268	-3.021709	0.139798
O	4.630445	-1.214853	0.065045
O	2.483392	-2.752732	0.114197
O	3.869213	3.458609	-0.140908
C	2.389352	-0.385938	0.006800
C	1.544747	0.729601	-0.042766
C	-0.382303	-0.656535	-0.000359
C	1.821873	-1.698903	0.056044
C	0.373618	-1.777772	0.054825
C	-1.851717	-0.584302	0.017230
C	3.790488	-0.174739	0.013135
C	2.030821	2.023699	-0.093490
C	3.409423	2.191057	-0.089105
C	4.294725	1.108668	-0.033879
C	-2.456234	0.531023	0.524020
C	-2.628701	-1.654835	-0.501215
C	-3.936128	0.682303	0.567221
C	-4.037882	-1.579352	-0.467597
C	-4.681426	-0.498491	0.045902
H	-1.872180	1.354115	0.907832
H	-2.145089	-2.516204	-0.929511
H	-4.259137	0.930452	1.583905
H	-4.609713	-2.409352	-0.860760
H	-5.761539	-0.464339	0.083857
H	0.616433	-3.621309	0.184028
H	4.093358	-2.034397	0.097546
H	4.832510	3.464334	-0.135194
H	5.365314	1.265035	-0.025327
H	1.363776	2.871287	-0.136455
O	-4.209028	1.872892	-0.229700
O	-5.541780	2.283565	0.055446
H	-6.005154	2.040832	-0.756355

SCF Energy: -1104.86017268
Sum of electronic and zero-point Energies= -1104.618675
Sum of electronic and thermal Energies= -1104.600195
Sum of electronic and thermal Enthalpies= -1104.599251
Sum of electronic and thermal Free Energies= -1104.666213

==> ./AOX-I/pet/vIP <==

O	-0.214759	-0.875135	0.069170
O	-1.346650	2.553377	-0.137720
O	3.708279	1.843965	-0.090211
O	1.277980	2.867897	-0.143060
O	4.000098	-2.881838	0.151896
C	1.707134	0.540315	-0.012099
C	1.129938	-0.732810	0.054026
C	-1.058992	0.193451	0.015596
C	0.862653	1.694939	-0.068765
C	-0.565637	1.453729	-0.054431
C	-2.470873	-0.207108	0.013493
C	3.119873	0.644185	-0.024776
C	1.890636	-1.887340	0.109329
C	3.272034	-1.746413	0.097046
C	3.895974	-0.495491	0.031091
C	-2.818101	-1.490659	-0.422711
C	-3.474664	0.663409	0.450306
C	-4.146285	-1.888764	-0.434117
C	-4.800586	0.253131	0.442718
C	-5.141602	-1.018822	-0.001970
H	1.428164	-2.861185	0.161471
H	4.974436	-0.408860	0.022714
H	-2.049746	-2.171410	-0.758345
H	-3.220037	1.650310	0.802439
H	-4.403975	-2.880253	-0.780930
H	-5.568747	0.931197	0.788846
H	-6.176871	-1.332019	-0.009583
H	-0.733972	3.303838	-0.204875
H	3.001267	2.523027	-0.128269
H	4.940886	-2.675283	0.137530

SCF Energy: -953.692288893

==> ./AOX-I/pet/PA/C7 <==

O	-0.204314	-0.898865	0.062433
O	-1.247894	2.567183	-0.102401
O	3.763636	1.787438	-0.080494
O	1.338640	2.840960	-0.110120
O	4.035677	-2.916850	0.124834
C	1.746752	0.502522	-0.009305
C	1.158033	-0.785573	0.047417
C	-1.030733	0.188235	0.019893
C	0.934021	1.647275	-0.050007
C	-0.510419	1.432775	-0.035660
C	-2.450789	-0.188523	0.012794
C	3.177345	0.570035	-0.023329
C	1.892457	-1.936175	0.090507
C	3.334314	-1.881768	0.081569
C	3.933116	-0.563188	0.018940
C	-2.811557	-1.501016	-0.315765
C	-3.458154	0.727902	0.337977
C	-4.145573	-1.881965	-0.330662
C	-4.790225	0.336657	0.324931
C	-5.141579	-0.965723	-0.011131

H	1.402466	-2.898585	0.135758
H	5.012565	-0.491502	0.007145
H	-2.043986	-2.219191	-0.562514
H	-3.200223	1.739433	0.607049
H	-4.406107	-2.898818	-0.592537
H	-5.556347	1.055501	0.583421
H	-6.181209	-1.264643	-0.021512
H	-0.580983	3.275566	-0.152650
H	3.045806	2.454490	-0.107174

SCF Energy: -953.459161823
Sum of electronic and zero-point Energies= -953.247723
Sum of electronic and thermal Energies= -953.232533
Sum of electronic and thermal Enthalpies= -953.231589
Sum of electronic and thermal Free Energies= -953.290893

==> ./AOX-I/pet/PA/C3 <==

O	-0.217686	-0.841537	-0.000052
O	-1.335506	2.609881	0.000026
O	3.689270	1.850957	-0.000292
O	1.391909	2.877471	0.000394
O	3.982290	-2.891933	0.000168
C	1.691710	0.557658	-0.000058
C	1.106295	-0.711948	-0.000053
C	-1.071399	0.257689	-0.000050
C	0.873056	1.743921	-0.000086
C	-0.610083	1.578089	-0.000036
C	-2.464801	-0.162058	-0.000032
C	3.110476	0.647175	-0.000067
C	1.872702	-1.878511	0.000016
C	3.248700	-1.747717	0.000087
C	3.883362	-0.498025	0.000055
C	-2.797995	-1.531463	-0.000102
C	-3.524081	0.766866	0.000065
C	-4.120465	-1.947903	-0.000081
C	-4.844206	0.334869	0.000089
C	-5.158126	-1.019809	0.000014
H	1.402886	-2.850680	0.000030
H	4.962583	-0.417519	0.000050
H	-2.011720	-2.270905	-0.000172
H	-3.283593	1.817386	0.000118
H	-4.341673	-3.007817	-0.000139
H	-5.637368	1.072060	0.000169
H	-6.189343	-1.347246	0.000029
H	2.925789	2.506643	-0.000754
H	4.919336	-2.673634	0.000157

SCF Energy: -953.446681273
Sum of electronic and zero-point Energies= -953.236769
Sum of electronic and thermal Energies= -953.221495
Sum of electronic and thermal Enthalpies= -953.220551
Sum of electronic and thermal Free Energies= -953.279689

==> ./AOX-I/pet/PA/C5 <==

O	-0.183951	-0.824344	-0.000124
O	-1.314286	2.601978	0.000205
O	3.831133	1.826998	0.000098
O	1.182170	2.968488	0.000115
O	3.976660	-2.903119	-0.000063
C	1.760848	0.627861	-0.000039
C	1.169523	-0.655467	-0.000089
C	-1.054368	0.229002	-0.000031
C	0.892924	1.763393	0.000078
C	-0.561668	1.481128	0.000072
C	-2.458933	-0.202468	-0.000015
C	3.227591	0.736751	-0.000079
C	1.890963	-1.832377	-0.000114
C	3.287255	-1.724495	-0.000066
C	3.937177	-0.513712	-0.000035
C	-2.758808	-1.571366	0.000345
C	-3.518602	0.715084	-0.000375
C	-4.075967	-2.007919	0.000366
C	-4.833332	0.268451	-0.000364
C	-5.121375	-1.091426	0.000010
H	1.397618	-2.791870	-0.000143
H	5.020201	-0.469411	0.000012
H	-1.956027	-2.292644	0.000616
H	-3.314564	1.772879	-0.000672
H	-4.284175	-3.069713	0.000658
H	-5.637375	0.992550	-0.000655
H	-6.147878	-1.432895	0.000021
H	-0.630023	3.306022	0.000273
H	4.919050	-2.709571	0.000055

SCF Energy: -953.441682500
Sum of electronic and zero-point Energies= -953.231254
Sum of electronic and thermal Energies= -953.216624
Sum of electronic and thermal Enthalpies= -953.215680
Sum of electronic and thermal Free Energies= -953.272904

==> ./AOX-I/water <==

O	-0.214839	-0.882227	0.077729
O	-1.377255	2.536238	-0.179317

O	3.718376	1.846488	-0.106162
O	1.275382	2.870259	-0.181785
O	4.008839	-2.882371	0.198778
C	1.704816	0.542743	-0.018595
C	1.132833	-0.732628	0.061200
C	-1.055508	0.187947	0.012807
C	0.856685	1.695330	-0.091967
C	-0.567693	1.447712	-0.073973
C	-2.468677	-0.207498	0.014783
C	3.117843	0.639167	-0.028222
C	1.892282	-1.886779	0.133086
C	3.272290	-1.745560	0.123742
C	3.895881	-0.495548	0.042246
C	-2.839128	-1.415812	-0.583949
C	-3.441729	0.592813	0.619000
C	-4.169832	-1.807886	-0.591126
C	-4.770086	0.188642	0.614422
C	-5.137850	-1.007181	0.007076
H	1.425629	-2.858203	0.196578
H	4.974684	-0.417024	0.033744
H	-2.088387	-2.037703	-1.050573
H	-3.160324	1.514009	1.106081
H	-4.450892	-2.738465	-1.064674
H	-5.517466	0.807470	1.091563
H	-6.174223	-1.316083	0.002859
H	-0.792527	3.305806	-0.268934
H	3.017051	2.529938	-0.156361
H	4.949678	-2.669628	0.192590

SCF Energy: -953.939655662
Sum of electronic and zero-point Energies= -953.717083
Sum of electronic and thermal Energies= -953.701284
Sum of electronic and thermal Enthalpies= -953.700340
Sum of electronic and thermal Free Energies= -953.760451

==> ./AOX-I/water/RAF/C5p <==

O	0.440920	-0.879095	-0.069704
O	-0.682786	2.539946	-0.466010
O	4.383021	1.840571	0.092577
O	1.955056	2.869406	-0.212621
O	4.627597	-2.882248	0.492847
C	2.363929	0.543031	0.010515
C	1.783853	-0.731077	0.048748
C	-0.384503	0.191720	-0.233350
C	1.528961	1.696229	-0.153852
C	0.107806	1.450584	-0.275580
C	-1.791546	-0.206568	-0.384695
C	3.772268	0.636651	0.132671
C	2.529369	-1.885225	0.209451
C	3.904247	-1.746148	0.332381
C	4.536022	-0.498282	0.293479
C	-2.080469	-1.425039	-1.057936
C	-2.793602	0.573109	0.113240
C	-3.420577	-1.824020	-1.242879
C	-4.226287	0.194621	-0.028054
C	-4.453932	-1.070414	-0.781073
H	-1.274906	-2.033352	-1.437808
H	-2.580537	1.484675	0.650112
H	-3.620938	-2.746069	-1.771635
H	-5.478773	-1.377674	-0.937371
H	-0.086910	3.305130	-0.513539
H	3.694989	2.526611	-0.034775
H	5.565402	-2.670814	0.575143
H	5.610872	-0.421764	0.387750
H	2.056179	-2.855121	0.238352
H	-4.796415	1.021443	-0.462464
O	-4.712500	0.072934	1.348622
O	-6.133298	0.090836	1.319650
H	-6.366262	-0.846893	1.364958

SCF Energy: -1104.86390750
Sum of electronic and zero-point Energies= -1104.623850
Sum of electronic and thermal Energies= -1104.605190
Sum of electronic and thermal Enthalpies= -1104.604246
Sum of electronic and thermal Free Energies= -1104.672095

==> ./AOX-I/water/RAF/C7 <==

O	-0.511203	-0.652502	-0.055138
O	-2.195249	2.530525	0.047245
O	2.919879	2.716840	-0.127577
O	0.350956	3.302153	0.009206
O	3.732283	-1.703119	-1.259508
C	1.156363	1.068138	-0.064219
C	0.792579	-0.278362	-0.094789
C	-1.510755	0.258109	0.003609
C	0.135220	2.066850	-0.000226
C	-1.224926	1.589446	0.029826
C	-2.840681	-0.355591	-0.005535
C	2.573763	1.403160	-0.124634
C	1.712361	-1.298396	-0.170905
C	3.173392	-0.997400	-0.178694
C	3.516029	0.451567	-0.185560
C	-3.019542	-1.577469	-0.663819
C	-3.922114	0.246803	0.644294
C	-4.269147	-2.178218	-0.683559
C	-5.167015	-0.366893	0.626152
C	-5.345132	-1.574700	-0.039775
H	1.392580	-2.327902	-0.213062

H	-2.185430	-2.045703	-1.166432
H	-3.787937	1.175489	1.176772
H	-4.403192	-3.117329	-1.202343
H	-5.997981	0.098551	1.137813
H	-6.318262	-2.046204	-0.054328
H	-1.747100	3.392555	0.026261
H	2.098576	3.250283	-0.083019
H	4.626692	-1.374701	-1.418846
H	4.565355	0.710580	-0.236472
O	3.770732	-1.473829	1.053857
O	3.548045	-2.870319	1.183541
H	4.270773	-3.255873	0.667633

SCF Energy: -1104.86373761
Sum of electronic and zero-point Energies= -1104.623157
Sum of electronic and thermal Energies= -1104.604576
Sum of electronic and thermal Enthalpies= -1104.603632
Sum of electronic and thermal Free Energies= -1104.670803

==> ./AOX-I/water/RAF/C2p <==

O	-0.039561	0.529446	0.298726
O	-0.754966	-2.973024	-0.270752
O	4.193822	-1.633750	-0.270054
O	1.897334	-2.954551	-0.378756
O	3.889586	3.049008	0.479165
C	2.037556	-0.617214	0.007181
C	1.311449	0.558141	0.229542
C	-0.760499	-0.622570	0.142188
C	1.336506	-1.855043	-0.161542
C	-0.102825	-1.801445	-0.084619
C	-2.179798	-0.401151	0.233715
C	3.449040	-0.527181	-0.054855
C	1.920806	1.789605	0.391153
C	3.306303	1.833270	0.322804
C	4.079524	0.687758	0.102773
C	-2.635233	1.030218	0.168464
C	-3.106244	-1.421228	0.371226
C	-4.098235	1.228698	0.377966
C	-4.466655	-1.149088	0.502477
C	-4.950913	0.190249	0.512401
H	-2.058965	1.651892	0.854982
H	-2.776781	-2.445942	0.412011
H	-4.450770	2.251260	0.389254
H	-5.160114	-1.968975	0.615029
H	-6.010487	0.367086	0.637277
H	-0.072301	-3.642372	-0.445270
H	3.581767	-2.393582	-0.366488
H	4.848849	2.978494	0.402034
H	5.158195	0.751151	0.053396
H	1.336891	2.681867	0.560728
O	-2.265575	1.474396	-1.176383
O	-2.194484	2.892516	-1.174397
H	-3.050231	3.158681	-1.538586

SCF Energy: -1104.87117380
Sum of electronic and zero-point Energies= -1104.630691
Sum of electronic and thermal Energies= -1104.612204
Sum of electronic and thermal Enthalpies= -1104.611260
Sum of electronic and thermal Free Energies= -1104.677954

==> ./AOX-I/water/RAF/C4p <==

O	-0.453467	-0.773090	0.092859
O	0.394214	2.749791	0.093506
O	-4.591222	1.628263	-0.164168
O	-2.236058	2.852322	-0.064130
O	-4.488814	-3.118966	-0.065266
C	-2.480566	0.492104	-0.034948
C	-1.804580	-0.731872	0.025295
C	0.327932	0.356071	0.112460
C	-1.726117	1.708393	-0.018011
C	-0.289988	1.586959	0.063929
C	1.721580	0.064783	0.189374
C	-3.893816	0.472745	-0.107010
C	-2.467362	-1.945434	0.016324
C	-3.853738	-1.919866	-0.056720
C	-4.576976	-0.724304	-0.118804
C	2.147020	-1.307951	0.266715
C	2.735091	1.082473	0.168724
C	3.448162	-1.644677	0.350415
C	4.044594	0.770167	0.259151
C	4.534979	-0.629324	0.400493
H	1.402015	-2.088342	0.259376
H	2.450396	2.115651	0.071082
H	3.745239	-2.683122	0.405403
H	4.790311	1.553722	0.236495
H	5.113537	-0.744200	1.323195
H	-0.268506	3.460870	0.051967
H	-3.951580	2.370409	-0.141853
H	-5.444381	-2.994991	-0.116622
H	-5.657168	-0.735081	-0.174715
H	-1.924151	-2.877256	0.063269
O	5.440956	-0.979259	-0.682533
O	6.674669	-0.309708	-0.462470
H	6.624760	0.445911	-1.064299

SCF Energy: -1104.87244088
Sum of electronic and zero-point Energies= -1104.631953

Sum of electronic and thermal Energies= -1104.613407
Sum of electronic and thermal Enthalpies= -1104.612463
Sum of electronic and thermal Free Energies= -1104.680233

==> ./AOX-I/water/RAF/C8a <==

O	-0.245405	-0.740891	-0.223849
O	-1.506378	2.654855	0.137956
O	3.551221	2.044138	-0.555316
O	1.127423	3.060597	-0.112515
O	3.952777	-2.677932	-0.654845
C	1.632258	0.752254	-0.034209
C	1.054139	-0.573064	0.304765
C	-1.125227	0.310346	-0.087800
C	0.750970	1.871838	-0.008949
C	-0.673376	1.575757	0.062515
C	-2.525780	-0.107580	-0.211704
C	2.966841	0.863636	-0.342227
C	1.882380	-1.738579	-0.102639
C	3.208798	-1.561437	-0.416075
C	3.782166	-0.290778	-0.501251
C	-2.829981	-1.259705	-0.946847
C	-3.559756	0.608302	0.400833
C	-4.147162	-1.671829	-1.084761
C	-4.874345	0.181808	0.266734
C	-5.173181	-0.952993	-0.479133
H	-2.037375	-1.823048	-1.417216
H	-3.337750	1.480219	0.995706
H	-4.372553	-2.556853	-1.663849
H	-5.665776	0.735910	0.752477
H	-6.199031	-1.279119	-0.583584
H	-0.938847	3.440847	0.096777
H	2.875435	2.745779	-0.427734
H	4.848200	-2.433594	-0.918230
H	4.829001	-0.166645	-0.740665
H	1.445799	-2.724702	-0.054429
O	0.943655	-0.525379	1.750807
O	0.333491	-1.720309	2.218966
H	1.086171	-2.227289	2.555603

SCF Energy: -1104.86154543
Sum of electronic and zero-point Energies= -1104.621099
Sum of electronic and thermal Energies= -1104.602714
Sum of electronic and thermal Enthalpies= -1104.601770
Sum of electronic and thermal Free Energies= -1104.667832

==> ./AOX-I/water/RAF/C3/TS <==

O	0.171696	1.114011	0.155316
O	1.357288	-2.136872	-0.799691
O	-3.752496	-1.510662	-0.740196
O	-1.266050	-2.501791	-0.934518
O	-4.065086	3.037374	0.563570
C	-1.744872	-0.269306	-0.294772
C	-1.182860	0.960310	0.071963
C	1.040965	0.119808	-0.133810
C	-0.888809	-1.384495	-0.555853
C	0.553811	-1.181543	-0.317565
C	2.428984	0.535467	-0.134406
C	-3.153418	-0.356390	-0.384341
C	-1.945750	2.069548	0.361704
C	-3.330076	1.938410	0.273335
C	-3.941363	0.740611	-0.099377
C	2.734141	1.888152	-0.354981
C	3.468965	-0.375475	0.104874
C	4.052495	2.311992	-0.356008
C	4.784085	0.063497	0.109323
C	5.081265	1.402284	-0.125867
H	-1.486984	3.005947	0.641397
H	-5.018525	0.667401	-0.164064
H	1.938344	2.595032	-0.538565
H	3.249734	-1.411133	0.310300
H	4.279042	3.353380	-0.537918
H	5.579638	-0.642368	0.303509
H	6.109547	1.736715	-0.125525
H	0.798466	-2.903311	-1.014580
H	-3.064871	-2.180715	-0.918360
H	-5.008150	2.848450	0.478283
O	0.330884	-1.714596	1.565354
O	0.398249	-3.094252	1.578774
H	-0.517491	-3.392314	1.471347

SCF Energy: -1104.86512457
Sum of electronic and zero-point Energies= -1104.625536
Sum of electronic and thermal Energies= -1104.607118
Sum of electronic and thermal Enthalpies= -1104.606173
Sum of electronic and thermal Free Energies= -1104.672894

==> ./AOX-I/water/RAF/C3 <==

O	0.209713	1.043062	0.400370
O	1.369586	-2.296113	-0.412770
O	-3.632214	-1.384938	-1.166790
O	-1.141963	-2.420066	-1.151096
O	-4.018748	3.010775	0.551488
C	-1.674727	-0.267704	-0.333297
C	-1.131740	0.913799	0.201022
C	1.089270	0.039113	0.098808

C	-0.835374	-1.403088	-0.532380
C	0.543435	-1.347587	0.150728
C	2.444007	0.455511	-0.064148
C	-3.061325	-0.295678	-0.618636
C	-1.908941	2.007961	0.501156
C	-3.278311	1.922803	0.243739
C	-3.862399	0.787914	-0.317753
C	2.714739	1.822081	-0.303554
C	3.533908	-0.437488	0.031073
C	4.015782	2.264967	-0.458447
C	4.829591	0.023935	-0.124443
C	5.082059	1.371496	-0.373171
H	-1.471824	2.911127	0.899631
H	-4.923554	0.754209	-0.522954
H	1.896408	2.522250	-0.380678
H	3.363263	-1.479308	0.245221
H	4.201308	3.313063	-0.650014
H	5.651918	-0.673633	-0.041871
H	6.097496	1.721967	-0.494815
H	0.795088	-2.958106	-0.830814
H	-2.941926	-2.045087	-1.361493
H	-4.948211	2.862772	0.336091
O	0.359109	-1.582578	1.562124
O	-0.334193	-2.814460	1.737581
H	-1.210130	-2.522928	2.030258

SCF Energy: -1104.88528290
Sum of electronic and zero-point Energies= -1104.643930
Sum of electronic and thermal Energies= -1104.625614
Sum of electronic and thermal Enthalpies= -1104.624670
Sum of electronic and thermal Free Energies= -1104.691083

==> ./AOX-I/water/RAF/C4 <==

O	0.401665	-1.171308	-0.256203
O	1.418816	2.290952	0.157764
O	-3.761742	1.234633	0.216748
O	-1.264033	1.784009	-1.381724
O	-3.645146	-3.478771	-0.075596
C	-1.614935	0.118473	-0.015041
C	-0.946403	-1.109453	-0.150297
C	1.181757	-0.034460	-0.107790
C	-0.842305	1.390951	-0.143302
C	0.639942	1.183454	-0.024452
C	2.614443	-0.365390	-0.033729
C	-3.029759	0.103909	0.095043
C	-1.621670	-2.311550	-0.163550
C	-3.009009	-2.286050	-0.054926
C	-3.716488	-1.091888	0.083921
C	3.010069	-1.536878	0.618482
C	3.582652	0.456202	-0.615396
C	4.355328	-1.869621	0.702746
C	4.926878	0.116448	-0.529257
C	5.317697	-1.043148	0.131767
H	-1.084656	-3.244611	-0.246715
H	-4.793313	-1.094451	0.183214
H	2.264837	-2.180836	1.064129
H	3.285546	1.349020	-1.144836
H	4.651250	-2.774591	1.215720
H	5.668973	0.756483	-0.986738
H	6.365287	-1.303861	0.197324
H	0.857976	3.080676	0.173771
H	-3.182639	1.990838	0.392953
H	-4.599006	-3.356298	0.009031
O	-1.315983	2.294770	0.881375
O	-0.944659	3.616816	0.518455
H	-1.634775	3.882816	-0.109727

SCF Energy: -1104.82947665
Sum of electronic and zero-point Energies= -1104.588999
Sum of electronic and thermal Energies= -1104.570512
Sum of electronic and thermal Enthalpies= -1104.569568
Sum of electronic and thermal Free Energies= -1104.636130

==> ./AOX-I/water/RAF/C3p <==

O	0.205469	0.581018	-0.111718
O	-0.193315	-2.999339	0.223206
O	4.637100	-1.222984	0.157278
O	2.473946	-2.754108	0.228364
O	3.894313	3.450497	-0.230165
C	2.388599	-0.392034	0.021446
C	1.554066	0.727828	-0.083464
C	-0.379047	-0.645618	-0.027628
C	1.811847	-1.700659	0.113915
C	0.366233	-1.767681	0.087838
C	-1.848669	-0.577464	-0.043562
C	3.788743	-0.177879	0.047963
C	2.045034	2.018149	-0.170180
C	3.422637	2.181228	-0.145388
C	4.301488	1.097654	-0.036145
C	-2.469662	0.476038	0.562986
C	-2.599892	-1.588528	-0.698183
C	-3.950911	0.607851	0.587710
C	-4.010026	-1.519135	-0.705433
C	-4.673602	-0.501978	-0.095124
H	-1.899576	1.248792	1.058231
H	-2.094343	-2.390820	-1.209819
H	-4.300846	0.732857	1.617002
H	-4.565119	-2.296521	-1.212683

H	-5.754324	-0.462327	-0.098498
H	0.539339	-3.628443	0.318993
H	4.102492	-2.042475	0.214081
H	4.858911	3.453625	-0.199807
H	5.371093	1.257770	-0.015718
H	1.378514	2.863471	-0.252497
O	-4.226346	1.887951	-0.068846
O	-5.547070	2.286925	0.272881
H	-6.069943	1.997756	-0.487782

SCF Energy: -1104.86380347
Sum of electronic and zero-point Energies= -1104.624148
Sum of electronic and thermal Energies= -1104.605414
Sum of electronic and thermal Enthalpies= -1104.604469
Sum of electronic and thermal Free Energies= -1104.672272

==> ./AOX-I/water/RAF/C4a/TS <==

O	-0.489483	0.997921	-0.004307
O	-1.476777	-2.428552	-0.635075
O	3.586455	-1.522282	-0.454385
O	1.199176	-2.630870	-0.581939
O	3.588614	3.223762	-0.532482
C	1.527074	-0.349970	0.060306
C	0.866829	0.932957	-0.102176
C	-1.270530	-0.100441	-0.177617
C	0.705529	-1.513587	-0.364422
C	-0.715116	-1.317398	-0.414576
C	-2.698484	0.212504	-0.069130
C	2.945088	-0.346923	-0.329999
C	1.553466	2.103017	-0.246351
C	2.944011	2.046012	-0.403654
C	3.631292	0.824627	-0.482674
C	-3.094701	1.280279	0.745032
C	-3.664564	-0.515715	-0.770743
C	-4.438252	1.600506	0.867612
C	-5.006416	-0.180599	-0.649222
C	-5.397506	0.871378	0.171553
H	1.033735	3.049617	-0.279309
H	4.691450	0.805785	-0.698008
H	-2.352530	1.847101	1.288401
H	-3.370355	-1.323016	-1.422403
H	-4.736308	2.420051	1.506883
H	-5.747140	-0.741568	-1.202218
H	-6.444638	1.124817	0.265782
H	-0.867523	-3.180688	-0.689904
H	2.921430	-2.242688	-0.454286
H	4.537066	3.082270	-0.645561
O	1.516388	-0.492182	1.721162
O	2.074992	-1.722804	2.122297
H	3.013572	-1.521511	2.244836

SCF Energy: -1104.84331264
Sum of electronic and zero-point Energies= -1104.604236
Sum of electronic and thermal Energies= -1104.585737
Sum of electronic and thermal Enthalpies= -1104.584793
Sum of electronic and thermal Free Energies= -1104.651528

==> ./AOX-I/water/RAF/C4a <==

O	-0.489858	0.994947	0.068282
O	-1.457654	-2.401879	-0.728195
O	3.610816	-1.519383	-0.389159
O	1.217279	-2.605081	-0.631304
O	3.573275	3.229530	-0.605791
C	1.538040	-0.359256	0.177518
C	0.870089	0.934518	-0.048172
C	-1.263445	-0.096511	-0.155742
C	0.716454	-1.504935	-0.358103
C	-0.699203	-1.302183	-0.437276
C	-2.694005	0.207069	-0.057811
C	2.960966	-0.342439	-0.274864
C	1.547108	2.098566	-0.250281
C	2.936222	2.050865	-0.424246
C	3.628286	0.826183	-0.479713
C	-3.106057	1.248213	0.782989
C	-3.647492	-0.499634	-0.798154
C	-4.451942	1.562872	0.892965
C	-4.991619	-0.169498	-0.689109
C	-5.398275	0.855389	0.157874
H	1.016710	3.037881	-0.317130
H	4.681911	0.806537	-0.726368
H	-2.374199	1.798949	1.356032
H	-3.341437	-1.285403	-1.470077
H	-4.762085	2.361447	1.552685
H	-5.721903	-0.713384	-1.272248
H	-6.447201	1.104882	0.242120
H	-0.847635	-3.151248	-0.803365
H	2.947648	-2.239053	-0.418040
H	4.519624	3.087706	-0.734364
O	1.502784	-0.485939	1.695772
O	2.007799	-1.749247	2.101152
H	2.942279	-1.567211	2.277442

SCF Energy: -1104.84407499
Sum of electronic and zero-point Energies= -1104.604108
Sum of electronic and thermal Energies= -1104.585372

Sum of electronic and thermal Enthalpies=
-1104.584428
Sum of electronic and thermal Free Energies=
-1104.651589

==> ./AOX-I/water/RAF/C6 <==

O	-0.745455	-0.820171	-0.055993
O	-2.110494	2.524921	-0.135407
O	3.027338	2.114174	-0.418711
O	0.519538	3.010400	-0.296007
O	3.564395	-2.639224	-0.376834
C	1.085433	0.709778	-0.192937
C	0.577436	-0.590292	-0.151386
C	-1.650568	0.193872	-0.027665
C	0.164267	1.813789	-0.203134
C	-1.235105	1.484103	-0.108050
C	-3.034163	-0.283809	0.060684
C	2.467582	0.896445	-0.284554
C	1.412476	-1.730212	-0.208615
C	2.752933	-1.560869	-0.279220
C	3.425669	-0.226329	-0.233707
C	-3.373891	-1.507242	-0.526097
C	-4.010105	0.453228	0.737447
C	-4.676638	-1.977415	-0.447833
C	-5.309996	-0.028193	0.816246
C	-5.647597	-1.239440	0.222148
H	0.975540	-2.718583	-0.209998
H	-2.621897	-2.080222	-1.049837
H	-3.751277	1.385433	1.215754
H	-4.933992	-2.919684	-0.911635
H	-6.058430	0.542226	1.348885
H	-6.662174	-1.608731	0.283841
H	-1.577969	3.329214	-0.242392
H	2.303303	2.776922	-0.412940
H	4.481292	-2.373381	-0.211323
H	4.180495	-0.135262	-1.021113
O	4.161157	-0.089383	1.033435
O	5.332220	-0.894831	0.949139
H	5.989125	-0.289503	0.573822

SCF Energy: -1104.86688246
Sum of electronic and zero-point Energies=
-1104.625847
Sum of electronic and thermal Energies=
-1104.607337
Sum of electronic and thermal Enthalpies=
-1104.606393
Sum of electronic and thermal Free Energies=
-1104.673197

==> ./AOX-I/water/RAF/C5/TS <==

O	-0.700393	0.944827	-0.160975
O	-1.658020	-2.538752	-0.100714
O	3.359757	-1.495950	-0.781464
O	1.007332	-2.712839	-0.316221
O	3.352193	3.247767	-0.528432
C	1.272080	-0.360713	-0.354461
C	0.639394	0.863130	-0.265002
C	-1.483633	-0.169567	-0.119175
C	0.510432	-1.565485	-0.292526
C	-0.922474	-1.403509	-0.181467
C	-2.907591	0.148488	0.026499
C	2.718689	-0.378292	-0.380222
C	1.334192	2.082528	-0.301125
C	2.710892	2.052234	-0.455565
C	3.408320	0.851276	-0.550619
C	-3.280013	1.315152	0.702770
C	-3.893110	-0.682790	-0.513360
C	-4.621785	1.635317	0.847856
C	-5.233376	-0.350770	-0.368513
C	-5.601246	0.803573	0.314028
H	0.797931	3.016401	-0.222527
H	4.481765	0.836524	-0.680686
H	-2.521320	1.961318	1.120615
H	-3.615269	-1.571494	-1.058616
H	-4.902609	2.534394	1.378846
H	-5.990568	-0.993812	-0.795464
H	-6.646731	1.056366	0.426471
H	-1.029867	-3.279248	-0.120412
H	2.744012	-2.249022	-0.666276
H	4.305362	3.113943	-0.595900
O	2.931483	-0.511892	1.508503
O	4.233612	-0.888415	1.758941
H	4.264960	-1.826769	1.522129

SCF Energy: -1104.85238548
Sum of electronic and zero-point Energies=
-1104.613254
Sum of electronic and thermal Energies=
-1104.594784
Sum of electronic and thermal Enthalpies=
-1104.593840
Sum of electronic and thermal Free Energies=
-1104.660636

==> ./AOX-I/water/RAF/C5 <==

O	-0.720579	0.939064	-0.113266
O	-1.651327	-2.546437	-0.052318
O	3.304548	-1.327074	-1.018705
O	1.010640	-2.704739	-0.208219
O	3.275740	3.322380	-0.493253
C	1.264982	-0.348467	-0.164301
C	0.618993	0.858689	-0.149641
C	-1.502268	-0.177299	-0.077165
C	0.514265	-1.558412	-0.167102
C	-0.926856	-1.405473	-0.108288

C	-2.931116	0.136144	0.022480
C	2.766197	-0.386379	-0.132341
C	1.298095	2.099080	-0.210651
C	2.692379	2.095135	-0.357377
C	3.412840	0.933547	-0.382830
C	-3.326535	1.315369	0.663175
C	-3.899231	-0.712764	-0.521470
C	-4.673105	1.630854	0.768913
C	-5.244465	-0.385910	-0.415226
C	-5.635220	0.781213	0.231870
H	0.738611	3.021626	-0.188799
H	4.484385	0.943086	-0.522304
H	-2.582140	1.975477	1.084966
H	-3.604409	-1.611826	-1.039990
H	-4.971020	2.539953	1.272878
H	-5.987601	-1.043597	-0.844582
H	-6.684482	1.029766	0.314561
H	-1.012957	-3.279381	-0.056140
H	2.784586	-2.142394	-0.912893
H	4.235610	3.232743	-0.532413
O	3.025997	-0.813472	1.233363
O	4.427148	-0.887760	1.440707
H	4.648353	-1.773097	1.117971

SCF Energy: -1104.87151717
Sum of electronic and zero-point Energies= -1104.630444
Sum of electronic and thermal Energies= -1104.611998
Sum of electronic and thermal Enthalpies= -1104.611054
Sum of electronic and thermal Free Energies= -1104.677474

==> ./AOX-I/water/RAF/C1p <==

O	-0.038663	-0.792517	0.219638
O	1.012522	2.650639	-0.120787
O	-4.054910	1.768336	-0.312562
O	-1.644102	2.884021	-0.304355
O	-4.184143	-2.948655	0.198522
C	-2.000733	0.552209	-0.052551
C	-1.388904	-0.695508	0.123801
C	0.753994	0.303672	0.143411
C	-1.194204	1.732055	-0.139801
C	0.240344	1.539138	-0.030335
C	2.233756	-0.024233	0.241739
C	-3.414432	0.591992	-0.143270
C	-2.107346	-1.873838	0.208769
C	-3.488811	-1.787795	0.114173
C	-4.152251	-0.567978	-0.060312
C	2.476492	-1.449819	0.621361
C	2.927204	0.386420	-1.019813
C	3.314721	-2.249393	-0.084201
C	3.733963	-0.465463	-1.705254
C	3.956137	-1.786451	-1.257810
H	1.985124	-1.800248	1.518372
H	2.763603	1.399180	-1.357888
H	3.492809	-3.261950	0.252163
H	4.218784	-0.123935	-2.609937
H	4.612905	-2.442001	-1.809114
H	0.407920	3.395162	-0.273012
H	-3.382053	2.478824	-0.357300
H	-5.130358	-2.778662	0.118272
H	-5.231038	-0.533480	-0.131685
H	-1.609133	-2.822016	0.343791
O	2.648647	0.837635	1.364704
O	4.055433	0.755674	1.507722
H	4.173601	0.018023	2.123823

SCF Energy: -1104.85769195
Sum of electronic and zero-point Energies= -1104.618174
Sum of electronic and thermal Energies= -1104.599611
Sum of electronic and thermal Enthalpies= -1104.598667
Sum of electronic and thermal Free Energies= -1104.665300

==> ./AOX-I/water/RAF/C6p <==

O	0.321141	-1.070739	-0.031254
O	-1.178495	2.185300	-0.558569
O	3.921052	2.086891	-0.141451
O	1.384443	2.809778	-0.406970
O	4.727381	-2.556126	0.441565
C	2.069277	0.560888	-0.095470
C	1.639653	-0.765679	0.022499
C	-0.650439	-0.121519	-0.208198
C	1.099549	1.596312	-0.284829
C	-0.286594	1.191943	-0.346214
C	-1.974850	-0.681788	-0.242114
C	3.458930	0.822721	-0.030094
C	2.519787	-1.817398	0.201421
C	3.873602	-1.516443	0.262496
C	4.355668	-0.208337	0.147882
C	-2.155420	-2.056551	-0.280479
C	-3.164586	0.231285	-0.173696
C	-3.423942	-2.623863	-0.325916
C	-4.482877	-0.460096	-0.285879
C	-4.590325	-1.804243	-0.339032
H	-1.292523	-2.704932	-0.296939
H	-3.524315	-3.698321	-0.361220
H	-5.563542	-2.272277	-0.396289
H	-0.663928	3.007554	-0.620373
H	3.150386	2.680606	-0.262258
H	5.638096	-2.239148	0.478230

H	5.417218	-0.005863	0.198316
H	2.164636	-2.832872	0.292301
H	-5.359176	0.173996	-0.297717
H	-3.093051	1.032179	-0.907413
O	-3.057326	0.874801	1.136124
O	-3.858396	2.046903	1.121554
H	-4.676801	1.761215	1.551108

SCF Energy: -1104.87152018
Sum of electronic and zero-point Energies= -1104.631435
Sum of electronic and thermal Energies= -1104.612830
Sum of electronic and thermal Enthalpies= -1104.611885
Sum of electronic and thermal Free Energies= -1104.679127

==> ./AOX-I/water/RAF/C2/TS <==

O	-0.157689	-0.928596	-0.017724
O	-1.363964	2.422908	-0.464540
O	3.740343	1.827451	-0.552122
O	1.277749	2.825397	-0.624307
O	4.093660	-2.861760	0.059123
C	1.747097	0.518695	-0.287231
C	1.193539	-0.755255	-0.091200
C	-1.012401	0.119938	-0.000484
C	0.887378	1.655792	-0.416792
C	-0.529398	1.394140	-0.318244
C	-2.423514	-0.279867	-0.161863
C	3.157109	0.626061	-0.363462
C	1.966404	-1.890712	0.019126
C	3.347303	-1.738699	-0.059620
C	3.952822	-0.494388	-0.248776
C	-2.719784	-1.482639	-0.804274
C	-3.456087	0.527119	0.321530
C	-4.043213	-1.869396	-0.970896
C	-4.775130	0.129024	0.155535
C	-5.071870	-1.065805	-0.492769
H	1.515983	-2.860012	0.171042
H	5.029227	-0.406803	-0.307825
H	-1.921383	-2.106518	-1.179106
H	-3.230904	1.448574	0.836727
H	-4.268670	-2.798792	-1.475441
H	-5.572256	0.752177	0.536801
H	-6.101590	-1.370111	-0.621889
H	-0.823134	3.217993	-0.614523
H	3.035208	2.502892	-0.629988
H	5.031979	-2.646051	-0.004607
O	-1.066618	0.475496	1.978873
O	0.143641	0.110085	2.499543
H	0.647036	0.934757	2.574572

SCF Energy: -1104.86598298
Sum of electronic and zero-point Energies= -1104.626843
Sum of electronic and thermal Energies= -1104.608412
Sum of electronic and thermal Enthalpies= -1104.607468
Sum of electronic and thermal Free Energies= -1104.673823

==> ./AOX-I/water/RAF/C2 <==

O	-0.079849	-0.915388	-0.233092
O	-1.400428	2.389516	0.073933
O	3.729327	2.018790	-0.211719
O	1.236943	2.937677	-0.306521
O	4.253227	-2.698553	-0.225431
C	1.785197	0.615829	-0.171083
C	1.275496	-0.692610	-0.175426
C	-0.931769	0.033811	0.370730
C	0.880214	1.742086	-0.164462
C	-0.494319	1.432099	0.043767
C	-2.348348	-0.256362	-0.067260
C	3.184095	0.783166	-0.197555
C	2.092314	-1.797556	-0.193066
C	3.473864	-1.589123	-0.209086
C	4.029305	-0.314155	-0.209015
C	-2.591815	-0.766579	-1.338238
C	-3.408465	0.060572	0.777364
C	-3.901011	-0.975971	-1.757518
C	-4.714001	-0.151083	0.353339
C	-4.962113	-0.670215	-0.913420
H	1.680208	-2.795650	-0.200698
H	5.101483	-0.173955	-0.225309
H	-1.767475	-0.999301	-1.997008
H	-3.214392	0.464187	1.761269
H	-4.088916	-1.376892	-2.744051
H	-5.536664	0.089007	1.012763
H	-5.979662	-0.834500	-1.240995
H	-0.955345	3.241030	-0.090242
H	3.001100	2.673023	-0.253849
H	5.186214	-2.452941	-0.235397
O	-0.767039	-0.019805	1.786887
O	-1.139505	-1.307999	2.251941
H	-0.299825	-1.791376	2.231120

SCF Energy: -1104.89977958
Sum of electronic and zero-point Energies= -1104.657537
Sum of electronic and thermal Energies= -1104.639437
Sum of electronic and thermal Enthalpies= -1104.638493

Sum of electronic and thermal Free Energies= -1104.704209

==> ./AOX-I/water/RAF/C8 <==
O -0.344163 -0.551502 -0.099762
O -1.902757 2.693577 0.118398
O 3.258541 2.615566 -0.040153
O 0.691928 3.337736 0.128289
O 4.066412 -2.045634 -0.681388
C 1.395341 1.075232 -0.034490
C 0.959777 -0.227622 -0.106479
C -1.311186 0.395503 -0.020183
C 0.409700 2.123471 0.065818
C -0.970539 1.708064 0.068115
C -2.665151 -0.164699 -0.078552
C 2.789202 1.348361 -0.125920
C 1.856886 -1.403070 -0.148536
C 3.257205 -0.987792 -0.419793
C 3.696053 0.301912 -0.342218
C -2.885816 -1.336557 -0.809699
C -3.729080 0.444162 0.592353
C -4.159499 -1.881329 -0.879487
C -4.999280 -0.112005 0.521937
C -5.218812 -1.270139 -0.215744
H -2.064922 -1.809967 -1.329355
H -3.562848 1.333954 1.179675
H -4.325369 -2.782258 -1.453887
H -5.817343 0.359191 1.049014
H -6.210997 -1.696852 -0.270897
H -1.417102 3.534065 0.129743
H 2.490621 3.214941 0.054022
H 4.975123 -1.748790 -0.820014
H 4.745460 0.529999 -0.480176
H 1.504448 -2.152816 -0.858861
O 1.758049 -2.037774 1.178223
O 1.994781 -3.431414 1.049552
H 2.951803 -3.506848 1.172720

SCF Energy: -1104.86834123
Sum of electronic and zero-point Energies= -1104.627174
Sum of electronic and thermal Energies= -1104.608549
Sum of electronic and thermal Enthalpies= -1104.607605
Sum of electronic and thermal Free Energies= -1104.675149

==> ./AOX-I/water/PA/C5 <==
O -0.180244 -0.843592 0.085347
O -1.368493 2.559355 -0.162275
O 3.801442 1.850700 -0.105906
O 1.194373 2.949918 -0.179043
O 4.009099 -2.876894 0.178948
C 1.750143 0.615617 -0.017848
C 1.172847 -0.669736 0.062516
C -1.043648 0.210591 0.022260
C 0.873738 1.746632 -0.086075
C -0.562996 1.465344 -0.060524
C -2.450651 -0.210617 0.018704
C 3.201721 0.726306 -0.034510
C 1.907228 -1.836036 0.128287
C 3.296335 -1.711180 0.111921
C 3.932136 -0.487331 0.033892
C -2.790472 -1.456884 -0.518350
C -3.454092 0.602445 0.553721
C -4.114132 -1.873444 -0.533251
C -4.775797 0.176275 0.539309
C -5.110822 -1.058242 -0.006322
H 1.422810 -2.798445 0.190885
H 5.013914 -0.437886 0.022929
H -2.020173 -2.092498 -0.931056
H -3.201831 1.554360 0.994795
H -4.366974 -2.835207 -0.958266
H -5.544131 0.809242 0.961799
H -6.141639 -1.385128 -0.017435
H -0.753120 3.310167 -0.243631
H 4.952503 -2.678933 0.161029

SCF Energy: -953.471637221
Sum of electronic and zero-point Energies= -953.262050
Sum of electronic and thermal Energies= -953.246409
Sum of electronic and thermal Enthalpies= -953.245465
Sum of electronic and thermal Free Energies= -953.305980

==> ./AOX-I/water/PA/C7/aIP <==
O -0.205565 -0.899277 0.089821
O -1.292437 2.519110 -0.186992
O 3.768256 1.771829 -0.099991
O 1.359627 2.821887 -0.177567
O 3.993809 -2.912971 0.187745
C 1.742257 0.482602 -0.012128
C 1.138541 -0.765124 0.069210
C -1.024742 0.172039 0.019949
C 0.917453 1.656107 -0.087074
C -0.504015 1.431281 -0.074117
C -2.442686 -0.190270 0.017488
C 3.191603 0.559228 -0.023574
C 1.881513 -1.934389 0.1374[67]57
C 3.333034 -1.856230 0.125628
C 3.953125 -0.561356 0.042050
C -2.828279 -1.411786 -0.545864

C -3.406096 0.651176 0.582035
C -4.165709 -1.776517 -0.557988
C -4.740890 0.271456 0.574455
C -5.123857 -0.936576 0.001554
H 1.399838 -2.898794 0.198217
H 5.031577 -0.497152 0.033420
H -2.084615 -2.062460 -0.983393
H -3.113521 1.582456 1.041781
H -4.460367 -2.715712 -1.005161
H -5.481908 0.919719 1.021085
H -6.166171 -1.224606 -0.006660
H -0.704154 3.288319 -0.269188
H 3.062292 2.453749 -0.148723

SCF Energy: -953.278669842
Sum of electronic and zero-point Energies= -953.069079
Sum of electronic and thermal Energies= -953.053631
Sum of electronic and thermal Enthalpies= -953.052687
Sum of electronic and thermal Free Energies= -953.112844

==> ./AOX-I/water/PA/C7/aIP/vEA <==
O -0.205565 -0.899277 0.089821
O -1.292437 2.519110 -0.186992
O 3.768256 1.771829 -0.099991
O 1.359627 2.821887 -0.177567
O 3.993809 -2.912971 0.187745
C 1.742257 0.482602 -0.012128
C 1.138541 -0.765124 0.069210
C -1.024742 0.172039 0.019949
C 0.917453 1.656107 -0.087074
C -0.504015 1.431281 -0.074117
C -2.442686 -0.190270 0.017488
C 3.191603 0.559228 -0.023574
C 1.881513 -1.934389 0.137457
C 3.333034 -1.856230 0.125628
C 3.953125 -0.561356 0.042050
C -2.828279 -1.411786 -0.545864
C -3.406096 0.651176 0.582035
C -4.165709 -1.776517 -0.557988
C -4.740890 0.271456 0.574455
C -5.123857 -0.936576 0.001554
H 1.399838 -2.898794 0.198217
H 5.031577 -0.497152 0.033420
H -2.084615 -2.062460 -0.983393
H -3.113521 1.582456 1.041781
H -4.460367 -2.715712 -1.005161
H -5.481908 0.919719 1.021085
H -6.166171 -1.224606 -0.006660
H -0.704154 3.288319 -0.269188
H 3.062292 2.453749 -0.148723

SCF Energy: -953.473419258

==> ./AOX-I/water/PA/C7/HAT/C5/vEA <==
O -0.151675 -0.840458 0.093066
O -1.318216 2.552543 -0.159035
O 3.837155 1.748383 -0.125526
O 1.259870 2.935896 -0.175428
O 4.005301 -2.943668 0.195678
C 1.769785 0.588892 -0.018698
C 1.184080 -0.659141 0.066869
C -1.009559 0.209926 0.027817
C 0.915683 1.745007 -0.084104
C -0.516032 1.469095 -0.057797
C -2.417346 -0.200280 0.020602
C 3.254373 0.642102 -0.040868
C 1.927175 -1.849052 0.138713
C 3.376452 -1.834331 0.126176
C 3.979750 -0.577422 0.035121
C -2.761994 -1.439325 -0.529884
C -3.413838 0.616076 0.563047
C -4.087809 -1.846685 -0.549676
C -4.737511 0.197341 0.544249
C -5.078642 -1.029687 -0.014227
H 1.401809 -2.791051 0.204684
H 5.059526 -0.507108 0.018793
H -1.995065 -2.074152 -0.950265
H -3.155671 1.562240 1.012916
H -4.347245 -2.801696 -0.985431
H -5.502557 0.830348 0.972242
H -6.111532 -1.349659 -0.029706
H -0.716145 3.314407 -0.237303

SCF Energy: -952.994407006

==> ./AOX-I/water/PA/C7/HAT/C5 <==
O -0.151675 -0.840458 0.093066
O -1.318216 2.552543 -0.159035
O 3.837155 1.748383 -0.125526
O 1.259870 2.935896 -0.175428
O 4.005301 -2.943668 0.195678
C 1.769785 0.588892 -0.018698
C 1.184080 -0.659141 0.066869
C -1.009559 0.209926 0.027817
C 0.915683 1.745007 -0.084104
C -0.516032 1.469095 -0.057797
C -2.417346 -0.200280 0.020602
C 3.254373 0.642102 -0.040868
C 1.927175 -1.849052 0.138713
C 3.376452 -1.834331 0.126176

C	3.979750	-0.577422	0.035121
C	-2.761994	-1.439325	-0.529884
C	-3.413838	0.616076	0.563047
C	-4.087809	-1.846685	-0.549676
C	-4.737511	0.197341	0.544249
C	-5.078642	-1.029687	-0.014227
H	1.401809	-2.791051	0.204684
H	5.059526	-0.507108	0.018793
H	-1.995065	-2.074152	-0.950265
H	-3.155671	1.562240	1.012916
H	-4.347245	-2.801696	-0.985431
H	-5.502557	0.830348	0.972242
H	-6.111532	-1.349659	-0.029706
H	-0.716145	3.314407	-0.237303

SCF Energy: -952.821192915
Sum of electronic and zero-point Energies= -952.624416
Sum of electronic and thermal Energies= -952.609035
Sum of electronic and thermal Enthalpies= -952.608091
Sum of electronic and thermal Free Energies= -952.668654

==> ./AOX-I/water/PA/C7/HAT/C5/TS <==

O	0.778477	0.932358	-0.021976
O	1.698691	-2.541473	-0.418074
O	-3.376818	-1.334453	-0.798459
O	-0.886694	-2.683590	-0.743343
O	-3.240820	3.317605	-0.174718
C	-1.229171	-0.328804	-0.426981
C	-0.567934	0.895178	-0.185482
C	1.551549	-0.194371	-0.080260
C	-0.462953	-1.535653	-0.508657
C	0.979425	-1.391745	-0.311728
C	2.979707	0.095741	0.095096
C	-2.643033	-0.250155	-0.592028
C	-1.200063	2.122037	-0.087698
C	-2.598169	2.220775	-0.246537
C	-3.294690	0.984093	-0.513802
C	3.479075	1.340055	-0.304317
C	3.845707	-0.840988	0.666822
C	4.826253	1.633551	-0.148368
C	5.191524	-0.537088	0.824349
C	5.686494	0.696353	0.414967
H	-0.608695	3.006556	0.102750
H	-4.362848	1.014100	-0.684963
H	2.814562	2.070119	-0.743786
H	3.467329	-1.793851	1.003467
H	5.204100	2.595101	-0.467883
H	5.852809	-1.264676	1.274609
H	6.735755	0.927866	0.537791
H	1.044330	-3.230604	-0.628614
H	-4.058586	-1.456276	0.056325
O	-4.638847	-1.410131	1.225702
O	-4.150472	-0.249131	1.735629
H	-4.819191	0.428308	1.539953

SCF Energy: -1104.38759235
Sum of electronic and zero-point Energies= -1104.165889
Sum of electronic and thermal Energies= -1104.147539
Sum of electronic and thermal Enthalpies= -1104.146594
Sum of electronic and thermal Free Energies= -1104.214457

==> ./AOX-I/water/PA/C7/HAT/C3 <==

O	-0.230642	-0.854522	0.070595
O	-1.252200	2.529758	-0.240533
O	3.806769	1.757579	-0.060191
O	1.433510	2.864500	-0.124084
O	3.955646	-2.949099	0.150893
C	1.746057	0.532203	-0.006923
C	1.142273	-0.743522	0.051135
C	-1.043689	0.204504	0.006452
C	0.967985	1.705148	-0.070789
C	-0.524528	1.531234	-0.100850
C	-2.453215	-0.147660	0.010959
C	3.177854	0.559058	-0.006053
C	1.843268	-1.908246	0.103574
C	3.281579	-1.875147	0.102121
C	3.914582	-0.591054	0.045324
C	-2.833944	-1.422958	-0.436824
C	-3.434816	0.739985	0.476329
C	-4.168994	-1.790148	-0.439510
C	-4.767133	0.355792	0.480707
C	-5.139187	-0.902588	0.018457
H	1.328515	-2.857316	0.149823
H	4.994826	-0.538686	0.043376
H	-2.083666	-2.111164	-0.797713
H	-3.152896	1.709627	0.852857
H	-4.454490	-2.768834	-0.799435
H	-5.517068	1.040378	0.851943
H	-6.180849	-1.193001	0.018969
H	3.112407	2.453673	-0.097917

SCF Energy: -952.840683767
Sum of electronic and zero-point Energies= -952.642988
Sum of electronic and thermal Energies= -952.627949
Sum of electronic and thermal Enthalpies= -952.627005

Sum of electronic and thermal Free Energies= -952.686596

==> ./AOX-I/water/PA/C7/HAT/C3/vEA <==

O	-0.230642	-0.854522	0.070595
O	-1.252200	2.529758	-0.240533
O	3.806769	1.757579	-0.060191
O	1.433510	2.864500	-0.124084
O	3.955646	-2.949099	0.150893
C	1.746057	0.532203	-0.006923
C	1.142273	-0.743522	0.051135
C	-1.043689	0.204504	0.006452
C	0.967985	1.705148	-0.070789
C	-0.524528	1.531234	-0.100850
C	-2.453215	-0.147660	0.010959
C	3.177854	0.559058	-0.006053
C	1.843268	-1.908246	0.103574
C	3.281579	-1.875147	0.102121
C	3.914582	-0.591054	0.045324
C	-2.833944	-1.422958	-0.436824
C	-3.434816	0.739985	0.476329
C	-4.168994	-1.790148	-0.439510
C	-4.767133	0.355792	0.480707
C	-5.139187	-0.902588	0.018457
H	1.328515	-2.857316	0.149823
H	4.994826	-0.538686	0.043376
H	-2.083666	-2.111164	-0.797713
H	-3.152896	1.709627	0.852857
H	-4.454490	-2.768834	-0.799435
H	-5.517068	1.040378	0.851943
H	-6.180849	-1.193001	0.018969
H	3.112407	2.453673	-0.097917

SCF Energy: -952.998319577

==> ./AOX-I/water/PA/C7/HAT/C3/TS <==

O	-0.010366	-1.200890	0.084595
O	-1.311170	1.949687	-1.022216
O	3.808881	1.647245	-0.538251
O	1.347586	2.506169	-0.889897
O	4.330903	-2.878172	0.693175
C	1.852284	0.287251	-0.254750
C	1.350166	-0.988833	0.067950
C	-0.887063	-0.232816	-0.220609
C	0.972164	1.346056	-0.595652
C	-0.459640	1.025288	-0.590283
C	-2.276571	-0.689714	-0.167624
C	3.270084	0.437121	-0.236076
C	2.140567	-2.056270	0.387319
C	3.563922	-1.902351	0.401418
C	4.092210	-0.610597	0.077533
C	-2.558734	-2.024874	-0.480435
C	-3.313174	0.168298	0.214527
C	-3.864729	-2.487368	-0.432455
C	-4.614766	-0.308522	0.274302
C	-4.894448	-1.630989	-0.054701
H	1.697862	-3.012078	0.629136
H	5.164427	-0.467464	0.082730
H	-1.758520	-2.688550	-0.774992
H	-3.102362	1.190726	0.487027
H	-4.078804	-3.515654	-0.688719
H	-5.411634	0.353869	0.582668
H	-5.912028	-1.994735	-0.012791
H	3.064030	2.257174	-0.735413
H	-1.462184	2.670203	-0.248737
O	-1.524220	3.250368	0.982515
O	-0.944609	2.306982	1.752547
H	-0.010466	2.565309	1.836549

SCF Energy: -1104.40268758
Sum of electronic and zero-point Energies= -1104.180378
Sum of electronic and thermal Energies= -1104.162280
Sum of electronic and thermal Enthalpies= -1104.161336
Sum of electronic and thermal Free Energies= -1104.227983

==> ./AOX-I/water/PA/C7/vIP <==

O	-0.198570	-0.904164	0.081962
O	-1.296620	2.540593	-0.178149
O	3.774268	1.784559	-0.101687
O	1.341930	2.837450	-0.178594
O	4.042981	-2.921050	0.195153
C	1.743099	0.503566	-0.015361
C	1.161278	-0.778594	0.064357
C	-1.024314	0.181597	0.015393
C	0.920233	1.652980	-0.088423
C	-0.514246	1.429047	-0.071070
C	-2.444328	-0.192310	0.015405
C	3.166507	0.567889	-0.023822
C	1.899461	-1.933450	0.133049
C	3.322709	-1.865378	0.127665
C	3.926356	-0.565774	0.045144
C	-2.830690	-1.411097	-0.551885
C	-3.412463	0.638318	0.586750
C	-4.167431	-1.783240	-0.560508
C	-4.747411	0.255792	0.579315
C	-5.129360	-0.951160	0.003572
H	1.404497	-2.892305	0.195375
H	5.005573	-0.490250	0.038034
H	-2.086300	-2.058830	-0.992521
H	-3.122240	1.568299	1.051189
H	-4.457827	-2.723403	-1.009072

H	-5.488970	0.900546	1.030744
H	-6.170411	-1.244003	-0.002020
H	-0.681875	3.287591	-0.267199
H	3.069235	2.464140	-0.151486

SCF Energy: -953.274333877

==> ./AOX-I/water/PA/C7/RAF/C8a <==

O	-0.334897	-0.816679	-0.338181
O	-1.485732	2.564246	0.380919
O	3.497113	1.961805	-0.480773
O	1.121546	2.962420	0.001274
O	3.881589	-2.701178	-0.969512
C	1.575017	0.636152	-0.092448
C	0.982665	-0.695381	0.138616
C	-1.190857	0.249024	-0.105048
C	0.743058	1.747907	0.022767
C	-0.695214	1.479747	0.122221
C	-2.603152	-0.144259	-0.161686
C	2.950809	0.736718	-0.401860
C	1.800412	-1.813506	-0.379630
C	3.208378	-1.669413	-0.681670
C	3.756080	-0.361293	-0.659572
C	-2.967213	-1.440218	0.220026
C	-3.591320	0.741841	-0.604192
C	-4.297323	-1.835270	0.175340
C	-4.918600	0.338035	-0.650436
C	-5.276846	-0.948010	-0.258163
H	-2.209139	-2.131125	0.559998
H	-3.320127	1.735880	-0.925323
H	-4.568327	-2.836978	0.479925
H	-5.673962	1.028206	-1.000700
H	-6.312426	-1.257678	-0.294472
H	-0.880684	3.322680	0.425605
H	2.782274	2.618483	-0.293370
H	4.800069	-0.214048	-0.896855
H	1.342074	-2.791064	-0.434734
O	0.755713	-0.949607	1.592836
O	1.965238	-0.747958	2.292229
H	1.980318	0.208801	2.444000

SCF Energy: -1104.40256946
Sum of electronic and zero-point Energies= -1104.174996
Sum of electronic and thermal Energies= -1104.156901
Sum of electronic and thermal Enthalpies= -1104.155957
Sum of electronic and thermal Free Energies= -1104.221627

==> ./AOX-I/water/PA/C7/RAF/C5/TS <==

O	-0.693149	0.957328	-0.170980
O	-1.608096	-2.547767	-0.080691
O	3.375624	-1.481712	-0.747619
O	1.043905	-2.695630	-0.291164
O	3.392676	3.230707	-0.581792
C	1.290877	-0.342697	-0.349569
C	0.656344	0.901618	-0.277580
C	-1.468795	-0.171710	-0.117144
C	0.547736	-1.535394	-0.277289
C	-0.895503	-1.393078	-0.169146
C	-2.896214	0.136150	0.028628
C	2.735768	-0.346137	-0.385493
C	1.324078	2.116271	-0.329869
C	2.728237	2.148944	-0.490393
C	3.415102	0.879376	-0.578834
C	-3.280913	1.311855	0.682195
C	-3.877260	-0.715923	-0.487204
C	-4.625887	1.619829	0.828905
C	-5.220932	-0.397810	-0.339862
C	-5.599660	0.766150	0.320198
H	0.763523	3.037761	-0.265715
H	4.488144	0.867472	-0.705738
H	-2.528184	1.976548	1.081205
H	-3.592747	-1.612269	-1.016461
H	-4.913260	2.527194	1.342264
H	-5.972581	-1.059087	-0.748708
H	-6.647365	1.009113	0.433821
H	-0.954451	-3.266670	-0.094854
H	2.740941	-2.221205	-0.624721
O	3.040620	-0.372404	1.500570
O	4.352848	-0.770813	1.728363
H	4.382677	-1.681189	1.400155

SCF Energy: -1104.39366721
Sum of electronic and zero-point Energies= -1104.167883
Sum of electronic and thermal Energies= -1104.149598
Sum of electronic and thermal Enthalpies= -1104.148654
Sum of electronic and thermal Free Energies= -1104.215274

==> ./AOX-I/water/PA/C7/RAF/C5 <==

O	-0.698439	0.952566	-0.118850
O	-1.594774	-2.554668	-0.077683
O	3.348754	-1.356619	-0.937146
O	1.053127	-2.688666	-0.200520
O	3.332759	3.298463	-0.561638
C	1.292405	-0.331872	-0.176637
C	0.650418	0.898877	-0.166756
C	-1.476642	-0.177541	-0.086960
C	0.558391	-1.527083	-0.173449

C -0.892663 -1.393332 -0.126680
C -2.907622 0.128004 0.021038
C 2.786758 -0.353282 -0.135689
C 1.301633 2.135347 -0.234307
C 2.698316 2.199173 -0.417584
C 3.414381 0.952401 -0.467210
C -3.310834 1.310094 0.651870
C -3.875388 -0.734261 -0.503247
C -4.659721 1.614354 0.767609
C -5.223082 -0.420256 -0.386065
C -5.620096 0.750146 0.251379
H 0.713437 3.041399 -0.209960
H 4.476104 0.962888 -0.665100
H -2.569526 1.983255 1.057988
H -3.577737 -1.636107 -1.015458
H -4.960492 2.526991 1.263725
H -5.963563 -1.090489 -0.800736
H -6.670834 0.989713 0.341746
H -0.929135 -3.263965 -0.078706
H 2.785870 -2.144180 -0.819599
O 3.051693 -0.676887 1.268340
O 4.455520 -0.731439 1.484198
H 4.689238 -1.616125 1.168627

SCF Energy: -1104.40900718
Sum of electronic and zero-point Energies= -1104.181005
Sum of electronic and thermal Energies= -1104.162809
Sum of electronic and thermal Enthalpies= -1104.161865
Sum of electronic and thermal Free Energies= -1104.228089

==> ./AOX-I/water/PA/C7/RAF/C4p <==
O 0.473492 -0.806676 -0.092048
O -0.330189 2.729184 -0.094631
O 4.631737 1.580094 0.164717
O 2.284183 2.816920 0.064513
O 4.540900 -3.139776 0.082123
C 2.513180 0.453779 0.036984
C 1.834464 -0.781430 -0.021797
C -0.297573 0.331696 -0.115358
C 1.777893 1.660081 0.018890
C 0.334620 1.552886 -0.064448
C -1.693343 0.057779 -0.199096
C 3.935979 0.410878 0.109678
C 2.483133 -1.988822 -0.009460
C 3.907353 -2.029926 0.066370
C 4.608204 -0.778561 0.124107
C -2.137909 -1.308045 -0.287553
C -2.696577 1.086631 -0.182056
C -3.443066 -1.629062 -0.377275
C -4.009740 0.791790 -0.277537
C -4.519563 -0.601843 -0.410187
H -1.402404 -2.097591 -0.288084
H -2.399359 2.116712 -0.086201
H -3.751015 -2.663664 -0.444806
H -4.744368 1.586045 -0.260986
H -5.123119 -0.712032 -1.316647
H 0.354030 3.420684 -0.047229
H 3.981985 2.313686 0.140302
H 5.688522 -0.785776 0.180075
H 1.919546 -2.910064 -0.055317
O -5.406618 -0.941610 0.695796
O -6.636859 -0.255454 0.505893
H -6.559327 0.503554 1.100423

SCF Energy: -1104.41146759
Sum of electronic and zero-point Energies= -1104.184595
Sum of electronic and thermal Energies= -1104.166126
Sum of electronic and thermal Enthalpies= -1104.165181
Sum of electronic and thermal Free Energies= -1104.233678

==> ./AOX-I/water/PA/C7/RAF/C8 <==
O -0.340452 -0.580744 -0.115378
O -1.895926 2.670526 0.132173
O 3.244640 2.632513 -0.089479
O 0.682120 3.330775 0.103197
O 4.140873 -2.005310 -0.500174
C 1.407044 1.071888 -0.052208
C 0.980511 -0.252519 -0.119276
C -1.300022 0.370597 -0.020829
C 0.421430 2.105190 0.050513
C -0.961068 1.681176 0.070016
C -2.659573 -0.182464 -0.065297
C 2.792369 1.347707 -0.156014
C 1.881817 -1.404841 -0.193727
C 3.329102 -1.018786 -0.379510
C 3.716583 0.320526 -0.326275
C -2.902022 -1.336534 -0.817177
C -3.706640 0.413678 0.642248
C -4.179443 -1.875260 -0.871531
C -4.980838 -0.135644 0.587508
C -5.221750 -1.275894 -0.171190
H -2.094355 -1.801732 -1.364343
H -3.523110 1.288942 1.246320
H -4.361412 -2.762487 -1.462282
H -5.784954 0.326239 1.143666
H -6.216569 -1.697967 -0.213749
H -1.402706 3.506827 0.134976
H 2.464478 3.216001 0.001070

H	4.767176	0.565270	-0.411965
H	1.563030	-2.135512	-0.942615
O	1.795029	-2.132270	1.099520
O	2.333417	-3.445228	0.907794
H	3.205799	-3.240598	0.504184

SCF Energy: -1104.41236738
Sum of electronic and zero-point Energies= -1104.184392
Sum of electronic and thermal Energies= -1104.166300
Sum of electronic and thermal Enthalpies= -1104.165356
Sum of electronic and thermal Free Energies= -1104.231820

==> ./AOX-I/water/PA/C7/RAF/C2 <==

O	-0.066476	-0.941865	-0.234796
O	-1.324943	2.393911	0.123523
O	3.768020	1.969998	-0.232273
O	1.305208	2.912448	-0.270714
O	4.288877	-2.719541	-0.236497
C	1.815427	0.579855	-0.177278
C	1.299911	-0.739110	-0.175502
C	-0.899406	0.019546	0.370040
C	0.940220	1.700874	-0.152283
C	-0.437538	1.408295	0.055939
C	-2.321821	-0.240717	-0.071214
C	3.231177	0.720514	-0.216431
C	2.098245	-1.842345	-0.192565
C	3.528290	-1.697802	-0.220450
C	4.059836	-0.370091	-0.230749
C	-2.572312	-0.727575	-1.350164
C	-3.379198	0.080537	0.775191
C	-3.883772	-0.909152	-1.775513
C	-4.687468	-0.102841	0.345594
C	-4.941861	-0.598596	-0.929208
H	1.662898	-2.831964	-0.199250
H	5.132317	-0.232182	-0.254723
H	-1.750094	-0.963588	-2.010499
H	-3.181002	0.465681	1.765648
H	-4.075936	-1.291920	-2.768482
H	-5.507406	0.141098	1.007103
H	-5.961283	-0.740998	-1.261256
H	-0.845595	3.230019	-0.019873
H	3.022370	2.610354	-0.256212
O	-0.744582	-0.052643	1.791536
O	-1.136950	-1.341507	2.242278
H	-0.307881	-1.841244	2.200638

SCF Energy: -1104.43991722
Sum of electronic and zero-point Energies= -1104.211059
Sum of electronic and thermal Energies= -1104.193230
Sum of electronic and thermal Enthalpies= -1104.192286
Sum of electronic and thermal Free Energies= -1104.257779

==> ./AOX-I/water/PA/C7/RAF/C2/TS <==

O	-0.178561	-0.907097	-0.184947
O	-1.224967	2.529927	0.033164
O	3.819817	1.770551	-0.277242
O	1.405174	2.849075	-0.138822
O	4.040402	-2.936108	-0.505959
C	1.781846	0.501889	-0.228821
C	1.189369	-0.782920	-0.241091
C	-0.987998	0.162848	0.007293
C	0.978931	1.659826	-0.156335
C	-0.450714	1.446733	-0.092128
C	-2.414068	-0.174718	-0.179451
C	3.207514	0.557120	-0.297641
C	1.912077	-1.936246	-0.335923
C	3.340699	-1.876513	-0.414741
C	3.957387	-0.580422	-0.386040
C	-3.257313	0.636056	-0.937841
C	-2.898530	-1.367330	0.363886
C	-4.581146	0.260344	-1.137404
C	-4.219205	-1.735894	0.160559
C	-5.064486	-0.920348	-0.587814
H	1.413200	-2.894894	-0.344458
H	5.035559	-0.512779	-0.439821
H	-2.884871	1.545438	-1.384348
H	-2.240514	-1.994407	0.948779
H	-5.230388	0.890401	-1.729539
H	-4.591067	-2.657231	0.587163
H	-6.095164	-1.208447	-0.744118
H	-0.625544	3.297927	0.052689
H	3.121773	2.457234	-0.212207
O	-0.996507	0.227132	2.033600
O	0.179844	-0.337808	2.465280
H	0.769231	0.408830	2.646212

SCF Energy: -1104.40600925
Sum of electronic and zero-point Energies= -1104.180079
Sum of electronic and thermal Energies= -1104.161845
Sum of electronic and thermal Enthalpies= -1104.160901
Sum of electronic and thermal Free Energies= -1104.227342

==> ./AOX-I/water/PA/C7/RAF/C3p <==

O	-0.220624	0.610820	0.082292

O	0.107958	-2.987613	-0.196101
O	-4.680572	-1.159247	-0.089611
O	-2.533352	-2.711839	-0.159806
O	-3.930869	3.494269	0.168269
C	-2.420747	-0.345916	-0.006117
C	-1.576044	0.781735	0.065119
C	0.348823	-0.627686	0.011396
C	-1.866759	-1.645840	-0.078089
C	-0.416003	-1.736120	-0.073453
C	1.819507	-0.575501	0.004764
C	-3.824933	-0.101957	-0.016645
C	-2.047445	2.068934	0.126891
C	-3.452268	2.309173	0.116620
C	-4.321712	1.168986	0.044161
C	2.446013	0.498332	-0.559928
C	2.573723	-1.624735	0.595538
C	3.927854	0.622110	-0.589994
C	3.984275	-1.579174	0.567858
C	4.650298	-0.540571	-0.002614
H	1.878315	1.300900	-1.007809
H	2.070240	-2.443830	1.081177
H	4.271799	0.829391	-1.607497
H	4.538230	-2.393704	1.014647
H	5.731174	-0.520240	-0.027844
H	-0.653195	-3.587827	-0.259579
H	-4.138951	-1.975129	-0.133907
H	-5.391710	1.328599	0.034052
H	-1.357244	2.899001	0.181389
O	4.214368	1.844517	0.168794
O	5.549588	2.241660	-0.115299
H	6.051870	1.871672	0.624030

SCF Energy: -1104.40266815
Sum of electronic and zero-point Energies= -1104.175772
Sum of electronic and thermal Energies= -1104.157473
Sum of electronic and thermal Enthalpies= -1104.156529
Sum of electronic and thermal Free Energies= -1104.223290

==> ./AOX-I/water/PA/C7/RAF/C5p <==

O	0.462691	-0.908001	-0.056909
O	-0.616039	2.528468	-0.469668
O	4.431030	1.792125	0.087921
O	2.010505	2.836575	-0.221356
O	4.677043	-2.902852	0.518893
C	2.400796	0.507033	0.013208
C	1.817328	-0.776469	0.060940
C	-0.352073	0.173934	-0.226830
C	1.585892	1.652492	-0.154251
C	0.155880	1.422850	-0.274151
C	-1.764132	-0.209099	-0.378412
C	3.819095	0.576137	0.136603
C	2.547295	-1.926423	0.226150
C	3.964337	-1.852770	0.357530
C	4.570535	-0.552840	0.302673
C	-2.066174	-1.429495	-1.043634
C	-2.760827	0.584409	0.108142
C	-3.409816	-1.814797	-1.232156
C	-4.197285	0.221696	-0.036895
C	-4.436985	-1.045902	-0.781660
H	-1.266256	-2.050481	-1.414787
H	-2.540157	1.497785	0.639029
H	-3.618099	-2.738674	-1.754778
H	-5.464539	-1.342529	-0.940721
H	0.002611	3.275967	-0.520805
H	3.735012	2.469921	-0.040786
H	5.645465	-0.473360	0.395688
H	2.050585	-2.885833	0.258784
H	-4.757628	1.051532	-0.478175
O	-4.689769	0.114539	1.339688
O	-6.110626	0.140036	1.305774
H	-6.348869	-0.795995	1.358272

SCF Energy: -1104.40270100
Sum of electronic and zero-point Energies= -1104.175795
Sum of electronic and thermal Energies= -1104.157416
Sum of electronic and thermal Enthalpies= -1104.156472
Sum of electronic and thermal Free Energies= -1104.223846

==> ./AOX-I/water/PA/C7/RAF/C6 <==

O	-0.701573	-0.827494	-0.066853
O	-2.010332	2.548080	-0.126093
O	3.101803	2.077990	-0.475050
O	0.602754	2.998348	-0.325955
O	3.585000	-2.616833	-0.658886
C	1.143875	0.689330	-0.229228
C	0.628693	-0.628955	-0.188959
C	-1.602784	0.200953	-0.025689
C	0.243327	1.784245	-0.226814
C	-1.162828	1.479761	-0.111322
C	-2.988105	-0.266317	0.074265
C	2.530739	0.848333	-0.346139
C	1.423027	-1.761095	-0.293915
C	2.817000	-1.646112	-0.430054
C	3.461963	-0.282563	-0.223445
C	-3.330447	-1.521283	-0.443503
C	-3.974334	0.509998	0.692390
C	-4.636387	-1.981554	-0.355674
C	-5.278350	0.040500	0.777934

C	-5.615411	-1.202572	0.253198
H	0.959600	-2.736705	-0.337074
H	-2.575818	-2.129415	-0.921369
H	-3.720495	1.467248	1.120661
H	-4.889780	-2.949746	-0.765607
H	-6.030972	0.646198	1.264047
H	-6.632548	-1.563542	0.321588
H	-1.442051	3.328117	-0.239099
H	2.368773	2.731773	-0.466631
H	4.332090	-0.165926	-0.871310
O	3.970251	-0.210663	1.163886
O	5.178954	-0.957366	1.275808
H	5.856469	-0.300513	1.061655

SCF Energy: -1104.41212967
Sum of electronic and zero-point Energies= -1104.184218
Sum of electronic and thermal Energies= -1104.165938
Sum of electronic and thermal Enthalpies= -1104.164994
Sum of electronic and thermal Free Energies= -1104.231597

==> ./AOX-I/water/PA/C7/RAF/C2p <==

O	0.021207	0.556586	-0.293382
O	0.659092	-2.964116	0.274466
O	-4.253487	-1.553840	0.255952
O	-1.975723	-2.907140	0.371982
O	-3.921503	3.098821	-0.476203
C	-2.079316	-0.566033	-0.009691
C	-1.338575	0.613752	-0.228806
C	0.721785	-0.611066	-0.136216
C	-1.407900	-1.799475	0.158041
C	0.039142	-1.773774	0.086941
C	2.145029	-0.420774	-0.226364
C	-3.497897	-0.440454	0.044141
C	-1.922810	1.844686	-0.389063
C	-3.342027	1.967629	-0.334641
C	-4.106302	0.772660	-0.112735
C	2.631504	1.001221	-0.182273
C	3.054950	-1.462294	-0.336417
C	4.095024	1.167688	-0.416105
C	4.418451	-1.220318	-0.473161
C	4.927192	0.110393	-0.524646
H	2.055411	1.631378	-0.860222
H	2.705577	-2.481035	-0.352057
H	4.466527	2.182560	-0.463492
H	5.097108	-2.055350	-0.563399
H	5.988774	0.263945	-0.663812
H	-0.050304	-3.608945	0.439689
H	-3.639886	-2.312749	0.350574
H	-5.184894	0.843344	-0.067526
H	-1.310366	2.719968	-0.554595
O	2.293373	1.461660	1.168412
O	2.265493	2.881880	1.162486
H	3.134937	3.123035	1.511101

SCF Energy: -1104.40980363
Sum of electronic and zero-point Energies= -1104.182792
Sum of electronic and thermal Energies= -1104.164463
Sum of electronic and thermal Enthalpies= -1104.163518
Sum of electronic and thermal Free Energies= -1104.230908

==> ./AOX-I/water/PA/C7/RAF/C1p <==

O	-0.055194	0.820274	-0.222270
O	0.934520	-2.645263	0.122100
O	-4.107916	-1.701184	0.313998
O	-1.709818	-2.846019	0.302503
O	-4.219239	2.991275	-0.197823
C	-2.037912	-0.508081	0.052220
C	-1.416923	0.746587	-0.126371
C	0.721584	-0.290872	-0.143578
C	-1.256474	-1.683947	0.137954
C	0.186361	-1.513934	0.029761
C	2.206308	0.010489	-0.241728
C	-3.460855	-0.515329	0.142832
C	-2.114438	1.924057	-0.211721
C	-3.536807	1.912577	-0.120580
C	-4.181070	0.642568	0.061040
C	2.475565	1.435258	-0.606811
C	2.899522	-0.426598	1.010866
C	3.331728	2.211479	0.103682
C	3.725390	0.401992	1.702415
C	3.969980	1.723755	1.268971
H	1.986934	1.805633	-1.497193
H	2.717594	-1.439526	1.338976
H	3.526617	3.224514	-0.221927
H	4.207006	0.041595	2.601524
H	4.640903	2.361020	1.824718
H	0.303786	-3.369705	0.269527
H	-3.430105	-2.407323	0.354919
H	-5.260052	0.609675	0.132792
H	-1.589206	2.858790	-0.348282
O	2.605363	-0.846174	-1.376339
O	4.013131	-0.781704	-1.527032
H	4.137998	-0.034333	-2.129867

SCF Energy: -1104.39693501
Sum of electronic and zero-point Energies= -1104.170515

Sum of electronic and thermal Energies= -1104.152280
Sum of electronic and thermal Enthalpies= -1104.151336
Sum of electronic and thermal Free Energies= -1104.217469

==> ./AOX-I/water/PA/C7/RAF/C3/TS <==

O	-0.151725	-1.138652	0.167747
O	-1.275185	2.112872	-0.856140
O	3.806173	1.458205	-0.712190
O	1.336528	2.476420	-0.908963
O	4.105869	-3.068720	0.571170
C	1.784399	0.231265	-0.284308
C	1.215817	-1.006558	0.086346
C	-1.001744	-0.136615	-0.141404
C	0.955842	1.340607	-0.545917
C	-0.496194	1.148501	-0.348695
C	-2.399204	-0.528882	-0.141029
C	3.206870	0.289952	-0.359621
C	1.957088	-2.114986	0.376578
C	3.387068	-2.051342	0.305913
C	3.978062	-0.802928	-0.077097
C	-2.729207	-1.876591	-0.351171
C	-3.422213	0.401911	0.091177
C	-4.055451	-2.276932	-0.348551
C	-4.745664	-0.012478	0.098719
C	-5.067673	-1.347382	-0.125547
H	1.472302	-3.040559	0.652921
H	5.055556	-0.730616	-0.137737
H	-1.946173	-2.599252	-0.528500
H	-3.183367	1.434995	0.288700
H	-4.300548	-3.315686	-0.521699
H	-5.528110	0.709486	0.287353
H	-6.101890	-1.663089	-0.121942
H	-0.687863	2.860434	-1.065986
H	3.108343	2.122024	-0.877937
O	-0.340951	1.719533	1.577022
O	-0.448986	3.097888	1.562853
H	0.451184	3.416645	1.400486

SCF Energy: -1104.40637210
Sum of electronic and zero-point Energies= -1104.180185
Sum of electronic and thermal Energies= -1104.161940
Sum of electronic and thermal Enthalpies= -1104.160995
Sum of electronic and thermal Free Energies= -1104.227536

==> ./AOX-I/water/PA/C7/RAF/C3 <==

O	0.197856	1.065229	0.413135
O	1.284794	-2.277128	-0.480851
O	-3.685608	-1.315987	-1.152876
O	-1.243316	-2.407468	-1.099241
O	-4.040016	3.061323	0.546172
C	-1.712436	-0.227252	-0.315412
C	-1.157320	0.961989	0.220379
C	1.054939	0.051328	0.090736
C	-0.912394	-1.362829	-0.507113
C	0.489353	-1.327427	0.129957
C	2.418023	0.441932	-0.069296
C	-3.113312	-0.214388	-0.604074
C	-1.906673	2.059638	0.516116
C	-3.323081	2.049712	0.271395
C	-3.890799	0.869990	-0.312711
C	2.718582	1.806026	-0.287939
C	3.490302	-0.474463	0.007238
C	4.028424	2.223812	-0.441001
C	4.795171	-0.038344	-0.146290
C	5.076464	1.307359	-0.374526
H	-1.438960	2.953954	0.903111
H	-4.950851	0.847191	-0.525423
H	1.914845	2.524437	-0.350418
H	3.298251	-1.516041	0.204966
H	4.235020	3.270905	-0.616440
H	5.602637	-0.754744	-0.077916
H	6.098706	1.638077	-0.494626
H	0.676060	-2.914876	-0.890523
H	-2.986851	-1.979509	-1.312888
O	0.365506	-1.591360	1.546914
O	-0.325697	-2.822189	1.737472
H	-1.208480	-2.531257	2.009610

SCF Energy: -1104.42797869
Sum of electronic and zero-point Energies= -1104.200276
Sum of electronic and thermal Energies= -1104.182009
Sum of electronic and thermal Enthalpies= -1104.181065
Sum of electronic and thermal Free Energies= -1104.249161

==> ./AOX-I/water/PA/C7/RAF/C4 <==

O	-0.417241	0.896213	0.697825
O	-1.213089	-2.280002	-0.895626
O	3.483887	-1.142072	-1.112183
O	1.403888	-2.406571	-0.860267
O	3.471751	3.445067	0.022336
C	1.575408	-0.134293	-0.135127
C	0.894174	0.996013	0.310205
C	-1.186449	-0.129551	0.142413
C	0.893494	-1.471163	-0.042727
C	-0.587883	-1.258946	-0.254139

C	-2.609359	0.213950	0.044194
C	2.920097	-0.027896	-0.626007
C	1.516740	2.215293	0.381479
C	2.900132	2.330606	-0.048876
C	3.572520	1.170197	-0.569459
C	-2.993684	1.550416	-0.106914
C	-3.594107	-0.777243	0.104662
C	-4.337120	1.884276	-0.214069
C	-4.935900	-0.435956	-0.003141
C	-5.312433	0.893402	-0.165130
H	1.004038	3.096205	0.739871
H	4.592371	1.268853	-0.913467
H	-2.239921	2.323790	-0.148339
H	-3.311774	-1.809184	0.251397
H	-4.622064	2.920416	-0.336805
H	-5.688993	-1.210482	0.049890
H	-6.358455	1.155784	-0.246857
H	-0.483370	-2.845901	-1.213795
H	2.704376	-1.855378	-1.092087
O	0.930677	-1.889048	1.378339
O	2.288929	-2.106986	1.743994
H	2.519601	-1.291538	2.210668

SCF Energy: -1104.40754305
Sum of electronic and zero-point Energies= -1104.181588
Sum of electronic and thermal Energies= -1104.163752
Sum of electronic and thermal Enthalpies= -1104.162807
Sum of electronic and thermal Free Energies= -1104.228246

==> ./AOX-I/water/PA/C7/RAF/C6p <==

O	-0.344530	1.095196	-0.013537
O	1.104844	-2.189016	-0.531349
O	-3.978371	-2.038619	-0.138857
O	-1.448151	-2.784169	-0.395716
O	-4.778824	2.578915	0.429319
C	-2.111412	-0.527307	-0.086463
C	-1.677206	0.808931	0.035790
C	0.616927	0.131865	-0.190097
C	-1.165176	-1.560214	-0.270001
C	0.233484	-1.174158	-0.325764
C	1.947535	0.673986	-0.226333
C	-3.515599	-0.763160	-0.024159
C	-2.539898	1.860763	0.208519
C	-3.945068	1.622732	0.270439
C	-4.397529	0.265865	0.147194
C	2.149778	2.048460	-0.228461
C	3.126167	-0.255314	-0.197265
C	3.423734	2.598122	-0.288773
C	4.449875	0.417902	-0.349282
C	4.576417	1.761001	-0.366514
H	1.295843	2.708376	-0.206647
H	3.540902	3.671528	-0.293581
H	5.554318	2.215869	-0.448119
H	0.561978	-2.994183	-0.592718
H	-3.201395	-2.625181	-0.255758
H	-5.459242	0.063054	0.191226
H	-2.159348	2.868506	0.298263
H	5.315511	-0.227394	-0.415356
H	3.020394	-1.057956	-0.924044
O	3.052052	-0.895409	1.119826
O	3.867966	-2.057634	1.093917
H	4.698986	-1.753573	1.484924

SCF Energy: -1104.41044891
Sum of electronic and zero-point Energies= -1104.183405
Sum of electronic and thermal Energies= -1104.165141
Sum of electronic and thermal Enthalpies= -1104.164197
Sum of electronic and thermal Free Energies= -1104.230780

==> ./AOX-I/water/PA/C7/RAF/C4a <==

O	-0.451400	1.028583	0.141078
O	-1.322003	-2.287349	-1.059145
O	3.596737	-1.495682	-0.332123
O	1.365003	-2.462725	-0.936314
O	3.651971	3.209162	-0.373500
C	1.563499	-0.319286	0.190957
C	0.893289	0.998066	0.020293
C	-1.232555	-0.072750	-0.153837
C	0.791667	-1.387435	-0.502375
C	-0.603202	-1.226073	-0.564866
C	-2.654397	0.202826	-0.046759
C	3.017609	-0.292619	-0.202177
C	1.573919	2.150188	-0.131946
C	3.018417	2.140144	-0.261230
C	3.697531	0.859814	-0.335335
C	-3.106517	1.533306	0.006103
C	-3.606508	-0.828760	0.028201
C	-4.459905	1.816493	0.112271
C	-4.957426	-0.532805	0.134229
C	-5.395300	0.787873	0.174181
H	1.053380	3.094805	-0.205770
H	4.756228	0.852031	-0.555737
H	-2.396221	2.344794	-0.045061
H	-3.288636	-1.858662	0.018247
H	-4.784904	2.847738	0.145024
H	-5.672502	-1.342279	0.194633
H	-6.449907	1.012106	0.256635
H	-0.667933	-2.970576	-1.273316

H	2.841041	-2.134020	-0.559781
O	1.509225	-0.499390	1.680143
O	1.734670	-1.857107	2.016044
H	2.695116	-1.903778	2.129399

SCF Energy: -1104.39278205

Sum of electronic and zero-point Energies= -1104.166213

Sum of electronic and thermal Energies= -1104.148061

Sum of electronic and thermal Enthalpies= -1104.147117

Sum of electronic and thermal Free Energies= -1104.213151

==> ./AOX-I/water/PA/C7/RAF/C4a/TS <==

O	-0.441326	1.033167	-0.019066
O	-1.343929	-2.393113	-0.780147
O	3.641459	-1.496667	-0.214825
O	1.322576	-2.552557	-0.719030
O	3.673759	3.199376	-0.583381
C	1.569975	-0.302706	0.089083
C	0.913326	0.984095	-0.111298
C	-1.216556	-0.076225	-0.214481
C	0.798314	-1.434208	-0.430142
C	-0.619800	-1.268543	-0.494300
C	-2.645360	0.213152	-0.089980
C	3.017198	-0.297099	-0.219085
C	1.593904	2.143765	-0.290647
C	3.027020	2.131683	-0.420756
C	3.700108	0.849967	-0.410236
C	-3.051282	1.323722	0.663610
C	-3.620212	-0.576970	-0.712876
C	-4.398070	1.625179	0.800594
C	-4.965658	-0.263599	-0.573453
C	-5.361752	0.833051	0.184288
H	1.064564	3.081995	-0.381358
H	4.767486	0.825710	-0.583233
H	-2.311722	1.942675	1.150060
H	-3.330321	-1.419691	-1.319100
H	-4.694897	2.480924	1.391577
H	-5.706689	-0.878021	-1.066454
H	-6.411356	1.070826	0.290653
H	-0.696682	-3.110877	-0.867179
H	2.947143	-2.185618	-0.350716
O	1.527675	-0.414490	1.759869
O	1.549182	-1.766949	2.172567
H	2.490378	-1.989643	2.185931

SCF Energy: -1104.39073278

Sum of electronic and zero-point Energies= -1104.164584

Sum of electronic and thermal Energies= -1104.146558

Sum of electronic and thermal Enthalpies= -1104.145613

Sum of electronic and thermal Free Energies= -1104.211372

==> ./AOX-I/water/PA/C7/RAF/C7 <==

O	-0.499107	-0.662272	-0.034209
O	-2.189349	2.521334	0.006146
O	2.927609	2.728480	-0.114033
O	0.347280	3.298195	-0.024243
O	3.774577	-1.675450	-1.237021
C	1.167764	1.066230	-0.062548
C	0.809212	-0.284606	-0.076486
C	-1.501009	0.247119	0.005472
C	0.142920	2.057840	-0.019684
C	-1.217829	1.577127	0.009013
C	-2.830972	-0.369755	0.003802
C	2.585687	1.402481	-0.110078
C	1.739351	-1.296880	-0.127415
C	3.207765	-1.006575	-0.222184
C	3.530667	0.454010	-0.165295
C	-3.006531	-1.604248	-0.631021
C	-3.915807	0.241997	0.638842
C	-4.254992	-2.208278	-0.642265
C	-5.159919	-0.373959	0.628885
C	-5.334265	-1.594966	-0.013560
H	1.424202	-2.330227	-0.132726
H	-2.170143	-2.080782	-1.121947
H	-3.784755	1.181120	1.153655
H	-4.385364	-3.157771	-1.142887
H	-5.993126	0.099975	1.129113
H	-6.306389	-2.068738	-0.021477
H	-1.734840	3.379732	-0.027490
H	2.101969	3.253362	-0.084297
H	4.576476	0.725011	-0.224163
O	3.801177	-1.510929	1.084801
O	3.889406	-2.930512	0.965595
H	4.086964	-2.997675	0.003057

SCF Energy: -1104.39462230

Sum of electronic and zero-point Energies= -1104.167730

Sum of electronic and thermal Energies= -1104.149691

Sum of electronic and thermal Enthalpies= -1104.148747

Sum of electronic and thermal Free Energies= -1104.214802

==> ./AOX-I/water/PA/C7/PA/C5 <==

O	-0.162462	-0.862019	0.100295

O	-1.289997	2.564288	-0.146089
O	3.866386	1.781515	-0.122262
O	1.258731	2.923148	-0.165127
O	4.033345	-2.926342	0.173174
C	1.791981	0.579488	-0.014106
C	1.201913	-0.710324	0.071284
C	-1.011462	0.206000	0.034457
C	0.937101	1.710408	-0.074200
C	-0.508808	1.450459	-0.045920
C	-2.424708	-0.194981	0.022821
C	3.252342	0.650643	-0.041111
C	1.916126	-1.877942	0.133890
C	3.343297	-1.833059	0.112935
C	3.957726	-0.562185	0.024356
C	-2.774761	-1.455736	-0.473801
C	-3.428638	0.649909	0.507048
C	-4.103812	-1.854814	-0.499052
C	-4.756016	0.241847	0.481668
C	-5.099475	-1.007425	-0.023722
H	1.400597	-2.826000	0.200020
H	5.040449	-0.515597	0.006334
H	-2.006888	-2.117691	-0.847302
H	-3.172955	1.614438	0.917192
H	-4.361467	-2.828868	-0.892244
H	-5.522824	0.901328	0.864750
H	-6.134528	-1.320297	-0.043142
H	-0.647241	3.294229	-0.224824

SCF Energy: -953.000866389
Sum of electronic and zero-point Energies= -952.804005
Sum of electronic and thermal Energies= -952.788845
Sum of electronic and thermal Enthalpies= -952.787901
Sum of electronic and thermal Free Energies= -952.847343

==> ./AOX-I/water/PA/C7/PA/C3/aIP <==

O	-0.230642	-0.854524	0.070581
O	-1.252199	2.529756	-0.240544
O	3.806766	1.757582	-0.060181
O	1.433508	2.864500	-0.124076
O	3.955652	-2.949101	0.150900
C	1.746055	0.532204	-0.006925
C	1.142273	-0.743522	0.051127
C	-1.043689	0.204503	0.006444
C	0.967984	1.705147	-0.070788
C	-0.524527	1.531233	-0.100858
C	-2.453215	-0.147660	0.010956
C	3.177853	0.559058	-0.006049
C	1.843272	-1.908244	0.103565
C	3.281580	-1.875145	0.102121
C	3.914580	-0.591052	0.045326
C	-2.833946	-1.422959	-0.436823
C	-3.434815	0.739985	0.476329
C	-4.168996	-1.790149	-0.439503
C	-4.767131	0.355792	0.480715
C	-5.139187	-0.902589	0.018468
H	1.328520	-2.857316	0.149810
H	4.994823	-0.538683	0.043384
H	-2.083669	-2.111165	-0.797716
H	-3.152892	1.709629	0.852854
H	-4.454493	-2.768835	-0.799427
H	-5.517064	1.040378	0.851954
H	-6.180849	-1.193002	0.018986
H	3.112402	2.453673	-0.097906

SCF Energy: -952.840683766
Sum of electronic and zero-point Energies= -952.642988
Sum of electronic and thermal Energies= -952.627949
Sum of electronic and thermal Enthalpies= -952.627005
Sum of electronic and thermal Free Energies= -952.686596

==> ./AOX-I/water/PA/C7/PA/C3/aIP/vEA <==

O	-0.230642	-0.854524	0.070581
O	-1.252199	2.529756	-0.240544
O	3.806766	1.757582	-0.060181
O	1.433508	2.864500	-0.124076
O	3.955652	-2.949101	0.150900
C	1.746055	0.532204	-0.006925
C	1.142273	-0.743522	0.051127
C	-1.043689	0.204503	0.006444
C	0.967984	1.705147	-0.070788
C	-0.524527	1.531233	-0.100858
C	-2.453215	-0.147660	0.010956
C	3.177853	0.559058	-0.006049
C	1.843272	-1.908244	0.103565
C	3.281580	-1.875145	0.102121
C	3.914580	-0.591052	0.045326
C	-2.833946	-1.422959	-0.436823
C	-3.434815	0.739985	0.476329
C	-4.168996	-1.790149	-0.439503
C	-4.767131	0.355792	0.480715
C	-5.139187	-0.902589	0.018468
H	1.328520	-2.857316	0.149810
H	4.994823	-0.538683	0.043384
H	-2.083669	-2.111165	-0.797716
H	-3.152892	1.709629	0.852854
H	-4.454493	-2.768835	-0.799427
H	-5.517064	1.040378	0.851954
H	-6.180849	-1.193002	0.018986

H 3.112402 2.453673 -0.097906

SCF Energy: -952.998319798

==> ./AOX-I/water/PA/C7/PA/C3/HAT/C5/TS <==

O	0.790114	0.858708	-0.108185
O	-3.402370	-1.370893	-0.714072
O	-0.973989	-2.750316	-0.579027
O	-3.181138	3.311233	-0.376223
C	-1.218818	-0.391812	-0.420628
C	-0.540576	0.831097	-0.257704
C	1.573735	-0.283855	-0.107157
C	2.986799	0.050280	0.071857
C	-0.468789	-1.622462	-0.436476
C	1.018738	-1.534492	-0.275338
C	-2.634959	-0.288280	-0.576513
C	-1.162760	2.077482	-0.229109
C	-2.553801	2.191839	-0.385167
C	-3.267464	0.955785	-0.569850
C	3.977997	-0.932685	0.238974
C	3.390820	1.398246	0.092743
C	5.309237	-0.574992	0.415762
C	4.722902	1.743425	0.266925
C	5.694648	0.760474	0.429810
H	-0.553591	2.961231	-0.096443
H	-4.334525	0.989096	-0.738868
H	3.696967	-1.971671	0.233060
H	2.658187	2.181071	-0.030679
H	6.050310	-1.352949	0.544503
H	5.001850	2.788847	0.275442
H	-4.078030	-1.419557	0.121098
O	-4.669654	-1.297687	1.329963
O	-4.324925	-0.032337	1.677003
H	-3.499820	-0.108988	2.185785
O	1.681939	-2.641127	-0.305561
H	6.732578	1.031974	0.566139

SCF Energy: -1103.91632395
Sum of electronic and zero-point Energies= -1103.707754
Sum of electronic and thermal Energies= -1103.689621
Sum of electronic and thermal Enthalpies= -1103.688676
Sum of electronic and thermal Free Energies= -1103.756130

==> ./AOX-I/water/PA/C7/PA/C3/HAT/C5 <==

O	-0.191246	-0.807919	0.091267
O	-1.271005	2.539916	-0.233791
O	3.907916	1.745253	-0.086350
O	1.353727	2.959424	-0.103118
O	3.942408	-2.959286	0.145355
C	1.793630	0.613362	-0.008000
C	1.185520	-0.668966	0.059153
C	-1.024997	0.229121	0.018175
C	0.979849	1.771340	-0.059977
C	-0.521215	1.555303	-0.092282
C	-2.430295	-0.151074	0.014985
C	3.263846	0.641046	-0.022718
C	1.861453	-1.847559	0.111073
C	3.303442	-1.844120	0.097358
C	3.943826	-0.593583	0.030764
C	-2.783195	-1.438911	-0.418432
C	-3.435825	0.721798	0.455798
C	-4.111260	-1.833108	-0.430581
C	-4.761249	0.312665	0.449034
C	-5.104901	-0.959001	0.001601
H	1.322219	-2.782929	0.165651
H	5.026888	-0.567594	0.018327
H	-2.016116	-2.118387	-0.760288
H	-3.177697	1.702825	0.819357
H	-4.372373	-2.822930	-0.778807
H	-5.528282	0.989286	0.799798
H	-6.140812	-1.269254	-0.006661

SCF Energy: -952.366401641
Sum of electronic and zero-point Energies= -952.181535
Sum of electronic and thermal Energies= -952.166630
Sum of electronic and thermal Enthalpies= -952.165685
Sum of electronic and thermal Free Energies= -952.225294

==> ./AOX-I/water/PA/C7/PA/C3/HAT/C5/vEA <==

O	-0.191246	-0.807919	0.091267
O	-1.271005	2.539916	-0.233791
O	3.907916	1.745253	-0.086350
O	1.353727	2.959424	-0.103118
O	3.942408	-2.959286	0.145355
C	1.793630	0.613362	-0.008000
C	1.185520	-0.668966	0.059153
C	-1.024997	0.229121	0.018175
C	0.979849	1.771340	-0.059977
C	-0.521215	1.555303	-0.092282
C	-2.430295	-0.151074	0.014985
C	3.263846	0.641046	-0.022718
C	1.861453	-1.847559	0.111073
C	3.303442	-1.844120	0.097358
C	3.943826	-0.593583	0.030764
C	-2.783195	-1.438911	-0.418432

C	-3.435825	0.721798	0.455798
C	-4.111260	-1.833108	-0.430581
C	-4.761249	0.312665	0.449034
C	-5.104901	-0.959001	0.001601
H	1.322219	-2.782929	0.165651
H	5.026888	-0.567594	0.018327
H	-2.016116	-2.118387	-0.760288
H	-3.177697	1.702825	0.819357
H	-4.372373	-2.822930	-0.778807
H	-5.528282	0.989286	0.799798
H	-6.140812	-1.269254	-0.006661

SCF Energy: -952.513461221

==> ./AOX-I/water/PA/C7/PA/C3/vIP <==

O	-0.208933	-0.852777	0.057950
O	-1.256904	2.626576	-0.117491
O	3.778489	1.775656	-0.080243
O	1.437779	2.849000	-0.123948
O	3.997453	-2.944191	0.141547
C	1.733972	0.520834	-0.014336
C	1.136558	-0.751261	0.045591
C	-1.036216	0.260825	0.015872
C	0.932009	1.692885	-0.063297
C	-0.542411	1.546341	-0.050035
C	-2.445194	-0.137907	0.007699
C	3.160878	0.562643	-0.021236
C	1.867258	-1.921151	0.096634
C	3.285816	-1.873762	0.091556
C	3.907748	-0.581822	0.028971
C	-2.799359	-1.449784	-0.350499
C	-3.472309	0.748282	0.367887
C	-4.127288	-1.851897	-0.359956
C	-4.798921	0.335201	0.360896
C	-5.137521	-0.962759	-0.005499
H	1.358702	-2.874170	0.144268
H	4.988044	-0.519501	0.022759
H	-2.030989	-2.154837	-0.630641
H	-3.223910	1.754292	0.661972
H	-4.373092	-2.865810	-0.646752
H	-5.572050	1.035174	0.649023
H	-6.171933	-1.278322	-0.012443
H	3.052032	2.449059	-0.112195

SCF Energy: -952.831576738

==> ./AOX-I/water/PA/C7/PA/C3 <==

O	-0.208933	-0.852777	0.057950
O	-1.256904	2.626576	-0.117491
O	3.778489	1.775656	-0.080243
O	1.437779	2.849000	-0.123948
O	3.997453	-2.944191	0.141547
C	1.733972	0.520834	-0.014336
C	1.136558	-0.751261	0.045591
C	-1.036216	0.260825	0.015872
C	0.932009	1.692885	-0.063297
C	-0.542411	1.546341	-0.050035
C	-2.445194	-0.137907	0.007699
C	3.160878	0.562643	-0.021236
C	1.867258	-1.921151	0.096634
C	3.285816	-1.873762	0.091556
C	3.907748	-0.581822	0.028971
C	-2.799359	-1.449784	-0.350499
C	-3.472309	0.748282	0.367887
C	-4.127288	-1.851897	-0.359956
C	-4.798921	0.335201	0.360896
C	-5.137521	-0.962759	-0.005499
H	1.358702	-2.874170	0.144268
H	4.988044	-0.519501	0.022759
H	-2.030989	-2.154837	-0.630641
H	-3.223910	1.754292	0.661972
H	-4.373092	-2.865810	-0.646752
H	-5.572050	1.035174	0.649023
H	-6.171933	-1.278322	-0.012443
H	3.052032	2.449059	-0.112195

SCF Energy: -953.007806794
Sum of electronic and zero-point Energies= -952.810962
Sum of electronic and thermal Energies= -952.796078
Sum of electronic and thermal Enthalpies= -952.795133
Sum of electronic and thermal Free Energies= -952.853602

==> ./AOX-I/water/PA/C7/PA/C3/RAF/OOH/C4a/TS <==

O	0.378318	-0.909359	0.093255
O	1.360378	2.455294	-0.874828
O	-3.642375	1.599443	-0.582448
O	-1.333286	2.690030	-0.647640
O	-3.737806	-3.101543	-0.302665
C	-1.608439	0.423422	-0.071830
C	-0.967162	-0.853766	-0.034711
C	1.176879	0.182685	-0.198452
C	-0.798158	1.576523	-0.414410
C	0.659432	1.423030	-0.515656
C	2.594257	-0.177758	-0.146267
C	-3.024259	0.409830	-0.409705
C	-1.658198	-2.027261	-0.063101
C	-3.079315	-2.021739	-0.269974
C	-3.725085	-0.749789	-0.475826
C	2.979021	-1.522873	-0.265920

C	3.593551	0.786428	0.054130
C	4.316735	-1.886012	-0.203688
C	4.929401	0.413155	0.122497
C	5.300407	-0.920960	-0.010362
H	-1.138677	-2.972013	0.016833
H	-4.785518	-0.731973	-0.688175
H	2.227724	-2.284062	-0.415816
H	3.315917	1.821981	0.164340
H	4.591081	-2.927381	-0.306839
H	5.684246	1.171161	0.283993
H	6.342580	-1.205859	0.039346
H	-2.925949	2.281035	-0.617701
O	-1.959024	0.756464	1.848996
O	-0.649567	0.684621	2.423741
H	-0.778913	1.118018	3.276381

SCF Energy: -1103.92026974

==> ./AOX-I/water/PA/C7/PA/C3/RAF/OOH/C4a <==

O	0.442666	-0.906073	-0.054398
O	1.402848	2.538799	-0.734897
O	-3.512692	1.761667	-0.069316
O	-1.322765	2.753559	-0.475145
O	-3.711278	-2.864269	-0.945366
C	-1.519731	0.464166	0.204123
C	-0.892468	-0.823625	-0.162225
C	1.258677	0.221096	-0.194432
C	-0.726942	1.613163	-0.307852
C	0.701139	1.477541	-0.442182
C	2.661392	-0.133702	-0.096560
C	-2.975983	0.540131	-0.165341
C	-1.607450	-1.923489	-0.492809
C	-3.042274	-1.846421	-0.645256
C	-3.682148	-0.552137	-0.517010
C	3.042002	-1.484749	0.054891
C	3.691655	0.829393	-0.127228
C	4.376093	-1.847388	0.161304
C	5.022878	0.452029	-0.017557
C	5.381598	-0.885168	0.124710
H	-1.111424	-2.860583	-0.705823
H	-4.737358	-0.475412	-0.742506
H	2.286537	-2.254612	0.088604
H	3.434431	1.869177	-0.234455
H	4.630962	-2.893182	0.273802
H	5.788723	1.216465	-0.043101
H	6.421142	-1.171471	0.207212
H	-2.689679	2.399861	-0.192600
O	-1.422988	0.639247	1.707410
O	-2.199420	-0.344830	2.390560
H	-1.564922	-1.060079	2.538643

SCF Energy: -1103.92142920
Sum of electronic and zero-point Energies= -1103.708888
Sum of electronic and thermal Energies= -1103.691090
Sum of electronic and thermal Enthalpies= -1103.690146
Sum of electronic and thermal Free Energies= -1103.755439

==> ./AOX-I/water/PA/C7/PA/C3/RAF/OOH/C6 <==

O	-0.741737	-0.781987	-0.069592
O	-1.993384	2.623017	-0.041075
O	3.090950	2.053052	-0.439933
O	0.690451	3.003233	-0.241974
O	3.523340	-2.672412	-0.544926
C	1.112271	0.694236	-0.201461
C	0.578913	-0.607948	-0.174936
C	-1.639013	0.272964	-0.010064
C	0.239685	1.816640	-0.174000
C	-1.213815	1.588953	-0.064645
C	-3.016223	-0.211020	0.065509
C	2.508884	0.825780	-0.322284
C	1.367549	-1.759585	-0.269892
C	2.756396	-1.674837	-0.385692
C	3.427125	-0.317238	-0.255326
C	-3.305141	-1.557960	-0.219877
C	-4.084318	0.626093	0.430578
C	-4.604298	-2.039569	-0.152726
C	-5.381483	0.132608	0.498116
C	-5.654305	-1.198870	0.205160
H	0.887089	-2.728071	-0.298659
H	-2.508495	-2.229474	-0.502878
H	-3.889013	1.658121	0.667984
H	-4.796582	-3.079084	-0.383480
H	-6.184297	0.798439	0.786445
H	-6.666416	-1.576502	0.256561
H	2.338912	2.699220	-0.397871
H	4.246208	-0.222241	-0.971576
O	4.071175	-0.243103	1.082162
O	5.370091	-0.829570	1.035091
H	5.932136	-0.079726	0.794673

SCF Energy: -1103.94083142
Sum of electronic and zero-point Energies= -1103.726253
Sum of electronic and thermal Energies= -1103.708193
Sum of electronic and thermal Enthalpies= -1103.707249
Sum of electronic and thermal Free Energies= -1103.773685

==> ./AOX-I/water/PA/C7/PA/C3/RAF/OOH/C8/TS <==
O -0.368584 -0.538368 -0.175340
O -1.887166 2.726039 0.231906
O 3.211050 2.615338 -0.066325
O 0.753088 3.339115 0.195816
O 4.047581 -1.990552 -0.742201
C 1.366259 1.082799 -0.070262
C 0.937426 -0.234064 -0.204871
C -1.335659 0.431552 -0.022694
C 0.409068 2.131333 0.089266
C -1.024261 1.773587 0.114020
C -2.680700 -0.146402 -0.075628
C 2.771285 1.339223 -0.181070
C 1.830387 -1.333133 -0.315096
C 3.241740 -1.019994 -0.535032
C 3.669669 0.325045 -0.411574
C -2.883296 -1.384482 -0.705598
C -3.784298 0.493872 0.506281
C -4.147943 -1.952270 -0.763496
C -5.046141 -0.084656 0.448516
C -5.238026 -1.305598 -0.188670
H -2.048545 -1.898026 -1.159886
H -3.646266 1.434758 1.013096
H -4.282007 -2.903111 -1.261787
H -5.882939 0.422526 0.910051
H -6.223222 -1.749439 -0.234543
H 2.403692 3.176923 0.062034
H 4.722203 0.553364 -0.511603
H 1.450997 -2.232010 -0.786211
O 1.941761 -2.075895 1.330475
O 2.512842 -3.368271 1.133182
H 3.308461 -3.165106 0.604181

SCF Energy: -1103.93855478
Sum of electronic and zero-point Energies= -1103.724987
Sum of electronic and thermal Energies= -1103.707329
Sum of electronic and thermal Enthalpies= -1103.706385
Sum of electronic and thermal Free Energies= -1103.772246

==> ./AOX-I/water/PA/C7/PA/C3/RAF/OOH/C8 <==
O -0.354219 -0.526235 -0.117625
O -1.860455 2.754213 0.156718
O 3.270511 2.616632 -0.072672
O 0.781372 3.352513 0.126961
O 4.086176 -2.050313 -0.526357
C 1.400962 1.086114 -0.047646
C 0.955863 -0.225425 -0.122263
C -1.314349 0.449552 -0.014094
C 0.434798 2.145320 0.061870
C -0.996855 1.793955 0.082429
C -2.663832 -0.120861 -0.065938
C 2.797707 1.335009 -0.148542
C 1.840264 -1.394854 -0.204628
C 3.288747 -1.038677 -0.393652
C 3.701684 0.289749 -0.327588
C -2.867067 -1.373864 -0.664766
C -3.768664 0.541343 0.487397
C -4.134324 -1.936150 -0.719837
C -5.033339 -0.031649 0.433354
C -5.225849 -1.268175 -0.172727
H -2.030498 -1.903486 -1.096972
H -3.629197 1.495187 0.969088
H -4.269484 -2.899075 -1.194027
H -5.871669 0.492180 0.872988
H -6.213114 -1.707679 -0.216200
H 2.477385 3.193474 0.023005
H 4.757091 0.515108 -0.413428
H 1.500282 -2.119882 -0.950183
O 1.738328 -2.128837 1.090591
O 2.292583 -3.437339 0.897881
H 3.150356 -3.219218 0.461539

SCF Energy: -1103.94349974
Sum of electronic and zero-point Energies= -1103.728594
Sum of electronic and thermal Energies= -1103.710940
Sum of electronic and thermal Enthalpies= -1103.709996
Sum of electronic and thermal Free Energies= -1103.775940

==> ./AOX-I/water/PA/C7/PA/C3/RAF/OOH/C1p <==
O -0.063003 0.795456 -0.220992
O 0.926192 -2.686820 0.113809
O -4.096393 -1.715503 0.320618
O -1.780047 -2.850091 0.299228
O -4.219112 2.983437 -0.190757
C -2.028503 -0.524640 0.055957
C -1.408849 0.726831 -0.123829
C 0.723167 -0.336530 -0.137871
C -1.255109 -1.714303 0.139479
C 0.221627 -1.602967 0.036351
C 2.195499 -0.011380 -0.243041
C -3.453892 -0.526777 0.148227
C -2.114339 1.909525 -0.208612
C -3.530527 1.900380 -0.114823
C -4.175842 0.631422 0.068297

C	2.454066	1.438022	-0.515374
C	2.930233	-0.522574	0.956396
C	3.327806	2.171240	0.219907
C	3.770773	0.263979	1.682628
C	4.000878	1.611733	1.332857
H	1.944370	1.867248	-1.366594
H	2.751982	-1.552910	1.224475
H	3.510030	3.204694	-0.043975
H	4.271498	-0.151339	2.547340
H	4.683444	2.215379	1.911866
H	-3.389306	-2.407387	0.352512
H	-5.254889	0.597829	0.142179
H	-1.587770	2.843684	-0.346949
O	2.601181	-0.770064	-1.454567
O	4.008542	-0.664125	-1.626940
H	4.109839	0.160726	-2.123337

SCF Energy: -1103.92822104
Sum of electronic and zero-point Energies= -1103.714906
Sum of electronic and thermal Energies= -1103.697122
Sum of electronic and thermal Enthalpies= -1103.696178
Sum of electronic and thermal Free Energies= -1103.761208

==> ./AOX-I/water/PA/C7/PA/C3/RAF/OOH/C2/TS <==

O	-0.035731	-1.006379	-0.250756
O	-1.184308	2.374081	-0.348415
O	3.900060	1.750039	-0.274402
O	1.501801	2.766452	-0.432444
O	4.218081	-2.938680	0.137900
C	1.884192	0.448143	-0.260608
C	1.323267	-0.844379	-0.177317
C	-0.881398	0.042235	-0.287328
C	1.062456	1.594553	-0.361895
C	-0.415569	1.378197	-0.360262
C	-2.282616	-0.363461	-0.335924
C	3.310014	0.529057	-0.202383
C	2.070113	-1.981928	-0.051598
C	3.497557	-1.894244	0.010278
C	4.085297	-0.590194	-0.074861
C	-3.244782	0.389580	-1.017694
C	-2.664455	-1.551532	0.301900
C	-4.565041	-0.040085	-1.052732
C	-3.985901	-1.967624	0.269694
C	-4.940795	-1.212784	-0.407051
H	1.587559	-2.947490	0.004087
H	5.162053	-0.496169	-0.031850
H	-2.958086	1.294155	-1.530113
H	-1.925968	-2.132714	0.835554
H	-5.301014	0.543043	-1.589157
H	-4.272958	-2.879325	0.775511
H	-5.971542	-1.539240	-0.431939
H	3.178148	2.415321	-0.353946
O	-0.710193	0.600806	2.056471
O	-1.844669	1.460674	2.223959
H	-1.772551	2.073276	1.470042

SCF Energy: -1103.95387767
Sum of electronic and zero-point Energies= -1103.740878
Sum of electronic and thermal Energies= -1103.722791
Sum of electronic and thermal Enthalpies= -1103.721847
Sum of electronic and thermal Free Energies= -1103.788201

==> ./AOX-I/water/PA/C7/PA/C3/RAF/OOH/C2 <==

O	-0.082576	-0.902307	-0.272863
O	-1.333516	2.436822	0.067653
O	3.757927	1.963039	-0.183210
O	1.390556	2.930288	-0.296322
O	4.262205	-2.747889	-0.241816
C	1.798906	0.590587	-0.169892
C	1.284731	-0.715870	-0.198085
C	-0.896940	0.072667	0.342831
C	0.925047	1.739209	-0.158623
C	-0.471004	1.511726	0.039135
C	-2.324180	-0.203592	-0.067521
C	3.209364	0.713382	-0.186474
C	2.075716	-1.836218	-0.219609
C	3.498553	-1.709032	-0.222701
C	4.034613	-0.389532	-0.205062
C	-2.599420	-0.668189	-1.350205
C	-3.371182	0.076298	0.806360
C	-3.916578	-0.864737	-1.751880
C	-4.685977	-0.122467	0.403177
C	-4.961846	-0.594162	-0.876294
H	1.623836	-2.818664	-0.243633
H	5.108222	-0.253916	-0.213997
H	-1.789006	-0.874123	-2.035025
H	-3.158759	0.444137	1.800401
H	-4.123313	-1.228436	-2.749281
H	-5.494659	0.091759	1.088695
H	-5.985834	-0.748408	-1.188665
H	2.983519	2.590337	-0.232408
O	-0.716520	-0.005750	1.761962
O	-1.090786	-1.300142	2.227071
H	-0.261152	-1.795586	2.163163

SCF Energy: -1103.99110640

Sum of electronic and zero-point Energies= -1103.774930
Sum of electronic and thermal Energies= -1103.757646
Sum of electronic and thermal Enthalpies= -1103.756701
Sum of electronic and thermal Free Energies= -1103.820907

==> ./AOX-I/water/PA/C7/PA/C3/RAF/OOH/C6p <==

O	-0.358930	1.045430	0.048928
O	1.075999	-2.257220	-0.303556
O	-4.007034	-2.046773	-0.200765
O	-1.555911	-2.827428	-0.337477
O	-4.787281	2.583661	0.340305
C	-2.130063	-0.559370	-0.083014
C	-1.689104	0.768638	0.057753
C	0.617313	0.074606	-0.096552
C	-1.194055	-1.620329	-0.217478
C	0.243466	-1.298125	-0.208362
C	1.911451	0.606994	-0.134339
C	-3.539611	-0.774843	-0.070308
C	-2.549703	1.831202	0.202430
C	-3.957608	1.612646	0.211690
C	-4.419938	0.263984	0.070069
C	2.131707	2.005964	0.027068
C	3.107709	-0.286488	-0.275575
C	3.362597	2.567747	-0.153196
C	4.353881	0.424320	-0.696686
C	4.474995	1.758877	-0.582348
H	1.292481	2.639778	0.272353
H	3.496898	3.632618	-0.025989
H	5.406903	2.245834	-0.840661
H	-3.205345	-2.624744	-0.279029
H	-5.484416	0.070260	0.075550
H	-2.160098	2.834566	0.305510
H	5.176742	-0.183108	-1.049998
H	2.900012	-1.143024	-0.906497
O	3.304549	-0.845387	1.082718
O	4.223590	-1.929242	0.992209
H	5.078734	-1.511448	1.162968

SCF Energy: -1103.94821951
Sum of electronic and zero-point Energies= -1103.733891
Sum of electronic and thermal Energies= -1103.716048
Sum of electronic and thermal Enthalpies= -1103.715104
Sum of electronic and thermal Free Energies= -1103.780807

==> ./AOX-I/water/PA/C7/PA/C3/RAF/OOH/C6p/TS <==

O	-0.329723	1.026642	-0.100887
O	1.001522	-2.276320	-0.675186
O	-4.051275	-1.993828	-0.089721
O	-1.617672	-2.814362	-0.450546
O	-4.683277	2.639790	0.555549
C	-2.136427	-0.546519	-0.110817
C	-1.658098	0.775238	0.004245
C	0.609987	0.039029	-0.320295
C	-1.243184	-1.618639	-0.339513
C	0.215075	-1.310543	-0.457949
C	1.933884	0.555388	-0.392698
C	-3.548597	-0.734831	0.013259
C	-2.483181	1.850478	0.223141
C	-3.890841	1.657676	0.346947
C	-4.391675	0.317954	0.231464
C	2.166798	1.949104	-0.232821
C	3.073847	-0.295512	-0.528621
C	3.434740	2.467265	-0.300815
C	4.359582	0.279953	-0.694214
C	4.545846	1.628024	-0.552018
H	1.331178	2.612355	-0.071462
H	3.584465	3.531786	-0.181949
H	5.533419	2.059267	-0.646571
H	-3.281350	-2.592238	-0.244544
H	-5.455961	0.146309	0.321836
H	-2.067901	2.845328	0.304692
H	5.196488	-0.373432	-0.899503
H	2.925349	-1.320464	-0.812834
O	3.101973	-0.897647	1.456469
O	4.249651	-1.679107	1.680392
H	4.938177	-1.034654	1.886002

SCF Energy: -1103.93128849
Sum of electronic and zero-point Energies= -1103.719732
Sum of electronic and thermal Energies= -1103.701280
Sum of electronic and thermal Enthalpies= -1103.700336
Sum of electronic and thermal Free Energies= -1103.768553

==> ./AOX-I/water/PA/C7/PA/C3/RAF/OOH/C5 <==

O	-0.710879	0.893195	-0.026913
O	-1.551083	-2.620778	-0.252235
O	3.355740	-1.256213	-1.036846
O	1.155730	-2.673396	-0.413233
O	3.261072	3.366590	-0.317387
C	1.284453	-0.342514	-0.183162
C	0.621235	0.866127	-0.084250

C -1.485940 -0.255315 -0.061664
C 0.577103 -1.558499 -0.269846
C -0.908365 -1.507388 -0.194497
C -2.909397 0.065244 0.035204
C 2.781223 -0.327627 -0.155735
C 1.262606 2.121885 -0.070200
C 2.656091 2.227651 -0.249154
C 3.385976 1.011234 -0.379121
C -3.324797 1.409075 0.086762
C -3.902335 -0.928864 0.085467
C -4.668449 1.739313 0.181439
C -5.245667 -0.585735 0.182048
C -5.641684 0.745543 0.230173
H 0.657148 3.012982 0.020624
H 4.450805 1.049295 -0.557776
H -2.592133 2.200718 0.050514
H -3.615370 -1.965483 0.051041
H -4.955445 2.781940 0.217633
H -5.987519 -1.372607 0.220865
H -6.688779 1.005688 0.304462
H 2.784526 -2.049230 -0.975692
O 3.062798 -0.769255 1.218702
O 4.469073 -0.824932 1.420266
H 4.714841 -1.665771 1.008707

SCF Energy: -1103.94089488
Sum of electronic and zero-point Energies= -1103.726140
Sum of electronic and thermal Energies= -1103.708323
Sum of electronic and thermal Enthalpies= -1103.707379
Sum of electronic and thermal Free Energies= -1103.773075

==> ./AOX-I/water/PA/C7/PA/C3/RAF/OOH/C5/TS <==
O -0.701398 0.904586 -0.105743
O -1.561505 -2.618562 -0.232147
O 3.378181 -1.427977 -0.813167
O 1.144348 -2.682706 -0.439063
O 3.337787 3.280908 -0.419339
C 1.282270 -0.345670 -0.357376
C 0.630104 0.878163 -0.223525
C -1.477703 -0.246212 -0.108563
C 0.564894 -1.561611 -0.347070
C -0.913182 -1.503796 -0.228746
C -2.896405 0.077633 0.036684
C 2.727865 -0.317522 -0.402789
C 1.290310 2.110420 -0.222826
C 2.687713 2.175712 -0.383175
C 3.389787 0.926106 -0.534086
C -3.292731 1.405963 0.278361
C -3.902372 -0.899190 -0.064220
C -4.632194 1.737827 0.417194
C -5.241565 -0.554130 0.073248
C -5.618900 0.761414 0.315719
H 0.714519 3.018412 -0.109778
H 4.462874 0.933077 -0.663673
H -2.548145 2.182771 0.361777
H -3.626411 -1.922287 -0.253422
H -4.905379 2.767655 0.605745
H -5.995173 -1.325942 -0.012329
H -6.662762 1.023002 0.423242
H 2.719578 -2.166820 -0.727211
O 3.032545 -0.445670 1.502309
O 4.352993 -0.841751 1.702191
H 4.389955 -1.731150 1.322335

SCF Energy: -1103.92565873
Sum of electronic and zero-point Energies= -1103.712847
Sum of electronic and thermal Energies= -1103.695071
Sum of electronic and thermal Enthalpies= -1103.694127
Sum of electronic and thermal Free Energies= -1103.759933

==> ./AOX-I/water/PA/C7/PA/C3/RAF/OOH/C7 <==
O -0.533531 -0.635774 -0.041516
O -2.150116 2.540780 0.004197
O 2.916272 2.727005 -0.126909
O 0.438848 3.353392 0.025837
O 3.774582 -1.725570 -1.212659
C 1.144810 1.101065 -0.060657
C 0.796704 -0.281870 -0.090226
C -1.517437 0.276348 0.008476
C 0.179826 2.096909 0.001743
C -1.244233 1.673190 0.012692
C -2.845983 -0.324188 0.002625
C 2.570852 1.401817 -0.124478
C 1.693865 -1.283375 -0.161787
C 3.168844 -1.014797 -0.240204
C 3.505460 0.440983 -0.201126
C -3.013184 -1.601661 -0.555374
C -3.956609 0.322876 0.563642
C -4.260973 -2.204533 -0.568034
C -5.199754 -0.293926 0.554352
C -5.359693 -1.553343 -0.014327
H 1.367259 -2.314270 -0.187396
H -2.165921 -2.110383 -0.991693
H -3.840291 1.292154 1.019802
H -4.376881 -3.183758 -1.012085
H -6.046605 0.210922 0.998762

H	-6.332381	-2.025943	-0.023130
H	2.058590	3.232477	-0.068873
H	4.553354	0.702466	-0.274890
O	3.749688	-1.507674	1.102337
O	3.898705	-2.924785	0.971741
H	4.052316	-2.955624	-0.008618

SCF Energy: -1103.93445087
Sum of electronic and zero-point Energies= -1103.721140
Sum of electronic and thermal Energies= -1103.703489
Sum of electronic and thermal Enthalpies= -1103.702545
Sum of electronic and thermal Free Energies= -1103.768214

==> ./AOX-I/water/PA/C7/PA/C3/RAF/OOH/C5p <==

O	0.514194	-0.898828	-0.030708
O	-0.670977	2.526414	-0.284775
O	4.391002	1.894975	0.029432
O	2.009777	2.868114	-0.165769
O	4.790615	-2.804684	0.357171
C	2.398142	0.556599	-0.004463
C	1.851622	-0.738918	0.041709
C	-0.357470	0.175273	-0.144479
C	1.551639	1.692486	-0.116296
C	0.085867	1.481411	-0.181780
C	-1.745283	-0.282475	-0.246768
C	3.820196	0.658614	0.073335
C	2.625510	-1.876090	0.158836
C	4.038206	-1.768358	0.240723
C	4.608833	-0.452099	0.190352
C	-1.996639	-1.682657	-0.416423
C	-2.812044	0.579007	-0.202325
C	-3.309667	-2.170965	-0.563158
C	-4.222881	0.118166	-0.311522
C	-4.388447	-1.342535	-0.534814
H	-1.172474	-2.375153	-0.445758
H	-2.656399	1.635551	-0.071922
H	-3.451708	-3.233715	-0.709586
H	-5.391736	-1.725393	-0.661458
H	3.643177	2.538556	-0.055723
H	5.683939	-0.344322	0.248708
H	2.154773	-2.849007	0.190745
H	-4.757729	0.696469	-1.070969
O	-4.839435	0.515496	0.964986
O	-6.253525	0.448626	0.819047
H	-6.470553	-0.428646	1.163331

SCF Energy: -1103.93269898
Sum of electronic and zero-point Energies= -1103.719095
Sum of electronic and thermal Energies= -1103.701098
Sum of electronic and thermal Enthalpies= -1103.700154
Sum of electronic and thermal Free Energies= -1103.766713

==> ./AOX-I/water/PA/C7/PA/C3/RAF/OOH/C2p/TS <==

O	-0.015954	0.526314	-0.320024
O	0.537946	-2.981561	0.203593
O	-4.331241	-1.466819	0.329230
O	-2.139864	-2.865017	0.413293
O	-3.888752	3.159961	-0.478904
C	-2.136411	-0.547526	0.013505
C	-1.370881	0.610650	-0.238411
C	0.676791	-0.645859	-0.171518
C	-1.510656	-1.802048	0.183818
C	-0.018884	-1.861703	0.076330
C	2.080651	-0.481467	-0.311331
C	-3.554982	-0.378285	0.088746
C	-1.925993	1.851432	-0.406653
C	-3.343818	2.015681	-0.329288
C	-4.136173	0.847162	-0.075459
C	2.594919	0.825928	-0.578127
C	3.016241	-1.535655	-0.118160
C	3.969108	0.991398	-0.868540
C	4.359692	-1.318065	-0.290732
C	4.840922	-0.051557	-0.695682
H	1.913709	1.606898	-0.869897
H	2.661839	-2.514439	0.153406
H	4.323842	1.960659	-1.190548
H	5.055719	-2.132160	-0.141621
H	5.897993	0.083324	-0.883502
H	-3.721805	-2.237398	0.415506
H	-5.211534	0.946552	-0.013026
H	-1.295108	2.708457	-0.596629
O	2.444668	1.529905	1.399408
O	3.036928	2.824010	1.447034
H	3.982333	2.643231	1.506281

SCF Energy: -1103.93094223
Sum of electronic and zero-point Energies= -1103.718836
Sum of electronic and thermal Energies= -1103.700408
Sum of electronic and thermal Enthalpies= -1103.699464
Sum of electronic and thermal Free Energies= -1103.768000

==> ./AOX-I/water/PA/C7/PA/C3/RAF/OOH/C2p <==

O	0.000978	0.544922	-0.158182
O	0.559710	-3.012917	0.146376
O	-4.309020	-1.515029	0.160825
O	-2.139875	-2.897205	0.255390
O	-3.889417	3.165643	-0.352127
C	-2.117187	-0.556179	0.006381
C	-1.354908	0.616664	-0.139463
C	0.697248	-0.646060	-0.063703
C	-1.482065	-1.822732	0.121711
C	-0.013010	-1.877458	0.072421
C	2.083669	-0.475421	-0.131463
C	-3.535058	-0.403493	0.023856
C	-1.917674	1.865406	-0.261220
C	-3.334278	2.013784	-0.242183
C	-4.123144	0.826899	-0.095152
C	2.604431	0.927649	-0.283295
C	3.030883	-1.529452	0.016851
C	4.021493	1.010864	-0.747419
C	4.365095	-1.324212	-0.193504
C	4.848302	-0.045382	-0.647465
H	1.950266	1.540166	-0.898573
H	2.673559	-2.511289	0.276047
H	4.371933	1.963782	-1.120908
H	5.061867	-2.141787	-0.073863
H	5.887273	0.047809	-0.937862
H	-3.679195	-2.278353	0.227496
H	-5.201704	0.911471	-0.079266
H	-1.286038	2.736283	-0.370159
O	2.496053	1.490705	1.082489
O	2.763918	2.887616	1.016152
H	3.720472	2.936790	1.151572

SCF Energy: -1103.94843474
Sum of electronic and zero-point Energies= -1103.734238
Sum of electronic and thermal Energies= -1103.716263
Sum of electronic and thermal Enthalpies= -1103.715319
Sum of electronic and thermal Free Energies= -1103.781878

==> ./AOX-I/water/PA/C7/PA/C3/RAF/OOH/C4/TS <==

O	-0.267925	0.991771	-0.039540
O	-1.269850	-2.294340	-0.951309
O	3.773196	-1.559424	-0.510657
O	1.425456	-2.670477	-0.709404
O	3.915457	3.125084	0.145954
C	1.714207	-0.356031	-0.301742
C	1.109119	0.887994	-0.099774
C	-1.083017	-0.056551	-0.258376
C	0.925645	-1.556345	-0.403196
C	-0.557707	-1.360057	-0.555545
C	-2.492238	0.284599	-0.202893
C	3.129275	-0.371332	-0.331889
C	1.806655	2.059435	0.047492
C	3.233892	2.043249	0.009420
C	3.867217	0.780739	-0.189535
C	-2.886192	1.619507	-0.395664
C	-3.471904	-0.680722	0.080786
C	-4.224352	1.971288	-0.328364
C	-4.807385	-0.314957	0.154331
C	-5.190375	1.006488	-0.055197
H	1.280081	2.992125	0.194732
H	4.947560	0.731345	-0.224383
H	-2.142095	2.371889	-0.611917
H	-3.183919	-1.703913	0.260936
H	-4.515209	3.000138	-0.490222
H	-5.552177	-1.065032	0.382260
H	-6.234157	1.284037	-0.001001
H	3.068686	-2.243344	-0.616014
O	0.432511	-1.917102	1.604832
O	0.307792	-0.744325	2.418869
H	-0.638904	-0.560549	2.416533

SCF Energy: -1103.94815929
Sum of electronic and zero-point Energies= -1103.734904
Sum of electronic and thermal Energies= -1103.716840
Sum of electronic and thermal Enthalpies= -1103.715896
Sum of electronic and thermal Free Energies= -1103.782482

==> ./AOX-I/water/PA/C7/PA/C3/RAF/OOH/C4 <==

O	-0.318741	0.993791	0.236108
O	-1.253471	-2.220393	-0.951032
O	3.690594	-1.448623	-0.633044
O	1.478242	-2.536557	-0.918095
O	3.826389	3.253226	0.112192
C	1.676913	-0.288750	-0.155326
C	1.064573	0.928379	0.085051
C	-1.105601	-0.039032	-0.114496
C	0.925471	-1.577871	-0.163916
C	-0.558808	-1.320803	-0.453472
C	-2.522168	0.291946	-0.122229
C	3.071144	-0.268859	-0.346370
C	1.741257	2.125929	0.183402
C	3.155230	2.145633	0.024412
C	3.794936	0.906400	-0.256866
C	-2.910574	1.630316	-0.294013

C	-3.510338	-0.684779	0.074962
C	-4.252360	1.976332	-0.290240
C	-4.849956	-0.326312	0.085320
C	-5.226882	1.000174	-0.102256
H	1.200819	3.044800	0.365479
H	4.865789	0.887816	-0.414846
H	-2.158172	2.390561	-0.444673
H	-3.227979	-1.712245	0.240123
H	-4.538781	3.008941	-0.435167
H	-5.602766	-1.085611	0.246760
H	-6.273425	1.272546	-0.096854
H	2.896764	-2.093559	-0.807115
O	0.939828	-2.180062	1.230177
O	0.430873	-1.253210	2.183997
H	-0.468998	-1.570846	2.340086

SCF Energy: -1103.95832502
Sum of electronic and zero-point Energies= -1103.744111
Sum of electronic and thermal Energies= -1103.726616
Sum of electronic and thermal Enthalpies= -1103.725672
Sum of electronic and thermal Free Energies= -1103.790601

==> ./AOX-I/water/PA/C7/PA/C3/RAF/OOH/C8a <==

O	-0.246412	-0.640962	-0.468768
O	-1.337862	2.732436	0.392742
O	3.636918	1.998956	-0.189153
O	1.343816	3.042932	0.008849
O	3.950819	-2.663049	-0.782282
C	1.662576	0.723709	-0.030604
C	1.020905	-0.597473	0.114017
C	-1.114827	0.425683	-0.160729
C	0.857805	1.863276	0.030977
C	-0.628134	1.685480	0.106775
C	-2.512350	0.011667	-0.212300
C	3.062287	0.788388	-0.186425
C	1.838839	-1.719646	-0.411729
C	3.278307	-1.617775	-0.523798
C	3.861064	-0.337436	-0.366667
C	-2.834874	-1.355395	-0.314964
C	-3.578297	0.931246	-0.179417
C	-4.155062	-1.778838	-0.375474
C	-4.895816	0.495830	-0.240993
C	-5.198332	-0.858681	-0.337589
H	-2.044336	-2.089725	-0.341208
H	-3.362940	1.983925	-0.108916
H	-4.368618	-2.837173	-0.450126
H	-5.693749	1.226630	-0.214941
H	-6.226517	-1.190932	-0.383393
H	2.876202	2.657729	-0.093015
H	4.930451	-0.220121	-0.472341
H	1.358241	-2.665186	-0.617909
O	0.877367	-0.758793	1.570613
O	0.223976	-1.993254	1.856549
H	0.957747	-2.571497	2.106975

SCF Energy: -1103.93290897
Sum of electronic and zero-point Energies= -1103.719384
Sum of electronic and thermal Energies= -1103.701515
Sum of electronic and thermal Enthalpies= -1103.700571
Sum of electronic and thermal Free Energies= -1103.766590

==> ./AOX-I/water/PA/C7/PA/C3/RAF/OOH/C4p <==

O	0.461373	-0.790900	-0.152433
O	-0.391791	2.725165	-0.073211
O	4.571150	1.632087	0.244236
O	2.299588	2.834855	0.109986
O	4.542809	-3.095370	0.105837
C	2.474149	0.488384	0.042454
C	1.814481	-0.749778	-0.044277
C	-0.333445	0.342941	-0.187544
C	1.734071	1.703164	0.027600
C	0.270152	1.634405	-0.081040
C	-1.694929	0.050618	-0.315416
C	3.894801	0.453714	0.154266
C	2.478304	-1.953970	-0.026462
C	3.897932	-1.984891	0.085065
C	4.582961	-0.730318	0.173736
C	-2.138762	-1.326887	-0.365022
C	-2.723559	1.058529	-0.440956
C	-3.437949	-1.667766	-0.443296
C	-4.030463	0.741231	-0.529235
C	-4.531839	-0.657944	-0.424378
H	-1.396229	-2.110885	-0.358197
H	-2.424053	2.091348	-0.493681
H	-3.726836	-2.709332	-0.494739
H	-4.764790	1.525656	-0.660157
H	-5.294745	-0.874215	-1.174503
H	3.881415	2.344605	0.214483
H	5.661467	-0.724333	0.260355
H	1.923871	-2.879645	-0.096315
O	-5.204113	-0.875023	0.869928
O	-6.429704	-0.151058	0.866920
H	-6.190038	0.690167	1.279674

SCF Energy: -1103.95047804

Sum of electronic and zero-point Energies= -1103.736127
Sum of electronic and thermal Energies= -1103.718167
Sum of electronic and thermal Enthalpies= -1103.717222
Sum of electronic and thermal Free Energies= -1103.784287

==> ./AOX-I/water/PA/C7/PA/C3/RAF/OOH/C4p/TS <==

O	0.306952	-0.761537	-0.293746
O	-0.526883	2.747437	-0.085307
O	4.419165	1.608067	0.354908
O	2.146319	2.833084	0.181597
O	4.358252	-3.102038	0.012988
C	2.318531	0.493604	0.031835
C	1.651071	-0.737528	-0.132955
C	-0.472207	0.383007	-0.303553
C	1.595443	1.709633	0.040163
C	0.112185	1.652373	-0.121947
C	-1.853590	0.096471	-0.492161
C	3.738597	0.442168	0.191760
C	2.306930	-1.944917	-0.142714
C	3.722164	-1.991012	0.017540
C	4.414512	-0.745421	0.185475
C	-2.290622	-1.260505	-0.581230
C	-2.844118	1.111497	-0.629057
C	-3.611104	-1.572737	-0.751607
C	-4.165308	0.789428	-0.796733
C	-4.599276	-0.556848	-0.760433
H	-1.562252	-2.055042	-0.530118
H	-2.540877	2.143787	-0.621990
H	-3.914850	-2.608517	-0.822466
H	-4.897127	1.577595	-0.911591
H	-5.611727	-0.799766	-1.046740
H	3.745334	2.330768	0.329279
H	5.489191	-0.754687	0.309688
H	1.750840	-2.862964	-0.272472
O	-5.225515	-0.719533	1.213307
O	-4.105434	-0.468859	2.003105
H	-3.580635	-1.279418	1.945310

SCF Energy: -1103.93408843
Sum of electronic and zero-point Energies= -1103.722000
Sum of electronic and thermal Energies= -1103.703900
Sum of electronic and thermal Enthalpies= -1103.702956
Sum of electronic and thermal Free Energies= -1103.769591

==> ./AOX-I/water/PA/C7/PA/C3/RAF/OOH/C3p <==

O	-0.227308	0.579226	0.020528
O	0.087654	-3.038108	-0.177937
O	-4.672426	-1.179744	-0.014311
O	-2.603478	-2.713909	-0.086490
O	-3.920787	3.485115	0.117782
C	-2.412401	-0.371279	-0.001328
C	-1.566804	0.753914	0.026784
C	0.344169	-0.683302	-0.015884
C	-1.871468	-1.685213	-0.050412
C	-0.398141	-1.840560	-0.077925
C	1.808426	-0.602801	-0.042214
C	-3.817623	-0.119200	0.014104
C	-2.041399	2.049438	0.068282
C	-3.439713	2.293285	0.082578
C	-4.312770	1.154373	0.055426
C	2.432195	0.501419	-0.555050
C	2.593195	-1.666933	0.495918
C	3.911244	0.647541	-0.579926
C	4.001139	-1.611652	0.446739
C	4.655283	-0.541133	-0.080183
H	1.858802	1.320607	-0.963853
H	2.101507	-2.509335	0.950660
H	4.253329	0.940226	-1.576579
H	4.566791	-2.445830	0.840587
H	5.735385	-0.514489	-0.125015
H	-4.101381	-1.988753	-0.046781
H	-5.382876	1.314895	0.065723
H	-1.347469	2.878410	0.090202
O	4.185630	1.818156	0.271074
O	5.523338	2.241214	0.032973
H	6.022938	1.801286	0.734643

SCF Energy: -1103.93279684
Sum of electronic and zero-point Energies= -1103.719242
Sum of electronic and thermal Energies= -1103.701175
Sum of electronic and thermal Enthalpies= -1103.700231
Sum of electronic and thermal Free Energies= -1103.767264

==> ./AOX-I/water/PA/C7/PA/C3/RAF/OOH/C3/TS <==

O	-0.167949	-1.124044	0.121021
O	-1.219109	2.106787	-1.008867
O	3.830469	1.467592	-0.552809
O	1.446999	2.533457	-0.707925
O	4.052348	-3.133434	0.460602
C	1.784912	0.246321	-0.257843
C	1.194937	-1.000260	0.040090

C	-1.022373	-0.108753	-0.185867
C	0.984329	1.393570	-0.477339
C	-0.519770	1.238797	-0.431119
C	-2.408516	-0.494789	-0.155631
C	3.210637	0.287700	-0.286732
C	1.922404	-2.132079	0.281841
C	3.353003	-2.088237	0.239428
C	3.966906	-0.830256	-0.056431
C	-2.754975	-1.860382	-0.202407
C	-3.442620	0.455322	-0.041613
C	-4.082405	-2.253231	-0.159028
C	-4.766209	0.047350	0.006515
C	-5.096942	-1.304121	-0.056849
H	1.421263	-3.062533	0.509490
H	5.046196	-0.765427	-0.088414
H	-1.978392	-2.606099	-0.286367
H	-3.199108	1.502969	0.025623
H	-4.327827	-3.305634	-0.206023
H	-5.546204	0.790704	0.102107
H	-6.132203	-1.614292	-0.021878
H	3.126970	2.144770	-0.668381
O	-0.375768	1.733613	1.471536
O	-0.407014	3.157412	1.483375
H	0.369595	3.402767	0.956305

SCF Energy: -1103.95126517
Sum of electronic and zero-point Energies= -1103.738375
Sum of electronic and thermal Energies= -1103.720257
Sum of electronic and thermal Enthalpies= -1103.719313
Sum of electronic and thermal Free Energies= -1103.786233

==> ./AOX-I/water/PA/C7/PA/C3/RAF/OOH/C3 <==

O	0.235955	0.990014	0.458918
O	1.265424	-2.394490	-0.370951
O	-3.669968	-1.250220	-1.230054
O	-1.337262	-2.423651	-1.113792
O	-3.938198	3.123768	0.513744
C	-1.683144	-0.231120	-0.349946
C	-1.111318	0.927057	0.221743
C	1.079310	-0.035876	0.107815
C	-0.911404	-1.407569	-0.533144
C	0.513068	-1.436705	0.085190
C	2.434794	0.374632	-0.069418
C	-3.075070	-0.170799	-0.656987
C	-1.838430	2.045340	0.521260
C	-3.242788	2.087374	0.241766
C	-3.830213	0.935222	-0.368881
C	2.737154	1.757422	-0.144625
C	3.512592	-0.538492	-0.161927
C	4.039588	2.193410	-0.310596
C	4.809778	-0.083585	-0.327201
C	5.088522	1.280077	-0.405152
H	-1.351479	2.914581	0.941171
H	-4.885998	0.942810	-0.604069
H	1.939297	2.481507	-0.081260
H	3.312578	-1.593900	-0.096200
H	4.239557	3.255032	-0.369691
H	5.615644	-0.802671	-0.391711
H	6.104881	1.625153	-0.535000
H	-2.971664	-1.931146	-1.354291
O	0.219013	-1.576451	1.598091
O	-0.491231	-2.790829	1.828305
H	-1.407297	-2.494832	1.917593

SCF Energy: -1103.95450701
Sum of electronic and zero-point Energies= -1103.740011
Sum of electronic and thermal Energies= -1103.722252
Sum of electronic and thermal Enthalpies= -1103.721308
Sum of electronic and thermal Free Energies= -1103.786579

==> ./AOX-I/water/PA/C7/PA/C3/PA/C5 <==

O	-0.171760	-0.798217	0.025415
O	-1.270670	2.650992	-0.041250
O	3.916456	1.752824	-0.031706
O	1.362740	2.948109	-0.035961
O	3.970467	-2.962452	0.043421
C	1.790768	0.605259	-0.004273
C	1.179405	-0.672472	0.017316
C	-1.027474	0.293709	0.010098
C	0.951616	1.764449	-0.018081
C	-0.541193	1.577629	-0.014921
C	-2.426056	-0.134191	0.004308
C	3.256155	0.640192	-0.010601
C	1.874348	-1.863673	0.033153
C	3.295155	-1.851300	0.028099
C	3.934962	-0.589933	0.006017
C	-2.740634	-1.502515	-0.105259
C	-3.496950	0.771919	0.112551
C	-4.057802	-1.938493	-0.112582
C	-4.812438	0.323758	0.106879
C	-5.107047	-1.030308	-0.007133
H	1.334676	-2.800949	0.050674
H	5.018851	-0.563390	0.001461
H	-1.947996	-2.230002	-0.188993
H	-3.289298	1.823876	0.204201
H	-4.263952	-2.997148	-0.201665
H	-5.614011	1.045691	0.194259

H -6.133034 -1.372305 -0.012717

SCF Energy: -952.523181947
Sum of electronic and zero-point Energies= -952.339072
Sum of electronic and thermal Energies= -952.324309
Sum of electronic and thermal Enthalpies= -952.323365
Sum of electronic and thermal Free Energies= -952.381728

==> ./AOX-I/water/PA/C7 <==

O	-0.198570	-0.904164	0.081962
O	-1.296620	2.540593	-0.178149
O	3.774268	1.784559	-0.101687
O	1.341930	2.837450	-0.178594
O	4.042981	-2.921050	0.195153
C	1.743099	0.503566	-0.015361
C	1.161278	-0.778594	0.064357
C	-1.024314	0.181597	0.015393
C	0.920233	1.652980	-0.088423
C	-0.514246	1.429047	-0.071070
C	-2.444328	-0.192310	0.015405
C	3.166507	0.567889	-0.023822
C	1.899461	-1.933450	0.133049
C	3.322709	-1.865378	0.127665
C	3.926356	-0.565774	0.045144
C	-2.830690	-1.411097	-0.551885
C	-3.412463	0.638318	0.586750
C	-4.167431	-1.783240	-0.560508
C	-4.747411	0.255792	0.579315
C	-5.129360	-0.951160	0.003572
H	1.404497	-2.892305	0.195375
H	5.005573	-0.490250	0.038034
H	-2.086300	-2.058830	-0.992521
H	-3.122240	1.568299	1.051189
H	-4.457827	-2.723403	-1.009072
H	-5.488970	0.900546	1.030744
H	-6.170411	-1.244003	-0.002020
H	-0.681875	3.287591	-0.267199
H	3.069235	2.464140	-0.151486

SCF Energy: -953.478243123
Sum of electronic and zero-point Energies= -953.268471
Sum of electronic and thermal Energies= -953.253069
Sum of electronic and thermal Enthalpies= -953.252125
Sum of electronic and thermal Free Energies= -953.311724

==> ./AOX-I/water/PA/C3 <==

O	-0.226450	-0.831362	0.063051
O	-1.330037	2.626000	-0.119307
O	3.720766	1.840619	-0.085335
O	1.365371	2.883856	-0.125837
O	3.964629	-2.903537	0.137434
C	1.695511	0.560503	-0.012637
C	1.107718	-0.706785	0.049113
C	-1.068936	0.265733	0.016992
C	0.868757	1.732865	-0.063151
C	-0.597167	1.562829	-0.050075
C	-2.471859	-0.154774	0.007785
C	3.113637	0.634590	-0.024574
C	1.859599	-1.875573	0.100764
C	3.237271	-1.753314	0.087077
C	3.879274	-0.509015	0.024273
C	-2.804619	-1.467964	-0.362961
C	-3.508432	0.714535	0.378470
C	-4.126277	-1.889351	-0.373031
C	-4.828636	0.281500	0.371861
C	-5.147483	-1.017872	-0.006305
H	1.380506	-2.842039	0.149073
H	4.959094	-0.444711	0.012601
H	-2.025911	-2.157907	-0.652438
H	-3.273070	1.721607	0.679794
H	-4.358765	-2.903457	-0.669594
H	-5.611718	0.966474	0.668540
H	-6.177081	-1.348768	-0.013516
H	2.995956	2.515175	-0.116837
H	4.906589	-2.698449	0.120721

SCF Energy: -953.473300411
Sum of electronic and zero-point Energies= -953.263555
Sum of electronic and thermal Energies= -953.248303
Sum of electronic and thermal Enthalpies= -953.247359
Sum of electronic and thermal Free Energies= -953.306334

==> ./AOX-I/water/vIP <==

O	-0.214839	-0.882227	0.077729
O	-1.377255	2.536238	-0.179317
O	3.718376	1.846488	-0.106162
O	1.275382	2.870259	-0.181785
O	4.008839	-2.882371	0.198778
C	1.704816	0.542743	-0.018595
C	1.132833	-0.732628	0.061200
C	-1.055508	0.187947	0.012807
C	0.856685	1.695330	-0.091967
C	-0.567693	1.447712	-0.073973
C	-2.468677	-0.207498	0.014783

C	3.117843	0.639167	-0.028222
C	1.892282	-1.886779	0.133086
C	3.272290	-1.745560	0.123742
C	3.895881	-0.495548	0.042246
C	-2.839128	-1.415812	-0.583949
C	-3.441729	0.592813	0.619000
C	-4.169832	-1.807886	-0.591126
C	-4.770086	0.188642	0.614422
C	-5.137850	-1.007181	0.007076
H	1.425629	-2.858203	0.196578
H	4.974684	-0.417024	0.033744
H	-2.088387	-2.037703	-1.050573
H	-3.160324	1.514009	1.106081
H	-4.450892	-2.738465	-1.064674
H	-5.517466	0.807470	1.091563
H	-6.174223	-1.316083	0.002859
H	-0.792527	3.305806	-0.268934
H	3.017051	2.529938	-0.156361
H	4.949678	-2.669628	0.192590

SCF Energy: -953.705559966

==> ./AOX-I/water/aIP/vEA <==

O	-0.238376	-0.850108	0.079712
O	-1.326147	2.509234	-0.166678
O	3.761550	1.813760	-0.072853
O	1.284386	2.890956	-0.104143
O	3.940216	-2.909313	0.116050
C	1.721409	0.559848	-0.001867
C	1.131030	-0.711865	0.055068
C	-1.083173	0.163761	0.029221
C	0.902076	1.717598	-0.048287
C	-0.555532	1.473350	-0.051874
C	-2.472992	-0.201968	0.019575
C	3.137493	0.631671	-0.019173
C	1.853052	-1.870898	0.095826
C	3.252780	-1.756668	0.076486
C	3.896293	-0.524569	0.018585
C	-2.811080	-1.521859	-0.343099
C	-3.485756	0.708076	0.379211
C	-4.134431	-1.909763	-0.365892
C	-4.805430	0.297003	0.367501
C	-5.132639	-1.002670	-0.010141
H	1.370971	-2.835741	0.138948
H	4.976009	-0.465278	0.002203
H	-2.035130	-2.216991	-0.626202
H	-3.242807	1.708492	0.695914
H	-4.394970	-2.915858	-0.661359
H	-5.582377	0.989089	0.658323
H	-6.168442	-1.312331	-0.024930
H	-0.767410	3.314595	-0.203257
H	3.101459	2.533087	-0.098881
H	4.891075	-2.741606	0.095705

SCF Energy: -953.928647943

==> ./AOX-I/water/aIP <==

O	-0.238376	-0.850108	0.079712
O	-1.326147	2.509234	-0.166678
O	3.761550	1.813760	-0.072853
O	1.284386	2.890956	-0.104143
O	3.940216	-2.909313	0.116050
C	1.721409	0.559848	-0.001867
C	1.131030	-0.711865	0.055068
C	-1.083173	0.163761	0.029221
C	0.902076	1.717598	-0.048287
C	-0.555532	1.473350	-0.051874
C	-2.472992	-0.201968	0.019575
C	3.137493	0.631671	-0.019173
C	1.853052	-1.870898	0.095826
C	3.252780	-1.756668	0.076486
C	3.896293	-0.524569	0.018585
C	-2.811080	-1.521859	-0.343099
C	-3.485756	0.708076	0.379211
C	-4.134431	-1.909763	-0.365892
C	-4.805430	0.297003	0.367501
C	-5.132639	-1.002670	-0.010141
H	1.370971	-2.835741	0.138948
H	4.976009	-0.465278	0.002203
H	-2.035130	-2.216991	-0.626202
H	-3.242807	1.708492	0.695914
H	-4.394970	-2.915858	-0.661359
H	-5.582377	0.989089	0.658323
H	-6.168442	-1.312331	-0.024930
H	-0.767410	3.314595	-0.203257
H	3.101459	2.533087	-0.098881
H	4.891075	-2.741606	0.095705

SCF Energy: -953.716714414
Sum of electronic and zero-point Energies= -953.493354
Sum of electronic and thermal Energies= -953.477702
Sum of electronic and thermal Enthalpies= -953.476758
Sum of electronic and thermal Free Energies= -953.537675

==> ./AOX-I/water/HAT/C5 <==

O	-0.164803	-0.815641	0.093005
O	-1.398526	2.556175	-0.155239
O	3.785866	1.809326	-0.131723
O	1.193646	2.975992	-0.177449
O	3.987592	-2.899640	0.197274

C 1.722950 0.640570 -0.020699
C 1.159963 -0.615440 0.066291
C -1.042721 0.222473 0.028683
C 0.852339 1.789177 -0.085200
C -0.574189 1.491006 -0.056018
C -2.441484 -0.215571 0.021164
C 3.187667 0.726082 -0.047336
C 1.920967 -1.803992 0.139159
C 3.316083 -1.725029 0.120876
C 3.942213 -0.505741 0.028382
C -2.764231 -1.450700 -0.550610
C -3.449589 0.572238 0.583359
C -4.082159 -1.882515 -0.571789
C -4.764996 0.128457 0.563596
C -5.085155 -1.094458 -0.015976
H 1.415382 -2.755716 0.207588
H 5.021038 -0.429287 0.009022
H -1.987380 -2.062547 -0.986731
H -3.206155 1.515006 1.048653
H -4.326045 -2.833766 -1.024335
H -5.539890 0.738306 1.007110
H -6.111809 -1.433825 -0.032385
H -0.820766 3.335316 -0.235017
H 4.939479 -2.742521 0.175850

SCF Energy: -953.279881331
Sum of electronic and zero-point Energies= -953.070195
Sum of electronic and thermal Energies= -953.054489
Sum of electronic and thermal Enthalpies= -953.053545
Sum of electronic and thermal Free Energies= -953.114432

==> ./AOX-I/water/HAT/C5/vEA <==
O -0.164803 -0.815641 0.093005
O -1.398526 2.556175 -0.155239
O 3.785866 1.809326 -0.131723
O 1.193646 2.975992 -0.177449
O 3.987592 -2.899640 0.197274
C 1.722950 0.640570 -0.020699
C 1.159963 -0.615440 0.066291
C -1.042721 0.222473 0.028683
C 0.852339 1.789177 -0.085200
C -0.574189 1.491006 -0.056018
C -2.441484 -0.215571 0.021164
C 3.187667 0.726082 -0.047336
C 1.920967 -1.803992 0.139159
C 3.316083 -1.725029 0.120876
C 3.942213 -0.505741 0.028382
C -2.764231 -1.450700 -0.550610
C -3.449589 0.572238 0.583359
C -4.082159 -1.882515 -0.571789
C -4.764996 0.128457 0.563596
C -5.085155 -1.094458 -0.015976
H 1.415382 -2.755716 0.207588
H 5.021038 -0.429287 0.009022
H -1.987380 -2.062547 -0.986731
H -3.206155 1.515006 1.048653
H -4.326045 -2.833766 -1.024335
H -5.539890 0.738306 1.007110
H -6.111809 -1.433825 -0.032385
H -0.820766 3.335316 -0.235017
H 4.939479 -2.742521 0.175850

SCF Energy: -953.466348472

==> ./AOX-I/water/HAT/C5/TS <==
O 0.705697 0.995178 0.022255
O 1.536292 -2.455351 -0.616775
O -3.498839 -1.089875 -0.924804
O -1.078544 -2.523202 -0.863589
O -3.206530 3.515812 0.144774
C -1.335332 -0.174943 -0.441436
C -0.635507 0.985652 -0.127367
C 1.446903 -0.136257 -0.126749
C -0.601714 -1.412355 -0.595310
C 0.839598 -1.308751 -0.426955
C 2.883761 0.102856 0.042182
C -2.759247 -0.060412 -0.573397
C -1.257338 2.221952 0.065944
C -2.634868 2.302989 -0.052174
C -3.387871 1.172140 -0.359739
C 3.408474 1.358016 -0.283855
C 3.730710 -0.892937 0.537421
C 4.764697 1.605426 -0.130534
C 5.085579 -0.633745 0.694287
C 5.606476 0.610942 0.357732
H -0.663932 3.092067 0.306795
H -4.463751 1.230075 -0.457845
H 2.757414 2.131422 -0.665594
H 3.331494 -1.855965 0.815571
H 5.164477 2.575194 -0.393076
H 5.733674 -1.405699 1.085713
H 6.663173 0.806831 0.478617
H 0.875417 -3.132209 -0.842861
H -4.163091 3.465283 0.025814
H -3.611074 -1.771092 -0.022725
O -3.694981 -2.166258 1.158055
O -3.103124 -1.151552 1.837205
H -3.829503 -0.577369 2.132327

SCF Energy: -1104.84599412

Sum of electronic and zero-point Energies= -1104.611551
Sum of electronic and thermal Energies= -1104.592830
Sum of electronic and thermal Enthalpies= -1104.591886
Sum of electronic and thermal Free Energies= -1104.660302

==> ./AOX-I/water/HAT/C3/vEA <==

O	-0.245742	-0.825469	0.068092
O	-1.327357	2.540421	-0.231592
O	3.764526	1.816566	-0.063873
O	1.356970	2.900148	-0.117042
O	3.922427	-2.914545	0.144610
C	1.714285	0.574505	-0.008112
C	1.117405	-0.691806	0.048966
C	-1.081470	0.216199	0.007331
C	0.902971	1.752919	-0.067764
C	-0.586857	1.552870	-0.095286
C	-2.480720	-0.163377	0.012410
C	3.130251	0.632677	-0.011064
C	1.838543	-1.859235	0.100617
C	3.230903	-1.755577	0.093766
C	3.883084	-0.525514	0.038428
C	-2.834368	-1.450275	-0.426223
C	-3.479755	0.711398	0.466543
C	-4.161957	-1.840974	-0.432117
C	-4.804005	0.302289	0.468788
C	-5.149602	-0.966653	0.014717
H	1.347752	-2.819645	0.145890
H	4.963342	-0.471904	0.033067
H	-2.069918	-2.126912	-0.778909
H	-3.217337	1.688717	0.837025
H	-4.428694	-2.827130	-0.785624
H	-5.568462	0.975099	0.831435
H	-6.185710	-1.276141	0.012872
H	3.088395	2.525803	-0.098080
H	4.871800	-2.741522	0.134776

SCF Energy: -953.464489235

==> ./AOX-I/water/HAT/C3/TS <==

O	-0.021196	-1.176925	0.068007
O	-1.378307	1.959697	-1.001297
O	3.757318	1.715590	-0.542755
O	1.274333	2.543567	-0.886385
O	4.303227	-2.838250	0.672605
C	1.818642	0.331840	-0.262127
C	1.326297	-0.939361	0.050604
C	-0.919020	-0.225450	-0.224369
C	0.908279	1.391897	-0.599701
C	-0.516531	1.047648	-0.590192
C	-2.296083	-0.708145	-0.167031
C	3.221658	0.514370	-0.250726
C	2.139513	-2.005012	0.365627
C	3.514298	-1.782126	0.363124
C	4.063246	-0.535980	0.059081
C	-2.551158	-2.050816	-0.474014
C	-3.348763	0.133027	0.210564
C	-3.847799	-2.536939	-0.426328
C	-4.640288	-0.368963	0.271664
C	-4.893552	-1.697814	-0.053175
H	1.726357	-2.973510	0.603985
H	5.134977	-0.389343	0.064995
H	-1.738202	-2.699935	-0.765899
H	-3.157985	1.160026	0.479919
H	-4.042759	-3.569679	-0.679503
H	-5.450257	0.278659	0.576901
H	-5.904195	-2.080421	-0.011805
H	3.021818	2.334170	-0.739411
H	5.234546	-2.586330	0.641624
H	-1.545009	2.677595	-0.190969
O	-1.596296	3.227024	0.996249
O	-0.975932	2.298824	1.759924
H	-0.056900	2.603485	1.851381

SCF Energy: -1104.86170958
Sum of electronic and zero-point Energies= -1104.626327
Sum of electronic and thermal Energies= -1104.607952
Sum of electronic and thermal Enthalpies= -1104.607008
Sum of electronic and thermal Free Energies= -1104.674145

==> ./AOX-I/water/HAT/C3 <==

O	-0.245742	-0.825469	0.068092
O	-1.327357	2.540421	-0.231592
O	3.764526	1.816566	-0.063873
O	1.356970	2.900148	-0.117042
O	3.922427	-2.914545	0.144610
C	1.714285	0.574505	-0.008112
C	1.117405	-0.691806	0.048966
C	-1.081470	0.216199	0.007331
C	0.902971	1.752919	-0.067764
C	-0.586857	1.552870	-0.095286
C	-2.480720	-0.163377	0.012410
C	3.130251	0.632677	-0.011064
C	1.838543	-1.859235	0.100617
C	3.230903	-1.755577	0.093766
C	3.883084	-0.525514	0.038428
C	-2.834368	-1.450275	-0.426223
C	-3.479755	0.711398	0.466543

C	-4.161957	-1.840974	-0.432117
C	-4.804005	0.302289	0.468788
C	-5.149602	-0.966653	0.014717
H	1.347752	-2.819645	0.145890
H	4.963342	-0.471904	0.033067
H	-2.069918	-2.126912	-0.778909
H	-3.217337	1.688717	0.837025
H	-4.428694	-2.827130	-0.785624
H	-5.568462	0.975099	0.831435
H	-6.185710	-1.276141	0.012872
H	3.088395	2.525803	-0.098080
H	4.871800	-2.741522	0.134776

SCF Energy: -953.297133097
Sum of electronic and zero-point Energies= -953.086318
Sum of electronic and thermal Energies= -953.071006
Sum of electronic and thermal Enthalpies= -953.070062
Sum of electronic and thermal Free Energies= -953.129913

==> ./AOX-I/water/HAT/C7 <==

O	-0.205565	-0.899276	0.089823
O	-1.292436	2.519110	-0.186988
O	3.768255	1.771828	-0.099996
O	1.359627	2.821887	-0.177567
O	3.993807	-2.912972	0.187747
C	1.742256	0.482602	-0.012128
C	1.138540	-0.765121	0.069211
C	-1.024742	0.172039	0.019951
C	0.917454	1.656107	-0.087074
C	-0.504015	1.431282	-0.074115
C	-2.442686	-0.190270	0.017489
C	3.191603	0.559227	-0.023576
C	1.881513	-1.934387	0.137459
C	3.333035	-1.856229	0.125629
C	3.953126	-0.561356	0.042049
C	-2.828278	-1.411786	-0.545864
C	-3.406097	0.651176	0.582035
C	-4.165708	-1.776517	-0.557991
C	-4.740890	0.271455	0.574453
C	-5.123856	-0.936577	0.001551
H	1.399838	-2.898792	0.198220
H	5.031578	-0.497152	0.033417
H	-2.084613	-2.062459	-0.983394
H	-3.113522	1.582455	1.041782
H	-4.460366	-2.715711	-1.005164
H	-5.481909	0.919718	1.021083
H	-6.166171	-1.224606	-0.006664
H	-0.704152	3.288319	-0.269184
H	3.062289	2.453747	-0.148728

SCF Energy: -953.278669841
Sum of electronic and zero-point Energies= -953.069079
Sum of electronic and thermal Energies= -953.053631
Sum of electronic and thermal Enthalpies= -953.052686
Sum of electronic and thermal Free Energies= -953.112844

==> ./AOX-I/water/HAT/C7/vEA <==

O	-0.205565	-0.899276	0.089823
O	-1.292436	2.519110	-0.186988
O	3.768255	1.771828	-0.099996
O	1.359627	2.821887	-0.177567
O	3.993807	-2.912972	0.187747
C	1.742256	0.482602	-0.012128
C	1.138540	-0.765121	0.069211
C	-1.024742	0.172039	0.019951
C	0.917454	1.656107	-0.087074
C	-0.504015	1.431282	-0.074115
C	-2.442686	-0.190270	0.017489
C	3.191603	0.559227	-0.023576
C	1.881513	-1.934387	0.137459
C	3.333035	-1.856229	0.125629
C	3.953126	-0.561356	0.042049
C	-2.828278	-1.411786	-0.545864
C	-3.406097	0.651176	0.582035
C	-4.165708	-1.776517	-0.557991
C	-4.740890	0.271455	0.574453
C	-5.123856	-0.936577	0.001551
H	1.399838	-2.898792	0.198220
H	5.031578	-0.497152	0.033417
H	-2.084613	-2.062459	-0.983394
H	-3.113522	1.582455	1.041782
H	-4.460366	-2.715711	-1.005164
H	-5.481909	0.919718	1.021083
H	-6.166171	-1.224606	-0.006664
H	-0.704152	3.288319	-0.269184
H	3.062289	2.453747	-0.148728

SCF Energy: -953.473418963

==> ./AOX-I/water/HAT/C7/TS <==

O	-0.452045	-0.571374	-0.289513
O	-2.189471	2.529822	0.323607
O	2.921372	2.816472	-0.060126
O	0.353202	3.354447	0.237648
O	4.058992	-1.680766	-0.882880
C	1.181678	1.165631	-0.160249
C	0.834482	-0.166353	-0.347269

C -1.463366 0.301481 -0.054442
C 0.146083 2.132912 0.082085
C -1.202879 1.624324 0.126409
C -2.779706 -0.342404 -0.060090
C 2.565026 1.531499 -0.234204
C 1.787879 -1.147344 -0.605212
C 3.145691 -0.759402 -0.663292
C 3.526332 0.584436 -0.469849
C -2.982677 -1.467622 -0.866424
C -3.822081 0.135626 0.739259
C -4.219110 -2.095346 -0.881977
C -5.053370 -0.505122 0.723531
C -5.256443 -1.616106 -0.087831
H 1.497613 -2.170094 -0.788680
H 4.570474 0.857776 -0.513395
H -2.178356 -1.839369 -1.485048
H -3.666752 0.987386 1.383092
H -4.373190 -2.958671 -1.514336
H -5.853456 -0.137462 1.350977
H -6.218856 -2.109179 -0.099189
H -1.764869 3.400661 0.389897
H 2.108925 3.346490 0.089067
H 4.040859 -2.422490 -0.003816
O 3.884107 -2.973623 1.089783
O 2.723763 -2.417492 1.537483
H 2.993008 -1.672193 2.100751

SCF Energy: -1104.84974567
Sum of electronic and zero-point Energies= -1104.615369
Sum of electronic and thermal Energies= -1104.596869
Sum of electronic and thermal Enthalpies= -1104.595925
Sum of electronic and thermal Free Energies= -1104.663626

==> ./AOX-II/aIP <==
O -0.236125 -0.834011 0.059886
O -1.299137 2.546950 -0.086430
O 3.772653 1.795528 -0.071035
O 1.301834 2.898701 -0.085941
O 3.918915 -2.926882 0.097057
C 1.723198 0.562160 -0.007840
C 1.124412 -0.707115 0.042489
C -1.085832 0.180583 0.025062
C 0.910486 1.722843 -0.039629
C -0.547296 1.490387 -0.026510
C -2.475022 -0.196182 0.012999
C 3.145790 0.624037 -0.024273
C 1.844218 -1.872644 0.078690
C 3.249517 -1.771857 0.061507
C 3.897855 -0.540648 0.008801
C -2.809097 -1.543895 -0.249481
C -3.509037 0.729818 0.267891
C -4.130158 -1.942202 -0.270309
C -4.827667 0.312690 0.256166
C -5.143636 -1.016476 -0.016849
H 1.355378 -2.836611 0.116092
H 4.979961 -0.486744 -0.005948
H -2.026691 -2.262615 -0.453255
H -3.283843 1.760110 0.496542
H -4.377230 -2.974728 -0.486173
H -5.615015 1.027137 0.464083
H -6.180181 -1.333586 -0.031211
H -0.706822 3.329175 -0.109010
H 3.111500 2.517067 -0.090591
H 4.871435 -2.770492 0.078894

SCF Energy: -953.194478668
Sum of electronic and zero-point Energies= -952.973058
Sum of electronic and thermal Energies= -952.957419
Sum of electronic and thermal Enthalpies= -952.956475
Sum of electronic and thermal Free Energies= -953.017424

==> ./AOX-II <==
O -0.215140 -0.861630 0.076038
O -1.349206 2.558481 -0.154834
O 3.741687 1.826454 -0.102977
O 1.287932 2.882527 -0.170510
O 3.983502 -2.903991 0.181953
C 1.711167 0.549988 -0.016706
C 1.128876 -0.724652 0.060208
C -1.061233 0.200964 0.017483
C 0.868306 1.705050 -0.084564
C -0.558917 1.465705 -0.062812
C -2.470837 -0.201728 0.015436
C 3.128047 0.632322 -0.028356
C 1.879972 -1.886788 0.127261
C 3.264539 -1.762068 0.114862
C 3.895675 -0.514081 0.036836
C -2.833418 -1.447538 -0.515882
C -3.469247 0.625549 0.546146
C -4.162345 -1.847210 -0.528330
C -4.796072 0.214726 0.538198
C -5.148721 -1.018075 -0.001807
H 1.403022 -2.856142 0.187683
H 4.977509 -0.443766 0.025957
H -2.073079 -2.099091 -0.929501
H -3.209974 1.581114 0.981788
H -4.427992 -2.809643 -0.951895
H -5.556838 0.861580 0.961301

H	-6.186608	-1.332860	-0.009918
H	-0.746738	3.316778	-0.231298
H	3.049427	2.518646	-0.148161
H	4.926304	-2.704324	0.168848

SCF Energy: -953.399559852
Sum of electronic and zero-point Energies= -953.178723
Sum of electronic and thermal Energies= -953.163003
Sum of electronic and thermal Enthalpies= -953.162058
Sum of electronic and thermal Free Energies= -953.222114

==> ./AOX-II/vIP <==

O	-0.215140	-0.861630	0.076038
O	-1.349206	2.558481	-0.154834
O	3.741687	1.826454	-0.102977
O	1.287932	2.882527	-0.170510
O	3.983502	-2.903991	0.181953
C	1.711167	0.549988	-0.016706
C	1.128876	-0.724652	0.060208
C	-1.061233	0.200964	0.017483
C	0.868306	1.705050	-0.084564
C	-0.558917	1.465705	-0.062812
C	-2.470837	-0.201728	0.015436
C	3.128047	0.632322	-0.028356
C	1.879972	-1.886788	0.127261
C	3.264539	-1.762068	0.114862
C	3.895675	-0.514081	0.036836
C	-2.833418	-1.447538	-0.515882
C	-3.469247	0.625549	0.546146
C	-4.162345	-1.847210	-0.528330
C	-4.796072	0.214726	0.538198
C	-5.148721	-1.018075	-0.001807
H	1.403022	-2.856142	0.187683
H	4.977509	-0.443766	0.025957
H	-2.073079	-2.099091	-0.929501
H	-3.209974	1.581114	0.981788
H	-4.427992	-2.809643	-0.951895
H	-5.556838	0.861580	0.961301
H	-6.186608	-1.332860	-0.009918
H	-0.746738	3.316778	-0.231298
H	3.049427	2.518646	-0.148161
H	4.926304	-2.704324	0.168848

SCF Energy: -953.186754358

==> ./AOX-II/bis/c4c5/HAT/c5 <==

O	5.402793	-1.124837	-0.133269
O	3.925814	2.144686	-0.307993
O	0.704008	-1.889370	-0.461084
O	1.791921	0.662710	-0.356466
O	3.800739	-5.490807	-0.056310
C	3.027339	-1.408336	-0.273580
C	4.329357	-1.937507	-0.169321
C	5.312606	0.221441	-0.186857
C	2.873924	0.015920	-0.309113
C	4.080250	0.806913	-0.281425
C	6.613403	0.883210	-0.081845
C	1.970182	-2.353211	-0.315729
C	4.598477	-3.295466	-0.098274
C	3.528236	-4.174777	-0.130705
C	2.210740	-3.705770	-0.241897
C	7.664255	0.231464	0.580957
C	6.842144	2.148291	-0.640237
C	8.906789	0.837326	0.692594
C	8.092000	2.743395	-0.531843
C	9.125390	2.095052	0.137009
H	5.617272	-3.649855	-0.015436
H	1.383272	-4.406587	-0.280955
H	7.502468	-0.746263	1.018159
H	6.053245	2.658963	-1.175022
H	9.707063	0.325889	1.215899
H	8.258511	3.718076	-0.976965
H	10.098292	2.566462	0.223526
H	2.967936	2.306907	-0.318894
H	2.987849	-6.009816	-0.073895
Cu	-0.036336	-0.000193	0.219629
H	0.057027	-2.600491	-0.356705
O	-5.370671	1.173511	0.037030
O	-4.123530	-2.128733	0.490234
O	-0.739319	1.745608	0.881143
O	-1.885655	-0.767593	0.673802
O	-3.490732	5.417404	-0.216489
C	-3.017618	1.321395	0.391534
C	-4.250021	1.912344	0.132957
C	-5.383580	-0.153892	0.148886
C	-2.929604	-0.092285	0.486650
C	-4.173589	-0.818534	0.377381
C	-6.685374	-0.753984	-0.070350
C	-1.857475	2.186810	0.484562
C	-4.404543	3.279368	-0.061672
C	-3.269844	4.110061	-0.022020
C	-2.012806	3.572408	0.186973
C	-7.644278	-0.046288	-0.818589
C	-7.020143	-2.010376	0.464014
C	-8.897678	-0.589837	-1.035495
C	-8.283630	-2.537927	0.250845
C	-9.221409	-1.836007	-0.501214
H	-5.383392	3.691482	-0.272147
H	-1.136804	4.208790	0.232273
H	-7.393802	0.918525	-1.241360
H	-6.309318	-2.557989	1.065704

H -9.625309 -0.043648 -1.624444
H -8.538827 -3.500574 0.678327
H -10.205605 -2.258283 -0.670720
H -2.663667 5.914593 -0.183684
H -3.186379 -2.378433 0.597138

SCF Energy: -2103.31522693
Sum of electronic and zero-point Energies= -2102.881463
Sum of electronic and thermal Energies= -2102.847114
Sum of electronic and thermal Enthalpies= -2102.846170
Sum of electronic and thermal Free Energies= -2102.949658

==> ./AOX-II/bis/c4c5/HAT/c7 <==

O	-5.474279	1.053461	0.032539
O	-3.852386	-2.075415	-0.674666
O	-0.890295	2.153645	-0.706493
O	-1.808327	-0.463900	-0.709009
O	-4.095507	5.472897	0.495639
C	-3.140918	1.502954	-0.302247
C	-4.450807	1.930584	-0.003800
C	-5.320080	-0.268857	-0.208238
C	-2.914965	0.101093	-0.509213
C	-4.075381	-0.759849	-0.476490
C	-6.571658	-1.019521	-0.091191
C	-2.144439	2.514775	-0.355129
C	-4.781127	3.249976	0.263865
C	-3.767049	4.193697	0.228525
C	-2.449527	3.831143	-0.083997
C	-7.582682	-0.549437	0.759472
C	-6.791000	-2.193145	-0.824525
C	-8.776463	-1.245623	0.882431
C	-7.992359	-2.878917	-0.702724
C	-8.985854	-2.412325	0.152313
H	-5.801945	3.526541	0.491718
H	-1.672621	4.588008	-0.128802
H	-7.427725	0.356828	1.332416
H	-6.033909	-2.560474	-1.504054
H	-9.545718	-0.875582	1.551272
H	-8.153246	-3.780440	-1.283540
H	-9.920754	-2.953750	0.247693
H	-2.891066	-2.168853	-0.781350
H	-3.315279	6.037360	0.444975
Cu	0.046663	0.139711	-0.114325
H	-0.298763	2.916332	-0.673831
O	5.473818	-1.189108	0.096694
O	4.094726	2.047415	0.318039
O	0.807696	-1.765046	0.902508
O	1.917456	0.719308	0.506814
O	3.655638	-5.465659	0.143509
C	3.085897	-1.395519	0.426224
C	4.360299	-1.967967	0.226392
C	5.462433	0.122081	0.128612
C	2.953450	0.013553	0.405127
C	4.191393	0.760651	0.287044
C	6.729831	0.756237	-0.078975
C	1.988513	-2.328563	0.605434
C	4.574635	-3.310123	0.126873
C	3.451381	-4.243302	0.241671
C	2.158238	-3.674806	0.484115
C	7.785296	-0.011901	-0.625337
C	6.965226	2.104487	0.273218
C	9.024911	0.556788	-0.823001
C	8.218156	2.654241	0.083078
C	9.245105	1.889629	-0.468069
H	5.569514	-3.696523	-0.055293
H	1.314593	-4.342309	0.616274
H	7.610236	-1.041374	-0.908730
H	6.186287	2.702371	0.720783
H	9.825211	-0.030281	-1.256560
H	8.399714	3.682890	0.369389
H	10.222617	2.332388	-0.621809
H	0.112220	-2.435512	0.973504
H	3.136324	2.257750	0.408025

SCF Energy: -2103.29313273
Sum of electronic and zero-point Energies= -2102.860021
Sum of electronic and thermal Energies= -2102.825370
Sum of electronic and thermal Enthalpies= -2102.824426
Sum of electronic and thermal Free Energies= -2102.929267

==> ./AOX-II/bis/c4c5/HAT/c3 <==

O	-5.530047	1.067064	-0.078731
O	-3.848538	-2.105183	-0.296219
O	-0.900957	2.128161	-0.580697
O	-1.821173	-0.489334	-0.411637
O	-4.210510	5.526701	-0.093131
C	-3.178578	1.499342	-0.303050
C	-4.508681	1.944092	-0.155534
C	-5.356108	-0.274989	-0.139115
C	-2.935711	0.084467	-0.337308
C	-4.096287	-0.778426	-0.269006
C	-6.612375	-1.016979	-0.008655
C	-2.183022	2.509950	-0.397697
C	-4.864002	3.282265	-0.083859
C	-3.854634	4.228137	-0.164973
C	-2.515968	3.845891	-0.323546
C	-7.689864	-0.436700	0.676458
C	-6.771370	-2.293035	-0.565519

C -8.888496 -1.122412 0.812002
C -7.977213 -2.969162 -0.432823
C -9.037108 -2.390951 0.258461
H -5.900392 3.570916 0.030718
H -1.740304 4.601275 -0.399402
H -7.583688 0.549464 1.111950
H -5.962107 -2.751616 -1.117049
H -9.709266 -0.663678 1.352364
H -8.088543 -3.952300 -0.876822
H -9.975428 -2.924682 0.363434
H -2.881324 -2.187506 -0.354159
H -3.433928 6.094447 -0.159976
Cu 0.049769 0.119073 0.183139
H -0.324738 2.902284 -0.605371
O 5.521930 -1.034826 -0.110465
O 3.862988 2.023944 0.084815
O 0.978367 -2.162906 0.878901
O 1.886535 0.416289 0.977397
O 4.133920 -5.442669 -0.524135
C 3.185339 -1.498956 0.374901
C 4.465867 -1.926826 -0.024253
C 5.439440 0.246673 0.045530
C 2.936201 -0.105657 0.581100
C 4.065384 0.845016 0.236128
C 6.652135 0.978232 -0.016525
C 2.198189 -2.519178 0.477243
C 4.799999 -3.218893 -0.315831
C 3.784882 -4.188413 -0.226123
C 2.497543 -3.839712 0.162731
C 7.824199 0.328480 -0.482786
C 6.726592 2.326929 0.410688
C 9.014449 1.015573 -0.542183
C 7.933181 2.994240 0.360691
C 9.071003 2.347446 -0.119849
H 5.807360 -3.486325 -0.604454
H 1.725398 -4.598192 0.236039
H 7.771733 -0.699683 -0.815595
H 5.853981 2.826986 0.801855
H 9.904928 0.525654 -0.915840
H 7.994021 4.020356 0.700940
H 10.013014 2.882136 -0.162495
H 3.379026 -6.039256 -0.441394
H 0.376903 -2.919999 0.851653

SCF Energy: -2103.33019971
Sum of electronic and zero-point Energies= -2102.896600
Sum of electronic and thermal Energies= -2102.861541
Sum of electronic and thermal Enthalpies= -2102.860597
Sum of electronic and thermal Free Energies= -2102.966875

==> ./AOX-II/bis/c4c5/aIP <==
O 5.489157 -1.094102 -0.037935
O 3.939502 2.106301 -0.241635
O 0.807893 -1.976744 -0.359021
O 1.843218 0.608675 -0.288271
O 3.999944 -5.486999 0.007920
C 3.114682 -1.439744 -0.183473
C 4.431555 -1.935785 -0.077258
C 5.386043 0.236064 -0.102068
C 2.928866 -0.031955 -0.231969
C 4.118534 0.796481 -0.207002
C 6.655520 0.930934 -0.008689
C 2.076128 -2.413430 -0.230759
C 4.734349 -3.278369 -0.010640
C 3.679742 -4.192295 -0.056853
C 2.352457 -3.760293 -0.170248
C 7.759799 0.260951 0.552391
C 6.820582 2.246755 -0.478942
C 8.984023 0.896461 0.654210
C 8.055456 2.867193 -0.384295
C 9.136914 2.200842 0.186103
H 5.760045 -3.610821 0.074048
H 1.544192 -4.482497 -0.213526
H 7.645777 -0.749766 0.922828
H 5.997363 2.772056 -0.940322
H 9.823117 0.376687 1.101794
H 8.175451 3.876007 -0.761870
H 10.098541 2.695552 0.264444
H 2.979639 2.267114 -0.298466
H 3.211846 -6.043805 -0.028560
Cu 0.000001 0.000000 -0.000014
H 0.167593 -2.700125 -0.300694
O -5.489157 1.094101 0.037945
O -3.939498 -2.106302 0.241617
O -0.807890 1.976744 0.358995
O -1.843216 -0.608675 0.288243
O -3.999947 5.486999 -0.007912
C -3.114681 1.439744 0.183464
C -4.431555 1.935784 0.077262
C -5.386042 -0.236065 0.102073
C -2.928864 0.031955 0.231953
C -4.118532 -0.796482 0.206991
C -6.655521 -0.930935 0.008705
C -2.076127 2.413430 0.230744
C -4.734350 3.278369 0.010650
C -3.679744 4.192295 0.056856
C -2.352457 3.760293 0.170238
C -7.759806 -0.260948 -0.552358
C -6.820577 -2.246758 0.478951
C -8.984032 -0.896458 -0.654166
C -8.055452 -2.867195 0.384315
C -9.136917 -2.200842 -0.186065

H	-5.760048	3.610821	-0.074029
H	-1.544192	4.482498	0.213511
H	-7.645789	0.749771	-0.922791
H	-5.997352	-2.772062	0.940318
H	-9.823131	-0.376681	-1.101736
H	-8.175442	-3.876012	0.761886
H	-10.098545	-2.695551	-0.264397
H	-2.979635	-2.267114	0.298438
H	-3.211849	6.043806	0.028562
H	-0.167592	2.700127	0.300667

SCF Energy: -2103.76138976
Sum of electronic and zero-point Energies= -2103.313059
Sum of electronic and thermal Energies= -2103.278827
Sum of electronic and thermal Enthalpies= -2103.277883
Sum of electronic and thermal Free Energies= -2103.380434

==> ./AOX-II/bis/c4c5/aEA <==

O	5.593041	-1.055418	0.081329
O	3.928283	2.024262	-0.743986
O	1.035666	-2.252382	-0.676935
O	1.925988	0.381977	-0.806521
O	4.294287	-5.491152	0.603292
C	3.270969	-1.554821	-0.287855
C	4.584291	-1.950689	0.042166
C	5.420971	0.261122	-0.191548
C	3.027382	-0.162714	-0.546971
C	4.176782	0.719110	-0.503439
C	6.657419	1.035790	-0.059917
C	2.291355	-2.586703	-0.327336
C	4.936554	-3.257701	0.341168
C	3.942221	-4.222753	0.307689
C	2.623721	-3.893422	-0.028920
C	7.662706	0.591791	0.811325
C	6.868259	2.209250	-0.796156
C	8.840487	1.312059	0.951566
C	8.053255	2.920009	-0.656392
C	9.040523	2.478726	0.219009
H	5.958985	-3.508681	0.590664
H	1.861928	-4.666106	-0.064933
H	7.516068	-0.313939	1.387219
H	6.116825	2.558687	-1.491070
H	9.604547	0.960480	1.636240
H	8.206068	3.821631	-1.239384
H	9.962572	3.039319	0.328177
H	2.968217	2.080175	-0.889297
H	3.526135	-6.071779	0.552503
Cu	0.034511	0.120069	-0.171219
H	0.467868	-3.031804	-0.645440
O	-5.625900	1.020040	0.092257
O	-3.786235	-2.069141	0.192453
O	-1.058956	2.291400	0.644650
O	-1.846971	-0.367837	0.379847
O	-4.533965	5.537037	0.267468
C	-3.297042	1.561968	0.333860
C	-4.648545	1.943392	0.199535
C	-5.385974	-0.314088	0.106535
C	-2.983966	0.159818	0.318237
C	-4.102375	-0.756916	0.214542
C	-6.605289	-1.111763	-0.045751
C	-2.353247	2.618283	0.470094
C	-5.071206	3.263600	0.174629
C	-4.111056	4.256126	0.291805
C	-2.755276	3.938641	0.438147
C	-7.716771	-0.557613	-0.697385
C	-6.696622	-2.416509	0.457837
C	-8.881921	-1.295045	-0.852307
C	-7.869253	-3.145068	0.305917
C	-8.963156	-2.591389	-0.351723
H	-6.121197	3.503142	0.070837
H	-2.019093	4.729410	0.542328
H	-7.663514	0.449906	-1.091597
H	-5.860460	-2.857710	0.982929
H	-9.729355	-0.854903	-1.366392
H	-7.927873	-4.150365	0.708491
H	-9.875321	-3.165793	-0.471663
H	-2.815406	-2.100050	0.249084
H	-3.788531	6.140385	0.368035
H	-0.522363	3.090748	0.707623

SCF Energy: -2104.15858063
Sum of electronic and zero-point Energies= -2103.714273
Sum of electronic and thermal Energies= -2103.678562
Sum of electronic and thermal Enthalpies= -2103.677618
Sum of electronic and thermal Free Energies= -2103.786940

==> ./AOX-II/bis/c4c5/RAF/c2p <==

O	-5.703977	-1.054370	0.045980
O	-4.000524	2.026535	0.785700
O	-1.121358	-2.254725	0.623025
O	-1.993518	0.380067	0.737733
O	-4.427622	-5.492139	-0.528233
C	-3.369726	-1.555145	0.314168
C	-4.695861	-1.951023	0.042015
C	-5.518413	0.261930	0.310184
C	-3.111465	-0.161550	0.548766
C	-4.260476	0.720419	0.562372
C	-6.759281	1.036914	0.235738

C	-2.390716	-2.587626	0.319681
C	-5.060665	-3.258425	-0.240566
C	-4.065753	-4.223483	-0.246874
C	-2.734903	-3.894374	0.038279
C	-7.804280	0.591312	-0.586469
C	-6.934893	2.211653	0.979130
C	-8.987171	1.311480	-0.672736
C	-8.125119	2.922291	0.893778
C	-9.152284	2.479570	0.066280
H	-6.092620	-3.509658	-0.446924
H	-1.973100	-4.668042	0.047465
H	-7.684757	-0.315580	-1.166788
H	-6.151201	2.561449	1.637379
H	-9.782584	0.959021	-1.320236
H	-8.250497	3.824937	1.481686
H	-10.078325	3.040219	-0.000375
H	-3.034227	2.084780	0.878988
H	-3.656454	-6.070748	-0.507909
Cu	-0.190381	-0.009891	-0.134793
H	-0.558901	-3.037473	0.579203
O	5.284234	1.047038	-0.104919
O	3.568989	-2.035886	-0.739090
O	0.789354	2.239347	-1.174828
O	1.616478	-0.389812	-1.007827
O	4.078407	5.530031	-0.144845
C	2.989084	1.553508	-0.630235
C	4.304235	1.951952	-0.291504
C	5.094651	-0.288810	-0.245900
C	2.710122	0.161892	-0.758456
C	3.847527	-0.756246	-0.589819
C	6.257138	-1.080776	0.031613
C	2.032576	2.595226	-0.818853
C	4.674114	3.273853	-0.129586
C	3.702343	4.250810	-0.308350
C	2.385879	3.917406	-0.651717
C	7.390223	-0.454791	0.782510
C	6.410198	-2.372806	-0.451414
C	8.536729	-1.338505	1.089373
C	7.573142	-3.089943	-0.197948
C	8.646153	-2.583652	0.570369
H	5.691346	3.533808	0.130816
H	1.647218	4.698914	-0.798091
H	7.825347	0.231964	0.017792
H	5.638735	-2.833006	-1.048239
H	9.307983	-0.910731	1.720387
H	7.653325	-4.090044	-0.608907
H	9.512732	-3.202013	0.763780
H	3.334441	6.124606	-0.299205
H	0.232901	3.023355	-1.261753
H	2.608733	-2.084145	-0.920654
O	6.941131	0.267169	1.901285
H	7.579555	0.960825	2.095762

SCF Energy: -2179.75345972

Sum of electronic and zero-point Energies= -2179.293404

Sum of electronic and thermal Energies= -2179.256246

Sum of electronic and thermal Enthalpies= -2179.255302

Sum of electronic and thermal Free Energies= -2179.366261

==> ./AOX-II/bis/c4c5/RAF/c6 <==

O	-5.536372	-1.172584	0.055779
O	-4.113703	2.068448	0.691099
O	-0.815892	-1.867134	0.131072
O	-1.958834	0.664846	0.396460
O	-3.858755	-5.432285	-0.759210
C	-3.144783	-1.412908	0.064648
C	-4.441730	-1.949570	-0.078887
C	-5.471346	0.151236	0.337377
C	-3.019672	-0.001924	0.306060
C	-4.251670	0.745209	0.460674
C	-6.791786	0.778659	0.430396
C	-2.064697	-2.332723	-0.056463
C	-4.690821	-3.284760	-0.356913
C	-3.604516	-4.134821	-0.491187
C	-2.294442	-3.663680	-0.341291
C	-7.874050	0.219327	-0.264295
C	-7.006642	1.920176	1.214485
C	-9.133703	0.796095	-0.186247
C	-8.272248	2.486504	1.294124
C	-9.338015	1.931481	0.592803
H	-5.705930	-3.645434	-0.457236
H	-1.456177	-4.347160	-0.431297
H	-7.725118	-0.663743	-0.873756
H	-6.191948	2.355812	1.776673
H	-9.958691	0.356521	-0.736041
H	-8.425538	3.364124	1.912526
H	-10.323919	2.379157	0.655449
H	-3.154658	2.230868	0.692321
H	-3.037934	-5.937048	-0.797919
Cu	-0.142296	0.534895	-0.476582
H	-0.168957	-2.570772	-0.005196
O	5.416209	0.482755	0.251184
O	3.134244	-2.167729	-0.616539
O	1.528950	2.748199	-1.132909
O	1.689961	-0.044038	-1.159550
O	5.268336	5.100088	0.474631
C	3.425471	1.489961	-0.544324
C	4.735055	1.579346	-0.005275
C	4.947617	-0.793851	0.039415
C	2.836958	0.183804	-0.737419
C	3.681344	-0.957148	-0.430911
C	5.925687	-1.811585	0.410146

C	2.768516	2.693015	-0.789849
C	5.384204	2.774142	0.351719
C	4.761048	3.955597	0.092105
C	3.523170	3.985461	-0.751316
C	6.931613	-1.504039	1.338932
C	5.889435	-3.095322	-0.153507
C	7.866187	-2.461813	1.703206
C	6.834708	-4.044169	0.210969
C	7.821594	-3.734768	1.141641
H	6.334806	2.733488	0.865329
H	3.924292	4.040627	-1.781627
H	6.974947	-0.517057	1.782511
H	5.137463	-3.347834	-0.888033
H	8.632432	-2.212693	2.428783
H	6.800567	-5.029979	-0.239300
H	8.554884	-4.481349	1.426185
H	4.659568	5.830900	0.277213
H	1.239210	3.681207	-1.180915
H	2.222554	-2.017900	-0.919708
O	2.685505	5.066382	-0.488292
H	2.682648	5.669136	-1.239784

SCF Energy: -2179.78074578
Sum of electronic and zero-point Energies= -2179.319463
Sum of electronic and thermal Energies= -2179.283353
Sum of electronic and thermal Enthalpies= -2179.282409
Sum of electronic and thermal Free Energies= -2179.391790

==> ./AOX-II/bis/c4c5/RAF/c5p <==

O	5.688756	-1.159126	-0.027778
O	4.000127	1.947438	-0.693335
O	1.127796	-2.335262	-0.792634
O	1.998875	0.294474	-0.786986
O	4.403109	-5.618330	0.320381
C	3.365893	-1.651420	-0.391649
C	4.683635	-2.057028	-0.095130
C	5.506989	0.167365	-0.238916
C	3.112137	-0.250722	-0.580047
C	4.257365	0.633561	-0.517039
C	6.739630	0.943048	-0.081250
C	2.387771	-2.681896	-0.467463
C	5.041954	-3.374942	0.142937
C	4.048082	-4.339140	0.080410
C	2.725412	-3.998880	-0.228455
C	7.750691	0.470607	0.768038
C	6.939411	2.147319	-0.769024
C	8.923462	1.193399	0.934882
C	8.119499	2.860629	-0.603061
C	9.112405	2.391020	0.250953
H	6.067351	-3.634580	0.370433
H	1.965232	-4.771723	-0.289464
H	7.612061	-0.459171	1.306440
H	6.183382	2.519285	-1.447167
H	9.692015	0.819856	1.602697
H	8.263963	3.786737	-1.148565
H	10.030373	2.953784	0.381305
H	3.036867	2.007340	-0.813345
H	3.632446	-6.194834	0.260510
Cu	0.199035	-0.077437	0.085921
H	0.564700	-3.118835	-0.795494
O	-5.182545	1.433259	-0.052427
O	-3.656383	-1.717472	0.747869
O	-0.630466	2.451356	0.962382
O	-1.612105	-0.144827	0.973607
O	-3.745526	5.848131	-0.263001
C	-2.868131	1.845785	0.453833
C	-4.154578	2.297183	0.085977
C	-5.057398	0.104110	0.166973
C	-2.676306	0.442485	0.669046
C	-3.851410	-0.408546	0.529161
C	-6.312667	-0.619047	-0.060566
C	-1.859416	2.843257	0.585762
C	-4.457525	3.625903	-0.156543
C	-3.438618	4.557950	-0.025883
C	-2.144298	4.172033	0.343769
C	-7.400004	0.044526	-0.671098
C	-6.475282	-1.949981	0.271968
C	-8.619390	-0.577110	-0.943074
C	-7.763093	-2.599275	0.114203
C	-8.797169	-1.898904	-0.631195
H	-5.459966	3.920800	-0.436742
H	-1.364629	4.919296	0.451689
H	-7.285950	1.085083	-0.946386
H	-5.692273	-2.530264	0.739052
H	-9.405694	-0.019099	-1.433628
H	-9.722392	-2.423856	-0.840461
H	-2.968205	6.407763	-0.149424
H	-0.044157	3.216604	1.011271
H	-2.710361	-1.811363	0.960740
H	-8.133831	-2.231002	1.136444
O	-7.701865	-3.980811	0.081672
H	-8.585427	-4.346513	0.205280

SCF Energy: -2179.75056574
Sum of electronic and zero-point Energies= -2179.291590
Sum of electronic and thermal Energies= -2179.254597
Sum of electronic and thermal Enthalpies= -2179.253652
Sum of electronic and thermal Free Energies= -2179.364611

==> ./AOX-II/bis/c4c5/RAF/c5 <==

O	5.506360	0.890829	0.024337
O	4.054015	-2.342648	0.627497
O	0.808265	1.663217	0.337038
O	1.914441	-0.895865	0.463628
O	3.872143	5.203332	-0.574729
C	3.122178	1.170892	0.145169
C	4.421395	1.690140	-0.037336
C	5.430315	-0.439377	0.270015
C	2.982515	-0.244213	0.347298
C	4.206034	-1.015939	0.429355
C	6.742086	-1.091032	0.286336
C	2.054555	2.112646	0.108242
C	4.682747	3.029647	-0.281329
C	3.607367	3.902140	-0.336328
C	2.297314	3.449207	-0.138886
C	7.807895	-0.519547	-0.424009
C	6.966008	-2.269459	1.011353
C	9.058925	-1.119775	-0.419244
C	8.223200	-2.859476	1.017714
C	9.271838	-2.291816	0.300973
H	5.698826	3.376069	-0.416172
H	1.468831	4.150013	-0.164377
H	7.652852	0.391809	-0.988496
H	6.165309	-2.716636	1.584245
H	9.870471	-0.669777	-0.980479
H	8.383381	-3.766339	1.590503
H	10.250919	-2.758427	0.305581
H	3.093016	-2.489493	0.662201
H	3.057053	5.718891	-0.575886
Cu	0.117647	-0.876751	-0.438125
H	0.173405	2.387473	0.271276
O	-5.272431	0.222010	0.206163
O	-2.130743	1.615382	-0.858990
O	-2.711799	-3.563782	-0.898668
O	-1.728134	-1.027482	-1.236215
O	-7.013071	-4.006042	0.913235
C	-3.915028	-1.584081	-0.493820
C	-5.122581	-1.076626	0.047737
C	-4.314260	1.158409	-0.100670
C	-2.834121	-0.669609	-0.793540
C	-3.108193	0.741195	-0.578785
C	-4.759835	2.517909	0.191654
C	-3.813142	-2.970265	-0.598686
C	-6.192940	-1.855507	0.520908
C	-6.111285	-3.208115	0.400920
C	-5.030226	-3.827264	-0.431544
C	-5.753129	2.727269	1.160860
C	-4.222540	3.624795	-0.481396
C	-6.183611	4.011667	1.458006
C	-4.666674	4.905703	-0.183329
C	-5.642169	5.105404	0.788387
H	-7.018647	-1.377192	1.029309
H	-5.459696	-3.813877	-1.451507
H	-6.179936	1.883962	1.689531
H	-3.473963	3.486086	-1.248871
H	-6.944621	4.158362	2.216329
H	-4.249153	5.751735	-0.717777
H	-5.981613	6.108828	1.020917
H	-6.740513	-4.933827	0.814413
H	-2.827050	-4.532706	-0.835374
H	-1.349837	1.116473	-1.145349
O	-4.706664	-5.124844	-0.045561
H	-4.967678	-5.748052	-0.732415

SCF Energy: -2179.78195713

Sum of electronic and zero-point Energies= -2179.319094

Sum of electronic and thermal Energies= -2179.282720

Sum of electronic and thermal Enthalpies= -2179.281776

Sum of electronic and thermal Free Energies= -2179.391227

==> ./AOX-II/bis/c4c5/RAF/c3p <==

O	-5.757197	-1.085469	0.037698
O	-4.060965	1.989216	0.821185
O	-1.195999	-2.299531	0.733891
O	-2.061306	0.332355	0.836438
O	-4.482417	-5.528065	-0.502276
C	-3.433303	-1.595458	0.369848
C	-4.752871	-1.986292	0.061905
C	-5.572858	0.230833	0.304210
C	-3.175950	-0.203283	0.613266
C	-4.320855	0.683986	0.592298
C	-6.806765	1.012066	0.189478
C	-2.457632	-2.630595	0.399262
C	-5.114364	-3.292429	-0.230289
C	-4.122639	-4.260785	-0.211141
C	-2.798302	-3.936376	0.107619
C	-7.825782	0.573214	-0.668285
C	-7.000930	2.187037	0.927755
C	-9.001138	1.300062	-0.793528
C	-8.183639	2.904421	0.803144
C	-9.184769	2.468214	-0.059008
H	-6.141180	-3.540328	-0.464453
H	-2.039094	-4.712048	0.134674
H	-7.691648	-0.333536	-1.245625
H	-6.238082	2.532194	1.612444
H	-9.776090	0.952670	-1.468023
H	-8.323805	3.807178	1.387535
H	-10.104978	3.033981	-0.156310
H	-3.097361	2.042232	0.941745
H	-3.714780	-6.110343	-0.463213

Cu	-0.255517	-0.042154	-0.032666
H	-0.631855	-3.081408	0.697302
O	5.196094	1.181882	-0.171596
O	3.611845	-2.000243	-0.700586
O	0.614667	2.183150	-1.059267
O	1.558386	-0.419308	-0.896246
O	3.776577	5.607596	-0.051115
C	2.862014	1.584915	-0.588492
C	4.164619	2.044910	-0.295925
C	5.046161	-0.153892	-0.320243
C	2.646076	0.172371	-0.706274
C	3.821929	-0.679339	-0.584043
C	6.299513	-0.885094	-0.103815
C	1.855374	2.582682	-0.728578
C	4.481035	3.380689	-0.116751
C	3.459429	4.311327	-0.235425
C	2.151589	3.917888	-0.544193
C	7.296896	-0.321215	0.665122
C	6.519622	-2.171345	-0.638656
C	8.577635	-0.981508	0.818589
C	7.697476	-2.889590	-0.419527
C	8.693732	-2.355991	0.354456
H	5.495039	3.681330	0.110733
H	1.370027	4.663314	-0.651744
H	7.189943	0.658708	1.113497
H	5.753402	-2.624614	-1.252758
H	8.984814	-0.537815	-0.159070
H	7.803537	-3.879776	-0.842180
H	9.617375	-2.886844	0.555538
H	2.994324	6.163484	-0.147787
H	0.027344	2.946989	-1.119964
H	2.654689	-2.104796	-0.844315
O	9.315190	-0.547117	1.904407
H	10.231537	-0.832355	1.812334

SCF Energy: -2179.75034943
Sum of electronic and zero-point Energies= -2179.291832
Sum of electronic and thermal Energies= -2179.254547
Sum of electronic and thermal Enthalpies= -2179.253603
Sum of electronic and thermal Free Energies= -2179.365491

==> ./AOX-II/bis/c4c5/RAF/c4p <==

O	-5.847932	-1.114337	0.311064
O	-4.147381	2.052307	0.351256
O	-1.389170	-2.084915	-1.103333
O	-2.215152	0.500869	-0.386138
O	-4.711029	-5.489018	-0.764966
C	-3.573754	-1.499385	-0.369368
C	-4.880333	-1.959770	-0.098401
C	-5.645093	0.214912	0.475158
C	-3.300231	-0.098270	-0.194806
C	-4.408938	0.732991	0.234308
C	-6.859872	0.929450	0.875492
C	-2.641304	-2.481655	-0.808651
C	-5.267907	-3.284956	-0.223630
C	-4.320139	-4.203676	-0.644685
C	-3.009931	-3.805585	-0.937129
C	-8.113796	0.408451	0.524452
C	-6.802834	2.119808	1.612762
C	-9.277710	1.070222	0.888709
C	-7.972693	2.771544	1.980878
C	-9.212161	2.254285	1.617639
H	-6.283837	-3.583169	-0.001222
H	-2.281084	-4.535760	-1.274658
H	-8.175209	-0.511252	-0.044617
H	-5.847807	2.528374	1.913640
H	-10.239361	0.659579	0.601018
H	-7.913478	3.687233	2.558915
H	-10.122777	2.769777	1.903039
H	-3.216530	2.156745	0.090167
H	-3.981165	-6.040899	-1.069433
Cu	-0.253425	0.162797	-0.707363
H	-0.862561	-2.840323	-1.391972
O	5.293893	1.229892	0.135872
O	3.539143	-1.861372	-0.307521
O	0.955302	2.385015	-1.481898
O	1.712355	-0.234775	-1.013561
O	4.202692	5.705093	-0.421034
C	3.074101	1.719990	-0.661706
C	4.359100	2.127570	-0.226708
C	5.090098	-0.115946	0.121804
C	2.772926	0.330755	-0.680447
C	3.854440	-0.589314	-0.266986
C	6.237958	-0.872294	0.521034
C	2.165671	2.754336	-1.040953
C	4.739607	3.452839	-0.144215
C	3.814359	4.423804	-0.509725
C	2.532192	4.080242	-0.958769
C	7.431264	-0.168146	0.871661
C	6.214343	-2.297217	0.605608
C	8.546975	-0.838796	1.243655
C	7.324978	-2.977172	0.979364
C	8.620917	-2.302891	1.167564
H	5.730979	3.721844	0.194780
H	1.830862	4.855490	-1.249396
H	7.432030	0.911656	0.868241
H	5.303896	-2.839599	0.411717
H	9.445873	-0.308261	1.535819
H	7.306444	-4.057335	1.073558
H	9.070860	-2.426541	0.147154
H	3.491095	6.296001	-0.695939
H	0.423234	3.165257	-1.682849

H	2.610614	-1.902236	-0.619712
O	9.477402	-2.846483	2.136231
H	9.712861	-3.742618	1.874049

SCF Energy: -2179.75830095
Sum of electronic and zero-point Energies= -2179.298326
Sum of electronic and thermal Energies= -2179.261078
Sum of electronic and thermal Enthalpies= -2179.260134
Sum of electronic and thermal Free Energies= -2179.373255

==> ./AOX-II/bis/c4c5/RAF/c8 <==

O	5.685589	0.853658	-0.263590
O	3.798229	-1.957405	0.952585
O	1.521616	2.661394	1.203374
O	2.050652	-0.060178	1.236091
O	4.894878	5.410591	-0.757348
C	3.543530	1.666964	0.458761
C	4.821599	1.876811	-0.099108
C	5.395165	-0.420634	0.094797
C	3.165467	0.324598	0.801532
C	4.170478	-0.702219	0.620525
C	6.485447	-1.354588	-0.196025
C	2.726705	2.820653	0.623350
C	5.281575	3.118413	-0.509514
C	4.438909	4.207506	-0.352027
C	3.166808	4.064091	0.216031
C	7.375703	-1.072559	-1.242087
C	6.671891	-2.520482	0.558324
C	8.416225	-1.943071	-1.533586
C	7.721095	-3.382310	0.265907
C	8.593006	-3.100930	-0.781240
H	6.270588	3.226464	-0.934661
H	2.530502	4.933145	0.348567
H	7.245392	-0.174345	-1.833534
H	6.011187	-2.745873	1.384542
H	9.091051	-1.716006	-2.351615
H	7.859592	-4.276177	0.864175
H	9.408575	-3.778849	-1.008431
H	2.874531	-1.881474	1.248063
H	4.236488	6.094569	-0.589054
Cu	0.190039	0.493021	0.637987
H	1.060247	3.507813	1.249292
O	-5.314933	-0.866810	-0.295494
O	-3.721842	2.342332	-0.196540
O	-0.732591	-1.839634	-0.299548
O	-1.657919	0.768333	-0.157501
O	-3.925268	-5.294336	-0.049366
C	-2.991441	-1.243880	-0.268997
C	-4.294906	-1.678333	-0.294417
C	-5.196420	0.496109	-0.275025
C	-2.754366	0.182637	-0.223262
C	-3.939155	1.024123	-0.247456
C	-6.488453	1.174970	-0.243723
C	-1.961947	-2.261183	-0.256598
C	-4.698852	-3.115406	-0.408517
C	-3.551109	-4.054149	-0.177529
C	-2.250898	-3.627278	-0.187761
C	-7.622178	0.485936	0.213940
C	-6.625199	2.504055	-0.668707
C	-8.856707	1.116550	0.254077
C	-7.866983	3.123850	-0.631194
C	-8.984326	2.437043	-0.167599
H	-1.436115	-4.339650	-0.122225
H	-7.534441	-0.540827	0.547677
H	-5.769876	3.049002	-1.043158
H	-9.722340	0.574083	0.617676
H	-7.960093	4.149480	-0.970487
H	-9.951087	2.927440	-0.136489
H	-3.170319	-5.895492	0.046476
H	-0.094403	-2.570345	-0.261051
H	-2.757510	2.457306	-0.130587
H	-4.935404	-3.247320	-1.479435
O	-5.813474	-3.385189	0.386881
H	-6.391169	-3.996319	-0.080961

SCF Energy: -2179.78415153
Sum of electronic and zero-point Energies= -2179.322140
Sum of electronic and thermal Energies= -2179.284966
Sum of electronic and thermal Enthalpies= -2179.284022
Sum of electronic and thermal Free Energies= -2179.397657

==> ./AOX-II/bis/c4c5/RAF/c2 <==

O	5.496012	-1.184839	-0.236882
O	4.029697	2.083948	0.078516
O	0.778208	-1.900994	-0.477979
O	1.891250	0.619952	-0.059840
O	3.873535	-5.522403	-0.667238
C	3.109680	-1.444852	-0.315364
C	4.413332	-1.982937	-0.333634
C	5.411538	0.161724	-0.116697
C	2.964668	-0.024795	-0.155911
C	4.183996	0.752352	-0.081559
C	6.721688	0.804085	0.012963
C	2.042970	-2.375873	-0.445998
C	4.679591	-3.338511	-0.451210
C	3.604357	-4.205920	-0.556929
C	2.287017	-3.727956	-0.555245
C	7.791820	0.082900	0.562093

C 6.936923 2.123651 -0.406380
C 9.039623 0.673385 0.701333
C 8.191309 2.704477 -0.271964
C 9.244235 1.986147 0.285730
H 5.699454 -3.699493 -0.458490
H 1.457398 -4.421179 -0.648694
H 7.641828 -0.938463 0.890496
H 6.132305 2.690837 -0.854275
H 9.854666 0.106457 1.137636
H 8.345651 3.723635 -0.609041
H 10.220659 2.445809 0.393741
H 3.069922 2.230920 0.129672
H 3.057085 -6.031918 -0.731882
Cu 0.019669 0.037282 0.535596
H 0.149883 -2.629266 -0.558419
O -5.251054 1.063004 -0.396030
O -3.861026 -1.508418 1.555148
O -0.897300 2.560955 0.908339
O -1.776082 -0.003014 1.423569
O -3.758879 5.226524 -1.800696
C -3.028121 1.750195 0.311524
C -4.269511 1.998459 -0.347123
C -5.385799 0.084639 0.621403
C -2.809350 0.492912 0.921808
C -4.000274 -0.378736 1.027561
C -6.220034 -1.044368 0.048603
C -2.036154 2.780645 0.251162
C -4.500979 3.156160 -1.040440
C -3.485610 4.124592 -1.101412
C -2.261512 3.944625 -0.455677
C -6.114884 -1.378864 -1.302062
C -7.052216 -1.785844 0.886030
C -6.856419 -2.436735 -1.813253
C -7.794250 -2.841075 0.367604
C -7.698080 -3.167888 -0.980711
H -5.442352 3.315999 -1.548871
H -1.499511 4.715728 -0.494415
H -5.461750 -0.811824 -1.954281
H -7.127614 -1.541997 1.939335
H -6.776355 -2.687925 -2.865022
H -8.447086 -3.408089 1.021769
H -8.277440 -3.992078 -1.382321
H -3.004257 5.829834 -1.806564
H -0.292513 3.306455 0.793404
H -2.907385 -1.624080 1.800869
O -5.873921 0.643111 1.794600
H -6.823827 0.797292 1.703519

SCF Energy: -2179.76636117
Sum of electronic and zero-point Energies= -2179.305174
Sum of electronic and thermal Energies= -2179.268708
Sum of electronic and thermal Enthalpies= -2179.267764
Sum of electronic and thermal Free Energies= -2179.377104

==> ./AOX-II/bis/c4c5/RAF/c8a <==
O -5.117605 1.069268 -0.204295
O -4.229596 -2.414825 -0.252946
O -0.353159 0.981617 0.015620
O -1.884227 -1.338591 0.003538
O -2.788877 5.080304 0.133574
C -2.724914 0.923492 -0.056836
C -3.917575 1.675103 -0.096997
C -5.259583 -0.275253 -0.279496
C -2.824380 -0.508308 -0.086151
C -4.151683 -1.067438 -0.226133
C -6.659518 -0.696727 -0.368459
C -1.514200 1.665440 0.026957
C -3.949864 3.059420 -0.033197
C -2.743988 3.734525 0.070791
C -1.525595 3.042812 0.099048
C -7.661953 0.115216 0.181400
C -7.025491 -1.891065 -1.003408
C -8.993909 -0.266870 0.109331
C -8.361742 -2.261728 -1.079413
C -9.349076 -1.456174 -0.520871
H -4.892128 3.590002 -0.066181
H -0.590342 3.590112 0.162398
H -7.395398 1.041545 0.675842
H -6.271202 -2.522157 -1.453577
H -9.757038 0.366696 0.547890
H -8.631162 -3.183994 -1.582376
H -10.390886 -1.752258 -0.578279
H -3.316365 -2.731325 -0.147964
H -1.899012 5.446439 0.195199
Cu -0.059019 -1.208909 0.880443
H 0.395968 1.575046 0.162778
O 4.860125 -0.045270 -0.494425
O 2.274990 1.382590 1.569677
O 2.144922 -3.672751 0.636028
O 1.718891 -1.194152 1.795915
O 6.213033 -4.209247 -1.825970
C 3.625203 -1.843221 0.514509
C 4.984439 -1.317089 0.138984
C 4.102199 0.895583 0.122801
C 2.763845 -0.894646 1.157359
C 3.098519 0.496792 0.982200
C 4.426343 2.253016 -0.281947
C 3.302667 -3.157825 0.222289
C 5.800427 -2.171152 -0.768051
C 5.414311 -3.440754 -1.068527
C 4.185282 -3.923415 -0.565460
C 5.139729 2.455032 -1.478842

C	4.062624	3.372868	0.487808
C	5.455870	3.735122	-1.900519
C	4.397542	4.649160	0.063402
C	5.087072	4.836782	-1.131390
H	6.754307	-1.771573	-1.092911
H	3.892345	-4.942751	-0.805444
H	5.428771	1.604685	-2.082732
H	3.542599	3.245269	1.425745
H	5.993130	3.876143	-2.831466
H	4.122318	5.502869	0.672235
H	5.340344	5.838463	-1.460741
H	5.826732	-5.080894	-1.974229
H	2.021485	-4.569968	0.300467
H	1.631775	0.865032	2.082998
O	5.686101	-1.185966	1.345241
H	6.612955	-0.985020	1.154745

SCF Energy: -2179.71130760
Sum of electronic and zero-point Energies= -2179.250665
Sum of electronic and thermal Energies= -2179.214159
Sum of electronic and thermal Enthalpies= -2179.213215
Sum of electronic and thermal Free Energies= -2179.321216

==> ./AOX-II/bis/c4c5/RAF/c7 <==

O	5.550930	-1.006316	-0.241225
O	3.556157	1.986306	-0.301553
O	1.017978	-2.508987	-0.190951
O	1.698858	0.189647	-0.162760
O	4.669619	-5.570433	-0.059097
C	3.241212	-1.663944	-0.185307
C	4.616535	-1.977501	-0.188220
C	5.243318	0.311872	-0.298301
C	2.862944	-0.279765	-0.209231
C	3.934543	0.690668	-0.285810
C	6.428369	1.172273	-0.324480
C	2.342143	-2.765634	-0.153776
C	5.104810	-3.274207	-0.144587
C	4.187711	-4.311897	-0.099657
C	2.809564	-4.061913	-0.103872
C	7.629787	0.712279	0.234201
C	6.395810	2.446030	-0.907811
C	8.763288	1.512532	0.220700
C	7.536973	3.237373	-0.924669
C	8.721676	2.777741	-0.358254
H	6.170294	-3.461884	-0.150050
H	2.108277	-4.889831	-0.080044
H	7.671936	-0.270248	0.688373
H	5.486661	2.813421	-1.363743
H	9.682242	1.146112	0.664916
H	7.499043	4.217391	-1.387454
H	9.609198	3.401089	-0.369743
H	2.585557	1.971656	-0.238045
H	3.945136	-6.207144	-0.047775
Cu	-0.062185	-0.517719	0.562902
H	0.517838	-3.333999	-0.166150
O	-5.193329	0.880148	-0.295248
O	-3.867262	-2.181354	0.940406
O	-0.927629	1.914300	1.660939
O	-1.858471	-0.634994	1.451167
O	-3.068175	4.625776	-1.364585
C	-3.001738	1.327291	0.653973
C	-4.191624	1.750456	-0.006098
C	-5.148419	-0.410980	0.028040
C	-2.865945	-0.066100	0.952836
C	-3.998761	-0.900160	0.646762
C	-6.327226	-1.157758	-0.363550
C	-2.007836	2.329138	0.976994
C	-4.410146	3.028929	-0.388414
C	-3.410835	4.103215	-0.105646
C	-2.195876	3.617038	0.613772
C	-7.162816	-0.634605	-1.368165
C	-6.671257	-2.375813	0.249187
C	-8.297222	-1.322819	-1.759873
C	-7.818889	-3.047095	-0.140745
C	-8.629535	-2.529693	-1.147203
H	-5.321522	3.307305	-0.903677
H	-1.449588	4.367664	0.858070
H	-6.907640	0.300331	-1.850482
H	-6.061651	-2.782421	1.042686
H	-8.925596	-0.918976	-2.545167
H	-8.083275	-3.978266	0.346775
H	-9.521624	-3.064812	-1.453126
H	-2.247356	5.130271	-1.296046
H	-0.344512	2.663286	1.836690
H	-2.975835	-2.283675	1.328505
O	-4.108032	5.039773	0.678249
H	-3.482697	5.593931	1.161824

SCF Energy: -2179.71863786
Sum of electronic and zero-point Energies= -2179.258250
Sum of electronic and thermal Energies= -2179.221150
Sum of electronic and thermal Enthalpies= -2179.220206
Sum of electronic and thermal Free Energies= -2179.332540

==> ./AOX-II/bis/c4c5/RAF/c4a <==

O	-5.022293	1.238185	-0.209591
O	-4.251054	-2.274069	-0.204511
O	-0.259223	0.998677	-0.152313

O	-1.861701	-1.271071	-0.077479
O	-2.555283	5.176097	-0.014143
C	-2.631204	1.015818	-0.145192
C	-3.799773	1.805169	-0.155369
C	-5.212637	-0.102973	-0.245504
C	-2.777497	-0.413580	-0.143740
C	-4.129601	-0.929494	-0.215333
C	-6.629574	-0.474498	-0.269351
C	-1.394832	1.719652	-0.113090
C	-3.786328	3.190864	-0.112164
C	-2.556725	3.828161	-0.057693
C	-1.361283	3.098275	-0.057989
C	-7.582495	0.405321	0.264806
C	-7.062905	-1.685785	-0.825558
C	-8.929875	0.074296	0.254711
C	-8.414274	-2.005919	-0.839231
C	-9.351362	-1.132303	-0.296821
H	-4.712034	3.750779	-0.121994
H	-0.407337	3.615576	-0.033576
H	-7.264598	1.346124	0.697477
H	-6.349412	-2.370556	-1.263067
H	-9.653150	0.761635	0.679645
H	-8.735315	-2.942876	-1.281015
H	-10.405185	-1.388656	-0.306070
H	-3.344095	-2.616106	-0.128585
H	-1.651077	5.510416	0.001692
Cu	-0.022796	-1.216891	0.811666
H	0.513255	1.567429	-0.028170
O	4.935135	-0.179494	-0.603779
O	2.342810	1.412982	1.338527
O	2.980592	-3.787715	1.806939
O	1.811100	-1.237029	1.699328
O	4.350767	-4.267701	-2.686079
C	3.949747	-1.928622	0.738604
C	4.530255	-1.458919	-0.559720
C	4.252635	0.797936	0.073190
C	2.840082	-0.910007	1.102552
C	3.167722	0.461972	0.836428
C	4.769053	2.130195	-0.208022
C	3.450229	-3.357103	0.668424
C	4.660840	-2.232266	-1.647563
C	4.181499	-3.578510	-1.592990
C	3.561488	-4.120301	-0.458353
C	5.479451	2.365905	-1.396011
C	4.593287	3.183705	0.703034
C	5.984298	3.627555	-1.670582
C	5.113031	4.438819	0.424833
C	5.803338	4.667054	-0.762332
H	5.083406	-1.850824	-2.567135
H	3.228628	-5.151896	-0.462637
H	5.623039	1.562813	-2.107835
H	4.070063	3.016627	1.634450
H	6.521245	3.800180	-2.596497
H	4.981542	5.241864	1.141268
H	6.202255	5.652187	-0.977939
H	3.998067	-5.168188	-2.611527
H	2.720287	-4.721191	1.761288
H	1.710327	0.978656	1.928246
O	4.981134	-1.834789	1.698042
H	4.615744	-1.979259	2.580304

SCF Energy: -2179.75051259
Sum of electronic and zero-point Energies= -2179.288460
Sum of electronic and thermal Energies= -2179.251670
Sum of electronic and thermal Enthalpies= -2179.250725
Sum of electronic and thermal Free Energies= -2179.361131

==> ./AOX-II/bis/c4c5/RAF/c6p <==

O	5.406408	-0.986019	0.557468
O	3.878155	2.071195	-0.562972
O	1.642806	-2.336243	-2.048000
O	2.226874	0.330140	-1.553433
O	4.491165	-5.520100	0.081672
C	3.469931	-1.569075	-0.740033
C	4.590516	-1.927122	0.038110
C	5.218287	0.342136	0.365367
C	3.201502	-0.172435	-0.937043
C	4.149730	0.762929	-0.368219
C	6.220684	1.162452	1.049746
C	2.694924	-2.641408	-1.266405
C	4.937848	-3.238782	0.320758
C	4.142025	-4.247596	-0.199008
C	3.025106	-3.954102	-0.991289
C	6.862695	0.658986	2.190668
C	6.566037	2.439196	0.586430
C	7.812835	1.419462	2.857024
C	7.524419	3.190655	1.254089
C	8.147319	2.688177	2.392130
H	5.808285	-3.460461	0.923924
H	2.424807	-4.758701	-1.404173
H	6.608409	-0.326418	2.561751
H	6.101681	2.839748	-0.304323
H	8.293159	1.019333	3.743189
H	7.787780	4.173277	0.878275
H	8.891977	3.280674	2.912519
H	3.043978	2.089104	-1.063039
H	3.879583	-6.138677	-0.334106
Cu	0.261323	0.021900	-1.276044
H	1.209977	-3.145359	-2.346474
O	-5.175999	1.223170	0.376976
O	-3.535691	-1.924698	-0.088679
O	-0.898421	2.265262	-1.480324

O -1.694963 -0.354377 -0.935996
O -4.071973 5.656920 -0.426622
C -2.994063 1.646803 -0.561389
C -4.248217 2.091271 -0.076124
C -4.991084 -0.119817 0.370710
C -2.724790 0.245222 -0.570654
C -3.806272 -0.628704 -0.091393
C -6.068913 -0.847075 0.986966
C -2.092032 2.657359 -1.010069
C -4.613205 3.423069 -0.029386
C -3.699554 4.368017 -0.478588
C -2.442049 3.989851 -0.966461
C -6.938886 -0.186954 1.840591
C -6.223388 -2.325133 0.803569
C -7.963587 -0.873622 2.482924
C -7.403373 -2.936147 1.439236
C -8.196445 -2.252127 2.300744
H -5.583712 3.714703 0.348917
H -1.743369 4.743173 -1.315412
H -6.825831 0.873681 2.015408
H -8.616819 -0.317013 3.145463
H -7.565647 -3.984633 1.218718
H -9.023765 -2.734671 2.803533
H -3.370285 6.228436 -0.761475
H -0.369451 3.032297 -1.733060
H -2.624246 -2.023986 -0.428791
H -5.424975 -2.723045 1.482094
O -6.057725 -2.818136 -0.495778
H -5.113007 -2.866353 -0.690170

SCF Energy: -2179.75619944
Sum of electronic and zero-point Energies= -2179.295178
Sum of electronic and thermal Energies= -2179.258517
Sum of electronic and thermal Enthalpies= -2179.257572
Sum of electronic and thermal Free Energies= -2179.367118

==> ./AOX-II/bis/c4c5/RAF/c3 <==
O -5.479301 -1.223006 0.126652
O -4.315337 2.164458 0.452638
O -0.745010 -1.556550 0.616101
O -2.061464 0.886245 0.534533
O -3.482006 -5.418962 -0.004425
C -3.087961 -1.290229 0.352557
C -4.332672 -1.931421 0.182285
C -5.517608 0.127882 0.228549
C -3.070131 0.144120 0.431768
C -4.352491 0.816728 0.382563
C -6.873991 0.668360 0.109779
C -1.945272 -2.136218 0.419965
C -4.474513 -3.305064 0.062964
C -3.329816 -4.084447 0.117662
C -2.067047 -3.506071 0.299631
C -7.842374 -0.045990 -0.610561
C -7.234627 1.881499 0.711335
C -9.132510 0.449157 -0.736446
C -8.530421 2.365953 0.587956
C -9.481791 1.656586 -0.138215
H -5.452888 -3.747732 -0.069034
H -1.183195 -4.133315 0.354662
H -7.580491 -0.985719 -1.081549
H -6.510224 2.438657 1.289768
H -9.867438 -0.110367 -1.304778
H -8.797264 3.301678 1.066899
H -10.491328 2.040915 -0.235682
H -3.372231 2.393054 0.517524
H -2.627626 -5.863743 0.036729
Cu -0.207196 0.714214 -0.268123
H -0.044006 -2.220430 0.611166
O 5.360082 0.783836 0.227079
O 4.008977 -1.020201 -2.076527
O 1.295308 2.999340 -1.000378
O 1.591936 0.266338 -1.138088
O 5.026298 5.380570 0.765772
C 3.257709 1.820140 -0.428291
C 4.567834 1.916218 0.073898
C 5.043276 -0.417615 -0.099073
C 2.739947 0.537856 -0.779767
C 3.691867 -0.678380 -0.756552
C 6.014369 -1.421791 0.163621
C 2.536188 3.041963 -0.516766
C 5.170003 3.073484 0.473309
C 4.420157 4.257470 0.369956
C 3.118890 4.240022 -0.119236
C 7.200052 -1.068159 0.852662
C 5.830328 -2.756969 -0.264657
C 8.160262 -2.022052 1.106713
C 6.806922 -3.698835 -0.009334
C 7.965437 -3.336109 0.675222
H 6.179525 3.078458 0.860604
H 2.554048 5.162955 -0.196131
H 7.344732 -0.049236 1.186472
H 4.938047 -3.045995 -0.798975
H 9.063477 -1.752551 1.639977
H 6.669121 -4.719807 -0.342827
H 8.725427 -4.082883 0.875128
H 4.440400 6.142599 0.673106
H 0.901747 3.881900 -1.004889
H 3.202250 -1.000837 -2.609132
O 3.085254 -1.704717 -0.041945
H 2.138596 -1.687149 -0.245750

SCF Energy: -2179.77470967

Sum of electronic and zero-point Energies= -2179.312921
Sum of electronic and thermal Energies= -2179.276135
Sum of electronic and thermal Enthalpies= -2179.275191
Sum of electronic and thermal Free Energies= -2179.384374

==> ./AOX-II/bis/c4c5/RAF/c4 <==

O	-5.610750	0.850171	0.040991
O	-3.605900	-1.841091	-1.256210
O	-1.277105	2.723874	-0.660487
O	-1.827902	0.042879	-1.113072
O	-4.852252	5.298233	1.172352
C	-3.389780	1.695414	-0.311834
C	-4.725951	1.866905	0.106007
C	-5.286510	-0.381302	-0.422806
C	-2.983580	0.395691	-0.767271
C	-4.008780	-0.625308	-0.828436
C	-6.408927	-1.321352	-0.375225
C	-2.548089	2.839943	-0.229007
C	-5.224067	3.061870	0.601390
C	-4.359077	4.141923	0.683193
C	-3.026431	4.036574	0.265457
C	-7.430151	-1.134562	0.567611
C	-6.495753	-2.401698	-1.263436
C	-8.501326	-2.014687	0.627632
C	-7.575312	-3.273155	-1.202600
C	-8.578097	-3.087388	-0.256278
H	-6.257224	3.140956	0.912668
H	-2.369584	4.898682	0.319148
H	-7.378979	-0.303491	1.260600
H	-5.731769	-2.552110	-2.013939
H	-9.278625	-1.862211	1.368376
H	-7.633575	-4.099376	-1.902680
H	-9.417298	-3.772922	-0.209501
H	-2.651452	-1.752722	-1.422462
H	-4.166290	5.975779	1.193878
Cu	-0.050638	0.490264	-0.232738
H	-0.810258	3.560093	-0.541297
O	5.233966	-1.213853	0.044698
O	4.001181	2.056417	0.071470
O	0.652295	-1.697979	1.345673
O	1.805443	0.759927	0.587666
O	3.489524	-5.470345	0.695287
C	2.895467	-1.365725	0.699989
C	4.128736	-1.975755	0.412716
C	5.309526	0.072447	0.063443
C	2.811719	0.057619	0.636172
C	4.149460	0.840132	0.690264
C	6.520103	0.627497	-0.444783
C	1.824401	-2.241151	1.018544
C	4.347530	-3.322665	0.398001
C	3.258966	-4.154011	0.713460
C	2.014637	-3.618051	1.022983
C	7.413361	-0.221705	-1.142359
C	6.864412	1.985219	-0.252643
C	8.597128	0.277966	-1.639529
C	8.062993	2.465817	-0.741227
C	8.924184	1.620116	-1.437881
H	5.313086	-3.734227	0.137821
H	1.187873	-4.272328	1.278423
H	7.154324	-1.259822	-1.302310
H	6.209972	2.648630	0.293486
H	9.270610	-0.370839	-2.185723
H	8.329991	3.503027	-0.581346
H	9.858481	2.008471	-1.827095
H	2.683834	-5.963902	0.895896
H	0.008572	-2.383005	1.570372
H	3.068130	2.307825	0.166762
O	4.420324	0.924414	2.068330
H	5.163317	1.524942	2.215447

SCF Energy: -2179.77535996
Sum of electronic and zero-point Energies= -2179.314007
Sum of electronic and thermal Energies= -2179.277044
Sum of electronic and thermal Enthalpies= -2179.276100
Sum of electronic and thermal Free Energies= -2179.386539

==> ./AOX-II/bis/c4c5/RAF/c1p <==

O	5.818148	-1.030478	-0.123555
O	4.161848	2.034114	0.769822
O	1.229905	-2.206867	0.464956
O	2.141244	0.413725	0.709784
O	4.502860	-5.442713	-0.782211
C	3.481700	-1.517289	0.159390
C	4.802573	-1.918193	-0.132448
C	5.649361	0.281888	0.170025
C	3.241960	-0.129460	0.444092
C	4.398931	0.743873	0.451220
C	6.909087	1.030106	0.167761
C	2.493984	-2.542180	0.146790
C	5.154259	-3.221211	-0.449330
C	4.151941	-4.178123	-0.470109
C	2.825676	-3.844607	-0.169451
C	8.112122	0.355919	0.423766
C	6.946737	2.406159	-0.095228
C	9.317048	1.044142	0.430358
C	8.157806	3.086104	-0.095029
C	9.345059	2.411599	0.171662
H	6.182422	-3.475277	-0.670541

H	2.057066	-4.611448	-0.174423
H	8.101212	-0.707727	0.628416
H	6.034911	2.943679	-0.316497
H	10.237341	0.509837	0.639634
H	8.171887	4.149287	-0.308432
H	10.287665	2.948055	0.175611
H	3.206227	2.086027	0.942738
H	3.729117	-6.017894	-0.765236
Cu	0.244631	0.167899	0.056466
H	0.659991	-2.982975	0.404055
O	-5.434887	0.968967	-0.166505
O	-3.517524	-2.053866	-0.312990
O	-0.892258	2.322685	-0.742341
O	-1.632267	-0.343690	-0.529244
O	-4.427567	5.493864	-0.363147
C	-3.112719	1.556600	-0.431530
C	-4.477509	1.907627	-0.289348
C	-5.173954	-0.362958	-0.165069
C	-2.762358	0.175669	-0.431115
C	-3.872022	-0.786534	-0.309953
C	-6.327803	-1.187744	0.028015
C	-2.185871	2.633257	-0.568447
C	-4.921349	3.216193	-0.268409
C	-3.977662	4.228945	-0.387829
C	-2.613961	3.943116	-0.532959
C	-7.600767	-0.542611	0.507770
C	-6.343436	-2.538905	-0.292493
C	-8.746663	-1.454159	0.726738
C	-7.502718	-3.288423	-0.137628
C	-8.711827	-2.758390	0.369671
H	-5.974244	3.439095	-0.159399
H	-1.895842	4.750182	-0.633646
H	-7.929278	0.048229	-0.373610
H	-5.463413	-3.024974	-0.682583
H	-9.630805	-1.001674	1.160865
H	-7.470815	-4.335899	-0.415808
H	-9.572150	-3.401883	0.497849
H	-3.697983	6.116963	-0.465684
H	-0.360880	3.126671	-0.801650
H	-2.541510	-2.060842	-0.396134
O	-7.441629	0.375278	1.561464
H	-7.233973	-0.117131	2.363377

SCF Energy: -2179.75354242
Sum of electronic and zero-point Energies= -2179.292855
Sum of electronic and thermal Energies= -2179.255791
Sum of electronic and thermal Enthalpies= -2179.254846
Sum of electronic and thermal Free Energies= -2179.365819

==> ./AOX-II/bis/c4c5 <==

O	5.478159	-1.116263	-0.050317
O	3.942692	2.125369	-0.236960
O	0.790606	-1.965680	-0.367841
O	1.840568	0.609090	-0.279933
O	3.957620	-5.509954	-0.009898
C	3.107818	-1.444446	-0.192024
C	4.421079	-1.949822	-0.090620
C	5.361370	0.227090	-0.108795
C	2.935060	-0.027271	-0.233113
C	4.119030	0.788603	-0.207987
C	6.647745	0.918342	-0.011105
C	2.066477	-2.407756	-0.240109
C	4.714062	-3.302059	-0.028116
C	3.659661	-4.199842	-0.072249
C	2.332544	-3.754913	-0.183153
C	7.706320	0.307823	0.677163
C	6.852566	2.172702	-0.601336
C	8.933687	0.945385	0.783932
C	8.087507	2.799044	-0.498301
C	9.128743	2.192605	0.197081
H	5.738761	-3.639316	0.052389
H	1.518313	-4.470885	-0.226779
H	7.562182	-0.661543	1.138758
H	6.057232	2.650939	-1.156362
H	9.740352	0.466639	1.327892
H	8.236080	3.765022	-0.968103
H	10.089643	2.688666	0.279869
H	2.984647	2.277901	-0.262057
H	3.156627	-6.046196	-0.042281
Cu	0.000111	0.000059	-0.000066
H	0.152407	-2.687541	-0.289522
O	-5.478198	1.116241	0.050283
O	-3.942613	-2.125341	0.237190
O	-0.790670	1.965658	0.367395
O	-1.840535	-0.608985	0.279746
O	-3.957663	5.509949	0.009276
C	-3.107864	1.444479	0.191765
C	-4.421135	1.949821	0.090412
C	-5.361379	-0.227117	0.108946
C	-2.935045	0.027308	0.232974
C	-4.119014	-0.788586	0.208076
C	-6.647760	-0.918390	0.011486
C	-2.066520	2.407783	0.239673
C	-4.714114	3.302056	0.027784
C	-3.659700	4.199838	0.071734
C	-2.332569	3.754934	0.182575
C	-7.706418	-0.308004	-0.676779
C	-6.852535	-2.172639	0.601975
C	-8.933793	-0.945587	-0.783304
C	-8.087485	-2.799006	0.499176
C	-9.128794	-2.192703	-0.196212
H	-5.738812	3.639328	-0.052673

H	-1.518329	4.470903	0.226060
H	-7.562343	0.661277	-1.138572
H	-6.057157	-2.650779	1.157018
H	-9.740511	-0.466930	-1.327266
H	-8.235996	-3.764898	0.969173
H	-10.089703	-2.688778	-0.278809
H	-2.984554	-2.277819	0.262133
H	-3.156664	6.046189	0.041578
H	-0.152384	2.687398	0.288632

SCF Energy: -2103.98503007

Sum of electronic and zero-point Energies= -2103.538619

Sum of electronic and thermal Energies= -2103.503968

Sum of electronic and thermal Enthalpies= -2103.503024

Sum of electronic and thermal Free Energies= -2103.607729

==> ./AOX-II/bis/c4c5/vIP <==

O	5.478159	-1.116263	-0.050317
O	3.942692	2.125369	-0.236960
O	0.790606	-1.965680	-0.367841
O	1.840568	0.609090	-0.279933
O	3.957620	-5.509954	-0.009898
C	3.107818	-1.444446	-0.192024
C	4.421079	-1.949822	-0.090620
C	5.361370	0.227090	-0.108795
C	2.935060	-0.027271	-0.233113
C	4.119030	0.788603	-0.207987
C	6.647745	0.918342	-0.011105
C	2.066477	-2.407756	-0.240109
C	4.714062	-3.302059	-0.028116
C	3.659661	-4.199842	-0.072249
C	2.332544	-3.754913	-0.183153
C	7.706320	0.307823	0.677163
C	6.852566	2.172702	-0.601336
C	8.933687	0.945385	0.783932
C	8.087507	2.799044	-0.498301
C	9.128743	2.192605	0.197081
H	5.738761	-3.639316	0.052389
H	1.518313	-4.470885	-0.226779
H	7.562182	-0.661543	1.138758
H	6.057232	2.650939	-1.156362
H	9.740352	0.466639	1.327892
H	8.236080	3.765022	-0.968103
H	10.089643	2.688666	0.279869
H	2.984647	2.277901	-0.262057
H	3.156627	-6.046196	-0.042281
Cu	0.000111	0.000059	-0.000066
H	0.152407	-2.687541	-0.289522
O	-5.478198	1.116241	0.050283
O	-3.942613	-2.125341	0.237190
O	-0.790670	1.965658	0.367395
O	-1.840535	-0.608985	0.279746
O	-3.957663	5.509949	0.009276
C	-3.107864	1.444479	0.191765
C	-4.421135	1.949821	0.090412
C	-5.361379	-0.227117	0.108946
C	-2.935045	0.027308	0.232974
C	-4.119014	-0.788586	0.208076
C	-6.647760	-0.918390	0.011486
C	-2.066520	2.407783	0.239673
C	-4.714114	3.302056	0.027784
C	-3.659700	4.199838	0.071734
C	-2.332569	3.754934	0.182575
C	-7.706418	-0.308004	-0.676779
C	-6.852535	-2.172639	0.601975
C	-8.933793	-0.945587	-0.783304
C	-8.087485	-2.799006	0.499176
C	-9.128794	-2.192703	-0.196212
H	-5.738812	3.639328	-0.052673
H	-1.518329	4.470903	0.226060
H	-7.562343	0.661277	-1.138572
H	-6.057157	-2.650779	1.157018
H	-9.740511	-0.466930	-1.327266
H	-8.235996	-3.764898	0.969173
H	-10.089703	-2.688778	-0.278809
H	-2.984554	-2.277819	0.262133
H	-3.156664	6.046189	0.041578
H	-0.152384	2.687398	0.288632

SCF Energy: -2103.75770648

==> ./AOX-II/bis/c4c5/vEA <==

O	5.478159	-1.116263	-0.050317
O	3.942692	2.125369	-0.236960
O	0.790606	-1.965680	-0.367841
O	1.840568	0.609090	-0.279933
O	3.957620	-5.509954	-0.009898
C	3.107818	-1.444446	-0.192024
C	4.421079	-1.949822	-0.090620
C	5.361370	0.227090	-0.108795
C	2.935060	-0.027271	-0.233113
C	4.119030	0.788603	-0.207987
C	6.647745	0.918342	-0.011105
C	2.066477	-2.407756	-0.240109
C	4.714062	-3.302059	-0.028116
C	3.659661	-4.199842	-0.072249
C	2.332544	-3.754913	-0.183153
C	7.706320	0.307823	0.677163
C	6.852566	2.172702	-0.601336
C	8.933687	0.945385	0.783932
C	8.087507	2.799044	-0.498301

C	9.128743	2.192605	0.197081
H	5.738761	-3.639316	0.052389
H	1.518313	-4.470885	-0.226779
H	7.562182	-0.661543	1.138758
H	6.057232	2.650939	-1.156362
H	9.740352	0.466639	1.327892
H	8.236080	3.765022	-0.968103
H	10.089643	2.688666	0.279869
H	2.984647	2.277901	-0.262057
H	3.156627	-6.046196	-0.042281
Cu	0.000111	0.000059	-0.000066
H	0.152407	-2.687541	-0.289522
O	-5.478198	1.116241	0.050283
O	-3.942613	-2.125341	0.237190
O	-0.790670	1.965658	0.367395
O	-1.840535	-0.608985	0.279746
O	-3.957663	5.509949	0.009276
C	-3.107864	1.444479	0.191765
C	-4.421135	1.949821	0.090412
C	-5.361379	-0.227117	0.108946
C	-2.935045	0.027308	0.232974
C	-4.119014	-0.788586	0.208076
C	-6.647760	-0.918390	0.011486
C	-2.066520	2.407783	0.239673
C	-4.714114	3.302056	0.027784
C	-3.659700	4.199838	0.071734
C	-2.332569	3.754934	0.182575
C	-7.706418	-0.308004	-0.676779
C	-6.852535	-2.172639	0.601975
C	-8.933793	-0.945587	-0.783304
C	-8.087485	-2.799006	0.499176
C	-9.128794	-2.192703	-0.196212
H	-5.738812	3.639328	-0.052673
H	-1.518329	4.470903	0.226060
H	-7.562343	0.661277	-1.138572
H	-6.057157	-2.650779	1.157018
H	-9.740511	-0.466930	-1.327266
H	-8.235996	-3.764898	0.969173
H	-10.089703	-2.688778	-0.278809
H	-2.984554	-2.277819	0.262133
H	-3.156664	6.046189	0.041578
H	-0.152384	2.687398	0.288632

SCF Energy: -2104.14598039

==> ./AOX-II/bis/c3c4/aEA <==

O	5.615632	-0.149343	-0.049098
O	2.392029	1.513042	0.120904
O	2.326985	-3.636161	-0.001003
O	1.672901	-1.090609	0.107111
O	6.950414	-4.611820	-0.345510
C	3.903784	-1.814875	-0.020058
C	5.259169	-1.453238	-0.091099
C	4.702327	0.842740	0.053275
C	2.898187	-0.796738	0.064335
C	3.364233	0.570141	0.083688
C	5.324262	2.174173	0.077739
C	3.597697	-3.203802	-0.061039
C	6.285323	-2.375824	-0.198217
C	5.940764	-3.722015	-0.236607
C	4.606999	-4.140452	-0.165575
C	6.377822	2.460369	-0.799249
C	4.899298	3.154005	0.982605
C	6.979898	3.710536	-0.781416
C	5.510156	4.402106	0.997850
C	6.546922	4.684391	0.114935
H	7.317362	-2.055286	-0.250066
H	4.358671	-5.195376	-0.196235
H	6.715801	1.704830	-1.499199
H	4.108962	2.935153	1.692383
H	7.789325	3.925897	-1.470104
H	5.178826	5.151064	1.708394
H	7.020601	5.659902	0.127524
H	2.743906	2.388834	-0.075999
H	1.745730	-2.850809	0.057558
H	6.605992	-5.512283	-0.372591
Cu	0.000395	-0.000205	0.110524
O	-5.616041	0.149811	-0.045451
O	-2.392151	-1.514018	0.100073
O	-2.325810	3.635285	-0.006055
O	-1.672746	1.089218	0.103451
O	-6.949382	4.612834	-0.342233
C	-3.903405	1.814576	-0.021841
C	-5.259119	1.453529	-0.089505
C	-4.702458	-0.842748	0.050364
C	-2.898005	0.795911	0.059281
C	-3.364145	-0.570676	0.073810
C	-5.324697	-2.174018	0.076972
C	-3.596825	3.203410	-0.063302
C	-6.285131	2.376541	-0.194493
C	-5.940039	3.722545	-0.234493
C	-4.605944	4.140423	-0.166149
C	-6.383181	-2.458848	-0.794506
C	-4.895418	-3.154873	0.978696
C	-6.986033	-3.708611	-0.774370
C	-5.507067	-4.402550	0.996325
C	-6.548854	-4.683433	0.118886
H	-7.317418	2.056411	-0.243914
H	-4.357163	5.195218	-0.197653
H	-6.724410	-1.702527	-1.492044
H	-4.101068	-2.936997	1.684293
H	-7.799320	-3.922921	-1.458823
H	-5.172411	-5.152268	1.704512
H	-7.023164	-5.658613	0.133363
H	-2.746289	-2.389089	-0.096072

H	-1.744796	2.849748	0.052745
H	-6.604356	5.512994	-0.371847

SCF Energy: -2104.16597254
Sum of electronic and zero-point Energies= -2103.722239
Sum of electronic and thermal Energies= -2103.688298
Sum of electronic and thermal Enthalpies= -2103.687354
Sum of electronic and thermal Free Energies= -2103.790283

==> ./AOX-II/bis/c3c4/aIP <==

O	5.425726	-0.025775	-0.033393
O	2.112732	1.399644	0.074759
O	2.372205	-3.713387	0.082701
O	1.553133	-1.195950	0.108226
O	7.036573	-4.385881	-0.184200
C	3.819834	-1.792703	0.031197
C	5.149109	-1.348547	-0.039553
C	4.483754	0.918658	0.032260
C	2.771854	-0.837838	0.079059
C	3.146341	0.553991	0.067709
C	5.030810	2.271517	0.007298
C	3.598778	-3.208400	0.024896
C	6.227764	-2.205155	-0.110541
C	5.969589	-3.586129	-0.110741
C	4.669993	-4.084247	-0.040602
C	6.147452	2.533988	-0.801564
C	4.490242	3.298999	0.794196
C	6.693710	3.806246	-0.837600
C	5.053500	4.566318	0.760538
C	6.148876	4.824301	-0.057873
H	7.238892	-1.825434	-0.166857
H	4.488789	-5.152502	-0.043443
H	6.568028	1.743397	-1.411219
H	3.660136	3.102604	1.463320
H	7.546664	4.006155	-1.475832
H	4.640783	5.351190	1.383611
H	6.581847	5.817945	-0.085270
H	2.340106	2.309943	-0.156894
H	1.720855	-2.984544	0.113177
H	6.771746	-5.314572	-0.187127
Cu	0.000191	-0.007345	0.096025
O	-5.426315	0.031111	-0.034435
O	-2.119568	-1.409120	0.074003
O	-2.356290	3.704868	0.084049
O	-1.548754	1.183936	0.109786
O	-7.017540	4.398561	-0.183538
C	-3.812555	1.790703	0.031379
C	-5.143778	1.352636	-0.039986
C	-4.488606	-0.917617	0.031096
C	-2.768976	0.831049	0.079517
C	-3.149518	-0.559007	0.066865
C	-5.041787	-2.267926	0.006222
C	-3.585082	3.205423	0.025659
C	-6.218515	2.214193	-0.111012
C	-5.954115	3.594016	-0.110466
C	-4.652270	4.086130	-0.039857
C	-6.160327	-2.525395	-0.801621
C	-4.505165	-3.297678	0.792803
C	-6.712339	-3.795181	-0.837000
C	-5.074172	-4.562443	0.759833
C	-6.171420	-4.815571	-0.057582
H	-7.231331	1.839057	-0.167680
H	-4.466181	5.153569	-0.042170
H	-6.577842	-1.732955	-1.410980
H	-3.673504	-3.104956	1.461012
H	-7.566757	-3.991345	-1.474433
H	-4.664472	-5.349103	1.382645
H	-6.608925	-5.807244	-0.084428
H	-2.350238	-2.317497	-0.161901
H	-1.708306	2.973043	0.114525
H	-6.748938	5.326205	-0.185622

SCF Energy: -2103.75741713
Sum of electronic and zero-point Energies= -2103.310994
Sum of electronic and thermal Energies= -2103.277449
Sum of electronic and thermal Enthalpies= -2103.276504
Sum of electronic and thermal Free Energies= -2103.376384

==> ./AOX-II/bis/c3c4/HAT/c5 <==

O	-5.515134	-0.427505	-0.230496
O	-2.480773	1.355385	0.667403
O	-1.922225	-3.548209	-0.806844
O	-1.519377	-1.071929	-0.004448
O	-6.425425	-4.803857	-1.567430
C	-3.659145	-1.907574	-0.491102
C	-5.040310	-1.655357	-0.538614
C	-4.702248	0.588450	0.135995
C	-2.761691	-0.868048	-0.082573
C	-3.352633	0.407397	0.246020
C	-5.447487	1.821852	0.422617
C	-3.224768	-3.220003	-0.831086
C	-5.973817	-2.611418	-0.897658
C	-5.505430	-3.879139	-1.222382
C	-4.140417	-4.188257	-1.193141
C	-6.635613	1.755364	1.160559
C	-5.003339	3.059609	-0.055662
C	-7.354270	2.911708	1.428871
C	-5.731297	4.212645	0.212414

C	-6.903811	4.142071	0.957038
H	-7.029533	-2.376199	-0.922426
H	-3.796547	-5.183887	-1.449607
H	-6.987078	0.798730	1.529816
H	-4.103599	3.119879	-0.658537
H	-8.268727	2.852785	2.008619
H	-5.384218	5.165780	-0.170484
H	-7.469032	5.043628	1.166630
H	-2.936216	2.105152	1.067231
H	-1.418743	-2.760393	-0.517674
H	-5.997982	-5.645344	-1.765011
Cu	0.021729	0.030271	0.663638
O	5.377681	0.444675	-0.381125
O	3.102684	-1.334934	1.632700
O	1.658075	3.383597	0.019236
O	1.730074	0.847386	1.321201
O	5.557344	4.614774	-2.367183
C	3.437572	1.820506	-0.064800
C	4.710679	1.602883	-0.582878
C	4.910097	-0.565964	0.316609
C	2.832023	0.808396	0.721688
C	3.611986	-0.408627	0.893009
C	5.793186	-1.692388	0.439111
C	2.792361	3.115049	-0.376822
C	5.404003	2.532445	-1.339369
C	4.804724	3.785841	-1.634982
C	3.545495	4.065022	-1.180571
C	7.159466	-1.516097	0.124027
C	5.336560	-2.965829	0.840913
C	8.036927	-2.575459	0.225658
C	6.226115	-4.020959	0.923341
C	7.573314	-3.830346	0.624051
H	6.399344	2.301999	-1.698767
H	3.069907	5.013253	-1.402706
H	7.522221	-0.541494	-0.175203
H	4.293280	-3.136360	1.058329
H	9.086048	-2.429341	-0.001283
H	5.867635	-4.999501	1.218860
H	8.265617	-4.661149	0.699785
H	5.098111	5.450032	-2.523046
H	2.226579	-1.013129	1.944178

SCF Energy: -2103.29966269
Sum of electronic and zero-point Energies= -2102.867939
Sum of electronic and thermal Energies= -2102.832580
Sum of electronic and thermal Enthalpies= -2102.831636
Sum of electronic and thermal Free Energies= -2102.940596

==> ./AOX-II/bis/c3c4/HAT/c3 <==

O	5.569787	0.209346	0.025728
O	2.469809	-1.679629	-0.099429
O	2.038862	3.448626	-0.061324
O	1.571546	0.860664	-0.123554
O	6.584756	4.759150	0.222526
C	3.743742	1.747524	-0.025796
C	5.121898	1.484910	0.041609
C	4.730209	-0.847903	-0.047665
C	2.815171	0.657791	-0.084776
C	3.376058	-0.672654	-0.083623
C	5.444797	-2.130911	-0.031720
C	3.338356	3.111626	-0.007251
C	6.079959	2.480254	0.120930
C	5.640029	3.798774	0.137096
C	4.278849	4.119791	0.070534
C	6.523153	-2.310002	0.843642
C	5.079623	-3.171497	-0.893165
C	7.208872	-3.515948	0.867329
C	5.773849	-4.375287	-0.866459
C	6.834976	-4.551788	0.014917
H	7.132709	2.235909	0.171743
H	3.957088	5.154914	0.085615
H	6.814715	-1.506570	1.510266
H	4.273992	-3.034717	-1.606560
H	8.037164	-3.648170	1.554555
H	5.488981	-5.172213	-1.544168
H	7.374309	-5.492411	0.034603
H	2.886829	-2.532611	0.068735
H	1.516476	2.621279	-0.095547
H	6.179381	5.634020	0.243115
Cu	-0.037183	-0.344060	-0.120262
O	-5.536068	0.082848	0.177405
O	-2.269877	1.203532	0.559271
O	-2.791739	-3.846072	-0.313848
O	-1.767672	-1.362674	-0.338063
O	-7.443638	-4.151145	0.401947
C	-4.055464	-1.827153	-0.033426
C	-5.340660	-1.287947	0.165764
C	-4.592921	0.969599	0.109821
C	-2.925050	-0.976580	-0.108088
C	-3.165844	0.486300	0.194523
C	-4.986922	2.322991	-0.006617
C	-3.957886	-3.247340	-0.110451
C	-6.468877	-2.039088	0.301702
C	-6.324914	-3.444427	0.247231
C	-5.088847	-4.040732	0.038037
C	-6.359966	2.654712	0.136086
C	-4.048751	3.345265	-0.298779
C	-6.766365	3.962657	0.016689
C	-4.477760	4.649077	-0.429557
C	-5.827435	4.960122	-0.266683
H	-7.439600	-1.582559	0.439126
H	-4.996014	-5.119001	-0.010169

H -7.079448 1.878632 0.360086
H -3.006018 3.108352 -0.446070
H -7.810924 4.219390 0.140713
H -3.764893 5.429075 -0.665077
H -6.155193 5.988550 -0.366696
H -2.088580 -3.175829 -0.414026
H -7.260082 -5.098282 0.350958

SCF Energy: -2103.33917326
Sum of electronic and zero-point Energies= -2102.905235
Sum of electronic and thermal Energies= -2102.870248
Sum of electronic and thermal Enthalpies= -2102.869304
Sum of electronic and thermal Free Energies= -2102.975813

==> ./AOX-II/bis/c3c4/HAT/c7 <==
O 5.480719 0.334840 0.025101
O 2.413585 -1.572269 0.384909
O 2.032610 2.971704 -2.012421
O 1.541227 0.636460 -0.884901
O 6.502803 4.518950 -1.766128
C 3.694166 1.576029 -0.963109
C 5.047325 1.449492 -0.606021
C 4.651033 -0.685805 0.334749
C 2.771237 0.537174 -0.618051
C 3.316034 -0.616866 0.055168
C 5.344429 -1.785896 1.017716
C 3.305677 2.770978 -1.631889
C 5.994631 2.422807 -0.869453
C 5.570572 3.575378 -1.520320
C 4.236692 3.752934 -1.906968
C 6.288981 -1.493751 2.009562
C 5.096135 -3.120680 0.677848
C 6.957618 -2.520976 2.659843
C 5.773709 -4.143941 1.329999
C 6.700992 -3.847629 2.323119
H 7.027170 2.287543 -0.576085
H 3.929595 4.658464 -2.417940
H 6.488930 -0.461959 2.275040
H 4.397433 -3.362098 -0.115714
H 7.680974 -2.285446 3.432515
H 5.582105 -5.174268 1.052020
H 7.227017 -4.648411 2.831160
H 2.793773 -2.218657 0.990869
H 1.506112 2.196212 -1.733043
H 6.104378 5.277585 -2.207866
Cu -0.015465 -0.447265 -0.228154
O -5.503800 0.015887 -0.011690
O -2.474610 0.893696 1.572612
O -2.374880 -3.520254 -0.997229
O -1.699512 -1.377679 0.399833
O -6.831086 -3.728337 -2.422711
C -3.876159 -1.711940 -0.467586
C -5.190271 -1.189765 -0.560205
C -4.648306 0.756635 0.665389
C -2.890223 -0.987683 0.224800
C -3.321560 0.252818 0.833290
C -5.185957 1.981433 1.178227
C -3.600800 -3.013330 -1.083580
C -6.184292 -1.836866 -1.211650
C -5.911111 -3.152878 -1.841674
C -4.579524 -3.689784 -1.738370
C -6.592079 2.149016 1.177558
C -4.367951 3.032555 1.653273
C -7.152514 3.313799 1.654903
C -4.945449 4.199316 2.113883
C -6.332074 4.342308 2.123160
H -7.179573 -1.417471 -1.284760
H -4.370662 -4.648103 -2.196002
H -7.229340 1.350159 0.822842
H -3.292677 2.947104 1.635498
H -8.229458 3.428141 1.667884
H -4.313540 5.006091 2.464362
H -6.776696 5.258412 2.494765
H -1.798913 -2.912441 -0.488763
H -1.620358 0.416545 1.588997

SCF Energy: -2103.29636068
Sum of electronic and zero-point Energies= -2102.863411
Sum of electronic and thermal Energies= -2102.828556
Sum of electronic and thermal Enthalpies= -2102.827612
Sum of electronic and thermal Free Energies= -2102.934066

==> ./AOX-II/bis/c3c4/RAF/c8 <==
O 5.710090 0.030999 0.357769
O 2.577919 -1.751515 -0.083577
O 2.768229 3.059749 -1.911061
O 2.028670 0.624194 -1.235863
O 7.153204 4.349536 -0.661165
C 4.173017 1.466278 -0.774393
C 5.430865 1.233696 -0.193745
C 4.807947 -0.975668 0.382598
C 3.175918 0.437712 -0.745541
C 3.550941 -0.810273 -0.124694
C 5.331485 -2.180927 1.039837
C 3.944773 2.752448 -1.339430
C 6.434588 2.185440 -0.152176
C 6.168121 3.428435 -0.714341
C 4.933796 3.715621 -1.309648

C 6.061514 -2.055631 2.228395
C 5.134388 -3.450607 0.485702
C 6.568388 -3.183731 2.857401
C 5.649320 -4.575676 1.119010
C 6.362451 -4.445711 2.305714
H 7.389781 1.966056 0.306204
H 4.746930 4.691711 -1.743095
H 6.221299 -1.074626 2.661037
H 4.604667 -3.560607 -0.454609
H 7.125307 -3.077671 3.781747
H 5.500083 -5.554054 0.676178
H 6.762089 -5.325322 2.798348
H 2.840580 -2.499930 0.464409
H 2.184824 2.277595 -1.835805
H 6.867012 5.176146 -1.067173
Cu 0.296986 -0.344489 -1.010729
O -5.286824 0.299082 0.236570
O -1.955615 1.618128 0.295751
O -2.645828 -3.241756 -1.394185
O -1.592768 -0.951570 -0.732284
O -7.238639 -3.825754 -0.757709
C -3.892909 -1.386599 -0.583984
C -5.123714 -0.908847 -0.233075
C -4.255269 1.173080 0.418647
C -2.739883 -0.538830 -0.435292
C -2.981126 0.783044 0.092090
C -4.696048 2.439771 0.997140
C -3.798386 -2.752898 -1.056041
C -6.394630 -1.669371 -0.428716
C -6.147590 -3.112779 -0.779979
C -4.916053 -3.588210 -1.140687
C -5.833398 2.459070 1.818330
C -4.021008 3.639414 0.734301
C -6.274413 3.650710 2.373570
C -4.476999 4.828770 1.286283
C -5.598374 4.838791 2.109574
H -4.783324 -4.616863 -1.451854
H -6.363718 1.538293 2.029924
H -3.156343 3.649150 0.085154
H -7.148476 3.651220 3.015130
H -3.953520 5.752712 1.067472
H -5.946890 5.770012 2.542496
H -1.942927 -2.558722 -1.258754
H -7.077457 -4.743477 -1.024870
H -1.119775 1.181598 0.070004
H -6.835432 -1.241439 -1.345928
O -7.247861 -1.495970 0.663602
H -8.158205 -1.511710 0.351217

SCF Energy: -2179.79416152
Sum of electronic and zero-point Energies= -2179.331662
Sum of electronic and thermal Energies= -2179.295135
Sum of electronic and thermal Enthalpies= -2179.294191
Sum of electronic and thermal Free Energies= -2179.403901

==> ./AOX-II/bis/c3c4/RAF/c2 <==
O -5.627840 -0.275628 -0.107752
O -2.411776 1.328846 0.408601
O -2.327438 -3.709473 -0.658327
O -1.682214 -1.213385 -0.109252
O -6.953525 -4.658202 -1.013512
C -3.910056 -1.914781 -0.368657
C -5.268837 -1.557483 -0.345043
C -4.717142 0.697918 0.115982
C -2.909673 -0.921222 -0.115794
C -3.380081 0.419706 0.140494
C -5.344226 2.002690 0.367447
C -3.600121 -3.280612 -0.622787
C -6.294409 -2.461606 -0.559164
C -5.946052 -3.784574 -0.805864
C -4.609067 -4.198082 -0.838404
C -6.471351 2.074351 1.195656
C -4.849471 3.172695 -0.220073
C -7.078570 3.297217 1.443054
C -5.466271 4.393168 0.028015
C -6.577182 4.459139 0.861881
H -7.328610 -2.144959 -0.534070
H -4.358906 -5.235267 -1.030839
H -6.862797 1.172195 1.651674
H -4.000669 3.128973 -0.893876
H -7.945462 3.343486 2.092819
H -5.080802 5.291863 -0.440303
H -7.055476 5.413077 1.055393
H -2.783184 2.146637 0.757898
H -1.746154 -2.944282 -0.475341
H -6.603919 -5.542834 -1.173608
Cu -0.067026 -0.174926 0.529817
O 5.309701 0.471287 -0.657515
O 2.898901 -1.734352 0.733650
O 1.510530 3.190152 0.535408
O 1.664487 0.577397 1.192504
O 5.149941 4.942697 -1.893230
C 3.331635 1.736241 -0.063870
C 4.604047 1.622617 -0.700825
C 4.690674 -0.796146 -0.515419
C 2.729495 0.612618 0.523851
C 3.412387 -0.676105 0.298541
C 5.693306 -1.701019 0.177297
C 2.688988 3.021220 -0.058158
C 5.193227 2.696509 -1.307026
C 4.523231 3.935399 -1.291374

C 3.278556 4.101556 -0.678183
C 6.541404 -1.180651 1.155973
C 5.729778 -3.060909 -0.126639
C 7.437821 -2.017034 1.809274
C 6.632685 -3.891120 0.527444
C 7.486618 -3.371583 1.494777
H 6.162266 2.605059 -1.778718
H 2.785492 5.065884 -0.676526
H 6.507880 -0.125794 1.400933
H 5.062999 -3.473127 -0.874970
H 8.100524 -1.607637 2.563479
H 6.666169 -4.946089 0.279493
H 8.189387 -4.022073 2.003860
H 1.224603 2.360609 0.961518
H 4.635060 5.758918 -1.840415
H 2.049948 -1.546413 1.202513
O 4.224383 -1.254745 -1.738768
H 4.976140 -1.522850 -2.284480

SCF Energy: -2179.77061409
Sum of electronic and zero-point Energies= -2179.309656
Sum of electronic and thermal Energies= -2179.273165
Sum of electronic and thermal Enthalpies= -2179.272220
Sum of electronic and thermal Free Energies= -2179.382468

==> ./AOX-II/bis/c3c4/RAF/c3p <==
O -5.856315 -0.446990 -0.081007
O -2.782077 1.401929 0.479386
O -2.287497 -3.575092 -0.759076
O -1.847379 -1.057686 -0.131615
O -6.824813 -4.898575 -1.087194
C -4.010937 -1.930034 -0.397258
C -5.394046 -1.688340 -0.352379
C -5.026396 0.592369 0.161730
C -3.093298 -0.864082 -0.120235
C -3.671025 0.424880 0.179922
C -5.757018 1.838644 0.431264
C -3.591580 -3.256655 -0.698357
C -6.343373 -2.668606 -0.582046
C -5.889532 -3.950418 -0.869474
C -4.523112 -4.248973 -0.932287
C -6.878325 1.813718 1.269645
C -5.366318 3.046648 -0.157500
C -7.581627 2.981866 1.527551
C -6.078517 4.211581 0.101330
C -7.182858 4.182846 0.946155
H -7.400000 -2.440212 -0.537899
H -4.191313 -5.255927 -1.158991
H -7.188929 0.880306 1.725088
H -4.519943 3.072874 -0.835658
H -8.443195 2.955295 2.185502
H -5.772916 5.140774 -0.366476
H -7.735459 5.093786 1.148746
H -3.221487 2.174138 0.852941
H -1.771789 -2.767504 -0.559259
H -6.406368 -5.748541 -1.267022
Cu -0.260250 0.076193 0.251827
O 5.307484 0.615639 -0.347954
O 2.617836 -1.354889 0.981095
O 1.613834 3.660293 -0.064652
O 1.408018 1.109937 0.636749
O 5.851892 5.061814 -1.645263
C 3.409638 2.056540 -0.169948
C 4.760828 1.842900 -0.498799
C 4.600430 -0.430660 0.125762
C 2.620066 0.971807 0.318033
C 3.287474 -0.305496 0.470427
C 5.422379 -1.642301 0.254055
C 2.891232 3.370015 -0.357653
C 5.585993 2.836350 -0.991478
C 5.037597 4.103173 -1.161751
C 3.700129 4.375228 -0.846977
C 6.763412 -1.524746 0.550313
C 4.882603 -2.935166 0.099092
C 7.617976 -2.694396 0.584560
C 5.650895 -4.094756 0.232997
C 6.981922 -4.002773 0.542213
H 6.619662 2.631407 -1.236073
H 3.296189 5.371518 -0.985871
H 7.237064 -0.560992 0.692900
H 3.834286 -3.042851 -0.145158
H 7.803067 -2.657522 -0.548324
H 5.180604 -5.061983 0.116477
H 7.608822 -4.878797 0.664116
H 1.182499 2.850238 0.271431
H 5.380085 5.899066 -1.727953
H 1.728587 -1.076964 1.243477
O 8.796776 -2.517424 1.284186
H 9.398061 -3.246353 1.094339

SCF Energy: -2179.75951008
Sum of electronic and zero-point Energies= -2179.300505
Sum of electronic and thermal Energies= -2179.263503
Sum of electronic and thermal Enthalpies= -2179.262559
Sum of electronic and thermal Free Energies= -2179.373532

==> ./AOX-II/bis/c3c4/RAF/c4p <==
O 5.942410 0.447547 -0.072850

O	2.824468	-1.392386	0.202560
O	2.460842	3.740949	0.000961
O	1.955997	1.163609	0.140225
O	7.025672	4.981505	-0.284714
C	4.139293	2.013427	-0.026486
C	5.514038	1.730070	-0.083637
C	5.085020	-0.595941	-0.013807
C	3.194877	0.939388	0.065506
C	3.736813	-0.398598	0.079359
C	5.778080	-1.891540	-0.021243
C	3.755051	3.383433	-0.052297
C	6.487120	2.710666	-0.165410
C	6.067032	4.035499	-0.194009
C	4.710490	4.376795	-0.135370
C	6.928339	-2.065059	0.758217
C	5.322738	-2.947384	-0.818857
C	7.598436	-3.280138	0.750475
C	6.002118	-4.159768	-0.825359
C	7.136865	-4.329977	-0.039517
H	7.536069	2.450048	-0.213957
H	4.403475	5.416240	-0.158625
H	7.288560	-1.249593	1.374950
H	4.456059	-2.815124	-1.457577
H	8.483925	-3.408283	1.363111
H	5.647655	-4.968301	-1.454831
H	7.664865	-5.277206	-0.045795
H	3.250226	-2.236871	0.389523
H	1.926586	2.922542	0.057378
H	6.632496	5.861574	-0.319164
Cu	0.344918	-0.016882	0.160398
O	-5.464209	-0.851772	-0.017636
O	-2.702888	1.422935	0.175769
O	-1.546832	-3.622501	0.057458
O	-1.425985	-0.960591	0.155428
O	-5.884078	-5.484847	-0.150346
C	-3.452184	-2.145945	0.027339
C	-4.859587	-2.053385	-0.026949
C	-4.789069	0.326091	0.045161
C	-2.675419	-0.962466	0.098875
C	-3.408611	0.315311	0.110882
C	-5.659003	1.471136	0.005773
C	-2.873879	-3.451986	0.011701
C	-5.678828	-3.162071	-0.088239
C	-5.073097	-4.416559	-0.096263
C	-3.680553	-4.567093	-0.049886
C	-7.062347	1.248720	-0.133225
C	-5.169460	2.805244	0.122430
C	-7.926894	2.290273	-0.200797
C	-6.027135	3.855032	0.063526
C	-7.447255	3.673191	-0.270836
H	-6.754715	-3.057346	-0.126422
H	-3.235240	-5.554974	-0.057856
H	-7.442025	0.238582	-0.159127
H	-4.123608	2.989903	0.299983
H	-8.994695	2.125421	-0.285941
H	-5.666432	4.871048	0.180144
H	-7.398227	3.795417	-1.387513
H	-1.115951	-2.748535	0.101321
H	-5.370142	-6.301879	-0.144856
H	-1.753675	1.210620	0.187141
O	-8.354746	4.589693	0.274196
H	-8.120963	5.474991	-0.024178

SCF Energy: -2179.76545646
Sum of electronic and zero-point Energies= -2179.305242
Sum of electronic and thermal Energies= -2179.268408
Sum of electronic and thermal Enthalpies= -2179.267464
Sum of electronic and thermal Free Energies= -2179.377623

==> ./AOX-II/bis/c3c4/RAF/c5 <==

O	5.521843	0.216882	-0.381449
O	2.379823	-1.380014	0.488661
O	2.363417	3.765729	0.263734
O	1.704013	1.231498	0.536055
O	6.879632	4.657630	-0.848444
C	3.876791	1.914770	-0.037406
C	5.193885	1.528301	-0.337340
C	4.615995	-0.758962	-0.147128
C	2.889436	0.915491	0.243561
C	3.325509	-0.459276	0.183779
C	5.198466	-2.103596	-0.250608
C	3.595767	3.309926	-0.015908
C	6.206214	2.432186	-0.607514
C	5.886604	3.784575	-0.578518
C	4.591813	4.227970	-0.284632
C	6.457911	-2.355390	0.307329
C	4.525725	-3.132599	-0.919427
C	7.022433	-3.619397	0.212215
C	5.099319	-4.394872	-1.014403
C	6.344343	-4.642088	-0.446263
H	7.207627	2.093374	-0.837513
H	4.364945	5.287999	-0.267088
H	6.986497	-1.561380	0.822252
H	3.569615	-2.941967	-1.395712
H	7.994618	-3.807194	0.654315
H	4.574710	-5.182589	-1.543425
H	6.789049	-5.628376	-0.520918
H	2.766726	-2.257870	0.583581
H	1.787857	2.992791	0.433231
H	6.547038	5.561949	-0.813915
Cu	0.064985	0.157582	0.936549
O	-5.208520	-0.105079	-0.522467

O	-2.770517	1.799492	1.240318
O	-1.784615	-3.197774	-0.039532
O	-1.689832	-0.700735	1.396761
O	-5.898641	-4.568425	-1.599499
C	-3.557180	-1.608972	0.281070
C	-4.733029	-1.349031	-0.380561
C	-4.583310	0.959944	-0.006397
C	-2.794025	-0.536140	0.812486
C	-3.383840	0.771422	0.676984
C	-5.297084	2.209650	-0.217566
C	-3.092654	-3.042320	0.422798
C	-5.497957	-2.352161	-0.989794
C	-5.092982	-3.700079	-0.969609
C	-3.928843	-4.030922	-0.336205
C	-6.685750	2.171755	-0.432619
C	-4.634899	3.447956	-0.234992
C	-7.392154	3.344195	-0.641708
C	-5.350775	4.614049	-0.459051
C	-6.727563	4.568390	-0.656235
H	-6.414482	-2.073765	-1.498866
H	-3.589228	-5.061345	-0.298307
H	-7.209288	1.224103	-0.417390
H	-3.564580	3.501158	-0.098388
H	-8.464758	3.304019	-0.792775
H	-4.828241	5.563388	-0.482374
H	-7.282534	5.484920	-0.823038
H	-1.237374	-2.515456	0.380194
H	-5.552692	-5.467466	-1.536966
H	-1.976779	1.479717	1.701383
O	-3.225015	-3.342217	1.790906
H	-2.994991	-4.270435	1.933399

SCF Energy: -2179.73509583
Sum of electronic and zero-point Energies= -2179.273941
Sum of electronic and thermal Energies= -2179.237265
Sum of electronic and thermal Enthalpies= -2179.236321
Sum of electronic and thermal Free Energies= -2179.346248

==> ./AOX-II/bis/c3c4/RAF/c8a <==

O	5.669582	0.343051	-0.140781
O	2.559351	-1.310157	0.743432
O	2.173265	3.472336	-1.119518
O	1.681572	1.068157	-0.162313
O	6.732918	4.595008	-1.740983
C	3.859860	1.833428	-0.596655
C	5.235945	1.553762	-0.557703
C	4.819604	-0.628536	0.260502
C	2.922878	0.842592	-0.159416
C	3.470938	-0.413332	0.294110
C	5.524564	-1.850865	0.669735
C	3.468908	3.123308	-1.053587
C	6.204927	2.465242	-0.938094
C	5.778634	3.713572	-1.376402
C	4.420598	4.047035	-1.438358
C	6.699254	-1.748774	1.425398
C	5.060140	-3.115827	0.290704
C	7.384589	-2.893183	1.807265
C	5.755006	-4.257145	0.672292
C	6.914251	-4.149321	1.433059
H	7.255610	2.210991	-0.894098
H	4.110192	5.026569	-1.784064
H	7.067078	-0.772283	1.718759
H	4.173428	-3.210449	-0.326617
H	8.288982	-2.804535	2.398888
H	5.392922	-5.231643	0.364275
H	7.454097	-5.041813	1.730035
H	2.979256	-2.032501	1.224506
H	1.640115	2.714977	-0.803302
H	6.331393	5.425233	-2.022175
Cu	0.130404	-0.047781	0.468632
O	-5.319010	-0.300167	-0.262306
O	-2.741003	1.414873	1.584168
O	-1.858048	-3.467825	0.148748
O	-1.547190	-0.948090	1.062567
O	-5.774016	-4.385204	-2.356478
C	-3.339013	-1.647335	-0.305084
C	-4.513558	-1.141252	-1.084556
C	-4.699902	0.669986	0.449329
C	-2.667552	-0.699120	0.520231
C	-3.367488	0.515018	0.811945
C	-5.583502	1.757227	0.825516
C	-2.951802	-2.967874	-0.417874
C	-5.393385	-2.185660	-1.681789
C	-4.999285	-3.490969	-1.723405
C	-3.781269	-3.868717	-1.116151
C	-6.975969	1.546484	0.781869
C	-5.099136	3.019222	1.217573
C	-7.849802	2.557054	1.141155
C	-5.984429	4.028803	1.558587
C	-7.357922	3.801662	1.529770
H	-6.294248	-1.847732	-2.181670
H	-3.466459	-4.906607	-1.169785
H	-7.364840	0.582044	0.482660
H	-4.038019	3.218231	1.232191
H	-8.918237	2.376039	1.118219
H	-5.599866	5.000384	1.846645
H	-8.044505	4.593994	1.806536
H	-1.390700	-2.742492	0.611085
H	-5.395348	-5.271900	-2.315987
H	-1.856463	1.067224	1.783579
O	-3.973477	-0.395057	-2.141845
H	-4.674177	-0.162366	-2.767334

SCF Energy: -2179.71806952
Sum of electronic and zero-point Energies= -2179.258048
Sum of electronic and thermal Energies= -2179.221364
Sum of electronic and thermal Enthalpies= -2179.220420
Sum of electronic and thermal Free Energies= -2179.330565

==> ./AOX-II/bis/c3c4/RAF/c2p <==

O	-5.910446	-0.089289	-0.109055
O	-2.565366	1.272857	0.252869
O	-2.978257	-3.861167	0.288515
O	-2.088443	-1.382688	0.300020
O	-7.667546	-4.411639	-0.091532
C	-4.370454	-1.903888	0.098427
C	-5.683146	-1.419668	-0.029243
C	-4.908021	0.816096	-0.056088
C	-3.277390	-0.980481	0.175960
C	-3.611756	0.422739	0.118487
C	-5.394382	2.197597	-0.168341
C	-4.199686	-3.315212	0.163786
C	-6.792171	-2.244796	-0.092761
C	-6.579265	-3.616669	-0.025454
C	-5.293660	-4.155790	0.102640
C	-6.559777	2.577978	0.508295
C	-4.726265	3.134418	-0.965586
C	-7.036162	3.876814	0.400815
C	-5.212543	4.431792	-1.072573
C	-6.363761	4.806677	-0.388093
H	-7.787139	-1.832358	-0.195100
H	-5.151411	-5.229367	0.154049
H	-7.084238	1.856510	1.124275
H	-3.844618	2.845237	-1.527363
H	-7.935001	4.164252	0.934978
H	-4.694140	5.147126	-1.701239
H	-6.739895	5.820346	-0.472497
H	-2.865812	2.174346	0.415638
H	-2.324842	-3.133097	0.320626
H	-7.407989	-5.338640	-0.039991
Cu	-0.326199	-0.442261	0.271514
O	5.478329	0.503739	-0.132851
O	2.787655	-1.863958	-0.151553
O	1.490002	3.147769	0.211380
O	1.441825	0.483243	0.086237
O	5.771503	5.145360	0.083079
C	3.434249	1.731916	0.041080
C	4.841088	1.688376	-0.036498
C	4.830748	-0.690352	-0.168824
C	2.690080	0.521732	0.015234
C	3.453690	-0.724865	-0.113599
C	5.726933	-1.812756	-0.237644
C	2.820873	3.016969	0.137980
C	5.630700	2.819154	-0.022185
C	4.990265	4.052922	0.072898
C	3.595792	4.157327	0.153033
C	7.196478	-1.579535	-0.027177
C	5.315154	-3.083223	-0.611337
C	8.054466	-2.774123	-0.022831
C	6.226720	-4.131429	-0.700724
C	7.598064	-3.990404	-0.412085
H	6.708404	2.746642	-0.084538
H	3.122625	5.129706	0.224756
H	7.483014	-1.149092	-1.022550
H	4.281184	-3.281769	-0.841910
H	9.089503	-2.611126	0.253770
H	5.852991	-5.103914	-1.000598
H	8.257194	-4.846485	-0.464024
H	1.082285	2.262070	0.186711
H	5.234721	5.945399	0.138768
H	1.834908	-1.689120	-0.080856
O	7.576829	-0.719189	1.009858
H	7.278164	0.172683	0.801923

SCF Energy: -2179.76276376
Sum of electronic and zero-point Energies= -2179.302066
Sum of electronic and thermal Energies= -2179.266259
Sum of electronic and thermal Enthalpies= -2179.265314
Sum of electronic and thermal Free Energies= -2179.372176

==> ./AOX-II/bis/c3c4/RAF/c6 <==

O	5.753519	-0.102380	0.300012
O	2.300269	-1.212815	0.425604
O	3.125402	3.561679	-1.328950
O	2.041963	1.280120	-0.577637
O	7.832935	3.891956	-0.919593
C	4.358553	1.666099	-0.496610
C	5.627346	1.160643	-0.166355
C	4.686832	-0.914629	0.472474
C	3.196571	0.848405	-0.309347
C	3.418865	-0.479848	0.212214
C	5.066460	-2.235029	0.993311
C	4.298052	2.998798	-0.992430
C	6.794138	1.891755	-0.302613
C	6.688329	3.188344	-0.791445
C	5.451226	3.744871	-1.137262
C	6.012802	-2.325725	2.021432
C	4.517646	-3.406637	0.459891
C	6.387132	-3.566814	2.516554
C	4.901044	-4.646351	0.957497

C	5.831661	-4.729127	1.987587
H	7.752232	1.463395	-0.039732
H	5.392606	4.758879	-1.516362
H	6.445280	-1.422336	2.436033
H	3.812034	-3.353778	-0.362571
H	7.114435	-3.627262	3.318617
H	4.477193	-5.548479	0.530734
H	6.128139	-5.697669	2.375089
H	2.497104	-2.017872	0.917942
H	2.417690	2.908446	-1.156884
H	7.649812	4.771303	-1.270504
Cu	0.244025	0.406026	-0.586677
O	-5.540056	0.111806	0.147324
O	-2.578967	2.162114	0.206514
O	-2.131084	-2.788411	-1.319782
O	-1.631539	-0.295023	-0.747127
O	-6.497490	-4.358885	-0.627866
C	-3.773708	-1.225089	-0.642905
C	-5.128754	-1.063730	-0.285867
C	-4.720082	1.200646	0.307551
C	-2.843410	-0.137832	-0.477154
C	-3.386966	1.107157	0.016052
C	-5.442733	2.351865	0.841952
C	-3.354275	-2.486844	-1.066323
C	-6.087994	-2.086993	-0.299573
C	-5.696556	-3.327670	-0.707024
C	-4.359232	-3.558377	-1.346359
C	-6.562338	2.139996	1.661104
C	-5.049985	3.665387	0.549095
C	-7.258676	3.217448	2.187360
C	-5.760983	4.737303	1.070740
C	-6.860883	4.518993	1.894320
H	-7.082112	-1.902931	0.083769
H	-4.569445	-3.393646	-2.422364
H	-6.877154	1.130513	1.895751
H	-4.205758	3.851256	-0.100347
H	-8.114662	3.039784	2.828665
H	-5.454361	5.748633	0.828348
H	-7.408542	5.360358	2.304563
H	-1.571767	-1.978100	-1.197519
H	-6.017091	-5.168407	-0.875407
H	-1.660270	1.908743	0.043281
O	-3.922910	-4.859467	-1.115964
H	-3.518899	-5.213218	-1.914954

SCF Energy: -2179.78983446

Sum of electronic and zero-point Energies= -2179.327502

Sum of electronic and thermal Energies= -2179.291076

Sum of electronic and thermal Enthalpies= -2179.290131

Sum of electronic and thermal Free Energies= -2179.399534

==> ./AOX-II/bis/c3c4/RAF/c5p <==

O	5.797521	0.406101	-0.025056
O	2.728499	-1.520457	-0.241594
O	2.241573	3.463328	0.973387
O	1.795684	0.938000	0.379088
O	6.774248	4.839246	1.052592
C	3.958317	1.851157	0.462293
C	5.338912	1.629050	0.325824
C	4.964564	-0.634310	-0.250436
C	3.039206	0.771666	0.250957
C	3.613732	-0.505472	-0.099452
C	5.681529	-1.861138	-0.621741
C	3.542790	3.164405	0.821128
C	6.288796	2.616060	0.521746
C	5.838534	3.884414	0.868638
C	4.474964	4.163714	1.019453
C	6.839679	-2.227654	0.074314
C	5.235239	-2.658527	-1.681590
C	7.525566	-3.382545	-0.274377
C	5.929682	-3.811497	-2.027693
C	7.071680	-4.177789	-1.323646
H	7.343023	2.403972	0.402377
H	4.145688	5.160586	1.290236
H	7.193146	-1.611643	0.893333
H	4.363284	-2.365705	-2.256676
H	8.416936	-3.663482	0.275556
H	5.581246	-4.417727	-2.856356
H	7.611125	-5.078857	-1.594293
H	3.179956	-2.366257	-0.344075
H	1.724368	2.650201	0.802627
H	6.358008	5.677918	1.283371
Cu	0.203499	-0.194076	0.005852
O	-5.453600	-0.672902	0.189053
O	-2.629294	1.238620	-0.929820
O	-1.760327	-3.719708	0.446179
O	-1.480017	-1.210318	-0.381103
O	-6.144313	-5.042965	1.655038
C	-3.552043	-2.116512	0.279847
C	-4.928555	-1.888221	0.458926
C	-4.701680	0.352978	-0.258869
C	-2.715244	-1.056839	-0.184132
C	-3.358856	0.213646	-0.455753
C	-5.506444	1.552341	-0.523277
C	-3.060014	-3.416817	0.590035
C	-5.802820	-2.856065	0.916226
C	-5.279565	-4.111057	1.208594
C	-3.917497	-4.396360	1.048648
C	-6.883997	1.422616	-0.798710
C	-4.973384	2.822050	-0.453093
C	-7.716911	2.516883	-1.037089

C	-5.776086	3.976801	-0.802291
C	-7.207759	3.787301	-0.978388
H	-6.855197	-2.640009	1.043435
H	-3.531013	-5.381953	1.281655
H	-7.316174	0.430199	-0.830766
H	-3.936787	3.001587	-0.202199
H	-8.768028	2.356483	-1.236650
H	-7.820310	4.664735	-1.152711
H	-1.294706	-2.929948	0.108852
H	-5.689194	-5.875496	1.827894
H	-1.723089	0.939226	-1.090498
H	-5.550212	3.863194	-1.922660
O	-5.288639	5.176019	-0.319050
H	-5.758236	5.905785	-0.738452

SCF Energy: -2179.75990004
Sum of electronic and zero-point Energies= -2179.300464
Sum of electronic and thermal Energies= -2179.263616
Sum of electronic and thermal Enthalpies= -2179.262672
Sum of electronic and thermal Free Energies= -2179.373183

==> ./AOX-II/bis/c3c4/RAF/c6p <==

O	-5.669575	-0.238686	-0.214973
O	-2.474875	1.427581	0.230371
O	-2.398807	-3.722092	0.166955
O	-1.753050	-1.172793	0.275483
O	-7.006262	-4.708690	-0.330017
C	-3.967549	-1.902691	-0.013348
C	-5.317874	-1.543520	-0.159919
C	-4.757718	0.755663	-0.127236
C	-2.969955	-0.881942	0.117954
C	-3.433349	0.484358	0.067169
C	-5.367972	2.088323	-0.224016
C	-3.664020	-3.292472	0.026188
C	-6.341019	-2.469312	-0.266013
C	-5.999072	-3.816140	-0.224060
C	-4.670388	-4.232136	-0.078776
C	-6.567405	2.351082	0.449068
C	-4.782101	3.093844	-1.002192
C	-7.157966	3.603434	0.357677
C	-5.382587	4.343819	-1.093100
C	-6.566991	4.602553	-0.411545
H	-7.368628	-2.151118	-0.381393
H	-4.424599	-5.287642	-0.047266
H	-7.028889	1.575447	1.049374
H	-3.874011	2.894318	-1.561284
H	-8.082499	3.800587	0.888943
H	-4.926716	5.112718	-1.706771
H	-7.032502	5.579415	-0.483314
H	-2.863369	2.297788	0.375946
H	-1.821062	-2.935136	0.235459
H	-6.664480	-5.609928	-0.297203
Cu	-0.133265	-0.070347	0.652897
O	5.376772	0.596967	-0.328794
O	2.848375	-1.592699	0.975017
O	1.714667	3.474066	0.831662
O	1.599730	0.831316	1.129067
O	5.776986	5.210790	-0.861363
C	3.496241	1.949502	0.278518
C	4.811917	1.819645	-0.211274
C	4.709853	-0.532082	0.007911
C	2.766002	0.789798	0.659576
C	3.446909	-0.491513	0.522617
C	5.541763	-1.725259	-0.106079
C	2.957426	3.266327	0.373396
C	5.580337	2.900283	-0.597593
C	5.014692	4.167317	-0.487254
C	3.711973	4.355809	-0.007947
C	6.811844	-1.729352	0.440270
C	5.139689	-2.882295	-0.924574
C	7.597001	-2.881096	0.409816
C	5.939490	-4.098109	-0.803832
C	7.168101	-4.064463	-0.205634
H	6.587197	2.763460	-0.968747
H	3.295267	5.353592	0.067650
H	7.169387	-0.850450	0.960726
H	8.563773	-2.861120	0.898962
H	5.564833	-4.988389	-1.296798
H	7.788744	-4.950310	-0.177637
H	1.320934	2.611362	1.065794
H	5.300209	6.040137	-0.736046
H	1.960399	-1.364955	1.292293
H	5.708470	-2.495159	-1.835694
O	3.799689	-2.990778	-1.234580
H	3.699401	-3.590659	-1.982247

SCF Energy: -2179.75694892
Sum of electronic and zero-point Energies= -2179.297254
Sum of electronic and thermal Energies= -2179.260550
Sum of electronic and thermal Enthalpies= -2179.259605
Sum of electronic and thermal Free Energies= -2179.369196

==> ./AOX-II/bis/c3c4/RAF/c4 <==

O	5.370013	0.512613	-0.323535
O	1.799671	0.658531	-0.962677
O	3.393479	-2.878068	2.430241
O	1.956793	-1.350151	0.833379
O	8.077598	-2.174389	2.362535

C	4.291923	-1.148661	1.012464
C	5.455835	-0.495992	0.572926
C	4.186292	0.931418	-0.823810
C	3.017483	-0.762315	0.483655
C	3.015272	0.324576	-0.467932
C	4.343082	2.029844	-1.787376
C	4.453329	-2.200971	1.956955
C	6.724328	-0.827322	1.015482
C	6.835326	-1.860311	1.938713
C	5.710755	-2.547831	2.410402
C	5.369422	1.969887	-2.738184
C	3.495367	3.143372	-1.761331
C	5.529663	2.997331	-3.656898
C	3.665073	4.170743	-2.681970
C	4.677271	4.098256	-3.632884
H	7.595350	-0.297787	0.652869
H	5.819639	-3.351276	3.130044
H	6.032156	1.112374	-2.761382
H	2.721127	3.223785	-1.005587
H	6.321623	2.937724	-4.395249
H	3.008848	5.033225	-2.648099
H	4.805590	4.900145	-4.351702
H	1.876668	1.251238	-1.719177
H	2.590115	-2.520124	2.001545
H	8.043139	-2.897126	3.000098
Cu	0.146936	-1.361161	-0.025284
O	-5.181950	0.333327	0.331942
O	-2.420056	-0.552552	1.748007
O	-3.093817	-2.940774	-2.520385
O	-1.700930	-1.360746	-0.874816
O	-7.780470	-2.399844	-2.389442
C	-4.030252	-1.291955	-1.044701
C	-5.220086	-0.699689	-0.593969
C	-4.127679	0.845826	0.862751
C	-2.783592	-0.884494	-0.507753
C	-2.761816	0.184355	0.608682
C	-4.358279	1.966989	1.717181
C	-4.157566	-2.318988	-2.019858
C	-6.465554	-1.048418	-1.020789
C	-6.550417	-2.075898	-1.982680
C	-5.413030	-2.703896	-2.474535
C	-5.692813	2.403977	1.907913
C	-3.311045	2.652907	2.377449
C	-5.962436	3.478998	2.725875
C	-3.598157	3.727748	3.194783
C	-4.916902	4.141972	3.370558
H	-7.351874	-0.554670	-0.646623
H	-5.494152	-3.491063	-3.214750
H	-6.504261	1.890804	1.410279
H	-2.281136	2.354963	2.248669
H	-6.984923	3.806556	2.867159
H	-2.792224	4.247923	3.697174
H	-5.132998	4.987282	4.013981
H	-2.216995	0.029885	2.491217
H	-2.283899	-2.574356	-2.118001
H	-7.749028	-3.098219	-3.056056
O	-1.890395	1.206848	0.287531
H	-1.048180	0.835269	-0.012138

SCF Energy: -2179.78366236
Sum of electronic and zero-point Energies= -2179.321603
Sum of electronic and thermal Energies= -2179.285030
Sum of electronic and thermal Enthalpies= -2179.284085
Sum of electronic and thermal Free Energies= -2179.393333

==> ./AOX-II/bis/c3c4/RAF/c3 <==

O	-4.949407	0.743514	-0.669459
O	-1.341477	0.392558	-0.805573
O	-3.906629	-3.091947	2.010401
O	-2.054019	-1.724859	0.738586
O	-8.364010	-1.562753	1.516518
C	-4.329448	-1.154129	0.641496
C	-5.306741	-0.277264	0.142404
C	-3.663935	0.966512	-1.019591
C	-2.951631	-0.953964	0.300814
C	-2.654303	0.177858	-0.545818
C	-3.516811	2.136844	-1.895516
C	-4.774257	-2.211584	1.484249
C	-6.654875	-0.401841	0.426952
C	-7.045498	-1.450652	1.251293
C	-6.115432	-2.352276	1.782412
C	-4.238001	3.303692	-1.613945
C	-2.687382	2.097189	-3.022203
C	-4.116542	4.414013	-2.437083
C	-2.574372	3.211810	-3.844669
C	-3.283795	4.371718	-3.552430
H	-7.375847	0.292934	0.017058
H	-6.440643	-3.162579	2.425079
H	-4.885857	3.339425	-0.745588
H	-2.153606	1.187575	-3.276466
H	-4.673069	5.315772	-2.206960
H	-1.937386	3.167757	-4.720966
H	-3.193103	5.240289	-4.195440
H	-1.209600	1.245381	-1.236997
H	-3.009796	-2.850195	1.702422
H	-8.531171	-2.315168	2.096017
Cu	-0.151385	-1.996392	0.206588
O	4.819797	0.584390	0.442105
O	2.126590	-0.476685	2.055765
O	3.442351	-3.371139	-1.945738
O	1.787089	-1.994453	-0.371660
O	7.842005	-1.695870	-2.259135

C	4.015887	-1.387131	-0.713469
C	5.049345	-0.485704	-0.412906
C	3.705806	0.869867	1.015480
C	2.726460	-1.216004	-0.146268
C	2.472098	-0.014655	0.787731
C	3.701199	2.029622	1.837796
C	4.340543	-2.455146	-1.595120
C	6.315860	-0.567678	-0.907405
C	6.595860	-1.636868	-1.782813
C	5.622092	-2.568145	-2.120650
C	4.891434	2.789168	1.956239
C	2.540643	2.446088	2.531593
C	4.913042	3.921412	2.739626
C	2.580024	3.582481	3.313926
C	3.758886	4.318834	3.418405
H	7.075152	0.155211	-0.642739
H	5.852763	-3.386248	-2.792690
H	5.782882	2.479196	1.427644
H	1.623319	1.883137	2.456747
H	5.823272	4.501431	2.827274
H	1.691463	3.899728	3.845321
H	3.780251	5.211018	4.033945
H	2.591685	-3.177843	-1.508662
H	7.949282	-2.448919	-2.854530
H	1.457144	-1.171796	1.979704
O	1.537562	0.837937	0.205394
H	0.757284	0.339324	-0.082023

SCF Energy: -2179.78653314
Sum of electronic and zero-point Energies= -2179.323462
Sum of electronic and thermal Energies= -2179.287403
Sum of electronic and thermal Enthalpies= -2179.286459
Sum of electronic and thermal Free Energies= -2179.393875

==> ./AOX-II/bis/c3c4/RAF/c1p <==

O	-5.693808	-0.125914	-0.010801
O	-2.475386	1.513075	0.365667
O	-2.396227	-3.334059	-1.358919
O	-1.750531	-0.921468	-0.523672
O	-7.017297	-4.375518	-1.416280
C	-3.978484	-1.655202	-0.664474
C	-5.334679	-1.350025	-0.460296
C	-4.784485	0.842351	0.242754
C	-2.978079	-0.670475	-0.378749
C	-3.447616	0.612370	0.087957
C	-5.402955	2.082983	0.729006
C	-3.668037	-2.959373	-1.141796
C	-6.358186	-2.248876	-0.704653
C	-6.010237	-3.509705	-1.175799
C	-4.675148	-3.869724	-1.393905
C	-6.427587	2.014549	1.680868
C	-4.997325	3.332131	0.245081
C	-7.019787	3.177492	2.152322
C	-5.598050	4.492385	0.719327
C	-6.605168	4.418419	1.675383
H	-7.391247	-1.973651	-0.538425
H	-4.424721	-4.859081	-1.759750
H	-6.749616	1.049874	2.056019
H	-4.238045	3.400513	-0.527189
H	-7.807052	3.115025	2.895455
H	-5.283663	5.454570	0.330850
H	-7.071083	5.325321	2.044889
H	-2.836149	2.299472	0.790118
H	-1.816293	-2.582493	-1.121582
H	-6.669785	-5.211132	-1.749279
Cu	-0.110942	0.145233	-0.093317
O	5.520136	0.097212	-0.391150
O	2.638445	-1.496847	1.000976
O	2.135209	3.497976	-0.447986
O	1.644207	1.011560	0.370577
O	6.573842	4.410957	-1.811280
C	3.767871	1.727081	-0.367893
C	5.107148	1.367843	-0.614182
C	4.692544	-0.834250	0.099140
C	2.853942	0.757274	0.141545
C	3.401088	-0.561338	0.423561
C	5.411076	-2.173182	0.276992
C	3.391724	3.072843	-0.646931
C	6.050803	2.250929	-1.099204
C	5.641474	3.558001	-1.348119
C	4.321959	3.971183	-1.128676
C	6.208088	-1.950661	1.526135
C	4.448686	-3.320480	0.360539
C	6.001414	-2.685236	2.649654
C	4.340974	-4.092966	1.470909
C	5.094775	-3.763014	2.611908
H	7.070554	1.935787	-1.275016
H	4.024991	4.993310	-1.333853
H	6.943132	-1.154338	1.492596
H	3.902623	-3.546461	-0.547897
H	6.565757	-2.489879	3.551775
H	3.686222	-4.953747	1.487985
H	4.990822	-4.380507	3.497525
H	1.602635	2.751592	-0.111831
H	6.196215	5.289607	-1.937724
H	1.709637	-1.219760	0.985675
O	6.231846	-2.349187	-0.857482
H	6.842462	-3.078058	-0.691782

SCF Energy: -2179.73990363
Sum of electronic and zero-point Energies= -2179.278767

Sum of electronic and thermal Energies= -2179.242164
Sum of electronic and thermal Enthalpies= -2179.241220
Sum of electronic and thermal Free Energies= -2179.350397

==> ./AOX-II/bis/c3c4/RAF/c7 <==

O	5.635714	0.201585	-0.324435
O	2.538916	-1.335142	0.779012
O	2.348507	3.692595	-0.356087
O	1.761309	1.214399	0.323830
O	6.865285	4.548351	-1.493684
C	3.926589	1.870851	-0.309904
C	5.263845	1.488680	-0.509646
C	4.759024	-0.750572	0.064342
C	2.965269	0.897976	0.119271
C	3.452079	-0.446088	0.318300
C	5.390352	-2.068879	0.213592
C	3.601200	3.238911	-0.530365
C	6.253751	2.370618	-0.905852
C	5.891226	3.696909	-1.109915
C	4.574494	4.134871	-0.926579
C	6.642984	-2.167170	0.831753
C	4.772564	-3.225865	-0.275227
C	7.255002	-3.404368	0.973822
C	5.394279	-4.460705	-0.134026
C	6.632169	-4.553394	0.493150
H	7.272042	2.034849	-1.050374
H	4.313614	5.174466	-1.089937
H	7.128533	-1.274214	1.208469
H	3.819950	-3.160644	-0.789849
H	8.220930	-3.472182	1.461858
H	4.912421	-5.349978	-0.524638
H	7.113998	-5.518654	0.603868
H	2.965025	-2.145888	1.080501
H	1.792634	2.941354	-0.067292
H	6.508964	5.437567	-1.604223
Cu	0.130217	0.125270	0.748307
O	-5.264985	-0.006866	-0.313659
O	-2.701506	1.686286	1.468891
O	-1.774933	-3.266413	0.436568
O	-1.591183	-0.764172	1.255733
O	-6.086681	-4.370785	-0.482417
C	-3.485779	-1.587268	0.098499
C	-4.775785	-1.271550	-0.398236
C	-4.601135	0.987979	0.258204
C	-2.726422	-0.558442	0.733038
C	-3.337079	0.731772	0.818982
C	-5.317802	2.244783	0.260799
C	-2.992271	-2.939787	-0.028448
C	-5.578269	-2.185920	-0.986913
C	-5.125826	-3.602204	-1.161096
C	-3.759020	-3.880296	-0.626558
C	-6.719827	2.225221	0.126893
C	-4.652969	3.480884	0.357973
C	-7.434047	3.408982	0.114060
C	-5.378876	4.659699	0.324588
C	-6.766185	4.628677	0.211053
H	-6.561277	-1.918445	-1.355191
H	-3.392577	-4.896554	-0.731795
H	-7.242097	1.279729	0.056806
H	-3.576165	3.522244	0.429177
H	-8.514032	3.383723	0.028845
H	-4.858636	5.608332	0.384594
H	-7.328749	5.555393	0.196255
H	-1.375781	-2.479731	0.857523
H	-5.720020	-5.238283	-0.270199
H	-1.869110	1.332234	1.828305
O	-5.172819	-3.811641	-2.550417
H	-4.588153	-4.539552	-2.793611

SCF Energy: -2179.72661435
Sum of electronic and zero-point Energies= -2179.265577
Sum of electronic and thermal Energies= -2179.228995
Sum of electronic and thermal Enthalpies= -2179.228051
Sum of electronic and thermal Free Energies= -2179.337506

==> ./AOX-II/bis/c3c4/RAF/c4a <==

O	5.771271	0.053453	-0.133346
O	2.381089	-1.101471	-0.734309
O	2.887061	3.630325	1.229928
O	1.954662	1.357399	0.278947
O	7.594581	3.958658	1.653825
C	4.253644	1.778014	0.519540
C	5.561989	1.284701	0.384391
C	4.754074	-0.740464	-0.536994
C	3.142498	0.957174	0.139513
C	3.453950	-0.343835	-0.403215
C	5.226376	-2.027635	-1.064613
C	4.103261	3.083052	1.067438
C	6.685202	2.002230	0.756358
C	6.491862	3.271424	1.288994
C	5.211484	3.816601	1.442358
C	6.253885	-2.709511	-0.400910
C	4.686754	-2.571546	-2.235621
C	6.717404	-3.921407	-0.892469
C	5.159020	-3.783858	-2.724438
C	6.170652	-4.462255	-2.053485
H	7.676272	1.584950	0.637273
H	5.084068	4.809955	1.857265
H	6.680956	-2.292013	0.503648

H	3.918791	-2.036319	-2.783683
H	7.508397	-4.445605	-0.367751
H	4.740743	-4.191607	-3.637873
H	6.536981	-5.408342	-2.436599
H	2.642290	-2.005310	-0.944561
H	2.222009	2.977123	0.932126
H	7.351814	4.816053	2.022630
Cu	0.192655	0.413053	0.246359
O	-5.623513	-0.323887	-0.037599
O	-2.821393	1.900652	-0.489227
O	-1.791869	-3.067759	0.996184
O	-1.599717	-0.502352	0.248462
O	-5.997426	-3.911068	2.848706
C	-3.656218	-1.717576	0.199182
C	-5.071731	-1.386841	0.566144
C	-4.884764	0.756668	-0.421434
C	-2.827782	-0.423620	0.122440
C	-3.520804	0.765089	-0.259478
C	-5.723076	1.834250	-0.929037
C	-3.061869	-2.787450	1.112417
C	-5.816356	-2.125538	1.399487
C	-5.203661	-3.233627	2.069317
C	-3.844263	-3.542480	1.938151
C	-7.066412	1.911269	-0.525239
C	-5.227283	2.782734	-1.837402
C	-7.883464	2.923393	-1.003345
C	-6.057892	3.781288	-2.322744
C	-7.382930	3.859988	-1.903644
H	-6.849563	-1.880600	1.605155
H	-3.409942	-4.357399	2.503455
H	-7.462411	1.186306	0.174458
H	-4.203403	2.727395	-2.178929
H	-8.914520	2.980514	-0.673463
H	-5.668003	4.500643	-3.033855
H	-8.025434	4.648404	-2.280000
H	-1.313995	-2.345213	0.542927
H	-5.539443	-4.638751	3.298661
H	-1.874906	1.725879	-0.408192
O	-3.720291	-2.259250	-1.108923
H	-2.824667	-2.393586	-1.448262

SCF Energy: -2179.75342104
Sum of electronic and zero-point Energies= -2179.291372
Sum of electronic and thermal Energies= -2179.254868
Sum of electronic and thermal Enthalpies= -2179.253924
Sum of electronic and thermal Free Energies= -2179.362846

==> ./AOX-II/bis/c3c4/vEA <==

O	5.201775	0.191439	-0.155713
O	1.781027	1.339482	0.225233
O	2.510672	-3.747805	0.411932
O	1.471925	-1.277518	0.383136
O	7.185688	-4.028813	-0.250654
C	3.779407	-1.713770	0.139656
C	5.053496	-1.153302	-0.071293
C	4.170926	1.047537	-0.044504
C	2.660644	-0.841045	0.230479
C	2.904835	0.562931	0.126145
C	4.573334	2.450192	-0.174305
C	3.687089	-3.131248	0.216416
C	6.198531	-1.913674	-0.200246
C	6.064500	-3.296539	-0.118110
C	4.821767	-3.906655	0.090306
C	5.627699	2.785130	-1.034037
C	3.940696	3.456841	0.565404
C	6.027764	4.106973	-1.162704
C	4.355585	4.775845	0.438789
C	5.393392	5.104697	-0.427496
H	7.161603	-1.448253	-0.361582
H	4.744238	-4.986151	0.150743
H	6.122837	2.010542	-1.607433
H	3.145436	3.211227	1.258217
H	6.837632	4.358786	-1.838084
H	3.868898	5.547629	1.024308
H	5.710684	6.136914	-0.526985
H	1.766930	2.053326	-0.426589
H	1.804684	-3.079725	0.475734
H	6.981076	-4.968709	-0.181764
Cu	0.000388	0.001595	0.393676
O	-5.201754	-0.192247	-0.155863
O	-1.780119	-1.338293	0.223607
O	-2.512427	3.748460	0.409578
O	-1.472418	1.278968	0.379813
O	-7.188194	4.026866	-0.248553
C	-3.780237	1.713690	0.138394
C	-5.054200	1.152540	-0.071505
C	-4.170315	-1.047751	-0.045122
C	-2.660942	0.841596	0.228502
C	-2.904382	-0.562512	0.124882
C	-4.572089	-2.450637	-0.174590
C	-3.688666	3.131244	0.215165
C	-6.199778	1.912280	-0.199391
C	-6.066477	3.295203	-0.117159
C	-4.823913	3.906001	0.090265
C	-5.626199	-2.786253	-1.034370
C	-3.939172	-3.456782	0.565545
C	-6.025756	-4.108288	-1.162630
C	-4.353529	-4.775999	0.439307
C	-5.391104	-5.105525	-0.426996
H	-7.162727	1.446343	-0.359966
H	-4.746961	4.985530	0.150841
H	-6.121547	-2.012025	-1.608075

H	-3.144093	-3.210608	1.258383
H	-6.835457	-4.360642	-1.838009
H	-3.866620	-5.547403	1.025144
H	-5.707996	-6.137894	-0.526184
H	-1.766671	-2.053379	-0.426847
H	-1.806013	3.080725	0.472435
H	-6.984022	4.966871	-0.179810

SCF Energy: -2104.15134676

==> ./AOX-II/bis/c3c4/vIP <==

O	5.201775	0.191439	-0.155713
O	1.781027	1.339482	0.225233
O	2.510672	-3.747805	0.411932
O	1.471925	-1.277518	0.383136
O	7.185688	-4.028813	-0.250654
C	3.779407	-1.713770	0.139656
C	5.053496	-1.153302	-0.071293
C	4.170926	1.047537	-0.044504
C	2.660644	-0.841045	0.230479
C	2.904835	0.562931	0.126145
C	4.573334	2.450192	-0.174305
C	3.687089	-3.131248	0.216416
C	6.198531	-1.913674	-0.200246
C	6.064500	-3.296539	-0.118110
C	4.821767	-3.906655	0.090306
C	5.627699	2.785130	-1.034037
C	3.940696	3.456841	0.565404
C	6.027764	4.106973	-1.162704
C	4.355585	4.775845	0.438789
C	5.393392	5.104697	-0.427496
H	7.161603	-1.448253	-0.361582
H	4.744238	-4.986151	0.150743
H	6.122837	2.010542	-1.607433
H	3.145436	3.211227	1.258217
H	6.837632	4.358786	-1.838084
H	3.868898	5.547629	1.024308
H	5.710684	6.136914	-0.526985
H	1.766930	2.053326	-0.426589
H	1.804684	-3.079725	0.475734
H	6.981076	-4.968709	-0.181764
Cu	0.000388	0.001595	0.393676
O	-5.201754	-0.192247	-0.155863
O	-1.780119	-1.338293	0.223607
O	-2.512427	3.748460	0.409578
O	-1.472418	1.278968	0.379813
O	-7.188194	4.026866	-0.248553
C	-3.780237	1.713690	0.138394
C	-5.054200	1.152540	-0.071505
C	-4.170315	-1.047751	-0.045122
C	-2.660942	0.841596	0.228502
C	-2.904382	-0.562512	0.124882
C	-4.572089	-2.450637	-0.174590
C	-3.688666	3.131244	0.215165
C	-6.199778	1.912280	-0.199391
C	-6.066477	3.295203	-0.117159
C	-4.823913	3.906001	0.090265
C	-5.626199	-2.786253	-1.034370
C	-3.939172	-3.456782	0.565545
C	-6.025756	-4.108288	-1.162630
C	-4.353529	-4.775999	0.439307
C	-5.391104	-5.105525	-0.426996
H	-7.162727	1.446343	-0.359966
H	-4.746961	4.985530	0.150841
H	-6.121547	-2.012025	-1.608075
H	-3.144093	-3.210608	1.258383
H	-6.835457	-4.360642	-1.838009
H	-3.866620	-5.547403	1.025144
H	-5.707996	-6.137894	-0.526184
H	-1.766671	-2.053379	-0.426847
H	-1.806013	3.080725	0.472435
H	-6.984022	4.966871	-0.179810

SCF Energy: -2103.75258017

==> ./AOX-II/bis/c3c4 <==

O	5.201775	0.191439	-0.155713
O	1.781027	1.339482	0.225233
O	2.510672	-3.747805	0.411932
O	1.471925	-1.277518	0.383136
O	7.185688	-4.028813	-0.250654
C	3.779407	-1.713770	0.139656
C	5.053496	-1.153302	-0.071293
C	4.170926	1.047537	-0.044504
C	2.660644	-0.841045	0.230479
C	2.904835	0.562931	0.126145
C	4.573334	2.450192	-0.174305
C	3.687089	-3.131248	0.216416
C	6.198531	-1.913674	-0.200246
C	6.064500	-3.296539	-0.118110
C	4.821767	-3.906655	0.090306
C	5.627699	2.785130	-1.034037
C	3.940696	3.456841	0.565404
C	6.027764	4.106973	-1.162704
C	4.355585	4.775845	0.438789
C	5.393392	5.104697	-0.427496
H	7.161603	-1.448253	-0.361582
H	4.744238	-4.986151	0.150743
H	6.122837	2.010542	-1.607433
H	3.145436	3.211227	1.258217
H	6.837632	4.358786	-1.838084
H	3.868898	5.547629	1.024308
H	5.710684	6.136914	-0.526985
H	1.766930	2.053326	-0.426589

H	1.804684	-3.079725	0.475734
H	6.981076	-4.968709	-0.181764
Cu	0.000388	0.001595	0.393676
O	-5.201754	-0.192247	-0.155863
O	-1.780119	-1.338293	0.223607
O	-2.512427	3.748460	0.409578
O	-1.472418	1.278968	0.379813
O	-7.188194	4.026866	-0.248553
C	-3.780237	1.713690	0.138394
C	-5.054200	1.152540	-0.071505
C	-4.170315	-1.047751	-0.045122
C	-2.660942	0.841596	0.228502
C	-2.904382	-0.562512	0.124882
C	-4.572089	-2.450637	-0.174590
C	-3.688666	3.131244	0.215165
C	-6.199778	1.912280	-0.199391
C	-6.066477	3.295203	-0.117159
C	-4.823913	3.906001	0.090265
C	-5.626199	-2.786253	-1.034370
C	-3.939172	-3.456782	0.565545
C	-6.025756	-4.108288	-1.162630
C	-4.353529	-4.775999	0.439307
C	-5.391104	-5.105525	-0.426996
H	-7.162727	1.446343	-0.359966
H	-4.746961	4.985530	0.150841
H	-6.121547	-2.012025	-1.608075
H	-3.144093	-3.210608	1.258383
H	-6.835457	-4.360642	-1.838009
H	-3.866620	-5.547403	1.025144
H	-5.707996	-6.137894	-0.526184
H	-1.766671	-2.053379	-0.426847
H	-1.806013	3.080725	0.472435
H	-6.984022	4.966871	-0.179810

SCF Energy: -2103.98637842
Sum of electronic and zero-point Energies= -2103.540246
Sum of electronic and thermal Energies= -2103.505507
Sum of electronic and thermal Enthalpies= -2103.504563
Sum of electronic and thermal Free Energies= -2103.610129

==> ./AOX-II/PA/vIP <==

O	-0.199130	-0.882597	0.078446
O	-1.270594	2.563037	-0.147414
O	3.798202	1.764772	-0.100298
O	1.352100	2.850034	-0.165672
O	4.023230	-2.944396	0.178034
C	1.749736	0.510722	-0.014629
C	1.157215	-0.769365	0.061871
C	-1.030345	0.194985	0.020735
C	0.929786	1.664615	-0.079787
C	-0.506639	1.447853	-0.057093
C	-2.446831	-0.186480	0.016292
C	3.175739	0.561119	-0.026007
C	1.888500	-1.932778	0.125896
C	3.315244	-1.884261	0.117098
C	3.924797	-0.585253	0.037267
C	-2.825272	-1.441831	-0.481490
C	-3.440519	0.669162	0.510995
C	-4.159976	-1.822797	-0.496127
C	-4.773634	0.278486	0.499727
C	-5.140387	-0.964543	-0.006532
H	1.381639	-2.889254	0.185068
H	5.007713	-0.515842	0.027429
H	-2.071183	-2.117574	-0.866514
H	-3.172787	1.633471	0.921287
H	-4.434686	-2.794252	-0.892813
H	-5.528618	0.949641	0.894777
H	-6.182757	-1.264306	-0.016451
H	-0.639059	3.298860	-0.223303
H	3.104798	2.454650	-0.143540

SCF Energy: -952.749089115

==> ./AOX-II/PA/aIP <==

O	-0.206853	-0.879589	0.084858
O	-1.266197	2.535662	-0.161569
O	3.788257	1.750758	-0.097967
O	1.366094	2.839088	-0.166803
O	3.983299	-2.935820	0.174980
C	1.745957	0.495396	-0.012104
C	1.136485	-0.756239	0.065501
C	-1.032526	0.180913	0.023263
C	0.929403	1.668060	-0.080786
C	-0.497132	1.448580	-0.063150
C	-2.445942	-0.186878	0.017614
C	3.197853	0.553806	-0.024798
C	1.871604	-1.930155	0.129885
C	3.331934	-1.873870	0.117263
C	3.950500	-0.581141	0.036880
C	-2.826867	-1.439462	-0.487562
C	-3.430660	0.676034	0.519364
C	-4.162566	-1.810513	-0.504121
C	-4.763952	0.290389	0.509853
C	-5.135099	-0.947694	-0.005472
H	1.376987	-2.891318	0.187435
H	5.031677	-0.518163	0.026515
H	-2.076008	-2.114041	-0.880545
H	-3.156295	1.635572	0.935546
H	-4.445575	-2.775761	-0.908826
H	-5.515639	0.960940	0.910946
H	-6.179092	-1.241039	-0.016883

H	-0.660840	3.295477	-0.229753
H	3.094040	2.444186	-0.142193

SCF Energy: -952.753306124
Sum of electronic and zero-point Energies= -952.545466
Sum of electronic and thermal Energies= -952.530026
Sum of electronic and thermal Enthalpies= -952.529081
Sum of electronic and thermal Free Energies= -952.589380

==> ./AOX-II/PA/mono-/c3c4 <==

O	1.632851	1.438462	-0.004955
O	0.075596	-1.844461	-0.004470
O	-3.124912	2.177171	-0.060993
O	-2.044392	-0.262161	0.003750
O	-0.115195	5.796670	-0.079766
C	-0.753826	1.710468	-0.032911
C	0.549234	2.269938	-0.021647
C	1.531111	0.094466	-0.018702
C	-0.895250	0.311483	-0.011612
C	0.298368	-0.486423	-0.000510
C	2.838364	-0.572239	-0.014228
C	-1.848972	2.632365	-0.053456
C	0.793080	3.617600	-0.034626
C	-0.300088	4.540316	-0.061338
C	-1.625740	3.982411	-0.068015
C	3.921085	0.037861	0.632495
C	3.033771	-1.791844	-0.674225
C	5.168819	-0.569154	0.630460
C	4.287918	-2.388219	-0.679373
C	5.356125	-1.782885	-0.024935
H	1.812103	3.985944	-0.030364
H	-2.473592	4.658961	-0.086550
H	3.781243	0.983307	1.142708
H	2.215395	-2.263690	-1.203634
H	5.997721	-0.091497	1.140938
H	4.431169	-3.326710	-1.203306
H	6.333332	-2.253306	-0.028825
H	0.592535	-2.304513	0.670090
H	-3.108970	1.203392	-0.043740
O	-2.052242	-4.215965	0.250007
H	-2.789111	-4.626497	-0.218419
O	-4.265142	-2.168388	0.019324
H	-4.588828	-2.840063	0.629989
H	-1.256045	-4.566268	-0.167419
H	-4.544483	-1.328892	0.402085
Cu	-2.116517	-2.215091	0.021000

SCF Energy: -1303.00041962
Sum of electronic and zero-point Energies= -1302.740248
Sum of electronic and thermal Energies= -1302.717402
Sum of electronic and thermal Enthalpies= -1302.716458
Sum of electronic and thermal Free Energies= -1302.792522

==> ./AOX-II/PA/mono-/c3c4/aEA <==

O	1.854762	1.320623	-0.071515
O	0.342795	-1.974074	0.171643
O	-2.896241	2.025413	0.088128
O	-1.829706	-0.381492	0.160977
O	0.081740	5.669426	-0.124849
C	-0.527154	1.569137	0.023019
C	0.766131	2.139153	-0.042706
C	1.746074	-0.029145	-0.033096
C	-0.683774	0.160961	0.083679
C	0.528994	-0.632679	0.065812
C	3.055698	-0.698137	-0.057607
C	-1.624214	2.489012	0.034578
C	1.000244	3.492214	-0.090415
C	-0.094298	4.408136	-0.077418
C	-1.412842	3.842054	-0.012658
C	4.108894	-0.180499	0.706917
C	3.278984	-1.832705	-0.846947
C	5.352287	-0.796764	0.693919
C	4.527666	-2.443673	-0.857273
C	5.564703	-1.931076	-0.085495
H	2.017070	3.863778	-0.143973
H	-2.267178	4.511016	-0.004351
H	3.947130	0.700869	1.316943
H	2.488824	-2.223347	-1.479546
H	6.158198	-0.390833	1.295461
H	4.690822	-3.316386	-1.479869
H	6.537831	-2.409781	-0.094986
H	1.180648	-2.429602	0.307316
H	-2.853826	1.049045	0.123143
O	-3.322548	-4.080253	0.193764
H	-4.276442	-3.997287	0.091401
O	-5.237404	-0.348324	-0.535244
H	-5.138294	0.559237	-0.234584
H	-3.039226	-4.611243	-0.558037
H	-4.352213	-0.717619	-0.449494
Cu	-2.433272	-2.268081	0.180052

SCF Energy: -1303.17561995
Sum of electronic and zero-point Energies= -1302.917059
Sum of electronic and thermal Energies= -1302.893013

Sum of electronic and thermal Enthalpies= -1302.892069
Sum of electronic and thermal Free Energies= -1302.971986

==> ./AOX-II/PA/mono-/c3c4/HAT/c5 <==

O	-1.926188	1.036040	0.019049
O	0.268242	-1.761240	0.493830
O	2.420355	2.940727	0.410272
O	2.018390	0.160832	0.455475
O	-1.357976	5.627915	-0.385007
C	0.333613	1.827358	0.189626
C	-1.026872	2.041045	0.029785
C	-1.559020	-0.234669	0.178046
C	0.820437	0.493738	0.333944
C	-0.213660	-0.536217	0.340373
C	-2.667054	-1.180375	0.110117
C	1.209957	3.056308	0.186659
C	-1.572204	3.302872	-0.185006
C	-0.772845	4.523173	-0.214059
C	0.604657	4.329885	-0.051988
C	-3.684085	-0.972354	-0.833486
C	-2.740544	-2.278183	0.978930
C	-4.740457	-1.864382	-0.919636
C	-3.808934	-3.159681	0.890538
C	-4.804041	-2.959737	-0.060843
H	-2.640062	3.378315	-0.361913
H	1.267999	5.187509	-0.054816
H	-3.629579	-0.124975	-1.506619
H	-1.996293	-2.420039	1.755064
H	-5.515906	-1.707363	-1.660550
H	-3.868130	-3.998103	1.574738
H	-5.633760	-3.654256	-0.130567
H	-0.392541	-2.456932	0.360391
O	3.083204	-3.597830	-1.022406
H	3.889424	-3.959357	-0.639877
O	4.866018	-1.103725	0.191611
H	5.399857	-1.274606	-0.590194
H	2.395356	-4.227616	-0.784069
H	4.874937	-0.145907	0.284141
Cu	2.710298	-1.701763	-0.185736

SCF Energy: -1302.31496611
Sum of electronic and zero-point Energies= -1302.070317
Sum of electronic and thermal Energies= -1302.046665
Sum of electronic and thermal Enthalpies= -1302.045721
Sum of electronic and thermal Free Energies= -1302.125750

==> ./AOX-II/PA/mono-/c3c4/HAT/c3 <==

O	-1.293907	-1.704934	0.123185
O	1.471778	0.317994	0.716915
O	-2.825433	2.839939	-0.370534
O	-0.210889	2.210855	-0.219870
O	-5.863965	-0.729683	0.019138
C	-1.968657	0.619265	-0.083260
C	-2.299443	-0.747302	0.066662
C	-0.024762	-1.424475	0.148617
C	-0.629468	1.042400	-0.063019
C	0.370476	-0.010510	0.278748
C	0.876758	-2.527854	0.062102
C	-3.076151	1.530753	-0.216368
C	-3.566724	-1.225610	0.096084
C	-4.686392	-0.299522	-0.018570
C	-4.371530	1.087735	-0.182721
C	0.368962	-3.842491	0.182290
C	2.254121	-2.336828	-0.193199
C	1.217859	-4.923188	0.077072
C	3.088727	-3.431390	-0.311060
C	2.577401	-4.720053	-0.170542
H	-3.758666	-2.287188	0.188494
H	-5.183379	1.799065	-0.280415
H	-0.684326	-3.995881	0.377338
H	2.656643	-1.342723	-0.324269
H	0.829193	-5.928435	0.184762
H	4.141225	-3.283576	-0.519738
H	3.240059	-5.573463	-0.259568
H	-1.857216	2.979930	-0.378366
O	4.148240	1.618127	-0.714337
H	4.847723	2.036060	-0.202196
O	2.355222	4.274659	0.424344
H	2.862246	4.671626	-0.290992
H	4.283761	0.674102	-0.582842
H	1.471041	4.642747	0.326872
Cu	2.210906	2.146431	0.134247

SCF Energy: -1302.36403429
Sum of electronic and zero-point Energies= -1302.117539
Sum of electronic and thermal Energies= -1302.093682
Sum of electronic and thermal Enthalpies= -1302.092738
Sum of electronic and thermal Free Energies= -1302.171942

==> ./AOX-II/PA/mono-/c3c4/HAT/h2o <==

O	-0.998923	1.912757	0.030776
O	-0.704317	-1.649531	0.478697
O	3.695229	0.841684	0.309804
O	1.793328	-1.003109	0.499180
O	2.233674	5.256380	-0.304204
C	1.331214	1.296316	0.191655

C 0.316489 2.274970 0.040055
C -1.395274 0.647674 0.156379
C 0.964192 -0.054015 0.351365
C -0.445519 -0.346713 0.327613
C -2.833717 0.463384 0.080224
C 2.714586 1.740069 0.166966
C 0.591846 3.593922 -0.139879
C 1.984428 4.048024 -0.154441
C 3.016951 3.056987 0.000358
C -3.579644 1.267656 -0.797381
C -3.489939 -0.485617 0.881469
C -4.949731 1.098459 -0.892398
C -4.865016 -0.636298 0.786147
C -5.594244 0.145886 -0.104276
H -0.201138 4.313743 -0.297028
H 4.050680 3.379262 -0.014537
H -3.075477 2.000989 -1.414978
H -2.943165 -1.060328 1.620080
H -5.519550 1.708195 -1.583793
H -5.368852 -1.357382 1.418948
H -6.668450 0.019817 -0.179418
H -1.598590 -1.907174 0.200126
H 3.307773 -0.049454 0.420201
O 0.234266 -4.404603 -0.681793
H 0.906971 -5.044589 -0.930417
O 3.121407 -3.594619 -0.224108
H 3.167780 -4.252028 -0.927531
H 3.676958 -2.867614 -0.528454
Cu 1.126483 -2.842046 -0.138384

SCF Energy: -1302.29662310
Sum of electronic and zero-point Energies= -1302.049131
Sum of electronic and thermal Energies= -1302.026583
Sum of electronic and thermal Enthalpies= -1302.025638
Sum of electronic and thermal Free Energies= -1302.101654

==> ./AOX-II/PA/mono-/c3c4/aIP <==
O 1.684168 1.404790 -0.020813
O 0.031350 -1.803973 0.018378
O -3.042100 2.265925 -0.071833
O -2.034462 -0.192111 -0.022716
O 0.062933 5.778806 -0.056853
C -0.684076 1.749246 -0.049137
C 0.622867 2.239793 -0.029454
C 1.554021 0.074894 -0.033214
C -0.877889 0.334890 -0.034528
C 0.292063 -0.479373 -0.013095
C 2.828132 -0.638711 -0.026736
C -1.781053 2.708501 -0.061134
C 0.903835 3.598050 -0.030077
C -0.194669 4.561695 -0.052187
C -1.536201 4.047433 -0.066100
C 3.900612 -0.115670 0.708757
C 3.001941 -1.816424 -0.766175
C 5.119196 -0.776153 0.720674
C 4.230040 -2.462978 -0.756751
C 5.285862 -1.950038 -0.009976
H 1.926466 3.952054 -0.015580
H -2.362010 4.747759 -0.080080
H 3.769745 0.795985 1.279385
H 2.196382 -2.208731 -1.375516
H 5.941466 -0.374385 1.301582
H 4.363057 -3.366294 -1.340862
H 6.241326 -2.462605 -0.000529
H 0.722314 -2.333583 0.438884
H -3.051942 1.289982 -0.059343
O -2.166206 -4.183285 0.269884
H -3.061399 -4.532107 0.176101
O -4.309840 -2.034756 0.024153
H -4.676837 -2.727868 0.584478
H -1.659646 -4.590867 -0.443430
H -4.561742 -1.207241 0.450054
Cu -2.192483 -2.183317 0.033036

SCF Energy: -1302.80699503
Sum of electronic and zero-point Energies= -1302.546179
Sum of electronic and thermal Energies= -1302.523684
Sum of electronic and thermal Enthalpies= -1302.522740
Sum of electronic and thermal Free Energies= -1302.597137

==> ./AOX-II/PA/mono-/c3c4/RAF/c8 <==
O -2.075479 -0.026814 -0.168709
O 1.083439 1.723293 0.098409
O 1.278170 -3.382415 -0.214793
O 1.871252 -0.836664 -0.127515
O -3.236330 -4.482226 0.264612
C -0.346790 -1.618681 -0.203869
C -1.668123 -1.276467 -0.220042
C -1.204906 1.017730 -0.084792
C 0.643717 -0.578172 -0.133318
C 0.139247 0.776332 -0.043146
C -1.887490 2.312992 -0.011649
C -0.002817 -3.043868 -0.197958
C -2.750008 -2.278824 -0.411893
C -2.334852 -3.697897 -0.038437
C -0.952619 -4.025100 -0.124829
C -3.034925 2.445574 0.780556
C -1.424212 3.413804 -0.741474

C -3.693744 3.664376 0.852569
C -2.091680 4.630076 -0.666139
C -3.223135 4.759261 0.132225
H -0.644534 -5.057581 -0.014578
H -3.402014 1.595739 1.344630
H -0.560590 3.316469 -1.390205
H -4.576798 3.760160 1.474404
H -1.730412 5.474700 -1.241952
H -3.741346 5.710123 0.189858
H 0.700238 2.574868 0.340692
H 1.824615 -2.564706 -0.203841
O 5.163255 1.405131 -0.928691
H 5.978051 1.170831 -0.472522
O 4.644837 -0.053821 2.005400
H 5.300351 0.646970 2.068065
H 5.326987 1.180269 -1.849844
H 4.002746 0.164147 2.686992
Cu 3.570947 0.291966 -0.143617
H -2.873918 -2.336612 -1.511169
O -3.948903 -1.895060 0.197271
H -4.408770 -2.720593 0.409642

SCF Energy: -1378.83058858
Sum of electronic and zero-point Energies= -1378.556168
Sum of electronic and thermal Energies= -1378.529867
Sum of electronic and thermal Enthalpies= -1378.528923
Sum of electronic and thermal Free Energies= -1378.615385

==> ./AOX-II/PA/mono-/c3c4/RAF/c2 <==
O -1.793848 1.469346 0.031147
O -0.489949 -1.815810 0.076101
O 3.024676 1.830464 -0.148506
O 1.825045 -0.490194 -0.129370
O 0.303986 5.644418 -0.045909
C 0.632883 1.550635 0.001056
C -0.646563 2.207381 0.049940
C -1.794994 0.178253 0.597945
C 0.741345 0.156642 0.003612
C -0.519526 -0.557137 0.186047
C -3.040012 -0.530774 0.101660
C 1.812534 2.398734 -0.086744
C -0.762222 3.553411 0.037160
C 0.418033 4.402163 -0.034414
C 1.703680 3.754992 -0.095011
C -3.456800 -0.356044 -1.220702
C -3.742105 -1.395939 0.941567
C -4.579530 -1.024699 -1.687613
C -4.868100 -2.062453 0.466510
C -5.288416 -1.876686 -0.844298
H -1.739380 4.019568 0.061393
H 2.594796 4.368870 -0.148100
H -2.908819 0.307058 -1.879596
H -3.422003 -1.551140 1.965456
H -4.902619 -0.879014 -2.712262
H -5.414502 -2.725657 1.127511
H -6.166846 -2.395892 -1.211519
H -1.362816 -2.245012 0.196451
H 2.918756 0.855542 -0.154666
O 3.243621 -4.213227 0.041068
H 4.197738 -4.123746 -0.054000
O 5.305266 -0.591233 -0.563126
H 4.997980 0.184740 -1.039860
H 3.120404 -4.656693 0.886927
H 4.497894 -1.081275 -0.375769
Cu 2.359896 -2.395909 0.018024
O -1.668445 0.229987 1.981994
H -2.511621 0.503121 2.367864

SCF Energy: -1378.79384365
Sum of electronic and zero-point Energies= -1378.519675
Sum of electronic and thermal Energies= -1378.493878
Sum of electronic and thermal Enthalpies= -1378.492933
Sum of electronic and thermal Free Energies= -1378.577822

==> ./AOX-II/PA/mono-/c3c4/RAF/c4a <==
O -1.937076 1.203916 0.142739
O -0.161883 -1.965126 -0.103659
O 2.727129 2.260793 0.924370
O 1.848076 -0.213002 0.412604
O -0.157523 5.344760 -1.078747
C 0.373198 1.645864 0.757459
C -0.894283 2.083099 0.091887
C -1.717195 -0.131349 0.097239
C 0.680949 0.192037 0.353133
C -0.446466 -0.646331 0.081003
C -2.962459 -0.894232 0.003656
C 1.519574 2.608082 0.471630
C -1.076030 3.269483 -0.485375
C 0.028689 4.234100 -0.573441
C 1.327535 3.814930 -0.093868
C -4.044396 -0.363921 -0.713144
C -3.102390 -2.133797 0.643098
C -5.233160 -1.072109 -0.805508
C -4.300916 -2.828358 0.557836
C -5.364393 -2.304424 -0.170938
H -2.029852 3.533365 -0.925229
H 2.161933 4.496196 -0.209456
H -3.944696 0.594872 -1.207394

H -2.288778 -2.541876 1.230397
H -6.060119 -0.660534 -1.373103
H -4.405281 -3.779449 1.067504
H -6.296682 -2.853915 -0.240817
H -0.835691 -2.387011 -0.648533
H 2.781817 1.289508 0.991056
O 3.644025 -3.858610 0.325603
H 4.597760 -3.837943 0.198339
O 3.420825 -1.368487 -2.263207
H 3.201268 -2.073654 -2.879033
H 3.528973 -4.085950 1.253469
H 2.831927 -0.648844 -2.508637
Cu 2.800239 -1.983377 -0.095991
O 0.109648 1.639734 2.155661
H 0.875168 1.282910 2.624266

SCF Energy: -1378.79080752
Sum of electronic and zero-point Energies= -1378.516825
Sum of electronic and thermal Energies= -1378.491004
Sum of electronic and thermal Enthalpies= -1378.490060
Sum of electronic and thermal Free Energies= -1378.572716

==> ./AOX-II/PA/mono-/c3c4/RAF/c5 <==
O -0.948184 -1.891152 -0.071336
O -1.507451 1.706871 -0.257672
O 3.337680 0.082747 -0.583833
O 1.179309 1.532252 -0.488882
O 2.908544 -4.479440 0.289319
C 1.150926 -0.805849 -0.378603
C 0.391949 -2.001492 -0.161601
C -1.607302 -0.697181 -0.144251
C 0.505733 0.478034 -0.378385
C -0.934474 0.482071 -0.254390
C -3.060412 -0.865300 -0.040518
C 2.525936 -0.928501 -0.490497
C 0.924218 -3.250345 0.001657
C 2.336792 -3.436194 -0.028703
C 3.163287 -2.277989 -0.593279
C -3.588351 -1.774945 0.884743
C -3.929691 -0.143458 -0.867294
C -4.961474 -1.940233 0.992755
C -5.303480 -0.319550 -0.757306
C -5.821856 -1.212541 0.174368
H 0.282329 -4.083258 0.258286
H 3.115249 -2.482279 -1.680904
H -2.921238 -2.340719 1.524610
H -3.535401 0.532467 -1.618570
H -5.361924 -2.639159 1.718591
H -5.967839 0.236574 -1.409101
H -6.894647 -1.346318 0.259838
H -2.445486 1.655107 -0.041338
H 2.800580 0.913407 -0.575028
O 0.447386 5.421290 0.209257
H 0.803859 5.674952 1.067142
O 2.115831 1.196920 2.664912
H 2.699672 0.709198 3.251877
H 0.920842 5.970133 -0.424650
H 2.504147 1.073586 1.794376
Cu 0.761341 3.455536 -0.141444
O 4.480701 -2.319067 -0.134351
H 5.059629 -1.926769 -0.794726

SCF Energy: -1378.82375144
Sum of electronic and zero-point Energies= -1378.548732
Sum of electronic and thermal Energies= -1378.522785
Sum of electronic and thermal Enthalpies= -1378.521841
Sum of electronic and thermal Free Energies= -1378.607072

==> ./AOX-II/PA/mono-/c3c4/RAF/c1p <==
O -1.642163 1.444571 0.153577
O -0.280445 -1.929649 0.092800
O 3.138610 1.891757 0.031723
O 1.940229 -0.458750 0.067624
O 0.353052 5.688121 0.051186
C 0.748921 1.562273 0.088685
C -0.512771 2.205044 0.112963
C -1.599454 0.098682 0.188654
C 0.832728 0.151510 0.094890
C -0.429889 -0.590699 0.130371
C -2.963426 -0.497426 0.175583
C 1.894952 2.423460 0.052924
C -0.677891 3.565252 0.101584
C 0.465733 4.422108 0.063739
C 1.754045 3.785369 0.040861
C -3.960811 -0.040830 -0.819058
C -3.193723 -1.805151 0.796905
C -5.040360 -0.940204 -1.215032
C -4.251386 -2.553198 0.428849
C -5.173152 -2.132789 -0.609292
H -1.674056 3.991304 0.122127
H 2.641905 4.408358 0.015396
H -3.681859 0.762135 -1.487883
H -2.508122 -2.136126 1.566415
H -5.675137 -0.630855 -2.034805
H -4.413526 -3.512948 0.904354
H -5.957984 -2.815509 -0.910515
H -1.111870 -2.395884 -0.047019
H 3.048579 0.918593 0.048172

O	3.419070	-4.172115	0.096711
H	4.291382	-4.129450	-0.308992
O	5.296750	-0.462630	-0.917531
H	5.021825	0.422323	-1.173004
H	3.580319	-4.470960	0.997949
H	4.492496	-0.862216	-0.570228
Cu	2.532622	-2.355349	0.094058
O	-3.996156	0.577351	0.574079
H	-4.692669	0.206713	1.153237

SCF Energy: -1378.73066434
Sum of electronic and zero-point Energies= -1378.455211
Sum of electronic and thermal Energies= -1378.430372
Sum of electronic and thermal Enthalpies= -1378.429428
Sum of electronic and thermal Free Energies= -1378.509801

==> ./AOX-II/PA/mono-/c3c4/RAF/c6 <==

O	1.728438	-1.450096	0.006755
O	0.639085	2.021884	-0.244148
O	-3.014991	-1.552502	-0.024679
O	-1.708107	0.690566	-0.127307
O	-0.602226	-5.453850	0.564262
C	-0.650018	-1.398648	-0.068011
C	0.573476	-2.138726	0.007298
C	1.790890	-0.085061	-0.046856
C	-0.637992	0.038726	-0.100489
C	0.663830	0.675271	-0.123086
C	3.177194	0.394812	-0.051785
C	-1.842098	-2.108363	-0.062582
C	0.665623	-3.499900	0.145537
C	-0.523951	-4.267606	0.229397
C	-1.816302	-3.586308	-0.210290
C	4.138954	-0.277125	-0.817259
C	3.560665	1.502436	0.713529
C	5.453443	0.166362	-0.829972
C	4.880374	1.937140	0.699766
C	5.827180	1.274569	-0.073905
H	1.627741	-3.964838	0.317897
H	3.851447	-1.139187	-1.407959
H	2.839048	2.006346	1.347297
H	6.189226	-0.354921	-1.432181
H	5.168746	2.789558	1.304555
H	6.856241	1.616514	-0.083662
H	1.521793	2.380119	-0.393577
H	-2.887100	-0.570260	-0.030343
O	-2.181113	4.597991	0.991867
H	-2.633211	4.587939	1.841458
O	-4.092851	2.924500	-1.290892
H	-4.155788	3.865025	-1.480681
H	-1.285565	4.884329	1.196847
H	-3.924746	2.521982	-2.147786
Cu	-2.164415	2.683802	0.134913
H	-1.762250	-3.699347	-1.315313
O	-2.959023	-4.206215	0.293194
H	-2.678834	-5.097218	0.550807

SCF Energy: -1378.82215340
Sum of electronic and zero-point Energies= -1378.548541
Sum of electronic and thermal Energies= -1378.522355
Sum of electronic and thermal Enthalpies= -1378.521411
Sum of electronic and thermal Free Energies= -1378.606129

==> ./AOX-II/PA/mono-/c3c4/RAF/c6p <==

O	1.588772	-1.553317	-0.187570
O	0.333638	1.819719	0.045759
O	-3.162663	-1.847040	0.480739
O	-1.892600	0.456498	0.399109
O	-0.571544	-5.703560	-0.240920
C	-0.788017	-1.598160	0.137645
C	0.443187	-2.271239	-0.091013
C	1.643385	-0.194327	-0.097248
C	-0.828953	-0.197433	0.201474
C	0.448759	0.520072	0.020471
C	2.983480	0.284694	-0.021176
C	-1.964100	-2.422544	0.253320
C	0.541691	-3.626914	-0.220680
C	-0.631601	-4.447478	-0.119806
C	-1.883542	-3.779706	0.129376
C	4.025467	-0.646863	0.110982
C	3.353052	1.737412	0.056396
C	5.341694	-0.253036	0.206965
C	4.800676	2.058736	0.035568
C	5.746522	1.109567	0.167413
H	1.505787	-4.088969	-0.396187
H	-2.783714	-4.378114	0.216109
H	3.795709	-1.701432	0.118051
H	6.102305	-1.020630	0.295058
H	5.048186	3.112858	-0.029750
H	6.797725	1.363490	0.205955
H	1.141436	2.266328	-0.322850
H	-3.029394	-0.879704	0.541342
O	-3.156426	4.238619	-0.062086
H	-4.101150	4.214116	-0.247119
O	-5.143941	0.652521	-0.676043
H	-6.028938	0.510404	-1.022392
H	-3.080940	4.736623	0.758583
H	-4.656422	-0.127730	-0.955294
Cu	-2.444874	2.355687	0.166286

H	3.052204	2.020771	1.090874
O	2.619784	2.508701	-0.876787
H	2.828198	3.443257	-0.750407

SCF Energy: -1378.77630345
Sum of electronic and zero-point Energies= -1378.503588
Sum of electronic and thermal Energies= -1378.478280
Sum of electronic and thermal Enthalpies= -1378.477336
Sum of electronic and thermal Free Energies= -1378.561654

==> ./AOX-II/PA/mono-/c3c4/RAF/c4 <==

O	0.394220	1.699431	0.982807
O	-0.292995	0.010802	-2.071453
O	2.925605	-0.596933	-2.254055
O	1.332862	-2.140639	-0.580833
O	5.096224	1.996972	0.983094
C	1.597961	0.237953	-0.371697
C	1.647139	1.284401	0.689618
C	-0.424861	1.359078	-0.098066
C	1.326608	-1.192268	0.172212
C	0.249475	0.608345	-0.993802
C	-1.794419	1.844410	-0.028644
C	2.889403	0.153754	-1.143480
C	2.758583	1.837275	1.185076
C	4.033622	1.534890	0.544239
C	4.009653	0.737015	-0.673841
C	-2.261244	2.448460	1.147428
C	-2.668493	1.718891	-1.119586
C	-3.570345	2.904247	1.232572
C	-3.974204	2.179964	-1.025333
C	-4.432664	2.771079	0.149305
H	2.719704	2.606615	1.946195
H	4.946682	0.588587	-1.197409
H	-1.599943	2.558128	1.997890
H	-2.326354	1.269123	-2.042243
H	-3.916113	3.365904	2.151085
H	-4.637340	2.079115	-1.877612
H	-5.454437	3.128269	0.217634
H	0.333897	-0.044810	-2.803281
H	2.241106	-1.284676	-2.217637
O	-0.430558	-3.507348	1.887824
H	-0.915412	-3.566095	2.718417
O	-2.407515	-1.793054	-1.253995
H	-3.096451	-1.194696	-0.945946
H	-0.080214	-4.390525	1.726739
H	-1.738009	-1.210673	-1.648129
Cu	-1.542507	-2.752896	0.315246
O	1.039314	-1.233032	1.450666
H	0.681042	-2.124830	1.683715

SCF Energy: -1378.82512923
Sum of electronic and zero-point Energies= -1378.549024
Sum of electronic and thermal Energies= -1378.524293
Sum of electronic and thermal Enthalpies= -1378.523349
Sum of electronic and thermal Free Energies= -1378.604236

==> ./AOX-II/PA/mono-/c3c4/RAF/c5p <==

O	1.060914	-1.826729	-0.069295
O	0.605896	1.775000	0.112063
O	-3.640862	-1.097854	0.621600
O	-1.916694	0.896950	0.427892
O	-1.914581	-5.449889	0.131856
C	-1.265886	-1.355889	0.270779
C	-0.214646	-2.285688	0.076918
C	1.355119	-0.507503	-0.028253
C	-0.999267	0.034416	0.279131
C	0.390635	0.438839	0.108293
C	2.803289	-0.279980	-0.189007
C	-2.577752	-1.912531	0.425960
C	-0.395853	-3.644306	0.028581
C	-1.705110	-4.196187	0.177323
C	-2.781076	-3.266059	0.381619
C	3.510745	-0.990699	-1.181595
C	3.498775	0.590797	0.617329
C	4.882947	-0.841716	-1.385106
C	4.942958	0.691999	0.516800
C	5.592154	0.038655	-0.609490
H	0.454013	-4.299344	-0.123566
H	-3.787467	-3.653055	0.502472
H	2.963485	-1.677508	-1.816892
H	3.016625	1.153374	1.408947
H	5.371734	-1.389657	-2.179371
H	6.655733	0.198965	-0.746867
H	1.444907	2.007111	-0.300380
H	-3.316459	-0.176294	0.616480
O	-2.287281	4.820936	-0.223047
H	-3.155824	4.970566	-0.611199
O	-4.341153	1.086606	-1.741498
H	-3.595145	1.349130	-1.191228
H	-2.297390	5.317949	0.601693
H	-4.568290	0.214230	-1.406927
Cu	-1.975737	2.858558	0.120251
H	5.112834	-0.197132	1.223733
O	5.476843	1.851120	1.047331
H	6.430743	1.749475	1.144912

SCF Energy: -1378.76634051

Sum of electronic and zero-point Energies= -1378.493916
Sum of electronic and thermal Energies= -1378.468156
Sum of electronic and thermal Enthalpies= -1378.467211
Sum of electronic and thermal Free Energies= -1378.550632

==> ./AOX-II/PA/mono-/c3c4/RAF/c2p <==

O	1.784899	1.058127	0.042396
O	-0.109403	-2.001193	0.325655
O	-2.867848	2.266435	0.061378
O	-2.059455	-0.243624	0.135988
O	0.488681	5.556587	0.071164
C	-0.559633	1.561299	0.065934
C	0.795511	1.989177	0.060792
C	1.561131	-0.284854	0.025818
C	-0.873580	0.192700	0.111011
C	0.258370	-0.746846	0.155168
C	2.759972	-1.050923	-0.072461
C	-1.560372	2.597305	0.063656
C	1.172482	3.300687	0.062563
C	0.174885	4.333272	0.066700
C	-1.203440	3.915289	0.063199
C	4.086722	-0.350501	0.172746
C	2.786785	-2.423447	-0.319765
C	5.288309	-1.207144	0.136920
C	3.970721	-3.140635	-0.279993
C	5.224688	-2.550323	0.004418
H	2.223206	3.566120	0.060931
H	-1.976286	4.675992	0.061000
H	1.899214	-2.963594	-0.620405
H	6.234320	-0.686666	0.232032
H	3.931643	-4.199874	-0.507180
H	6.118120	-3.160310	0.020765
H	0.605184	-2.570103	0.638357
H	-2.938082	1.291513	0.077526
O	-4.092394	-3.682199	0.152721
H	-5.012561	-3.463695	-0.028256
O	-5.394687	0.192871	-0.841651
H	-5.227642	1.091048	-0.542753
H	-3.838406	-4.283954	-0.554835
H	-4.558002	-0.260053	-0.693296
Cu	-2.938740	-2.025881	0.149165
H	4.009373	-0.084996	1.251256
O	4.348204	0.794762	-0.600030
H	3.674093	1.454657	-0.402107

SCF Energy: -1378.76929652
Sum of electronic and zero-point Energies= -1378.494310
Sum of electronic and thermal Energies= -1378.469091
Sum of electronic and thermal Enthalpies= -1378.468146
Sum of electronic and thermal Free Energies= -1378.550164

==> ./AOX-II/PA/mono-/c3c4/RAF/c3 <==

O	1.882179	1.258268	-0.007258
O	0.402386	-1.448282	-1.134208
O	-2.850188	2.210017	0.185426
O	-1.887966	-0.271917	0.229579
O	0.319768	5.667238	-0.066505
C	-0.517918	1.619830	0.091145
C	0.795801	2.138092	0.014795
C	1.814999	-0.020457	0.012683
C	-0.744655	0.242871	0.131458
C	0.452156	-0.718685	0.057689
C	3.058356	-0.720372	-0.023499
C	-1.568207	2.602892	0.118396
C	1.115368	3.450049	-0.035360
C	0.052198	4.439935	-0.014392
C	-1.287779	3.944664	0.067306
C	4.261650	0.023083	-0.058109
C	3.116237	-2.132126	-0.036947
C	5.475676	-0.627683	-0.105708
C	4.340783	-2.769499	-0.085896
C	5.516663	-2.023114	-0.120659
H	2.148428	3.769099	-0.090448
H	-2.105596	4.656095	0.085299
H	4.227792	1.104321	-0.046830
H	2.212279	-2.721037	-0.012547
H	6.394802	-0.055489	-0.131744
H	4.382364	-3.851638	-0.097828
H	6.473691	-2.531017	-0.159328
H	-0.498045	-1.769024	-1.281572
H	-2.881098	1.235378	0.221501
O	-3.493085	-3.939967	0.096348
H	-4.366781	-3.894629	-0.306424
O	-5.208209	-0.067817	-0.720646
H	-5.142387	0.704719	-0.152214
H	-3.003820	-4.565309	-0.448484
H	-4.319427	-0.437687	-0.712978
Cu	-2.604872	-2.128431	0.114807
O	0.479684	-1.513454	1.204140
H	-0.425418	-1.768874	1.431767

SCF Energy: -1378.80593552
Sum of electronic and zero-point Energies= -1378.528673
Sum of electronic and thermal Energies= -1378.503916

Sum of electronic and thermal Enthalpies= -1378.502972
Sum of electronic and thermal Free Energies= -1378.583007

==> ./AOX-II/PA/mono-/c3c4/RAF/c8a <==

O	-1.887212	1.035745	-0.100351
O	-0.069786	-2.116568	0.188723
O	2.628730	2.268697	-0.257664
O	1.930842	-0.282889	0.092496
O	-0.784310	5.481013	-0.436857
C	0.427426	1.500021	0.234518
C	-0.969433	1.889922	0.575996
C	-1.660158	-0.304600	0.007183
C	0.736840	0.103000	0.185157
C	-0.390860	-0.800485	0.163006
C	-2.878487	-1.099176	-0.154995
C	1.335554	2.492579	-0.047514
C	-1.343985	3.303610	0.278971
C	-0.442604	4.296228	-0.162791
C	0.911473	3.846002	-0.184212
C	-3.852871	-0.685153	-1.075500
C	-3.105373	-2.250170	0.612026
C	-5.013692	-1.424693	-1.241714
C	-4.276951	-2.977486	0.448264
C	-5.228691	-2.572191	-0.481863
H	-2.360785	3.580200	0.544890
H	1.689461	4.585357	-0.349728
H	-3.689073	0.206871	-1.668275
H	-2.387962	-2.558310	1.363746
H	-5.754826	-1.103877	-1.965109
H	-4.449062	-3.858298	1.056443
H	-6.140289	-3.145202	-0.610777
H	-0.774684	-2.659400	-0.182728
H	2.794299	1.309254	-0.145656
O	3.952612	-3.725153	0.049525
H	4.787673	-3.568417	0.502689
O	5.200435	0.199136	-1.022029
H	4.880234	1.077375	-1.245346
H	3.537997	-4.444141	0.537677
H	4.399104	-0.297247	-0.826364
Cu	2.795540	-2.067178	0.067576
O	-1.111022	1.744519	1.967201
H	-1.974177	2.083544	2.241284

SCF Energy: -1378.73722284
Sum of electronic and zero-point Energies= -1378.463968
Sum of electronic and thermal Energies= -1378.438054
Sum of electronic and thermal Enthalpies= -1378.437110
Sum of electronic and thermal Free Energies= -1378.521462

==> ./AOX-II/PA/mono-/c3c4/RAF/c7 <==

O	1.724552	-1.103217	0.011363
O	0.267637	2.201163	0.014441
O	-2.990221	-1.746218	0.544639
O	-1.908672	0.635528	0.192198
O	0.319008	-4.028297	-1.045216
C	-0.622478	-1.338483	0.178897
C	0.637920	-1.862127	0.063936
C	1.661301	0.247255	0.039928
C	-0.771966	0.092324	0.149167
C	0.441099	0.868178	0.065547
C	2.980598	0.884633	-0.013268
C	-1.750329	-2.259837	0.389624
C	0.909220	-3.303075	0.031436
C	-0.243246	-4.183662	0.258456
C	-1.565872	-3.592289	0.476342
C	3.959171	0.379656	-0.878558
C	3.281749	1.980894	0.803154
C	5.211624	0.973903	-0.933750
C	4.539587	2.568246	0.743667
C	5.503863	2.069709	-0.125613
H	1.909429	-3.618094	0.311481
H	-2.398551	-4.246421	0.706811
H	3.731848	-0.470143	-1.511989
H	2.548229	2.356766	1.508481
H	5.961575	0.581776	-1.611577
H	4.767313	3.410896	1.386661
H	6.484187	2.531111	-0.170986
H	1.098086	2.662301	-0.154110
H	-2.938091	-0.774279	0.474452
O	-3.439666	4.350284	0.189366
H	-4.311633	4.301787	-0.216302
O	-3.595760	0.130546	-2.267558
H	-3.807414	-0.772315	-2.014889
H	-3.602565	4.653935	1.088609
H	-3.052920	0.447167	-1.535587
Cu	-2.546300	2.531920	0.193625
O	0.015204	-5.395428	0.855123
H	-0.716893	-5.997003	0.675183

SCF Energy: -1378.79905927
Sum of electronic and zero-point Energies= -1378.524947
Sum of electronic and thermal Energies= -1378.499056
Sum of electronic and thermal Enthalpies= -1378.498112
Sum of electronic and thermal Free Energies= -1378.583328

==> ./AOX-II/PA/mono-/c3c4/RAF/c4p <==

O	1.357031	1.624989	-0.004484
O	0.025121	-1.724572	0.280717
O	-3.439229	1.983445	-0.084517
O	-2.205273	-0.341474	-0.004560
O	-0.718176	5.818570	-0.093946
C	-1.042568	1.700451	-0.032175
C	0.217228	2.361932	-0.016492
C	1.362896	0.266007	0.015217
C	-1.113345	0.298331	0.004787
C	0.164927	-0.423973	0.095338
C	2.688785	-0.277799	-0.015011
C	-2.210646	2.542339	-0.070615
C	0.352417	3.720383	-0.037436
C	-0.811076	4.558457	-0.075316
C	-2.093697	3.902632	-0.092093
C	3.772433	0.566692	0.377492
C	2.963671	-1.610574	-0.450740
C	5.042225	0.097904	0.387865
C	4.233602	-2.079505	-0.481930
C	5.385571	-1.215440	-0.185540
H	1.338265	4.169453	-0.029273
H	-2.989008	4.513774	-0.122932
H	3.560154	1.561253	0.742552
H	2.175992	-2.256755	-0.813470
H	5.856518	0.708530	0.762824
H	4.444017	-3.085689	-0.824957
H	5.691462	-0.929706	-1.225152
H	0.828614	-2.161021	0.589484
H	-3.331734	1.011656	-0.064603
O	-3.285765	-4.042868	-1.077607
H	-3.910772	-4.394349	-0.434395
O	-3.910495	-2.803416	2.639085
H	-3.666537	-2.443519	1.780004
H	-3.776945	-4.009866	-1.905261
H	-3.072717	-2.877444	3.103841
Cu	-2.685082	-2.194288	-0.525080
O	6.427567	-1.916324	0.441963
H	7.239286	-1.405780	0.355333

SCF Energy: -1378.77190633
Sum of electronic and zero-point Energies= -1378.499162
Sum of electronic and thermal Energies= -1378.473745
Sum of electronic and thermal Enthalpies= -1378.472801
Sum of electronic and thermal Free Energies= -1378.556128

==> ./AOX-II/PA/mono-/c3c4/RAF/c3p <==

O	1.543117	1.342844	-0.110645
O	0.064824	-1.972731	0.030737
O	-3.217627	2.001245	-0.032248
O	-2.128255	-0.401433	0.000636
O	-0.269834	5.672266	-0.093052
C	-0.844482	1.565541	-0.064016
C	0.444941	2.151580	-0.087018
C	1.438666	-0.004013	-0.110364
C	-0.991286	0.157145	-0.033005
C	0.235176	-0.630224	-0.032416
C	2.762952	-0.647868	-0.157800
C	-1.950538	2.476721	-0.052305
C	0.668425	3.504644	-0.097057
C	-0.435979	4.411120	-0.083910
C	-1.750469	3.831609	-0.062172
C	3.818039	-0.098365	0.539982
C	3.011954	-1.762226	-0.980504
C	5.110888	-0.751149	0.553948
C	4.270710	-2.361598	-1.079854
C	5.330943	-1.840965	-0.386643
H	1.682566	3.886121	-0.118880
H	-2.610685	4.492773	-0.054050
H	3.695703	0.781819	1.159826
H	2.202818	-2.166944	-1.577796
H	4.411001	-3.204919	-1.742643
H	6.326138	-2.266225	-0.448992
H	0.815012	-2.414440	0.442812
H	-3.168382	1.025316	-0.021791
O	-3.494565	-4.142440	0.334265
H	-4.434917	-4.085822	0.534112
O	-5.608118	-0.387621	-0.032239
H	-5.479766	0.532606	0.214036
H	-3.448738	-4.629884	-0.494962
H	-4.716173	-0.749862	-0.047765
Cu	-2.694609	-2.298485	0.142157
H	4.800649	-1.449038	1.412217
O	6.163301	0.059898	0.934402
H	6.926038	-0.483371	1.165269

SCF Energy: -1378.76596549
Sum of electronic and zero-point Energies= -1378.493196
Sum of electronic and thermal Energies= -1378.467392
Sum of electronic and thermal Enthalpies= -1378.466448
Sum of electronic and thermal Free Energies= -1378.550733

==> ./AOX-II/PA/mono-/c3c4/vEA <==

O	1.632851	1.438462	-0.004955
O	0.075596	-1.844461	-0.004470
O	-3.124912	2.177171	-0.060993
O	-2.044392	-0.262161	0.003750
O	-0.115195	5.796670	-0.079766

C	-0.753826	1.710468	-0.032911
C	0.549234	2.269938	-0.021647
C	1.531111	0.094466	-0.018702
C	-0.895250	0.311483	-0.011612
C	0.298368	-0.486423	-0.000510
C	2.838364	-0.572239	-0.014228
C	-1.848972	2.632365	-0.053456
C	0.793080	3.617600	-0.034626
C	-0.300088	4.540316	-0.061338
C	-1.625740	3.982411	-0.068015
C	3.921085	0.037861	0.632495
C	3.033771	-1.791844	-0.674225
C	5.168819	-0.569154	0.630460
C	4.287918	-2.388219	-0.679373
C	5.356125	-1.782885	-0.024935
H	1.812103	3.985944	-0.030364
H	-2.473592	4.658961	-0.086550
H	3.781243	0.983307	1.142708
H	2.215395	-2.263690	-1.203634
H	5.997721	-0.091497	1.140938
H	4.431169	-3.326710	-1.203306
H	6.333332	-2.253306	-0.028825
H	0.592535	-2.304513	0.670090
H	-3.108970	1.203392	-0.043740
O	-2.052242	-4.215965	0.250007
H	-2.789111	-4.626497	-0.218419
O	-4.265142	-2.168388	0.019324
H	-4.588828	-2.840063	0.629989
H	-1.256045	-4.566268	-0.167419
H	-4.544483	-1.328892	0.402085
Cu	-2.116517	-2.215091	0.021000

SCF Energy: -1303.15657094

==> ./AOX-II/PA/mono-/c3c4/vIP <==

O	1.632851	1.438462	-0.004955
O	0.075596	-1.844461	-0.004470
O	-3.124912	2.177171	-0.060993
O	-2.044392	-0.262161	0.003750
O	-0.115195	5.796670	-0.079766
C	-0.753826	1.710468	-0.032911
C	0.549234	2.269938	-0.021647
C	1.531111	0.094466	-0.018702
C	-0.895250	0.311483	-0.011612
C	0.298368	-0.486423	-0.000510
C	2.838364	-0.572239	-0.014228
C	-1.848972	2.632365	-0.053456
C	0.793080	3.617600	-0.034626
C	-0.300088	4.540316	-0.061338
C	-1.625740	3.982411	-0.068015
C	3.921085	0.037861	0.632495
C	3.033771	-1.791844	-0.674225
C	5.168819	-0.569154	0.630460
C	4.287918	-2.388219	-0.679373
C	5.356125	-1.782885	-0.024935
H	1.812103	3.985944	-0.030364
H	-2.473592	4.658961	-0.086550
H	3.781243	0.983307	1.142708
H	2.215395	-2.263690	-1.203634
H	5.997721	-0.091497	1.140938
H	4.431169	-3.326710	-1.203306
H	6.333332	-2.253306	-0.028825
H	0.592535	-2.304513	0.670090
H	-3.108970	1.203392	-0.043740
O	-2.052242	-4.215965	0.250007
H	-2.789111	-4.626497	-0.218419
O	-4.265142	-2.168388	0.019324
H	-4.588828	-2.840063	0.629989
H	-1.256045	-4.566268	-0.167419
H	-4.544483	-1.328892	0.402085
Cu	-2.116517	-2.215091	0.021000

SCF Energy: -1302.80211529

==> ./AOX-II/PA/mono-/c4c5/vIP <==

O	-1.868424	1.223047	-0.080186
O	-1.132169	-2.292499	0.099043
O	2.913358	0.927220	0.081483
O	1.250473	-1.334284	0.097593
O	0.733412	5.114283	-0.216241
C	0.524762	0.969239	-0.006873
C	-0.635610	1.790975	-0.073214
C	-2.072818	-0.114766	-0.031635
C	0.352341	-0.430608	0.036302
C	-0.997832	-0.948983	0.023325
C	-3.491534	-0.481308	-0.009360
C	1.769445	1.670519	0.001864
C	-0.597814	3.162656	-0.141235
C	0.649099	3.850211	-0.144615
C	1.832329	3.031492	-0.061456
C	-4.436962	0.442183	0.460880
C	-3.935946	-1.735108	-0.453072
C	-5.784597	0.113061	0.500872
C	-5.286986	-2.054077	-0.416194
C	-6.215647	-1.136448	0.064392
H	-1.524364	3.721902	-0.196868
H	2.800617	3.522828	-0.053877
H	-4.112694	1.416623	0.805278
H	-3.231145	-2.457173	-0.840722
H	-6.500827	0.835539	0.876607
H	-5.613871	-3.026296	-0.768845
H	-7.269149	-1.392422	0.096440
H	-0.232137	-2.651875	0.153239
Cu	3.179694	-1.213572	0.086134

O	5.201336	-1.051490	0.066092
H	5.600227	-1.849869	0.431780
O	3.175668	-3.299768	-0.153313
H	3.997540	-3.601301	-0.556445
H	2.483790	-3.535835	-0.781666
H	5.469150	-0.339190	0.659443
H	3.699113	1.484339	0.022090

SCF Energy: -1302.80454143

==> ./AOX-II/PA/mono-/c4c5/vEA <==

O	-1.868424	1.223047	-0.080186
O	-1.132169	-2.292499	0.099043
O	2.913358	0.927220	0.081483
O	1.250473	-1.334284	0.097593
O	0.733412	5.114283	-0.216241
C	0.524762	0.969239	-0.006873
C	-0.635610	1.790975	-0.073214
C	-2.072818	-0.114766	-0.031635
C	0.352341	-0.430608	0.036302
C	-0.997832	-0.948983	0.023325
C	-3.491534	-0.481308	-0.009360
C	1.769445	1.670519	0.001864
C	-0.597814	3.162656	-0.141235
C	0.649099	3.850211	-0.144615
C	1.832329	3.031492	-0.061456
C	-4.436962	0.442183	0.460880
C	-3.935946	-1.735108	-0.453072
C	-5.784597	0.113061	0.500872
C	-5.286986	-2.054077	-0.416194
C	-6.215647	-1.136448	0.064392
H	-1.524364	3.721902	-0.196868
H	2.800617	3.522828	-0.053877
H	-4.112694	1.416623	0.805278
H	-3.231145	-2.457173	-0.840722
H	-6.500827	0.835539	0.876607
H	-5.613871	-3.026296	-0.768845
H	-7.269149	-1.392422	0.096440
H	-0.232137	-2.651875	0.153239
Cu	3.179694	-1.213572	0.086134
O	5.201336	-1.051490	0.066092
H	5.600227	-1.849869	0.431780
O	3.175668	-3.299768	-0.153313
H	3.997540	-3.601301	-0.556445
H	2.483790	-3.535835	-0.781666
H	5.469150	-0.339190	0.659443
H	3.699113	1.484339	0.022090

SCF Energy: -1303.15251800

==> ./AOX-II/PA/mono-/c4c5/RAF/c7 <==

O	-1.902666	0.798216	-0.193314
O	-0.419388	-2.416163	0.353236
O	2.712444	1.663747	0.261227
O	1.679773	-0.900381	0.410659
O	-0.462926	5.133282	0.295747
C	0.425376	1.130861	0.041207
C	-0.854241	1.585306	-0.157045
C	-1.828280	-0.553808	-0.028111
C	0.609356	-0.287229	0.205953
C	-0.594127	-1.101200	0.165956
C	-3.136984	-1.204006	-0.059981
C	1.480998	2.142418	0.136846
C	-1.165504	3.011353	-0.454103
C	-0.109902	3.978198	0.057693
C	1.222221	3.484438	0.153566
C	-4.291936	-0.445347	0.184543
C	-3.270836	-2.571927	-0.337913
C	-5.543395	-1.043058	0.161563
C	-4.528581	-3.159704	-0.365260
C	-5.667508	-2.402151	-0.112579
H	-1.057669	3.087556	-1.554128
H	2.029747	4.183825	0.338702
H	-4.208428	0.612682	0.400933
H	-2.398855	-3.175258	-0.547630
H	-6.425364	-0.444268	0.360364
H	-4.616300	-4.217242	-0.588752
H	-6.647010	-2.867371	-0.131074
Cu	3.603750	-0.851377	-0.188722
O	5.478443	-1.402777	-0.764207
H	5.600006	-2.341770	-0.587246
O	3.168892	-3.968485	0.763544
H	3.933111	-3.875942	0.187588
H	2.809001	-3.076582	0.804405
H	6.145885	-0.963928	-0.226519
H	3.351744	2.387109	0.319099
H	0.536166	-2.545374	0.486034
O	-2.457713	3.370635	-0.062094
H	-2.415625	4.317037	0.138789

SCF Energy: -1378.83080545
Sum of electronic and zero-point Energies= -1378.554222
Sum of electronic and thermal Energies= -1378.528945
Sum of electronic and thermal Enthalpies= -1378.528001
Sum of electronic and thermal Free Energies= -1378.610914

==> ./AOX-II/PA/mono-/c4c5/RAF/c3p <==

O	-1.533850	1.216171	-0.109776
O	-0.562221	-2.236361	-0.385930
O	3.223698	1.257292	-0.638822

O 1.761387 -1.105030 -0.472672
O 0.791494 5.276215 -0.018433
C 0.866375 1.129766 -0.340794
C -0.340421 1.866404 -0.175667
C -1.638362 -0.131764 -0.197340
C 0.805215 -0.284699 -0.388891
C -0.524021 -0.892502 -0.333319
C -3.024392 -0.592516 -0.075494
C 2.058882 1.919342 -0.429402
C -0.395715 3.234363 -0.070488
C 0.798511 4.009980 -0.127432
C 2.023378 3.283562 -0.322781
C -3.980458 0.241085 0.472361
C -3.443518 -1.850152 -0.556864
C -5.334950 -0.223446 0.687225
C -4.764659 -2.294525 -0.463727
C -5.726942 -1.485324 0.079669
H -1.353506 3.724426 0.060254
H 2.949412 3.846442 -0.396232
H -3.733517 1.230910 0.835173
H -2.719876 -2.499124 -1.030725
H -5.065982 -0.710346 1.692668
H -5.029819 -3.263356 -0.865727
H -6.764638 -1.790061 0.154444
Cu 3.566096 -1.104503 0.402499
O 5.355095 -1.509089 1.291468
H 5.441377 -2.450898 1.471890
O 2.425982 -4.273908 -0.607076
H 1.808053 -4.239526 -1.342678
H 2.579844 -3.346678 -0.396229
H 5.412254 -1.088393 2.155700
H 3.948683 1.892311 -0.675363
H 0.366871 -2.523378 -0.442998
O -6.266919 0.782956 0.861918
H -7.086785 0.404863 1.199598

SCF Energy: -1378.76615734
Sum of electronic and zero-point Energies= -1378.494058
Sum of electronic and thermal Energies= -1378.468349
Sum of electronic and thermal Enthalpies= -1378.467405
Sum of electronic and thermal Free Energies= -1378.549874

==> ./AOX-II/PA/mono-/c4c5/RAF/c4p <==
O -0.841113 1.587251 -0.068825
O -0.806454 -1.632121 1.500752
O 3.714040 0.664839 1.113145
O 1.634717 -1.044894 1.764000
O 2.488071 4.734186 -0.975456
C 1.423473 1.049814 0.597952
C 0.470934 1.930419 0.002867
C -1.340391 0.407171 0.396725
C 0.985390 -0.168603 1.160353
C -0.462829 -0.473218 1.006315
C -2.736941 0.250290 0.204263
C 2.784470 1.509529 0.594616
C 0.798159 3.151829 -0.521424
C 2.151943 3.611602 -0.501836
C 3.128416 2.724114 0.077666
C -3.463079 1.301481 -0.452033
C -3.442507 -0.929027 0.611901
C -4.797818 1.213391 -0.629349
C -4.779203 -1.022907 0.440357
C -5.589698 0.106203 -0.050118
H 0.026359 3.775561 -0.956453
H 4.168080 3.036542 0.087228
H -2.923088 2.157308 -0.830027
H -2.905773 -1.762039 1.035296
H -5.342182 1.997146 -1.145096
H -5.310514 -1.923898 0.725206
H -5.979762 0.550628 0.895036
Cu 2.338647 -2.607814 -0.824773
O 4.000847 -1.468636 -0.807470
H 4.778451 -2.002495 -0.616164
O 0.652110 -3.720116 -0.811686
H 0.222386 -3.715132 -1.673482
H 0.010096 -3.320011 -0.214220
H 3.952285 -0.822929 -0.082369
H 4.578510 1.094389 1.099762
H 0.032195 -1.999610 1.872409
O -6.666175 -0.342350 -0.840258
H -7.318068 0.363337 -0.901863

SCF Energy: -1378.78297265
Sum of electronic and zero-point Energies= -1378.507801
Sum of electronic and thermal Energies= -1378.482885
Sum of electronic and thermal Enthalpies= -1378.481941
Sum of electronic and thermal Free Energies= -1378.565260

==> ./AOX-II/PA/mono-/c4c5/RAF/c8a <==
O -1.639928 1.096683 -0.225128
O -0.954051 -2.225966 1.030639
O 2.992810 1.080971 0.430142
O 1.440969 -1.142351 1.123726
O 0.689648 4.973005 -1.004800
C 0.640412 1.030087 0.519404
C -0.637563 1.815875 0.479038
C -1.856857 -0.206742 0.129634
C 0.501897 -0.364040 0.805853

C	-0.828399	-0.927792	0.670092
C	-3.217807	-0.653393	-0.157620
C	1.818438	1.697727	0.251387
C	-0.566260	3.173648	-0.133083
C	0.628370	3.809552	-0.504727
C	1.796501	3.020235	-0.251610
C	-4.222703	0.305749	-0.370876
C	-3.558588	-2.013612	-0.249778
C	-5.524000	-0.082255	-0.652069
C	-4.861364	-2.392335	-0.541384
C	-5.849796	-1.432707	-0.739438
H	-1.509988	3.708339	-0.192540
H	2.756167	3.479954	-0.475963
H	-3.981342	1.358794	-0.305717
H	-2.805423	-2.774836	-0.108196
H	-6.286593	0.673597	-0.804474
H	-5.104396	-3.446599	-0.616023
H	-6.867193	-1.735217	-0.962292
Cu	3.008704	-1.405879	-0.160210
O	4.703981	-1.205359	-1.439909
H	5.346110	-1.891101	-1.229923
O	2.970150	-3.661589	-0.142451
H	3.111818	-4.003929	-1.030379
H	2.069185	-3.921684	0.074400
H	5.157127	-0.379191	-1.242946
H	3.723118	1.610944	0.082131
H	-0.072867	-2.507007	1.327675
O	-1.026194	1.999163	1.821378
H	-1.780891	2.601987	1.852609

SCF Energy: -1378.73321629
Sum of electronic and zero-point Energies= -1378.460319
Sum of electronic and thermal Energies= -1378.434689
Sum of electronic and thermal Enthalpies= -1378.433744
Sum of electronic and thermal Free Energies= -1378.515995

==> ./AOX-II/PA/mono-/c4c5/RAF/c4 <==

O	1.747776	1.290343	-0.005476
O	0.945863	-2.101073	0.129899
O	-3.019924	1.200776	0.561332
O	-1.450149	-1.121543	0.371060
O	-0.685462	5.273098	-0.042589
C	-0.667400	1.137975	0.311707
C	0.503744	1.915865	0.133148
C	1.979149	0.035440	0.130019
C	-0.555310	-0.257622	0.414101
C	0.855951	-0.840813	0.674782
C	3.313105	-0.384083	-0.173736
C	-1.885777	1.895004	0.358624
C	0.543846	3.262816	0.005549
C	-0.689114	4.020267	0.065949
C	-1.889506	3.262542	0.243559
C	4.185634	0.538934	-0.796010
C	3.789389	-1.673643	0.148473
C	5.482658	0.176053	-1.091491
C	5.095902	-2.018048	-0.140381
C	5.939773	-1.100780	-0.762560
H	1.483407	3.773158	-0.164678
H	-2.831998	3.797571	0.302934
H	3.828421	1.526845	-1.054606
H	3.146819	-2.389332	0.640281
H	6.143552	0.882995	-1.577899
H	5.460296	-3.004200	0.119748
H	6.961635	-1.380815	-0.992327
Cu	-3.263122	-1.158736	-0.533183
O	-5.049957	-1.568180	-1.435318
H	-5.393532	-2.418614	-1.142818
O	-2.138357	-4.131502	0.741734
H	-1.826353	-4.298974	1.634878
H	-2.104531	-3.172022	0.659461
H	-4.941162	-1.662938	-2.387332
H	-3.770577	1.806796	0.598451
H	0.051999	-2.478849	0.159643
O	0.982693	-0.823027	2.078575
H	1.750425	-1.348846	2.342426

SCF Energy: -1378.79957628
Sum of electronic and zero-point Energies= -1378.524759
Sum of electronic and thermal Energies= -1378.498859
Sum of electronic and thermal Enthalpies= -1378.497915
Sum of electronic and thermal Free Energies= -1378.582256

==> ./AOX-II/PA/mono-/c4c5/RAF/c2p <==

O	-2.052044	0.881697	0.121488
O	-0.484539	-2.331590	0.045506
O	2.670255	1.692656	0.141249
O	1.587173	-0.891456	0.034232
O	-0.435428	5.258723	0.190358
C	0.349239	1.185296	0.108393
C	-0.978902	1.715107	0.125589
C	-1.956611	-0.476701	0.083161
C	0.529908	-0.209594	0.069686
C	-0.694681	-1.038404	0.074208
C	-3.208196	-1.145193	0.054972
C	1.404552	2.164028	0.134539
C	-1.263675	3.051223	0.151433
C	-0.207457	4.017171	0.166720
C	1.137019	3.502194	0.158252

C	-4.464355	-0.308161	-0.014504
C	-3.340820	-2.529266	0.203165
C	-5.738157	-1.055239	-0.080389
C	-4.589027	-3.119721	0.238269
C	-5.799522	-2.391046	0.101078
H	-2.295671	3.381627	0.163909
H	1.960039	4.209949	0.178682
H	-4.506666	0.139891	1.005058
H	-2.469666	-3.157457	0.294259
H	-6.627008	-0.456046	-0.240422
H	-4.641780	-4.195428	0.363273
H	-6.746431	-2.914831	0.103521
Cu	3.544776	-0.693545	-0.243248
O	5.528216	-0.817420	-0.556164
H	5.891637	-1.645426	-0.176355
O	6.539895	-3.153457	0.485218
H	6.711656	-3.809906	-0.196363
H	5.910486	-3.579893	1.074217
H	5.991795	-0.104616	-0.106910
H	3.290124	2.431867	0.156416
H	0.491696	-2.437494	0.014176
O	-4.482808	0.702391	-0.992999
H	-3.800374	1.348027	-0.777785

SCF Energy: -1378.77862548
Sum of electronic and zero-point Energies= -1378.504091
Sum of electronic and thermal Energies= -1378.478773
Sum of electronic and thermal Enthalpies= -1378.477829
Sum of electronic and thermal Free Energies= -1378.562070

==> ./AOX-II/PA/mono-/c4c5/RAF/c5p <==

O	1.266302	1.580043	0.060298
O	0.974552	-1.939265	-0.610584
O	-3.401579	0.778753	-0.636166
O	-1.500808	-1.268817	-0.699173
O	-1.791114	5.098256	0.454684
C	-1.068479	1.066758	-0.269627
C	-0.027575	1.995053	0.016274
C	1.636288	0.294125	-0.154419
C	-0.732511	-0.293535	-0.468848
C	0.687303	-0.642816	-0.408461
C	3.087300	0.109853	-0.066996
C	-2.388325	1.622362	-0.321392
C	-0.237152	3.329772	0.260826
C	-1.555811	3.869636	0.231059
C	-2.616419	2.949409	-0.077036
C	3.912899	1.205676	0.269436
C	3.700691	-1.112236	-0.273384
C	5.301005	1.113578	0.381267
C	5.145714	-1.223646	-0.281565
C	5.926670	-0.088215	0.181333
H	0.606747	3.975096	0.475236
H	-3.631612	3.331855	-0.128640
H	3.448871	2.166606	0.450020
H	3.143544	-2.004208	-0.522274
H	5.872970	1.989184	0.658540
H	7.000871	-0.205000	0.268290
Cu	-3.362214	-1.764747	-0.166243
O	-5.103787	-2.588612	0.478543
H	-5.081450	-3.546636	0.385833
O	-0.915268	-2.661173	2.169318
H	-0.033472	-2.321165	1.993155
H	-1.414201	-2.411095	1.383685
H	-5.841983	-2.306031	-0.071446
H	-4.234231	1.264873	-0.624578
H	0.112098	-2.371923	-0.753493
H	5.205695	-0.915046	-1.387113
O	5.617542	-2.511327	-0.102945
H	6.554064	-2.546327	-0.328761

SCF Energy: -1378.76683915
Sum of electronic and zero-point Energies= -1378.495323
Sum of electronic and thermal Energies= -1378.469236
Sum of electronic and thermal Enthalpies= -1378.468292
Sum of electronic and thermal Free Energies= -1378.552180

==> ./AOX-II/PA/mono-/c4c5/RAF/c3 <==

O	1.685205	1.311320	0.047962
O	1.016607	-2.112326	-0.076679
O	-3.093240	0.988430	0.393335
O	-1.420120	-1.250998	0.173751
O	-0.912865	5.184741	0.234717
C	-0.730980	1.039285	0.266113
C	0.412454	1.875298	0.192727
C	1.961306	0.058524	0.092372
C	-0.562814	-0.351958	0.286836
C	0.863229	-0.895366	0.548159
C	3.312406	-0.290965	-0.224154
C	-1.982679	1.742250	0.309463
C	0.399416	3.227926	0.170548
C	-0.867137	3.929079	0.240415
C	-2.040961	3.112737	0.302253
C	4.153445	0.701419	-0.779690
C	3.833512	-1.582062	0.013455
C	5.462551	0.404190	-1.094508
C	5.151810	-1.860236	-0.291966
C	5.963713	-0.874926	-0.849483
H	1.321477	3.787317	0.075761

H	-3.008274	3.602355	0.355562
H	3.762410	1.690968	-0.975681
H	3.216192	-2.351751	0.452561
H	6.098505	1.163757	-1.532386
H	5.550147	-2.848193	-0.096379
H	6.994710	-1.103693	-1.094764
Cu	-3.234071	-1.303266	-0.722740
O	-5.016064	-1.730527	-1.625941
H	-4.978528	-2.570982	-2.093898
O	-1.637773	-3.387689	2.179716
H	-0.982420	-3.154706	2.842397
H	-1.554988	-2.686775	1.518999
H	-5.219076	-1.079213	-2.305274
H	-3.874109	1.555697	0.406177
H	0.131750	-2.507750	-0.132454
O	0.952225	-0.963994	1.952648
H	1.762833	-1.425161	2.207519

SCF Energy: -1378.80051242
Sum of electronic and zero-point Energies= -1378.525578
Sum of electronic and thermal Energies= -1378.500037
Sum of electronic and thermal Enthalpies= -1378.499093
Sum of electronic and thermal Free Energies= -1378.581907

==> ./AOX-II/PA/mono-/c4c5/RAF/c6p <==

O	1.537002	1.572895	0.123863
O	0.926432	-1.930247	-0.163227
O	-3.203335	1.133346	-0.420285
O	-1.511224	-1.054817	-0.381326
O	-1.196605	5.354227	0.315204
C	-0.846419	1.243028	-0.120030
C	0.283640	2.093953	0.075340
C	1.794152	0.240508	0.047969
C	-0.659291	-0.152271	-0.207516
C	0.732203	-0.637664	-0.086726
C	3.179067	-0.077814	-0.005616
C	-2.119352	1.909712	-0.194993
C	0.194928	3.451324	0.220597
C	-1.077206	4.103311	0.175451
C	-2.224764	3.263297	-0.047486
C	4.106270	0.942666	-0.243565
C	3.679535	-1.495571	0.110123
C	5.450308	0.664990	-0.382531
C	5.148737	-1.665586	0.021418
C	5.983662	-0.647028	-0.269009
H	1.094532	4.036949	0.367821
H	-3.202299	3.732315	-0.107049
H	3.767616	1.965268	-0.329147
H	6.129254	1.487724	-0.576906
H	5.509267	-2.676407	0.172776
H	7.049343	-0.806139	-0.370602
Cu	-3.408789	-1.471070	0.014072
O	-5.197950	-2.330182	0.449132
H	-5.530214	-2.826161	-0.306370
O	-0.938393	-3.879412	-0.409191
H	-0.778235	-4.548008	0.263329
H	-1.808504	-3.522321	-0.198488
H	-5.871458	-1.663553	0.619819
H	-3.994010	1.685645	-0.431718
H	0.081085	-2.450127	-0.271372
H	3.326207	-1.982096	-0.825478
O	3.211937	-2.204277	1.232668
H	2.306555	-2.485467	1.050020

SCF Energy: -1378.77856812
Sum of electronic and zero-point Energies= -1378.504364
Sum of electronic and thermal Energies= -1378.479305
Sum of electronic and thermal Enthalpies= -1378.478361
Sum of electronic and thermal Free Energies= -1378.559573

==> ./AOX-II/PA/mono-/c4c5/RAF/c6 <==

O	1.745448	1.352229	-0.077790
O	1.341325	-2.192559	0.472341
O	-2.897312	0.803550	0.689874
O	-1.149802	-1.388836	0.547790
O	-1.078602	5.039067	0.116995
C	-0.594614	0.919671	0.197302
C	0.494410	1.836911	-0.021609
C	2.068720	0.034691	0.096614
C	-0.309180	-0.480134	0.376280
C	1.086199	-0.875331	0.330327
C	3.504131	-0.206952	-0.045594
C	-1.857903	1.477331	0.294913
C	0.382425	3.200921	-0.116975
C	-0.886009	3.826571	0.007688
C	-2.091875	2.895299	-0.107080
C	4.279309	0.666438	-0.823368
C	4.131265	-1.289531	0.587223
C	5.641090	0.450730	-0.976372
C	5.496655	-1.491930	0.436991
C	6.255142	-0.628704	-0.347499
H	1.277928	3.807220	-0.167481
H	-2.223012	2.814011	-1.202792
H	3.811411	1.509044	-1.317296
H	3.559145	-1.962028	1.211267
H	6.224731	1.129000	-1.589012
H	5.969979	-2.327768	0.940260
H	7.320489	-0.794194	-0.465712

Cu	-2.909306	-1.826675	-0.310707
O	-4.647406	-2.485029	-1.131013
H	-4.524917	-3.337814	-1.561252
O	-1.133493	-4.755199	0.487362
H	-0.587993	-4.646081	1.271247
H	-1.532701	-3.888149	0.361519
H	-4.944124	-1.895936	-1.832528
H	-3.658622	1.415592	0.737985
H	0.479152	-2.632273	0.556411
O	-3.264646	3.353971	0.498826
H	-3.724269	3.949504	-0.100227

SCF Energy: -1378.82337396
Sum of electronic and zero-point Energies= -1378.547955
Sum of electronic and thermal Energies= -1378.522409
Sum of electronic and thermal Enthalpies= -1378.521465
Sum of electronic and thermal Free Energies= -1378.604444

==> ./AOX-II/PA/mono-/c4c5/RAF/c1p <==

O	-1.501273	1.575549	0.180688
O	-0.893298	-1.909994	-0.269452
O	3.200977	1.167865	-0.640687
O	1.530662	-1.024785	-0.531780
O	1.233590	5.355510	0.348980
C	0.867739	1.260583	-0.191207
C	-0.253198	2.101549	0.086614
C	-1.767435	0.247603	0.054170
C	0.681268	-0.130881	-0.303822
C	-0.706458	-0.629800	-0.150652
C	-3.145216	-0.066492	0.016316
C	2.134802	1.933589	-0.320353
C	-0.157963	3.452838	0.270919
C	1.110424	4.109813	0.178047
C	2.245932	3.282059	-0.135012
C	-4.088572	0.956983	-0.155734
C	-3.624627	-1.493187	0.128269
C	-5.426030	0.665407	-0.298532
C	-5.092653	-1.684689	-0.019069
C	-5.939698	-0.664175	-0.252471
H	-1.048886	4.032391	0.481315
H	3.216269	3.757902	-0.238884
H	-3.762256	1.986380	-0.198998
H	-3.175770	-2.037207	-0.718124
H	-6.120779	1.484478	-0.447811
H	-5.441058	-2.707049	0.074895
H	-7.003214	-0.830519	-0.368312
Cu	3.349152	-1.545001	0.071597
O	5.090489	-2.357174	0.717734
H	4.918911	-3.019176	1.395638
O	0.938969	-3.904235	-0.551907
H	0.839651	-4.401038	-1.369425
H	1.829676	-3.539627	-0.596684
H	5.642682	-1.698349	1.151619
H	3.986245	1.725004	-0.698669
H	-0.046420	-2.403738	-0.446766
O	-3.148980	-2.133612	1.296975
H	-3.599428	-1.744608	2.054583

SCF Energy: -1378.77628892
Sum of electronic and zero-point Energies= -1378.501887
Sum of electronic and thermal Energies= -1378.476671
Sum of electronic and thermal Enthalpies= -1378.475727
Sum of electronic and thermal Free Energies= -1378.557175

==> ./AOX-II/PA/mono-/c4c5/RAF/c2 <==

O	1.698323	1.433419	-0.172150
O	1.044342	-1.774289	1.115102
O	-3.012871	1.199069	0.729442
O	-1.369878	-1.020632	0.924638
O	-0.980692	5.219593	-0.697655
C	-0.680071	1.239131	0.337529
C	0.470281	2.018189	-0.047776
C	2.059480	0.343968	0.650868
C	-0.526787	-0.117313	0.653192
C	0.861934	-0.571152	0.784088
C	3.232382	-0.353282	-0.014451
C	-1.951912	1.931251	0.348394
C	0.379712	3.326072	-0.388675
C	-0.896293	4.011412	-0.384533
C	-2.052128	3.244896	0.001377
C	3.304887	-0.430670	-1.405715
C	4.213635	-0.962090	0.766277
C	4.367013	-1.093213	-2.008826
C	5.275791	-1.621223	0.157384
C	5.355037	-1.687113	-1.229417
H	1.266277	3.871759	-0.687556
H	-3.016765	3.740552	0.029475
H	2.539581	0.031619	-2.017512
H	4.155690	-0.919156	1.847631
H	4.422710	-1.142872	-3.090616
H	6.041038	-2.083639	0.770924
H	6.184750	-2.200881	-1.702592
Cu	-2.919196	-1.627180	-0.241276
O	-4.521634	-1.770850	-1.602182
H	-4.480687	-2.614583	-2.063629
O	-2.869787	-3.880723	0.388479
H	-3.770144	-4.170802	0.562083
H	-2.433800	-3.930476	1.244461

H	-4.461767	-1.110174	-2.299365
H	-3.807777	1.746913	0.716506
H	0.141047	-2.185582	1.194684
O	2.294106	0.753146	1.960862
H	3.143490	1.212493	2.001924

SCF Energy: -1378.79175591
Sum of electronic and zero-point Energies= -1378.518839
Sum of electronic and thermal Energies= -1378.492682
Sum of electronic and thermal Enthalpies= -1378.491738
Sum of electronic and thermal Free Energies= -1378.577227

==> ./AOX-II/PA/mono-/c4c5/RAF/c8 <==

O	1.901984	0.836468	-0.140800
O	0.516239	-2.425092	0.393584
O	-2.728093	1.559592	0.386649
O	-1.623905	-0.977923	0.464014
O	0.328176	5.133171	0.268716
C	-0.434812	1.096999	0.108880
C	0.827315	1.588061	-0.113060
C	1.872405	-0.514652	0.052418
C	-0.575422	-0.327397	0.277918
C	0.656774	-1.101153	0.241035
C	3.197397	-1.128844	-0.008395
C	-1.518748	2.076580	0.214531
C	1.088010	3.017491	-0.441223
C	0.008845	3.961373	0.064423
C	-1.303283	3.427102	0.198104
C	4.241020	-0.465150	-0.670676
C	3.456525	-2.366857	0.596599
C	5.505443	-1.032170	-0.734385
C	4.726806	-2.923962	0.532337
C	5.753669	-2.263534	-0.134454
H	-2.127840	4.103154	0.394818
H	4.059703	0.492659	-1.143049
H	2.673727	-2.887781	1.130144
H	6.299979	-0.510053	-1.255928
H	4.914085	-3.878520	1.011716
H	6.743283	-2.704402	-0.184605
Cu	-3.598509	-1.144792	-0.061927
O	-5.624993	-0.987442	-0.481172
H	-5.932253	-1.797125	-0.901576
O	-3.207301	-3.659333	-0.425548
H	-3.910104	-4.219171	-0.083706
H	-2.501472	-3.747698	0.221588
H	-5.791296	-0.297778	-1.131674
H	-3.389106	2.261012	0.465040
H	-0.440785	-2.579246	0.491353
H	0.963072	3.066685	-1.540996
O	2.373161	3.427724	-0.076138
H	2.301950	4.376603	0.103995

SCF Energy: -1378.82799509
Sum of electronic and zero-point Energies= -1378.552947
Sum of electronic and thermal Energies= -1378.526968
Sum of electronic and thermal Enthalpies= -1378.526024
Sum of electronic and thermal Free Energies= -1378.610305

==> ./AOX-II/PA/mono-/c4c5/RAF/c4a <==

O	-1.781254	1.250881	0.416144
O	-0.736132	-1.721705	-1.366356
O	2.968750	1.716844	0.140771
O	1.505831	-0.227829	-1.364464
O	0.122978	5.397434	-0.618941
C	0.631889	1.359794	0.281198
C	-0.698565	2.046515	0.187061
C	-1.808023	-0.020174	-0.087245
C	0.559111	0.165262	-0.702443
C	-0.704628	-0.536081	-0.702127
C	-3.118181	-0.649378	0.061412
C	1.779064	2.303020	-0.001819
C	-0.871135	3.340341	-0.085041
C	0.285482	4.202496	-0.356274
C	1.604617	3.603399	-0.306121
C	-4.264763	0.152342	0.168845
C	-3.254658	-2.043869	0.124743
C	-5.515531	-0.428141	0.318540
C	-4.508684	-2.615961	0.285488
C	-5.641936	-1.813102	0.377736
H	-1.862854	3.772333	-0.138994
H	2.461296	4.245207	-0.479791
H	-4.174457	1.230421	0.123303
H	-2.381652	-2.679814	0.068321
H	-6.394284	0.203097	0.389759
H	-4.599772	-3.694889	0.343453
H	-6.620335	-2.265289	0.498337
Cu	3.058728	-2.350230	0.246250
O	3.743344	-0.801778	1.340951
H	3.323138	-0.811785	2.206942
O	2.368680	-3.898598	-0.854029
H	3.073384	-4.285604	-1.384216
H	1.723392	-3.582842	-1.495836
H	3.446509	0.028574	0.935978
H	3.687291	2.350312	0.009086
H	0.140334	-1.844784	-1.760666
O	0.794777	0.744181	1.546304
H	0.734756	1.417526	2.236321

SCF Energy: -1378.80126075
Sum of electronic and zero-point Energies= -1378.525574
Sum of electronic and thermal Energies= -1378.500461
Sum of electronic and thermal Enthalpies= -1378.499517
Sum of electronic and thermal Free Energies= -1378.581759

==> ./AOX-II/PA/mono-/c4c5/RAF/c5 <==

O	1.909342	1.157564	0.146057
O	1.339455	-2.313961	-0.552849
O	-2.199412	1.278446	-1.728051
O	-1.107792	-1.378131	-0.734075
O	-0.747979	4.993509	0.550739
C	-0.404447	0.859600	-0.287151
C	0.669215	1.657341	0.039267
C	2.182315	-0.155475	-0.043801
C	-0.213945	-0.532472	-0.470719
C	1.150265	-1.007108	-0.342304
C	3.605562	-0.469402	0.070020
C	-1.753337	1.516346	-0.418940
C	0.564090	3.036835	0.301397
C	-0.666886	3.757013	0.278735
C	-1.771052	2.974897	-0.122443
C	4.554740	0.527688	-0.201030
C	4.047908	-1.742389	0.456432
C	5.910338	0.252056	-0.101010
C	5.406819	-2.006615	0.562235
C	6.341701	-1.015449	0.280542
H	1.478071	3.571710	0.538362
H	-2.742037	3.452813	-0.233864
H	4.228611	1.515801	-0.502320
H	3.335253	-2.520422	0.692862
H	6.632380	1.029896	-0.323278
H	5.735040	-2.992837	0.871362
H	7.402111	-1.228780	0.359839
Cu	-3.009062	-1.362087	0.164127
O	-4.925294	-1.039784	1.100399
H	-5.550136	-1.727326	0.849208
O	-3.324074	-3.584272	-0.121222
H	-4.028364	-3.860870	0.473083
H	-2.544473	-4.048030	0.199470
H	-5.309092	-0.224353	0.762630
H	-2.937338	1.866809	-1.929757
H	0.462402	-2.683076	-0.760779
O	-2.593110	0.896910	0.546791
H	-3.448447	1.349762	0.573802

SCF Energy: -1378.75393545
Sum of electronic and zero-point Energies= -1378.480508
Sum of electronic and thermal Energies= -1378.455009
Sum of electronic and thermal Enthalpies= -1378.454065
Sum of electronic and thermal Free Energies= -1378.535855

==> ./AOX-II/PA/mono-/c4c5/aIP <==

O	-1.884414	1.202932	-0.074326
O	-1.105907	-2.262487	0.101802
O	2.893187	0.976992	0.075068
O	1.267565	-1.309794	0.090449
O	0.635805	5.107185	-0.198230
C	0.510836	0.992362	-0.006574
C	-0.660375	1.771247	-0.064513
C	-2.085638	-0.114636	-0.027807
C	0.365708	-0.420639	0.033977
C	-0.975921	-0.945224	0.026316
C	-3.488323	-0.503422	-0.006669
C	1.768992	1.719368	0.004004
C	-0.648779	3.150648	-0.130185
C	0.617149	3.866324	-0.132452
C	1.818644	3.074425	-0.054706
C	-4.451036	0.436472	0.399519
C	-3.907788	-1.787263	-0.392152
C	-5.792323	0.092821	0.435255
C	-5.254545	-2.116774	-0.362356
C	-6.198729	-1.184357	0.055673
H	-1.577010	3.704725	-0.181472
H	2.774635	3.585459	-0.049136
H	-4.142675	1.429655	0.700968
H	-3.191411	-2.519737	-0.733469
H	-6.523947	0.822469	0.762700
H	-5.566366	-3.108531	-0.669675
H	-7.249388	-1.451133	0.084431
H	-0.207294	-2.637372	0.139916
Cu	3.205712	-1.212226	0.083144
O	5.211579	-1.049919	0.047670
H	5.614983	-1.849351	0.406800
O	3.193994	-3.297974	-0.152032
H	4.019952	-3.600670	-0.546045
H	2.508065	-3.535101	-0.786554
H	5.486843	-0.337720	0.638162
H	3.686271	1.528266	0.035020

SCF Energy: -1302.80874854
Sum of electronic and zero-point Energies= -1302.547614
Sum of electronic and thermal Energies= -1302.525281
Sum of electronic and thermal Enthalpies= -1302.524336

Sum of electronic and thermal Free Energies= -1302.598751

==> ./AOX-II/PA/mono-/c4c5/HAT/c3 <==

O	-1.862695	1.199252	-0.079363
O	-1.169580	-1.885970	1.374361
O	2.864323	1.192751	0.745956
O	1.360434	-1.111496	0.883002
O	0.590601	5.124779	-0.581394
C	0.536152	1.078498	0.353634
C	-0.621350	1.820750	0.011284
C	-2.091123	-0.033030	0.223058
C	0.442648	-0.302666	0.645158
C	-0.958349	-0.849867	0.788183
C	-3.412533	-0.513889	-0.001880
C	1.754310	1.849975	0.375290
C	-0.643472	3.139038	-0.306423
C	0.591419	3.902096	-0.298078
C	1.775746	3.183467	0.053036
C	-4.446047	0.421555	-0.252728
C	-3.710664	-1.896425	-0.005923
C	-5.734308	-0.015115	-0.467794
C	-5.004631	-2.317544	-0.242524
C	-6.015109	-1.383914	-0.463924
H	-1.574177	3.624687	-0.571407
H	2.715752	3.724568	0.091665
H	-4.225039	1.480813	-0.245500
H	-2.929591	-2.625951	0.144311
H	-6.527518	0.702287	-0.638289
H	-5.229314	-3.376820	-0.259891
H	-7.029803	-1.722833	-0.639808
Cu	3.131416	-1.375958	-0.091423
O	5.158319	-1.206671	-0.742867
H	5.470730	-2.080419	-0.998921
O	2.869270	-3.542736	-0.905540
H	3.692827	-3.750036	-1.357416
H	2.203381	-3.577827	-1.598737
H	5.737780	-0.943629	-0.021157
H	3.622376	1.791108	0.730942

SCF Energy: -1302.35399178
Sum of electronic and zero-point Energies= -1302.108094
Sum of electronic and thermal Energies= -1302.083720
Sum of electronic and thermal Enthalpies= -1302.082776
Sum of electronic and thermal Free Energies= -1302.162995

==> ./AOX-II/PA/mono-/c4c5/HAT/h2o <==

O	1.754308	1.248226	0.043265
O	1.218903	-2.216265	0.562376
O	-2.907953	0.737901	0.810727
O	-1.245132	-1.407025	0.620014
O	-1.063856	4.945828	-0.352031
C	-0.601788	0.899640	0.363574
C	0.489077	1.746418	0.096996
C	2.050078	-0.028441	0.204225
C	-0.383460	-0.496019	0.473162
C	0.991301	-0.935204	0.430069
C	3.444585	-0.353791	0.022351
C	-1.888092	1.540510	0.443072
C	0.363626	3.090902	-0.142110
C	-0.943390	3.718351	-0.125701
C	-2.057400	2.869520	0.176372
C	4.256851	0.532964	-0.713805
C	4.016766	-1.516688	0.573397
C	5.596146	0.253011	-0.904088
C	5.363687	-1.776424	0.388334
C	6.153344	-0.901124	-0.353417
H	1.239194	3.686064	-0.370407
H	-3.045632	3.310809	0.242019
H	3.823195	1.423278	-1.150674
H	3.421026	-2.195128	1.165996
H	6.210139	0.930489	-1.485594
H	5.801621	-2.664785	0.827646
H	7.205237	-1.117179	-0.503051
H	0.348611	-2.660544	0.625961
Cu	-3.126404	-1.276334	-0.141152
O	-4.978960	-1.082571	-0.479611
H	-5.243752	-1.826950	-1.026415
O	-2.838337	-3.178121	-1.082589
H	-3.684878	-3.638927	-1.080433
H	-2.276146	-3.690359	-0.490117
H	-3.752668	1.211383	0.766662

SCF Energy: -1302.29925176
Sum of electronic and zero-point Energies= -1302.051136
Sum of electronic and thermal Energies= -1302.028982
Sum of electronic and thermal Enthalpies= -1302.028037
Sum of electronic and thermal Free Energies= -1302.102121

==> ./AOX-II/PA/mono-/c4c5/HAT/c5 <==

O	-1.826560	1.188232	-0.072835
O	-1.346939	-2.334841	-0.285018
O	2.830976	0.654045	-0.749181
O	1.146718	-1.543925	-0.348057
O	1.006285	4.899320	0.077169
C	0.512105	0.769928	-0.307441
C	-0.560075	1.640472	-0.151268
C	-2.135791	-0.109536	-0.129445

C	0.285179	-0.629451	-0.298685
C	-1.104593	-1.037286	-0.246656
C	-3.554800	-0.385323	0.029030
C	1.844184	1.377091	-0.417793
C	-0.401566	3.017090	-0.034908
C	0.900083	3.647358	-0.055617
C	1.996939	2.766261	-0.214709
C	-4.359784	0.546132	0.707252
C	-4.143663	-1.546322	-0.497002
C	-5.715316	0.312686	0.865238
C	-5.504701	-1.763748	-0.343113
C	-6.292106	-0.841922	0.340366
H	-1.282009	3.631622	0.116998
H	2.999340	3.173822	-0.283963
H	-3.915457	1.442028	1.122712
H	-3.548693	-2.263199	-1.044929
H	-6.324570	1.031636	1.400962
H	-5.952922	-2.656571	-0.763722
H	-7.354416	-1.021530	0.463519
Cu	3.196685	-1.113984	0.078539
O	5.110293	-0.553275	0.856616
H	5.338304	-1.164373	1.565341
O	3.652257	-3.174310	0.375098
H	4.534221	-3.345383	0.028006
H	3.064175	-3.706751	-0.171087
H	5.038377	0.306742	1.284152
H	-0.481009	-2.785058	-0.297462

SCF Energy: -1302.33741798
Sum of electronic and zero-point Energies= -1302.090590
Sum of electronic and thermal Energies= -1302.067561
Sum of electronic and thermal Enthalpies= -1302.066617
Sum of electronic and thermal Free Energies= -1302.142790

==> ./AOX-II/PA/mono-/c4c5/aEA <==

O	-1.928108	1.206608	-0.112148
O	-0.860079	-2.211883	0.263245
O	2.829555	1.400253	0.378156
O	1.419104	-1.016777	0.332787
O	0.270068	5.335699	-0.273015
C	0.472618	1.193937	0.109415
C	-0.754846	1.889760	-0.060519
C	-2.005628	-0.145134	-0.009283
C	0.444607	-0.224180	0.194760
C	-0.861923	-0.864407	0.135993
C	-3.386908	-0.636767	-0.039035
C	1.638648	2.020494	0.173894
C	-0.851771	3.256414	-0.187935
C	0.315701	4.068883	-0.148546
C	1.562374	3.382348	0.047030
C	-4.431393	0.212661	0.355446
C	-3.699966	-1.935660	-0.462921
C	-5.746770	-0.229972	0.339161
C	-5.019609	-2.369204	-0.483627
C	-6.047369	-1.522821	-0.079788
H	-1.825667	3.713549	-0.320464
H	2.473152	3.971891	0.100874
H	-4.210635	1.220730	0.684553
H	-2.916355	-2.603865	-0.792631
H	-6.539910	0.438629	0.656033
H	-5.244245	-3.374873	-0.822216
H	-7.075904	-1.866841	-0.093983
H	0.076653	-2.451138	0.376485
Cu	3.386955	-0.989970	-0.023054
O	5.257262	-1.567081	-0.567809
H	5.301584	-2.527973	-0.615177
O	2.709338	-4.338455	0.164328
H	3.173328	-3.498085	0.083912
H	1.781500	-4.088397	0.137678
H	5.903733	-1.324494	0.103418
H	3.537350	2.054972	0.381466

SCF Energy: -1303.17204671
Sum of electronic and zero-point Energies= -1302.913802
Sum of electronic and thermal Energies= -1302.889653
Sum of electronic and thermal Enthalpies= -1302.888709
Sum of electronic and thermal Free Energies= -1302.968611

==> ./AOX-II/PA/mono-/c4c5 <==

O	-1.868424	1.223047	-0.080186
O	-1.132169	-2.292499	0.099043
O	2.913358	0.927220	0.081483
O	1.250473	-1.334284	0.097593
O	0.733412	5.114283	-0.216241
C	0.524762	0.969239	-0.006873
C	-0.635610	1.790975	-0.073214
C	-2.072818	-0.114766	-0.031635
C	0.352341	-0.430608	0.036302
C	-0.997832	-0.948983	0.023325
C	-3.491534	-0.481308	-0.009360
C	1.769445	1.670519	0.001864
C	-0.597814	3.162656	-0.141235
C	0.649099	3.850211	-0.144615
C	1.832329	3.031492	-0.061456
C	-4.436962	0.442183	0.460880
C	-3.935946	-1.735108	-0.453072
C	-5.784597	0.113061	0.500872
C	-5.286986	-2.054077	-0.416194

C	-6.215647	-1.136448	0.064392
H	-1.524364	3.721902	-0.196868
H	2.800617	3.522828	-0.053877
H	-4.112694	1.416623	0.805278
H	-3.231145	-2.457173	-0.840722
H	-6.500827	0.835539	0.876607
H	-5.613871	-3.026296	-0.768845
H	-7.269149	-1.392422	0.096440
H	-0.232137	-2.651875	0.153239
Cu	3.179694	-1.213572	0.086134
O	5.201336	-1.051490	0.066092
H	5.600227	-1.849869	0.431780
O	3.175668	-3.299768	-0.153313
H	3.997540	-3.601301	-0.556445
H	2.483790	-3.535835	-0.781666
H	5.469150	-0.339190	0.659443
H	3.699113	1.484339	0.022090

SCF Energy: -1303.00072766
Sum of electronic and zero-point Energies= -1302.739758
Sum of electronic and thermal Energies= -1302.717336
Sum of electronic and thermal Enthalpies= -1302.716392
Sum of electronic and thermal Free Energies= -1302.790965

==> ./AOX-II/PA/bis-/c3c4/aEA <==

O	-5.609523	0.221157	0.052531
O	-2.405916	-1.480363	-0.149709
O	-2.260936	3.666256	-0.016503
O	-1.644083	1.110859	-0.087658
O	-6.844697	4.752453	0.107122
C	-3.866937	1.865440	0.011883
C	-5.241308	1.532821	0.043948
C	-4.701507	-0.783681	0.021573
C	-2.884166	0.842942	-0.040020
C	-3.366242	-0.524457	-0.049570
C	-5.335612	-2.111141	0.025258
C	-3.554816	3.262366	0.014639
C	-6.250310	2.465150	0.077257
C	-5.937481	3.857573	0.078987
C	-4.545313	4.208889	0.047796
C	-6.460578	-2.342508	-0.776181
C	-4.850333	-3.144425	0.835185
C	-7.073925	-3.587582	-0.778382
C	-5.471008	-4.388223	0.830714
C	-6.580192	-4.614028	0.022824
H	-7.284607	2.141886	0.103427
H	-4.272774	5.259230	0.048768
H	-6.846462	-1.546811	-1.403232
H	-4.003135	-2.971156	1.490180
H	-7.939809	-3.757916	-1.408754
H	-5.090773	-5.178039	1.469092
H	-7.062392	-5.585502	0.020334
H	-2.792925	-2.338721	-0.355410
H	-1.703528	2.862657	-0.044485
Cu	-0.000346	-0.000456	-0.093409
O	1.643863	-1.110477	-0.086892
C	2.883991	-0.842577	-0.040309
C	3.866661	-1.865177	0.011664
C	3.366261	0.524716	-0.050770
C	5.241072	-1.532740	0.043886
C	3.554360	-3.262054	0.014567
O	2.406218	1.480948	-0.152065
C	4.701520	0.783846	0.021236
O	5.609377	-0.221118	0.053104
C	6.249972	-2.465174	0.077165
O	2.260449	-3.665805	-0.016704
C	4.544747	-4.208694	0.047785
H	2.794119	2.339379	-0.355728
C	5.335972	2.111135	0.025235
C	5.936960	-3.857564	0.079072
H	7.284301	-2.142052	0.103698
H	1.703124	-2.862151	-0.044355
H	4.272086	-5.259003	0.048875
C	6.461614	2.341983	-0.775420
C	4.850522	3.144781	0.834613
O	6.844047	-4.752568	0.107199
C	7.075426	3.586826	-0.777438
H	6.847657	1.546062	-1.402081
C	5.471634	4.388356	0.830308
H	4.002847	2.972044	1.489152
C	6.581489	4.613626	0.023177
H	7.941852	3.756669	-1.407198
H	5.091191	5.178424	1.468251
H	7.064021	5.584934	0.020825

SCF Energy: -2103.24394565
Sum of electronic and zero-point Energies= -2102.825341
Sum of electronic and thermal Energies= -2102.790573
Sum of electronic and thermal Enthalpies= -2102.789629
Sum of electronic and thermal Free Energies= -2102.896200

==> ./AOX-II/PA/bis-/c3c4/HAT/c5 <==

O	-5.466243	0.076127	-0.120814
O	-2.210538	-1.275796	0.521464
O	-2.693353	3.880511	0.544888
O	-1.655798	1.291703	0.607900
O	-7.214145	4.343775	-0.671052
C	-3.917100	1.878909	0.181794

C	-5.184881	1.392253	-0.083485
C	-4.530472	-0.845954	0.093105
C	-2.841882	0.963917	0.389606
C	-3.219782	-0.443954	0.335630
C	-5.019482	-2.214968	-0.009769
C	-3.772123	3.381961	0.214094
C	-6.266102	2.220483	-0.380416
C	-6.178588	3.676538	-0.402817
C	-4.907878	4.190558	-0.117158
C	-5.960152	-2.529290	-1.002312
C	-4.587728	-3.211417	0.877848
C	-6.437609	-3.824481	-1.116797
C	-5.080507	-4.503188	0.759708
C	-5.997953	-4.813329	-0.239036
H	-7.214654	1.754266	-0.627001
H	-4.757128	5.263923	-0.095923
H	-6.295036	-1.761196	-1.689246
H	-3.911774	-2.973265	1.691670
H	-7.153199	-4.065495	-1.894351
H	-4.754605	-5.265216	1.457969
H	-6.376177	-5.825294	-0.330848
H	-2.441347	-2.202613	0.363190
Cu	-0.023900	0.189569	0.267648
O	1.540082	-1.028695	0.015820
C	2.786685	-0.780985	-0.014689
C	3.754625	-1.816375	-0.036209
C	3.283257	0.580022	-0.046396
C	5.132872	-1.500150	-0.086213
C	3.424785	-3.209395	-0.025463
O	2.325199	1.543290	-0.079901
C	4.623211	0.823840	-0.057212
O	5.516971	-0.193229	-0.089693
C	6.129530	-2.444865	-0.121885
O	2.126010	-3.596086	0.016424
C	4.403102	-4.167967	-0.060185
H	2.696196	2.398073	-0.325456
C	5.274322	2.142158	-0.083876
C	5.799368	-3.833561	-0.109502
H	7.167703	-2.135045	-0.154693
H	1.578193	-2.785950	0.028735
H	4.118128	-5.214921	-0.050906
C	6.360358	2.360455	-0.940771
C	4.844094	3.179748	0.751320
O	6.695037	-4.739050	-0.139372
C	6.988732	3.597548	-0.972421
H	6.703940	1.561279	-1.587621
C	5.479682	4.415414	0.716844
H	4.028638	3.016697	1.447607
C	6.549244	4.628732	-0.146094
H	7.823509	3.757867	-1.645881
H	5.142250	5.208984	1.374316
H	7.042930	5.594049	-0.172137

SCF Energy: -2102.38641046
Sum of electronic and zero-point Energies= -2101.980332
Sum of electronic and thermal Energies= -2101.945476
Sum of electronic and thermal Enthalpies= -2101.944532
Sum of electronic and thermal Free Energies= -2102.052400

==> ./AOX-II/PA/bis-/c3c4/HAT/c3 <==

O	5.563537	-0.021620	-0.030544
O	2.379456	1.269135	-0.614887
O	2.598973	-3.831764	-0.114564
O	1.685894	-1.342823	-0.086371
O	7.264084	-4.363961	-0.190841
C	3.981093	-1.867155	-0.104242
C	5.313567	-1.386894	-0.106533
C	4.645488	0.887100	-0.031143
C	2.892968	-0.988383	-0.115258
C	3.215302	0.468771	-0.265756
C	5.073251	2.229796	0.158470
C	3.829920	-3.306512	-0.120456
C	6.411092	-2.175014	-0.128384
C	6.253751	-3.628024	-0.159794
C	4.916302	-4.137758	-0.147781
C	6.452480	2.531663	0.041241
C	4.161859	3.257707	0.498602
C	6.893318	3.822608	0.226913
C	4.624852	4.542385	0.701832
C	5.981372	4.828362	0.558423
H	7.404873	-1.745964	-0.115256
H	4.766747	-5.210811	-0.163885
H	7.152345	1.750165	-0.223769
H	3.112905	3.040369	0.632099
H	7.944807	4.056875	0.115706
H	3.930290	5.326076	0.977531
H	6.334417	5.842110	0.710090
H	1.942579	-3.105933	-0.093768
Cu	0.042544	-0.206064	-0.108746
O	-1.565952	0.962266	-0.099205
C	-2.816324	0.747990	-0.048804
C	-3.757039	1.810068	-0.075069
C	-3.352868	-0.596592	0.027042
C	-5.143895	1.534204	-0.037714
C	-3.390024	3.191130	-0.161184
O	-2.430915	-1.594429	-0.001143
C	-4.697827	-0.797671	0.103006
O	-5.564584	0.242003	0.056451
C	-6.115251	2.504914	-0.079997
O	-2.081001	3.541853	-0.201407
C	-4.342647	4.175586	-0.204389
H	-2.852274	-2.450583	-0.137325

C	-5.384190	-2.095079	0.194008
C	-5.747921	3.881281	-0.165637
H	-7.161740	2.224753	-0.043563
H	-1.555279	2.717533	-0.172464
H	-4.028603	5.212245	-0.268810
C	-6.518635	-2.333735	-0.591711
C	-4.940761	-3.089973	1.073226
O	-6.620349	4.809593	-0.203919
C	-7.183090	-3.549506	-0.510103
H	-6.872588	-1.566677	-1.271058
C	-5.612421	-4.304488	1.152138
H	-4.087105	-2.907760	1.717273
C	-6.731065	-4.538657	0.359774
H	-8.056426	-3.726154	-1.128336
H	-5.264427	-5.064121	1.843336
H	-7.253385	-5.487041	0.422961

SCF Energy: -2102.43377767
Sum of electronic and zero-point Energies= -2102.026310
Sum of electronic and thermal Energies= -2101.991679
Sum of electronic and thermal Enthalpies= -2101.990734
Sum of electronic and thermal Free Energies= -2102.097960

==> ./AOX-II/PA/bis-/c3c4/aIP <==

O	-5.221091	-0.117863	0.004749
O	-1.791717	-1.265601	0.081232
O	-2.430071	3.803418	-0.135138
O	-1.430163	1.329901	-0.078827
O	-7.114797	4.153921	-0.204230
C	-3.752662	1.784651	-0.058143
C	-5.051841	1.231681	-0.051945
C	-4.192197	-0.973863	0.074966
C	-2.640232	0.912362	-0.029233
C	-2.909673	-0.491501	0.041972
C	-4.597121	-2.378216	0.137227
C	-3.644262	3.221073	-0.122661
C	-6.190512	1.997406	-0.099573
C	-6.083744	3.436544	-0.159098
C	-4.761816	4.003371	-0.166944
C	-5.722507	-2.802067	-0.582521
C	-3.892181	-3.300791	0.922061
C	-6.119281	-4.129868	-0.533285
C	-4.303781	-4.625624	0.973424
C	-5.411173	-5.044175	0.242716
H	-7.167163	1.530383	-0.095230
H	-4.659908	5.081327	-0.212030
H	-6.272573	-2.092700	-1.189459
H	-3.043441	-2.982650	1.515841
H	-6.983255	-4.452993	-1.102925
H	-3.760041	-5.330537	1.592056
H	-5.725462	-6.081333	0.280737
H	-1.901806	-2.123816	-0.350525
H	-1.740883	3.114646	-0.109724
Cu	-0.000003	-0.000042	-0.083108
O	1.430181	-1.329964	-0.078814
C	2.640243	-0.912397	-0.029227
C	3.752694	-1.784657	-0.058141
C	2.909651	0.491473	0.041975
C	5.051859	-1.231651	-0.051957
C	3.644333	-3.221083	-0.122648
O	1.791669	1.265535	0.081271
C	4.192162	0.973875	0.074951
O	5.221074	0.117898	0.004723
C	6.190549	-1.997346	-0.099588
O	2.430157	-3.803459	-0.135112
C	4.761908	-4.003351	-0.166932
H	1.901664	2.123726	-0.350555
C	4.597063	2.378238	0.137212
C	6.083820	-3.436487	-0.159101
H	7.167187	-1.530297	-0.095256
H	1.740951	-3.114703	-0.109701
H	4.660030	-5.081310	-0.212009
C	5.722508	2.802085	-0.582446
C	3.892054	3.300823	0.921973
O	7.114894	-4.153835	-0.204236
C	6.119273	4.129888	-0.533192
H	6.272631	2.092714	-1.189327
C	4.303646	4.625658	0.973357
H	3.043262	2.982691	1.515683
C	5.411100	5.044203	0.242739
H	6.983296	4.453009	-1.102762
H	3.759851	5.330575	1.591935
H	5.725384	6.081361	0.280777

SCF Energy: -2102.86693024
Sum of electronic and zero-point Energies= -2102.445843
Sum of electronic and thermal Energies= -2102.412114
Sum of electronic and thermal Enthalpies= -2102.411169
Sum of electronic and thermal Free Energies= -2102.513137

==> ./AOX-II/PA/bis-/c3c4/RAF/c4p <==

O	5.360991	-0.537540	0.189057
O	2.211386	0.746847	-1.038097
O	1.985957	-3.819750	1.173443
O	1.404334	-1.480281	0.115751
O	6.528883	-4.682164	2.025386
C	3.607322	-2.115362	0.634550
C	4.985548	-1.763152	0.635804

C	4.484636	0.370559	-0.316289
C	2.642963	-1.225120	0.134919
C	3.143098	0.039365	-0.427848
C	5.120753	1.586947	-0.720731
C	3.274513	-3.419270	1.148278
C	5.970952	-2.591207	1.091884
C	5.639752	-3.891380	1.600059
C	4.247367	-4.260796	1.606022
C	6.530880	1.566286	-0.952205
C	4.404471	2.815137	-0.884268
C	7.175833	2.672609	-1.389305
C	5.043033	3.936702	-1.289819
C	6.509681	3.980528	-1.462276
H	7.005084	-2.268962	1.073477
H	3.970811	-5.237600	1.987299
H	7.068102	0.634292	-0.854321
H	3.351498	2.885030	-0.647065
H	8.226850	2.634761	-1.652100
H	4.501413	4.868914	-1.405160
H	6.827074	4.445548	-0.495058
H	2.558632	1.443024	-1.609610
H	1.435474	-3.096144	0.812310
Cu	-0.218337	-0.328188	0.152168
O	-1.771058	0.839084	0.525424
C	-3.025981	0.715895	0.378810
C	-3.936804	1.668683	0.902930
C	-3.595536	-0.401566	-0.347408
C	-5.328009	1.508969	0.703205
C	-3.532913	2.827778	1.639262
O	-2.692366	-1.267207	-0.876701
C	-4.945535	-0.517193	-0.483701
O	-5.782635	0.422007	0.019205
C	-6.271105	2.391594	1.171861
O	-2.217267	3.056804	1.873957
C	-4.456828	3.720390	2.116456
H	-3.125381	-1.903016	-1.457077
C	-5.663772	-1.585022	-1.195431
C	-5.867216	3.546150	1.907255
H	-7.323218	2.207529	0.987176
H	-1.712897	2.327930	1.460748
H	-4.115823	4.588044	2.671773
C	-6.718725	-1.254243	-2.055090
C	-5.329581	-2.932047	-1.011734
O	-6.711800	4.386044	2.360823
C	-7.410711	-2.250584	-2.729278
H	-6.988320	-0.214105	-2.198954
C	-6.027931	-3.925284	-1.688726
H	-4.543334	-3.209918	-0.317681
C	-7.066014	-3.587970	-2.550409
H	-8.221581	-1.982158	-3.397525
H	-5.765301	-4.965585	-1.531619
H	-7.609443	-4.364756	-3.077234
O	6.986477	4.747604	-2.538899
H	6.744057	5.668682	-2.397708

SCF Energy: -2178.84245120
Sum of electronic and zero-point Energies= -2178.407996
Sum of electronic and thermal Energies= -2178.371613
Sum of electronic and thermal Enthalpies= -2178.370668
Sum of electronic and thermal Free Energies= -2178.481729

==> ./AOX-II/PA/bis-/c3c4/RAF/c8a <==

O	5.315821	-0.177547	-0.315736
O	2.325350	1.526460	0.889803
O	2.037015	-3.515038	-0.193680
O	1.480706	-1.045588	0.642768
O	6.454369	-4.482684	-1.455088
C	3.648733	-1.804290	0.197569
C	5.097530	-1.459989	0.268706
C	4.501627	0.827980	0.115419
C	2.702365	-0.773259	0.491933
C	3.220277	0.575399	0.531465
C	5.113276	2.151739	-0.010958
C	3.291175	-3.072727	-0.196534
C	6.032135	-2.428079	-0.375802
C	5.649206	-3.679391	-0.902940
C	4.269346	-3.980536	-0.689845
C	5.922930	2.431417	-1.122114
C	4.927915	3.137988	0.967707
C	6.508330	3.680366	-1.261490
C	5.528656	4.382203	0.826002
C	6.312261	4.658776	-0.289611
H	7.085728	-2.169585	-0.313476
H	3.922577	-4.976681	-0.948370
H	6.076473	1.673427	-1.880956
H	4.347997	2.924692	1.858767
H	7.120419	3.892071	-2.131037
H	5.389958	5.133436	1.595253
H	6.775125	5.633268	-0.399632
H	2.609033	2.399918	0.595400
H	1.464837	-2.799137	0.153577
Cu	-0.134227	0.050376	0.323473
O	-1.758561	1.141620	-0.004469
C	-2.996884	0.859237	-0.030579
C	-3.989805	1.866667	-0.133658
C	-3.462106	-0.512622	0.030368
C	-5.359406	1.515670	-0.181234
C	-3.692863	3.265441	-0.202255
O	-2.483996	-1.454320	0.087301
C	-4.795839	-0.788093	0.006738
O	-5.713048	0.201797	-0.111471
C	-6.377611	2.432475	-0.284995

O	-2.404967	3.687091	-0.165210
C	-4.692839	4.196562	-0.304472
H	-2.844550	-2.340002	-0.033017
C	-5.414343	-2.121761	0.054455
C	-6.080068	3.826960	-0.348123
H	-7.407789	2.096371	-0.310929
H	-1.837792	2.893061	-0.098169
H	-4.432646	5.248946	-0.351644
C	-6.461824	-2.431034	-0.821949
C	-4.990987	-3.083471	0.979068
O	-6.996078	4.708777	-0.435318
C	-7.058623	-3.683583	-0.783909
H	-6.800506	-1.690267	-1.537339
C	-5.594373	-4.335366	1.013632
H	-4.206263	-2.847472	1.690050
C	-6.625514	-4.639545	0.131510
H	-7.864015	-3.915014	-1.472311
H	-5.262474	-5.069307	1.739594
H	-7.094696	-5.616960	0.159710
O	5.449919	-1.432212	1.629397
H	6.402858	-1.289342	1.712792

SCF Energy: -2178.80641300
Sum of electronic and zero-point Energies= -2178.374073
Sum of electronic and thermal Energies= -2178.337852
Sum of electronic and thermal Enthalpies= -2178.336908
Sum of electronic and thermal Free Energies= -2178.447006

==> ./AOX-II/PA/bis-/c3c4/RAF/c3 <==

O	5.476800	0.233512	-0.120464
O	2.603800	-1.219843	-0.948154
O	2.182380	3.761495	0.028052
O	1.525495	1.187938	0.154976
O	6.767290	4.727441	-0.314305
C	3.737765	1.925833	-0.034366
C	5.105793	1.581297	-0.127149
C	4.682023	-0.765151	-0.014051
C	2.755202	0.940207	0.081098
C	3.173732	-0.536670	0.127777
C	5.285381	-2.058343	-0.041750
C	3.453416	3.334851	-0.053851
C	6.126858	2.461400	-0.225891
C	5.836927	3.884868	-0.235636
C	4.459550	4.262438	-0.150426
C	6.684475	-2.156063	-0.228798
C	4.522944	-3.238081	0.110580
C	7.292739	-3.392080	-0.265802
C	5.148053	-4.469513	0.074918
C	6.526112	-4.548974	-0.113916
H	7.151623	2.118811	-0.293187
H	4.207499	5.316805	-0.157488
H	7.276101	-1.258184	-0.348068
H	3.455085	-3.188211	0.258309
H	8.363272	-3.463647	-0.413527
H	4.561902	-5.372380	0.193637
H	7.008634	-5.519371	-0.143439
H	1.692920	-0.916829	-1.070394
H	1.594123	2.985362	0.092486
Cu	-0.124518	0.088842	0.401839
O	-1.734522	-1.070547	0.425421
C	-2.964893	-0.827091	0.219457
C	-3.906332	-1.866314	0.007120
C	-3.477134	0.528941	0.209742
C	-5.272059	-1.559637	-0.200094
C	-3.562499	-3.256061	-0.002343
O	-2.562268	1.498640	0.471177
C	-4.797453	0.764575	-0.027072
O	-5.667653	-0.256094	-0.214764
C	-6.244128	-2.509526	-0.400769
O	-2.275193	-3.635838	0.189212
C	-4.516141	-4.219745	-0.201066
H	-2.991558	2.349836	0.611935
C	-5.456128	2.078936	-0.061883
C	-5.900002	-3.894630	-0.407326
H	-7.272990	-2.206119	-0.556723
H	-1.745327	-2.823891	0.317909
H	-4.220368	-5.263738	-0.201768
C	-6.682574	2.254550	0.591026
C	-4.890364	3.156334	-0.754071
O	-6.773572	-4.805078	-0.586781
C	-7.317966	3.488202	0.566220
H	-7.131197	1.424404	1.124617
C	-5.533162	4.388806	-0.776603
H	-3.961064	3.027695	-1.299299
C	-6.744252	4.559026	-0.114609
H	-8.263595	3.614871	1.081850
H	-5.088722	5.213776	-1.322259
H	-7.243444	5.521682	-0.132918
O	2.845745	-1.074983	1.375389
H	1.956412	-0.784756	1.624150

SCF Energy: -2178.87559623
Sum of electronic and zero-point Energies= -2178.439375
Sum of electronic and thermal Energies= -2178.403354
Sum of electronic and thermal Enthalpies= -2178.402410
Sum of electronic and thermal Free Energies= -2178.511130

==> ./AOX-II/PA/bis-/c3c4/RAF/c4 <==

O	4.888519	0.743366	-0.460643
O	3.409079	-1.818141	1.703511
O	0.549027	-0.283937	-2.385041
O	2.048657	0.721350	-0.090104
O	3.932241	1.677111	-4.938980
C	2.671621	0.241272	-1.295989
C	4.044113	0.755302	-1.510537
C	4.804613	-0.197014	0.560785
C	2.499680	-0.642341	-0.124119
C	3.670874	-0.895471	0.746338
C	6.036157	-0.242745	1.361912
C	1.793234	0.183927	-2.501199
C	4.453830	1.231581	-2.695878
C	3.556933	1.232570	-3.845236
C	2.230304	0.665293	-3.681669
C	6.697507	0.948348	1.686463
C	6.576686	-1.459613	1.792665
C	7.864242	0.919864	2.438111
C	7.743319	-1.481239	2.548716
C	8.389195	-0.293742	2.874696
H	5.469848	1.581337	-2.829890
H	1.576209	0.642671	-4.544984
H	6.293376	1.897196	1.352663
H	6.101814	-2.395776	1.517702
H	8.364815	1.849765	2.685279
H	8.153654	-2.432015	2.870774
H	9.301296	-0.312843	3.461197
H	4.086819	-1.828353	2.389571
H	0.396916	-0.709039	-1.525940
Cu	0.000301	1.247412	0.148388
O	-1.921620	1.662783	0.511430
C	-2.981099	0.964781	0.470279
C	-4.262036	1.539608	0.675184
C	-2.941025	-0.460670	0.204126
C	-5.424252	0.736740	0.604134
C	-4.470145	2.931442	0.936803
O	-1.705913	-0.974018	-0.027810
C	-4.091422	-1.189577	0.187787
O	-5.297346	-0.599040	0.367459
C	-6.698450	1.223997	0.769530
O	-3.408528	3.770690	1.020417
C	-5.732012	3.438420	1.105126
H	-1.761514	-1.866974	-0.386531
C	-4.206453	-2.634925	-0.059330
C	-6.904653	2.612862	1.025619
H	-7.547152	0.552910	0.704225
H	-2.601066	3.239491	0.868013
H	-5.853802	4.498706	1.301204
C	-5.207230	-3.113853	-0.914100
C	-3.347355	-3.547318	0.564329
O	-8.070328	3.105929	1.176500
C	-5.332270	-4.475654	-1.151089
H	-5.880760	-2.415371	-1.397468
C	-3.479598	-4.910211	0.324774
H	-2.592717	-3.197169	1.260588
C	-4.468044	-5.377120	-0.534892
H	-6.106506	-4.834654	-1.820283
H	-2.813867	-5.607728	0.820956
H	-4.569061	-6.440845	-0.720955
O	1.537933	-1.591899	-0.176664
H	1.361605	-1.936902	0.710007

SCF Energy: -2178.83784812

Sum of electronic and zero-point Energies= -2178.403183

Sum of electronic and thermal Energies= -2178.367002

Sum of electronic and thermal Enthalpies= -2178.366058

Sum of electronic and thermal Free Energies= -2178.474699

==> ./AOX-II/PA/bis-/c3c4/RAF/c3p <==

O	5.323267	0.569100	-0.095522
O	2.137457	-1.176770	-0.164296
O	1.924950	3.962465	-0.319009
O	1.344516	1.389136	-0.275781
O	6.487869	5.116853	-0.128207
C	3.555861	2.187580	-0.216127
C	4.935413	1.876831	-0.138570
C	4.428015	-0.442585	-0.132836
C	2.588489	1.153430	-0.226606
C	3.089468	-0.213727	-0.167804
C	5.091946	-1.757086	-0.075824
C	3.222813	3.580353	-0.254590
C	5.928670	2.822253	-0.107972
C	5.594938	4.210989	-0.152421
C	4.198567	4.541232	-0.225691
C	6.158261	-1.948046	0.776542
C	4.671326	-2.837020	-0.874885
C	6.893786	-3.196779	0.768728
C	5.283065	-4.092503	-0.832281
C	6.331519	-4.312287	0.021910
H	6.966816	2.516394	-0.050976
H	3.911917	5.587183	-0.257319
H	6.527198	-1.156025	1.417467
H	3.845335	-2.693134	-1.561966
H	7.558177	-2.852298	-0.104224
H	4.907962	-4.892085	-1.456953
H	6.827703	-5.273648	0.090814
H	2.425444	-1.970413	0.298818
H	1.376495	3.152970	-0.320040
Cu	-0.215464	0.157395	-0.112040
O	-1.768046	-1.082837	-0.019187
C	-3.021877	-0.883696	-0.006204
C	-3.950095	-1.953017	-0.097728

C -3.573499 0.451197 0.121270
C -5.339973 -1.693314 -0.064161
C -3.566716 -3.327676 -0.208036
O -2.657521 1.444761 0.263592
C -4.921819 0.642303 0.110975
O -5.775991 -0.406384 0.032482
C -6.299938 -2.673820 -0.133052
O -2.253258 -3.663090 -0.240746
C -4.507695 -4.321808 -0.278300
H -3.075672 2.260087 0.561944
C -5.625538 1.928343 0.226814
C -5.916395 -4.044150 -0.241301
H -7.349871 -2.405861 -0.104714
H -1.736629 -2.835079 -0.175473
H -4.181480 -5.353499 -0.359822
C -6.737559 2.033656 1.071762
C -5.220848 3.046618 -0.511101
O -6.778309 -4.981323 -0.301083
C -7.417230 3.238126 1.187539
H -7.061755 1.171603 1.643604
C -5.908030 4.249226 -0.393287
H -4.383977 2.973512 -1.197167
C -7.003784 4.349415 0.457266
H -8.272019 3.309706 1.851125
H -5.590076 5.106270 -0.976620
H -7.537581 5.289111 0.548328
O 7.646984 -3.426759 1.902951
H 8.268287 -4.144690 1.736618

SCF Energy: -2178.83472728
Sum of electronic and zero-point Energies= -2178.402747
Sum of electronic and thermal Energies= -2178.367649
Sum of electronic and thermal Enthalpies= -2178.366705
Sum of electronic and thermal Free Energies= -2178.472987

==> ./AOX-II/PA/bis-/c3c4/RAF/c5p <==
O 5.324115 -0.502702 -0.312841
O 2.144361 1.208427 0.106234
O 2.219933 -3.790651 1.317512
O 1.517143 -1.291218 0.876915
O 6.687140 -4.943071 0.370278
C 3.702469 -2.068467 0.505328
C 5.018144 -1.774956 0.071639
C 4.409467 0.493418 -0.281220
C 2.709292 -1.058912 0.518201
C 3.121123 0.272456 0.089198
C 4.991868 1.768281 -0.737865
C 3.453300 -3.424951 0.893715
C 6.027032 -2.702914 0.014701
C 5.777593 -4.054115 0.406194
C 4.446047 -4.367324 0.845656
C 5.849052 1.776056 -1.859156
C 4.736802 2.959789 -0.100311
C 6.440497 2.939319 -2.352270
C 5.413981 4.176344 -0.510903
C 6.176367 4.142344 -1.750204
H 7.013783 -2.410696 -0.325272
H 4.223938 -5.385199 1.148573
H 6.051575 0.838050 -2.362913
H 4.102196 3.021068 0.776531
H 7.068638 2.892534 -3.231668
H 6.597495 5.072571 -2.114530
H 2.335582 1.943627 -0.485488
H 1.645651 -3.000101 1.276809
Cu -0.158439 -0.216176 0.859574
O -1.802925 0.898335 0.876900
C -3.004150 0.701647 0.516624
C -3.964602 1.745960 0.519324
C -3.458701 -0.602603 0.074173
C -5.287669 1.497900 0.085427
C -3.673455 3.087638 0.924314
O -2.505799 -1.570324 0.057858
C -4.753895 -0.787542 -0.305452
O -5.634414 0.242240 -0.312404
C -6.268486 2.459411 0.043613
O -2.426713 3.410530 1.348188
C -4.636889 4.061845 0.891551
H -2.817271 -2.359267 -0.398944
C -5.355838 -2.040238 -0.786024
C -5.978106 3.795655 0.451994
H -7.264707 2.201243 -0.296786
H -1.879610 2.601296 1.293820
H -4.381740 5.068346 1.206731
C -6.203481 -2.017514 -1.900637
C -5.113034 -3.257114 -0.138229
O -6.861909 4.713681 0.430767
C -6.779633 -3.191435 -2.365596
H -6.401968 -1.079431 -2.406318
C -5.694956 -4.429077 -0.607697
H -4.493150 -3.287846 0.751557
C -6.525336 -4.400389 -1.722751
H -7.428541 -3.162999 -3.234101
H -5.505557 -5.363969 -0.091922
H -6.977787 -5.315883 -2.087985
H 6.335371 3.969721 0.142034
O 4.781716 5.341499 -0.116981
H 5.380828 6.089000 -0.227208

SCF Energy: -2178.83502658
Sum of electronic and zero-point Energies= -2178.402108

Sum of electronic and thermal Energies= -2178.365700
Sum of electronic and thermal Enthalpies= -2178.364755
Sum of electronic and thermal Free Energies= -2178.474621

==> ./AOX-II/PA/bis-/c3c4/RAF/c2p <==

O	-5.418747	0.417491	0.089361
O	-2.279007	-1.396620	0.006244
O	-1.919573	3.714603	-0.029344
O	-1.411725	1.131106	-0.006079
O	-6.450531	4.986746	0.021759
C	-3.600398	1.982989	0.028406
C	-4.993326	1.707472	0.053587
C	-4.555791	-0.625014	0.101497
C	-2.660251	0.930951	0.026907
C	-3.200245	-0.431290	0.058854
C	-5.214880	-1.916468	0.166823
C	-3.227109	3.369953	-0.003642
C	-5.957420	2.680003	0.053250
C	-5.583772	4.060437	0.024452
C	-4.175864	4.355039	-0.004294
C	-6.332736	-2.271426	-0.742764
C	-4.759740	-2.903153	1.027004
C	-7.103285	-3.473892	-0.415374
C	-5.447184	-4.105868	1.175122
C	-6.620796	-4.397436	0.463429
H	-7.005222	2.404279	0.070464
H	-3.862061	5.393172	-0.025200
H	-3.913362	-2.706605	1.672507
H	-8.018172	-3.637277	-0.974554
H	-5.080468	-4.820121	1.903098
H	-7.157340	-5.318463	0.649156
H	-2.660806	-2.269699	-0.146955
H	-1.392674	2.891000	-0.024892
Cu	0.211090	-0.014689	-0.013348
O	1.842867	-1.142382	-0.021080
C	3.086282	-0.884434	-0.012412
C	4.061597	-1.915202	-0.022112
C	3.579039	0.479062	-0.004977
C	5.438980	-1.593382	-0.029239
C	3.738549	-3.309431	-0.048099
O	2.624571	1.445284	-0.049045
C	4.918070	0.726637	0.029553
O	5.817975	-0.285279	0.003699
C	6.440867	-2.533331	-0.058327
O	2.440899	-3.702674	-0.046240
C	4.721837	-4.263514	-0.077277
H	3.010501	2.302300	-0.262505
C	5.562738	2.048658	0.049538
C	6.117139	-3.923052	-0.084124
H	7.478024	-2.218300	-0.058743
H	1.890148	-2.894076	-0.033647
H	4.441052	-5.311494	-0.096668
C	6.670174	2.290892	-0.772853
C	5.105320	3.065788	0.895471
O	7.017605	-4.824567	-0.110854
C	7.293441	3.530967	-0.759881
H	7.034772	1.507723	-1.427754
C	5.735917	4.304483	0.906013
H	4.272565	2.883499	1.566129
C	6.827295	4.541472	0.077326
H	8.145334	3.709937	-1.406683
H	5.377357	5.081514	1.572022
H	7.317216	5.509025	0.086602
H	-5.659846	-2.763210	-1.513793
O	-7.014089	-1.211509	-1.321615
H	-7.527297	-1.542294	-2.067082

SCF Energy: -2178.83625835
Sum of electronic and zero-point Energies= -2178.402746
Sum of electronic and thermal Energies= -2178.366453
Sum of electronic and thermal Enthalpies= -2178.365509
Sum of electronic and thermal Free Energies= -2178.475206

==> ./AOX-II/PA/bis-/c3c4/RAF/c7 <==

O	-5.418060	0.117243	-0.030779
O	-2.141316	1.625236	0.194806
O	-2.291420	-3.508472	-0.038583
O	-1.545702	-0.994871	0.135436
O	-6.422656	-2.938087	1.099352
C	-3.814561	-1.619137	0.037037
C	-5.107693	-1.172227	0.009582
C	-4.471456	1.084853	-0.013200
C	-2.752992	-0.646807	0.089568
C	-3.150750	0.741535	0.089872
C	-5.038534	2.436823	-0.064586
C	-3.568406	-3.066892	-0.051546
C	-6.267573	-2.070289	-0.021853
C	-5.974321	-3.503494	-0.134390
C	-4.579331	-3.948987	-0.180681
C	-6.165788	2.741461	0.708160
C	-4.484058	3.420210	-0.892078
C	-6.716518	4.014154	0.663583
C	-5.043486	4.691484	-0.933101
C	-6.156104	4.991761	-0.154523
H	-7.189383	-1.661784	-0.423759
H	-4.372504	-5.004377	-0.314540
H	-6.600700	1.982970	1.348969
H	-3.633810	3.187297	-1.524725
H	-7.584750	4.244403	1.270930

H	-4.612814	5.444492	-1.583446
H	-6.589364	5.985353	-0.187638
H	-2.466247	2.524668	0.323215
H	-1.696658	-2.736045	0.028975
Cu	0.202175	-0.036203	0.134624
O	1.863437	1.055026	0.126662
C	3.100831	0.778819	0.062063
C	4.090325	1.793915	0.002113
C	3.571542	-0.592249	0.059506
C	5.461580	1.450894	-0.048130
C	3.788529	3.193104	0.008501
O	2.602987	-1.539280	0.167450
C	4.903729	-0.861927	-0.028757
O	5.819001	0.136331	-0.066550
C	6.477148	2.375682	-0.090084
O	2.498147	3.606537	0.056680
C	4.785593	4.132183	-0.032991
H	2.984545	-2.404879	0.351646
C	5.528657	-2.193723	-0.045818
C	6.174683	3.770478	-0.082074
H	7.508630	2.044975	-0.130048
H	1.935088	2.807147	0.089108
H	4.521239	5.184590	-0.026542
C	6.661010	-2.435594	0.742002
C	5.028526	-3.220204	-0.855478
O	7.087900	4.658845	-0.116606
C	7.267248	-3.684085	0.731210
H	7.058539	-1.645209	1.368486
C	5.642145	-4.467486	-0.864058
H	4.175396	-3.038532	-1.500663
C	6.758937	-4.703609	-0.069687
H	8.139224	-3.862387	1.350894
H	5.250627	-5.251811	-1.502390
H	7.235818	-5.677669	-0.077677
O	-6.908378	-4.275556	-0.784882
H	-6.770951	-5.200782	-0.549752

SCF Energy: -2178.86789959
Sum of electronic and zero-point Energies= -2178.432018
Sum of electronic and thermal Energies= -2178.396171
Sum of electronic and thermal Enthalpies= -2178.395227
Sum of electronic and thermal Free Energies= -2178.504663

==> ./AOX-II/PA/bis-/c3c4/RAF/c5 <==

O	5.469433	0.049555	-0.228791
O	2.250986	1.673277	0.028448
O	2.809497	-3.192652	1.727317
O	1.618034	-0.907732	0.581813
O	7.043287	-4.339445	0.317374
C	3.833407	-1.576546	0.271077
C	5.133962	-1.219578	0.042418
C	4.542811	1.027714	-0.287632
C	2.798843	-0.598680	0.285320
C	3.220898	0.751991	-0.016576
C	5.119553	2.339981	-0.591081
C	3.535955	-3.028941	0.545241
C	6.215652	-2.130373	0.073702
C	6.073759	-3.524961	0.330270
C	4.750343	-3.896081	0.657557
C	6.329453	2.712741	0.009133
C	4.492150	3.219741	-1.481811
C	6.889003	3.951612	-0.265359
C	5.062873	4.456593	-1.754917
C	6.256908	4.826549	-1.145354
H	7.208000	-1.730083	-0.108454
H	4.547870	-4.923637	0.948327
H	6.819565	2.035340	0.698800
H	3.580061	2.928334	-1.992019
H	7.820615	4.236186	0.210742
H	4.576417	5.126695	-2.454568
H	6.698256	5.793763	-1.359078
H	2.597809	2.569017	-0.067130
H	2.078348	-2.553736	1.705077
Cu	-0.125419	0.033837	0.562858
O	-1.809424	1.088292	0.570020
C	-3.022515	0.812905	0.315354
C	-4.033857	1.807085	0.337237
C	-3.441396	-0.539040	0.001620
C	-5.378343	1.461592	0.065820
C	-3.782597	3.184058	0.637573
O	-2.453538	-1.470884	0.033058
C	-4.748186	-0.808221	-0.272487
O	-5.687014	0.168011	-0.229183
C	-6.413496	2.365383	0.073465
O	-2.521892	3.596589	0.917461
C	-4.799865	4.101884	0.649684
H	-2.815418	-2.361536	-0.030537
C	-5.324363	-2.125023	-0.583320
C	-6.160693	3.738934	0.367421
H	-7.422087	2.035196	-0.147099
H	-1.939273	2.812191	0.870992
H	-4.574374	5.137969	0.880240
C	-6.525197	-2.512107	0.024802
C	-4.705813	-2.995862	-1.487848
O	-7.091777	4.609009	0.380763
C	-7.083609	-3.751470	-0.254556
H	-7.013592	-1.842290	0.723355
C	-5.272001	-4.235003	-1.765363
H	-3.798024	-2.696689	-2.001407
C	-6.457857	-4.617344	-1.147870
H	-8.009853	-4.043204	0.228303
H	-4.788293	-4.896938	-2.475124

H	-6.897292	-5.584701	-1.365645
O	2.831945	-3.496514	-0.587278
H	2.657449	-4.440393	-0.476799

SCF Energy: -2178.82222981
Sum of electronic and zero-point Energies= -2178.387753
Sum of electronic and thermal Energies= -2178.351154
Sum of electronic and thermal Enthalpies= -2178.350209
Sum of electronic and thermal Free Energies= -2178.461221

==> ./AOX-II/PA/bis-/c3c4/RAF/c1p <==

O	-5.442124	0.362829	0.253346
O	-2.328231	-1.292093	-0.645479
O	-1.903440	3.431550	1.324384
O	-1.439647	1.037646	0.326689
O	-6.413195	4.614834	1.985114
C	-3.609113	1.822493	0.751655
C	-5.001405	1.569065	0.710948
C	-4.592420	-0.596919	-0.156543
C	-2.689204	0.843596	0.309628
C	-3.246717	-0.415493	-0.186684
C	-5.306663	-1.799912	-0.669744
C	-3.215794	3.107744	1.250206
C	-5.954180	2.468793	1.112815
C	-5.560372	3.751186	1.606278
C	-4.150137	4.023052	1.655621
C	-6.328058	-1.659124	-1.734232
C	-4.698519	-3.123071	-0.496615
C	-6.612911	-2.795622	-2.603871
C	-5.056846	-4.142385	-1.300223
C	-6.010040	-3.976652	-2.380703
H	-7.004768	2.209241	1.055545
H	-3.819118	4.987046	2.027337
H	-6.574452	-0.663136	-2.076771
H	-3.965319	-3.247144	0.290095
H	-7.272326	-2.630456	-3.445590
H	-4.603094	-5.115752	-1.158042
H	-6.201904	-4.819706	-3.032931
H	-2.716490	-2.009526	-1.159043
H	-1.388531	2.668937	0.993969
Cu	0.167698	-0.085652	0.048843
O	1.811272	-1.200102	0.052300
C	3.041261	-0.879245	0.063382
C	4.030534	-1.720395	0.631721
C	3.500644	0.359033	-0.533906
C	5.391357	-1.333792	0.611346
C	3.739853	-2.982140	1.241980
O	2.535881	1.100842	-1.137479
C	4.820010	0.695946	-0.492143
O	5.735577	-0.136606	0.060372
C	6.407139	-2.096705	1.134760
O	2.460193	-3.426025	1.299898
C	4.737612	-3.758765	1.769893
H	2.922376	1.827253	-1.638841
C	5.431545	1.912809	-1.047003
C	6.116184	-3.355553	1.741403
H	7.429714	-1.739768	1.091388
H	1.894622	-2.748689	0.876972
H	4.482638	-4.710356	2.224791
C	6.637552	1.821052	-1.753248
C	4.841716	3.169698	-0.865550
O	7.030069	-4.092167	2.237651
C	7.228586	2.960539	-2.280915
H	7.105095	0.853317	-1.894664
C	5.440039	4.307309	-1.394853
H	3.930312	3.267830	-0.285341
C	6.630863	4.206055	-2.105907
H	8.158698	2.875829	-2.832118
H	4.977369	5.275758	-1.240015
H	7.095432	5.095058	-2.518338
O	-6.789710	-1.724106	-0.282540
H	-7.141764	-2.600216	-0.025871

SCF Energy: -2178.79939580
Sum of electronic and zero-point Energies= -2178.363619
Sum of electronic and thermal Energies= -2178.328101
Sum of electronic and thermal Enthalpies= -2178.327156
Sum of electronic and thermal Free Energies= -2178.434231

==> ./AOX-II/PA/bis-/c3c4/RAF/c6p <==

O	-5.501889	0.421587	-0.089511
O	-2.322818	-1.268389	-0.261417
O	-2.100647	3.815121	-0.304368
O	-1.523355	1.249295	-0.214419
O	-6.663867	4.946622	-0.351482
C	-3.733665	2.041197	-0.210369
C	-5.118448	1.718797	-0.183138
C	-4.629120	-0.623264	-0.049851
C	-2.767176	1.023643	-0.198515
C	-3.262511	-0.367229	-0.188838
C	-5.269806	-1.855971	0.258283
C	-3.396821	3.440507	-0.281022
C	-6.106348	2.660001	-0.234510
C	-5.771106	4.053719	-0.310019
C	-4.371911	4.394738	-0.329448
C	-6.623576	-1.840999	0.629571
C	-4.548373	-3.170895	0.335764
C	-7.290381	-2.994958	0.975036

C	-5.378099	-4.368454	0.613428
C	-6.672269	-4.276675	0.972129
H	-7.146822	2.359463	-0.205850
H	-4.092541	5.441166	-0.382970
H	-7.162746	-0.905784	0.630386
H	-8.338640	-2.922744	1.241966
H	-4.861759	-5.320934	0.560900
H	-7.251306	-5.156751	1.219742
H	-2.685955	-2.158565	-0.514111
H	-1.551005	3.006701	-0.270633
Cu	0.123354	0.128739	-0.204649
O	1.746111	-1.012721	-0.203842
C	2.991390	-0.790571	-0.095417
C	3.941439	-1.844251	-0.122650
C	3.512763	0.554413	0.051125
C	5.323425	-1.560247	-0.022413
C	3.589122	-3.224319	-0.266772
O	2.581993	1.543782	0.028713
C	4.852784	0.763086	0.178176
O	5.729405	-0.268599	0.127498
C	6.303684	-2.522419	-0.057544
O	2.285540	-3.583202	-0.369040
C	4.550681	-4.200245	-0.303375
H	2.997686	2.410793	-0.035680
C	5.526203	2.062016	0.328686
C	5.951028	-3.897800	-0.200691
H	7.345658	-2.236294	0.028274
H	1.751801	-2.764260	-0.335198
H	4.248007	-5.236477	-0.413129
C	6.677130	2.333164	-0.421858
C	5.053544	3.027026	1.225741
O	6.831393	-4.818744	-0.234568
C	7.328594	3.551375	-0.288869
H	7.054025	1.589801	-1.115054
C	5.712097	4.244399	1.355984
H	4.187012	2.818856	1.844668
C	6.847047	4.511068	0.597939
H	8.214848	3.753134	-0.880476
H	5.340770	4.980422	2.060454
H	7.358925	5.461647	0.701084
H	-3.925924	-3.074527	1.253694
O	-3.706976	-3.354396	-0.789303
H	-3.226335	-4.187044	-0.698710

SCF Energy: -2178.84738272
Sum of electronic and zero-point Energies= -2178.412732
Sum of electronic and thermal Energies= -2178.376796
Sum of electronic and thermal Enthalpies= -2178.375852
Sum of electronic and thermal Free Energies= -2178.485019

==> ./AOX-II/PA/bis-/c3c4/RAF/c8 <==

O	5.388151	-0.007182	-0.068538
O	2.127131	1.554942	-0.221275
O	2.253551	-3.569612	-0.136732
O	1.501641	-1.065744	-0.192039
O	6.803212	-4.361350	0.563818
C	3.763091	-1.703906	-0.116575
C	5.061252	-1.280708	-0.101153
C	4.451765	0.981374	-0.058582
C	2.710505	-0.726073	-0.149982
C	3.126037	0.659268	-0.140633
C	5.046254	2.320046	0.003080
C	3.507989	-3.147487	-0.074748
C	6.209078	-2.219058	-0.215265
C	5.867895	-3.646644	0.198308
C	4.512977	-4.062949	0.071952
C	6.175600	2.609535	-0.772848
C	4.517127	3.305757	0.844837
C	6.753035	3.869769	-0.717345
C	5.103261	4.564351	0.896363
C	6.217641	4.849704	0.114632
H	6.376662	-2.309903	-1.306636
H	4.265156	-5.108785	0.205574
H	6.591554	1.849246	-1.423980
H	3.666088	3.083181	1.480097
H	7.622768	4.088479	-1.326746
H	4.692418	5.318831	1.557691
H	6.672192	5.833391	0.156501
H	2.460186	2.450712	-0.355736
H	1.654764	-2.791524	-0.185972
Cu	-0.234224	-0.070287	-0.183215
O	-1.880329	1.044709	-0.168001
C	-3.119396	0.784016	-0.075557
C	-4.093812	1.811093	0.018940
C	-3.608188	-0.580961	-0.076234
C	-5.468065	1.485280	0.096220
C	-3.773960	3.206273	0.023352
O	-2.654725	-1.539094	-0.214719
C	-4.941852	-0.833997	0.035944
O	-5.842580	0.175558	0.105662
C	-6.470083	2.422464	0.172663
O	-2.479729	3.603826	-0.049244
C	-4.757349	4.157617	0.099523
H	-3.051584	-2.400001	-0.387668
C	-5.584370	-2.157492	0.046636
C	-6.149719	3.813219	0.174118
H	-7.504695	2.104525	0.231156
H	-1.928001	2.797964	-0.105576
H	-4.479184	5.206476	0.099920
C	-6.733312	-2.373736	-0.724467
C	-5.083711	-3.201357	0.833257
O	-7.050714	4.712537	0.236920

C	-7.355637	-3.614303	-0.719995
H	-7.131163	-1.569597	-1.332977
C	-5.713327	-4.440733	0.835500
H	-4.217667	-3.039561	1.466494
C	-6.846811	-4.651350	0.057774
H	-8.240660	-3.772644	-1.326475
H	-5.321148	-5.238847	1.456070
H	-7.336340	-5.619135	0.060904
O	7.358569	-1.737938	0.418989
H	7.864241	-2.523768	0.673588

SCF Energy: -2178.90373247
Sum of electronic and zero-point Energies= -2178.467567
Sum of electronic and thermal Energies= -2178.431352
Sum of electronic and thermal Enthalpies= -2178.430408
Sum of electronic and thermal Free Energies= -2178.541067

==> ./AOX-II/PA/bis-/c3c4/RAF/c2 <==

O	-5.531391	0.221186	0.052697
O	-2.420045	-1.403613	-0.166965
O	-2.152381	3.673529	-0.198188
O	-1.527538	1.130877	-0.285192
O	-6.704123	4.746109	0.096058
C	-3.760215	1.881508	-0.031217
C	-5.152047	1.532759	0.069607
C	-4.651927	-0.748162	0.577351
C	-2.762977	0.902696	-0.105808
C	-3.241752	-0.470536	0.053770
C	-5.168333	-2.107708	0.149277
C	-3.435585	3.298120	-0.094402
C	-6.126085	2.468656	0.114245
C	-5.800421	3.885642	0.059672
C	-4.410110	4.246710	-0.039379
C	-5.720008	-2.271905	-1.123984
C	-5.051467	-3.207562	1.000249
C	-6.167044	-3.521137	-1.530608
C	-5.502353	-4.457512	0.586091
C	-6.061095	-4.615346	-0.675829
H	-7.166277	2.173640	0.176049
H	-4.145098	5.296550	-0.077411
H	-5.803860	-1.423776	-1.792899
H	-4.614066	-3.097285	1.985840
H	-6.599894	-3.640284	-2.517476
H	-5.415059	-5.305462	1.255903
H	-6.412359	-5.590116	-0.995999
H	-2.796489	-2.299850	-0.042212
H	-1.591762	2.872217	-0.254065
Cu	0.112793	0.011555	-0.278715
O	1.755098	-1.105683	-0.257473
C	2.989137	-0.831472	-0.131112
C	3.968624	-1.847908	0.008820
C	3.466490	0.537648	-0.139935
C	5.337250	-1.508870	0.124536
C	3.660768	-3.245852	0.024390
O	2.510699	1.484804	-0.326506
C	4.794051	0.804242	0.010031
O	5.700141	-0.195855	0.126424
C	6.344349	-2.435627	0.246218
O	2.373247	-3.656793	-0.083298
C	4.649413	-4.186855	0.145393
H	2.904828	2.348039	-0.493465
C	5.424008	2.133804	0.015149
C	6.035987	-3.829042	0.257121
H	7.373819	-2.107429	0.332235
H	1.816236	-2.857325	-0.168871
H	4.380565	-5.238099	0.152247
C	6.595135	2.346279	-0.722893
C	4.886541	3.189214	0.761436
O	6.942083	-4.719615	0.358230
C	7.202928	3.594055	-0.726836
H	7.021729	1.533600	-1.299708
C	5.501517	4.435899	0.755054
H	4.002290	3.031995	1.370382
C	6.657027	4.642546	0.009353
H	8.105358	3.749279	-1.307933
H	5.079843	5.243302	1.343412
H	7.134611	5.616280	0.005112
O	-4.483503	-0.596605	1.949937
H	-5.254571	-0.952833	2.411262

SCF Energy: -2178.86337126
Sum of electronic and zero-point Energies= -2178.427588
Sum of electronic and thermal Energies= -2178.391776
Sum of electronic and thermal Enthalpies= -2178.390832
Sum of electronic and thermal Free Energies= -2178.499523

==> ./AOX-II/PA/bis-/c3c4/RAF/c4a <==

O	5.500108	-0.199942	-0.199141
O	2.380045	1.672317	-0.268124
O	2.180973	-3.153263	1.735950
O	1.616116	-0.702068	0.825754
O	5.815210	-4.835308	-0.672542
C	3.850037	-1.550150	0.965094
C	5.034168	-1.458045	0.054547
C	4.649205	0.847496	-0.283289
C	2.803546	-0.507233	0.531066
C	3.303380	0.695485	-0.054460
C	5.327882	2.077621	-0.691393

C	3.300973	-2.969831	1.032184
C	5.677107	-2.503018	-0.462332
C	5.194796	-3.869855	-0.218830
C	3.963983	-4.026372	0.524817
C	6.420022	2.006993	-1.567739
C	4.929784	3.323168	-0.186110
C	7.084638	3.163560	-1.946405
C	5.610782	4.473384	-0.558470
C	6.682889	4.397838	-1.442649
H	6.548448	-2.365223	-1.090426
H	3.570700	-5.026531	0.661315
H	6.735211	1.047289	-1.958882
H	4.108574	3.391636	0.516893
H	7.920070	3.101681	-2.634604
H	5.305564	5.430903	-0.152067
H	7.208300	5.300074	-1.735892
H	2.638734	2.237770	-1.004839
H	1.665753	-2.326045	1.749800
Cu	-0.116770	0.284633	0.602574
O	-1.864340	1.233951	0.588532
C	-3.055384	0.886556	0.319315
C	-4.123908	1.819149	0.318353
C	-3.384707	-0.484916	-0.013379
C	-5.435354	1.399621	-0.005429
C	-3.958465	3.210273	0.611860
O	-2.328469	-1.338282	-0.048230
C	-4.672835	-0.838041	-0.281410
O	-5.663640	0.086340	-0.287795
C	-6.517509	2.245763	-0.046556
O	-2.732281	3.693521	0.929799
C	-5.023606	4.071177	0.576731
H	-2.572705	-2.177564	-0.453872
C	-5.155830	-2.181401	-0.634415
C	-6.350940	3.633008	0.245219
H	-7.497711	1.858862	-0.300470
H	-2.103585	2.944550	0.896751
H	-4.863370	5.120067	0.803904
C	-6.081578	-2.333010	-1.674441
C	-4.720630	-3.315120	0.062176
O	-7.326380	4.452847	0.215471
C	-6.546657	-3.594377	-2.019205
H	-6.427787	-1.460665	-2.216936
C	-5.192765	-4.575304	-0.286186
H	-4.035955	-3.213620	0.897553
C	-6.102242	-4.718905	-1.328393
H	-7.257740	-3.700246	-2.831200
H	-4.855186	-5.444632	0.267195
H	-6.468337	-5.703263	-1.599146
O	4.314106	-1.170185	2.255019
H	3.571059	-1.141908	2.871107

SCF Energy: -2178.86320090

Sum of electronic and zero-point Energies= -2178.427507
Sum of electronic and thermal Energies= -2178.391304
Sum of electronic and thermal Enthalpies= -2178.390360
Sum of electronic and thermal Free Energies= -2178.499609

==> ./AOX-II/PA/bis-/c3c4/RAF/c6 <==

O	5.495012	0.199563	0.073256
O	2.182197	1.704822	-0.139508
O	2.418009	-3.412889	-0.047060
O	1.612218	-0.943600	-0.103839
O	6.955706	-4.214626	-0.337586
C	3.868781	-1.539172	0.061087
C	5.238427	-1.120651	0.060266
C	4.517337	1.153212	0.056323
C	2.809222	-0.572451	-0.029708
C	3.199591	0.820385	-0.033639
C	5.077232	2.508480	0.094629
C	3.605854	-2.902436	0.067796
C	6.321103	-1.959481	0.000037
C	6.106433	-3.359871	-0.067923
C	4.714770	-3.861578	0.307694
C	6.210513	2.810034	-0.671447
C	4.510774	3.500785	0.904275
C	6.751442	4.087202	-0.642034
C	5.062701	4.775447	0.933117
C	6.178825	5.072562	0.158191
H	7.317851	-1.553547	-0.114752
H	4.771890	-3.867836	1.417936
H	6.657459	2.045523	-1.296308
H	3.657268	3.271935	1.533723
H	7.622919	4.314529	-1.245848
H	4.623085	5.534136	1.570771
H	6.605637	6.069239	0.180909
H	2.506469	2.594943	-0.320000
H	1.768874	-2.668935	-0.115565
Cu	-0.150738	-0.002592	-0.109440
O	-1.813934	1.088534	-0.107216
C	-3.051897	0.811685	-0.058256
C	-4.043767	1.825989	-0.037871
C	-3.520537	-0.559711	-0.032803
C	-5.415144	1.481376	-0.002931
C	-3.744175	3.225266	-0.072473
O	-2.549233	-1.507601	-0.104161
C	-4.853381	-0.830094	0.042350
O	-5.770741	0.166866	0.041781
C	-6.432751	2.404813	-0.000907
O	-2.453739	3.639868	-0.108181
C	-4.743346	4.162994	-0.071167
H	-2.926390	-2.376233	-0.283395

C	-5.475730	-2.162492	0.085343
C	-6.132479	3.799702	-0.036566
H	-7.464225	2.073121	0.029983
H	-1.888986	2.840977	-0.112001
H	-4.480704	5.215509	-0.098000
C	-6.599798	-2.425943	-0.707334
C	-4.981985	-3.167694	0.925231
O	-7.047591	4.686668	-0.036789
C	-7.204163	-3.674893	-0.671509
H	-6.992568	-1.652009	-1.356924
C	-5.593797	-4.415417	0.958808
H	-4.135312	-2.968565	1.573722
C	-6.702281	-4.673254	0.159532
H	-8.069657	-3.870127	-1.295156
H	-5.207388	-5.182928	1.620280
H	-7.177762	-5.647638	0.187115
O	4.447481	-5.140080	-0.179411
H	5.313227	-5.525881	-0.381680

SCF Energy: -2178.89575803
Sum of electronic and zero-point Energies= -2178.460170
Sum of electronic and thermal Energies= -2178.424005
Sum of electronic and thermal Enthalpies= -2178.423061
Sum of electronic and thermal Free Energies= -2178.533137

==> ./AOX-II/PA/bis-/c3c4/vEA <==

O	-5.205907	-0.132080	-0.046343
O	-1.771741	-1.270326	0.217335
O	-2.440646	3.812975	-0.012989
O	-1.419570	1.336928	0.064048
O	-7.128078	4.141435	-0.317140
C	-3.743897	1.780010	-0.013505
C	-5.049402	1.225430	-0.077707
C	-4.166626	-0.981910	0.070550
C	-2.635347	0.917482	0.059696
C	-2.897778	-0.493179	0.114493
C	-4.565385	-2.394392	0.080884
C	-3.651879	3.207643	-0.063155
C	-6.187163	1.981398	-0.175485
C	-6.095769	3.408036	-0.221446
C	-4.777514	3.979853	-0.158347
C	-5.636281	-2.814496	-0.718168
C	-3.901799	-3.329442	0.885023
C	-6.020825	-4.147943	-0.725217
C	-4.297942	-4.660774	0.878130
C	-5.352341	-5.074441	0.070534
H	-7.156473	1.499408	-0.223508
H	-4.678182	5.059509	-0.192457
H	-6.157307	-2.097081	-1.341257
H	-3.093184	-3.017127	1.534868
H	-6.844389	-4.465116	-1.355240
H	-3.783229	-5.374732	1.511473
H	-5.655523	-6.115705	0.063838
H	-1.827899	-2.077760	-0.310712
H	-1.750037	3.128225	0.036282
Cu	0.000115	0.000299	0.069099
O	1.419495	-1.336724	0.063775
C	2.635313	-0.917455	0.059631
C	3.743815	-1.780054	-0.013526
C	2.897860	0.493176	0.114554
C	5.049360	-1.225555	-0.077574
C	3.651697	-3.207672	-0.063310
O	1.771822	1.270367	0.217287
C	4.166726	0.981837	0.070677
O	5.205964	0.131951	-0.046128
C	6.187072	-1.981602	-0.175297
O	2.440411	-3.812913	-0.013298
C	4.777284	-3.979963	-0.158442
H	1.828100	2.077835	-0.310703
C	4.565519	2.394315	0.080956
C	6.095580	-3.408231	-0.221375
H	7.156423	-1.499685	-0.223209
H	1.749835	-3.128138	0.036003
H	4.677870	-5.059609	-0.192648
C	5.636218	2.814396	-0.718364
C	3.902103	3.329366	0.885229
O	7.127856	-4.141686	-0.317045
C	6.020731	4.147853	-0.725556
H	6.157105	2.096957	-1.341544
C	4.298214	4.660706	0.878189
H	3.093655	3.017030	1.535275
C	5.352408	5.074362	0.070317
H	6.844134	4.465030	-1.355786
H	3.783649	5.374684	1.511630
H	5.655558	6.115635	0.063498

SCF Energy: -2103.22629897

==> ./AOX-II/PA/bis-/c3c4/vIP <==

O	-5.205907	-0.132080	-0.046343
O	-1.771741	-1.270326	0.217335
O	-2.440646	3.812975	-0.012989
O	-1.419570	1.336928	0.064048
O	-7.128078	4.141435	-0.317140
C	-3.743897	1.780010	-0.013505
C	-5.049402	1.225430	-0.077707
C	-4.166626	-0.981910	0.070550
C	-2.635347	0.917482	0.059696
C	-2.897778	-0.493179	0.114493
C	-4.565385	-2.394392	0.080884
C	-3.651879	3.207643	-0.063155

C	-6.187163	1.981398	-0.175485
C	-6.095769	3.408036	-0.221446
C	-4.777514	3.979853	-0.158347
C	-5.636281	-2.814496	-0.718168
C	-3.901799	-3.329442	0.885023
C	-6.020825	-4.147943	-0.725217
C	-4.297942	-4.660774	0.878130
C	-5.352341	-5.074441	0.070534
H	-7.156473	1.499408	-0.223508
H	-4.678182	5.059509	-0.192457
H	-6.157307	-2.097081	-1.341257
H	-3.093184	-3.017127	1.534868
H	-6.844389	-4.465116	-1.355240
H	-3.783229	-5.374732	1.511473
H	-5.655523	-6.115705	0.063838
H	-1.827899	-2.077760	-0.310712
H	-1.750037	3.128225	0.036282
Cu	0.000115	0.000299	0.069099
O	1.419495	-1.336724	0.063775
C	2.635313	-0.917455	0.059631
C	3.743815	-1.780054	-0.013526
C	2.897860	0.493176	0.114554
C	5.049360	-1.225555	-0.077574
C	3.651697	-3.207672	-0.063310
O	1.771822	1.270367	0.217287
C	4.166726	0.981837	0.070677
O	5.205964	0.131951	-0.046128
C	6.187072	-1.981602	-0.175297
O	2.440411	-3.812913	-0.013298
C	4.777284	-3.979963	-0.158442
H	1.828100	2.077835	-0.310703
C	4.565519	2.394315	0.080956
C	6.095580	-3.408231	-0.221375
H	7.156423	-1.499685	-0.223209
H	1.749835	-3.128138	0.036003
H	4.677870	-5.059609	-0.192648
C	5.636218	2.814396	-0.718364
C	3.902103	3.329366	0.885229
O	7.127856	-4.141686	-0.317045
C	6.020731	4.147853	-0.725556
H	6.157105	2.096957	-1.341544
C	4.298214	4.660706	0.878189
H	3.093655	3.017030	1.535275
C	5.352408	5.074362	0.070317
H	6.844134	4.465030	-1.355786
H	3.783649	5.374684	1.511630
H	5.655558	6.115635	0.063498

SCF Energy: -2102.86475638

==> ./AOX-II/PA/bis-/c3c4 <==

O	-5.205907	-0.132080	-0.046343
O	-1.771741	-1.270326	0.217335
O	-2.440646	3.812975	-0.012989
O	-1.419570	1.336928	0.064048
O	-7.128078	4.141435	-0.317140
C	-3.743897	1.780010	-0.013505
C	-5.049402	1.225430	-0.077707
C	-4.166626	-0.981910	0.070550
C	-2.635347	0.917482	0.059696
C	-2.897778	-0.493179	0.114493
C	-4.565385	-2.394392	0.080884
C	-3.651879	3.207643	-0.063155
C	-6.187163	1.981398	-0.175485
C	-6.095769	3.408036	-0.221446
C	-4.777514	3.979853	-0.158347
C	-5.636281	-2.814496	-0.718168
C	-3.901799	-3.329442	0.885023
C	-6.020825	-4.147943	-0.725217
C	-4.297942	-4.660774	0.878130
C	-5.352341	-5.074441	0.070534
H	-7.156473	1.499408	-0.223508
H	-4.678182	5.059509	-0.192457
H	-6.157307	-2.097081	-1.341257
H	-3.093184	-3.017127	1.534868
H	-6.844389	-4.465116	-1.355240
H	-3.783229	-5.374732	1.511473
H	-5.655523	-6.115705	0.063838
H	-1.827899	-2.077760	-0.310712
H	-1.750037	3.128225	0.036282
Cu	0.000115	0.000299	0.069099
O	1.419495	-1.336724	0.063775
C	2.635313	-0.917455	0.059631
C	3.743815	-1.780054	-0.013526
C	2.897860	0.493176	0.114554
C	5.049360	-1.225555	-0.077574
C	3.651697	-3.207672	-0.063310
O	1.771822	1.270367	0.217287
C	4.166726	0.981837	0.070677
O	5.205964	0.131951	-0.046128
C	6.187072	-1.981602	-0.175297
O	2.440411	-3.812913	-0.013298
C	4.777284	-3.979963	-0.158442
H	1.828100	2.077835	-0.310703
C	4.565519	2.394315	0.080956
C	6.095580	-3.408231	-0.221375
H	7.156423	-1.499685	-0.223209
H	1.749835	-3.128138	0.036003
H	4.677870	-5.059609	-0.192648
C	5.636218	2.814396	-0.718364
C	3.902103	3.329366	0.885229
O	7.127856	-4.141686	-0.317045
C	6.020731	4.147853	-0.725556
H	6.157105	2.096957	-1.341544
C	4.298214	4.660706	0.878189

H	3.093655	3.017030	1.535275
C	5.352408	5.074362	0.070317
H	6.844134	4.465030	-1.355786
H	3.783649	5.374684	1.511630
H	5.655558	6.115635	0.063498

SCF Energy: -2103.07065001
Sum of electronic and zero-point Energies= -2102.650052
Sum of electronic and thermal Energies= -2102.616342
Sum of electronic and thermal Enthalpies= -2102.615398
Sum of electronic and thermal Free Energies= -2102.718310

==> ./AOX-II/PA/bis-/c4c5 <==

O	5.495758	-1.142610	0.090141
O	3.946495	2.104436	0.085108
O	0.783551	-1.980737	0.103954
O	1.849983	0.603294	0.124390
O	3.894154	-5.542354	0.089061
C	3.111331	-1.458961	0.104920
C	4.433115	-1.985240	0.093732
C	5.375117	0.205813	0.088977
C	2.942146	-0.056421	0.118861
C	4.133277	0.764731	0.111473
C	6.668688	0.891002	0.019011
C	2.070393	-2.435629	0.100388
C	4.721953	-3.328697	0.090216
C	3.673988	-4.292402	0.092125
C	2.329720	-3.774575	0.094859
C	7.761455	0.237106	-0.569247
C	6.850562	2.181658	0.533973
C	8.996620	0.863961	-0.652166
C	8.092139	2.799293	0.454112
C	9.167308	2.147665	-0.141693
H	5.755654	-3.653869	0.084380
H	1.502978	-4.478419	0.092474
H	7.638665	-0.761331	-0.971048
H	6.029746	2.697864	1.012683
H	9.828427	0.347590	-1.118774
H	8.218882	3.795123	0.864589
H	10.133643	2.635995	-0.205957
H	2.985882	2.241409	0.056546
H	0.150887	-2.709947	0.085232
Cu	-0.000004	-0.000028	0.000022
O	-1.849978	-0.603340	-0.124156
C	-2.942157	0.056396	-0.118848
C	-3.111311	1.458929	-0.105107
C	-4.133274	-0.764770	-0.111397
C	-4.433091	1.985219	-0.093993
C	-2.070345	2.435567	-0.100635
O	-3.946476	-2.104453	-0.084858
C	-5.375124	-0.205838	-0.088960
O	-5.495716	1.142591	-0.090359
C	-4.721905	3.328681	-0.090585
O	-0.783562	1.980603	-0.104219
C	-2.329650	3.774513	-0.095186
H	-2.985860	-2.241405	-0.056163
C	-6.668721	-0.890949	-0.018831
C	-3.673912	4.292363	-0.092574
H	-5.755595	3.653892	-0.084815
H	-0.150775	2.709696	-0.085134
H	-1.502901	4.478349	-0.092835
C	-7.761647	-0.236575	0.568617
C	-6.850493	-2.182033	-0.532785
O	-3.894049	5.542317	-0.089773
C	-8.996836	-0.863346	0.651729
H	-7.638962	0.762182	0.969647
C	-8.092097	-2.799587	-0.452734
H	-6.029566	-2.698649	-1.010853
C	-9.167413	-2.147468	0.142266
H	-9.828755	-0.346590	1.117710
H	-8.218739	-3.795755	-0.862422
H	-10.133770	-2.635732	0.206691

SCF Energy: -2103.07210793
Sum of electronic and zero-point Energies= -2102.652418
Sum of electronic and thermal Energies= -2102.617761
Sum of electronic and thermal Enthalpies= -2102.616817
Sum of electronic and thermal Free Energies= -2102.723513

==> ./AOX-II/PA/bis-/c4c5/aEA <==

O	5.465592	-1.148564	-0.014110
O	3.876806	2.028220	0.586418
O	0.921278	-2.166890	1.084782
O	1.858115	0.447419	0.881551
O	3.993543	-5.593984	-0.051977
C	3.148659	-1.556399	0.510112
C	4.441873	-2.026187	0.156516
C	5.324643	0.193528	0.137531
C	2.942751	-0.156418	0.635336
C	4.105417	0.700259	0.459481
C	6.564664	0.928203	-0.131518
C	2.157190	-2.570973	0.696067
C	4.750847	-3.353630	-0.034405
C	3.752322	-4.355071	0.124572
C	2.445772	-3.897198	0.506608
C	7.531634	0.366254	-0.978181
C	6.819483	2.180707	0.443836
C	8.710871	1.045013	-1.251975

C	8.005077	2.851155	0.170639
C	8.952831	2.290793	-0.680025
H	5.761555	-3.634493	-0.307691
H	1.664819	-4.636443	0.660961
H	7.353393	-0.601708	-1.430650
H	6.100457	2.625591	1.118193
H	9.443189	0.598826	-1.916157
H	8.189491	3.815922	0.630805
H	9.875409	2.819729	-0.893664
H	2.921801	2.098029	0.762557
H	0.348143	-2.938187	1.165375
Cu	0.027174	0.154167	0.090503
O	-1.758022	-0.259839	-0.760062
C	-2.891607	0.262013	-0.551096
C	-3.189728	1.633008	-0.328226
C	-4.010754	-0.667268	-0.535877
C	-4.531707	2.003260	-0.045882
C	-2.247954	2.709153	-0.347479
O	-3.694199	-1.966000	-0.748750
C	-5.278713	-0.254853	-0.273155
O	-5.511369	1.060945	-0.028667
C	-4.931749	3.292887	0.221062
O	-0.962223	2.401380	-0.656054
C	-2.626358	3.999026	-0.081761
H	-2.726390	-1.969476	-0.854586
C	-6.488971	-1.075379	-0.164166
C	-3.984310	4.355240	0.222238
H	-5.975450	3.497455	0.430699
H	-0.426684	3.201889	-0.613022
H	-1.879304	4.787607	-0.111201
C	-7.546700	-0.644294	0.649952
C	-6.625624	-2.282868	-0.862637
O	-4.310534	5.561664	0.470410
C	-8.700135	-1.406606	0.771726
H	-7.460213	0.286777	1.196782
C	-7.785790	-3.037218	-0.741321
H	-5.833855	-2.626565	-1.514339
C	-8.825006	-2.606670	0.077589
H	-9.504187	-1.061619	1.412819
H	-7.878049	-3.965148	-1.295291
H	-9.727639	-3.200653	0.172122

SCF Energy: -2103.23765425

Sum of electronic and zero-point Energies= -2102.819691

Sum of electronic and thermal Energies= -2102.785328

Sum of electronic and thermal Enthalpies= -2102.784384

Sum of electronic and thermal Free Energies= -2102.891445

==> ./AOX-II/PA/bis-/c4c5/HAT/c3 <==

O	5.561235	1.138755	0.089310
O	4.031216	-2.076392	-0.450086
O	0.964722	2.058215	-0.877437
O	1.968105	-0.550315	-0.681100
O	3.991884	5.548026	0.176953
C	3.218981	1.490765	-0.360135
C	4.511909	1.990946	-0.047343
C	5.448140	-0.206065	-0.057377
C	3.041856	0.084958	-0.473808
C	4.230498	-0.742910	-0.332676
C	6.716657	-0.908606	0.159949
C	2.202679	2.485419	-0.518314
C	4.797046	3.324965	0.134629
C	3.773098	4.304481	0.007093
C	2.466407	3.817257	-0.336567
C	7.704260	-0.320267	0.963792
C	6.979704	-2.153904	-0.427184
C	8.912838	-0.966109	1.183872
C	8.194446	-2.791160	-0.208093
C	9.163488	-2.204540	0.599837
H	5.809720	3.627484	0.375451
H	1.664852	4.538399	-0.469034
H	7.519917	0.642708	1.424425
H	6.243664	-2.618484	-1.069128
H	9.661533	-0.499618	1.815027
H	8.384936	-3.750340	-0.677375
H	10.109185	-2.707465	0.770932
H	3.073913	-2.171170	-0.599138
H	0.369147	2.814450	-0.938201
Cu	0.038482	-0.170601	-0.220017
O	-1.824511	0.331282	0.360691
C	-2.945242	-0.190593	0.199592
C	-3.298997	-1.558248	0.205958
C	-4.064392	0.776943	-0.106459
C	-4.648315	-1.951764	0.008736
C	-2.369853	-2.656508	0.314229
O	-3.841716	1.906474	-0.472406
C	-5.479383	0.271223	0.019495
O	-5.668121	-1.003190	-0.009348
C	-5.082279	-3.228462	-0.119491
O	-1.092930	-2.331501	0.570199
C	-2.762962	-3.961035	0.163522
C	-6.633926	1.093435	0.134374
C	-4.126335	-4.322528	-0.069537
H	-6.135259	-3.440953	-0.254563
H	-0.551654	-3.131193	0.590550
H	-2.023759	-4.751673	0.243770
C	-7.913424	0.508528	-0.030796
C	-6.532161	2.466514	0.459963
O	-4.506210	-5.508978	-0.208035
C	-9.045574	1.281589	0.097189
H	-7.996283	-0.540872	-0.281475
C	-7.678947	3.222565	0.602280

H -5.567333 2.920994 0.627490
C -8.929864 2.637702 0.415026
H -10.023172 0.838280 -0.046559
H -7.602126 4.270157 0.865637
H -9.824768 3.240180 0.522211

SCF Energy: -2102.42302525
Sum of electronic and zero-point Energies= -2102.015923
Sum of electronic and thermal Energies= -2101.980925
Sum of electronic and thermal Enthalpies= -2101.979981
Sum of electronic and thermal Free Energies= -2102.088042

==> ./AOX-II/PA/bis-/c4c5/HAT/c5 <==
O -5.307262 -1.109554 -0.460887
O -3.877492 2.054220 0.488398
O -0.810543 -2.089753 0.871571
O -1.843761 0.511159 0.844242
O -3.730193 -5.516437 -0.584835
C -3.025681 -1.492708 0.202525
C -4.280875 -1.973962 -0.258600
C -5.214167 0.223575 -0.231861
C -2.876403 -0.103296 0.437207
C -4.035667 0.739949 0.211849
C -6.471063 0.933368 -0.487761
C -2.022827 -2.491901 0.386817
C -4.541864 -3.296847 -0.529843
C -3.532009 -4.283026 -0.350751
C -2.256555 -3.811285 0.121874
C -7.684682 0.232374 -0.427081
C -6.496442 2.297911 -0.808749
C -8.887562 0.881488 -0.667534
C -7.703804 2.938697 -1.055420
C -8.903019 2.237275 -0.982503
H -5.525693 -3.587204 -0.879758
H -1.464622 -4.536289 0.283363
H -7.685177 -0.822772 -0.182530
H -5.574245 2.857235 -0.882464
H -9.816615 0.325029 -0.608181
H -7.704324 3.992851 -1.310532
H -9.843600 2.742992 -1.172413
H -2.960052 2.146921 0.796318
H -0.185835 -2.825905 0.880397
Cu 0.058138 -0.139563 0.892794
O 2.005141 -0.844227 0.919764
C 2.977179 -0.110676 0.595424
C 2.982354 1.299386 0.483487
C 4.235136 -0.772763 0.327864
C 4.119187 1.945328 0.009052
C 1.816794 2.135742 0.779781
O 4.267428 -2.087440 0.478723
C 5.349175 -0.062862 -0.102758
O 5.248263 1.261077 -0.260848
C 4.156962 3.311745 -0.242581
O 0.828568 1.632362 1.400495
C 1.839589 3.503627 0.434537
H 3.361990 -2.370261 0.705507
C 6.637592 -0.608462 -0.503537
C 3.005557 4.159199 -0.038559
H 5.066693 3.744708 -0.643214
H 0.953012 4.090066 0.648876
C 7.410465 0.087760 -1.448130
C 7.134448 -1.802211 0.043104
O 3.065878 5.396712 -0.290471
C 8.641701 -0.407240 -1.844087
H 7.034129 1.006460 -1.880803
C 8.374062 -2.282757 -0.352403
H 6.568840 -2.339908 0.790934
C 9.127407 -1.593333 -1.297819
H 9.224088 0.131159 -2.583049
H 8.754788 -3.198974 0.084299
H 10.092475 -1.978285 -1.608273

SCF Energy: -2102.40668746
Sum of electronic and zero-point Energies= -2102.000108
Sum of electronic and thermal Energies= -2101.965862
Sum of electronic and thermal Enthalpies= -2101.964918
Sum of electronic and thermal Free Energies= -2102.070298

==> ./AOX-II/PA/bis-/c4c5/aIP <==
O 5.508119 -1.131015 0.087533
O 3.947511 2.086723 0.084856
O 0.801450 -1.992529 0.098399
O 1.853665 0.595883 0.122258
O 3.934956 -5.522853 0.078632
C 3.124553 -1.464317 0.101100
C 4.447810 -1.973266 0.089032
C 5.390214 0.206476 0.087566
C 2.944147 -0.060227 0.116546
C 4.131747 0.761620 0.110053
C 6.672962 0.899509 0.019306
C 2.078928 -2.450536 0.094736
C 4.746473 -3.314450 0.082876
C 3.691010 -4.290247 0.083777
C 2.341718 -3.785349 0.087656
C 7.783930 0.227470 -0.516528
C 6.833009 2.213461 0.485018
C 9.014883 0.859267 -0.596718
C 8.072304 2.833531 0.410158

C	9.164133	2.164230	-0.133982
H	5.779108	-3.639701	0.075179
H	1.521919	-4.495887	0.084504
H	7.675986	-0.786162	-0.882033
H	5.999579	2.743499	0.923737
H	9.860655	0.331968	-1.023598
H	8.184754	3.845418	0.783005
H	10.128576	2.656266	-0.195813
H	2.985789	2.232171	0.068083
H	0.163791	-2.718495	0.081154
Cu	-0.000031	-0.000103	-0.001739
O	-1.853781	-0.596010	-0.125587
C	-2.944240	0.060137	-0.118436
C	-3.124559	1.464232	-0.102624
C	-4.131870	-0.761657	-0.110656
C	-4.447772	1.973244	-0.089143
C	-2.078883	2.450401	-0.097303
O	-3.947648	-2.086766	-0.085892
C	-5.390287	-0.206454	-0.086525
O	-5.508112	1.131043	-0.086338
C	-4.746370	3.314443	-0.082839
O	-0.801432	1.992332	-0.101984
C	-2.341602	3.785227	-0.090160
H	-2.985905	-2.232251	-0.070702
C	-6.672994	-0.899395	-0.016627
C	-3.690865	4.290191	-0.085032
H	-5.778983	3.639740	-0.074076
H	-0.163730	2.718263	-0.084766
H	-1.521767	4.495726	-0.087836
C	-7.783317	-0.227102	0.520236
C	-6.833671	-2.213495	-0.481722
O	-3.934747	5.522809	-0.079877
C	-9.014225	-0.858774	0.602023
H	-7.674894	0.786638	0.885295
C	-8.072933	-2.833438	-0.405262
H	-6.000779	-2.743743	-0.921198
C	-9.164099	-2.163877	0.139884
H	-9.859467	-0.331272	1.029702
H	-8.185875	-3.845441	-0.777647
H	-10.128509	-2.655820	0.202969

SCF Energy: -2102.87145155

Sum of electronic and zero-point Energies= -2102.451090

Sum of electronic and thermal Energies= -2102.416605

Sum of electronic and thermal Enthalpies= -2102.415661

Sum of electronic and thermal Free Energies= -2102.521050

==> ./AOX-II/PA/bis-/c4c5/vEA <==

O	5.495758	-1.142610	0.090141
O	3.946495	2.104436	0.085108
O	0.783551	-1.980737	0.103954
O	1.849983	0.603294	0.124390
O	3.894154	-5.542354	0.089061
C	3.111331	-1.458961	0.104920
C	4.433115	-1.985240	0.093732
C	5.375117	0.205813	0.088977
C	2.942146	-0.056421	0.118861
C	4.133277	0.764731	0.111473
C	6.668688	0.891002	0.019011
C	2.070393	-2.435629	0.100388
C	4.721953	-3.328697	0.090216
C	3.673988	-4.292402	0.092125
C	2.329720	-3.774575	0.094859
C	7.761455	0.237106	-0.569247
C	6.850562	2.181658	0.533973
C	8.996620	0.863961	-0.652166
C	8.092139	2.799293	0.454112
C	9.167308	2.147665	-0.141693
H	5.755654	-3.653869	0.084380
H	1.502978	-4.478419	0.092474
H	7.638665	-0.761331	-0.971048
H	6.029746	2.697864	1.012683
H	9.828427	0.347590	-1.118774
H	8.218882	3.795123	0.864589
H	10.133643	2.635995	-0.205957
H	2.985882	2.241409	0.056546
H	0.150887	-2.709947	0.085232
Cu	-0.000004	-0.000028	0.000022
O	-1.849978	-0.603340	-0.124156
C	-2.942157	0.056396	-0.118848
C	-3.111311	1.458929	-0.105107
C	-4.133274	-0.764770	-0.111397
C	-4.433091	1.985219	-0.093993
C	-2.070345	2.435567	-0.100635
O	-3.946476	-2.104453	-0.084858
C	-5.375124	-0.205838	-0.088960
O	-5.495716	1.142591	-0.090359
C	-4.721905	3.328681	-0.090585
O	-0.783562	1.980603	-0.104219
C	-2.329650	3.774513	-0.095186
H	-2.985860	-2.241405	-0.056163
C	-6.668721	-0.890949	-0.018831
C	-3.673912	4.292363	-0.092574
H	-5.755595	3.653892	-0.084815
H	-0.150775	2.709696	-0.085134
H	-1.502901	4.478349	-0.092835
C	-7.761647	-0.236575	0.568617
C	-6.850493	-2.182033	-0.532785
O	-3.894049	5.542317	-0.089773
C	-8.996836	-0.863346	0.651729
H	-7.638962	0.762182	0.969647
C	-8.092097	-2.799587	-0.452734

H	-6.029566	-2.698649	-1.010853
C	-9.167413	-2.147468	0.142266
H	-9.828755	-0.346590	1.117710
H	-8.218739	-3.795755	-0.862422
H	-10.133770	-2.635732	0.206691

SCF Energy: -2103.22346172

==> ./AOX-II/PA/bis-/c4c5/vIP <==

O	5.495758	-1.142610	0.090141
O	3.946495	2.104436	0.085108
O	0.783551	-1.980737	0.103954
O	1.849983	0.603294	0.124390
O	3.894154	-5.542354	0.089061
C	3.111331	-1.458961	0.104920
C	4.433115	-1.985240	0.093732
C	5.375117	0.205813	0.088977
C	2.942146	-0.056421	0.118861
C	4.133277	0.764731	0.111473
C	6.668688	0.891002	0.019011
C	2.070393	-2.435629	0.100388
C	4.721953	-3.328697	0.090216
C	3.673988	-4.292402	0.092125
C	2.329720	-3.774575	0.094859
C	7.761455	0.237106	-0.569247
C	6.850562	2.181658	0.533973
C	8.996620	0.863961	-0.652166
C	8.092139	2.799293	0.454112
C	9.167308	2.147665	-0.141693
H	5.755654	-3.653869	0.084380
H	1.502978	-4.478419	0.092474
H	7.638665	-0.761331	-0.971048
H	6.029746	2.697864	1.012683
H	9.828427	0.347590	-1.118774
H	8.218882	3.795123	0.864589
H	10.133643	2.635995	-0.205957
H	2.985882	2.241409	0.056546
H	0.150887	-2.709947	0.085232
Cu	-0.000004	-0.000028	0.000022
O	-1.849978	-0.603340	-0.124156
C	-2.942157	0.056396	-0.118848
C	-3.111311	1.458929	-0.105107
C	-4.133274	-0.764770	-0.111397
C	-4.433091	1.985219	-0.093993
C	-2.070345	2.435567	-0.100635
O	-3.946476	-2.104453	-0.084858
C	-5.375124	-0.205838	-0.088960
O	-5.495716	1.142591	-0.090359
C	-4.721905	3.328681	-0.090585
O	-0.783562	1.980603	-0.104219
C	-2.329650	3.774513	-0.095186
H	-2.985860	-2.241405	-0.056163
C	-6.668721	-0.890949	-0.018831
C	-3.673912	4.292363	-0.092574
H	-5.755595	3.653892	-0.084815
H	-0.150775	2.709696	-0.085134
H	-1.502901	4.478349	-0.092835
C	-7.761647	-0.236575	0.568617
C	-6.850493	-2.182033	-0.532785
O	-3.894049	5.542317	-0.089773
C	-8.996836	-0.863346	0.651729
H	-7.638962	0.762182	0.969647
C	-8.092097	-2.799587	-0.452734
H	-6.029566	-2.698649	-1.010853
C	-9.167413	-2.147468	0.142266
H	-9.828755	-0.346590	1.117710
H	-8.218739	-3.795755	-0.862422
H	-10.133770	-2.635732	0.206691

SCF Energy: -2102.86942162

==> ./AOX-II/PA/bis-/c4c5/RAF/c6p <==

O	-5.756989	-1.141087	0.107444
O	-4.175942	2.046415	-0.451179
O	-1.211135	-2.142642	-1.004806
O	-2.154468	0.478544	-0.760527
O	-4.272642	-5.581616	0.119783
C	-3.437448	-1.537525	-0.416484
C	-4.729755	-2.013517	-0.066618
C	-5.620058	0.202532	-0.033111
C	-3.234245	-0.135068	-0.524278
C	-4.400047	0.716358	-0.339393
C	-6.866914	0.930000	0.224120
C	-2.445840	-2.549784	-0.612546
C	-5.035374	-3.343006	0.115706
C	-4.035057	-4.341288	-0.049777
C	-2.730342	-3.877610	-0.431483
C	-7.842974	0.358849	1.053889
C	-7.120745	2.183014	-0.350554
C	-9.030640	1.029163	1.311833
C	-8.314687	2.844887	-0.093485
C	-9.271864	2.275283	0.740342
H	-6.045690	-3.627702	0.386404
H	-1.947894	-4.613683	-0.593030
H	-7.665762	-0.609927	1.505055
H	-6.394305	2.634744	-1.012312
H	-9.770394	0.575825	1.962790
H	-8.498331	3.809936	-0.553371
H	-10.201207	2.797347	0.941161
H	-3.221376	2.122392	-0.626141
H	-0.635367	-2.911581	-1.089324
Cu	-0.216581	0.109710	-0.369077
O	1.726747	-0.215043	0.041788
C	2.829119	0.388873	0.093505

C	3.115046	1.767766	0.084991
C	3.988575	-0.518333	0.184818
C	4.479636	2.195815	0.111495
C	2.135427	2.821038	0.048997
O	3.687929	-1.799991	0.195965
C	5.285172	-0.054241	0.201109
O	5.490975	1.287841	0.153601
C	4.860763	3.508965	0.093041
O	0.838496	2.441484	0.050393
C	2.501579	4.136458	0.025454
H	2.708674	-1.844487	0.125847
C	6.476417	-0.831730	0.136587
C	3.880475	4.549598	0.043447
H	5.913739	3.763447	0.110160
H	0.270702	3.220912	0.024868
H	1.733379	4.903546	0.000871
C	7.675619	-0.220999	-0.235300
C	6.466579	-2.317229	0.385851
O	4.199983	5.772052	0.019085
C	8.836463	-0.958110	-0.369474
H	7.700983	0.842272	-0.426534
C	7.782447	-2.990414	0.294561
C	8.898143	-2.353689	-0.120046
H	9.742161	-0.441820	-0.667746
H	7.784901	-4.043737	0.549282
H	9.839168	-2.878353	-0.221009
H	5.934320	-2.728015	-0.501646
O	5.825469	-2.723556	1.569711
H	4.872965	-2.728233	1.414680

SCF Energy: -2178.84189111
Sum of electronic and zero-point Energies= -2178.407551
Sum of electronic and thermal Energies= -2178.371110
Sum of electronic and thermal Enthalpies= -2178.370165
Sum of electronic and thermal Free Energies= -2178.481138

==> ./AOX-II/PA/bis-/c4c5/RAF/c6 <==

O	-5.655603	-1.171855	0.105889
O	-4.042394	2.008664	-0.395197
O	-1.157955	-2.204318	-1.165534
O	-2.057022	0.421804	-0.827157
O	-4.243009	-5.632088	-0.074313
C	-3.359410	-1.585142	-0.501719
C	-4.648467	-2.053543	-0.129367
C	-5.500987	0.173460	0.008810
C	-3.138796	-0.183276	-0.572904
C	-4.283592	0.678860	-0.321512
C	-6.725160	0.911137	0.336140
C	-2.389177	-2.605293	-0.756948
C	-4.970423	-3.383558	0.016740
C	-3.991127	-4.390626	-0.210065
C	-2.690148	-3.933863	-0.612563
C	-7.677039	0.329899	1.186659
C	-6.980515	2.184800	-0.190308
C	-8.842182	1.010315	1.511910
C	-8.152173	2.856857	0.133940
C	-9.084936	2.276889	0.988046
H	-5.976804	-3.661829	0.307985
H	-1.923615	-4.675695	-0.817939
H	-7.498531	-0.654814	1.601315
H	-6.273453	2.645049	-0.867089
H	-9.563064	0.548843	2.178132
H	-8.337705	3.838286	-0.288966
H	-9.996752	2.806913	1.241450
H	-3.093256	2.077275	-0.599473
H	-0.595345	-2.978379	-1.284132
Cu	-0.140405	0.016294	-0.400208
O	1.728590	-0.357356	0.222382
C	2.898520	0.073354	0.199103
C	3.308560	1.444948	0.041001
C	3.955287	-0.917281	0.317045
C	4.715044	1.760123	0.040704
C	2.438489	2.517052	-0.057236
O	3.550951	-2.200118	0.417544
C	5.265809	-0.561216	0.284470
O	5.603043	0.759260	0.150792
C	5.248374	3.023790	0.002312
O	1.155882	2.403486	0.119000
C	2.946627	3.867865	-0.436499
H	2.579939	-2.175505	0.374541
C	6.441303	-1.429732	0.341093
C	4.398380	4.159552	-0.059334
H	6.315501	3.156461	0.127759
H	0.755151	3.292569	0.047721
H	3.003534	3.799306	-1.538971
C	7.654429	-0.991319	-0.211637
C	6.391277	-2.694181	0.945548
O	4.755727	5.330954	0.077120
C	8.778964	-1.802945	-0.174498
H	7.712999	-0.016551	-0.679687
C	7.524151	-3.495886	0.986672
H	5.475280	-3.046064	1.399006
C	8.719056	-3.058137	0.424228
H	9.705938	-1.452475	-0.614857
H	7.471367	-4.467457	1.465461
H	9.599980	-3.690111	0.454667
O	2.018725	4.838173	-0.048877
H	1.954266	5.511390	-0.733038

SCF Energy: -2178.88917864
Sum of electronic and zero-point Energies= -2178.454374

Sum of electronic and thermal Energies= -2178.417548
Sum of electronic and thermal Enthalpies= -2178.416604
Sum of electronic and thermal Free Energies= -2178.530260

==> ./AOX-II/PA/bis-/c4c5/RAF/c1p <==

O	5.354036	-0.972907	0.055217
O	3.967552	2.305744	0.600066
O	0.613314	-1.508673	-0.325605
O	1.814599	0.981388	0.126750
O	3.551933	-5.172856	-0.953681
C	2.956614	-1.129630	-0.150737
C	4.247319	-1.723645	-0.185027
C	5.303125	0.354166	0.337168
C	2.856393	0.265260	0.101521
C	4.096885	0.979042	0.366412
C	6.630893	0.942720	0.538458
C	1.866355	-2.024761	-0.390435
C	4.473430	-3.054816	-0.450460
C	3.384548	-3.934260	-0.706461
C	2.072317	-3.352059	-0.660702
C	7.751522	0.341022	-0.052903
C	6.817690	2.088974	1.323346
C	9.018456	0.879164	0.124984
C	8.089670	2.617756	1.502814
C	9.193638	2.020044	0.902988
H	5.489146	-3.433337	-0.458824
H	1.215180	-3.996758	-0.834031
H	7.627281	-0.546862	-0.660893
H	5.973554	2.559307	1.808870
H	9.872114	0.404857	-0.347006
H	8.217154	3.500204	2.120477
H	10.184543	2.438399	1.042943
H	3.012836	2.478707	0.517889
H	-0.027482	-2.199651	-0.530853
Cu	0.026282	0.965276	-0.772643
O	-1.774670	1.317314	-1.612019
C	-2.873207	1.056555	-1.046700
C	-3.809281	1.991030	-0.528217
C	-3.241875	-0.345561	-0.920647
C	-4.998535	1.519462	0.096282
C	-3.659112	3.415327	-0.557867
O	-2.384951	-1.284514	-1.402104
C	-4.378664	-0.726720	-0.307669
O	-5.234543	0.175106	0.200205
C	-5.960762	2.334299	0.637706
O	-2.538495	3.910253	-1.140038
C	-4.609292	4.244717	-0.022260
H	-1.595193	-0.821986	-1.729929
C	-3.960537	-2.922872	0.860982
C	-5.805151	3.750902	0.600997
H	-6.837275	1.896802	1.101142
H	-2.565473	4.873052	-1.097790
H	-4.457524	5.319405	-0.066384
C	-4.540800	-3.706708	1.859576
C	-4.811066	-2.162946	-0.109348
O	-6.671201	4.541027	1.093083
C	-5.908483	-3.782615	1.944310
H	-3.896255	-4.243595	2.543198
C	-6.273824	-2.306021	0.092623
H	-4.615516	-2.676678	-1.072001
C	-6.786493	-3.100146	1.050954
H	-6.343121	-4.397997	2.724186
H	-6.911606	-1.759567	-0.591153
H	-7.855994	-3.219163	1.163478
O	-2.668015	-2.909209	0.766728
H	-2.335633	-2.395600	-0.005633

SCF Energy: -2178.86086419
Sum of electronic and zero-point Energies= -2178.424801
Sum of electronic and thermal Energies= -2178.389045
Sum of electronic and thermal Enthalpies= -2178.388101
Sum of electronic and thermal Free Energies= -2178.495400

==> ./AOX-II/PA/bis-/c4c5/RAF/c5 <==

O	5.525546	1.126533	0.025738
O	3.823136	-1.973816	-0.664529
O	0.959571	2.309811	-0.785752
O	1.837714	-0.324201	-0.783029
O	4.168155	5.596840	0.381334
C	3.199236	1.618317	-0.359616
C	4.518701	2.037118	-0.042839
C	5.339762	-0.200721	-0.193845
C	2.949239	0.233886	-0.555265
C	4.093000	-0.660182	-0.479170
C	6.571026	-0.981507	-0.038888
C	2.227033	2.662804	-0.435180
C	4.868869	3.344535	0.206728
C	3.889321	4.375674	0.151909
C	2.552957	3.968928	-0.187442
C	7.585790	-0.516708	0.810235
C	6.768907	-2.183376	-0.731790
C	8.758079	-1.242378	0.971296
C	7.947580	-2.900674	-0.571153
C	8.944119	-2.437466	0.282263
H	5.898207	3.587578	0.444167
H	1.782833	4.731828	-0.259484
H	7.451020	0.411377	1.352582
H	6.011204	-2.550975	-1.410643
H	9.528897	-0.872475	1.638744

H	8.088485	-3.824600	-1.121552
H	9.861545	-3.002362	0.407570
H	2.859361	-2.013707	-0.794818
H	0.394998	3.091722	-0.775996
Cu	-0.030699	0.271295	-0.183071
O	-1.831063	0.753107	0.683214
C	-2.896839	0.103404	0.510948
C	-3.018701	-1.304985	0.412868
C	-4.127605	0.856956	0.377583
C	-4.249588	-1.865235	0.155257
C	-1.844554	-2.236377	0.562817
O	-4.012371	2.186633	0.448883
C	-5.330011	0.235900	0.154321
O	-5.354197	-1.113402	0.045723
C	-4.458264	-3.247385	-0.021232
O	-0.921570	-1.888566	-0.457123
C	-2.157174	-3.678817	0.361516
H	-3.064962	2.367618	0.585369
C	-6.641912	0.852535	-0.031290
C	-3.416595	-4.219501	0.022382
H	-5.474540	-3.577501	-0.210068
H	-0.186225	-2.518308	-0.455377
H	-1.310854	-4.350848	0.486823
C	-7.622077	0.179127	-0.776013
C	-6.950841	2.100441	0.528266
O	-3.615154	-5.455620	-0.181967
C	-8.872902	0.747401	-0.965494
H	-7.398479	-0.784732	-1.216682
C	-8.208527	2.657504	0.340444
H	-6.218793	2.627361	1.124683
C	-9.171116	1.988112	-0.408631
H	-9.617163	0.219863	-1.551708
H	-8.437292	3.618862	0.786916
H	-10.150504	2.429914	-0.556384
O	-1.319653	-2.024492	1.846464
H	-0.689864	-2.724796	2.057354

SCF Energy: -2178.82361150

Sum of electronic and zero-point Energies= -2178.389077

Sum of electronic and thermal Energies= -2178.352836

Sum of electronic and thermal Enthalpies= -2178.351892

Sum of electronic and thermal Free Energies= -2178.460776

==> ./AOX-II/PA/bis-/c4c5/RAF/c2 <==

O	-5.633552	-1.066226	-0.155024
O	-3.880917	2.014835	0.487906
O	-1.072383	-2.309972	0.589194
O	-1.912020	0.350730	0.550836
O	-4.353805	-5.561155	-0.462853
C	-3.305232	-1.584466	0.184575
C	-4.639177	-1.989616	-0.088920
C	-5.427612	0.262628	0.035153
C	-3.031626	-0.199277	0.342049
C	-4.166505	0.709114	0.276275
C	-6.668544	1.041864	-0.024323
C	-2.349674	-2.645112	0.266225
C	-5.015624	-3.295448	-0.307100
C	-4.051473	-4.341063	-0.257662
C	-2.703291	-3.950122	0.049361
C	-7.892697	0.412334	0.246799
C	-6.671872	2.404361	-0.354895
C	-9.080839	1.128295	0.202886
C	-7.865800	3.112757	-0.403500
C	-9.073371	2.481901	-0.121460
H	-6.054028	-3.525256	-0.516732
H	-1.944831	-4.724665	0.123368
H	-7.911317	-0.640256	0.501477
H	-5.745199	2.909892	-0.588828
H	-10.016346	0.625835	0.424036
H	-7.850105	4.164547	-0.667949
H	-10.002498	3.040427	-0.156370
H	-2.920050	2.036396	0.641899
H	-0.525787	-3.104220	0.586403
Cu	-0.039531	-0.175441	-0.034235
O	1.823237	-0.427393	-0.752062
C	2.837910	0.229734	-0.371504
C	3.076760	1.607148	-0.295520
C	3.911613	-0.615640	0.152707
C	4.377386	2.089354	0.098740
C	2.065275	2.612235	-0.545141
O	3.680888	-1.854369	0.228612
C	5.233947	-0.096224	0.672096
O	5.426677	1.225836	0.217858
C	4.638446	3.406517	0.275649
O	0.871444	2.150548	-0.955530
C	2.315936	3.937855	-0.357898
H	2.765032	-1.992981	-0.133484
C	6.392004	-0.927436	0.145956
C	3.610740	4.405626	0.064046
H	5.631556	3.729230	0.563247
H	0.253283	2.886287	-1.052979
H	1.534868	4.668198	-0.539605
C	6.952104	-0.640321	-1.099211
C	6.857251	-2.016463	0.882162
O	3.848613	5.621848	0.234663
C	7.988646	-1.424687	-1.590810
H	6.585698	0.197066	-1.680668
C	7.896118	-2.795874	0.386505
H	6.416213	-2.254074	1.843165
C	8.463404	-2.501734	-0.849081
H	8.425581	-1.191789	-2.555565
H	8.260361	-3.635233	0.968442

H 9.273338 -3.111791 -1.234014
O 5.075953 -0.135275 2.055081
H 5.932339 0.025477 2.472589

SCF Energy: -2178.86108012
Sum of electronic and zero-point Energies= -2178.425834
Sum of electronic and thermal Energies= -2178.389639
Sum of electronic and thermal Enthalpies= -2178.388695
Sum of electronic and thermal Free Energies= -2178.499353

==> ./AOX-II/PA/bis-/c4c5/RAF/c8 <==
O 5.821220 0.952919 -0.092632
O 3.832087 -2.032812 -0.417173
O 1.290119 2.456738 -0.444229
O 1.974507 -0.252795 -0.405246
O 4.848332 5.523733 0.191983
C 3.504648 1.606350 -0.234785
C 4.879994 1.933469 -0.091435
C 5.518821 -0.364970 -0.220072
C 3.136968 0.237111 -0.328578
C 4.217328 -0.737982 -0.332837
C 6.703821 -1.227082 -0.174036
C 2.609440 2.721945 -0.255893
C 5.352686 3.217931 0.051574
C 4.453008 4.320457 0.056825
C 3.060869 4.007090 -0.110061
C 7.859957 -0.782351 0.483901
C 6.720768 -2.487194 -0.787859
C 8.993724 -1.581365 0.536893
C 7.861329 -3.278342 -0.736649
C 8.999827 -2.832857 -0.072361
H 6.418010 3.387702 0.157954
H 2.347936 4.826422 -0.135746
H 7.866717 0.189490 0.962317
H 5.849748 -2.843874 -1.320548
H 9.875961 -1.224156 1.057042
H 7.859964 -4.247041 -1.224586
H 9.887309 -3.455194 -0.032600
H 2.859350 -1.998065 -0.447791
H 0.791829 3.282266 -0.429778
Cu 0.148951 0.334153 0.213729
O -1.739727 0.584575 0.876590
C -2.832349 0.053528 0.576257
C -3.106149 -1.346231 0.374412
C -3.961209 0.956158 0.425341
C -4.369412 -1.691759 -0.035936
C -2.167282 -2.441956 0.624141
O -3.704627 2.253710 0.640893
C -5.195378 0.509831 0.056997
O -5.341079 -0.823850 -0.190770
C -4.734314 -3.076299 -0.444544
O -0.962020 -2.068836 1.032972
C -2.514559 -3.760022 0.510957
H -2.754741 2.304084 0.849092
C -6.425398 1.268743 -0.158971
C -3.836431 -4.146814 0.155278
H -0.405254 -2.843854 1.189305
H -1.807867 -4.525358 0.812092
C -7.416545 0.757741 -1.010142
C -6.645323 2.496625 0.480245
O -4.296645 -5.285699 0.249208
C -8.590926 1.464677 -1.223525
H -7.262623 -0.190190 -1.511446
C -7.826523 3.194263 0.265056
H -5.905136 2.899804 1.157527
C -8.800439 2.685406 -0.588079
H -9.344695 1.060555 -1.890199
H -7.986769 4.139115 0.772531
H -9.719835 3.235789 -0.755520
H -4.478412 -3.112364 -1.521803
O -6.092659 -3.348367 -0.254939
H -6.152867 -4.306676 -0.131918

SCF Energy: -2178.89751670
Sum of electronic and zero-point Energies= -2178.461785
Sum of electronic and thermal Energies= -2178.425210
Sum of electronic and thermal Enthalpies= -2178.424266
Sum of electronic and thermal Free Energies= -2178.536859

==> ./AOX-II/PA/bis-/c4c5/RAF/c4a <==
O 5.631047 1.032291 -0.023088
O 3.789831 -2.052397 0.264508
O 1.150296 2.067128 -1.346795
O 1.899763 -0.459778 -0.466411
O 4.492230 5.424454 -1.190372
C 3.341458 1.464101 -0.635357
C 4.672787 1.909785 -0.421609
C 5.386458 -0.285851 0.196231
C 3.029407 0.101601 -0.383576
C 4.125106 -0.762814 0.025807
C 6.575350 -1.016651 0.645397
C 2.423589 2.464636 -1.083183
C 5.082611 3.212073 -0.596691
C 4.157199 4.207338 -1.019557
C 2.810167 3.766645 -1.257024
C 7.618841 -0.322283 1.275415
C 6.707663 -2.398664 0.451024
C 8.753611 -0.993653 1.708881

C 7.849374 -3.062630 0.881360
C 8.874383 -2.366630 1.514433
H 6.117442 3.477606 -0.413220
H 2.081632 4.495406 -1.601316
H 7.535938 0.746108 1.433281
H 5.927231 -2.954449 -0.050469
H 9.546704 -0.441196 2.201100
H 7.938605 -4.130715 0.714866
H 9.762698 -2.889886 1.851515
H 2.827896 -2.088604 0.115021
H 0.633325 2.826733 -1.639411
Cu 0.038524 0.288787 -0.076452
O -1.850508 0.502564 0.621721
C -2.934830 -0.072235 0.508542
C -3.114907 -1.601695 0.339039
C -4.156728 0.666018 0.678194
C -4.306270 -1.756412 -0.552657
C -1.901718 -2.319870 -0.205275
O -4.042686 1.964920 1.059133
C -5.376244 0.092481 0.440214
O -5.447177 -1.151105 -0.112674
C -4.303979 -2.402589 -1.717462
O -0.888337 -2.293117 0.655994
C -1.889698 -2.954065 -1.393807
H -3.096551 2.158570 1.128276
C -6.691881 0.688852 0.647443
C -3.076286 -3.037265 -2.218449
H -5.195781 -2.446834 -2.330222
H -0.124609 -2.773072 0.306263
H -0.995599 -3.467896 -1.729462
C -7.778529 0.255529 -0.128184
C -6.902014 1.672685 1.625166
O -3.079494 -3.619673 -3.306556
C -9.037262 0.806378 0.059189
H -7.630627 -0.504802 -0.884804
C -8.167277 2.209739 1.813604
H -6.083078 2.003919 2.248736
C -9.236001 1.784431 1.029820
H -9.866050 0.470763 -0.554105
H -8.319036 2.962172 2.579382
H -10.221842 2.212023 1.176542
O -3.461324 -2.127904 1.606866
H -2.710902 -2.023622 2.204971

SCF Energy: -2178.86235993
Sum of electronic and zero-point Energies= -2178.427782
Sum of electronic and thermal Energies= -2178.390917
Sum of electronic and thermal Enthalpies= -2178.389972
Sum of electronic and thermal Free Energies= -2178.502570

==> ./AOX-II/PA/bis-/c4c5/RAF/c3p <==
O 5.738239 -1.210228 -0.007682
O 4.248730 2.031240 0.487037
O 1.180551 -2.060707 1.182878
O 2.190225 0.524826 0.867155
O 4.140306 -5.608827 0.125094
C 3.415742 -1.530981 0.554859
C 4.691141 -2.050631 0.204256
C 5.638447 0.139729 0.097222
C 3.252284 -0.121762 0.628664
C 4.436843 0.693298 0.407768
C 6.898234 0.828230 -0.200555
C 2.399409 -2.510579 0.790933
C 4.960145 -3.392060 0.056560
C 3.936047 -4.358528 0.261829
C 2.648521 -3.850204 0.644160
C 7.842107 0.214971 -1.037415
C 7.195123 2.086295 0.341430
C 9.040339 0.849458 -1.334297
C 8.399467 2.712163 0.045634
C 9.324359 2.100982 -0.795173
H 5.959282 -3.710720 -0.217836
H 1.849406 -4.560743 0.835794
H 7.631486 -0.758272 -1.463938
H 6.494179 2.570155 1.008026
H 9.754707 0.363873 -1.990339
H 8.616748 3.681792 0.480501
H 10.261881 2.595107 -1.026431
H 3.298926 2.135989 0.673560
H 0.591505 -2.814357 1.304557
Cu 0.355572 0.314714 0.089060
O -1.435898 -0.037302 -0.759705
C -2.587375 0.429178 -0.538276
C -2.959619 1.770412 -0.277820
C -3.662636 -0.565098 -0.566894
C -4.326838 2.063341 -0.011891
C -2.073027 2.895879 -0.245966
O -3.272789 -1.824285 -0.828912
C -4.951403 -0.219408 -0.319745
O -5.257332 1.072070 -0.041307
C -4.797913 3.318417 0.286167
O -0.770218 2.661078 -0.536547
C -2.525103 4.153706 0.051256
H -2.304859 -1.768949 -0.939668
C -6.131710 -1.087227 -0.281901
C -3.905823 4.428312 0.340915
H -5.854032 3.462904 0.482157
H -0.272746 3.484033 -0.464416
H -1.823059 4.982437 0.063782
C -7.343505 -0.577898 0.147738
C -6.106918 -2.429748 -0.718062
O -4.299825 5.603490 0.623446

C -8.504824 -1.430811 0.289457
H -7.446969 0.449698 0.471437
C -7.232371 -3.256984 -0.696073
H -5.182265 -2.844147 -1.093465
C -8.439469 -2.771317 -0.269386
H -8.190151 -1.772309 1.340498
H -7.149818 -4.274063 -1.055700
H -9.336033 -3.380383 -0.250289
O -9.708865 -0.751346 0.330382
H -10.412815 -1.355636 0.591945

SCF Energy: -2178.83184216
Sum of electronic and zero-point Energies= -2178.399950
Sum of electronic and thermal Energies= -2178.363045
Sum of electronic and thermal Enthalpies= -2178.362100
Sum of electronic and thermal Free Energies= -2178.474777

==> ./AOX-II/PA/bis-/c4c5/RAF/c4p <==
O -5.635070 -1.229608 0.473022
O -4.311829 1.867005 -0.805477
O -1.380973 -2.330209 -1.420742
O -2.342297 0.284509 -1.324059
O -4.039801 -5.625598 0.711886
C -3.459807 -1.674791 -0.465865
C -4.636865 -2.110916 0.199754
C -5.575728 0.084449 0.136112
C -3.331783 -0.298223 -0.791544
C -4.463173 0.561432 -0.481510
C -6.765469 0.833331 0.552209
C -2.494122 -2.695483 -0.734836
C -4.854052 -3.408453 0.603393
C -3.876730 -4.413083 0.356142
C -2.690500 -3.991534 -0.335810
C -7.525348 0.380788 1.640752
C -7.176177 1.991805 -0.121341
C -8.654370 1.076310 2.051008
C -8.311181 2.678648 0.290372
C -9.052083 2.228454 1.378669
H -5.778691 -3.664585 1.107950
H -1.934593 -4.737222 -0.565058
H -7.224871 -0.513954 2.172461
H -6.620164 2.348166 -0.977919
H -9.224975 0.716719 2.900498
H -8.619721 3.568559 -0.247538
H -9.935664 2.769908 1.699064
H -3.417106 1.926089 -1.184579
H -0.808952 -3.099093 -1.526562
Cu -0.374674 0.054874 -1.048264
O 1.593351 -0.178130 -0.782056
C 2.630645 0.445865 -0.440357
C 2.834198 1.816807 -0.208504
C 3.807335 -0.438834 -0.269012
C 4.134853 2.270963 0.179403
C 1.828141 2.840766 -0.328299
O 3.571196 -1.702112 -0.502378
C 5.046026 0.054310 0.107966
O 5.160443 1.394415 0.320615
C 4.433380 3.581167 0.429070
O 0.593916 2.438325 -0.699384
C 2.112873 4.152027 -0.081603
H 2.617085 -1.748220 -0.739985
C 6.249878 -0.670487 0.302696
C 3.427451 4.591846 0.309536
H 5.440020 3.857882 0.718566
H 0.004885 3.201424 -0.741952
H 1.329648 4.896742 -0.183884
C 7.426412 0.049841 0.701308
C 6.327532 -2.094673 0.153643
O 3.671989 5.810281 0.532061
C 8.602302 -0.586655 0.884539
H 7.356959 1.112046 0.883796
C 7.501736 -2.738020 0.333669
H 5.441099 -2.664944 -0.070248
C 8.772031 -2.014827 0.560993
H 9.482317 -0.045310 1.212976
H 7.556589 -3.817763 0.246336
H 9.221628 -1.976892 -0.458750
O 9.668815 -2.638854 1.450118
H 9.977711 -3.458063 1.050310

SCF Energy: -2178.84518849
Sum of electronic and zero-point Energies= -2178.410651
Sum of electronic and thermal Energies= -2178.374120
Sum of electronic and thermal Enthalpies= -2178.373176
Sum of electronic and thermal Free Energies= -2178.483332

==> ./AOX-II/PA/bis-/c4c5/RAF/c7 <==
O 5.634960 -1.170964 0.327313
O 4.224484 2.051027 -0.448055
O 0.912619 -1.657353 0.941356
O 2.073933 0.724346 0.045081
O 3.852711 -5.328427 1.530559
C 3.241100 -1.322484 0.571754
C 4.532721 -1.913459 0.610726
C 5.577574 0.143613 -0.008014
C 3.127904 0.041376 0.188545
C 4.366850 0.756096 -0.081365
C 6.897286 0.713671 -0.296018

C	2.160619	-2.192713	0.920772
C	4.764348	-3.232512	0.927166
C	3.681191	-4.098465	1.245937
C	2.371241	-3.509136	1.236253
C	7.937560	-0.127682	-0.717534
C	7.156397	2.083439	-0.150102
C	9.194686	0.389508	-0.997880
C	8.418897	2.592900	-0.426248
C	9.441198	1.751937	-0.854595
H	5.780058	-3.611124	0.931490
H	1.521791	-4.133656	1.498347
H	7.756638	-1.189362	-0.833875
H	6.378630	2.750515	0.195589
H	9.984593	-0.275227	-1.330551
H	8.604267	3.654141	-0.300107
H	10.424498	2.154712	-1.072309
H	3.262342	2.199650	-0.458212
H	0.275795	-2.344540	1.168979
Cu	0.176249	0.296511	-0.474534
O	-1.679194	-0.019173	-1.238393
C	-2.823754	0.277586	-0.822357
C	-3.251414	1.528369	-0.246465
C	-3.826078	-0.765803	-0.923113
C	-4.555701	1.610209	0.175404
C	-2.385047	2.692892	-0.014259
O	-3.410188	-1.924555	-1.455193
C	-5.095774	-0.596392	-0.450241
O	-5.415407	0.606231	0.091920
C	-5.131232	2.809181	0.801326
O	-1.125360	2.572738	-0.488071
C	-2.815378	3.791834	0.636179
H	-2.465632	-1.795725	-1.656628
C	-6.195762	-1.559484	-0.406152
C	-4.214066	3.920878	1.052044
H	-5.994724	2.650451	1.439766
H	-0.627933	3.375652	-0.291092
H	-2.137542	4.614708	0.837466
C	-7.208578	-1.409749	0.552672
C	-6.269032	-2.628470	-1.309636
O	-5.178604	3.987264	-0.002049
C	-8.259887	-2.312965	0.611043
H	-7.167124	-0.589699	1.259220
C	-7.328444	-3.524325	-1.247802
H	-5.511151	-2.752877	-2.070665
C	-8.323991	-3.373955	-0.287945
H	-9.031110	-2.188131	1.363137
H	-7.376071	-4.342325	-1.958177
H	-9.147356	-4.078388	-0.241347
O	-4.484390	4.725579	2.133223
H	-3.998293	5.553336	2.038349

SCF Energy: -2178.86212543

Sum of electronic and zero-point Energies= -2178.426881

Sum of electronic and thermal Energies= -2178.390598

Sum of electronic and thermal Enthalpies= -2178.389654

Sum of electronic and thermal Free Energies= -2178.500738

==> ./AOX-II/PA/bis-/c4c5/RAF/c8a <==

O	4.616493	0.616557	-1.230193
O	4.538227	-0.979346	1.996006
O	0.117680	-0.936181	-0.693548
O	2.075352	-1.242079	1.307795
O	1.427973	1.487602	-4.543983
C	2.370747	-0.172364	-0.840714
C	3.329889	0.496449	-1.650108
C	5.058460	0.126879	-0.044217
C	2.780289	-0.668922	0.426561
C	4.181346	-0.502945	0.780639
C	6.478108	0.405514	0.193252
C	1.057975	-0.262437	-1.404405
C	3.047834	1.053141	-2.876558
C	1.733536	0.980635	-3.416638
C	0.757080	0.290328	-2.620305
C	7.081633	1.506418	-0.432045
C	7.259372	-0.410425	1.023023
C	8.423837	1.789782	-0.220845
C	8.603855	-0.124749	1.223836
C	9.190862	0.976273	0.608296
H	3.835702	1.548758	-3.432038
H	-0.252944	0.194832	-3.008906
H	6.493741	2.146708	-1.078531
H	6.823316	-1.278009	1.499166
H	8.871899	2.650276	-0.705830
H	9.196145	-0.771263	1.862324
H	10.240219	1.197539	0.770795
H	3.714440	-1.331104	2.376929
H	-0.724200	-0.916114	-1.164872
Cu	0.109977	-1.248179	1.696311
O	-1.791035	-1.262789	2.276593
C	-2.693312	-0.868737	1.487745
C	-3.281259	-1.650831	0.444329
C	-3.144271	0.502377	1.602480
C	-4.546622	-1.095851	-0.141079
C	-2.787091	-2.829640	-0.077527
O	-2.602370	1.217198	2.612674
C	-3.951609	1.069849	0.651369
O	-4.403646	0.300566	-0.382829
C	-5.025981	-1.712030	-1.409922
O	-1.734413	-3.425616	0.488160
C	-3.364978	-3.394556	-1.242156
H	-2.003149	0.610586	3.078769

C	-4.357072	2.466979	0.547586
C	-4.456850	-2.854797	-1.995417
H	-5.932227	-1.280068	-1.824858
H	-1.440075	-4.177006	-0.042917
H	-2.931244	-4.322351	-1.607721
C	-4.779734	2.967211	-0.696694
C	-4.350631	3.331827	1.654710
O	-4.875420	-3.388073	-3.065358
C	-5.162378	4.292501	-0.830827
H	-4.795738	2.316223	-1.561361
C	-4.747723	4.653704	1.513166
H	-4.057527	2.969098	2.629218
C	-5.148508	5.141527	0.272838
H	-5.474575	4.664168	-1.800456
H	-4.747517	5.305321	2.379906
H	-5.452691	6.177147	0.167177
O	-5.554141	-1.317966	0.816283
H	-6.411336	-1.085199	0.435159

SCF Energy: -2178.80233297
Sum of electronic and zero-point Energies= -2178.368578
Sum of electronic and thermal Energies= -2178.332029
Sum of electronic and thermal Enthalpies= -2178.331085
Sum of electronic and thermal Free Energies= -2178.441765

==> ./AOX-II/PA/bis-/c4c5/RAF/c2p <==

O	-5.839034	1.062230	-0.026672
O	-4.094634	-2.070216	0.320693
O	-1.290516	2.222747	0.906500
O	-2.131903	-0.425918	0.606086
O	-4.546066	5.562410	0.070249
C	-3.515718	1.541517	0.402106
C	-4.842886	1.972737	0.133811
C	-5.637443	-0.278545	0.049640
C	-3.247776	0.146612	0.443922
C	-4.382839	-0.748587	0.276869
C	-6.861866	-1.051458	-0.180904
C	-2.559525	2.588493	0.590872
C	-5.213494	3.293553	0.020982
C	-4.248989	4.328071	0.177904
C	-2.908194	3.908215	0.476597
C	-7.902658	-0.492311	-0.936892
C	-7.029414	-2.338964	0.347256
C	-9.069625	-1.207182	-1.169006
C	-8.202750	-3.045783	0.116279
C	-9.224838	-2.487249	-0.644757
H	-6.247282	3.543389	-0.188525
H	-2.150127	4.671456	0.628750
H	-7.792419	0.502708	-1.350765
H	-6.251717	-2.783778	0.953090
H	-9.860512	-0.761776	-1.762780
H	-8.318806	-4.037895	0.539045
H	-10.137869	-3.044182	-0.825683
H	-3.130485	-2.108190	0.450474
H	-0.747236	3.013926	0.998721
Cu	-0.247325	0.040819	0.076573
O	1.629297	0.340972	-0.572556
C	2.744151	-0.202176	-0.353539
C	3.059469	-1.555867	-0.138460
C	3.871760	0.752592	-0.346354
C	4.415283	-1.923719	0.122361
C	2.111529	-2.638633	-0.140256
O	3.537750	1.996999	-0.580896
C	5.174229	0.340879	-0.132846
O	5.394276	-0.979737	0.125012
C	4.816722	-3.203073	0.384784
O	0.830478	-2.320786	-0.425401
C	2.495142	-3.922401	0.123666
H	2.562535	1.981892	-0.707168
C	6.349102	1.133503	-0.146267
C	3.863085	-4.271603	0.404956
H	5.862446	-3.411230	0.578501
H	0.286855	-3.116595	-0.382636
H	1.752729	-4.714811	0.110813
C	7.679676	0.435739	0.014511
C	6.332266	2.531275	-0.200054
O	4.199784	-5.464407	0.647203
C	8.876458	1.303017	-0.036775
H	7.659996	0.113656	1.079911
C	7.507262	3.253445	-0.141248
H	5.400302	3.066324	-0.284848
C	8.789705	2.648974	-0.050618
H	9.830521	0.789098	-0.021460
H	7.444456	4.335189	-0.179202
H	9.676574	3.269054	-0.035984
O	7.891752	-0.674181	-0.825445
H	7.256069	-1.359328	-0.590304

SCF Energy: -2178.84187190
Sum of electronic and zero-point Energies= -2178.407738
Sum of electronic and thermal Energies= -2178.370999
Sum of electronic and thermal Enthalpies= -2178.370055
Sum of electronic and thermal Free Energies= -2178.482628

==> ./AOX-II/PA/bis-/c4c5/RAF/c3 <==

O	-5.775673	-1.057605	-0.242394
O	-4.100684	2.043802	0.496554
O	-1.228855	-2.243834	0.674431

O	-2.118034	0.411884	0.664662
O	-4.429010	-5.534402	-0.499984
C	-3.455135	-1.545156	0.197443
C	-4.771191	-1.966758	-0.135279
C	-5.596359	0.273106	-0.039847
C	-3.211225	-0.157222	0.383424
C	-4.355200	0.735321	0.263058
C	-6.842301	1.037976	-0.153162
C	-2.488775	-2.594464	0.310254
C	-5.122010	-3.276441	-0.372007
C	-4.148595	-4.310623	-0.283404
C	-2.819011	-3.903408	0.075832
C	-8.068490	0.401754	0.090341
C	-6.846255	2.393899	-0.508739
C	-9.260717	1.106057	-0.004238
C	-8.043904	3.090468	-0.608571
C	-9.254305	2.453623	-0.353051
H	-6.147963	-3.517932	-0.625278
H	-2.053771	-4.668393	0.174730
H	-8.085497	-0.646227	0.363959
H	-5.916364	2.902610	-0.723167
H	-10.198582	0.599521	0.196702
H	-8.029026	4.137179	-0.892532
H	-10.186679	3.002766	-0.427901
H	-3.149892	2.076924	0.702822
H	-0.673178	-3.031539	0.691231
Cu	-0.175176	0.077982	0.270793
O	1.771927	-0.299878	-0.104670
C	2.887421	0.249941	-0.042338
C	3.205263	1.619767	-0.057190
C	4.032547	-0.775260	0.100354
C	4.564012	2.025138	-0.045130
C	2.264948	2.705036	-0.081399
O	3.874225	-1.758650	-0.870789
C	5.433046	-0.200392	-0.060570
O	5.585987	1.069612	-0.083910
C	5.009400	3.303393	-0.026916
O	0.961683	2.375890	-0.134407
C	2.673229	4.015835	-0.047743
H	2.921997	-1.882278	-0.998406
C	6.620098	-0.987000	-0.155463
C	4.053264	4.389680	-0.012419
H	6.070766	3.516864	-0.032733
H	0.420004	3.174693	-0.130321
H	1.925598	4.803013	-0.059140
C	7.849502	-0.344023	-0.429168
C	6.594562	-2.386053	0.035012
O	4.433398	5.588375	0.016717
C	9.010393	-1.082195	-0.515313
H	7.874879	0.726680	-0.583143
C	7.768500	-3.110446	-0.043529
H	5.668193	-2.894850	0.255700
C	8.971258	-2.464163	-0.320861
H	9.949697	-0.589270	-0.734073
H	7.749422	-4.182094	0.111663
H	9.887050	-3.040749	-0.386422
O	4.027041	-1.282111	1.409178
H	3.113596	-1.437401	1.683924

SCF Energy: -2178.86433248

Sum of electronic and zero-point Energies= -2178.429514

Sum of electronic and thermal Energies= -2178.392769

Sum of electronic and thermal Enthalpies= -2178.391825

Sum of electronic and thermal Free Energies= -2178.503330

==> ./AOX-II/PA/bis-/c4c5/RAF/c4 <==

O	5.260648	1.226283	-0.027301
O	4.162323	-2.189010	-0.351055
O	0.491339	1.426026	-0.331972
O	1.884213	-0.979325	-0.331299
O	3.075291	5.357318	0.261344
C	2.853830	1.222233	-0.156574
C	4.087844	1.913109	-0.023233
C	5.328906	-0.124637	-0.148819
C	2.873396	-0.194584	-0.254580
C	4.178132	-0.838369	-0.264936
C	6.703208	-0.632208	-0.089812
C	1.685517	2.048165	-0.165660
C	4.190980	3.278326	0.114064
C	3.022955	4.091509	0.127228
C	1.768130	3.408607	-0.025867
C	7.691905	0.117167	0.565141
C	7.064301	-1.850255	-0.682472
C	8.998217	-0.346458	0.636511
C	8.375177	-2.304535	-0.612684
C	9.346084	-1.559499	0.049147
H	5.169381	3.734280	0.213493
H	0.857181	4.000210	-0.040299
H	7.434045	1.061874	1.028092
H	6.326228	-2.437026	-1.212052
H	9.747231	0.242899	1.154345
H	8.638208	-3.245570	-1.083648
H	10.367669	-1.919713	0.104465
H	3.218158	-2.425113	-0.370671
H	-0.222006	2.074951	-0.303334
Cu	0.066237	-0.919488	0.540121
O	-1.674588	-1.300612	1.599569
C	-2.860647	-1.486289	1.435509
C	-3.550873	-1.433570	0.043991
C	-3.545914	0.028419	-0.352644
C	-5.027079	-1.658244	0.145618
C	-2.896075	-2.498365	-0.795556

O	-2.455825	0.720414	-0.711542
C	-4.771561	0.537050	-0.108629
O	-5.659440	-0.478611	0.288397
C	-5.618904	-2.851267	0.032042
O	-1.717881	-2.129208	-1.294294
C	-3.465832	-3.714768	-0.904787
H	-1.743154	0.121222	-0.971738
C	-5.331475	1.874066	-0.194695
C	-4.812125	-3.987520	-0.407779
H	-6.689725	-2.974516	0.134583
H	-1.282788	-2.862496	-1.751305
H	-2.950768	-4.513894	-1.426586
C	-6.692918	2.087401	0.067104
C	-4.530312	2.972623	-0.545242
O	-5.289739	-5.128484	-0.461034
C	-7.235798	3.362611	-0.022712
H	-7.327952	1.253691	0.339860
C	-5.083363	4.242452	-0.634553
H	-3.476823	2.835303	-0.750048
C	-6.436277	4.445321	-0.374990
H	-8.290807	3.508631	0.182824
H	-4.450868	5.079971	-0.908546
H	-6.862957	5.439865	-0.446707
O	-3.702774	-1.712625	2.418880
H	-3.226231	-1.697698	3.265448

SCF Energy: -2178.88591806
Sum of electronic and zero-point Energies= -2178.450549
Sum of electronic and thermal Energies= -2178.413846
Sum of electronic and thermal Enthalpies= -2178.412902
Sum of electronic and thermal Free Energies= -2178.524304

==> ./AOX-II/PA/bis-/c4c5/RAF/c5p <==

O	-5.813375	-0.992123	0.165476
O	-3.865217	1.981393	0.736273
O	-1.517313	-1.899188	-1.747337
O	-2.109040	0.513044	-0.434349
O	-4.960205	-5.153843	-1.797597
C	-3.607880	-1.358047	-0.741113
C	-4.927247	-1.808239	-0.463148
C	-5.507450	0.270541	0.559844
C	-3.231632	-0.053257	-0.319079
C	-4.255585	0.748484	0.337216
C	-6.624002	0.942292	1.232383
C	-2.775956	-2.296629	-1.430314
C	-5.400350	-3.056565	-0.798097
C	-4.561547	-3.989100	-1.469377
C	-3.229522	-3.544256	-1.769647
C	-7.613286	0.176243	1.866359
C	-6.741652	2.339083	1.247078
C	-8.680088	0.790930	2.507227
C	-7.815797	2.946982	1.884422
C	-8.786320	2.178734	2.519898
H	-6.421011	-3.325467	-0.551135
H	-2.565451	-4.225061	-2.294915
H	-7.540999	-0.904591	1.863473
H	-6.003855	2.951861	0.747243
H	-9.431315	0.182332	2.998942
H	-7.895547	4.028695	1.878807
H	-9.621518	2.657993	3.019144
H	-2.927255	2.038756	0.482056
H	-1.060836	-2.618618	-2.198909
Cu	-0.181225	0.087159	-0.773744
O	1.777378	-0.378037	-0.918641
C	2.836007	0.240840	-0.622764
C	3.121687	1.620536	-0.773364
C	3.901518	-0.597401	-0.073083
C	4.386897	2.113481	-0.346684
C	2.233940	2.602346	-1.320963
O	3.618571	-1.907916	0.028225
C	5.097194	-0.070284	0.289760
O	5.319135	1.260050	0.158234
C	4.754155	3.435758	-0.406050
O	1.039681	2.161848	-1.787144
C	2.581086	3.925034	-1.383847
H	2.710696	-1.997911	-0.315940
C	6.268652	-0.787929	0.798761
C	3.853415	4.410599	-0.924889
H	5.735652	3.736542	-0.058318
H	0.528485	2.912185	-2.112645
H	1.880076	4.638205	-1.807577
C	7.543746	-0.184715	0.742728
C	6.173302	-2.035852	1.382941
O	4.151595	5.644809	-0.992794
C	8.704857	-0.808620	1.204352
H	7.628941	0.807494	0.317747
C	7.365950	-2.766367	1.765043
H	5.227452	-2.544344	1.509147
C	8.635407	-2.054018	1.769620
H	9.651894	-0.289612	1.138238
H	9.511737	-2.572903	2.141037
H	7.513668	-3.187782	0.707909
O	7.137786	-3.784232	2.674114
H	7.905753	-4.365442	2.707323

SCF Energy: -2178.83204519
Sum of electronic and zero-point Energies= -2178.400057
Sum of electronic and thermal Energies= -2178.363085
Sum of electronic and thermal Enthalpies= -2178.362141

Sum of electronic and thermal Free Energies= -2178.474896

==> ./AOX-II/PA <==

O	-0.199130	-0.882597	0.078446
O	-1.270594	2.563037	-0.147414
O	3.798202	1.764772	-0.100298
O	1.352100	2.850034	-0.165672
O	4.023230	-2.944396	0.178034
C	1.749736	0.510722	-0.014629
C	1.157215	-0.769365	0.061871
C	-1.030345	0.194985	0.020735
C	0.929786	1.664615	-0.079787
C	-0.506639	1.447853	-0.057093
C	-2.446831	-0.186480	0.016292
C	3.175739	0.561119	-0.026007
C	1.888500	-1.932778	0.125896
C	3.315244	-1.884261	0.117098
C	3.924797	-0.585253	0.037267
C	-2.825272	-1.441831	-0.481490
C	-3.440519	0.669162	0.510995
C	-4.159976	-1.822797	-0.496127
C	-4.773634	0.278486	0.499727
C	-5.140387	-0.964543	-0.006532
H	1.381639	-2.889254	0.185068
H	5.007713	-0.515842	0.027429
H	-2.071183	-2.117574	-0.866514
H	-3.172787	1.633471	0.921287
H	-4.434686	-2.794252	-0.892813
H	-5.528618	0.949641	0.894777
H	-6.182757	-1.264306	-0.016451
H	-0.639059	3.298860	-0.223303
H	3.104798	2.454650	-0.143540

SCF Energy: -952.937052779
Sum of electronic and zero-point Energies= -952.729110
Sum of electronic and thermal Energies= -952.713733
Sum of electronic and thermal Enthalpies= -952.712788
Sum of electronic and thermal Free Energies= -952.772307

==> ./AOX-II/PA/PA/bis-/c4c5 <==

O	5.397713	-1.124364	-0.035103
O	3.959728	2.136566	0.527400
O	0.737392	-1.878339	0.641628
O	1.788324	0.634099	0.442682
O	3.693701	-5.482999	-0.203929
C	3.044594	-1.390442	0.254657
C	4.336337	-1.937273	0.040938
C	5.330800	0.240057	0.127340
C	2.900004	0.017136	0.347449
C	4.110354	0.877928	0.348138
C	6.651040	0.854269	0.012921
C	1.990811	-2.351763	0.344939
C	4.578726	-3.290398	-0.114270
C	3.517723	-4.228289	-0.056361
C	2.203349	-3.690480	0.191379
C	7.792364	0.049609	-0.184390
C	6.847349	2.247663	0.083393
C	9.056537	0.608441	-0.302903
C	8.117556	2.798081	-0.040109
C	9.232189	1.988383	-0.232535
H	5.593640	-3.631855	-0.285075
H	1.368061	-4.379392	0.275069
H	7.687821	-1.025981	-0.244789
H	5.994831	2.892741	0.232594
H	9.912543	-0.041887	-0.452274
H	8.233086	3.875935	0.016077
H	10.221490	2.423553	-0.326460
H	0.084910	-2.589919	0.611560
Cu	-0.000046	0.001009	-0.002248
O	-1.788179	-0.632286	-0.447876
C	-2.900106	-0.016030	-0.350825
C	-3.045256	1.391358	-0.256179
C	-4.110005	-0.877423	-0.351278
C	-4.337047	1.937314	-0.040537
C	-1.992015	2.353272	-0.346567
O	-3.958956	-2.135752	-0.532304
C	-5.330461	-0.240476	-0.127792
O	-5.397879	1.123737	0.036130
C	-4.580007	3.290185	0.115961
O	-0.738609	1.880717	-0.644642
C	-2.205139	3.691758	-0.191761
C	-6.650036	-0.855657	-0.011038
C	-3.519565	4.228687	0.057599
H	-5.594912	3.630962	0.288166
H	-0.086545	2.592703	-0.615121
H	-1.370269	4.381157	-0.275588
C	-7.791012	-0.052208	0.193145
C	-6.846004	-2.248887	-0.085776
O	-3.696056	5.483198	0.206262
C	-9.054502	-0.612023	0.314238
H	-7.686710	1.023204	0.257058
C	-8.115541	-2.800289	0.040200
H	-5.993736	-2.893053	-0.240236
C	-9.229818	-1.991787	0.239550
H	-9.910240	0.037372	0.469112
H	-8.230812	-3.877981	-0.019515
H	-10.218588	-2.427728	0.335460

SCF Energy: -2102.13749840
Sum of electronic and zero-point Energies= -2101.744145

Sum of electronic and thermal Energies= -2101.710231
Sum of electronic and thermal Enthalpies= -2101.709287
Sum of electronic and thermal Free Energies= -2101.815524

==> ./AOX-II/PA/PA/bis-/c4c5/RAF/c5 <==

O	-5.215581	0.946482	-0.081216
O	-4.438086	-2.500590	0.658925
O	-0.471224	0.809817	0.375782
O	-2.012530	-1.495678	0.321144
O	-2.681069	4.788817	-0.936261
C	-2.834737	0.741004	0.079072
C	-4.000657	1.512630	-0.159332
C	-5.416700	-0.382907	0.234510
C	-2.968411	-0.669825	0.264827
C	-4.339620	-1.245998	0.406618
C	-6.839432	-0.701102	0.306378
C	-1.597577	1.469000	0.025226
C	-3.975339	2.855379	-0.498431
C	-2.744636	3.549605	-0.616539
C	-1.557451	2.795765	-0.326756
C	-7.804723	0.276546	-0.014133
C	-7.313858	-1.969448	0.698078
C	-9.162786	-0.001252	0.048864
C	-8.676208	-2.237069	0.760403
C	-9.613503	-1.261065	0.435965
H	-4.910201	3.376532	-0.675254
H	-0.600003	3.308819	-0.345974
H	-7.485472	1.265257	-0.318075
H	-6.601640	-2.739823	0.953989
H	-9.875682	0.776165	-0.207717
H	-9.006077	-3.224545	1.068275
H	-10.675734	-1.476405	0.484478
H	0.345276	1.198581	-0.038165
Cu	-0.163549	-1.371890	-0.463270
O	1.705776	-1.224131	-1.189356
C	2.738354	-0.710960	-0.712516
C	3.906342	-1.507523	-0.396245
C	2.811377	0.759879	-0.506609
C	5.080807	-0.872710	0.137894
C	3.951680	-2.885833	-0.501418
O	1.784168	1.470389	-0.816251
C	4.002832	1.274278	-0.018668
O	5.076632	0.444181	0.279054
C	6.211865	-1.528116	0.585959
O	2.911429	-3.616149	-0.792502
C	5.243365	-3.620234	-0.338092
C	4.342471	2.671678	0.236040
C	6.296820	-2.935808	0.530403
H	6.990228	-0.960377	1.080901
H	3.177707	-4.554140	-0.722510
H	5.685659	-3.554996	-1.348859
C	5.648978	3.025153	0.628872
C	3.398497	3.709093	0.107496
O	7.183611	-3.631169	1.045024
C	5.992145	4.346840	0.874041
H	6.404203	2.258268	0.741316
C	3.751795	5.029186	0.357853
H	2.388553	3.469545	-0.189506
C	5.047353	5.360950	0.740565
H	7.007982	4.585274	1.172106
H	3.001297	5.805810	0.251198
H	5.317928	6.393725	0.932776
O	4.971615	-4.953939	-0.008672
H	5.586715	-5.529966	-0.471477

SCF Energy: -2177.95836289
Sum of electronic and zero-point Energies= -2177.549340
Sum of electronic and thermal Energies= -2177.513730
Sum of electronic and thermal Enthalpies= -2177.512786
Sum of electronic and thermal Free Energies= -2177.622749

==> ./AOX-II/PA/PA/bis-/c4c5/RAF/c1p <==

O	5.240239	-1.053534	-0.727072
O	4.611750	1.146379	2.065198
O	0.686456	-1.847000	0.481193
O	2.210626	0.112649	1.689614
O	2.706674	-4.178105	-3.115169
C	2.956712	-1.332953	-0.063363
C	4.056381	-1.660916	-0.898386
C	5.486267	-0.124114	0.263404
C	3.122214	-0.322477	0.933187
C	4.475249	0.280297	1.128243
C	6.869240	0.341045	0.220302
C	1.758393	-2.073729	-0.326729
C	3.996833	-2.593705	-1.920163
C	2.799604	-3.303270	-2.185165
C	1.682479	-2.998921	-1.335616
C	7.729496	-0.079040	-0.815832
C	7.406922	1.209927	1.190907
C	9.047755	0.349558	-0.878199
C	8.729411	1.631514	1.121235
C	9.562014	1.209643	0.089215
H	4.883087	-2.785204	-2.515552
H	0.752925	-3.540592	-1.489208
H	7.359084	-0.747019	-1.582914
H	6.776776	1.546723	2.000295
H	9.679028	0.007404	-1.692335
H	9.111350	2.299001	1.887521
H	10.593076	1.543390	0.039101

H	-0.049731	-2.396560	0.189322
Cu	0.235853	0.362688	1.593889
O	-1.726174	0.614230	1.870075
C	-2.645680	0.742478	1.029907
C	-2.633012	1.520354	-0.147448
C	-3.921251	-0.024092	1.355836
C	-3.727555	1.442923	-1.059634
C	-1.552189	2.382461	-0.554243
O	-3.978284	-0.625797	2.420740
C	-5.010414	0.001560	0.381755
O	-4.820954	0.690475	-0.782588
C	-3.752637	2.090212	-2.264544
O	-0.532152	2.533039	0.319976
C	-1.561685	3.035544	-1.755796
C	-6.227334	-0.631704	0.489683
C	-2.656485	2.914799	-2.677027
H	-4.609769	1.975338	-2.917703
H	0.130068	3.122812	-0.060336
H	-0.729094	3.679677	-2.021998
C	-7.216636	-0.451042	-0.653629
C	-6.617754	-1.438206	1.609556
O	-2.656641	3.514933	-3.791295
C	-8.384003	-1.383173	-0.620808
H	-6.680606	-0.601105	-1.594842
C	-7.757028	-2.174769	1.572779
H	-5.988102	-1.465723	2.484524
C	-8.634531	-2.183226	0.425382
H	-9.048142	-1.342846	-1.477921
H	-8.021922	-2.780491	2.432684
H	-9.504924	-2.830036	0.434495
O	-7.684356	0.901810	-0.720666
H	-8.234968	1.062749	0.053182

SCF Energy: -2177.93382418
Sum of electronic and zero-point Energies= -2177.525208
Sum of electronic and thermal Energies= -2177.488861
Sum of electronic and thermal Enthalpies= -2177.487917
Sum of electronic and thermal Free Energies= -2177.600170

==> ./AOX-II/PA/PA/bis-/c4c5/RAF/c6p <==

O	-5.326375	-1.088873	-0.730695
O	-4.633640	1.589470	1.589689
O	-0.755464	0.073036	-1.485432
O	-2.227940	1.157076	0.579145
O	-2.836688	-3.400396	-3.948254
C	-3.028695	-0.466934	-0.979664
C	-4.146668	-1.259699	-1.346376
C	-5.551422	-0.131543	0.238354
C	-3.166973	0.469807	0.089122
C	-4.518478	0.684571	0.687261
C	-6.939584	-0.151464	0.689135
C	-1.838030	-0.713557	-1.738141
C	-4.107386	-2.240568	-2.322994
C	-2.913845	-2.500932	-3.040771
C	-1.781883	-1.682463	-2.706316
C	-7.837172	-1.117903	0.188488
C	-7.445545	0.773628	1.624284
C	-9.160930	-1.157083	0.602625
C	-8.773320	0.726698	2.032130
C	-9.643355	-0.235844	1.529284
H	-5.004974	-2.809603	-2.540077
H	-0.857664	-1.826532	-3.259553
H	-7.491892	-1.848229	-0.532009
H	-6.785701	1.529064	2.023620
H	-9.821776	-1.916461	0.196354
H	-9.129870	1.455943	2.753002
H	-10.678637	-0.267973	1.851995
H	-0.028736	-0.199582	-2.057150
Cu	-0.276523	0.861994	0.841389
O	1.597776	0.534212	1.442011
C	2.720946	0.703069	0.918142
C	3.143263	1.759510	0.080032
C	3.764386	-0.333660	1.291662
C	4.433082	1.709668	-0.531335
C	2.364793	2.933097	-0.223751
O	3.503976	-1.125124	2.198653
C	4.998591	-0.367666	0.534198
O	5.260414	0.651205	-0.340053
C	4.910034	2.682380	-1.367628
O	1.157647	3.036227	0.374739
C	2.828931	3.914105	-1.055704
C	5.900337	-1.412114	0.522039
C	4.122592	3.837886	-1.674026
H	5.894153	2.574384	-1.808541
H	0.726633	3.848494	0.083340
H	2.212832	4.787667	-1.246290
C	6.993090	-1.401845	-0.405309
C	5.742825	-2.605968	1.448954
O	4.544772	4.749943	-2.443934
C	7.810327	-2.475924	-0.544852
H	7.160331	-0.519029	-1.007419
C	6.686054	-3.730275	1.179974
C	7.636540	-3.675883	0.236856
H	8.624109	-2.434563	-1.260747
H	6.559671	-4.605144	1.808718
H	8.299237	-4.516663	0.066808
H	4.727112	-3.002702	1.292841
O	5.883318	-2.256557	2.820229
H	5.056944	-1.807242	3.052006

SCF Energy: -2177.93770699

Sum of electronic and zero-point Energies= -2177.528998
Sum of electronic and thermal Energies= -2177.492942
Sum of electronic and thermal Enthalpies= -2177.491998
Sum of electronic and thermal Free Energies= -2177.603169

==> ./AOX-II/PA/PA/bis-/c4c5/RAF/c8 <==

O	5.694760	1.035532	0.373129
O	3.996026	-2.057893	-0.378295
O	1.131698	2.278151	-0.327117
O	2.007298	-0.339452	-0.572072
O	4.410444	5.518821	0.749169
C	3.383626	1.536691	-0.009851
C	4.707187	1.941096	0.307041
C	5.511483	-0.314935	0.154302
C	3.125144	0.153715	-0.263686
C	4.255684	-0.820183	-0.162973
C	6.763722	-1.050140	0.305184
C	2.422591	2.600175	-0.042977
C	5.074115	3.252396	0.561477
C	4.115494	4.294532	0.519605
C	2.770549	3.902847	0.204643
C	7.947127	-0.372134	0.666384
C	6.857052	-2.441856	0.101026
C	9.150028	-1.047470	0.815777
C	8.066902	-3.108943	0.252214
C	9.223271	-2.423176	0.610626
H	6.109261	3.476687	0.795462
H	2.003030	4.671713	0.167680
H	7.922431	0.697132	0.832939
H	5.971279	-2.992489	-0.178542
H	10.039299	-0.491418	1.095851
H	8.101958	-4.181203	0.085930
H	10.164668	-2.949479	0.728165
H	0.595559	3.078885	-0.312919
Cu	0.106794	0.085502	-0.959266
O	-1.817791	0.424675	-1.364123
C	-2.902078	-0.019510	-0.921070
C	-3.164458	-1.398541	-0.595695
C	-4.000555	0.971516	-0.716067
C	-4.373497	-1.707053	-0.012023
C	-2.230406	-2.510270	-0.771455
O	-3.786712	2.174493	-1.062200
C	-5.179571	0.510078	-0.123041
O	-5.297190	-0.820790	0.211997
C	-4.774662	-3.101460	0.338833
O	-1.153959	-2.245847	-1.503499
C	-2.420909	-3.745490	-0.207050
C	-6.373238	1.264532	0.243421
C	-3.595586	-4.040123	0.534002
H	-0.590673	-3.029233	-1.569321
H	-1.638518	-4.492993	-0.281317
C	-7.446323	0.627599	0.897938
C	-6.502815	2.637353	-0.038341
O	-3.765183	-5.028681	1.254265
C	-8.588497	1.330760	1.251523
H	-7.385821	-0.427685	1.133102
C	-7.651600	3.332460	0.320566
H	-5.696310	3.150855	-0.540174
C	-8.702080	2.689473	0.966548
H	-9.397265	0.811228	1.755161
H	-7.722464	4.390548	0.089589
H	-9.596473	3.236931	1.244277
H	-5.259052	-3.497519	-0.574386
O	-5.669684	-3.139250	1.416066
H	-5.521796	-3.993232	1.847357

SCF Energy: -2177.96437641
Sum of electronic and zero-point Energies= -2177.554297
Sum of electronic and thermal Energies= -2177.518297
Sum of electronic and thermal Enthalpies= -2177.517353
Sum of electronic and thermal Free Energies= -2177.628816

==> ./AOX-II/PA/PA/bis-/c4c5/RAF/c2 <==

O	-5.461188	-1.194227	-0.307159
O	-4.493520	2.067239	0.904682
O	-0.866762	-0.717085	-1.513878
O	-2.158543	1.108224	0.086163
O	-3.262558	-4.675493	-2.536397
C	-3.141237	-0.878909	-0.799184
C	-4.322844	-1.662402	-0.841435
C	-5.580479	0.061002	0.256000
C	-3.167113	0.377750	-0.121751
C	-4.475169	0.896963	0.377997
C	-6.950115	0.318183	0.690771
C	-2.004021	-1.463225	-1.445541
C	-4.388934	-2.925438	-1.406100
C	-3.246191	-3.510534	-2.006212
C	-2.051787	-2.712294	-2.007636
C	-7.931375	-0.690076	0.587645
C	-7.356890	1.560756	1.217458
C	-9.240015	-0.468242	0.992278
C	-8.670479	1.773540	1.618423
C	-9.623802	0.765619	1.512394
H	-5.329597	-3.465329	-1.391422
H	-1.164552	-3.109808	-2.493235
H	-7.664032	-1.659487	0.186911
H	-6.632504	2.356750	1.304284
H	-9.967063	-1.268993	0.899444

H	-8.949433	2.743855	2.017505
H	-10.647604	0.937212	1.827723
H	-0.184982	-1.225151	-1.967763
Cu	-0.220006	0.737308	0.417836
O	1.615298	0.469683	1.172717
C	2.774530	0.651912	0.742151
C	3.245146	1.638697	-0.150062
C	3.813864	-0.322103	1.288646
C	4.578941	1.561195	-0.671374
C	2.455510	2.741431	-0.636042
O	3.494010	-1.202462	2.051186
C	5.290179	-0.072074	0.939184
O	5.411479	0.529161	-0.326300
C	5.069500	2.436182	-1.592247
O	1.217987	2.875763	-0.113781
C	2.935597	3.626503	-1.562387
C	6.097196	-1.354145	0.885955
C	4.262242	3.515218	-2.095260
H	6.080799	2.315370	-1.962514
H	0.781315	3.636588	-0.515292
H	2.309501	4.450025	-1.891748
C	5.790698	-2.301640	-0.092907
C	7.122855	-1.610789	1.789957
O	4.708016	4.328969	-2.951577
C	6.507958	-3.487892	-0.169708
H	4.989975	-2.110909	-0.800293
C	7.841300	-2.801694	1.712263
H	7.374756	-0.885501	2.554722
C	7.536884	-3.740833	0.734432
H	6.263893	-4.215603	-0.935997
H	8.641678	-2.990786	2.419451
H	8.098009	-4.667258	0.675244
O	5.759942	0.872245	1.871715
H	5.635016	0.545007	2.770525

SCF Energy: -2177.97586584

Sum of electronic and zero-point Energies= -2177.566194

Sum of electronic and thermal Energies= -2177.530150

Sum of electronic and thermal Enthalpies= -2177.529206

Sum of electronic and thermal Free Energies= -2177.639942

==> ./AOX-II/PA/PA/bis-/c4c5/RAF/c4a <==

O	5.580289	1.042260	0.007189
O	3.845071	-2.116857	0.235765
O	1.077611	1.991269	-1.279608
O	1.854415	-0.484549	-0.395937
O	4.341964	5.419586	-1.104375
C	3.289184	1.412703	-0.572848
C	4.610226	1.888351	-0.371463
C	5.383884	-0.310599	0.200832
C	3.002165	0.037514	-0.320605
C	4.121350	-0.876563	0.054003
C	6.625991	-0.975205	0.585647
C	2.348746	2.402361	-1.008549
C	4.986378	3.211039	-0.537675
C	4.040782	4.186346	-0.940558
C	2.703593	3.715422	-1.173145
C	7.794845	-0.220389	0.818423
C	6.723200	-2.373367	0.734870
C	8.987313	-0.829242	1.182829
C	7.922793	-2.974184	1.097376
C	9.064282	-2.212477	1.326798
H	6.017530	3.496555	-0.359507
H	1.955678	4.428736	-1.509348
H	7.767485	0.856721	0.714886
H	5.849775	-2.983547	0.560672
H	9.865358	-0.215030	1.356436
H	7.961713	-4.054188	1.200410
H	9.997661	-2.686729	1.611274
H	0.552715	2.751057	-1.556810
Cu	0.034469	0.222512	0.074967
O	-1.850203	0.449995	0.744982
C	-2.953320	-0.070195	0.611915
C	-3.157683	-1.582333	0.321328
C	-4.194887	0.703948	0.867568
C	-4.229869	-1.616058	-0.721743
C	-1.910432	-2.317175	-0.101829
O	-4.070417	1.829244	1.473856
C	-5.380520	0.144857	0.403905
O	-5.379168	-1.011789	-0.389971
C	-4.075147	-2.173283	-1.930708
O	-1.029300	-2.405118	0.894306
C	-1.750715	-2.850779	-1.327927
C	-6.730699	0.661704	0.564085
C	-2.817850	-2.814627	-2.308873
H	-4.873589	-2.132219	-2.661699
H	-0.237572	-2.883091	0.612105
H	-0.836571	-3.375506	-1.584035
C	-7.818019	0.030261	-0.074365
C	-7.010416	1.785209	1.367918
O	-2.679946	-3.312083	-3.434515
C	-9.113564	0.501104	0.082339
H	-7.645688	-0.836754	-0.698558
C	-8.311166	2.247318	1.518716
H	-6.198180	2.287784	1.871378
C	-9.372356	1.613819	0.878608
H	-9.928322	-0.006791	-0.423523
H	-8.495072	3.113920	2.145641
H	-10.386161	1.980864	0.998093
O	-3.688740	-2.174951	1.495386
H	-3.047495	-2.083560	2.210687

SCF Energy: -2177.92636660
Sum of electronic and zero-point Energies= -2177.517898
Sum of electronic and thermal Energies= -2177.481517
Sum of electronic and thermal Enthalpies= -2177.480573
Sum of electronic and thermal Free Energies= -2177.592834

==> ./AOX-II/PA/PA/bis-/c4c5/RAF/c6 <==

O	-5.606439	-1.097735	-0.006750
O	-3.813990	1.983561	-0.575961
O	-1.032447	-2.384819	-0.501271
O	-1.813622	0.257004	-0.406102
O	-4.397497	-5.606970	0.349600
C	-3.279613	-1.620330	-0.211011
C	-4.628775	-2.016687	-0.020606
C	-5.390567	0.250301	-0.212966
C	-2.976045	-0.229914	-0.331274
C	-4.103409	0.748101	-0.386225
C	-6.644479	0.997410	-0.199663
C	-2.334055	-2.696065	-0.245540
C	-5.026099	-3.329943	0.169199
C	-4.075955	-4.381332	0.169595
C	-2.709747	-4.000370	-0.058221
C	-7.869258	0.330987	0.015110
C	-6.696683	2.392346	-0.397068
C	-9.073312	1.020631	0.031756
C	-7.907851	3.073743	-0.378559
C	-9.105876	2.399297	-0.164293
H	-6.077943	-3.549995	0.316697
H	-1.954750	-4.781165	-0.100238
H	-7.877166	-0.740174	0.170822
H	-5.777017	2.932487	-0.565507
H	-9.995741	0.473675	0.200158
H	-7.910915	4.148047	-0.534991
H	-10.048258	2.936653	-0.151111
H	-0.506434	-3.192458	-0.485760
Cu	-0.007872	-0.328660	0.240439
O	1.842674	-0.556511	0.971818
C	2.944753	-0.080251	0.630641
C	3.158708	1.324744	0.358754
C	4.094170	-1.020253	0.485606
C	4.458585	1.776941	-0.061618
C	2.201742	2.308995	0.536650
O	3.885861	-2.254168	0.740807
C	5.301916	-0.477520	0.061330
O	5.425261	0.886256	-0.195506
C	4.811705	3.096094	-0.287130
O	1.059893	2.087215	1.123081
C	2.414536	3.687513	0.002261
C	6.561921	-1.167725	-0.183083
C	3.870514	4.132903	-0.129770
H	5.848052	3.336113	-0.491489
H	0.575273	2.934050	1.179443
H	2.092407	3.591609	-1.050620
C	7.702075	-0.450114	-0.600253
C	6.697539	-2.560660	-0.016909
O	4.111848	5.348728	-0.171211
C	8.908785	-1.092193	-0.837255
H	7.642577	0.621575	-0.739391
C	7.910604	-3.193588	-0.258182
H	5.842403	-3.137735	0.301558
C	9.025573	-2.469728	-0.668807
H	9.766620	-0.508777	-1.156186
H	7.981454	-4.268023	-0.121368
H	9.970364	-2.969418	-0.854697
O	1.581299	4.578748	0.690491
H	1.266208	5.254184	0.083185

SCF Energy: -2177.95357556
Sum of electronic and zero-point Energies= -2177.544395
Sum of electronic and thermal Energies= -2177.508113
Sum of electronic and thermal Enthalpies= -2177.507169
Sum of electronic and thermal Free Energies= -2177.620217

==> ./AOX-II/PA/PA/bis-/c4c5/RAF/c4p <==

O	5.761883	-1.223295	-0.098910
O	4.346680	2.043880	0.498321
O	1.202414	-2.030492	1.072344
O	2.192398	0.509439	0.695070
O	4.113485	-5.603040	-0.033569
C	3.445988	-1.505880	0.434474
C	4.715398	-2.040508	0.094327
C	5.700382	0.147500	0.053553
C	3.288564	-0.088623	0.502313
C	4.496820	0.777520	0.353633
C	7.007223	0.763037	-0.158832
C	2.419524	-2.480457	0.661303
C	4.962091	-3.393146	-0.070024
C	3.928942	-4.345070	0.115822
C	2.647722	-3.822839	0.501025
C	8.126444	-0.033991	-0.479623
C	7.217337	2.153150	-0.054200
C	9.378856	0.527407	-0.683719
C	8.475722	2.705803	-0.260307
C	9.567522	1.903257	-0.576180
H	5.959677	-3.721910	-0.340852
H	1.838926	-4.523546	0.690548
H	8.013569	-1.106679	-0.569747
H	6.383283	2.793361	0.190450

H	10.216089	-0.118489	-0.929143
H	8.600542	3.780533	-0.170877
H	10.547645	2.340296	-0.735448
H	0.609899	-2.783739	1.177137
Cu	0.334965	0.188575	0.060294
O	-1.519341	-0.157984	-0.600679
C	-2.633401	0.383452	-0.427850
C	-2.905961	1.756272	-0.247079
C	-3.818206	-0.577796	-0.444782
C	-4.231467	2.180032	0.067948
C	-1.922811	2.806748	-0.316098
O	-3.598160	-1.756186	-0.702045
C	-5.141363	-0.041500	-0.153332
O	-5.251464	1.292138	0.144482
C	-4.563223	3.481715	0.333466
O	-0.670804	2.445587	-0.676838
C	-2.238194	4.110914	-0.052654
C	-6.328269	-0.744793	-0.107116
C	-3.572665	4.514217	0.293053
H	-5.588558	3.734824	0.576890
H	-0.100642	3.223940	-0.674126
H	-1.467190	4.872407	-0.122845
C	-7.562608	-0.047888	0.221709
C	-6.435234	-2.162961	-0.398666
O	-3.856391	5.723914	0.536819
C	-8.731489	-0.693763	0.340493
H	-7.529037	1.023271	0.364881
C	-7.603580	-2.813431	-0.276221
H	-5.557489	-2.693716	-0.731152
C	-8.855003	-2.169588	0.215127
H	-9.639612	-0.147567	0.574329
H	-7.663294	-3.871919	-0.511184
H	-9.050366	-2.562016	1.228300
O	-9.981350	-2.469784	-0.607609
H	-10.212593	-3.393870	-0.474146

SCF Energy: -2177.93817595
Sum of electronic and zero-point Energies= -2177.529882
Sum of electronic and thermal Energies= -2177.493437
Sum of electronic and thermal Enthalpies= -2177.492493
Sum of electronic and thermal Free Energies= -2177.604787

==> ./AOX-II/PA/PA/bis-/c4c5/RAF/c8a <==

O	4.991525	0.852368	0.251945
O	2.229997	-1.243512	-0.738660
O	1.585369	3.898924	-1.162632
O	1.273399	1.218602	-1.311366
O	5.768993	5.465819	0.419522
C	3.224627	2.283358	-0.493835
C	4.528668	2.095269	0.029257
C	4.255329	-0.286616	0.024806
C	2.420678	1.139206	-0.784245
C	2.968256	-0.210990	-0.484684
C	5.019330	-1.488151	0.364103
C	2.834857	3.650907	-0.680289
C	5.393810	3.129386	0.339114
C	5.002683	4.478297	0.145600
C	3.681702	4.686816	-0.377474
C	6.354618	-1.375425	0.801206
C	4.475078	-2.784257	0.275956
C	7.103241	-2.497110	1.128328
C	5.232316	-3.901767	0.606716
C	6.549769	-3.771479	1.033852
H	6.377054	2.900478	0.735552
H	3.345445	5.708705	-0.533269
H	6.811445	-0.397697	0.883854
H	3.454458	-2.905201	-0.054911
H	8.129324	-2.372786	1.459599
H	4.782247	-4.886339	0.527727
H	7.136477	-4.647453	1.289642
H	1.461205	4.852885	-1.215944
Cu	-0.476988	0.775362	-0.451350
O	-2.244630	0.287853	0.429747
C	-2.835788	-0.824375	0.439751
C	-2.320892	-2.115718	0.199323
C	-4.280939	-0.551745	0.828195
C	-3.190812	-3.223846	-0.090733
C	-0.907968	-2.374371	0.060129
O	-4.711642	-0.781946	1.932726
C	-5.111861	0.189191	-0.241260
O	-5.006625	-0.211591	-1.385823
C	-2.716604	-4.425310	-0.541897
O	-0.077714	-1.396433	0.450709
C	-0.432460	-3.574095	-0.406834
C	-6.022777	1.274800	0.165140
C	-1.313408	-4.653011	-0.739865
H	-3.412583	-5.226296	-0.772002
H	0.828934	-1.454216	0.023239
H	0.638718	-3.732840	-0.481850
C	-7.069214	1.623280	-0.698665
C	-5.838344	1.991984	1.353445
O	-0.877848	-5.764133	-1.168283
C	-7.927248	2.660510	-0.371498
H	-7.207440	1.066276	-1.618141
C	-6.689127	3.043876	1.667038
H	-5.022512	1.749001	2.023010
C	-7.735513	3.373314	0.811094
H	-8.744191	2.918149	-1.036036
H	-6.535890	3.605537	2.581484
H	-8.403662	4.189373	1.064155
O	-4.518506	-3.006959	0.066202
H	-5.016980	-3.776575	-0.230298

SCF Energy: -2177.98189510
Sum of electronic and zero-point Energies= -2177.575073
Sum of electronic and thermal Energies= -2177.538439
Sum of electronic and thermal Enthalpies= -2177.537495
Sum of electronic and thermal Free Energies= -2177.649808

==> ./AOX-II/PA/PA/bis-/c4c5/RAF/c3 <==

O	-5.680808	0.066869	-0.457447
O	-3.546455	-1.941585	1.651152
O	-1.555582	2.452581	-0.214527
O	-1.863781	-0.042694	0.887740
O	-5.175790	4.090000	-2.796534
C	-3.550761	1.148269	-0.314263
C	-4.885240	1.094017	-0.792891
C	-5.291630	-0.958772	0.380631
C	-3.054175	0.069156	0.478093
C	-3.995508	-1.017045	0.882899
C	-6.380999	-1.905070	0.600678
C	-2.814624	2.314412	-0.705753
C	-5.446520	2.053633	-1.618792
C	-4.696506	3.183808	-2.029699
C	-3.355108	3.272362	-1.524183
C	-7.612616	-1.740878	-0.067376
C	-6.264603	-3.003950	1.475585
C	-8.662288	-2.627814	0.125054
C	-7.322412	-3.885838	1.662736
C	-8.528708	-3.709836	0.992016
H	-6.473073	1.935526	-1.948632
H	-2.757721	4.141016	-1.788618
H	-7.746295	-0.909204	-0.747285
H	-5.336856	-3.156229	2.006368
H	-9.594699	-2.470005	-0.407966
H	-7.198473	-4.720795	2.345433
H	-9.350601	-4.402113	1.141362
H	-1.163784	3.259629	-0.567312
Cu	-0.096321	0.299526	0.028922
O	1.698873	0.164828	-0.856290
C	2.883563	0.342625	-0.497059
C	3.412726	1.283550	0.413959
C	3.877194	-0.595515	-1.133346
C	4.755556	1.149462	0.895031
C	2.675780	2.396631	0.942834
O	4.988868	0.040724	-1.772421
C	5.228914	-0.675870	-0.583759
O	5.558001	0.105626	0.515193
C	5.306788	2.004325	1.808385
O	1.426656	2.574311	0.461194
C	3.217222	3.257183	1.860885
C	6.074436	-1.901048	-0.644630
C	4.557321	3.101549	2.344096
H	6.322913	1.839733	2.147816
H	1.026794	3.348733	0.874099
H	2.626792	4.094368	2.221560
C	6.347249	-2.605000	0.529564
C	6.570206	-2.366376	-1.860892
O	5.056510	3.896693	3.195742
C	7.113210	-3.763624	0.483647
H	5.961619	-2.248154	1.477740
C	7.339843	-3.524065	-1.901330
H	6.357245	-1.823542	-2.774184
C	7.612348	-4.224926	-0.731052
H	7.320376	-4.306288	1.399560
H	7.727473	-3.877010	-2.850793
H	8.211584	-5.128473	-0.764720
O	3.320236	-1.637814	-1.802844
H	2.382255	-1.411887	-1.917242

SCF Energy: -2177.96835573
Sum of electronic and zero-point Energies= -2177.558842
Sum of electronic and thermal Energies= -2177.523228
Sum of electronic and thermal Enthalpies= -2177.522284
Sum of electronic and thermal Free Energies= -2177.632098

==> ./AOX-II/PA/PA/bis-/c4c5/RAF/c4 <==

O	-5.318450	-0.813264	-0.173460
O	-3.092409	1.819894	-1.245087
O	-0.945257	-2.730093	-0.032118
O	-1.361926	-0.068811	-0.573513
O	-4.766245	-5.263615	1.168358
C	-3.080311	-1.655340	-0.092071
C	-4.478782	-1.830759	0.070269
C	-4.909534	0.424854	-0.626541
C	-2.585080	-0.374017	-0.479101
C	-3.560078	0.703705	-0.819122
C	-6.048162	1.315036	-0.831472
C	-2.288986	-2.821571	0.170013
C	-5.063009	-3.014457	0.488963
C	-4.268118	-4.152126	0.774335
C	-2.852133	-3.999912	0.586115
C	-7.363161	0.848691	-0.622554
C	-5.897229	2.658581	-1.229718
C	-8.460452	1.678783	-0.801885
C	-7.002520	3.482820	-1.404013
C	-8.292265	3.004754	-1.193985
H	-6.141149	-3.065506	0.596551
H	-2.213030	-4.860447	0.764857
H	-7.526330	-0.176675	-0.316306

H	-4.903910	3.046990	-1.397052
H	-9.457577	1.284245	-0.633135
H	-6.849073	4.513473	-1.708591
H	-9.151531	3.652390	-1.332697
H	-0.536408	-3.575945	0.184450
Cu	0.194445	-0.540825	0.562198
O	4.129554	-0.530612	2.152072
C	3.092190	-0.133617	1.381765
C	2.935735	1.359373	1.194161
C	3.597088	-0.782908	0.119876
C	3.833392	1.986390	0.330452
C	2.087928	2.250397	1.878706
O	3.213177	-1.812666	-0.390717
C	4.820070	-0.058133	-0.405160
O	4.811834	1.223279	-0.345962
C	3.927078	3.334713	0.077316
O	1.215101	1.740659	2.794226
C	2.129709	3.621338	1.651177
C	5.923489	-0.679309	-1.071309
C	3.039397	4.230673	0.744457
H	4.671230	3.707185	-0.616793
H	0.713518	2.465304	3.182448
H	1.443670	4.260424	2.201388
C	6.838082	0.109775	-1.800195
C	6.130488	-2.069472	-0.964534
O	3.075707	5.498110	0.545274
C	7.918856	-0.484172	-2.419501
H	6.679310	1.177153	-1.885751
C	7.226748	-2.648805	-1.576787
H	5.448655	-2.680826	-0.389990
C	8.114360	-1.861817	-2.307349
H	8.614160	0.119299	-2.990434
H	7.393050	-3.715307	-1.485647
H	8.967321	-2.323520	-2.792000
O	1.839936	-0.708828	1.776866
H	2.012280	-1.637566	1.973627

SCF Energy: -2177.90317718
Sum of electronic and zero-point Energies= -2177.495631
Sum of electronic and thermal Energies= -2177.459238
Sum of electronic and thermal Enthalpies= -2177.458294
Sum of electronic and thermal Free Energies= -2177.570351

==> ./AOX-II/PA/PA/bis-/c4c5/RAF/c3p <==

O	5.690983	-1.091463	0.490105
O	4.132134	2.044577	-0.381960
O	1.318823	-2.269605	-1.041223
O	2.173045	0.353256	-0.896916
O	4.365508	-5.578692	0.439398
C	3.471040	-1.558601	-0.279154
C	4.728093	-1.986753	0.222269
C	5.545814	0.268245	0.300175
C	3.243919	-0.161322	-0.476994
C	4.352517	0.797013	-0.178872
C	6.762960	0.987529	0.663219
C	2.534626	-2.613083	-0.536297
C	5.050120	-3.311304	0.468375
C	4.110942	-4.342914	0.222269
C	2.838162	-3.928296	-0.296983
C	7.896126	0.284653	1.124069
C	6.868177	2.390474	0.574479
C	9.064079	0.947471	1.473399
C	8.042069	3.045003	0.927773
C	9.150008	2.334531	1.379234
H	6.034015	-3.554554	0.854844
H	2.094256	-4.691106	-0.510910
H	7.860707	-0.793913	1.208778
H	6.018068	2.957534	0.225779
H	9.916096	0.372698	1.822907
H	8.087575	4.126716	0.847320
H	10.064139	2.850819	1.652894
H	0.781498	-3.064216	-1.135269
Cu	0.232058	0.015684	-1.161808
O	-1.715396	-0.270928	-1.437971
C	-2.749912	0.228591	-0.923935
C	-2.939190	1.604866	-0.606254
C	-3.870440	-0.732197	-0.644047
C	-4.133004	2.011345	0.050568
C	-2.006867	2.662347	-0.876644
O	-3.712681	-1.951633	-0.977071
C	-5.000269	-0.217610	-0.016665
O	-5.093659	1.112965	0.331672
C	-4.388997	3.310471	0.443998
O	-0.876516	2.340661	-1.558263
C	-2.244670	3.954753	-0.487823
C	-6.183404	-0.951931	0.379209
C	-3.447486	4.342968	0.194492
H	-5.323718	3.540294	0.943617
H	-0.333437	3.131155	-1.656270
H	-1.511808	4.722296	-0.721029
C	-7.228801	-0.310227	1.038129
C	-6.372479	-2.327350	0.087635
O	-3.651833	5.556140	0.537395
C	-8.370028	-1.044161	1.538605
H	-7.184469	0.743027	1.282512
C	-7.508803	-3.043769	0.468604
H	-5.587316	-2.835783	-0.453969
C	-8.541350	-2.426180	1.126567
H	-7.806985	-1.324366	2.500261
H	-7.582600	-4.092281	0.208754
H	-9.438921	-2.951204	1.432173
O	-9.476344	-0.260309	1.826485

H -10.087001 -0.759093 2.380439

SCF Energy: -2177.89618984
Sum of electronic and zero-point Energies= -2177.490184
Sum of electronic and thermal Energies= -2177.453860
Sum of electronic and thermal Enthalpies= -2177.452915
Sum of electronic and thermal Free Energies= -2177.563955

==> ./AOX-II/PA/PA/bis-/c4c5/RAF/c5p <==

O	-5.616306	-1.194841	0.140860
O	-4.116747	1.938176	-0.842311
O	-1.148345	-2.305150	-1.147730
O	-2.073410	0.276805	-1.155689
O	-4.161907	-5.637886	0.354120
C	-3.353281	-1.629423	-0.506987
C	-4.618152	-2.073258	-0.040942
C	-5.505043	0.155882	-0.118241
C	-3.160254	-0.238525	-0.762802
C	-4.309489	0.696357	-0.581084
C	-6.752196	0.855743	0.174648
C	-2.372576	-2.662987	-0.674261
C	-4.911196	-3.393628	0.255990
C	-3.931568	-4.404931	0.097181
C	-2.648619	-3.974349	-0.383046
C	-7.858482	0.152086	0.695676
C	-6.913485	2.239233	-0.041590
C	-9.053371	0.795978	0.983533
C	-8.114805	2.874771	0.249018
C	-9.194717	2.164110	0.763520
H	-5.903933	-3.650895	0.609342
H	-1.875285	-4.723614	-0.530327
H	-7.780080	-0.911899	0.878615
H	-6.086374	2.807672	-0.439489
H	-9.882498	0.221459	1.384613
H	-8.203988	3.941625	0.068828
H	-10.129941	2.665684	0.989041
H	-0.590709	-3.090415	-1.185149
Cu	-0.289195	0.212344	-0.292002
O	1.493608	-0.090988	0.545927
C	2.625350	0.444152	0.417109
C	2.871897	1.840602	0.278878
C	3.799805	-0.490762	0.425062
C	4.199255	2.298280	0.053976
C	1.874873	2.871845	0.326622
O	3.583699	-1.731708	0.614142
C	5.054909	0.070786	0.209948
O	5.222518	1.424989	0.019426
C	4.530001	3.623580	-0.151550
O	0.599416	2.497546	0.607611
C	2.187371	4.189712	0.119827
C	6.314526	-0.636968	0.142404
C	3.529511	4.630706	-0.138857
H	5.565995	3.892569	-0.325993
H	0.035376	3.279607	0.600401
H	1.398686	4.935709	0.168710
C	7.538277	0.058934	-0.026337
C	6.401732	-2.022558	0.266955
O	3.793110	5.864918	-0.334436
C	8.781018	-0.571356	-0.101920
H	7.511500	1.137773	-0.107263
C	7.659014	-2.712780	0.076679
H	5.511789	-2.620345	0.407745
C	8.882064	-1.934814	0.010578
H	9.673019	0.030176	-0.223419
H	9.829548	-2.458106	-0.041632
H	7.497802	-2.708174	-1.062339
O	7.681164	-4.008431	0.566262
H	8.447138	-4.472770	0.210994

SCF Energy: -2177.89679189
Sum of electronic and zero-point Energies= -2177.491293
Sum of electronic and thermal Energies= -2177.454858
Sum of electronic and thermal Enthalpies= -2177.453914
Sum of electronic and thermal Free Energies= -2177.566328

==> ./AOX-II/PA/PA/bis-/c4c5/RAF/c7 <==

O	5.585881	-1.066316	-0.014907
O	3.912763	2.046462	0.725899
O	1.043230	-2.259752	0.851525
O	1.867735	0.356006	0.713181
O	4.255559	-5.546495	-0.302191
C	3.275199	-1.543812	0.382934
C	4.594239	-1.965967	0.075467
C	5.416721	0.284297	0.216558
C	3.013091	-0.148943	0.537572
C	4.160062	0.806419	0.506189
C	6.679505	1.005327	0.087361
C	2.310401	-2.598765	0.488026
C	4.945766	-3.284978	-0.158738
C	3.975879	-4.315914	-0.086688
C	2.642430	-3.909064	0.258683
C	7.860054	0.315322	-0.260178
C	6.785173	2.395299	0.297491
C	9.072231	0.977746	-0.390833
C	8.004136	3.049516	0.164202
C	9.157663	2.351967	-0.180377
H	5.976093	-3.525735	-0.397586
H	1.877094	-4.674732	0.355917

H	7.825606	-0.753027	-0.430856
H	5.901590	2.954490	0.567055
H	9.959144	0.412841	-0.660453
H	8.048832	4.120800	0.334390
H	10.106396	2.868115	-0.283046
H	0.497146	-3.054076	0.859765
Cu	0.119246	-0.069558	-0.155721
O	-1.642750	-0.468942	-1.028956
C	-2.783744	-0.020971	-0.750432
C	-3.093386	1.368441	-0.527237
C	-3.885241	-1.017768	-0.642508
C	-4.372029	1.680132	-0.117193
C	-2.135533	2.482117	-0.625165
O	-3.620510	-2.232222	-0.906782
C	-5.131784	-0.554657	-0.203658
O	-5.309855	0.778645	0.052955
C	-4.806351	3.041790	0.233067
O	-0.924862	2.166127	-1.141256
C	-2.436296	3.739121	-0.238125
C	-6.340707	-1.326563	0.070767
C	-3.791165	4.088536	0.194266
H	-5.642151	3.113515	0.922814
H	-0.374961	2.958233	-1.167042
H	-1.692876	4.527700	-0.299485
C	-7.454943	-0.710360	0.673541
C	-6.444939	-2.692253	-0.251647
O	-4.808465	4.016557	-0.814530
C	-8.611425	-1.427075	0.943694
H	-7.413800	0.339114	0.937041
C	-7.608941	-3.401222	0.021517
H	-5.608832	-3.190036	-0.719950
C	-8.698839	-2.779251	0.620928
H	-9.451127	-0.924118	1.412279
H	-7.660564	-4.453155	-0.240407
H	-9.604102	-3.337843	0.833687
O	-3.932739	5.138238	1.075334
H	-3.392884	5.877838	0.772688

SCF Energy: -2177.92747848
Sum of electronic and zero-point Energies= -2177.518060
Sum of electronic and thermal Energies= -2177.482429
Sum of electronic and thermal Enthalpies= -2177.481485
Sum of electronic and thermal Free Energies= -2177.591093

==> ./AOX-II/PA/PA/bis-/c4c5/RAF/c2p <==

O	5.777603	1.046439	0.127833
O	4.155610	-2.090804	-0.622830
O	1.268877	2.197070	-0.949761
O	2.116728	-0.407341	-0.807608
O	4.413272	5.522141	0.278663
C	3.485259	1.502966	-0.386051
C	4.785908	1.937777	-0.022450
C	5.624339	-0.310321	-0.076274
C	3.241440	0.105325	-0.547596
C	4.386646	-0.844839	-0.419604
C	6.883387	-1.020902	0.125860
C	2.519683	2.550229	-0.545570
C	5.119795	3.262530	0.205107
C	4.149272	4.286529	0.072153
C	2.833964	3.865937	-0.322551
C	8.060537	-0.308702	0.438126
C	6.987287	-2.422976	0.024997
C	9.268620	-0.961830	0.636547
C	8.201556	-3.068004	0.227369
C	9.352419	-2.348395	0.533448
H	6.136965	3.512935	0.486553
H	2.068271	4.624814	-0.460539
H	8.027003	0.769733	0.525375
H	6.104927	-2.998353	-0.211493
H	10.153670	-0.379970	0.874307
H	8.244672	-4.149526	0.143263
H	10.297829	-2.857283	0.689316
H	0.718000	2.986902	-0.992494
Cu	0.263170	-0.029977	-0.180133
O	-1.573793	0.376009	0.512198
C	-2.704458	-0.138800	0.368064
C	-3.019149	-1.507058	0.222969
C	-3.860397	0.856232	0.367620
C	-4.364233	-1.902022	-0.043253
C	-2.061608	-2.582154	0.274623
O	-3.604611	2.038787	0.555197
C	-5.211999	0.344544	0.150088
O	-5.368269	-0.992015	-0.084832
C	-4.737097	-3.196746	-0.282531
O	-0.790192	-2.249874	0.589884
C	-2.417203	-3.881002	0.036406
C	-6.375926	1.078880	0.124617
C	-3.770859	-4.254216	-0.263204
H	-5.775731	-3.425959	-0.490593
H	-0.240820	-3.042795	0.575423
H	-1.662762	-4.660369	0.090300
C	-7.674127	0.326017	-0.102059
C	-6.432792	2.501149	0.295737
O	-4.092528	-5.457543	-0.486964
C	-8.858039	1.191257	-0.394962
H	-7.525427	-0.356150	-0.947806
C	-7.588224	3.183547	0.091366
H	-5.537344	3.033322	0.573801
C	-8.813864	2.527142	-0.298940
H	-9.775080	0.669899	-0.651763
H	-7.594595	4.260311	0.221672
H	-9.694667	3.129262	-0.492835

O	-7.931894	-0.454481	1.075256
H	-8.604085	-1.107122	0.854755

SCF Energy: -2177.93217439
Sum of electronic and zero-point Energies= -2177.523867
Sum of electronic and thermal Energies= -2177.487293
Sum of electronic and thermal Enthalpies= -2177.486349
Sum of electronic and thermal Free Energies= -2177.600294

==> ./AOX-II/PA/PA/bis-/c4c5/vEA <==

O	5.397713	-1.124364	-0.035103
O	3.959728	2.136566	0.527400
O	0.737392	-1.878339	0.641628
O	1.788324	0.634099	0.442682
O	3.693701	-5.482999	-0.203929
C	3.044594	-1.390442	0.254657
C	4.336337	-1.937273	0.040938
C	5.330800	0.240057	0.127340
C	2.900004	0.017136	0.347449
C	4.110354	0.877928	0.348138
C	6.651040	0.854269	0.012921
C	1.990811	-2.351763	0.344939
C	4.578726	-3.290398	-0.114270
C	3.517723	-4.228289	-0.056361
C	2.203349	-3.690480	0.191379
C	7.792364	0.049609	-0.184390
C	6.847349	2.247663	0.083393
C	9.056537	0.608441	-0.302903
C	8.117556	2.798081	-0.040109
C	9.232189	1.988383	-0.232535
H	5.593640	-3.631855	-0.285075
H	1.368061	-4.379392	0.275069
H	7.687821	-1.025981	-0.244789
H	5.994831	2.892741	0.232594
H	9.912543	-0.041887	-0.452274
H	8.233086	3.875935	0.016077
H	10.221490	2.423553	-0.326460
H	0.084910	-2.589919	0.611560
Cu	-0.000046	0.001009	-0.002248
O	-1.788179	-0.632286	-0.447876
C	-2.900106	-0.016030	-0.350825
C	-3.045256	1.391358	-0.256179
C	-4.110005	-0.877423	-0.351278
C	-4.337047	1.937314	-0.040537
C	-1.992015	2.353272	-0.346567
O	-3.958956	-2.135752	-0.532304
C	-5.330461	-0.240476	-0.127792
O	-5.397879	1.123737	0.036130
C	-4.580007	3.290185	0.115961
O	-0.738609	1.880717	-0.644642
C	-2.205139	3.691758	-0.191761
C	-6.650036	-0.855657	-0.011038
C	-3.519565	4.228687	0.057599
H	-5.594912	3.630962	0.288166
H	-0.086545	2.592703	-0.615121
H	-1.370269	4.381157	-0.275588
C	-7.791012	-0.052208	0.193145
C	-6.846004	-2.248887	-0.085776
O	-3.696056	5.483198	0.206262
C	-9.054502	-0.612023	0.314238
H	-7.686710	1.023204	0.257058
C	-8.115541	-2.800289	0.040200
H	-5.993736	-2.893053	-0.240236
C	-9.229818	-1.991787	0.239550
H	-9.910240	0.037372	0.469112
H	-8.230812	-3.877981	-0.019515
H	-10.218588	-2.427728	0.335460

SCF Energy: -2102.27776851

==> ./AOX-II/PA/PA/bis-/c4c5/vIP <==

O	5.397713	-1.124364	-0.035103
O	3.959728	2.136566	0.527400
O	0.737392	-1.878339	0.641628
O	1.788324	0.634099	0.442682
O	3.693701	-5.482999	-0.203929
C	3.044594	-1.390442	0.254657
C	4.336337	-1.937273	0.040938
C	5.330800	0.240057	0.127340
C	2.900004	0.017136	0.347449
C	4.110354	0.877928	0.348138
C	6.651040	0.854269	0.012921
C	1.990811	-2.351763	0.344939
C	4.578726	-3.290398	-0.114270
C	3.517723	-4.228289	-0.056361
C	2.203349	-3.690480	0.191379
C	7.792364	0.049609	-0.184390
C	6.847349	2.247663	0.083393
C	9.056537	0.608441	-0.302903
C	8.117556	2.798081	-0.040109
C	9.232189	1.988383	-0.232535
H	5.593640	-3.631855	-0.285075
H	1.368061	-4.379392	0.275069
H	7.687821	-1.025981	-0.244789
H	5.994831	2.892741	0.232594
H	9.912543	-0.041887	-0.452274
H	8.233086	3.875935	0.016077
H	10.221490	2.423553	-0.326460
H	0.084910	-2.589919	0.611560
Cu	-0.000046	0.001009	-0.002248
O	-1.788179	-0.632286	-0.447876

C	-2.900106	-0.016030	-0.350825
C	-3.045256	1.391358	-0.256179
C	-4.110005	-0.877423	-0.351278
C	-4.337047	1.937314	-0.040537
C	-1.992015	2.353272	-0.346567
O	-3.958956	-2.135752	-0.532304
C	-5.330461	-0.240476	-0.127792
O	-5.397879	1.123737	0.036130
C	-4.580007	3.290185	0.115961
O	-0.738609	1.880717	-0.644642
C	-2.205139	3.691758	-0.191761
C	-6.650036	-0.855657	-0.011038
C	-3.519565	4.228687	0.057599
H	-5.594912	3.630962	0.288166
H	-0.086545	2.592703	-0.615121
H	-1.370269	4.381157	-0.275588
C	-7.791012	-0.052208	0.193145
C	-6.846004	-2.248887	-0.085776
O	-3.696056	5.483198	0.206262
C	-9.054502	-0.612023	0.314238
H	-7.686710	1.023204	0.257058
C	-8.115541	-2.800289	0.040200
H	-5.993736	-2.893053	-0.240236
C	-9.229818	-1.991787	0.239550
H	-9.910240	0.037372	0.469112
H	-8.230812	-3.877981	-0.019515
H	-10.218588	-2.427728	0.335460

SCF Energy: -2101.96504229

==> ./AOX-II/PA/PA/bis-/c4c5/aEA <==

O	5.562344	-1.047941	-0.068441
O	3.880399	2.074123	0.609201
O	1.013552	-2.235030	0.772351
O	1.842961	0.381171	0.642046
O	4.242016	-5.533989	-0.295618
C	3.248753	-1.522433	0.319566
C	4.570757	-1.946869	0.027759
C	5.389532	0.306625	0.137050
C	2.984652	-0.126237	0.461576
C	4.130616	0.831290	0.411115
C	6.652922	1.026515	0.007406
C	2.284968	-2.577479	0.430279
C	4.926570	-3.268937	-0.182142
C	3.958248	-4.300606	-0.101691
C	2.621150	-3.890646	0.224353
C	7.841304	0.328860	-0.295566
C	6.752047	2.422990	0.173438
C	9.054371	0.989896	-0.425441
C	7.971731	3.075875	0.040544
C	9.133046	2.370593	-0.259350
H	5.959416	-3.511281	-0.408206
H	1.855518	-4.655618	0.324577
H	7.812502	-0.744673	-0.431089
H	5.862778	2.988561	0.408356
H	9.947400	0.418728	-0.659337
H	8.010723	4.152428	0.175205
H	10.082384	2.885711	-0.361652
H	0.468679	-3.030075	0.788814
Cu	0.000060	-0.000016	0.000382
O	-1.842810	-0.381088	-0.641412
C	-2.984553	0.126279	-0.461179
C	-3.248720	1.522460	-0.319139
C	-4.130520	-0.831267	-0.411176
C	-4.570820	1.946852	-0.027708
C	-2.284929	2.577534	-0.429541
O	-3.880191	-2.074087	-0.609203
C	-5.389517	-0.306653	-0.137369
O	-5.562408	1.047893	0.068192
C	-4.926728	3.268904	0.182131
O	-1.013403	2.235132	-0.771245
C	-2.621207	3.890686	-0.223677
C	-6.652932	-1.026559	-0.008045
C	-3.958403	4.300597	0.102023
H	-5.959649	3.511217	0.407887
H	-0.468567	3.030206	-0.787584
H	-1.855569	4.655682	-0.323670
C	-7.841381	-0.328904	0.294662
C	-6.752032	-2.423031	-0.174138
O	-4.242280	5.533979	0.295803
C	-9.054486	-0.989932	0.424232
H	-7.812602	0.744625	0.430218
C	-7.971753	-3.075908	-0.041547
H	-5.862715	-2.988602	-0.408868
C	-9.133135	-2.370624	0.258087
H	-9.947565	-0.418761	0.657929
H	-8.010724	-4.152457	-0.176247
H	-10.082502	-2.885736	0.360148

SCF Energy: -2102.29251817
Sum of electronic and zero-point Energies= -2101.900380
Sum of electronic and thermal Energies= -2101.865689
Sum of electronic and thermal Enthalpies= -2101.864744
Sum of electronic and thermal Free Energies= -2101.973573

==> ./AOX-II/PA/PA/bis-/c4c5/aIP <==

O	5.483864	-1.111930	0.079774
O	3.973457	2.089927	0.168004
O	0.756000	-1.938858	0.032692
O	1.811073	0.619881	-0.085375
O	3.868989	-5.485397	0.033910

C	3.081805	-1.409710	0.023641
C	4.402759	-1.942676	0.060591
C	5.405380	0.220868	0.104821
C	2.897508	-0.015722	-0.004622
C	4.118915	0.863083	0.091720
C	6.696146	0.876982	0.164666
C	2.035198	-2.395787	0.015298
C	4.690817	-3.276436	0.062961
C	3.633221	-4.249121	0.035215
C	2.289878	-3.734210	0.015890
C	7.834671	0.123564	0.516507
C	6.860667	2.242661	-0.137347
C	9.083105	0.718565	0.578138
C	8.117776	2.825164	-0.081529
C	9.231632	2.071957	0.279643
H	5.722040	-3.606868	0.084748
H	1.464249	-4.438451	0.013228
H	7.731339	-0.927281	0.754534
H	6.007149	2.836229	-0.428293
H	9.945538	0.125564	0.861309
H	8.228161	3.875816	-0.325849
H	10.210995	2.535362	0.326185
H	0.121319	-2.666680	-0.003819
Cu	-0.035952	0.015052	-0.359420
O	-1.854786	-0.591971	-0.631893
C	-2.957380	0.010246	-0.408255
C	-3.105636	1.410717	-0.253866
C	-4.150478	-0.870303	-0.321295
C	-4.387664	1.935380	0.057454
C	-2.068401	2.387385	-0.371286
O	-3.993320	-2.126040	-0.511585
C	-5.363399	-0.250784	-0.019960
O	-5.435217	1.108753	0.168649
C	-4.635088	3.279625	0.268024
O	-0.825461	1.945914	-0.748649
C	-2.286725	3.717635	-0.162818
C	-6.666986	-0.883994	0.162344
C	-3.590115	4.232471	0.174404
H	-5.641852	3.602535	0.508523
H	-0.180774	2.664529	-0.715882
H	-1.464017	4.418230	-0.270319
C	-7.795195	-0.102594	0.487213
C	-6.860221	-2.272999	0.026471
O	-3.771037	5.480208	0.365510
C	-9.044082	-0.679461	0.665461
H	-7.692531	0.968791	0.601270
C	-8.115362	-2.841413	0.208027
H	-6.017401	-2.900285	-0.220993
C	-9.217076	-2.054688	0.527727
H	-9.890321	-0.047275	0.914799
H	-8.229279	-3.915094	0.096387
H	-10.194552	-2.503974	0.667630

SCF Energy: -2101.97929864

Sum of electronic and zero-point Energies= -2101.584195

Sum of electronic and thermal Energies= -2101.550526

Sum of electronic and thermal Enthalpies= -2101.549582

Sum of electronic and thermal Free Energies= -2101.653042

==> ./AOX-II/PA/PA/bis-/c4c5/HAT/c5 <==

O	5.426195	-1.106330	-0.045460
O	4.003802	2.135322	0.659710
O	0.767477	-1.903272	0.631814
O	1.816703	0.631130	0.531653
O	3.726648	-5.451629	-0.420497
C	3.069958	-1.381274	0.252124
C	4.358056	-1.916121	-0.002933
C	5.365753	0.251926	0.187413
C	2.920558	0.031457	0.404007
C	4.147908	0.882534	0.429261
C	6.693584	0.856733	0.131816
C	2.017320	-2.348868	0.305751
C	4.602379	-3.260071	-0.231864
C	3.544950	-4.202538	-0.210964
C	2.237025	-3.680963	0.077021
C	7.836114	0.047875	-0.043745
C	6.900110	2.246397	0.244102
C	9.108852	0.597623	-0.097434
C	8.178782	2.787817	0.186118
C	9.294166	1.973303	0.017626
H	5.617272	-3.588889	-0.427575
H	1.403088	-4.375014	0.135369
H	7.725557	-1.024858	-0.136192
H	6.047678	2.895745	0.375149
H	9.964410	-0.057159	-0.229977
H	8.300740	3.862877	0.274791
H	10.290208	2.401232	-0.024416
H	0.141192	-2.636346	0.596056
Cu	-0.051394	0.069637	-0.135803
O	-1.880508	-0.691259	-0.751569
C	-2.943380	-0.047985	-0.563888
C	-3.078393	1.350558	-0.449937
C	-4.208324	-0.851577	-0.552409
C	-4.333223	1.921137	-0.154727
C	-1.948990	2.285168	-0.555221
O	-4.201343	-2.027444	-0.903234
C	-5.446055	-0.166828	-0.150150
O	-5.444909	1.131139	0.001142
C	-4.549268	3.253578	0.051180
O	-0.796148	1.863505	-0.902810
C	-2.177118	3.650774	-0.288035
C	-6.702615	-0.810121	0.136101

C	-3.448553	4.196960	-0.014334
H	-5.545229	3.607280	0.291061
H	-1.328371	4.323212	-0.366176
C	-7.872982	-0.023448	0.206247
C	-6.798943	-2.197480	0.367935
O	-3.667049	5.435191	0.177855
C	-9.094791	-0.610257	0.472554
H	-7.815874	1.042197	0.025426
C	-8.027786	-2.769953	0.649347
H	-5.912625	-2.813729	0.349525
C	-9.176719	-1.984826	0.695853
H	-9.988923	0.001253	0.505946
H	-8.089339	-3.835354	0.838144
H	-10.136552	-2.441925	0.909435

SCF Energy: -2101.50148918
Sum of electronic and zero-point Energies= -2101.120843
Sum of electronic and thermal Energies= -2101.086705
Sum of electronic and thermal Enthalpies= -2101.085761
Sum of electronic and thermal Free Energies= -2101.192120

==> ./AOX-II/PA/PA/bis-/c3c4/aEA <==

O	-5.300842	-0.661999	0.000879
O	-1.705303	-0.984609	-0.000754
O	-3.606314	3.809914	-0.003103
O	-2.024951	1.723196	-0.002851
O	-8.257036	2.992821	-0.000404
C	-4.371251	1.529605	-0.001121
C	-5.490318	0.671365	0.000052
C	-4.053472	-1.250227	0.000610
C	-3.053727	0.992575	-0.001516
C	-2.900865	-0.479758	-0.000440
C	-4.158417	-2.709949	0.001392
C	-4.642804	2.933200	-0.002008
C	-6.794087	1.131505	0.000355
C	-7.063173	2.528885	-0.000611
C	-5.928365	3.410349	-0.001706
C	-5.426413	-3.325788	0.001775
C	-3.030544	-3.553625	0.001750
C	-5.557425	-4.707078	0.002471
C	-3.171834	-4.936374	0.002458
C	-4.431355	-5.526373	0.002815
H	-7.615576	0.423164	0.001210
H	-6.098802	4.482354	-0.002392
H	-6.319795	-2.715041	0.001503
H	-2.043695	-3.116681	0.001488
H	-6.549902	-5.146376	0.002740
H	-2.281484	-5.557224	0.002724
H	-4.535261	-6.606263	0.003357
H	-2.784008	3.269761	-0.003049
Cu	-0.005886	-0.027070	-0.000579
O	2.070280	-1.772596	-0.002038
C	3.081955	-1.018896	-0.001132
C	4.411266	-1.526991	-0.000833
C	2.897325	0.449940	-0.000200
C	5.511451	-0.644804	-0.000051
C	4.713852	-2.924301	-0.001649
O	1.692235	0.930742	-0.000307
C	4.033435	1.244911	0.000621
O	5.293296	0.684053	0.000562
C	6.825061	-1.076134	0.000139
O	3.696948	-3.823678	-0.002496
C	6.009590	-3.373141	-0.001466
C	4.107213	2.706578	0.001440
C	7.124922	-2.467162	-0.000593
H	7.630636	-0.349734	0.000732
H	2.863013	-3.301533	-0.002529
H	6.203264	-4.441198	-0.002073
C	5.361888	3.349277	0.001229
C	2.961680	3.526090	0.002532
O	8.328648	-2.904924	-0.000547
C	5.463468	4.733024	0.002029
H	6.267713	2.757091	0.000427
C	3.073528	4.911521	0.003341
H	1.984438	3.068034	0.002763
C	4.320213	5.528195	0.003088
H	6.446347	5.193419	0.001812
H	2.170201	5.513328	0.004197
H	4.401135	6.610050	0.003721

SCF Energy: -2102.32532479
Sum of electronic and zero-point Energies= -2101.932896
Sum of electronic and thermal Energies= -2101.899576
Sum of electronic and thermal Enthalpies= -2101.898632
Sum of electronic and thermal Free Energies= -2102.003306

==> ./AOX-II/PA/PA/bis-/c3c4/HAT/c5 <==

O	-4.991401	-0.506065	0.066504
O	-1.759619	-0.485594	-1.236316
O	-3.416392	3.994043	0.741571
O	-1.676528	1.847495	-0.004969
O	-7.945098	2.842804	1.376739
C	-4.051315	1.727481	0.358588
C	-5.151301	0.837122	0.360033
C	-3.861159	-1.026406	-0.326855
C	-2.776951	1.248641	-0.010783
C	-2.744875	-0.139378	-0.558037
C	-3.856908	-2.455676	-0.512197

C	-4.337066	3.130099	0.743629
C	-6.424703	1.176650	0.701723
C	-6.743312	2.546069	1.080314
C	-5.671694	3.456202	1.093515
C	-5.083689	-3.142271	-0.610410
C	-2.653043	-3.185625	-0.558254
C	-5.100643	-4.514278	-0.776480
C	-2.684923	-4.561486	-0.707207
C	-3.902734	-5.226959	-0.824153
H	-7.203500	0.423165	0.708730
H	-5.873712	4.488774	1.363468
H	-6.013760	-2.589219	-0.578301
H	-1.702949	-2.680807	-0.452514
H	-6.047235	-5.033571	-0.869509
H	-1.755930	-5.118934	-0.728386
H	-3.920163	-6.303863	-0.949320
Cu	-0.037222	0.351744	-0.674604
O	1.292662	-1.231735	-0.148167
C	2.487260	-0.805543	-0.027905
C	3.558497	-1.644738	0.352205
C	2.747646	0.616514	-0.294846
C	4.855912	-1.099533	0.480828
C	3.417090	-3.041600	0.628156
O	1.735081	1.348442	-0.661144
C	4.044931	1.073830	-0.137715
O	5.049427	0.211407	0.238410
C	5.952831	-1.851237	0.851296
O	2.195530	-3.624675	0.521206
C	4.494223	-3.803232	0.996690
C	4.548080	2.433827	-0.326505
C	5.812117	-3.241657	1.124779
H	6.925860	-1.380225	0.937072
H	1.561822	-2.926936	0.255363
H	4.355160	-4.860366	1.199141
C	5.908520	2.722206	-0.102658
C	3.715276	3.494609	-0.729206
O	6.805648	-3.968158	1.469617
C	6.407966	4.005486	-0.272469
H	6.581253	1.933472	0.208253
C	4.224638	4.776757	-0.896382
H	2.667533	3.306462	-0.909484
C	5.570844	5.044512	-0.670850
H	7.461189	4.194202	-0.091262
H	3.557212	5.573975	-1.207490
H	5.963060	6.047295	-0.802905

SCF Energy: -2101.51896079
Sum of electronic and zero-point Energies= -2101.137631
Sum of electronic and thermal Energies= -2101.103856
Sum of electronic and thermal Enthalpies= -2101.102912
Sum of electronic and thermal Free Energies= -2101.208114

==> ./AOX-II/PA/PA/bis-/c3c4/aIP <==

O	-5.069398	-0.284679	0.058154
O	-1.603710	-1.171196	-0.110970
O	-2.605491	3.834457	-0.096190
O	-1.413273	1.436248	-0.130328
O	-7.310441	3.827760	0.139137
C	-3.761087	1.723013	-0.025182
C	-5.016114	1.070507	0.044019
C	-3.982485	-1.086446	0.009094
C	-2.595900	0.947252	-0.074667
C	-2.707119	-0.508941	-0.059515
C	-4.316743	-2.501874	0.036517
C	-3.774196	3.159018	-0.034320
C	-6.207543	1.746149	0.098986
C	-6.221879	3.182589	0.088277
C	-4.951401	3.852635	0.018896
C	-5.665662	-2.898232	0.131691
C	-3.332067	-3.506052	-0.031350
C	-6.010940	-4.239377	0.158477
C	-3.689565	-4.846541	-0.004479
C	-5.025860	-5.222530	0.090377
H	-7.142058	1.200428	0.151352
H	-4.934127	4.936986	0.010636
H	-6.444976	-2.149456	0.185662
H	-2.290842	-3.231673	-0.106129
H	-7.055801	-4.520246	0.232900
H	-2.914396	-5.603183	-0.058980
H	-5.298821	-6.271967	0.111110
H	-1.873785	3.188660	-0.124820
Cu	-0.000017	0.000056	-0.135116
O	1.413203	-1.436148	-0.130543
C	2.595837	-0.947196	-0.074747
C	3.760984	-1.722997	-0.025148
C	2.707103	0.508998	-0.059595
C	5.016029	-1.070533	0.044118
C	3.774033	-3.159000	-0.034365
O	1.603715	1.171272	-0.111221
C	3.982482	1.086460	0.009119
O	5.069362	0.284646	0.058299
C	6.207427	-1.746229	0.099115
O	2.605298	-3.834374	-0.096352
C	4.951206	-3.852668	0.018874
C	4.316853	2.501862	0.036518
C	6.221704	-3.182671	0.088346
H	7.141964	-1.200547	0.151518
H	1.873623	-3.188546	-0.125104
H	4.933891	-4.937017	0.010530
C	5.665816	2.898085	0.131609
C	3.332272	3.506140	-0.031284
O	7.310242	-3.827887	0.139167

C	6.011232	4.239194	0.158373
H	6.445057	2.149227	0.185524
C	3.689908	4.846594	-0.004424
H	2.291018	3.231866	-0.105999
C	5.026247	5.222447	0.090342
H	7.056127	4.519959	0.232723
H	2.914813	5.603316	-0.058867
H	5.299317	6.271856	0.111060

SCF Energy: -2102.00856497
Sum of electronic and zero-point Energies= -2101.612258
Sum of electronic and thermal Energies= -2101.579217
Sum of electronic and thermal Enthalpies= -2101.578273
Sum of electronic and thermal Free Energies= -2101.679915

==> ./AOX-II/PA/PA/bis-/c3c4/vEA <==

O	-5.075572	-0.289137	0.029704
O	-1.573044	-1.178431	-0.058125
O	-2.619857	3.829456	-0.050909
O	-1.426012	1.439000	-0.064598
O	-7.336879	3.825625	0.066276
C	-3.775081	1.713666	-0.013260
C	-5.030208	1.059404	0.021591
C	-3.961666	-1.088712	0.004036
C	-2.604846	0.933800	-0.037702
C	-2.706112	-0.518202	-0.030939
C	-4.304573	-2.510251	0.019856
C	-3.791169	3.144211	-0.019152
C	-6.228127	1.739774	0.048128
C	-6.245248	3.164842	0.041558
C	-4.972325	3.835517	0.006410
C	-5.651965	-2.911635	0.080420
C	-3.321364	-3.515073	-0.025202
C	-5.997580	-4.255319	0.095673
C	-3.677157	-4.858071	-0.010382
C	-5.013523	-5.239355	0.050088
H	-7.161682	1.189115	0.074563
H	-4.954778	4.920525	0.001089
H	-6.435353	-2.165700	0.116463
H	-2.278950	-3.236815	-0.072215
H	-7.044896	-4.534948	0.143297
H	-2.897909	-5.612359	-0.046935
H	-5.285840	-6.289331	0.061614
H	-1.889957	3.180907	-0.063675
Cu	-0.000064	-0.000386	-0.067206
O	1.426474	-1.439759	-0.065026
C	2.605153	-0.934210	-0.037896
C	3.775613	-1.713724	-0.013313
C	2.705965	0.517841	-0.031046
C	5.030557	-1.059078	0.021701
C	3.792134	-3.144268	-0.019314
O	1.572764	1.177781	-0.058402
C	3.961349	1.088722	0.004078
O	5.075523	0.289444	0.029889
C	6.228662	-1.739128	0.048309
O	2.621026	-3.829860	-0.051220
C	4.973482	-3.835237	0.006326
C	4.303862	2.510346	0.019856
C	6.246199	-3.164189	0.041621
H	7.162068	-1.188218	0.074838
H	1.890950	-3.181495	-0.064047
H	4.956247	-4.920249	0.000925
C	5.651115	2.912139	0.080777
C	3.320374	3.514884	-0.025626
O	7.338018	-3.824661	0.066352
C	5.996332	4.255928	0.095964
H	6.434718	2.166447	0.117153
C	3.675770	4.857987	-0.010876
H	2.278053	3.236322	-0.072924
C	5.012008	5.239673	0.049947
H	7.043555	4.535860	0.143876
H	2.896312	5.612042	-0.047776
H	5.284012	6.289730	0.061409

SCF Energy: -2102.30035250

==> ./AOX-II/PA/PA/bis-/c3c4/vIP <==

O	-5.075572	-0.289137	0.029704
O	-1.573044	-1.178431	-0.058125
O	-2.619857	3.829456	-0.050909
O	-1.426012	1.439000	-0.064598
O	-7.336879	3.825625	0.066276
C	-3.775081	1.713666	-0.013260
C	-5.030208	1.059404	0.021591
C	-3.961666	-1.088712	0.004036
C	-2.604846	0.933800	-0.037702
C	-2.706112	-0.518202	-0.030939
C	-4.304573	-2.510251	0.019856
C	-3.791169	3.144211	-0.019152
C	-6.228127	1.739774	0.048128
C	-6.245248	3.164842	0.041558
C	-4.972325	3.835517	0.006410
C	-5.651965	-2.911635	0.080420
C	-3.321364	-3.515073	-0.025202
C	-5.997580	-4.255319	0.095673
C	-3.677157	-4.858071	-0.010382
C	-5.013523	-5.239355	0.050088
H	-7.161682	1.189115	0.074563
H	-4.954778	4.920525	0.001089
H	-6.435353	-2.165700	0.116463
H	-2.278950	-3.236815	-0.072215

H	-7.044896	-4.534948	0.143297
H	-2.897909	-5.612359	-0.046935
H	-5.285840	-6.289331	0.061614
H	-1.889957	3.180907	-0.063675
Cu	-0.000064	-0.000386	-0.067206
O	1.426474	-1.439759	-0.065026
C	2.605153	-0.934210	-0.037896
C	3.775613	-1.713724	-0.013313
C	2.705965	0.517841	-0.031046
C	5.030557	-1.059078	0.021701
C	3.792134	-3.144268	-0.019314
O	1.572764	1.177781	-0.058402
C	3.961349	1.088722	0.004078
O	5.075523	0.289444	0.029889
C	6.228662	-1.739128	0.048309
O	2.621026	-3.829860	-0.051220
C	4.973482	-3.835237	0.006326
C	4.303862	2.510346	0.019856
C	6.246199	-3.164189	0.041621
H	7.162068	-1.188218	0.074838
H	1.890950	-3.181495	-0.064047
H	4.956247	-4.920249	0.000925
C	5.651115	2.912139	0.080777
C	3.320374	3.514884	-0.025626
O	7.338018	-3.824661	0.066352
C	5.996332	4.255928	0.095964
H	6.434718	2.166447	0.117153
C	3.675770	4.857987	-0.010876
H	2.278053	3.236322	-0.072924
C	5.012008	5.239673	0.049947
H	7.043555	4.535860	0.143876
H	2.896312	5.612042	-0.047776
H	5.284012	6.289730	0.061409

SCF Energy: -2102.00545786

==> ./AOX-II/PA/PA/bis-/c3c4/RAF/c4 <==

O	4.546796	0.936009	-0.500982
O	2.001380	1.120578	1.784871
O	4.357954	-3.409055	1.606952
O	2.135569	-1.138719	-0.380007
O	8.382974	-1.783814	-0.243639
C	4.333146	-1.209462	0.608703
C	5.123515	-0.274721	-0.053638
C	3.389167	1.352570	-0.132189
C	2.858171	-0.929661	0.764059
C	2.672768	0.560302	0.952290
C	2.908158	2.568054	-0.703511
C	5.004863	-2.393002	0.971073
C	6.454157	-0.409745	-0.365064
C	7.138362	-1.609001	0.013211
C	6.351729	-2.580481	0.689402
C	3.765478	3.356436	-1.502693
C	1.567687	2.965441	-0.514317
C	3.293328	4.517461	-2.077729
C	1.104903	4.124448	-1.109203
C	1.964227	4.901112	-1.883000
H	6.977541	0.385306	-0.882919
H	6.819245	-3.509618	1.000205
H	4.794845	3.055033	-1.648145
H	0.892732	2.358502	0.071333
H	3.952890	5.129664	-2.680717
H	0.072278	4.422962	-0.971957
H	1.597348	5.812259	-2.342128
H	3.461985	-3.103821	1.831053
Cu	0.203303	-1.185942	-0.360780
O	-1.146927	0.949921	0.248586
C	-2.354468	0.589661	0.183256
C	-3.433411	1.474739	0.456904
C	-2.672867	-0.804325	-0.192636
C	-4.764589	1.013600	0.385282
C	-3.250706	2.847109	0.814431
O	-1.692784	-1.616172	-0.455669
C	-4.006662	-1.174597	-0.241063
O	-5.003979	-0.269109	0.050185
C	-5.856759	1.819230	0.646133
O	-1.993460	3.352202	0.892681
C	-4.321005	3.662946	1.077836
C	-4.562841	-2.489041	-0.565482
C	-5.673860	3.183675	1.007160
H	-6.858298	1.408685	0.576544
H	-1.379914	2.615241	0.676851
H	-4.146654	4.699921	1.347126
C	-5.947457	-2.719408	-0.440668
C	-3.765424	-3.561056	-1.009778
O	-6.662172	3.957633	1.259802
C	-6.501532	-3.956276	-0.738260
H	-6.596479	-1.921624	-0.103666
C	-4.329729	-4.795676	-1.308242
H	-2.701370	-3.418120	-1.121769
C	-5.698029	-5.006681	-1.174237
H	-7.571606	-4.099649	-0.627382
H	-3.687201	-5.600805	-1.650266
H	-6.132888	-5.973090	-1.406411
O	2.381124	-1.645864	1.887636
H	1.416037	-1.639329	1.843367

SCF Energy: -2177.93875907

Sum of electronic and zero-point Energies= -2177.529444

Sum of electronic and thermal Energies= -2177.494821

Sum of electronic and thermal Enthalpies= -2177.493877

Sum of electronic and thermal Free Energies= -2177.599104

==> ./AOX-II/PA/PA/bis-/c3c4/RAF/c6p <==

O	5.227628	-0.654580	0.090182
O	1.665873	-0.604202	0.239959
O	3.936724	3.974548	-0.144168
O	2.154350	2.032353	-0.089300
O	8.468716	2.719597	0.071898
C	4.481105	1.635809	-0.015548
C	5.520759	0.670413	0.059455
C	3.948694	-1.145970	0.085088
C	3.126925	1.248984	-0.015110
C	2.844322	-0.222544	0.121306
C	3.846115	-2.520415	-0.056544
C	4.884180	3.015637	-0.068741
C	6.845154	1.010092	0.091018
C	7.246240	2.388073	0.043736
C	6.203763	3.373643	-0.040040
C	5.025252	-3.301503	-0.263247
C	2.500568	-3.226261	-0.039244
C	4.953454	-4.627517	-0.552679
C	2.552036	-4.675180	-0.389031
C	3.696054	-5.323625	-0.649534
H	7.602301	0.236533	0.145742
H	6.476231	4.422724	-0.078916
H	5.991607	-2.820729	-0.197709
H	5.870297	-5.184317	-0.712746
H	1.593412	-5.182037	-0.411866
H	3.692964	-6.376358	-0.906981
H	3.062144	3.529658	-0.147106
Cu	-0.081918	0.326572	0.115913
O	-1.848984	-1.429036	0.113776
C	-2.951324	-0.813399	0.067011
C	-4.203210	-1.485316	0.036295
C	-2.948254	0.664799	0.041170
C	-5.402959	-0.744810	-0.015482
C	-4.331262	-2.909390	0.058611
O	-1.803765	1.280034	0.067401
C	-4.170621	1.314081	-0.016316
O	-5.349788	0.601207	-0.041330
C	-6.652236	-1.334697	-0.040989
O	-3.212109	-3.676702	0.104491
C	-5.561473	-3.514476	0.033855
C	-4.420477	2.755023	-0.067583
C	-6.778625	-2.752076	-0.015559
H	-7.541013	-0.714197	-0.079722
H	-2.447466	-3.059572	0.117162
H	-5.622934	-4.598009	0.052605
C	-5.738325	3.239852	-0.186152
C	-3.383576	3.705485	-0.003181
O	-7.919979	-3.331651	-0.035643
C	-6.002539	4.600915	-0.240275
H	-6.564262	2.542395	-0.238138
C	-3.658181	5.066856	-0.056521
H	-2.362634	3.366998	0.088864
C	-4.965161	5.527670	-0.175965
H	-7.029463	4.939428	-0.333635
H	-2.836069	5.773557	-0.003638
H	-5.173572	6.591476	-0.218261
H	1.874745	-2.742720	-0.806410
O	1.840655	-3.122672	1.215539
H	1.476359	-2.227482	1.248005

SCF Energy: -2177.96296140
Sum of electronic and zero-point Energies= -2177.553547
Sum of electronic and thermal Energies= -2177.518127
Sum of electronic and thermal Enthalpies= -2177.517182
Sum of electronic and thermal Free Energies= -2177.625894

==> ./AOX-II/PA/PA/bis-/c3c4/RAF/c1p <==

O	-4.978109	0.201120	-0.937186
O	-1.452537	0.169189	-1.402885
O	-3.075560	1.927733	3.142649
O	-1.592337	1.338084	1.046752
O	-7.710111	1.281405	2.711251
C	-3.922955	1.009251	1.080179
C	-5.089233	0.612091	0.354350
C	-3.775976	-0.322562	-1.397447
C	-2.650409	0.925445	0.498729
C	-2.535889	0.263296	-0.858527
C	-3.745781	-1.842513	-1.694852
C	-4.134684	1.525898	2.409190
C	-6.344952	0.701218	0.878738
C	-6.551731	1.205065	2.210491
C	-5.387757	1.612223	2.947724
C	-4.983292	-2.591004	-1.488320
C	-3.761929	-0.817664	-2.830378
C	-6.085106	-2.374017	-2.236674
C	-4.961540	-0.759766	-3.662293
C	-6.074155	-1.453278	-3.346578
H	-7.200968	0.394675	0.288763
H	-5.513442	1.999093	3.952973
H	-4.955701	-3.363782	-0.727403
H	-2.808653	-0.608789	-3.304393
H	-6.979797	-2.959376	-2.053295
H	-4.907275	-0.174693	-4.573805
H	-6.951464	-1.390382	-3.981022
H	-2.269752	1.825833	2.597762
Cu	0.315571	1.103573	0.495643
O	1.526543	-0.907338	0.968238
C	2.733743	-0.711919	0.646312

C	3.730316	-1.716625	0.756738
C	3.125619	0.616416	0.131256
C	5.060639	-1.435079	0.378583
C	3.466324	-3.037624	1.236166
O	2.203521	1.525344	0.033638
C	4.452821	0.809781	-0.215811
O	5.373034	-0.208805	-0.082778
C	6.078419	-2.366540	0.457700
O	2.204699	-3.367875	1.613844
C	4.462025	-3.976100	1.321450
C	5.074310	2.022620	-0.747040
C	5.813922	-3.682331	0.931605
H	7.082853	-2.095040	0.151399
H	1.649676	-2.569166	1.476893
H	4.227459	-4.970385	1.688211
C	6.444024	2.030118	-1.078429
C	4.352281	3.214668	-0.949298
O	6.729942	-4.573238	1.008006
C	7.055776	3.167741	-1.585417
H	7.035856	1.134851	-0.938823
C	4.973891	4.349169	-1.457155
H	3.301033	3.242840	-0.704871
C	6.326924	4.338251	-1.779929
H	8.112663	3.137855	-1.830436
H	4.388901	5.252061	-1.600981
H	6.806883	5.226625	-2.176590
O	-2.568990	-2.498924	-1.353987
H	-2.324460	-3.076768	-2.084774

SCF Energy: -2177.95697040
Sum of electronic and zero-point Energies= -2177.549076
Sum of electronic and thermal Energies= -2177.514164
Sum of electronic and thermal Enthalpies= -2177.513220
Sum of electronic and thermal Free Energies= -2177.620437

==> ./AOX-II/PA/PA/bis-/c3c4/RAF/c3 <==

O	4.544880	-1.051175	-0.555579
O	1.979067	0.391813	-0.273259
O	5.667526	3.237048	1.349805
O	3.375346	1.966020	1.911166
O	8.608055	1.043720	-1.606208
C	5.007873	1.122767	0.431647
C	5.388317	0.057417	-0.408722
C	3.412617	-1.199267	0.053017
C	3.764278	1.105465	1.099084
C	2.780877	-0.017993	0.746281
C	2.785659	-2.487294	-0.135609
C	5.953462	2.185917	0.555114
C	6.555715	-0.004682	-1.098484
C	7.507988	1.077990	-0.981976
C	7.147850	2.164761	-0.125553
C	3.535394	-3.530849	-0.713329
C	1.456556	-2.731771	0.257112
C	2.971712	-4.782089	-0.883261
C	0.899049	-3.984795	0.069059
C	1.652888	-5.010906	-0.495244
H	6.788368	-0.862581	-1.717357
H	7.841171	2.990567	-0.008824
H	4.561429	-3.358452	-1.011080
H	0.860032	-1.941290	0.685923
H	3.558829	-5.581979	-1.318847
H	-0.129165	-4.162779	0.363447
H	1.212158	-5.992033	-0.633301
H	4.796480	3.075877	1.765176
Cu	0.056181	0.673975	-0.076669
O	-1.614934	-1.177119	0.239950
C	-2.750978	-0.639275	0.121551
C	-3.958571	-1.388246	0.130917
C	-2.836047	0.828137	-0.037614
C	-5.199880	-0.730792	0.000481
C	-3.998743	-2.811036	0.267705
O	-1.729799	1.509357	-0.058610
C	-4.094854	1.393464	-0.156776
O	-5.228730	0.609410	-0.131717
C	-6.409623	-1.398835	0.003892
O	-2.835886	-3.499494	0.393131
C	-5.188659	-3.492818	0.272563
C	-4.430857	2.807115	-0.326875
C	-6.448868	-2.814895	0.141244
H	-7.334084	-0.840956	-0.098752
H	-2.109698	-2.837352	0.366726
H	-5.183621	-4.573232	0.377217
C	-5.767388	3.194948	-0.547879
C	-3.460240	3.825676	-0.274044
O	-7.552845	-3.463921	0.148597
C	-6.112070	4.529189	-0.710529
H	-6.544367	2.443000	-0.595385
C	-3.815467	5.159655	-0.435127
H	-2.427312	3.561817	-0.103603
C	-5.139664	5.524488	-0.655438
H	-7.150987	4.792335	-0.881836
H	-3.043596	5.921183	-0.386370
H	-5.410966	6.567167	-0.782377
O	2.136945	-0.420189	1.927872
H	2.039131	0.381012	2.462225

SCF Energy: -2177.96941662
Sum of electronic and zero-point Energies= -2177.560130
Sum of electronic and thermal Energies= -2177.524536

Sum of electronic and thermal Enthalpies= -2177.523592
Sum of electronic and thermal Free Energies= -2177.632738

==> ./AOX-II/PA/PA/bis-/c3c4/RAF/c7 <==

O	5.047791	0.448372	-0.179621
O	1.549110	0.923205	-0.805309
O	2.860724	-3.122711	2.098206
O	1.553478	-1.345258	0.694834
O	6.800803	-2.411995	0.444811
C	3.907430	-1.328553	0.864487
C	5.056606	-0.666697	0.518534
C	3.898875	1.032548	-0.645045
C	2.647861	-0.801097	0.414063
C	2.664309	0.435129	-0.392843
C	4.179519	2.269102	-1.374484
C	4.006577	-2.495414	1.751655
C	6.389511	-1.124898	0.928775
C	6.438264	-2.295498	1.818996
C	5.187064	-2.927493	2.241535
C	5.492645	2.774712	-1.424393
C	3.174536	2.988076	-2.045624
C	5.783326	3.944868	-2.109976
C	3.476489	4.158157	-2.732563
C	4.777659	4.646585	-2.770102
H	7.175570	-0.375112	0.954730
H	5.229077	-3.753353	2.941016
H	6.293200	2.249174	-0.919566
H	2.158573	2.623725	-2.027242
H	6.804633	4.310827	-2.127990
H	2.681078	4.690915	-3.243341
H	5.007114	5.561528	-3.305762
H	2.117840	-2.636840	1.675402
Cu	-0.228611	0.095053	-0.666102
O	-1.928623	1.471532	0.709024
C	-3.037642	0.899527	0.519624
C	-4.249712	1.373900	1.092550
C	-3.091615	-0.309120	-0.332026
C	-5.464418	0.702667	0.840795
C	-4.319683	2.528507	1.932854
O	-1.988072	-0.749994	-0.855015
C	-4.327467	-0.903609	-0.532396
O	-5.465515	-0.390165	0.053588
C	-6.675572	1.113158	1.365818
O	-3.184060	3.218858	2.209851
C	-5.510924	2.952557	2.463524
C	-4.636473	-2.078759	-1.347440
C	-6.743760	2.262460	2.202227
H	-7.578547	0.557548	1.136971
H	-2.454015	2.761303	1.736140
H	-5.528159	3.833448	3.097536
C	-5.975824	-2.475042	-1.535884
C	-3.638597	-2.853575	-1.969362
O	-7.848498	2.673301	2.703550
C	-6.297830	-3.581995	-2.308043
H	-6.773576	-1.907136	-1.075089
C	-3.970712	-3.961895	-2.739479
H	-2.601876	-2.580568	-1.842656
C	-5.298533	-4.336041	-2.918008
H	-7.340344	-3.856159	-2.434483
H	-3.177225	-4.538448	-3.204300
H	-5.552002	-5.201028	-3.521742
O	7.463727	-2.488143	2.720213
H	8.230017	-1.965997	2.457656

SCF Energy: -2177.95961038
Sum of electronic and zero-point Energies= -2177.549794
Sum of electronic and thermal Energies= -2177.515441
Sum of electronic and thermal Enthalpies= -2177.514497
Sum of electronic and thermal Free Energies= -2177.621210

==> ./AOX-II/PA/PA/bis-/c3c4/RAF/c4a <==

O	5.111372	-0.091669	0.078041
O	1.752012	-1.435611	0.299254
O	2.413168	2.824397	-2.655799
O	1.433108	0.717258	-1.372861
O	6.247168	4.398797	-0.488230
C	3.775754	1.198802	-1.464221
C	4.893127	1.115557	-0.469491
C	4.071616	-0.969219	0.353480
C	2.543302	0.440871	-0.929008
C	2.766943	-0.697017	-0.031397
C	4.557266	-2.129573	1.093464
C	3.479293	2.631262	-1.876939
C	5.708099	2.136992	-0.176150
C	5.478401	3.463029	-0.747200
C	4.307721	3.651531	-1.580174
C	5.911883	-2.203968	1.475235
C	3.720510	-3.209952	1.434216
C	6.401802	-3.302583	2.165239
C	4.222219	-4.307157	2.122065
C	5.561418	-4.362982	2.494165
H	6.532668	2.007525	0.514441
H	4.098490	4.646862	-1.954038
H	6.585412	-1.393585	1.230208
H	2.678232	-3.180948	1.156033
H	7.448927	-3.329897	2.447818
H	3.555125	-5.126220	2.369738
H	5.946529	-5.221153	3.034469
H	1.797916	2.074286	-2.531900
Cu	-0.154915	-1.160356	-0.016312

O	-1.370809	0.943023	0.814752
C	-2.595322	0.715027	0.609036
C	-3.615728	1.644801	0.949484
C	-2.996003	-0.566521	-0.011589
C	-4.968939	1.338036	0.695148
C	-3.350850	2.910950	1.558408
O	-2.068488	-1.419385	-0.325458
C	-4.346469	-0.785835	-0.231289
O	-5.284879	0.160583	0.122550
C	-6.007526	2.194632	1.008094
O	-2.067568	3.262718	1.827478
C	-4.366821	3.775120	1.877053
C	-4.978100	-1.956186	-0.841408
C	-5.742869	3.453810	1.616163
H	-7.029718	1.904776	0.789960
H	-1.502710	2.515535	1.528669
H	-4.130332	4.728104	2.339765
C	-6.367493	-1.966913	-1.077374
C	-4.249663	-3.102922	-1.212473
O	-6.680530	4.270710	1.921712
C	-6.991501	-3.062825	-1.656045
H	-6.964986	-1.106065	-0.806827
C	-4.883880	-4.196834	-1.789287
H	-3.183777	-3.128809	-1.043049
C	-6.255923	-4.188517	-2.017951
H	-8.063081	-3.035368	-1.826088
H	-4.293462	-5.065751	-2.062287
H	-6.745368	-5.044337	-2.470641
O	4.110191	0.446267	-2.629158
H	4.911106	0.820157	-3.017020

SCF Energy: -2177.95387888
Sum of electronic and zero-point Energies= -2177.544759
Sum of electronic and thermal Energies= -2177.509034
Sum of electronic and thermal Enthalpies= -2177.508089
Sum of electronic and thermal Free Energies= -2177.619645

==> ./AOX-II/PA/PA/bis-/c3c4/RAF/c8 <==

O	-5.025559	0.734822	-0.012092
O	-1.438689	0.843372	-0.066893
O	-3.682783	-3.717738	-0.833536
O	-1.952944	-1.796934	-0.585463
O	-8.155481	-2.673870	0.054436
C	-4.275086	-1.440886	-0.434414
C	-5.272968	-0.523107	-0.247850
C	-3.752212	1.252321	0.078633
C	-2.906204	-1.006702	-0.397611
C	-2.649801	0.422214	-0.118634
C	-3.777189	2.679247	0.394228
C	-4.651080	-2.838663	-0.623142
C	-6.711006	-0.871505	-0.415781
C	-7.000639	-2.361250	-0.258048
C	-5.949898	-3.272765	-0.541879
C	-5.001892	3.319975	0.664509
C	-2.606582	3.457776	0.438376
C	-5.050067	4.672925	0.965789
C	-2.666137	4.813384	0.739474
C	-3.882777	5.431492	1.005480
H	-6.162772	-4.334218	-0.582232
H	-5.924093	2.753257	0.641446
H	-1.652375	2.996310	0.234408
H	-6.008606	5.137888	1.171588
H	-1.746990	5.389532	0.764189
H	-3.923032	6.489680	1.240519
H	-2.819069	-3.232719	-0.809055
Cu	0.263441	-0.126014	-0.078590
O	2.162328	1.563530	-0.470116
C	3.221348	0.900071	-0.289901
C	4.511971	1.495925	-0.319591
C	3.129276	-0.554821	-0.037330
C	5.663235	0.707459	-0.111937
C	4.725758	2.890157	-0.553122
O	1.953736	-1.106353	-0.012040
C	4.309294	-1.255463	0.154588
O	5.529003	-0.614054	0.111046
C	6.944561	1.225781	-0.125147
O	3.656677	3.700817	-0.761021
C	5.988513	3.424190	-0.570024
C	4.471684	-2.685668	0.418986
C	7.155717	2.614339	-0.354590
H	7.792828	0.570825	0.041965
H	2.857635	3.130398	-0.706941
H	6.115106	4.487224	-0.749315
C	5.758816	-3.226577	0.612128
C	3.379860	-3.572346	0.492467
O	8.327538	3.130506	-0.370477
C	5.941912	-4.578621	0.864780
H	6.625597	-2.580346	0.564575
C	3.573295	-4.925188	0.745824
H	2.379715	-3.192245	0.349197
C	4.851075	-5.441431	0.934219
H	6.947660	-4.960410	1.008621
H	2.710005	-5.581231	0.795443
H	4.996019	-6.498261	1.131820
H	-6.917798	-0.689878	-1.488535
O	-7.549295	-0.080397	0.380707
H	-8.342540	-0.614593	0.532148

SCF Energy: -2177.99720449
Sum of electronic and zero-point Energies= -2177.586871

Sum of electronic and thermal Energies= -2177.551351
Sum of electronic and thermal Enthalpies= -2177.550407
Sum of electronic and thermal Free Energies= -2177.660972

==> ./AOX-II/PA/PA/bis-/c3c4/RAF/c2 <==

O	5.119886	-0.876752	-0.133769
O	1.648394	-0.633643	0.224347
O	4.184204	3.852769	0.184958
O	2.268084	2.048621	0.357403
O	8.560248	2.278695	-0.510020
C	4.550765	1.479196	0.072432
C	5.512364	0.436458	-0.095459
C	3.934517	-1.276428	0.512016
C	3.181808	1.202623	0.242535
C	2.805637	-0.261801	0.276926
C	3.606027	-2.662840	-0.004163
C	5.049303	2.830060	0.032943
C	6.835502	0.688899	-0.285904
C	7.332633	2.043258	-0.328905
C	6.377280	3.099054	-0.155764
C	3.347513	-2.832811	-1.365691
C	3.567623	-3.765438	0.843328
C	3.057442	-4.092633	-1.871746
C	3.275519	-5.028597	0.334237
C	3.019990	-5.195046	-1.021131
H	7.531418	-0.131748	-0.415289
H	6.719512	4.127708	-0.179396
H	3.374590	-1.978454	-2.034608
H	3.772041	-3.654907	1.901840
H	2.859053	-4.214147	-2.931078
H	3.250645	-5.882060	1.003089
H	2.792309	-6.179424	-1.415815
H	3.284494	3.474703	0.283822
Cu	-0.135861	0.278496	0.172753
O	-1.911531	-1.435092	0.191122
C	-3.007768	-0.810470	0.118208
C	-4.264685	-1.472076	0.079849
C	-2.990662	0.666786	0.068799
C	-5.456725	-0.721997	-0.003985
C	-4.406263	-2.894235	0.129132
O	-1.840780	1.271619	0.109439
C	-4.205388	1.325151	-0.027969
O	-5.390540	0.622581	-0.058667
C	-6.711062	-1.300836	-0.033457
O	-3.294961	-3.670570	0.205017
C	-5.641647	-3.488337	0.101387
C	-4.440037	2.766316	-0.124195
C	-6.851024	-2.716125	0.020961
H	-7.593350	-0.673223	-0.097327
H	-2.524749	-3.060532	0.214673
H	-5.713427	-4.570643	0.141857
C	-5.745343	3.257579	-0.324969
C	-3.400044	3.710164	-0.024862
O	-7.997201	-3.285886	-0.000475
C	-5.994512	4.618889	-0.425449
H	-6.572891	2.564822	-0.406286
C	-3.659651	5.071905	-0.124525
H	-2.388558	3.366829	0.131667
C	-4.954129	5.539200	-0.326476
H	-7.011870	4.962496	-0.583069
H	-2.835822	5.773796	-0.042560
H	-5.150806	6.603168	-0.405403
O	4.200220	-1.220223	1.893520
H	3.429309	-1.516934	2.392416

SCF Energy: -2177.99841647
Sum of electronic and zero-point Energies= -2177.588187
Sum of electronic and thermal Energies= -2177.552809
Sum of electronic and thermal Enthalpies= -2177.551864
Sum of electronic and thermal Free Energies= -2177.661109

==> ./AOX-II/PA/PA/bis-/c3c4/RAF/c3p <==

O	5.135872	-0.151841	-0.005981
O	1.571194	-0.696774	0.097663
O	3.163564	4.212157	0.062362
O	1.710615	2.027336	0.102630
O	7.850098	3.679344	-0.057040
C	4.065254	1.981735	0.031753
C	5.238253	1.193783	-0.003377
C	3.923568	-0.804005	0.027243
C	2.783255	1.371381	0.069691
C	2.721397	-0.116430	0.068642
C	4.099575	-2.246445	-0.010310
C	4.250219	3.401709	0.030951
C	6.507223	1.733270	-0.034041
C	6.688885	3.146690	-0.032131
C	5.502668	3.957091	-0.000232
C	5.371212	-2.798669	-0.112779
C	3.020058	-3.158386	0.095324
C	5.563697	-4.223330	-0.278167
C	3.179427	-4.545380	0.046400
C	4.424999	-5.102574	-0.073378
H	7.371523	1.078723	-0.058587
H	5.606758	5.037369	0.001015
H	6.256868	-2.179321	-0.167665
H	2.026017	-2.752286	0.215896
H	5.401637	-4.153076	-1.413551
H	2.307054	-5.180012	0.136015
H	4.586524	-6.173670	-0.111079

H	2.373828	3.627611	0.083584
Cu	-0.195455	0.157025	0.091242
O	-2.068141	-1.713600	0.101055
C	-3.149346	-1.063660	0.059518
C	-4.421298	-1.700525	0.025169
C	-3.108229	0.415871	0.045234
C	-5.601467	-0.929558	-0.018819
C	-4.586550	-3.120940	0.038598
O	-1.954220	1.010263	0.076949
C	-4.315268	1.095810	-0.005506
O	-5.513560	0.414260	-0.034791
C	-6.866164	-1.486740	-0.045825
O	-3.487153	-3.916530	0.078705
C	-5.832178	-3.693943	0.012085
C	-4.529038	2.542991	-0.043776
C	-7.029237	-2.900231	-0.029312
H	-7.738472	-0.842873	-0.077842
H	-2.708532	-3.315531	0.095164
H	-5.921333	-4.775678	0.024455
C	-5.834579	3.061580	-0.159436
C	-3.469773	3.468066	0.031072
O	-8.185176	-3.451104	-0.049735
C	-6.065580	4.429117	-0.201261
H	-6.677311	2.385172	-0.218967
C	-3.711179	4.836139	-0.010234
H	-2.457463	3.104509	0.121926
C	-5.006245	5.330020	-0.127278
H	-7.083971	4.793109	-0.292777
H	-2.872092	5.521982	0.050347
H	-5.188859	6.398899	-0.160092
O	6.845287	-4.664631	0.010948
H	6.957627	-5.566311	-0.310866

SCF Energy: -2177.92807026
Sum of electronic and zero-point Energies= -2177.521163
Sum of electronic and thermal Energies= -2177.485609
Sum of electronic and thermal Enthalpies= -2177.484665
Sum of electronic and thermal Free Energies= -2177.592748

==> ./AOX-II/PA/PA/bis-/c3c4/RAF/c4p <==

O	-5.176528	0.054254	0.000859
O	-1.651331	0.614741	-0.207849
O	-3.071976	-4.220045	0.660351
O	-1.672765	-2.017114	0.265308
O	-7.758903	-3.788946	0.669786
C	-4.031507	-2.041203	0.318821
C	-5.227802	-1.282396	0.218180
C	-4.012010	0.767998	-0.140872
C	-2.774251	-1.426517	0.187531
C	-2.751666	0.061235	-0.068254
C	-4.192490	2.125579	-0.348719
C	-4.177931	-3.452906	0.554920
C	-6.470482	-1.842879	0.333188
C	-6.616012	-3.252523	0.564912
C	-5.411815	-4.029870	0.672308
C	-5.540429	2.667148	-0.391401
C	-3.100277	3.067876	-0.495082
C	-5.774784	3.965545	-0.633740
C	-3.331565	4.368070	-0.742063
C	-4.693672	4.934892	-0.946996
H	-7.355565	-1.223001	0.248987
H	-5.491008	-5.096969	0.849405
H	-6.371807	2.000257	-0.211071
H	-2.086943	2.715704	-0.387745
H	-6.790347	4.347431	-0.645696
H	-2.499627	5.059833	-0.833024
H	-4.786793	5.175185	-2.021170
H	-2.292460	-3.634667	0.552915
Cu	0.158321	-0.239857	-0.134114
O	1.876042	1.536099	0.299469
C	2.999656	0.967108	0.201749
C	4.227402	1.669947	0.334700
C	3.048303	-0.487648	-0.060554
C	5.453375	0.981780	0.219195
C	4.304104	3.074939	0.588526
O	1.925337	-1.129900	-0.173408
C	4.294564	-1.084237	-0.165256
O	5.448366	-0.343421	-0.021626
C	6.681214	1.604308	0.340871
O	3.157329	3.791943	0.708300
C	5.512048	3.711779	0.712643
C	4.598142	-2.493082	-0.418714
C	6.756455	3.002738	0.594464
H	7.592203	1.023894	0.242464
H	2.415826	3.157593	0.591144
H	5.534277	4.779763	0.905265
C	5.936459	-2.934244	-0.441613
C	3.595612	-3.454565	-0.650040
O	7.876569	3.611377	0.713126
C	6.252797	-4.263946	-0.680350
H	6.738108	-2.227930	-0.269549
C	3.922255	-4.784145	-0.889287
H	2.559851	-3.150453	-0.641714
C	5.248831	-5.202213	-0.905888
H	7.294379	-4.568796	-0.689296
H	3.125346	-5.499966	-1.064266
H	5.497722	-6.241662	-1.091629
O	-4.909745	6.126710	-0.196160
H	-4.360755	6.822632	-0.570031

SCF Energy: -2177.96408358

Sum of electronic and zero-point Energies= -2177.555528
Sum of electronic and thermal Energies= -2177.519477
Sum of electronic and thermal Enthalpies= -2177.518533
Sum of electronic and thermal Free Energies= -2177.630684

==> ./AOX-II/PA/PA/bis-/c3c4/RAF/c5 <==

O	-5.000489	0.647697	-0.023992
O	-1.630762	0.848057	1.072911
O	-3.079253	-3.289256	-1.680892
O	-1.693521	-1.713993	0.184874
O	-7.617887	-2.528796	-2.234962
C	-3.970371	-1.470906	-0.384031
C	-5.064752	-0.623353	-0.478083
C	-3.894520	1.165060	0.506202
C	-2.740572	-1.017497	0.127766
C	-2.705259	0.381545	0.602831
C	-4.046913	2.555375	0.905353
C	-4.148325	-2.896063	-0.851591
C	-6.267719	-0.976656	-1.066517
C	-6.497319	-2.271430	-1.667773
C	-5.420621	-3.150261	-1.593097
C	-5.062878	3.333781	0.320009
C	-3.222615	3.143241	1.880341
C	-5.234688	4.658139	0.686728
C	-3.410630	4.467438	2.248679
C	-4.409538	5.231007	1.652407
H	-7.058217	-0.234176	-1.105444
H	-5.512917	-4.144711	-2.021984
H	-5.704986	2.900003	-0.436554
H	-2.452469	2.557646	2.360256
H	-6.013613	5.247736	0.216271
H	-2.773746	4.903902	3.010007
H	-4.547205	6.267739	1.939526
H	-2.267158	-3.008315	-1.226496
Cu	0.129973	0.021102	0.625328
O	1.689445	1.511104	-0.228618
C	2.829115	0.958052	-0.230535
C	4.002542	1.621742	-0.670210
C	2.939394	-0.435061	0.245533
C	5.242628	0.947457	-0.646395
C	4.013500	2.968417	-1.151451
O	1.851518	-1.025452	0.637719
C	4.195169	-1.019647	0.245609
O	5.298219	-0.321666	-0.198959
C	6.423329	1.530645	-1.064672
O	2.850742	3.668228	-1.195118
C	5.174580	3.564771	-1.569630
C	4.557449	-2.367497	0.682343
C	6.432808	2.870090	-1.546383
H	7.347827	0.964877	-1.025373
H	2.146051	3.071298	-0.861694
H	5.148698	4.588339	-1.929813
C	5.902919	-2.784956	0.649308
C	3.602758	-3.291863	1.148284
O	7.507732	3.440213	-1.943612
C	6.271851	-4.057538	1.061378
H	6.667821	-2.104444	0.298361
C	3.981941	-4.564473	1.558375
H	2.562817	-3.003989	1.185084
C	5.315141	-4.959650	1.519837
H	7.317469	-4.345931	1.023648
H	3.221542	-5.253531	1.912008
H	5.605485	-5.954269	1.841372
O	-4.148227	-3.679251	0.342256
H	-4.407179	-4.578668	0.109755

SCF Energy: -2177.92358778
Sum of electronic and zero-point Energies= -2177.515325
Sum of electronic and thermal Energies= -2177.479568
Sum of electronic and thermal Enthalpies= -2177.478624
Sum of electronic and thermal Free Energies= -2177.588997

==> ./AOX-II/PA/PA/bis-/c3c4/RAF/c8a <==

O	4.958785	0.090680	-0.216531
O	1.619223	1.403962	-0.275693
O	2.382804	-3.051180	2.185720
O	1.303518	-0.947504	1.099548
O	7.034920	-3.429468	2.046958
C	3.603034	-1.424010	0.936076
C	4.816113	-1.020623	0.451666
C	3.919984	0.952701	-0.486216
C	2.440125	-0.617672	0.689769
C	2.629483	0.641529	-0.061092
C	4.382002	2.138127	-1.205713
C	3.560908	-2.646266	1.730922
C	6.058779	-1.851346	0.554915
C	5.983727	-2.931269	1.635325
C	4.688228	-3.355141	2.050466
C	5.761064	2.377714	-1.358422
C	3.488346	3.065064	-1.770794
C	6.221304	3.494541	-2.040243
C	3.959573	4.179280	-2.454984
C	5.324596	4.405028	-2.593694
H	6.088929	-2.437060	-0.380320
H	4.609438	-4.208107	2.713841
H	6.479836	1.686218	-0.936179
H	2.424826	2.905472	-1.675956
H	7.289938	3.655029	-2.138080

H 3.247064 4.876274 -2.883859
H 5.686716 5.277971 -3.126157
H 1.701536 -2.394859 1.893363
Cu -0.286929 1.097135 0.062880
O -1.566969 -0.912243 -0.931752
C -2.778710 -0.696703 -0.652008
C -3.821758 -1.586869 -1.028720
C -3.139706 0.526270 0.097805
C -5.158844 -1.300490 -0.682555
C -3.595747 -2.793127 -1.762157
O -2.194006 1.351653 0.430210
C -4.475775 0.725104 0.407463
O -5.437386 -0.180960 0.012392
C -6.217808 -2.121598 -1.021608
O -2.329482 -3.123365 -2.123626
C -4.632411 -3.621076 -2.109393
C -5.070025 1.838688 1.147575
C -5.991773 -3.321274 -1.752912
H -7.225892 -1.850735 -0.726878
H -1.745098 -2.409805 -1.783037
H -4.425491 -4.528939 -2.667227
C -6.467238 1.907754 1.319507
C -4.296226 2.867488 1.718582
O -6.948954 -4.105278 -2.083330
C -7.056025 2.949414 2.022015
H -7.099377 1.137117 0.897944
C -4.894890 3.905891 2.422175
H -3.222660 2.843185 1.608279
C -6.275859 3.958867 2.580229
H -8.135357 2.971239 2.133970
H -4.269120 4.681827 2.851687
H -6.738109 4.772301 3.129359
O 7.230192 -1.093173 0.695485
H 7.445783 -0.695022 -0.153179

SCF Energy: -2177.99604658
Sum of electronic and zero-point Energies= -2177.585358
Sum of electronic and thermal Energies= -2177.549884
Sum of electronic and thermal Enthalpies= -2177.548940
Sum of electronic and thermal Free Energies= -2177.658071

==> ./AOX-II/PA/PA/bis-/c3c4/RAF/c2p <==
O 5.098439 0.051223 -0.015581
O 1.690261 -1.008422 0.045567
O 2.446283 3.950111 -0.968802
O 1.349080 1.601712 -0.465918
O 7.149280 4.158493 -0.909474
C 3.684484 1.946475 -0.486126
C 4.970007 1.366453 -0.323947
C 4.044184 -0.809145 0.134706
C 2.518450 1.170865 -0.346244
C 2.697504 -0.294337 -0.040484
C 4.398829 -2.111104 0.425671
C 3.643392 3.348990 -0.802910
C 6.127178 2.082194 -0.460139
C 6.085618 3.482718 -0.778095
C 4.790268 4.081648 -0.941582
C 5.878351 -2.415996 0.571162
C 3.463797 -3.181767 0.589484
C 6.219032 -3.870832 0.614993
C 3.886414 -4.468201 0.705975
C 5.283065 -4.828289 0.679814
H 7.085942 1.593916 -0.329746
H 4.727086 5.137328 -1.182152
H 6.396662 -1.965814 -0.284574
H 2.407759 -2.964833 0.603805
H 7.278196 -4.108269 0.634110
H 3.149025 -5.255383 0.820043
H 5.558615 -5.875471 0.735412
H 1.749842 3.272456 -0.833293
Cu -0.243039 -0.520102 -0.109617
O -1.745942 0.014671 1.484852
C -2.915527 0.026406 0.995682
C -4.066194 0.318544 1.769184
C -3.080604 -0.278913 -0.438881
C -5.340466 0.311600 1.160558
C -4.023503 0.626248 3.165191
O -2.009086 -0.542809 -1.121503
C -4.365585 -0.264739 -0.956525
O -5.446529 0.027801 -0.151465
C -6.504989 0.584506 1.852056
O -2.826833 0.646516 3.806373
C -5.167767 0.900435 3.867954
C -4.782932 -0.534410 -2.331403
C -6.461501 0.891821 3.241217
H -7.457260 0.564913 1.333340
H -2.141061 0.426469 3.140103
H -5.100862 1.130055 4.926688
C -6.146847 -0.477280 -2.681533
C -3.865164 -0.858603 -3.349003
O -7.519944 1.150565 3.912468
C -6.567911 -0.729866 -3.979160
H -6.883636 -0.232117 -1.927572
C -4.296658 -1.110362 -4.645794
H -2.812478 -0.911270 -3.114947
C -5.647090 -1.048864 -4.973989
H -7.626238 -0.676693 -4.213834
H -3.563874 -1.357347 -5.407459
H -5.977855 -1.246303 -5.988128
O 6.341259 -1.782087 1.772710
H 7.301127 -1.728054 1.726274

SCF Energy: -2177.95865542
Sum of electronic and zero-point Energies= -2177.550509
Sum of electronic and thermal Energies= -2177.517007
Sum of electronic and thermal Enthalpies= -2177.516063
Sum of electronic and thermal Free Energies= -2177.618883

==> ./AOX-II/PA/PA/bis-/c3c4/RAF/c6 <==

O	-5.047743	0.740214	0.150256
O	-1.530945	0.814986	1.004554
O	-3.279006	-3.304335	-1.514517
O	-1.794945	-1.607865	-0.265763
O	-7.836823	-2.494053	-1.738987
C	-4.064529	-1.228948	-0.701867
C	-5.212311	-0.402393	-0.499516
C	-3.836066	1.196655	0.660042
C	-2.785449	-0.856320	-0.153233
C	-2.676885	0.444988	0.540237
C	-4.004612	2.497142	1.303298
C	-4.229605	-2.428834	-1.374111
C	-6.497228	-0.738746	-0.881844
C	-6.730564	-1.973990	-1.520510
C	-5.512361	-2.717325	-2.065635
C	-5.280169	3.092015	1.376248
C	-2.924026	3.202288	1.867231
C	-5.463067	4.325649	1.983461
C	-3.118028	4.437885	2.472343
C	-4.384189	5.009630	2.537620
H	-7.329951	-0.115715	-0.580182
H	-5.358888	-2.223646	-3.048638
H	-6.136991	2.583433	0.954415
H	-1.933721	2.775357	1.826092
H	-6.458818	4.754967	2.024149
H	-2.265075	4.957043	2.897236
H	-4.529682	5.974031	3.012476
H	-2.467634	-2.923281	-1.076785
Cu	0.231033	-0.005055	0.785918
O	1.826981	1.356250	-0.748642
C	2.958930	0.827318	-0.569130
C	4.144869	1.341526	-1.162398
C	3.070351	-0.371538	0.291495
C	5.386216	0.715273	-0.925624
C	4.161587	2.493077	-2.009878
O	1.991410	-0.852567	0.831789
C	4.329093	-0.921681	0.474271
O	5.437882	-0.373466	-0.134599
C	6.574248	1.166334	-1.469289
O	2.997551	3.139005	-2.276237
C	5.329453	2.957441	-2.558735
C	4.692271	-2.086307	1.282451
C	6.589818	2.313265	-2.311564
H	7.499609	0.644333	-1.250967
H	2.291145	2.655833	-1.792080
H	5.306172	3.834766	-3.197492
C	6.039798	-2.489493	1.372882
C	3.741384	-2.841957	1.995300
O	7.672200	2.760049	-2.830201
C	6.414036	-3.586556	2.135483
H	6.802563	-1.936301	0.840527
C	4.125978	-3.939403	2.756587
H	2.700144	-2.561036	1.947134
C	5.460919	-4.322218	2.835161
H	7.461174	-3.868016	2.182863
H	3.368000	-4.500370	3.294095
H	5.755214	-5.179642	3.431098
O	-5.747772	-4.083597	-2.236609
H	-6.712706	-4.170800	-2.268554

SCF Energy: -2177.98652875
Sum of electronic and zero-point Energies= -2177.576923
Sum of electronic and thermal Energies= -2177.541438
Sum of electronic and thermal Enthalpies= -2177.540494
Sum of electronic and thermal Free Energies= -2177.650830

==> ./AOX-II/PA/PA/bis-/c3c4/RAF/c5p <==

O	-5.202836	0.171871	-0.171625
O	-1.702179	0.646252	0.552693
O	-3.106055	-4.025778	-1.137940
O	-1.739142	-1.948383	-0.291639
O	-7.754046	-3.454910	-1.714321
C	-4.070695	-1.880769	-0.629104
C	-5.257326	-1.113977	-0.578485
C	-4.027335	0.778772	0.207414
C	-2.823515	-1.311844	-0.256978
C	-2.815826	0.105925	0.195426
C	-4.245090	2.161583	0.593618
C	-4.206478	-3.236196	-1.070082
C	-6.493140	-1.614476	-0.931145
C	-6.625046	-2.962694	-1.373854
C	-5.425710	-3.752351	-1.424163
C	-5.540568	2.731971	0.544186
C	-3.222268	2.979884	1.061902
C	-5.817331	4.051288	0.905030
C	-3.453908	4.380882	1.339044
C	-4.819148	4.871399	1.364575
H	-7.369176	-0.977751	-0.874604
H	-5.491621	-4.783823	-1.755168
H	-6.362885	2.116609	0.203603
H	-2.208523	2.613062	1.139917

H -6.835434 4.414315 0.846214
H -4.996168 5.899997 1.656583
H -2.343007 -3.477314 -0.851264
Cu 0.078371 -0.184593 0.520761
O 1.831984 1.554925 -0.356267
C 2.934963 0.945374 -0.286142
C 4.156620 1.525456 -0.727270
C 2.973512 -0.424929 0.272480
C 5.364344 0.804655 -0.622148
C 4.242252 2.834996 -1.294830
O 1.863596 -0.968082 0.671589
C 4.204310 -1.058177 0.347443
O 5.351659 -0.434200 -0.094471
C 6.582447 1.308442 -1.038369
O 3.113448 3.578578 -1.422516
C 5.440479 3.353202 -1.714892
C 4.497164 -2.392056 0.873017
C 6.665787 2.610583 -1.606003
H 7.478908 0.706957 -0.933019
H 2.376458 3.031937 -1.068481
H 5.468988 4.350308 -2.142942
C 5.826993 -2.857295 0.916971
C 3.493093 -3.257268 1.349243
O 7.776350 3.108064 -2.005587
C 6.134010 -4.116571 1.412048
H 6.629369 -2.224428 0.560823
C 3.810332 -4.517524 1.842073
H 2.463829 -2.932592 1.329198
C 5.128754 -4.959372 1.879394
H 7.169425 -4.441309 1.432167
H 3.012347 -5.159932 2.200764
H 5.370566 -5.943703 2.266114
H -3.298363 4.645155 0.230527
O -2.484850 4.977584 2.127742
H -2.590960 5.934832 2.093847

SCF Energy: -2177.92825001
Sum of electronic and zero-point Energies= -2177.522010
Sum of electronic and thermal Energies= -2177.486125
Sum of electronic and thermal Enthalpies= -2177.485181
Sum of electronic and thermal Free Energies= -2177.595670

==> ./AOX-II/PA/PA/bis-/c3c4 <==
O -5.075572 -0.289137 0.029704
O -1.573044 -1.178431 -0.058125
O -2.619857 3.829456 -0.050909
O -1.426012 1.439000 -0.064598
O -7.336879 3.825625 0.066276
C -3.775081 1.713666 -0.013260
C -5.030208 1.059404 0.021591
C -3.961666 -1.088712 0.004036
C -2.604846 0.933800 -0.037702
C -2.706112 -0.518202 -0.030939
C -4.304573 -2.510251 0.019856
C -3.791169 3.144211 -0.019152
C -6.228127 1.739774 0.048128
C -6.245248 3.164842 0.041558
C -4.972325 3.835517 0.006410
C -5.651965 -2.911635 0.080420
C -3.321364 -3.515073 -0.025202
C -5.997580 -4.255319 0.095673
C -3.677157 -4.858071 -0.010382
C -5.013523 -5.239355 0.050088
H -7.161682 1.189115 0.074563
H -4.954778 4.920525 0.001089
H -6.435353 -2.165700 0.116463
H -2.278950 -3.236815 -0.072215
H -7.044896 -4.534948 0.143297
H -2.897909 -5.612359 -0.046935
H -5.285840 -6.289331 0.061614
H -1.889957 3.180907 -0.063675
Cu -0.000064 -0.000386 -0.067206
O 1.426474 -1.439759 -0.065026
C 2.605153 -0.934210 -0.037896
C 3.775613 -1.713724 -0.013313
C 2.705965 0.517841 -0.031046
C 5.030557 -1.059078 0.021701
C 3.792134 -3.144268 -0.019314
O 1.572764 1.177781 -0.058402
C 3.961349 1.088722 0.004078
O 5.075523 0.289444 0.029889
C 6.228662 -1.739128 0.048309
O 2.621026 -3.829860 -0.051220
C 4.973482 -3.835237 0.006326
C 4.303862 2.510346 0.019856
C 6.246199 -3.164189 0.041621
H 7.162068 -1.188218 0.074838
H 1.890950 -3.181495 -0.064047
H 4.956247 -4.920249 0.000925
C 5.651115 2.912139 0.080777
C 3.320374 3.514884 -0.025626
O 7.338018 -3.824661 0.066352
C 5.996332 4.255928 0.095964
H 6.434718 2.166447 0.117153
C 3.675770 4.857987 -0.010876
H 2.278053 3.236322 -0.072924
C 5.012008 5.239673 0.049947
H 7.043555 4.535860 0.143876
H 2.896312 5.612042 -0.047776
H 5.284012 6.289730 0.061409

SCF Energy: -2102.18827462

Sum of electronic and zero-point Energies= -2101.793626
Sum of electronic and thermal Energies= -2101.760430
Sum of electronic and thermal Enthalpies= -2101.759486
Sum of electronic and thermal Free Energies= -2101.863039

==> ./AOX-II/PA/PA <==

O	-0.207898	-0.828653	0.000102
O	-1.236212	2.648398	-0.000293
O	3.817396	1.750466	0.000016
O	1.453055	2.869264	-0.000174
O	3.971317	-2.973300	0.000081
C	1.744150	0.533383	0.000069
C	1.132376	-0.737880	0.000077
C	-1.045902	0.275107	0.000013
C	0.947074	1.712149	-0.000050
C	-0.535617	1.571800	-0.000111
C	-2.447672	-0.133122	0.000008
C	3.174281	0.554410	0.000042
C	1.853682	-1.918889	0.000082
C	3.275619	-1.896192	0.000052
C	3.906874	-0.605306	0.000041
C	-2.792550	-1.501312	-0.000382
C	-3.507205	0.796746	0.000414
C	-4.117847	-1.912402	-0.000380
C	-4.831399	0.374880	0.000427
C	-5.151888	-0.979189	0.000029
H	1.330010	-2.868897	0.000116
H	4.991113	-0.551185	0.000026
H	-2.011318	-2.250565	-0.000693
H	-3.277972	1.851959	0.000719
H	-4.343617	-2.974255	-0.000697
H	-5.622093	1.118849	0.000769
H	-6.187389	-1.302858	0.000019
H	3.116153	2.442450	-0.000094

SCF Energy: -952.465655913
Sum of electronic and zero-point Energies= -952.270905
Sum of electronic and thermal Energies= -952.255800
Sum of electronic and thermal Enthalpies= -952.254856
Sum of electronic and thermal Free Energies= -952.314762

==> ./AOX-II/PA/PA/vIP <==

O	-0.207898	-0.828653	0.000102
O	-1.236212	2.648398	-0.000293
O	3.817396	1.750466	0.000016
O	1.453055	2.869264	-0.000174
O	3.971317	-2.973300	0.000081
C	1.744150	0.533383	0.000069
C	1.132376	-0.737880	0.000077
C	-1.045902	0.275107	0.000013
C	0.947074	1.712149	-0.000050
C	-0.535617	1.571800	-0.000111
C	-2.447672	-0.133122	0.000008
C	3.174281	0.554410	0.000042
C	1.853682	-1.918889	0.000082
C	3.275619	-1.896192	0.000052
C	3.906874	-0.605306	0.000041
C	-2.792550	-1.501312	-0.000382
C	-3.507205	0.796746	0.000414
C	-4.117847	-1.912402	-0.000380
C	-4.831399	0.374880	0.000427
C	-5.151888	-0.979189	0.000029
H	1.330010	-2.868897	0.000116
H	4.991113	-0.551185	0.000026
H	-2.011318	-2.250565	-0.000693
H	-3.277972	1.851959	0.000719
H	-4.343617	-2.974255	-0.000697
H	-5.622093	1.118849	0.000769
H	-6.187389	-1.302858	0.000019
H	3.116153	2.442450	-0.000094

SCF Energy: -952.304713221

==> ./AOX-II/PA/PA/mono-/c3c4/aIP <==

O	-0.675057	-2.004022	0.005663
O	1.601695	0.730132	-0.011780
O	-3.333753	2.003337	-0.007896
O	-0.659752	2.047049	0.000638
O	-5.342901	-2.242794	-0.005215
C	-1.925797	0.050041	-0.001591
C	-1.877859	-1.366001	0.000801
C	0.508821	-1.387235	0.003466
C	-0.732530	0.769285	-0.000365
C	0.539421	0.038420	-0.003288
C	1.639094	-2.293132	0.006384
C	-3.235300	0.663584	-0.005258
C	-2.994594	-2.149371	0.000016
C	-4.309081	-1.533641	-0.004114
C	-4.367709	-0.096920	-0.006805
C	1.405491	-3.683987	-0.015529
C	2.970732	-1.834522	0.033305
C	2.462671	-4.575993	-0.011756
C	4.021394	-2.738960	0.037237
C	3.775863	-4.108685	0.014638
H	-2.912488	-3.228931	0.002423
H	-5.338815	0.384147	-0.009934
H	0.391242	-4.059897	-0.036712

H	3.177788	-0.775748	0.052295
H	2.263699	-5.641500	-0.029516
H	5.040506	-2.369988	0.058869
H	4.602682	-4.810337	0.017718
H	-2.437938	2.390845	-0.005035
O	3.223012	3.251990	-0.194227
H	3.427743	3.968713	0.417504
O	0.674034	4.724499	0.190075
H	1.257743	5.280374	-0.338615
H	3.775273	2.513879	0.088938
H	-0.200890	4.835799	-0.199361
Cu	1.233501	2.721166	-0.004120

SCF Energy: -1302.38777635
Sum of electronic and zero-point Energies= -1302.139108
Sum of electronic and thermal Energies= -1302.116991
Sum of electronic and thermal Enthalpies= -1302.116047
Sum of electronic and thermal Free Energies= -1302.190226

==> ./AOX-II/PA/PA/mono-/c3c4/HAT/c5 <==

O	1.434850	1.560072	0.066899
O	-1.559117	-0.125525	0.601253
O	2.577457	-3.100369	-0.306425
O	-0.117407	-2.191573	-0.226125
O	5.894412	0.236232	0.006794
C	1.859575	-0.837131	-0.094425
C	2.319265	0.494691	0.026231
C	0.142976	1.425585	0.099370
C	0.475017	-1.097299	-0.086263
C	-0.404345	0.074410	0.217481
C	-0.622052	2.640499	0.048402
C	2.903423	-1.887237	-0.199553
C	3.627201	0.867817	0.049393
C	4.680401	-0.138288	-0.035243
C	4.259219	-1.472294	-0.166486
C	0.031055	3.872744	0.263742
C	-2.001640	2.632277	-0.243048
C	-0.682338	5.053947	0.217048
C	-2.700078	3.825064	-0.303721
C	-2.048344	5.033072	-0.066572
H	3.891900	1.916310	0.117591
H	5.015229	-2.249209	-0.232234
H	1.089551	3.888038	0.489405
H	-2.513851	1.701948	-0.442864
H	-0.178714	5.995285	0.402206
H	-3.757571	3.814906	-0.539179
H	-2.604760	5.962789	-0.105883
O	-4.278137	-1.507649	-0.796620
H	-4.971388	-1.928171	-0.278389
O	-2.744673	-4.111992	0.674538
H	-2.527047	-4.693583	-0.060599
H	-4.419162	-0.564257	-0.668644
H	-2.101678	-4.339625	1.353233
Cu	-2.331642	-2.017418	0.049817

SCF Energy: -1301.90276560
Sum of electronic and zero-point Energies= -1301.669894
Sum of electronic and thermal Energies= -1301.646012
Sum of electronic and thermal Enthalpies= -1301.645067
Sum of electronic and thermal Free Energies= -1301.724277

==> ./AOX-II/PA/PA/mono-/c3c4/HAT/h2o <==

O	0.905176	-1.875722	-0.074283
O	-1.459341	0.584338	-0.843823
O	3.208929	2.353580	0.116117
O	0.505748	2.161957	-0.042788
O	5.578038	-1.712013	0.149174
C	1.981737	0.296596	-0.023132
C	2.062039	-1.116177	-0.046801
C	-0.303611	-1.369711	-0.156636
C	0.733036	0.925689	-0.115655
C	-0.431049	0.050540	-0.381800
C	-1.383094	-2.311517	-0.051327
C	3.224719	1.008971	0.083802
C	3.229167	-1.808250	0.017973
C	4.486497	-1.088116	0.102786
C	4.419833	0.343469	0.140239
C	-1.105175	-3.693210	-0.118797
C	-2.708759	-1.884345	0.172867
C	-2.125807	-4.613912	0.012977
C	-3.717962	-2.818588	0.320771
C	-3.433049	-4.179606	0.234863
H	3.231112	-2.891148	0.025129
H	5.343339	0.906614	0.211583
H	-0.090981	-4.029936	-0.290302
H	-2.939501	-0.831136	0.252162
H	-1.908038	-5.673199	-0.054152
H	-4.732387	-2.486150	0.505973
H	-4.230887	-4.905613	0.344443
H	2.283883	2.662558	0.070867
O	-3.603953	2.405457	0.320777
O	-1.415231	4.637352	0.175524
H	-2.065609	4.941042	0.816793
H	-3.878253	1.487986	0.256609
H	-0.566736	4.758885	0.613842
Cu	-1.716276	2.454122	-0.079701

SCF Energy: -1301.89119870

Sum of electronic and zero-point Energies= -1301.656907
Sum of electronic and thermal Energies= -1301.634109
Sum of electronic and thermal Enthalpies= -1301.633165
Sum of electronic and thermal Free Energies= -1301.709818

==> ./AOX-II/PA/PA/mono-/c3c4/aEA <==
O 0.877660 1.826927 -0.103341
O -1.532811 -0.864496 -0.259919
O 3.429868 -2.223124 -0.027745
O 0.800363 -2.225451 -0.175461
O 5.567282 1.979328 0.199364
C 2.068912 -0.238592 -0.074046
C 2.057064 1.172549 -0.041507
C -0.332067 1.178597 -0.193054
C 0.848571 -0.961840 -0.157169
C -0.409544 -0.198626 -0.214892
C -1.447982 2.129012 -0.209973
C 3.350838 -0.867546 -0.006913
C 3.205352 1.935616 0.045728
C 4.481142 1.307538 0.112489
C 4.502319 -0.128967 0.080201
C -1.297137 3.401780 0.366018
C -2.682272 1.815181 -0.801593
C -2.344205 4.313877 0.367897
C -3.724272 2.734118 -0.801535
C -3.565929 3.985670 -0.213036
H 3.133686 3.017620 0.066200
H 5.459205 -0.638754 0.131114
H -0.354444 3.673413 0.825834
H -2.822361 0.850400 -1.269282
H -2.204332 5.286141 0.829033
H -4.666426 2.469659 -1.270643
H -4.383975 4.698216 -0.210417
H 2.509335 -2.560092 -0.083998
O -3.330927 -0.380908 1.782564
H -2.703429 -0.515260 1.042626
O -2.080136 -4.793102 0.108853
H -2.787123 -5.006502 -0.508586
H -3.630023 0.524865 1.670987
H -1.357958 -5.381585 -0.134070
Cu -1.527837 -2.842818 -0.097759

SCF Energy: -1302.72301036
Sum of electronic and zero-point Energies= -1302.477961
Sum of electronic and thermal Energies= -1302.454329
Sum of electronic and thermal Enthalpies= -1302.453385
Sum of electronic and thermal Free Energies= -1302.533932

==> ./AOX-II/PA/PA/mono-/c3c4/vIP <==
O -0.756832 -1.968698 -0.011553
O 1.664488 0.722727 -0.016076
O -3.259335 2.123355 -0.000620
O -0.590091 2.058606 0.009753
O -5.456421 -2.045697 -0.008235
C -1.924800 0.111352 -0.007473
C -1.932160 -1.304248 -0.011049
C 0.469196 -1.357759 -0.010517
C -0.688539 0.777293 -0.004597
C 0.548423 0.018902 -0.013785
C 1.563679 -2.327972 0.000079
C -3.196695 0.767499 -0.003688
C -3.092031 -2.047115 -0.011770
C -4.360096 -1.394657 -0.008513
C -4.357853 0.045071 -0.004601
C 1.281951 -3.706279 0.019734
C 2.912373 -1.931454 -0.005721
C 2.304104 -4.643925 0.034463
C 3.929556 -2.877964 0.008252
C 3.635612 -4.237386 0.028743
H -3.041834 -3.130012 -0.013166
H -5.309223 0.566880 -0.001950
H 0.254725 -4.046616 0.025142
H 3.159882 -0.880148 -0.021462
H 2.057230 -5.700334 0.050863
H 4.962397 -2.545698 0.003066
H 4.433602 -4.972056 0.040170
H -2.347509 2.469261 0.003204
O 3.346635 3.150135 -0.199821
H 3.568951 3.861005 0.411562
O 0.822959 4.680002 0.207873
H 1.435574 5.221718 -0.302174
H 3.887790 2.402489 0.078019
H -0.037565 4.821748 -0.203365
Cu 1.318912 2.648972 0.000765

SCF Energy: -1302.38115767

==> ./AOX-II/PA/PA/mono-/c3c4/vEA <==
O -0.756832 -1.968698 -0.011553
O 1.664488 0.722727 -0.016076
O -3.259335 2.123355 -0.000620
O -0.590091 2.058606 0.009753
O -5.456421 -2.045697 -0.008235
C -1.924800 0.111352 -0.007473
C -1.932160 -1.304248 -0.011049
C 0.469196 -1.357759 -0.010517
C -0.688539 0.777293 -0.004597

C	0.548423	0.018902	-0.013785
C	1.563679	-2.327972	0.000079
C	-3.196695	0.767499	-0.003688
C	-3.092031	-2.047115	-0.011770
C	-4.360096	-1.394657	-0.008513
C	-4.357853	0.045071	-0.004601
C	1.281951	-3.706279	0.019734
C	2.912373	-1.931454	-0.005721
C	2.304104	-4.643925	0.034463
C	3.929556	-2.877964	0.008252
C	3.635612	-4.237386	0.028743
H	-3.041834	-3.130012	-0.013166
H	-5.309223	0.566880	-0.001950
H	0.254725	-4.046616	0.025142
H	3.159882	-0.880148	-0.021462
H	2.057230	-5.700334	0.050863
H	4.962397	-2.545698	0.003066
H	4.433602	-4.972056	0.040170
H	-2.347509	2.469261	0.003204
O	3.346635	3.150135	-0.199821
H	3.568951	3.861005	0.411562
O	0.822959	4.680002	0.207873
H	1.435574	5.221718	-0.302174
H	3.887790	2.402489	0.078019
H	-0.037565	4.821748	-0.203365
Cu	1.318912	2.648972	0.000765

SCF Energy: -1302.69373344

==> ./AOX-II/PA/PA/mono-/c3c4/RAF/c5p <==

O	0.762260	-1.910299	-0.035931
O	-1.098292	1.179110	-0.156617
O	4.035730	1.589563	-0.047229
O	1.446541	2.088312	-0.152231
O	5.336294	-2.941294	0.170179
C	2.322275	-0.100012	-0.043939
C	2.044394	-1.485970	0.000103
C	-0.301429	-1.044439	-0.105333
C	1.264548	0.841820	-0.110504
C	-0.125341	0.325240	-0.127108
C	-1.568143	-1.761390	-0.136864
C	3.703554	0.275199	-0.008930
C	3.027280	-2.448894	0.073776
C	4.402139	-2.073301	0.107299
C	4.695740	-0.666914	0.064259
C	-1.604052	-3.165267	0.040836
C	-2.787958	-1.123187	-0.319840
C	-2.781462	-3.914628	0.034264
C	-4.017074	-1.879722	-0.438596
C	-3.994664	-3.297128	-0.126373
H	2.754435	-3.497965	0.102369
H	5.732737	-0.347984	0.088769
H	-0.669355	-3.690115	0.189265
H	-2.845051	-0.055862	-0.479435
H	-2.731145	-4.983967	0.193891
H	-4.931862	-3.841349	-0.120596
H	3.197619	2.097387	-0.094136
O	-2.728219	1.355783	2.129126
H	-2.165979	1.284779	1.335876
O	-0.955808	5.165440	-0.282697
H	-1.874987	5.418000	-0.148988
H	-3.323973	0.605424	2.062512
H	-0.481264	5.572866	0.449247
Cu	-0.745761	3.141698	-0.209065
H	-3.836711	-2.045510	-1.561207
O	-5.166581	-1.136795	-0.228375
H	-5.931649	-1.631933	-0.542767

SCF Energy: -1378.32310170

Sum of electronic and zero-point Energies= -1378.063591

Sum of electronic and thermal Energies= -1378.039412

Sum of electronic and thermal Enthalpies= -1378.038468

Sum of electronic and thermal Free Energies= -1378.117688

==> ./AOX-II/PA/PA/mono-/c3c4/RAF/c7 <==

O	1.260837	1.472828	-0.030801
O	-1.829546	-0.347502	0.003917
O	2.478496	-3.133903	0.157118
O	-0.018141	-2.339518	0.006853
O	4.328314	0.544953	-1.196756
C	1.794535	-0.823011	-0.012329
C	2.149132	0.501597	-0.043570
C	-0.090402	1.254418	0.000098
C	0.398718	-1.153857	-0.009696
C	-0.577356	-0.050987	-0.007186
C	-0.845309	2.506577	0.027963
C	2.852399	-1.836041	0.097967
C	3.547995	0.945268	-0.059926
C	4.573450	-0.105122	0.047392
C	4.157568	-1.504076	0.171796
C	-0.168162	3.740250	-0.006799
C	-2.249874	2.530850	0.092765
C	-0.864941	4.939200	0.020665
C	-2.939052	3.737422	0.120872
C	-2.256072	4.947978	0.085185
H	3.745718	1.952673	0.295455
H	4.917135	-2.263571	0.310413
H	0.913011	3.761981	-0.056818
H	-2.796198	1.599917	0.121287
H	-0.315297	5.874180	-0.008067
H	-4.022927	3.725823	0.171951

H	-2.798999	5.886756	0.106708
H	1.497917	-3.164374	0.110449
O	-5.300012	-0.339858	-0.045846
H	-4.587628	-0.989100	-0.009440
O	-3.630925	-3.877969	0.018823
H	-4.546283	-3.644742	-0.167478
H	-4.835580	0.499256	-0.111837
H	-3.382028	-4.481972	-0.688567
Cu	-2.464948	-2.223983	0.000828
O	5.810437	0.131095	0.607418
H	6.003185	1.074903	0.584943

SCF Energy: -1378.35158310
Sum of electronic and zero-point Energies= -1378.089086
Sum of electronic and thermal Energies= -1378.064384
Sum of electronic and thermal Enthalpies= -1378.063440
Sum of electronic and thermal Free Energies= -1378.144076

==> ./AOX-II/PA/PA/mono-/c3c4/RAF/c2p <==

O	2.034185	-0.003241	-0.041917
O	-1.393352	-1.008624	-0.077055
O	-0.585399	4.032728	-0.022434
O	-1.703400	1.635904	-0.011749
O	4.120450	4.179912	-0.100685
C	0.636183	1.962368	-0.037505
C	1.917294	1.348192	-0.053588
C	0.971749	-0.866526	-0.046069
C	-0.533204	1.184636	-0.029285
C	-0.370619	-0.313971	-0.053142
C	1.312298	-2.204277	-0.060847
C	0.607340	3.400897	-0.039733
C	3.079712	2.066679	-0.074809
C	3.050534	3.503829	-0.079378
C	1.760054	4.136316	-0.059374
C	2.789267	-2.551137	-0.030477
C	0.361749	-3.272117	-0.115220
C	3.104720	-3.986529	-0.304218
C	0.763934	-4.558099	-0.293432
C	2.153116	-4.922539	-0.426852
H	4.034504	1.554101	-0.089573
H	1.706606	5.219499	-0.061053
H	3.289876	-1.938935	-0.791216
H	-0.689519	-3.048355	-0.031237
H	4.159433	-4.236937	-0.361170
H	0.015217	-5.341580	-0.339243
H	2.410720	-5.960716	-0.604200
H	-1.287331	3.348094	-0.009289
O	-5.037519	-0.034270	-1.359506
H	-4.838072	-0.357212	-2.243239
O	-4.638445	-0.204157	1.808350
H	-4.539215	-0.996231	2.344569
H	-5.202073	0.905838	-1.481590
H	-5.544895	-0.244690	1.488621
Cu	-3.316293	-0.263031	-0.014568
O	3.300267	-2.187930	1.259408
H	4.258175	-2.123879	1.189378

SCF Energy: -1378.34431759
Sum of electronic and zero-point Energies= -1378.085479
Sum of electronic and thermal Energies= -1378.061286
Sum of electronic and thermal Enthalpies= -1378.060342
Sum of electronic and thermal Free Energies= -1378.141203

==> ./AOX-II/PA/PA/mono-/c3c4/RAF/c8a <==

O	-1.683372	-0.905488	-0.159640
O	1.889789	-0.608782	-0.187212
O	-0.867471	3.744653	-0.271754
O	1.085402	2.007921	-0.265241
O	-5.213313	2.127720	0.261039
C	-1.183953	1.375920	-0.240918
C	-2.074460	0.337476	-0.210972
C	-0.363543	-1.284891	-0.137377
C	0.221847	1.094198	-0.241380
C	0.637895	-0.318973	-0.195797
C	-0.222941	-2.734678	-0.019640
C	-1.723374	2.732220	-0.225077
C	-3.557256	0.518615	-0.322073
C	-4.024348	1.941305	-0.008436
C	-3.063853	2.988660	-0.126403
C	-1.335044	-3.523075	0.329947
C	1.002527	-3.384306	-0.244970
C	-1.222529	-4.900112	0.450807
C	1.103932	-4.765220	-0.126276
C	-0.002518	-5.531862	0.223334
H	-3.778788	0.414732	-1.398440
H	-3.402551	4.014100	-0.038958
H	-2.292862	-3.052498	0.516127
H	1.872169	-2.803652	-0.516068
H	-2.094520	-5.482783	0.728731
H	2.060289	-5.243585	-0.310113
H	0.083111	-6.608838	0.319749
H	0.048177	3.373767	-0.288963
O	5.195333	1.571850	-0.270890
H	5.071856	2.514470	-0.112833
O	2.795965	2.805066	1.893190
H	3.595167	2.280444	1.784668
H	5.745483	1.272547	0.460668
H	2.226140	2.492107	1.172277

Cu	3.402774	0.628041	-0.243986
O	-4.288637	-0.417350	0.421881
H	-4.277963	-1.260389	-0.040847

SCF Energy: -1378.38906700

==> ./AOX-II/PA/PA/mono-/c3c4/RAF/c3 <==

O	-1.212531	1.520278	-0.267054
O	0.714174	-0.711030	-0.812733
O	-3.497837	-2.542923	0.975417
O	-0.855517	-2.206699	1.316444
O	-5.834405	1.076321	-0.931492
C	-2.267311	-0.581649	0.351319
C	-2.360828	0.719767	-0.180661
C	-0.030560	1.147660	0.090108
C	-1.021413	-1.109450	0.750564
C	0.240439	-0.310002	0.392428
C	0.974075	2.182622	0.042938
C	-3.498298	-1.298510	0.457183
C	-3.508386	1.301880	-0.609610
C	-4.750908	0.563158	-0.530014
C	-4.684710	-0.751158	0.028938
C	0.558845	3.528290	0.030384
C	2.349041	1.884872	0.031612
C	1.496977	4.544726	0.026298
C	3.277532	2.910211	0.012088
C	2.855984	4.238151	0.016371
H	-3.506567	2.314067	-0.994798
H	-5.598577	-1.328482	0.116964
H	-0.496712	3.767096	0.050622
H	2.686319	0.859999	-0.000432
H	1.170699	5.577920	0.033043
H	4.335031	2.674092	-0.010097
H	3.588975	5.037266	0.009944
H	-2.583730	-2.759912	1.246319
O	4.055316	-0.965705	1.570950
H	4.460155	-0.117103	1.372124
O	3.652837	-3.357395	-1.107457
H	4.293144	-3.151132	-0.417623
H	3.109070	-0.802041	1.448999
H	3.372869	-4.259680	-0.921129
Cu	2.109673	-2.052104	-0.932971
O	1.137163	-0.420178	1.475873
H	0.932343	-1.265828	1.902933

SCF Energy: -1378.35891327

Sum of electronic and zero-point Energies= -1378.096984

Sum of electronic and thermal Energies= -1378.071928

Sum of electronic and thermal Enthalpies= -1378.070984

Sum of electronic and thermal Free Energies= -1378.153178

==> ./AOX-II/PA/PA/mono-/c3c4/RAF/c4p <==

O	0.906220	1.888939	-0.025146
O	0.146144	-1.611501	-0.022123
O	-3.890576	1.495868	0.080404
O	-2.306672	-0.620744	0.056906
O	-1.796247	5.707917	0.008350
C	-1.485994	1.589829	0.025370
C	-0.336948	2.427589	-0.003861
C	1.164811	0.539345	-0.023471
C	-1.348541	0.195747	0.029284
C	0.044199	-0.383364	-0.006176
C	2.506669	0.207867	-0.049274
C	-2.767808	2.245335	0.050628
C	-0.420606	3.792508	-0.010007
C	-1.699947	4.445814	0.013914
C	-2.868148	3.607677	0.044783
C	3.507008	1.262736	-0.069076
C	2.992608	-1.158741	-0.025809
C	4.817774	0.992668	-0.157869
C	4.304088	-1.432064	-0.120370
C	5.346973	-0.386577	-0.317393
H	0.482187	4.391743	-0.032019
H	-3.846795	4.074621	0.064260
H	3.176741	2.289926	-0.000585
H	2.282750	-1.963132	0.082940
H	5.546045	1.797127	-0.157917
H	4.648825	-2.461129	-0.088307
H	5.698202	-0.470822	-1.361098
H	-3.624527	0.553568	0.081548
O	-2.236916	-4.328135	1.547273
H	-2.229608	-5.167960	1.077731
O	-2.285946	-4.074782	-1.745600
H	-2.630332	-4.901247	-1.393709
H	-1.494012	-4.388186	2.155145
H	-3.014941	-3.714654	-2.259222
Cu	-1.878871	-2.743326	0.058231
O	6.464799	-0.560293	0.549587
H	6.949421	-1.339769	0.261306

SCF Energy: -1378.35097798

Sum of electronic and zero-point Energies= -1378.091121

Sum of electronic and thermal Energies= -1378.064870

Sum of electronic and thermal Enthalpies= -1378.063926

Sum of electronic and thermal Free Energies= -1378.149196

==> ./AOX-II/PA/PA/mono-/c3c4/RAF/c3p <==

O	2.104785	-0.106516	0.046042
O	-1.464692	-0.636543	-0.001772
O	0.134224	4.260221	-0.025480
O	-1.322765	2.067691	-0.032950
O	4.820754	3.723352	0.053001
C	1.035195	2.029265	0.011646
C	2.208363	1.239498	0.037022
C	0.892565	-0.757805	0.035430
C	-0.243901	1.417435	-0.003235
C	-0.306828	-0.067741	0.014456
C	1.065816	-2.201323	0.019303
C	1.220848	3.449251	0.000205
C	3.477358	1.778035	0.052614
C	3.659746	3.191678	0.041426
C	2.473613	4.003306	0.015157
C	2.337241	-2.758719	-0.047733
C	-0.020042	-3.106367	0.110767
C	2.526290	-4.185915	-0.195790
C	0.133678	-4.494454	0.080867
C	1.379338	-5.057682	-0.005947
H	4.341563	1.123256	0.071295
H	2.579233	5.083348	0.005641
H	3.226245	-2.143056	-0.089442
H	-1.014772	-2.694614	0.204458
H	2.386098	-4.125440	-1.334887
H	-0.742933	-5.124710	0.159076
H	1.536981	-6.129759	-0.028539
H	-0.656967	3.679203	-0.035646
O	-6.867284	-0.920776	0.281698
H	-7.306197	-1.208053	-0.524517
O	-5.031870	1.063871	-0.241899
H	-5.685697	0.362062	-0.040100
H	-6.434224	-1.713268	0.612401
H	-5.207392	1.766322	0.390903
Cu	-3.150119	0.387242	-0.061640
O	3.799226	-4.631756	0.121541
H	3.916783	-5.532420	-0.201679

SCF Energy: -1378.32220229
Sum of electronic and zero-point Energies= -1378.062418
Sum of electronic and thermal Energies= -1378.037443
Sum of electronic and thermal Enthalpies= -1378.036499
Sum of electronic and thermal Free Energies= -1378.119017

==> ./AOX-II/PA/PA/mono-/c3c4/RAF/c4 <==

O	-2.394409	0.295618	0.238630
O	-0.057371	-1.027127	-1.818099
O	1.271625	3.361575	-0.319453
O	1.651929	0.794556	-0.528901
O	-3.151079	4.947625	0.240841
C	-0.481856	1.757327	-0.019427
C	-1.853970	1.602271	0.150775
C	-1.738211	-0.731671	-0.165788
C	0.441055	0.558429	-0.013771
C	-0.371563	-0.484279	-0.788505
C	-2.331785	-2.016461	-0.015125
C	-0.043043	3.093697	-0.132121
C	-2.774140	2.611302	0.250413
C	-2.325488	3.973239	0.156902
C	-0.930068	4.157952	-0.043480
C	-3.651449	-2.122896	0.480201
C	-1.607477	-3.186068	-0.333395
C	-4.228146	-3.365043	0.635065
C	-2.196964	-4.424469	-0.164828
C	-3.502413	-4.514623	0.313332
H	-3.826238	2.386401	0.378969
H	-0.541246	5.167245	-0.135453
H	-4.207926	-1.227949	0.726735
H	-0.590241	-3.124341	-0.694283
H	-5.241818	-3.448974	1.007248
H	-1.640758	-5.322661	-0.403047
H	-3.960243	-5.489134	0.440777
H	1.716792	2.487495	-0.432082
O	4.584474	-1.806181	-0.215166
H	5.266667	-1.432399	0.383536
O	6.522843	-0.693701	1.380520
H	6.637976	0.236688	1.165980
H	4.300324	-2.626328	0.199386
H	6.301035	-0.697136	2.316175
Cu	3.066590	-0.522402	-0.371833
O	0.482770	0.057532	1.348245
H	1.141421	-0.650662	1.354804

SCF Energy: -1378.33155128
Sum of electronic and zero-point Energies= -1378.070900
Sum of electronic and thermal Energies= -1378.047376
Sum of electronic and thermal Enthalpies= -1378.046432
Sum of electronic and thermal Free Energies= -1378.126919

==> ./AOX-II/PA/PA/mono-/c3c4/RAF/c4a <==

O	-0.896295	-1.792298	0.287547
O	1.536757	0.808876	-0.371860
O	-3.431940	2.313615	0.326500
O	-0.806171	2.230726	-0.067854
O	-5.377532	-1.721960	-1.016469
C	-2.085787	0.296176	0.534595
C	-2.059889	-1.135977	0.101807
C	0.319651	-1.149790	0.161444

C	-0.802571	1.005987	0.054020
C	0.413142	0.208478	-0.073494
C	1.425452	-2.102847	0.254478
C	-3.366028	0.997486	0.105457
C	-3.119849	-1.791128	-0.381766
C	-4.391563	-1.100122	-0.601770
C	-4.435171	0.327689	-0.364923
C	1.206410	-3.464585	-0.011554
C	2.712669	-1.697414	0.643717
C	2.242334	-4.382957	0.090227
C	3.740448	-2.623129	0.755148
C	3.514082	-3.967948	0.473493
H	-3.048698	-2.838894	-0.647237
H	-5.351892	0.859196	-0.591344
H	0.221586	-3.802936	-0.308511
H	2.902143	-0.656266	0.865656
H	2.053718	-5.428157	-0.130280
H	4.725160	-2.291177	1.066521
H	4.322284	-4.686879	0.554745
H	-2.521717	2.671654	0.350705
O	3.060975	-0.165839	-2.444245
H	3.355415	-1.037748	-2.169966
O	2.651006	4.553207	0.251077
H	3.360149	4.758332	-0.367273
H	2.515731	0.144905	-1.692217
H	1.955205	5.186054	0.044587
Cu	1.995970	2.670519	-0.038444
O	-2.028602	0.271892	1.960537
H	-1.917775	1.171597	2.292351

SCF Energy: -1378.34866160
Sum of electronic and zero-point Energies= -1378.086485
Sum of electronic and thermal Energies= -1378.061686
Sum of electronic and thermal Enthalpies= -1378.060742
Sum of electronic and thermal Free Energies= -1378.142818

==> ./AOX-II/PA/PA/mono-/c3c4/RAF/c6 <==

O	0.968282	-1.798245	-0.040921
O	-1.780798	0.558664	-0.060310
O	2.966325	2.478374	-0.048637
O	0.399490	2.194166	-0.035558
O	5.594625	-1.312927	0.375633
C	1.882947	0.379479	-0.103997
C	2.055704	-1.038840	-0.054347
C	-0.335499	-1.315065	-0.058464
C	0.565916	0.955240	-0.062708
C	-0.594553	0.044853	-0.062029
C	-1.299214	-2.412478	-0.050313
C	3.012417	1.181301	-0.118045
C	3.278076	-1.674420	0.040057
C	4.458493	-0.903166	0.090769
C	4.349595	0.565678	-0.316828
C	-0.856116	-3.743770	0.074865
C	-2.685162	-2.193953	-0.170707
C	-1.754571	-4.800577	0.082450
C	-3.576289	-3.259800	-0.165080
C	-3.122146	-4.568269	-0.037348
H	3.319127	-2.744347	0.201520
H	4.413335	0.503046	-1.423727
H	0.201238	-3.953397	0.169528
H	-3.057239	-1.185243	-0.273144
H	-1.381417	-5.814478	0.182949
H	-4.638543	-3.060515	-0.262703
H	-3.822818	-5.396345	-0.031743
H	2.003576	2.732771	-0.007279
O	-5.196007	1.178059	0.440689
H	-4.533654	1.865237	0.305685
O	-3.020295	4.306890	-0.042766
H	-3.970656	4.211616	0.080641
H	-4.678310	0.367690	0.445422
H	-2.725937	4.830876	0.709759
Cu	-2.141427	2.484530	-0.043382
O	5.393320	1.343013	0.190011
H	6.095550	0.710512	0.406376

SCF Energy: -1378.37775744
Sum of electronic and zero-point Energies= -1378.115506
Sum of electronic and thermal Energies= -1378.090448
Sum of electronic and thermal Enthalpies= -1378.089504
Sum of electronic and thermal Free Energies= -1378.172221

==> ./AOX-II/PA/PA/mono-/c3c4/RAF/c1p <==

O	1.978505	0.379487	-0.655117
O	-1.432096	1.344313	-0.325600
O	-0.466770	-3.626819	0.439951
O	-1.676278	-1.316841	-0.013079
O	4.218116	-3.676300	0.030150
C	0.670844	-1.562711	-0.060281
C	1.930836	-0.933636	-0.303999
C	0.942403	1.234573	-0.299239
C	-0.520819	-0.828364	-0.124129
C	-0.416094	0.672538	-0.288722
C	1.263087	2.306899	0.773129
C	0.694082	-2.977530	0.208995
C	3.108114	-1.621233	-0.280511
C	3.130198	-3.032885	-0.001581
C	1.868483	-3.676014	0.242892
C	2.624519	2.338612	1.302095

C	1.104962	2.690722	-0.697501
C	3.684266	2.663417	0.532341
C	2.298776	3.150339	-1.402493
C	3.521380	3.083967	-0.837328
H	4.040566	-1.106084	-0.480398
H	1.854632	-4.739649	0.453787
H	2.733431	2.130194	2.361161
H	0.151737	3.123302	-0.982455
H	4.676679	2.683842	0.969727
H	2.158650	3.594171	-2.381993
H	4.391651	3.430440	-1.383825
H	-1.200297	-2.987278	0.341779
O	-5.184569	0.005000	-1.399749
H	-5.695312	0.752640	-1.073261
O	-4.530734	0.849887	1.596946
H	-5.460957	0.834509	1.352235
H	-4.955984	0.245290	-2.302555
H	-4.485863	0.312261	2.392973
Cu	-3.415118	-0.175787	-0.154555
O	0.246251	2.571836	1.682596
H	0.179868	3.527506	1.784114

SCF Energy: -1378.34236388
Sum of electronic and zero-point Energies= -1378.082408
Sum of electronic and thermal Energies= -1378.056952
Sum of electronic and thermal Enthalpies= -1378.056008
Sum of electronic and thermal Free Energies= -1378.137861

==> ./AOX-II/PA/PA/mono-/c3c4/RAF/c5 <==

O	0.912984	-1.932040	0.010906
O	-1.789422	0.357390	0.303439
O	2.917586	2.235142	-0.710968
O	0.273678	2.076423	0.246999
O	5.548960	-1.574256	-0.449386
C	1.796572	0.278640	0.121891
C	1.971064	-1.093654	0.004232
C	-0.344982	-1.506097	0.109603
C	0.512128	0.836541	0.202103
C	-0.624703	-0.106266	0.214178
C	-1.320011	-2.582045	0.068179
C	3.035520	1.141588	0.169203
C	3.203479	-1.700532	-0.168676
C	4.441491	-0.958333	-0.262282
C	4.303861	0.423103	-0.159005
C	-0.945229	-3.833220	-0.458908
C	-2.628502	-2.421884	0.558283
C	-1.851852	-4.878287	-0.505899
C	-3.525991	-3.478752	0.514612
C	-3.146505	-4.706332	-0.019500
H	3.234102	-2.780710	-0.267887
H	5.186877	1.053389	-0.225271
H	0.056147	-3.975954	-0.845281
H	-2.930666	-1.478818	0.988444
H	-1.549669	-5.831503	-0.925033
H	-4.528058	-3.342570	0.905891
H	-3.854313	-5.527304	-0.054962
H	2.037517	2.616532	-0.555719
O	-4.248245	2.475655	-0.171543
H	-4.520374	3.157783	-0.792741
O	-1.902969	4.635772	-0.487240
H	-2.610265	5.055204	0.012119
H	-4.563477	1.656206	-0.564326
H	-1.094993	5.000401	-0.113476
Cu	-1.952689	2.443246	-0.124558
O	3.105181	1.605332	1.516844
H	3.965098	2.021047	1.649952

SCF Energy: -1378.30865526
Sum of electronic and zero-point Energies= -1378.049493
Sum of electronic and thermal Energies= -1378.023373
Sum of electronic and thermal Enthalpies= -1378.022429
Sum of electronic and thermal Free Energies= -1378.107444

==> ./AOX-II/PA/PA/mono-/c3c4/RAF/c2 <==

O	-1.672279	-1.513483	-0.007142
O	-0.562694	1.808584	-0.028432
O	3.154558	-1.749373	-0.144286
O	1.876424	0.549162	-0.091309
O	0.528473	-5.641328	-0.156511
C	0.756632	-1.522915	-0.030429
C	-0.500484	-2.212796	-0.004623
C	-1.716858	-0.218763	0.577343
C	0.811882	-0.127467	-0.007755
C	-0.497850	0.615298	0.133504
C	-3.027237	0.408342	0.150235
C	1.945500	-2.341356	-0.109895
C	-0.582526	-3.568504	-0.042989
C	0.607258	-4.384667	-0.118289
C	1.871769	-3.704570	-0.146347
C	-3.520635	0.197148	-1.138068
C	-3.720584	1.247592	1.020383
C	-4.702965	0.805041	-1.542511
C	-4.905345	1.853416	0.613550
C	-5.399526	1.633262	-0.667080
H	-1.551476	-4.053553	-0.029023
H	2.782095	-4.290938	-0.200125
H	-2.983606	-0.445603	-1.825907
H	-3.340424	1.430371	2.018834

H	-5.080659	0.629935	-2.544088
H	-5.440082	2.499689	1.301138
H	-6.323145	2.105997	-0.983487
H	3.023781	-0.779909	-0.136794
O	2.781548	4.435908	-0.073285
H	3.468821	4.588897	-0.730033
O	5.281605	0.903817	-0.024304
H	5.209799	0.008488	0.317939
H	2.063631	5.025607	-0.326439
H	4.369109	1.207516	-0.070373
Cu	2.169656	2.509027	-0.094911
O	-1.549318	-0.301291	1.961368
H	-2.338604	-0.698616	2.349821

SCF Energy: -1378.39331704
Sum of electronic and zero-point Energies= -1378.131001
Sum of electronic and thermal Energies= -1378.105789
Sum of electronic and thermal Enthalpies= -1378.104845
Sum of electronic and thermal Free Energies= -1378.187757

==> ./AOX-II/PA/PA/mono-/c3c4/RAF/c8 <==

O	-1.270268	1.287485	-0.236830
O	1.894763	-0.408197	-0.141617
O	-2.339981	-3.310844	-0.015016
O	0.126120	-2.486600	-0.118590
O	-5.655178	-0.047047	0.448982
C	-1.714146	-1.011317	-0.151993
C	-2.118907	0.295014	-0.198722
C	0.092094	1.105054	-0.227717
C	-0.309514	-1.311490	-0.156059
C	0.625375	-0.174947	-0.184497
C	0.810225	2.380040	-0.231993
C	-2.738239	-2.047742	-0.044044
C	-3.551981	0.678894	-0.327783
C	-4.520908	-0.411565	0.120096
C	-4.073048	-1.758677	0.073943
C	0.186933	3.541037	0.253575
C	2.118868	2.483517	-0.728350
C	0.856282	4.757200	0.256606
C	2.780328	3.705058	-0.726336
C	2.157453	4.846264	-0.230131
H	-4.780206	-2.559623	0.252666
H	-0.824300	3.488879	0.639485
H	2.614444	1.607193	-1.122522
H	0.358663	5.639410	0.645099
H	3.789760	3.763713	-1.119598
H	2.680032	5.796765	-0.225011
H	-1.351188	-3.322881	-0.059855
O	3.344223	0.706479	1.976040
H	3.338458	1.655921	1.830843
O	3.878410	-3.850400	-0.119483
H	4.706321	-3.689009	0.344794
H	2.835472	0.352361	1.222622
H	3.456989	-4.563954	0.370719
Cu	2.719592	-2.195717	-0.117523
H	-3.727832	0.738339	-1.419302
O	-3.828380	1.912682	0.276277
H	-4.767347	1.882169	0.510560

SCF Energy: -1378.39213577
Sum of electronic and zero-point Energies= -1378.129300
Sum of electronic and thermal Energies= -1378.104364
Sum of electronic and thermal Enthalpies= -1378.103420
Sum of electronic and thermal Free Energies= -1378.186387

==> ./AOX-II/PA/PA/mono-/c3c4/RAF/c6p <==

O	1.549238	-1.617820	0.170161
O	-1.560797	0.144756	0.081073
O	2.617274	3.068911	-0.157293
O	0.120707	2.200286	-0.082929
O	6.002299	-0.184865	0.122278
C	1.980970	0.751725	0.001856
C	2.436176	-0.593334	0.106053
C	0.189212	-1.450334	0.107226
C	0.609748	1.040415	-0.016112
C	-0.350697	-0.112106	0.071789
C	-0.546069	-2.614412	-0.019595
C	2.992430	1.775282	-0.061575
C	3.762514	-0.920895	0.151201
C	4.770184	0.100968	0.085021
C	4.321685	1.462845	-0.025036
C	0.125198	-3.872614	-0.136301
C	-2.064286	-2.591345	-0.083792
C	-0.557995	-5.016853	-0.398679
C	-2.696302	-3.908984	-0.385689
C	-1.990650	-5.035549	-0.556701
H	4.062473	-1.959114	0.232589
H	5.060659	2.254964	-0.075528
H	1.199571	-3.906798	-0.018749
H	-0.012480	-5.950011	-0.486811
H	-3.778637	-3.899699	-0.456041
H	-2.485385	-5.972201	-0.785999
H	1.639028	3.102587	-0.155041
O	-3.582324	2.470026	1.545520
H	-4.403503	2.741228	1.123998
O	-3.306047	3.576791	-1.437697
H	-4.001079	3.986121	-0.912967
H	-3.721463	1.542670	1.761061

H -2.859904 4.316167 -1.861173
Cu -1.969416 2.490550 -0.080171
H -2.332510 -1.920543 -0.916587
O -2.664572 -2.097326 1.104828
H -2.552638 -1.136233 1.070683

SCF Energy: -1378.34960924
Sum of electronic and zero-point Energies= -1378.088522
Sum of electronic and thermal Energies= -1378.063172
Sum of electronic and thermal Enthalpies= -1378.062227
Sum of electronic and thermal Free Energies= -1378.144616

==> ./AOX-II/PA/PA/mono-/c3c4 <==
O -0.756832 -1.968698 -0.011553
O 1.664488 0.722727 -0.016076
O -3.259335 2.123355 -0.000620
O -0.590091 2.058606 0.009753
O -5.456421 -2.045697 -0.008235
C -1.924800 0.111352 -0.007473
C -1.932160 -1.304248 -0.011049
C 0.469196 -1.357759 -0.010517
C -0.688539 0.777293 -0.004597
C 0.548423 0.018902 -0.013785
C 1.563679 -2.327972 0.000079
C -3.196695 0.767499 -0.003688
C -3.092031 -2.047115 -0.011770
C -4.360096 -1.394657 -0.008513
C -4.357853 0.045071 -0.004601
C 1.281951 -3.706279 0.019734
C 2.912373 -1.931454 -0.005721
C 2.304104 -4.643925 0.034463
C 3.929556 -2.877964 0.008252
C 3.635612 -4.237386 0.028743
H -3.041834 -3.130012 -0.013166
H -5.309223 0.566880 -0.001950
H 0.254725 -4.046616 0.025142
H 3.159882 -0.880148 -0.021462
H 2.057230 -5.700334 0.050863
H 4.962397 -2.545698 0.003066
H 4.433602 -4.972056 0.040170
H -2.347509 2.469261 0.003204
O 3.346635 3.150135 -0.199821
H 3.568951 3.861005 0.411562
O 0.822959 4.680002 0.207873
H 1.435574 5.221718 -0.302174
H 3.887790 2.402489 0.078019
H -0.037565 4.821748 -0.203365
Cu 1.318912 2.648972 0.000765

SCF Energy: -1302.56321569
Sum of electronic and zero-point Energies= -1302.315240
Sum of electronic and thermal Energies= -1302.293023
Sum of electronic and thermal Enthalpies= -1302.292078
Sum of electronic and thermal Free Energies= -1302.366990

==> ./AOX-II/PA/PA/mono-/c4c5/vEA <==
O 1.876707 1.159255 -0.006173
O 1.110102 -2.362661 0.020083
O -2.904585 0.928215 0.008484
O -1.291773 -1.348155 0.019482
O -0.664651 5.089351 -0.023731
C -0.503566 0.919786 0.002411
C 0.665090 1.731130 -0.004512
C 2.083588 -0.199899 -0.005439
C -0.349441 -0.488272 0.008950
C 1.010538 -1.086731 0.006493
C 3.505536 -0.531639 -0.005197
C -1.737532 1.646687 0.000021
C 0.639486 3.113671 -0.012545
C -0.590864 3.816892 -0.015683
C -1.784910 3.009615 -0.009499
C 4.474327 0.485442 0.113104
C 3.968578 -1.856738 -0.127243
C 5.830173 0.190249 0.114477
C 5.328554 -2.141356 -0.129799
C 6.270894 -1.125566 -0.006536
H 1.575634 3.660714 -0.017977
H -2.748332 3.511361 -0.010893
H 4.160821 1.517223 0.208337
H 3.250585 -2.657229 -0.223328
H 6.549489 0.997126 0.211684
H 5.651674 -3.172911 -0.228916
H 7.331458 -1.353832 -0.005498
Cu -3.192979 -1.160000 0.005641
O -5.232496 -0.969106 0.014769
H -5.624771 -1.605541 -0.594242
O -3.451123 -3.290868 0.002132
H -4.143455 -3.515441 0.633544
H -2.649893 -3.691236 0.357944
H -5.479893 -0.103667 -0.331746
H -3.677079 1.505571 -0.016868

SCF Energy: -1302.68074200

==> ./AOX-II/PA/PA/mono-/c4c5/vIP <==
O 1.876707 1.159255 -0.006173
O 1.110102 -2.362661 0.020083

O	-2.904585	0.928215	0.008484
O	-1.291773	-1.348155	0.019482
O	-0.664651	5.089351	-0.023731
C	-0.503566	0.919786	0.002411
C	0.665090	1.731130	-0.004512
C	2.083588	-0.199899	-0.005439
C	-0.349441	-0.488272	0.008950
C	1.010538	-1.086731	0.006493
C	3.505536	-0.531639	-0.005197
C	-1.737532	1.646687	0.000021
C	0.639486	3.113671	-0.012545
C	-0.590864	3.816892	-0.015683
C	-1.784910	3.009615	-0.009499
C	4.474327	0.485442	0.113104
C	3.968578	-1.856738	-0.127243
C	5.830173	0.190249	0.114477
C	5.328554	-2.141356	-0.129799
C	6.270894	-1.125566	-0.006536
H	1.575634	3.660714	-0.017977
H	-2.748332	3.511361	-0.010893
H	4.160821	1.517223	0.208337
H	3.250585	-2.657229	-0.223328
H	6.549489	0.997126	0.211684
H	5.651674	-3.172911	-0.228916
H	7.331458	-1.353832	-0.005498
Cu	-3.192979	-1.160000	0.005641
O	-5.232496	-0.969106	0.014769
H	-5.624771	-1.605541	-0.594242
O	-3.451123	-3.290868	0.002132
H	-4.143455	-3.515441	0.633544
H	-2.649893	-3.691236	0.357944
H	-5.479893	-0.103667	-0.331746
H	-3.677079	1.505571	-0.016868

SCF Energy: -1302.36714658

==> ./AOX-II/PA/PA/mono-/c4c5/RAF/c4a <==

O	1.781438	1.351195	0.165789
O	0.959527	-2.039179	-0.857516
O	-2.896136	1.035712	1.001427
O	-1.452849	-0.808172	-0.760085
O	-1.047355	4.879833	-1.006757
C	-0.571016	1.045281	0.563513
C	0.560698	1.907395	0.086789
C	1.957736	-0.007594	-0.103616
C	-0.468255	-0.240867	-0.296834
C	0.882578	-0.819706	-0.441088
C	3.366509	-0.370609	-0.045726
C	-1.905020	1.747882	0.466636
C	0.412772	3.144997	-0.403245
C	-0.913032	3.747538	-0.524655
C	-2.051594	2.978136	-0.064406
C	4.339603	0.607022	0.244465
C	3.807857	-1.693180	-0.247570
C	5.683784	0.275850	0.330571
C	5.155811	-2.014146	-0.157484
C	6.103586	-1.036606	0.130182
H	1.270774	3.708715	-0.749331
H	-3.030914	3.442531	-0.107418
H	4.039416	1.633968	0.405950
H	3.084273	-2.461623	-0.474136
H	6.409011	1.050918	0.555632
H	5.466696	-3.041771	-0.315168
H	7.155505	-1.292886	0.197360
Cu	-3.305892	-1.374989	-0.218728
O	-5.098898	-2.251985	0.223055
H	-5.151320	-3.129266	-0.170050
O	-0.808307	-3.893803	0.115805
H	-0.247361	-4.505008	0.598064
H	-0.189615	-3.210702	-0.227165
H	-5.828805	-1.763812	-0.171657
H	-3.727713	1.525895	0.951815
O	-0.345223	0.592720	1.887381
H	-0.288753	1.358802	2.471253

SCF Energy: -1378.33537681
Sum of electronic and zero-point Energies= -1378.074186
Sum of electronic and thermal Energies= -1378.048839
Sum of electronic and thermal Enthalpies= -1378.047895
Sum of electronic and thermal Free Energies= -1378.131955

==> ./AOX-II/PA/PA/mono-/c4c5/RAF/c6 <==

O	1.562150	1.401392	-0.070065
O	1.324313	-2.096311	0.825834
O	-3.044942	0.719170	0.732383
O	-1.239977	-1.337104	0.887933
O	-1.407014	4.981495	-0.165937
C	-0.732959	0.896224	0.270898
C	0.313788	1.838140	-0.027753
C	1.961817	0.092960	0.178899
C	-0.389387	-0.472180	0.588388
C	1.040156	-0.877356	0.548892
C	3.399738	-0.056423	-0.015314
C	-2.019099	1.404485	0.310989
C	0.136756	3.194743	-0.223095
C	-1.150263	3.769244	-0.159731
C	-2.312011	2.778821	-0.196218
C	4.178869	1.038732	-0.440792
C	4.061651	-1.281292	0.199005
C	5.545230	0.911897	-0.643886
C	5.430644	-1.397318	-0.007362

C	6.184066	-0.306844	-0.429724
H	1.006894	3.831312	-0.326894
H	-2.451943	2.610749	-1.280177
H	3.708931	1.997682	-0.616440
H	3.492162	-2.138151	0.526044
H	6.115354	1.774546	-0.973311
H	5.911466	-2.354820	0.165512
H	7.252571	-0.403540	-0.590132
Cu	-2.677272	-2.133531	-0.229457
O	-4.101114	-3.002487	-1.384537
H	-3.906705	-3.934264	-1.530201
O	-0.174085	-4.345653	0.345046
H	-1.101919	-4.095465	0.357752
H	0.294899	-3.500849	0.519604
H	-4.108370	-2.612229	-2.264761
H	-3.822591	1.311722	0.730027
O	-3.502310	3.230231	0.387020
H	-3.980006	3.776852	-0.243370

SCF Energy: -1378.36472876
Sum of electronic and zero-point Energies= -1378.101854
Sum of electronic and thermal Energies= -1378.077136
Sum of electronic and thermal Enthalpies= -1378.076191
Sum of electronic and thermal Free Energies= -1378.157845

==> ./AOX-II/PA/PA/mono-/c4c5/RAF/c1p <==

O	-1.666632	1.127754	0.081873
O	-0.588197	-2.263076	-0.072920
O	3.094570	1.292453	-0.502127
O	1.727408	-1.092704	-0.289630
O	0.538416	5.231160	0.056747
C	0.745713	1.088987	-0.178197
C	-0.487217	1.790101	-0.013512
C	-1.800708	-0.229764	0.003297
C	0.750260	-0.321136	-0.191780
C	-0.590779	-1.036177	-0.079636
C	-3.100118	-0.685485	-0.013274
C	1.915891	1.922277	-0.298053
C	-0.575504	3.152722	0.064478
C	0.597069	3.970543	-0.024133
C	1.844342	3.285063	-0.218909
C	-4.208962	0.355707	0.056014
C	-3.465317	-2.068119	-0.105916
C	-5.572167	-0.161980	-0.268251
C	-4.756376	-2.433354	-0.314522
C	-5.822916	-1.467961	-0.438179
H	-1.544616	3.620790	0.191462
H	2.750695	3.875390	-0.313021
H	-3.966003	1.153047	-0.652343
H	-2.700479	-2.823638	-0.023400
H	-6.362089	0.580356	-0.316465
H	-4.999887	-3.487452	-0.392180
H	-6.827308	-1.818415	-0.648315
Cu	3.675113	-1.175562	0.049590
O	5.634602	-1.549935	0.393192
H	5.750754	-2.241474	1.053042
O	1.688993	-4.046714	0.178275
H	2.530971	-3.585105	0.124451
H	1.039272	-3.333933	0.059364
H	6.074334	-0.778651	0.765734
H	3.802256	1.945441	-0.560934
O	-4.233178	1.007034	1.330974
H	-4.533304	0.368624	1.986862

SCF Energy: -1378.34248891
Sum of electronic and zero-point Energies= -1378.081419
Sum of electronic and thermal Energies= -1378.055786
Sum of electronic and thermal Enthalpies= -1378.054841
Sum of electronic and thermal Free Energies= -1378.139090

==> ./AOX-II/PA/PA/mono-/c4c5/RAF/c2 <==

O	-1.776693	1.271541	-0.228627
O	-1.195396	-2.063705	0.592201
O	2.908185	1.052371	0.823650
O	1.336331	-1.202439	0.677997
O	0.908833	5.074970	-0.650756
C	0.591134	1.048790	0.299986
C	-0.541733	1.839746	-0.086556
C	-2.120215	0.156842	0.580323
C	0.447035	-0.340441	0.503984
C	-0.981270	-0.877991	0.537751
C	-3.420873	-0.395961	0.037604
C	1.842097	1.757311	0.386498
C	-0.448886	3.161729	-0.403597
C	0.812533	3.850656	-0.355797
C	1.948470	3.082928	0.064784
C	-3.662951	-0.406577	-1.336761
C	-4.362629	-0.950443	0.902651
C	-4.842184	-0.947146	-1.835354
C	-5.543540	-1.489145	0.401132
C	-5.786818	-1.487686	-0.967637
H	-1.336783	3.705504	-0.704601
H	2.909051	3.581292	0.154368
H	-2.932516	0.011642	-2.019405
H	-4.179554	-0.962273	1.970867
H	-5.023379	-0.944395	-2.904758
H	-6.272218	-1.911561	1.084522
H	-6.707793	-1.907195	-1.358116

Cu	3.181047	-1.384063	-0.138527
O	5.063689	-1.075826	-1.059212
H	5.664049	-1.792695	-0.830449
O	3.541847	-3.813911	-0.158268
H	4.218347	-3.928922	-0.831896
H	2.762202	-4.232631	-0.534069
H	5.476811	-0.285970	-0.696486
H	3.686344	1.622956	0.839441
O	-2.177555	0.521317	1.927648
H	-2.969874	1.051858	2.076734

SCF Energy: -1378.38057402
Sum of electronic and zero-point Energies= -1378.120168
Sum of electronic and thermal Energies= -1378.093890
Sum of electronic and thermal Enthalpies= -1378.092946
Sum of electronic and thermal Free Energies= -1378.179503

==> ./AOX-II/PA/PA/mono-/c4c5/RAF/c8 <==

O	1.641897	0.919055	-0.176740
O	0.471793	-2.343756	0.741434
O	-2.937073	1.533815	0.755772
O	-1.769949	-0.916011	0.890774
O	-0.072780	5.180183	0.006139
C	-0.659546	1.104808	0.271309
C	0.556660	1.631342	-0.106741
C	1.699480	-0.422663	0.119086
C	-0.725878	-0.305281	0.554263
C	0.530966	-1.098509	0.475106
C	3.059089	-0.941383	0.002414
C	-1.764495	2.053704	0.408747
C	0.720509	3.037019	-0.577362
C	-0.351786	3.979225	-0.058180
C	-1.613670	3.407641	0.251861
C	4.125288	-0.073886	-0.305713
C	3.356851	-2.303149	0.192268
C	5.424091	-0.547869	-0.416192
C	4.661726	-2.767877	0.077939
C	5.704019	-1.898835	-0.225598
H	-2.446309	4.065473	0.476481
H	3.936245	0.981450	-0.457182
H	2.558983	-2.991255	0.428266
H	6.224499	0.145617	-0.652570
H	4.861591	-3.823769	0.228870
H	6.720707	-2.266899	-0.312476
Cu	-3.386301	-1.303628	-0.177183
O	-4.955747	-1.948291	-1.284819
H	-5.764081	-1.942752	-0.761304
O	-1.517433	-4.112593	-0.004389
H	-2.351232	-3.634209	-0.030595
H	-0.865828	-3.433269	0.268416
H	-4.823179	-2.872476	-1.521515
H	-3.595794	2.235544	0.849507
H	0.507983	2.991927	-1.663319
O	2.012513	3.529520	-0.356374
H	1.913560	4.488742	-0.267894

SCF Energy: -1378.37628533
Sum of electronic and zero-point Energies= -1378.112995
Sum of electronic and thermal Energies= -1378.088337
Sum of electronic and thermal Enthalpies= -1378.087393
Sum of electronic and thermal Free Energies= -1378.169357

==> ./AOX-II/PA/PA/mono-/c4c5/RAF/c5 <==

O	1.883683	1.195482	-0.024211
O	1.185128	-2.268620	-0.044831
O	-2.469800	1.218098	-1.336908
O	-1.301027	-1.337513	-0.156311
O	-0.542238	5.163943	-0.018799
C	-0.492513	0.912179	-0.103563
C	0.641931	1.723881	-0.050363
C	2.119650	-0.104740	-0.039514
C	-0.363991	-0.486484	-0.118501
C	1.015396	-1.040825	-0.073173
C	3.528845	-0.439971	0.020843
C	-1.826462	1.628208	-0.142926
C	0.626368	3.105639	-0.025190
C	-0.588787	3.884443	-0.052634
C	-1.748398	3.122112	-0.114684
C	4.447131	0.527303	0.476295
C	4.016541	-1.696393	-0.383773
C	5.799463	0.240330	0.537576
C	5.375358	-1.967910	-0.329867
C	6.270076	-1.008016	0.134010
H	1.579326	3.622474	0.019495
H	-2.713683	3.624574	-0.137442
H	4.091119	1.497669	0.798873
H	3.332782	-2.445316	-0.754115
H	6.490776	0.991027	0.903658
H	5.737936	-2.936395	-0.655567
H	7.330640	-1.229722	0.180557
Cu	-3.267137	-1.371101	0.138298
O	-5.234846	-1.707798	0.474333
H	-5.407482	-2.642273	0.628863
O	-1.330569	-4.174374	-0.177662
H	-2.290054	-4.214233	-0.179189
H	-1.138940	-3.219849	-0.164356
H	-5.760279	-1.486126	-0.301506
H	-3.197877	1.823734	-1.516344

O	-2.553726	1.188296	0.991610
H	-3.323762	1.757724	1.101445

SCF Energy: -1378.31009213

Sum of electronic and zero-point Energies= -1378.050542

Sum of electronic and thermal Energies= -1378.024622

Sum of electronic and thermal Enthalpies= -1378.023677

Sum of electronic and thermal Free Energies= -1378.109010

==> ./AOX-II/PA/PA/mono-/c4c5/RAF/c6p <==

O	1.445932	1.578209	0.284999
O	0.858110	-1.900169	-0.183855
O	-3.205517	1.192522	-0.854140
O	-1.550667	-1.000921	-0.729625
O	-1.313776	5.331923	0.419333
C	-0.896917	1.252771	-0.258993
C	0.198096	2.086090	0.130042
C	1.760407	0.260270	0.099091
C	-0.703281	-0.136978	-0.406328
C	0.689299	-0.677485	-0.143989
C	3.110433	-0.032247	0.065451
C	-2.155022	1.935904	-0.440055
C	0.080237	3.429176	0.362716
C	-1.179242	4.091649	0.209463
C	-2.285535	3.276965	-0.209627
C	4.070441	1.027502	0.136597
C	3.611018	-1.458690	-0.089045
C	5.396494	0.791597	-0.034581
C	5.085092	-1.585009	-0.278844
C	5.914442	-0.532209	-0.275980
H	0.952967	4.001010	0.655611
H	-3.250470	3.752595	-0.357649
H	3.725147	2.035703	0.320938
H	6.093128	1.621322	0.016540
H	5.451316	-2.596579	-0.417269
H	6.979639	-0.662957	-0.427712
Cu	-3.250454	-1.618252	0.081898
O	-4.971436	-2.286148	0.933324
H	-5.023119	-3.246627	0.890382
O	-0.987292	-4.095174	-0.284011
H	-0.335044	-4.771057	-0.084868
H	-0.476934	-3.268978	-0.297223
H	-5.737389	-1.977239	0.438285
H	-3.980340	1.761265	-0.934987
H	3.143977	-1.863235	-1.001655
O	3.253751	-2.289364	1.006841
H	2.311677	-2.478793	0.886122

SCF Energy: -1378.34557289

Sum of electronic and zero-point Energies= -1378.083921

Sum of electronic and thermal Energies= -1378.058812

Sum of electronic and thermal Enthalpies= -1378.057868

Sum of electronic and thermal Free Energies= -1378.140086

==> ./AOX-II/PA/PA/mono-/c4c5/RAF/c5p <==

O	1.433048	1.522745	-0.085006
O	1.020513	-1.996296	0.574828
O	-3.283460	0.916151	0.507313
O	-1.513787	-1.183123	0.608135
O	-1.456930	5.184110	-0.395612
C	-0.910176	1.089683	0.213869
C	0.170258	1.982712	-0.032600
C	1.756498	0.199319	0.110434
C	-0.642175	-0.296735	0.396398
C	0.783915	-0.761455	0.370427
C	3.181719	-0.024037	-0.000655
C	-2.206318	1.707019	0.254443
C	0.016268	3.339673	-0.237676
C	-1.271277	3.935540	-0.207712
C	-2.373056	3.051364	0.053623
C	4.073316	1.047933	-0.256842
C	3.755945	-1.282486	0.167674
C	5.454862	0.892069	-0.372149
C	5.169480	-1.494705	-0.054838
C	6.035131	-0.340568	-0.211821
H	0.891762	3.951808	-0.424080
H	-3.372809	3.474559	0.099760
H	3.664752	2.042916	-0.376029
H	3.138985	-2.146922	0.371364
H	6.071523	1.761368	-0.562593
H	7.104107	-0.499926	-0.291862
Cu	-2.937542	-1.959542	-0.513302
O	-4.468841	-2.753989	-1.594473
H	-5.265364	-2.753240	-1.052422
O	-4.797047	-1.258695	1.856064
H	-4.812712	-1.985575	1.225853
H	-4.122937	-0.669038	1.494322
H	-4.304789	-3.683055	-1.787668
H	-4.076396	1.465046	0.496431
H	4.982087	-1.608323	-1.183572
O	5.665265	-2.672810	0.480569
H	6.534079	-2.859586	0.106270

SCF Energy: -1378.30482993

Sum of electronic and zero-point Energies= -1378.045871

Sum of electronic and thermal Energies= -1378.020388

Sum of electronic and thermal Enthalpies= -1378.019443
Sum of electronic and thermal Free Energies= -1378.102862

==> ./AOX-II/PA/PA/mono-/c4c5/RAF/c7 <==
O -1.648690 1.116147 0.057553
O -0.936463 -2.321371 -0.645388
O 3.038232 0.984731 -0.733744
O 1.480091 -1.251324 -0.823341
O -0.087517 3.971319 -0.860237
C 0.677422 0.951723 -0.343843
C -0.462394 1.667682 -0.047243
C -1.883772 -0.217422 -0.134016
C 0.537031 -0.467096 -0.551365
C -0.819480 -1.065907 -0.451368
C -3.298191 -0.548735 0.025462
C 1.946454 1.698709 -0.372185
C -0.471738 3.114507 0.219671
C 0.821682 3.789426 0.232075
C 2.026269 3.007928 -0.054938
C -4.234563 0.464584 0.308693
C -3.777919 -1.865090 -0.101596
C -5.582894 0.173590 0.453955
C -5.130870 -2.146602 0.047181
C -6.043232 -1.134492 0.324180
H -1.292335 3.485734 0.826228
H 2.985020 3.516744 -0.053554
H -3.904307 1.490353 0.413075
H -3.083241 -2.662523 -0.318459
H -6.279829 0.976945 0.669248
H -5.471658 -3.171643 -0.056755
H -7.098361 -1.359579 0.437224
Cu 3.188302 -1.683400 0.082145
O 4.899260 -2.331280 0.957905
H 4.707166 -3.017852 1.605164
O 0.839509 -4.183762 0.365788
H 1.719014 -3.797533 0.321109
H 0.262473 -3.487451 -0.013375
H 5.309394 -1.623683 1.466227
H 3.807417 1.566395 -0.750040
O 0.957145 4.886557 1.053603
H 1.723119 5.397716 0.766615

SCF Energy: -1378.33930076
Sum of electronic and zero-point Energies= -1378.076351
Sum of electronic and thermal Energies= -1378.052112
Sum of electronic and thermal Enthalpies= -1378.051168
Sum of electronic and thermal Free Energies= -1378.131448

==> ./AOX-II/PA/PA/mono-/c4c5/RAF/c2p <==
O -1.551929 0.955212 -0.146983
O -0.976883 -2.490363 0.562930
O 3.035023 0.618940 1.199414
O 1.499468 -1.606526 0.674656
O 1.160424 4.747614 -0.144124
C 0.769404 0.661451 0.477850
C -0.324663 1.483969 0.073316
C -1.882365 -0.349655 0.090576
C 0.609318 -0.738604 0.521072
C -0.805883 -1.289679 0.393441
C -3.222887 -0.639669 -0.025158
C 2.005745 1.357851 0.727031
C -0.214627 2.830211 -0.145116
C 1.032712 3.505534 0.055499
C 2.132569 2.701944 0.514714
C -4.165669 0.511928 -0.323907
C -3.773434 -1.955506 0.115957
C -5.558084 0.105209 -0.685958
C -5.079224 -2.199887 -0.163075
C -5.981543 -1.163648 -0.606622
H -1.080928 3.393855 -0.471171
H 3.080755 3.189655 0.720001
H -3.132342 -2.761963 0.434059
H -6.224271 0.909316 -0.982957
H -5.461898 -3.208732 -0.052371
H -7.004139 -1.426502 -0.853642
Cu 3.395238 -1.661755 0.060974
O 5.252081 -1.943846 -0.728368
H 5.285234 -2.767192 -1.226464
O 2.785530 0.625425 -2.536404
H 2.953033 -0.001581 -1.824447
H 2.462415 1.407752 -2.078773
H 5.901931 -2.056441 -0.026815
H 3.802770 1.188026 1.333811
H -3.744535 1.082453 -1.160732
O -4.200405 1.357247 0.835360
H -4.574632 2.203941 0.571676

SCF Energy: -1378.33976428
Sum of electronic and zero-point Energies= -1378.079595
Sum of electronic and thermal Energies= -1378.053274
Sum of electronic and thermal Enthalpies= -1378.052330
Sum of electronic and thermal Free Energies= -1378.138843

==> ./AOX-II/PA/PA/mono-/c4c5/RAF/c4p <==
O 1.296633 1.474855 0.064620
O 0.705421 -1.984001 -0.579359

O	-3.424894	1.051037	-0.681024
O	-1.760823	-1.132481	-0.607262
O	-1.447024	5.236613	0.279513
C	-1.078239	1.137893	-0.288104
C	0.044007	1.981178	-0.023926
C	1.615610	0.156027	-0.127395
C	-0.889655	-0.252125	-0.428327
C	0.537623	-0.781801	-0.387728
C	2.964448	-0.132602	-0.046118
C	-2.349416	1.812434	-0.379434
C	-0.057769	3.332832	0.167732
C	-1.327895	3.989398	0.101758
C	-2.465466	3.160865	-0.187811
C	3.914691	0.939624	0.203770
C	3.505453	-1.474018	-0.163191
C	5.237379	0.718728	0.219420
C	4.828813	-1.699631	-0.136799
C	5.840303	-0.613751	-0.067799
H	0.834004	3.914137	0.371125
H	-3.440520	3.631467	-0.271195
H	3.536093	1.936174	0.384979
H	2.823785	-2.303961	-0.256475
H	5.927757	1.534593	0.411004
H	5.209711	-2.713446	-0.204920
H	6.307736	-0.548924	-1.066280
Cu	-3.496640	-1.570743	0.256454
O	-5.126758	-2.251765	1.260817
H	-5.479834	-3.038991	0.833322
O	-1.165685	-4.165080	-0.449276
H	-0.475071	-4.831764	-0.430150
H	-0.682297	-3.324654	-0.514032
H	-5.843356	-1.609762	1.221739
H	-4.208056	1.612378	-0.723485
O	6.844542	-0.974249	0.877441
H	7.601999	-0.395452	0.747167

SCF Energy: -1378.34661665
Sum of electronic and zero-point Energies= -1378.086635
Sum of electronic and thermal Energies= -1378.060352
Sum of electronic and thermal Enthalpies= -1378.059408
Sum of electronic and thermal Free Energies= -1378.147054

==> ./AOX-II/PA/PA/mono-/c4c5/RAF/c4 <==

O	-1.644345	1.446829	0.239504
O	-1.025281	-1.092401	-1.894182
O	3.077999	1.163669	-0.643378
O	1.455894	-1.133511	-0.686997
O	0.963986	5.350223	0.080191
C	0.739095	1.171855	-0.183664
C	-0.354534	2.009256	0.061319
C	-1.923141	0.263262	-0.167436
C	0.535130	-0.344151	-0.152758
C	-0.814946	-0.495761	-0.868137
C	-3.234590	-0.244766	0.049993
C	1.949259	1.873413	-0.379770
C	-0.344271	3.375517	0.162359
C	0.891706	4.076818	-0.012642
C	2.016946	3.258790	-0.298855
C	-4.237658	0.605494	0.566356
C	-3.534979	-1.594907	-0.232318
C	-5.510135	0.116977	0.772491
C	-4.812705	-2.072094	-0.009586
C	-5.797607	-1.219473	0.484730
H	-1.263034	3.915276	0.358586
H	2.979212	3.740304	-0.453974
H	-4.006817	1.640795	0.782127
H	-2.771039	-2.263948	-0.605676
H	-6.285057	0.769044	1.156537
H	-5.044777	-3.109032	-0.218758
H	-6.799802	-1.598348	0.650805
Cu	3.059641	-1.665045	0.282046
O	4.766440	-2.314721	1.132312
H	4.631256	-3.159067	1.575413
O	0.521326	-3.772922	-0.775335
H	-0.264620	-3.733205	-1.325729
H	0.852227	-2.851256	-0.769488
H	5.051785	-1.713998	1.828856
H	3.818577	1.774979	-0.721312
O	0.233894	-0.669063	1.238745
H	0.167758	-1.631446	1.283912

SCF Energy: -1378.32109010
Sum of electronic and zero-point Energies= -1378.060329
Sum of electronic and thermal Energies= -1378.035224
Sum of electronic and thermal Enthalpies= -1378.034279
Sum of electronic and thermal Free Energies= -1378.116312

==> ./AOX-II/PA/PA/mono-/c4c5/RAF/c8a <==

O	-1.523482	0.925258	-1.636042
O	-1.154720	0.743430	1.779670
O	3.156609	-0.791020	-0.223825
O	0.436684	-1.127388	0.002013
O	4.796842	3.636969	-0.029339
C	1.596434	0.985114	0.078012
C	1.422072	2.411688	0.163594
C	-1.944468	0.693274	-0.518722
C	0.490459	0.118047	0.168666
C	-0.891405	0.591955	0.612548

C	-3.378545	0.570896	-0.197922
C	2.954711	0.541424	-0.098919
C	2.470034	3.288043	0.130698
C	3.822064	2.830598	-0.004351
C	4.008822	1.413649	-0.125204
C	-4.299733	1.202713	-1.043946
C	-3.841678	-0.189059	0.883944
C	-5.659478	1.092911	-0.801319
C	-5.206519	-0.314825	1.107900
C	-6.113697	0.331234	0.273849
H	2.279004	4.355265	0.185662
H	5.017914	1.031501	-0.245901
H	-3.937808	1.790507	-1.879734
H	-3.144892	-0.703975	1.532926
H	-6.367523	1.597025	-1.449390
H	-5.561830	-0.916538	1.936716
H	-7.178365	0.240231	0.460065
Cu	1.640556	-2.743805	-0.017268
O	2.534248	-4.575381	-0.018526
H	1.976827	-5.235225	0.406473
O	-1.781919	-2.871311	-0.594039
H	-2.588221	-2.349292	-0.602285
H	-1.088946	-2.231715	-0.368373
H	3.343805	-4.565067	0.502235
H	4.100267	-0.966898	-0.319586
O	0.145057	2.848249	0.249272
H	0.124644	3.808113	0.335511

SCF Energy: -1378.38314553
Sum of electronic and zero-point Energies= -1378.122816
Sum of electronic and thermal Energies= -1378.096625
Sum of electronic and thermal Enthalpies= -1378.095681
Sum of electronic and thermal Free Energies= -1378.181565

==> ./AOX-II/PA/PA/mono-/c4c5/RAF/c3 <==

O	1.766732	1.363383	-0.357990
O	1.285313	-0.692660	-1.409468
O	-2.925753	1.128265	0.726272
O	-1.393783	-1.136331	0.547546
O	-0.867697	5.212054	-0.470811
C	-0.626688	1.103134	0.117140
C	0.513170	1.900681	-0.234701
C	1.959115	-0.010696	-0.375305
C	-0.516863	-0.299294	0.233615
C	0.841215	-0.888721	-0.058922
C	3.384347	-0.355123	-0.106495
C	-1.846811	1.836917	0.315086
C	0.443920	3.250169	-0.436894
C	-0.789318	3.962743	-0.282095
C	-1.928426	3.187624	0.114038
C	4.065554	0.306515	0.916494
C	4.032459	-1.344951	-0.841965
C	5.386543	-0.020767	1.197442
C	5.356325	-1.665578	-0.560807
C	6.035107	-1.006256	0.458757
H	1.341812	3.795921	-0.702627
H	-2.874077	3.696582	0.275329
H	3.563985	1.076310	1.492135
H	3.504059	-1.861852	-1.634260
H	5.909438	0.495521	1.995127
H	5.856979	-2.432755	-1.141417
H	7.066877	-1.259481	0.677704
Cu	-3.421816	-1.181622	0.251176
O	-5.303628	-1.779107	-0.286885
H	-5.273102	-2.624460	-0.746306
O	-1.812466	-4.074629	-0.533641
H	-1.150154	-4.447066	0.054988
H	-1.836880	-3.141235	-0.292682
H	-5.690400	-1.168670	-0.922870
H	-3.686245	1.715501	0.811176
O	1.058019	-2.120686	0.470925
H	0.188591	-2.517965	0.629688

SCF Energy: -1378.37361529
Sum of electronic and zero-point Energies= -1378.110954
Sum of electronic and thermal Energies= -1378.085919
Sum of electronic and thermal Enthalpies= -1378.084975
Sum of electronic and thermal Free Energies= -1378.167908

==> ./AOX-II/PA/PA/mono-/c4c5/RAF/c3p <==

O	-1.359637	1.271616	-0.014719
O	-0.349960	-2.061204	0.915794
O	3.368079	1.543735	0.689279
O	1.979176	-0.772238	1.053459
O	0.861270	5.342393	-0.619826
C	1.010321	1.290589	0.364769
C	-0.198471	1.952057	0.011944
C	-1.447241	-0.071121	0.267072
C	0.977808	-0.093668	0.690779
C	-0.333297	-0.812995	0.632516
C	-2.810141	-0.551685	0.132494
C	2.182768	2.120998	0.357792
C	-0.275201	3.290052	-0.318053
C	0.889897	4.101656	-0.323076
C	2.120933	3.449312	0.028608
C	-3.841466	0.329562	-0.174325
C	-3.174542	-1.906851	0.332613
C	-5.186239	-0.140718	-0.425262

C	-4.482684	-2.377434	0.199288
C	-5.508495	-1.521009	-0.105731
H	-1.236322	3.721312	-0.575219
H	3.032432	4.040843	0.036965
H	-3.658888	1.385400	-0.326722
H	-2.390715	-2.603016	0.595877
H	-4.685318	-3.427087	0.370421
H	-6.535316	-1.851362	-0.211596
Cu	3.383387	-1.572284	-0.077640
O	4.790772	-2.442955	-1.241883
H	4.925229	-3.361721	-0.986672
O	0.962988	-3.776204	-0.794945
H	1.833621	-3.394356	-0.939355
H	0.535193	-3.153012	-0.171309
H	5.647233	-2.023093	-1.108978
H	4.061127	2.211917	0.638523
H	-4.939901	-0.376437	-1.522597
O	-6.162009	0.839628	-0.341250
H	-6.975734	0.516215	-0.743605

SCF Energy: -1378.30853792
Sum of electronic and zero-point Energies= -1378.049361
Sum of electronic and thermal Energies= -1378.024399
Sum of electronic and thermal Enthalpies= -1378.023455
Sum of electronic and thermal Free Energies= -1378.104974

==> ./AOX-II/PA/PA/mono-/c4c5/aEA <==

O	1.584025	1.349303	-0.093154
O	0.861203	-2.065275	0.814144
O	-3.106229	1.276175	0.828914
O	-1.554377	-0.969148	0.989232
O	-0.944109	5.261110	-0.550798
C	-0.759871	1.190163	0.365201
C	0.381470	1.942506	-0.015951
C	1.798160	0.016591	0.177289
C	-0.608617	-0.200514	0.643237
C	0.745814	-0.809177	0.550319
C	3.206641	-0.338288	0.012751
C	-1.983464	1.936546	0.434567
C	0.349397	3.290518	-0.326880
C	-0.867788	4.014713	-0.272687
C	-2.030988	3.271444	0.124513
C	4.143155	0.641443	-0.376203
C	3.690419	-1.643516	0.229517
C	5.485981	0.331554	-0.537965
C	5.037289	-1.944169	0.065006
C	5.946962	-0.964296	-0.319177
H	1.267678	3.792929	-0.610961
H	-2.979113	3.798331	0.194556
H	3.814126	1.657397	-0.552785
H	2.999500	-2.417584	0.527349
H	6.178343	1.111749	-0.838047
H	5.376104	-2.960437	0.240395
H	6.997262	-1.204612	-0.446073
Cu	-3.074917	-1.588741	-0.100892
O	-4.511321	-2.429554	-1.249820
H	-4.113160	-2.891552	-1.994978
O	-0.688663	-3.978026	-0.367474
H	-1.599204	-3.670125	-0.348504
H	-0.184467	-3.252766	0.065807
H	-5.073294	-1.757127	-1.648832
H	-3.842789	1.897494	0.848376

SCF Energy: -1302.70698022
Sum of electronic and zero-point Energies= -1302.461571
Sum of electronic and thermal Energies= -1302.438348
Sum of electronic and thermal Enthalpies= -1302.437404
Sum of electronic and thermal Free Energies= -1302.515931

==> ./AOX-II/PA/PA/mono-/c4c5/HAT/h2o <==

O	1.958020	1.145576	0.138163
O	1.292924	-2.249090	0.392593
O	-2.752234	0.773955	-0.697066
O	-1.136820	-1.470373	-0.402489
O	-0.738330	4.934995	0.293195
C	-0.441844	0.822831	-0.203687
C	0.668907	1.666228	0.065699
C	2.245484	-0.108549	0.072024
C	-0.283167	-0.579668	-0.217936
C	1.104668	-1.094435	0.092815
C	3.624974	-0.452204	0.021099
C	-1.695326	1.524802	-0.362675
C	0.613388	3.009779	0.225470
C	-0.665147	3.695352	0.126299
C	-1.796847	2.880770	-0.187734
C	4.591469	0.546833	0.293681
C	4.050758	-1.751972	-0.341117
C	5.932921	0.242519	0.234234
C	5.400418	-2.034496	-0.414152
C	6.338715	-1.046844	-0.120802
H	1.513710	3.578044	0.421944
H	-2.761766	3.361716	-0.310281
H	4.271284	1.542090	0.572802
H	3.329618	-2.515684	-0.589594
H	6.670943	1.002082	0.460262
H	5.726308	-3.025246	-0.705672
H	7.396292	-1.279434	-0.173744
Cu	-2.990835	-1.801849	0.149808

O	-6.064108	0.009652	-0.234879
H	-5.590586	-0.802308	0.080292
O	-4.774157	-2.158021	0.719943
H	-5.064211	-2.983644	0.329162
H	-5.628244	0.236592	-1.058891
H	-3.547738	1.320167	-0.745694

SCF Energy: -1301.89070574
Sum of electronic and zero-point Energies= -1301.656104
Sum of electronic and thermal Energies= -1301.633279
Sum of electronic and thermal Enthalpies= -1301.632335
Sum of electronic and thermal Free Energies= -1301.710689

==> ./AOX-II/PA/PA/mono-/c4c5/HAT/c5 <==

O	1.824528	1.173472	0.019448
O	1.396791	-2.116549	1.147443
O	-2.903564	0.732094	0.700211
O	-1.193659	-1.453076	0.803360
O	-0.964275	4.885550	-0.377540
C	-0.540222	0.805396	0.398302
C	0.541493	1.663233	0.112362
C	2.148507	-0.068233	0.242074
C	-0.323083	-0.574071	0.606957
C	1.109502	-1.017792	0.691521
C	3.523506	-0.398678	-0.020322
C	-1.871704	1.426167	0.408445
C	0.428630	2.997992	-0.145490
C	-0.874972	3.638458	-0.146801
C	-1.979133	2.798896	0.106815
C	4.459281	0.647042	-0.181836
C	3.960198	-1.733221	-0.152561
C	5.785057	0.362821	-0.441772
C	5.289448	-2.002693	-0.428516
C	6.204171	-0.962139	-0.567491
H	1.313366	3.581355	-0.372306
H	-2.972229	3.237384	0.122497
H	4.138876	1.675724	-0.079491
H	3.256448	-2.547053	-0.063478
H	6.498091	1.171856	-0.548446
H	5.613801	-3.030470	-0.541021
H	7.245041	-1.181324	-0.777822
Cu	-3.161975	-1.147001	-0.099424
O	-5.280485	-0.794849	-0.781752
H	-5.487354	-1.428159	-1.475854
O	-3.246475	-3.231815	-0.786776
H	-4.129279	-3.587103	-0.643740
H	-2.678560	-3.750025	-0.207886
H	-5.327898	0.063162	-1.214427

SCF Energy: -1301.90594532
Sum of electronic and zero-point Energies= -1301.673128
Sum of electronic and thermal Energies= -1301.649348
Sum of electronic and thermal Enthalpies= -1301.648403
Sum of electronic and thermal Free Energies= -1301.727473

==> ./AOX-II/PA/PA/mono-/c4c5/aIP <==

O	1.906608	1.162361	-0.036424
O	1.128783	-2.284884	0.157597
O	-2.899298	0.957123	0.012188
O	-1.292891	-1.335520	0.029234
O	-0.613320	5.082849	-0.084094
C	-0.507829	0.934915	-0.004973
C	0.670151	1.740168	-0.025113
C	2.119141	-0.155285	-0.007866
C	-0.379354	-0.462483	0.019159
C	1.004247	-1.057589	0.056973
C	3.519001	-0.529085	-0.012280
C	-1.744728	1.674400	-0.011689
C	0.664795	3.103604	-0.050716
C	-0.577874	3.826674	-0.058806
C	-1.780868	3.035598	-0.037469
C	4.493175	0.431127	0.325731
C	3.943518	-1.824799	-0.364544
C	5.837316	0.098194	0.332125
C	5.292794	-2.143183	-0.365063
C	6.243937	-1.189933	-0.011717
H	1.600982	3.647850	-0.069120
H	-2.737099	3.548880	-0.040773
H	4.188059	1.433072	0.599815
H	3.217990	-2.570955	-0.651347
H	6.572878	0.845227	0.608243
H	5.602893	-3.143267	-0.647008
H	7.297283	-1.447766	-0.007179
Cu	-3.211513	-1.181304	0.014319
O	-5.234449	-0.973991	0.027288
H	-5.635539	-1.604274	-0.582811
O	-3.467672	-3.301391	0.017002
H	-4.163901	-3.521000	0.646282
H	-2.669325	-3.702391	0.378870
H	-5.470815	-0.103669	-0.315792
H	-3.676132	1.529798	-0.019268

SCF Energy: -1302.37388624
Sum of electronic and zero-point Energies= -1302.124963
Sum of electronic and thermal Energies= -1302.102706

Sum of electronic and thermal Enthalpies=
-1302.101762
Sum of electronic and thermal Free Energies=
-1302.176005

==> ./AOX-II/PA/PA/mono-/c4c5 <==

O	1.876707	1.159255	-0.006173
O	1.110102	-2.362661	0.020083
O	-2.904585	0.928215	0.008484
O	-1.291773	-1.348155	0.019482
O	-0.664651	5.089351	-0.023731
C	-0.503566	0.919786	0.002411
C	0.665090	1.731130	-0.004512
C	2.083588	-0.199899	-0.005439
C	-0.349441	-0.488272	0.008950
C	1.010538	-1.086731	0.006493
C	3.505536	-0.531639	-0.005197
C	-1.737532	1.646687	0.000021
C	0.639486	3.113671	-0.012545
C	-0.590864	3.816892	-0.015683
C	-1.784910	3.009615	-0.009499
C	4.474327	0.485442	0.113104
C	3.968578	-1.856738	-0.127243
C	5.830173	0.190249	0.114477
C	5.328554	-2.141356	-0.129799
C	6.270894	-1.125566	-0.006536
H	1.575634	3.660714	-0.017977
H	-2.748332	3.511361	-0.010893
H	4.160821	1.517223	0.208337
H	3.250585	-2.657229	-0.223328
H	6.549489	0.997126	0.211684
H	5.651674	-3.172911	-0.228916
H	7.331458	-1.353832	-0.005498
Cu	-3.192979	-1.160000	0.005641
O	-5.232496	-0.969106	0.014769
H	-5.624771	-1.605541	-0.594242
O	-3.451123	-3.290868	0.002132
H	-4.143455	-3.515441	0.633544
H	-2.649893	-3.691236	0.357944
H	-5.479893	-0.103667	-0.331746
H	-3.677079	1.505571	-0.016868

SCF Energy: -1302.53456830
Sum of electronic and zero-point Energies=
-1302.286843
Sum of electronic and thermal Energies=
-1302.264473
Sum of electronic and thermal Enthalpies=
-1302.263529
Sum of electronic and thermal Free Energies=
-1302.338689

==> ./AOX-II/PA/PA/aIP <==

O	-0.231661	-0.817729	0.000124
O	-1.210630	2.601499	-0.000363
O	3.843928	1.723353	0.000049
O	1.470055	2.879203	-0.000342
O	3.915036	-2.988662	0.000125
C	1.756779	0.539517	0.000053
C	1.131970	-0.727828	0.000105
C	-1.056226	0.239029	0.000021
C	0.989190	1.723750	0.000001
C	-0.513378	1.578075	-0.000157
C	-2.457708	-0.133785	0.000013
C	3.192355	0.542715	0.000031
C	1.820360	-1.907966	0.000133
C	3.261149	-1.904187	0.000076
C	3.909838	-0.625427	0.000037
C	-2.806693	-1.501829	-0.000523
C	-3.498399	0.817362	0.000573
C	-4.133421	-1.896172	-0.000529
C	-4.823971	0.409689	0.000589
C	-5.150956	-0.943418	0.000032
H	1.287906	-2.851594	0.000183
H	4.993791	-0.587394	0.000032
H	-2.029461	-2.254436	-0.000969
H	-3.262497	1.870034	0.001020
H	-4.376516	-2.952952	-0.000970
H	-5.608910	1.157949	0.001051
H	-6.189732	-1.254860	0.000037
H	3.166278	2.434791	0.000144

SCF Energy: -952.311769756
Sum of electronic and zero-point Energies=
-952.116274
Sum of electronic and thermal Energies=
-952.101963
Sum of electronic and thermal Enthalpies=
-952.101019
Sum of electronic and thermal Free Energies=
-952.158575

==> ./AOX-II/mono/c3c4 <==

O	0.654112	-2.044522	-0.023356
O	-1.702449	0.716585	-0.084536
O	3.277537	1.978765	-0.107897
O	0.594745	1.998565	-0.027563
O	5.312487	-2.293379	-0.035125
C	1.879996	0.014867	-0.069140
C	1.843793	-1.391959	-0.036907
C	-0.529092	-1.406674	-0.060156
C	0.648550	0.726792	-0.056970
C	-0.556146	-0.041098	-0.063561
C	-1.674743	-2.320170	-0.038012
C	3.155836	0.642345	-0.082949
C	2.981246	-2.173572	-0.023163

C	4.210777	-1.520445	-0.044304
C	4.303924	-0.123624	-0.074352
C	-1.590157	-3.508733	0.698767
C	-2.845651	-2.031693	-0.750039
C	-2.666765	-4.382687	0.734845
C	-3.913440	-2.918286	-0.717915
C	-3.829688	-4.090047	0.027582
H	2.916088	-3.253055	0.000317
H	5.273825	0.360039	-0.088924
H	-0.687498	-3.738066	1.252509
H	-2.914671	-1.131673	-1.348513
H	-2.597975	-5.294417	1.317459
H	-4.811943	-2.693526	-1.281449
H	-4.668846	-4.776466	0.055355
H	-2.356944	0.421851	0.564597
H	2.390641	2.381660	-0.094616
H	6.107914	-1.747964	-0.053527
O	-3.166950	3.429841	0.124130
O	-0.487482	4.729910	0.355454
Cu	-1.221010	2.799309	0.079865
H	-3.686765	2.848113	-0.443423
H	-3.223415	4.300774	-0.287550
H	0.364946	4.804220	-0.089214
H	-1.062716	5.362341	-0.090299

SCF Energy: -1303.45784526
Sum of electronic and zero-point Energies= -1303.183261
Sum of electronic and thermal Energies= -1303.160857
Sum of electronic and thermal Enthalpies= -1303.159913
Sum of electronic and thermal Free Energies= -1303.234355

==> ./AOX-II/mono/c3c4/vIP <==

O	0.654112	-2.044522	-0.023356
O	-1.702449	0.716585	-0.084536
O	3.277537	1.978765	-0.107897
O	0.594745	1.998565	-0.027563
O	5.312487	-2.293379	-0.035125
C	1.879996	0.014867	-0.069140
C	1.843793	-1.391959	-0.036907
C	-0.529092	-1.406674	-0.060156
C	0.648550	0.726792	-0.056970
C	-0.556146	-0.041098	-0.063561
C	-1.674743	-2.320170	-0.038012
C	3.155836	0.642345	-0.082949
C	2.981246	-2.173572	-0.023163
C	4.210777	-1.520445	-0.044304
C	4.303924	-0.123624	-0.074352
C	-1.590157	-3.508733	0.698767
C	-2.845651	-2.031693	-0.750039
C	-2.666765	-4.382687	0.734845
C	-3.913440	-2.918286	-0.717915
C	-3.829688	-4.090047	0.027582
H	2.916088	-3.253055	0.000317
H	5.273825	0.360039	-0.088924
H	-0.687498	-3.738066	1.252509
H	-2.914671	-1.131673	-1.348513
H	-2.597975	-5.294417	1.317459
H	-4.811943	-2.693526	-1.281449
H	-4.668846	-4.776466	0.055355
H	-2.356944	0.421851	0.564597
H	2.390641	2.381660	-0.094616
H	6.107914	-1.747964	-0.053527
O	-3.166950	3.429841	0.124130
O	-0.487482	4.729910	0.355454
Cu	-1.221010	2.799309	0.079865
H	-3.686765	2.848113	-0.443423
H	-3.223415	4.300774	-0.287550
H	0.364946	4.804220	-0.089214
H	-1.062716	5.362341	-0.090299

SCF Energy: -1303.22921323

==> ./AOX-II/mono/c3c4/vEA <==

O	0.654112	-2.044522	-0.023356
O	-1.702449	0.716585	-0.084536
O	3.277537	1.978765	-0.107897
O	0.594745	1.998565	-0.027563
O	5.312487	-2.293379	-0.035125
C	1.879996	0.014867	-0.069140
C	1.843793	-1.391959	-0.036907
C	-0.529092	-1.406674	-0.060156
C	0.648550	0.726792	-0.056970
C	-0.556146	-0.041098	-0.063561
C	-1.674743	-2.320170	-0.038012
C	3.155836	0.642345	-0.082949
C	2.981246	-2.173572	-0.023163
C	4.210777	-1.520445	-0.044304
C	4.303924	-0.123624	-0.074352
C	-1.590157	-3.508733	0.698767
C	-2.845651	-2.031693	-0.750039
C	-2.666765	-4.382687	0.734845
C	-3.913440	-2.918286	-0.717915
C	-3.829688	-4.090047	0.027582
H	2.916088	-3.253055	0.000317
H	5.273825	0.360039	-0.088924
H	-0.687498	-3.738066	1.252509
H	-2.914671	-1.131673	-1.348513
H	-2.597975	-5.294417	1.317459
H	-4.811943	-2.693526	-1.281449
H	-4.668846	-4.776466	0.055355
H	-2.356944	0.421851	0.564597

H	2.390641	2.381660	-0.094616
H	6.107914	-1.747964	-0.053527
O	-3.166950	3.429841	0.124130
O	-0.487482	4.729910	0.355454
Cu	-1.221010	2.799309	0.079865
H	-3.686765	2.848113	-0.443423
H	-3.223415	4.300774	-0.287550
H	0.364946	4.804220	-0.089214
H	-1.062716	5.362341	-0.090299

SCF Energy: -1303.61476070

==> ./AOX-II/mono/c3c4/RAF/c3p <==

O	1.674255	1.239806	-0.170757
O	-0.249266	-1.828135	0.078947
O	-2.923517	2.584376	0.068936
O	-2.193887	0.045554	0.078633
O	0.573433	5.771753	-0.111174
C	-0.645386	1.809295	-0.051611
C	0.707052	2.186491	-0.117727
C	1.380676	-0.077082	-0.144969
C	-0.998969	0.421487	0.000230
C	0.102137	-0.527060	-0.020150
C	2.600899	-0.902097	-0.218144
C	-1.610552	2.854636	-0.004421
C	1.127625	3.502877	-0.141114
C	0.150493	4.491605	-0.093142
C	-1.211544	4.176635	-0.028979
C	3.714399	-0.532323	0.504402
C	2.670660	-2.058119	-1.018714
C	4.962299	-1.251815	0.352461
C	3.821724	-2.845378	-1.105574
C	4.937022	-2.506798	-0.384887
H	2.179520	3.750354	-0.193071
H	-1.954977	4.964887	0.008090
H	3.718704	0.343605	1.142279
H	1.802962	-2.349995	-1.599501
H	5.257427	-0.629615	-0.569416
H	3.816887	-3.733409	-1.723271
H	5.846346	-3.095949	-0.419216
H	0.428372	-2.351633	0.521479
H	-3.034812	1.612951	0.096276
H	-0.177687	6.375214	-0.066973
O	-3.184753	-3.895361	0.350078
O	-5.562169	-1.789235	0.101262
Cu	-3.057767	-1.796464	0.113273
H	-2.480007	-4.332262	-0.137958
H	-3.997909	-4.234591	-0.037906
H	-5.920720	-1.163779	-0.534736
H	-5.757291	-2.649803	-0.280583
O	5.861275	-1.065314	1.383160
H	6.734245	-1.370194	1.110928

SCF Energy: -1379.22026006

Sum of electronic and zero-point Energies= -1378.934947

Sum of electronic and thermal Energies= -1378.909062

Sum of electronic and thermal Enthalpies= -1378.908118

Sum of electronic and thermal Free Energies= -1378.990152

==> ./AOX-II/mono/c3c4/RAF/c7 <==

O	-1.445625	1.488371	-0.046527
O	-0.927435	-2.008693	0.660045
O	3.292686	0.838800	0.680286
O	1.540707	-1.091561	0.945186
O	1.550872	4.473331	-1.416635
C	0.906336	1.098833	0.350422
C	-0.173988	1.961197	0.027660
C	-1.741030	0.206051	0.171307
C	0.631610	-0.283124	0.613974
C	-0.735718	-0.703898	0.476473
C	-3.159289	-0.090790	0.018525
C	2.246740	1.635178	0.406237
C	-0.007773	3.280568	-0.217890
C	1.348590	3.908372	-0.145730
C	2.449888	2.954478	0.180950
C	-3.884638	0.559403	-0.992093
C	-3.817380	-0.981877	0.879456
C	-5.234741	0.296188	-1.153797
C	-5.173057	-1.227383	0.716259
C	-5.880947	-0.597543	-0.302467
H	-0.848022	3.915272	-0.471767
H	3.451992	3.367930	0.235324
H	-3.380506	1.251028	-1.656298
H	-3.292580	-1.442707	1.708677
H	-5.786083	0.789349	-1.945965
H	-5.678525	-1.903997	1.395376
H	-6.939091	-0.797176	-0.429242
H	-1.824571	-2.288865	0.433738
H	2.958469	-0.063072	0.860676
H	2.494122	4.621096	-1.558517
O	1.709870	-4.854984	-0.666249
O	3.591604	-2.145536	-1.281829
Cu	1.841079	-2.849006	-0.026163
H	1.452623	-5.435090	0.057415
H	2.585708	-5.160566	-0.923707
H	3.973413	-1.374038	-0.851019
H	4.298791	-2.797918	-1.280709
O	1.235340	4.891413	0.853043
H	2.111203	5.116889	1.190481

SCF Energy: -1379.19094567

Sum of electronic and zero-point Energies= -1378.904257
Sum of electronic and thermal Energies= -1378.878290
Sum of electronic and thermal Enthalpies= -1378.877346
Sum of electronic and thermal Free Energies= -1378.960989

==> ./AOX-II/mono/c3c4/RAF/c8a <==

O	1.807447	-1.102275	-0.024670
O	0.191560	2.114018	-0.607431
O	-2.703077	-2.121764	-0.963274
O	-1.847456	0.392986	-0.901399
O	0.418926	-5.497530	0.175438
C	-0.563587	-1.421537	-0.158123
C	0.711364	-1.818610	0.521270
C	1.660623	0.247959	-0.142939
C	-0.744160	-0.034532	-0.468278
C	0.424224	0.806524	-0.359608
C	2.935013	0.962308	-0.101759
C	-1.497079	-2.387771	-0.473757
C	1.041384	-3.271732	0.475875
C	0.107162	-4.194680	0.099421
C	-1.157339	-3.753328	-0.337688
C	4.067899	0.385447	-0.695970
C	3.057330	2.195130	0.555974
C	5.286040	1.045459	-0.658404
C	4.284588	2.842117	0.599757
C	5.397805	2.274549	-0.012269
H	2.005934	-3.571234	0.870691
H	-1.909109	-4.487298	-0.611768
H	3.983698	-0.570008	-1.199667
H	2.208322	2.630947	1.070758
H	6.152773	0.599206	-1.132677
H	4.372186	3.787344	1.123239
H	6.353651	2.785539	0.020075
H	1.006287	2.593044	-0.799036
H	-2.789912	-1.147887	-1.042487
H	-0.294859	-6.054101	-0.159038
O	-3.367009	3.974689	0.232971
O	-4.522445	0.694155	0.669500
Cu	-2.794877	1.966931	-0.033003
H	-2.636944	4.509355	0.560996
H	-4.053756	4.060920	0.901863
H	-4.203115	-0.214001	0.653098
H	-4.743687	0.855149	1.591821
O	0.550253	-1.486415	1.877676
H	1.306774	-1.824714	2.377713

SCF Energy: -1379.18260528
Sum of electronic and zero-point Energies= -1378.896437
Sum of electronic and thermal Energies= -1378.870538
Sum of electronic and thermal Enthalpies= -1378.869594
Sum of electronic and thermal Free Energies= -1378.952980

==> ./AOX-II/mono/c3c4/RAF/c4p <==

O	1.349502	-1.627261	0.065179
O	0.040741	1.728114	-0.348866
O	-3.420555	-2.025242	-0.198139
O	-2.189613	0.309054	-0.308074
O	-0.642109	-5.830748	0.248056
C	-1.035511	-1.723813	-0.076146
C	0.216374	-2.358003	0.042297
C	1.349649	-0.270817	0.001283
C	-1.107569	-0.304913	-0.184711
C	0.171688	0.421387	-0.165326
C	2.693641	0.269180	0.058872
C	-2.194734	-2.554709	-0.088476
C	0.357591	-3.726852	0.149912
C	-0.800659	-4.500179	0.137543
C	-2.072042	-3.924824	0.017497
C	3.765262	-0.556558	-0.375816
C	2.956318	1.574191	0.553284
C	5.039401	-0.084110	-0.372525
C	4.229209	2.051882	0.593036
C	5.367630	1.198504	0.257003
H	1.335330	-4.179911	0.244013
H	-2.959469	-4.547091	0.011482
H	3.553983	-1.535616	-0.781576
H	2.162413	2.195159	0.945217
H	5.856756	-0.677422	-0.767420
H	4.442743	3.042056	0.977669
H	5.572471	0.824139	1.304384
H	0.864919	2.170667	-0.583456
H	-3.330665	-1.053431	-0.265097
H	-1.496565	-6.278429	0.241766
O	-2.111196	4.367613	-0.292211
O	-4.855602	2.925704	0.551442
Cu	-2.763377	2.279682	-0.100845
H	-1.234874	4.463236	0.093015
H	-2.678428	4.919933	0.254734
H	-5.204267	2.360935	1.247326
H	-4.745665	3.783642	0.973114
O	6.470477	1.903072	-0.226868
H	7.248599	1.336716	-0.181181

SCF Energy: -1379.22422999
Sum of electronic and zero-point Energies= -1378.938592
Sum of electronic and thermal Energies= -1378.912317

Sum of electronic and thermal Enthalpies= -1378.911373
Sum of electronic and thermal Free Energies= -1378.996205

==> ./AOX-II/mono/c3c4/RAF/c2p <==
O 1.873784 0.947450 -0.159186
O -0.418679 -1.850903 -0.468074
O -2.544999 2.798579 -0.268485
O -2.098663 0.200467 -0.438515
O 1.279914 5.550957 0.202542
C -0.366936 1.774761 -0.220829
C 1.019847 1.990774 -0.119280
C 1.450991 -0.331308 -0.301270
C -0.875730 0.447313 -0.349891
C 0.114566 -0.634036 -0.384392
C 2.552839 -1.275502 -0.296532
C -1.213409 2.920978 -0.174131
C 1.579061 3.245975 0.017523
C 0.715584 4.337089 0.062292
C -0.673201 4.183368 -0.033543
C 3.768076 -0.937987 0.490305
C 2.567689 -2.405863 -1.086522
C 4.816428 -1.972494 0.556349
C 3.661955 -3.275057 -1.061638
C 4.786977 -3.071421 -0.243183
H 2.650741 3.373035 0.092281
H -1.325637 5.048257 0.001102
H 4.265363 -0.212297 -0.208249
H 1.757051 -2.624382 -1.769184
H 5.646431 -1.771527 1.224474
H 3.636112 -4.143805 -1.709645
H 5.592173 -3.793899 -0.235831
H 0.220736 -2.559975 -0.330246
H -2.758539 1.848074 -0.360604
H 0.605846 6.240428 0.230407
O -2.750592 -3.125924 1.379297
O -5.499491 -1.779015 0.048564
Cu -3.362764 -1.353952 0.077285
H -1.954066 -3.483561 0.975474
H -3.403574 -3.825819 1.284731
H -5.876109 -1.634252 -0.824577
H -5.632632 -2.717224 0.215704
O 3.466706 -0.327985 1.712163
H 4.204001 0.233011 1.974916

SCF Energy: -1379.22167955
Sum of electronic and zero-point Energies= -1378.936306
Sum of electronic and thermal Energies= -1378.909667
Sum of electronic and thermal Enthalpies= -1378.908722
Sum of electronic and thermal Free Energies= -1378.993958

==> ./AOX-II/mono/c3c4/RAF/c4 <==
O 2.080666 -0.753310 -0.162624
O -0.222902 1.067670 -1.360652
O -2.088419 -3.048866 0.562316
O -1.896108 -0.378443 0.497860
O 1.930191 -5.406482 -0.202700
C -0.071540 -1.807434 0.170033
C 1.306094 -1.901884 -0.071914
C 1.663273 0.460602 -0.056433
C -0.698063 -0.535697 0.240424
C 0.147196 0.724533 -0.052381
C 2.687597 1.455127 -0.036901
C -0.781253 -3.029722 0.312267
C 1.988726 -3.075126 -0.197596
C 1.245622 -4.266199 -0.071488
C -0.119611 -4.244849 0.185132
C 4.039639 1.029495 -0.049634
C 2.405853 2.841376 -0.004353
C 5.059696 1.954932 -0.029172
C 3.439229 3.756436 0.008799
C 4.761885 3.318523 -0.001695
H 3.055173 -3.094352 -0.375759
H -0.675187 -5.169230 0.290203
H 4.272022 -0.026507 -0.068783
H 1.386201 3.196532 0.005956
H 6.090280 1.622502 -0.034479
H 3.216275 4.815912 0.028042
H 5.567946 4.043497 0.011801
H 0.107157 1.946900 -1.591734
H -2.414291 -2.132156 0.642329
H 1.344558 -6.168532 -0.107187
O -3.261672 3.211547 0.700920
O -5.070158 0.962212 -1.253945
Cu -3.311210 1.093552 0.016828
H -2.872887 3.276856 1.578515
H -4.164684 3.523528 0.814089
H -5.529030 0.125173 -1.135901
H -5.715804 1.630156 -1.004009
O -0.100496 1.699922 0.890584
H -1.057493 1.782038 1.014322

SCF Energy: -1379.25111870
Sum of electronic and zero-point Energies= -1378.963091
Sum of electronic and thermal Energies= -1378.937375
Sum of electronic and thermal Enthalpies= -1378.936431
Sum of electronic and thermal Free Energies= -1379.018396

==> ./AOX-II/mono/c3c4/RAF/c3 <==

O	2.007043	0.979170	-0.040923
O	0.044201	-1.335604	-1.199560
O	-2.457812	2.757013	0.306902
O	-1.937072	0.134124	0.356963
O	1.304692	5.577507	-0.139761
C	-0.276173	1.767125	0.129405
C	1.095623	2.027106	-0.003021
C	1.723502	-0.274611	-0.007665
C	-0.745507	0.429927	0.199427
C	0.259913	-0.725001	0.038631
C	2.827024	-1.169982	-0.033654
C	-1.140226	2.894582	0.178204
C	1.639813	3.272984	-0.091304
C	0.750267	4.365491	-0.048150
C	-0.620941	4.180178	0.084657
C	4.140660	-0.641527	0.015196
C	2.644577	-2.570463	-0.115237
C	5.226343	-1.488101	-0.008008
C	3.744201	-3.404324	-0.146565
C	5.029613	-2.868505	-0.089594
H	2.706758	3.420567	-0.187030
H	-1.291456	5.030866	0.117442
H	4.288878	0.427915	0.082280
H	1.654244	-2.996346	-0.162150
H	6.229624	-1.083254	0.038498
H	3.603701	-4.475863	-0.214346
H	5.887635	-3.530969	-0.108646
H	-0.908013	-1.426655	-1.349039
H	-2.681137	1.809147	0.376017
H	0.632178	6.269002	-0.091746
O	-3.477986	-3.658114	0.346783
O	-4.789096	-0.442423	-0.871426
Cu	-3.011105	-1.639075	0.117056
H	-2.861501	-4.223931	-0.128705
H	-4.336456	-3.848411	-0.044998
H	-4.435937	0.441941	-1.007322
H	-5.012853	-0.746892	-1.755644
O	0.159791	-1.583251	1.127863
H	-0.775825	-1.730580	1.332100

SCF Energy: -1379.25218378
Sum of electronic and zero-point Energies= -1378.965162
Sum of electronic and thermal Energies= -1378.938915
Sum of electronic and thermal Enthalpies= -1378.937971
Sum of electronic and thermal Free Energies= -1379.022143

==> ./AOX-II/mono/c3c4/RAF/c5p <==

O	1.242915	-1.765442	-0.096521
O	0.549730	1.800355	-0.066891
O	-3.518033	-1.343626	0.303827
O	-1.924017	0.757459	0.130089
O	-1.426538	-5.583874	0.081209
C	-1.119362	-1.448759	0.095222
C	0.000199	-2.294073	-0.004614
C	1.448326	-0.431289	-0.084886
C	-0.944972	-0.028368	0.068840
C	0.419695	0.457719	-0.035697
C	2.885249	-0.109205	-0.161694
C	-2.396437	-2.071298	0.199481
C	-0.088062	-3.673220	-0.010320
C	-1.354991	-4.237997	0.090733
C	-2.505395	-3.447993	0.198485
C	3.693242	-0.744246	-1.127572
C	3.467876	0.784600	0.705006
C	5.060767	-0.494196	-1.246855
C	4.905071	0.986002	0.694443
C	5.660933	0.410442	-0.408978
H	0.799170	-4.287255	-0.088295
H	-3.483233	-3.908770	0.280267
H	3.230348	-1.449238	-1.808676
H	2.900448	1.291192	1.477623
H	5.631540	-0.984135	-2.024112
H	6.716905	0.646372	-0.479063
H	1.413602	2.075672	-0.394022
H	-3.274748	-0.397207	0.281496
H	-2.343651	-5.872855	0.152227
O	-2.481462	4.672627	-0.487709
O	-5.306167	1.311913	0.132218
Cu	-2.117600	2.717728	-0.140947
H	-2.267506	5.208399	0.283312
H	-3.416778	4.835109	-0.648595
H	-4.427520	1.699236	0.059572
H	-5.899233	2.064408	0.061532
H	5.097576	0.091574	1.387470
O	5.322650	2.163937	1.284630
H	6.268021	2.117878	1.470311

SCF Energy: -1379.22429361
Sum of electronic and zero-point Energies= -1378.939118
Sum of electronic and thermal Energies= -1378.912929
Sum of electronic and thermal Enthalpies= -1378.911984
Sum of electronic and thermal Free Energies= -1378.995923

==> ./AOX-II/mono/c3c4/RAF/c6p <==

O	1.710566	-1.411344	0.183543
O	0.209132	1.864008	-0.086425

O	-3.004933	-2.094947	-0.350363
O	-1.910424	0.314622	-0.302675
O	-0.034197	-5.724757	0.252980
C	-0.651603	-1.649363	-0.083239
C	0.628802	-2.206406	0.105221
C	1.660441	-0.053271	0.086729
C	-0.799137	-0.239569	-0.146226
C	0.426697	0.574023	-0.020559
C	2.982825	0.512190	0.003862
C	-1.758038	-2.547904	-0.168172
C	0.843878	-3.564927	0.219047
C	-0.263908	-4.406158	0.136960
C	-1.558523	-3.907584	-0.059366
C	4.069340	-0.346567	-0.158042
C	3.264454	1.978782	-0.062673
C	5.365360	0.132192	-0.262979
C	4.681913	2.393722	-0.040894
C	5.687958	1.507040	-0.208122
H	1.840157	-3.960448	0.364468
H	-2.404007	-4.582611	-0.122978
H	3.909005	-1.413401	-0.185399
H	2.994639	2.206320	-1.125603
H	6.167686	-0.588590	-0.372803
H	4.862145	3.459850	0.046778
H	6.719859	1.828118	-0.253663
H	0.982480	2.381573	0.254457
H	-2.976563	-1.120155	-0.408019
H	-0.859968	-6.221961	0.204001
O	-3.650589	3.922596	0.072350
O	-5.460419	0.632364	0.287914
Cu	-2.614900	2.179830	-0.120696
H	-3.345985	4.426204	0.834544
H	-4.574139	3.724846	0.261791
H	-4.505103	0.518822	0.317079
H	-5.568537	1.551130	0.025877
O	2.453347	2.728388	0.810973
H	2.607819	3.670587	0.664009

SCF Energy: -1379.23192982
Sum of electronic and zero-point Energies= -1378.944213
Sum of electronic and thermal Energies= -1378.918913
Sum of electronic and thermal Enthalpies= -1378.917969
Sum of electronic and thermal Free Energies= -1379.000150

==> ./AOX-II/mono/c3c4/RAF/c1p <==

O	-1.549451	1.547959	0.158073
O	-0.425626	-1.902233	-0.091431
O	3.228015	1.706516	-0.195894
O	1.886266	-0.568402	-0.179569
O	0.646601	5.659377	0.162004
C	0.832265	1.522975	-0.024842
C	-0.379644	2.225705	0.091867
C	-1.591119	0.206174	0.131790
C	0.825411	0.094180	-0.075280
C	-0.475580	-0.559953	-0.004022
C	-2.983912	-0.327651	0.189752
C	2.028422	2.294585	-0.081779
C	-0.456773	3.603334	0.154183
C	0.736297	4.317161	0.098136
C	1.973757	3.672668	-0.018500
C	-3.952123	-0.012166	-0.889520
C	-3.241677	-1.528912	0.991233
C	-5.059108	-0.926263	-1.140996
C	-4.321528	-2.292660	0.736865
C	-5.235882	-1.999343	-0.349465
H	-1.410902	4.104462	0.247213
H	2.891187	4.248519	-0.057881
H	-3.642692	0.670066	-1.669714
H	-2.537232	-1.775229	1.775551
H	-5.680632	-0.732999	-2.005266
H	-4.501929	-3.175566	1.337944
H	-6.046366	-2.691183	-0.542519
H	-1.293788	-2.316030	-0.159151
H	3.092718	0.738765	-0.230523
H	1.521296	6.062223	0.109698
O	3.012469	-4.412429	0.137158
O	5.383371	-0.852644	-0.210060
Cu	2.329960	-2.516977	-0.030201
H	3.171184	-4.634039	1.060774
H	3.869660	-4.512454	-0.290186
H	4.487760	-1.200932	-0.270517
H	5.279735	-0.037016	0.288058
O	-3.966578	0.813616	0.395653
H	-4.682243	0.570647	1.018357

SCF Energy: -1379.18869155
Sum of electronic and zero-point Energies= -1378.900642
Sum of electronic and thermal Energies= -1378.875179
Sum of electronic and thermal Enthalpies= -1378.874235
Sum of electronic and thermal Free Energies= -1378.957680

==> ./AOX-II/mono/c3c4/RAF/c6 <==

O	-1.640065	-1.498998	0.015929
O	-0.869063	2.060007	0.005961
O	3.052804	-1.216881	-0.286532
O	1.583650	0.922815	-0.162735
O	0.916793	-5.370062	0.136636
C	0.690829	-1.237780	-0.180659

C	-0.448332	-2.060691	-0.058472
C	-1.840348	-0.138880	-0.016703
C	0.567340	0.197766	-0.130396
C	-0.781386	0.719766	-0.044051
C	-3.263185	0.202627	0.026276
C	1.938556	-1.855806	-0.273725
C	-0.416295	-3.459377	0.042621
C	0.793382	-4.081400	-0.050849
C	2.026454	-3.335708	-0.473692
C	-4.125037	-0.511618	0.868729
C	-3.779798	1.220026	-0.785732
C	-5.475412	-0.197257	0.908802
C	-5.134397	1.523892	-0.743512
C	-5.982766	0.820482	0.104970
H	-1.324128	-4.009680	0.247922
H	1.992611	-3.416893	-1.578572
H	-3.732875	-1.302805	1.497136
H	-3.131907	1.755247	-1.471794
H	-6.134564	-0.748313	1.570117
H	-5.527122	2.305761	-1.383646
H	-7.039715	1.060864	0.136801
H	-1.759551	2.355419	0.231312
H	2.857011	-0.243225	-0.240603
H	1.857416	-5.618676	0.111514
O	2.014312	4.987497	-0.235646
O	4.146624	2.206900	1.243586
Cu	1.959649	2.939344	-0.003831
H	1.547252	5.261146	-1.031461
H	2.916266	5.298816	-0.362837
H	4.232099	1.310685	0.906295
H	4.934985	2.653464	0.922732
O	3.170620	-3.939705	0.036691
H	3.859366	-3.951614	-0.636585

SCF Energy: -1379.25446399
Sum of electronic and zero-point Energies= -1378.967029
Sum of electronic and thermal Energies= -1378.940976
Sum of electronic and thermal Enthalpies= -1378.940032
Sum of electronic and thermal Free Energies= -1379.023603

==> ./AOX-II/mono/c3c4/RAF/c8 <==

O	-1.937289	-0.636665	-0.124123
O	0.300081	2.214075	-0.030825
O	2.411606	-2.440922	0.037904
O	2.009891	0.131387	0.019523
O	-1.321087	-5.251331	0.167013
C	0.262133	-1.429703	-0.070261
C	-1.089120	-1.630702	-0.106505
C	-1.538740	0.669861	-0.122660
C	0.778111	-0.085346	-0.041894
C	-0.206055	0.974159	-0.075414
C	-2.671261	1.597094	-0.130744
C	1.124304	-2.592641	-0.002722
C	-1.721120	-2.979595	-0.221473
C	-0.740098	-4.092006	0.034768
C	0.614325	-3.892972	0.053799
C	-3.785182	1.329777	0.675371
C	-2.666055	2.734095	-0.946959
C	-4.865831	2.199129	0.676136
C	-3.754404	3.597548	-0.942034
C	-4.852011	3.335041	-0.129407
H	1.298772	-4.724941	0.163399
H	-3.795995	0.448376	1.306252
H	-1.830594	2.928991	-1.611354
H	-5.721216	1.989921	1.308601
H	-3.746683	4.471017	-1.584070
H	-5.699400	4.011716	-0.126893
H	-0.386281	2.884417	0.074648
H	2.633364	-1.476279	0.030226
H	-0.679392	-5.967682	0.290404
O	4.522166	3.170356	0.798236
O	4.819760	-0.768032	-1.376082
Cu	3.132805	1.751944	0.378096
H	4.802594	3.637470	0.004200
H	5.326007	2.768282	1.143520
H	4.220144	-0.068170	-1.098523
H	5.465781	-0.318011	-1.926659
H	-1.957057	-3.078182	-1.296286
O	-2.879205	-3.057245	0.553423
H	-3.522763	-3.608712	0.097201

SCF Energy: -1379.26197469
Sum of electronic and zero-point Energies= -1378.971987
Sum of electronic and thermal Energies= -1378.946632
Sum of electronic and thermal Enthalpies= -1378.945688
Sum of electronic and thermal Free Energies= -1379.027582

==> ./AOX-II/mono/c3c4/RAF/c2 <==

O	1.786666	-0.533503	1.526313
O	-0.447128	2.118697	1.001539
O	-1.534749	-2.151941	-1.587389
O	-1.423594	0.420961	-0.864848
O	1.563308	-5.052225	0.468248
C	0.071552	-1.239405	-0.043379
C	1.169953	-1.514558	0.820400
C	1.584492	0.841598	1.243318
C	-0.463193	0.069024	-0.145146
C	0.183033	1.062454	0.732683

C 2.523190 1.339970 0.137283
C -0.508475 -2.337627 -0.756295
C 1.654385 -2.785975 0.987105
C 1.049857 -3.834198 0.275944
C -0.021377 -3.618072 -0.593227
C 3.306204 0.452860 -0.603087
C 2.597887 2.713747 -0.114870
C 4.175805 0.944014 -1.568405
C 3.466883 3.195062 -1.084012
C 4.255290 2.311102 -1.813519
H 2.493609 -2.977524 1.642183
H -0.467490 -4.441149 -1.138262
H 3.267174 -0.613146 -0.420755
H 1.988927 3.410335 0.451932
H 4.794180 0.252236 -2.128892
H 3.524800 4.261523 -1.268827
H 4.931578 2.687434 -2.572969
H 0.038569 2.690991 1.636566
H -1.773211 -1.205761 -1.609021
H 1.104651 -5.709888 -0.070520
O -4.810756 2.363436 0.307706
O -4.315540 -0.943867 -0.397618
Cu -3.245857 1.035607 -0.227521
H -4.557369 2.940556 1.034955
H -5.558580 1.860162 0.645392
H -3.706316 -1.685468 -0.324805
H -4.937512 -1.066116 0.325856
O 1.694524 1.547630 2.418471
H 2.612074 1.809722 2.567123

SCF Energy: -1379.24104565
Sum of electronic and zero-point Energies= -1378.952482
Sum of electronic and thermal Energies= -1378.927333
Sum of electronic and thermal Enthalpies= -1378.926389
Sum of electronic and thermal Free Energies= -1379.007679

==> ./AOX-II/mono/c3c4/RAF/c5 <==
O -1.550344 -1.504465 0.012552
O -0.882093 1.983098 -0.640964
O 2.857185 -1.525433 -1.484048
O 1.554168 0.917312 -0.961253
O 1.023935 -5.365046 0.293064
C 0.781136 -1.203870 -0.321316
C -0.305659 -1.998364 -0.042631
C -1.794073 -0.195271 -0.183165
C 0.602459 0.177924 -0.610341
C -0.752999 0.666963 -0.470971
C -3.205453 0.150602 -0.023942
C 2.164903 -1.817808 -0.310145
C -0.194352 -3.377069 0.177198
C 1.047648 -4.037328 0.097886
C 2.171146 -3.310068 -0.168750
C -3.946215 -0.462743 0.996585
C -3.836488 1.060632 -0.883087
C -5.285963 -0.150203 1.165986
C -5.180528 1.362157 -0.708678
C -5.905125 0.763863 0.316809
H -1.090375 -3.945478 0.401816
H 3.145784 -3.784670 -0.223560
H -3.464476 -1.170038 1.661410
H -3.297377 1.502617 -1.713892
H -5.849095 -0.620428 1.964065
H -5.663490 2.058440 -1.384570
H -6.953845 1.004391 0.451326
H -1.769096 2.297142 -0.421775
H 2.758470 -0.572991 -1.649703
H 1.911911 -5.737193 0.223227
O 2.232372 4.686450 0.486233
O 3.011547 1.503683 1.708454
Cu 1.995509 2.664812 -0.008412
H 2.258302 5.237920 -0.302247
H 3.080616 4.834108 0.917062
H 2.975783 0.591470 1.382368
H 3.949847 1.702833 1.773150
O 2.793899 -1.287244 0.838082
H 3.649291 -1.721012 0.959680

SCF Energy: -1379.20161098
Sum of electronic and zero-point Energies= -1378.914837
Sum of electronic and thermal Energies= -1378.889209
Sum of electronic and thermal Enthalpies= -1378.888265
Sum of electronic and thermal Free Energies= -1378.970485

==> ./AOX-II/mono/c3c4/RAF/c4a <==
O -1.980622 1.180176 0.056310
O -0.313838 -2.048726 -0.225705
O 2.715066 2.079351 0.683350
O 1.758843 -0.349278 0.223464
O -0.204897 5.317018 -0.998801
C 0.344524 1.532146 0.636339
C -0.935047 2.022420 0.028371
C -1.811013 -0.173575 0.028751
C 0.599766 0.076402 0.202860
C -0.558973 -0.728900 -0.030665
C -3.085405 -0.883548 -0.001648
C 1.514250 2.469420 0.350568
C -1.098128 3.256711 -0.466696
C 0.035809 4.130998 -0.516151

C	1.319888	3.734125	-0.124795
C	-4.193366	-0.296610	-0.630832
C	-3.229467	-2.131012	0.623829
C	-5.412082	-0.956972	-0.651562
C	-4.456934	-2.777027	0.609463
C	-5.546977	-2.196853	-0.032504
H	-2.049263	3.590777	-0.858365
H	2.158490	4.410939	-0.232153
H	-4.092058	0.667884	-1.112894
H	-2.396560	-2.582554	1.148578
H	-6.260000	-0.501820	-1.150521
H	-4.563229	-3.733745	1.107830
H	-6.502853	-2.708643	-0.046303
H	-1.026717	-2.463051	-0.725759
H	2.741269	1.104882	0.783649
H	0.593551	5.867744	-1.031555
O	3.527603	-3.927158	-0.690523
O	4.963176	-0.828292	-0.246498
Cu	2.662268	-2.109043	-0.244258
H	3.050015	-4.664854	-0.298240
H	4.412340	-3.979390	-0.314793
H	4.711847	0.060021	0.021682
H	5.545791	-1.126780	0.457402
O	0.120115	1.531810	2.035477
H	0.866962	1.115259	2.486371

SCF Energy: -1379.21887808
Sum of electronic and zero-point Energies= -1378.931405
Sum of electronic and thermal Energies= -1378.905325
Sum of electronic and thermal Enthalpies= -1378.904380
Sum of electronic and thermal Free Energies= -1378.987815

==> ./AOX-II/mono/c3c4/HAT/c3 <==

O	-1.352289	-1.614928	0.107472
O	1.512177	0.273615	0.688542
O	-2.714146	2.982110	-0.317448
O	-0.104929	2.227549	-0.226239
O	-5.874197	-0.518542	-0.018293
C	-1.938613	0.730579	-0.064762
C	-2.311637	-0.618996	0.057291
C	-0.065519	-1.406272	0.131365
C	-0.563539	1.092949	-0.056617
C	0.399509	-0.013207	0.258488
C	0.773313	-2.551740	0.061491
C	-2.983314	1.686321	-0.186810
C	-3.607907	-1.048038	0.071215
C	-4.616895	-0.067863	-0.036683
C	-4.312064	1.282282	-0.163370
C	0.197623	-3.838795	0.197167
C	2.163248	-2.432063	-0.181450
C	0.993386	-4.959950	0.122916
C	2.942710	-3.567611	-0.268202
C	2.365121	-4.826598	-0.109548
H	-3.855501	-2.098243	0.145338
H	-5.100960	2.020023	-0.249130
H	-0.863827	-3.935782	0.383335
H	2.615185	-1.461406	-0.326799
H	0.555991	-5.943175	0.244464
H	4.003489	-3.477402	-0.466582
H	2.985923	-5.713045	-0.172738
H	-1.746701	3.116488	-0.340755
O	4.107625	1.368166	-0.959369
O	2.854788	4.077029	0.389474
Cu	2.355675	2.034791	0.147336
H	4.426654	0.547487	-0.570040
H	4.813098	2.003625	-0.800353
H	2.499441	4.439725	1.207188
H	3.809934	4.157614	0.479788
H	-6.502064	0.209173	-0.114540

SCF Energy: -1302.81166463
Sum of electronic and zero-point Energies= -1302.551896
Sum of electronic and thermal Energies= -1302.529739
Sum of electronic and thermal Enthalpies= -1302.528794
Sum of electronic and thermal Free Energies= -1302.602679

==> ./AOX-II/mono/c3c4/HAT/c7 <==

O	1.207322	-1.840990	0.036617
O	0.560561	1.642684	0.602947
O	-3.568496	-1.244415	0.336551
O	-1.879000	0.761238	0.579396
O	-1.670353	-5.476992	-0.323540
C	-1.173764	-1.465245	0.220742
C	-0.065786	-2.327646	0.045357
C	1.480977	-0.557111	0.198231
C	-0.956424	-0.082605	0.418166
C	0.425611	0.346145	0.414973
C	2.884120	-0.228161	0.085588
C	-2.516167	-2.044510	0.185565
C	-0.207560	-3.661677	-0.155315
C	-1.563346	-4.258765	-0.165599
C	-2.690153	-3.380425	0.003367
C	3.667019	-0.943508	-0.840413
C	3.480613	0.767453	0.881633
C	5.006044	-0.636515	-0.991788
C	4.827432	1.051226	0.731412
C	5.587401	0.361604	-0.210265
H	0.649186	-4.300621	-0.327403

H -3.685524 -3.805178 -0.013051
H 3.207282 -1.708153 -1.454229
H 2.920645 1.265237 1.665412
H 5.601019 -1.172090 -1.721888
H 5.289212 1.802520 1.360580
H 6.638984 0.595978 -0.330051
H 1.450142 1.988343 0.426853
H -3.263505 -0.319902 0.456437
O -0.646081 4.610112 -0.533967
O -3.533894 3.109597 -0.767340
Cu -1.464587 2.723479 -0.116909
H 0.033181 4.825596 0.113923
H -1.324545 5.282777 -0.412002
H -4.102643 2.470128 -0.325932
H -3.816990 3.963043 -0.423518

SCF Energy: -1302.75979842
Sum of electronic and zero-point Energies= -1302.500881
Sum of electronic and thermal Energies= -1302.477071
Sum of electronic and thermal Enthalpies= -1302.476127
Sum of electronic and thermal Free Energies= -1302.554735

==> ./AOX-II/mono/c3c4/HAT/c5 <==
O 1.217640 1.734181 0.054001
O -1.733125 -0.220024 0.621907
O 2.762165 -2.739477 0.542856
O 0.011653 -2.119182 0.445939
O 5.727120 0.819181 -0.397823
C 1.823746 -0.578869 0.265452
C 2.149835 0.760437 0.085149
C -0.078119 1.479769 0.215372
C 0.454799 -0.955248 0.384688
C -0.481701 0.165375 0.417854
C -0.932815 2.650847 0.091846
C 2.944982 -1.555068 0.278174
C 3.446182 1.201341 -0.144421
C 4.526035 0.277534 -0.174814
C 4.285057 -1.055038 0.004842
C -0.624960 3.602122 -0.893982
C -2.039756 2.851044 0.931832
C -1.430174 4.716718 -1.053502
C -2.831402 3.978490 0.769342
C -2.535187 4.905368 -0.225644
H 3.625967 2.252282 -0.337012
H 5.092648 -1.777559 -0.002270
H 0.228064 3.446723 -1.543484
H -2.251299 2.166818 1.746264
H -1.199614 5.439221 -1.827641
H -3.674821 4.138689 1.430535
H -3.162495 5.780414 -0.352828
H -2.393137 0.473472 0.463914
O -4.130079 -2.331168 -0.359746
O -1.753592 -4.328260 -1.006335
Cu -2.038287 -2.362959 -0.095881
H -4.547655 -1.851728 0.363484
H -4.462853 -3.231601 -0.281425
H -0.961625 -4.720977 -0.624612
H -2.467947 -4.919022 -0.746344
H 6.411698 0.138116 -0.427575

SCF Energy: -1302.76209960
Sum of electronic and zero-point Energies= -1302.503456
Sum of electronic and thermal Energies= -1302.480267
Sum of electronic and thermal Enthalpies= -1302.479323
Sum of electronic and thermal Free Energies= -1302.555946

==> ./AOX-II/mono/c3c4/HAT/h2o <==
O -0.648763 -1.999283 0.088878
O 1.576468 0.787957 0.551393
O -3.336975 1.978511 0.140145
O -0.626786 2.038468 0.146942
O -5.285949 -2.310515 -0.165842
C -1.914327 0.046188 0.159960
C -1.852251 -1.357778 0.086384
C 0.515039 -1.369921 0.194898
C -0.707858 0.780935 0.221759
C 0.507159 0.011552 0.330909
C 1.681811 -2.225942 0.104357
C -3.201551 0.658387 0.088260
C -2.967990 -2.148967 -0.031787
C -4.222126 -1.510773 -0.069729
C -4.339630 -0.123322 -0.021331
C 1.661620 -3.294003 -0.810039
C 2.818627 -2.010868 0.902908
C 2.773270 -4.105271 -0.944082
C 3.919755 -2.841277 0.768501
C 3.903708 -3.879406 -0.158780
H -2.886071 -3.223845 -0.120270
H -5.313501 0.349329 -0.061980
H 0.786944 -3.455882 -1.428208
H 2.820399 -1.248396 1.674305
H 2.764311 -4.914418 -1.664846
H 4.786645 -2.686706 1.399627
H 4.770867 -4.521187 -0.265110
H 2.436277 0.363237 0.372382
H -2.460845 2.405268 0.185908
O 3.049501 3.186050 -0.543870
O 0.772369 4.696099 -0.372044

Cu	1.303358	2.739262	-0.115679
H	3.555373	2.371592	-0.434533
H	0.074703	4.884994	0.269091
H	1.521411	5.239074	-0.094614
H	-6.104337	-1.797147	-0.174988

SCF Energy: -1302.73667181
Sum of electronic and zero-point Energies= -1302.473871
Sum of electronic and thermal Energies= -1302.452264
Sum of electronic and thermal Enthalpies= -1302.451320
Sum of electronic and thermal Free Energies= -1302.523855

==> ./AOX-II/mono/c3c4/aIP <==

O	1.966580	1.003556	-0.044789
O	-0.210440	-1.846869	0.058584
O	-2.493417	2.711488	-0.099636
O	-1.989416	0.112780	-0.072045
O	1.229465	5.586618	-0.002730
C	-0.301532	1.746202	-0.072828
C	1.070233	2.011700	-0.043022
C	1.619808	-0.275829	-0.052856
C	-0.759502	0.407031	-0.070300
C	0.261431	-0.618235	-0.028966
C	2.743148	-1.197551	-0.038626
C	-1.193118	2.880586	-0.073889
C	1.586881	3.292843	-0.020345
C	0.673962	4.387094	-0.026302
C	-0.694425	4.179815	-0.051733
C	3.878487	-0.874403	0.721675
C	2.725889	-2.378019	-0.798305
C	4.961887	-1.736030	0.742857
C	3.823478	-3.224748	-0.780368
C	4.936287	-2.911365	-0.005437
H	2.654524	3.465077	0.005296
H	-1.383304	5.015431	-0.052884
H	3.892553	0.036292	1.308044
H	1.887720	-2.610954	-1.446026
H	5.829594	-1.492407	1.344640
H	3.813207	-4.125521	-1.382586
H	5.788769	-3.581047	0.010840
H	0.458283	-2.530304	0.213880
H	-2.711732	1.754640	-0.107219
H	0.559338	6.285541	-0.003527
O	-3.316112	-3.632664	0.336301
O	-4.732341	-0.981339	0.101520
Cu	-2.738408	-1.733039	0.085267
H	-2.739933	-4.222573	-0.165608
H	-4.194017	-3.743732	-0.050548
H	-4.758334	-0.164045	-0.409156
H	-5.314891	-1.591353	-0.364858

SCF Energy: -1303.23857292
Sum of electronic and zero-point Energies= -1302.964598
Sum of electronic and thermal Energies= -1302.942215
Sum of electronic and thermal Enthalpies= -1302.941271
Sum of electronic and thermal Free Energies= -1303.014876

==> ./AOX-II/mono/c3c4/aEA <==

O	1.891516	1.255646	-0.066882
O	0.348256	-2.022897	0.170464
O	-2.840906	2.012761	0.039140
O	-1.801544	-0.404340	0.125087
O	0.226748	5.614510	-0.186988
C	-0.480526	1.532262	-0.004327
C	0.814736	2.073597	-0.058940
C	1.773450	-0.090990	-0.022723
C	-0.653527	0.111776	0.058973
C	0.547829	-0.688049	0.061078
C	3.077055	-0.768598	-0.022215
C	-1.570701	2.446924	-0.008115
C	1.065269	3.433566	-0.116802
C	-0.028880	4.290861	-0.121482
C	-1.341411	3.807312	-0.064727
C	4.116603	-0.258009	0.765118
C	3.305290	-1.904078	-0.808653
C	5.354559	-0.884427	0.777421
C	4.549650	-2.523145	-0.794515
C	5.573510	-2.018896	0.000067
H	2.078218	3.810724	-0.163529
H	-2.180870	4.493377	-0.069107
H	3.947779	0.623492	1.372983
H	2.523642	-2.288104	-1.455683
H	6.150626	-0.486751	1.397107
H	4.719695	-3.396343	-1.414315
H	6.542670	-2.505410	0.010631
H	1.180298	-2.487705	0.312483
H	-2.825080	1.034732	0.075967
H	-0.597378	6.115205	-0.202381
O	-3.587195	-3.965276	0.042204
O	-5.312090	-0.227380	0.105753
Cu	-2.488617	-2.271151	0.051388
H	-3.322594	-4.555210	-0.671370
H	-4.511614	-3.761707	-0.134699
H	-4.484855	-0.698048	0.252574
H	-5.066364	0.500318	-0.472090

SCF Energy: -1303.63611536

Sum of electronic and zero-point Energies= -1303.364719
Sum of electronic and thermal Energies= -1303.342086
Sum of electronic and thermal Enthalpies= -1303.341141
Sum of electronic and thermal Free Energies= -1303.416993

==> ./AOX-II/mono/c4c5/aEA <==

O	1.951855	1.151797	0.045339
O	1.010398	-2.287084	-0.430558
O	-2.769116	1.225934	-0.645011
O	-1.312925	-1.149343	-0.536374
O	-0.305524	5.213558	0.232368
C	-0.430013	1.082259	-0.269259
C	0.765912	1.790465	-0.028035
C	2.078862	-0.189247	-0.109518
C	-0.358478	-0.349044	-0.384041
C	0.963375	-0.942708	-0.316348
C	3.465828	-0.644594	0.014090
C	-1.616509	1.862377	-0.367516
C	0.819640	3.165243	0.143105
C	-0.366635	3.876962	0.061836
C	-1.581572	3.232409	-0.198973
C	4.378953	0.112514	0.762454
C	3.912207	-1.813512	-0.617087
C	5.699047	-0.297027	0.885010
C	5.237103	-2.212201	-0.496436
C	6.133755	-1.460330	0.256153
H	1.763368	3.660308	0.330103
H	-2.496787	3.810320	-0.279066
H	4.050840	1.018983	1.256452
H	3.231232	-2.401825	-1.216884
H	6.390722	0.295283	1.474060
H	5.570156	-3.113638	-0.999053
H	7.166743	-1.777296	0.350159
H	0.084153	-2.568702	-0.528498
H	-1.184945	5.600307	0.152858
Cu	-3.237833	-1.221883	0.118168
O	-5.257666	-0.979496	0.628027
H	-5.765906	-1.743067	0.335710
O	-3.588735	-3.765861	0.525178
H	-4.477692	-3.781997	0.159022
H	-3.731459	-3.740315	1.475582
H	-5.636552	-0.235034	0.150088
H	-3.501868	1.853865	-0.651767

SCF Energy: -1303.62802089
Sum of electronic and zero-point Energies= -1303.358592
Sum of electronic and thermal Energies= -1303.335012
Sum of electronic and thermal Enthalpies= -1303.334067
Sum of electronic and thermal Free Energies= -1303.412649

==> ./AOX-II/mono/c4c5/aIP <==

O	1.897362	1.164071	-0.055759
O	1.125512	-2.279469	-0.155812
O	-2.884573	0.963146	-0.181254
O	-1.258412	-1.346931	-0.195587
O	-0.548671	5.091043	0.022378
C	-0.514256	0.952359	-0.119194
C	0.660962	1.738803	-0.062751
C	2.121058	-0.136210	-0.096340
C	-0.379220	-0.455771	-0.161610
C	0.992981	-0.989669	-0.153061
C	3.500837	-0.525645	-0.021541
C	-1.750547	1.670950	-0.124464
C	0.659141	3.106606	-0.012719
C	-0.582083	3.765350	-0.023353
C	-1.779494	3.048600	-0.079514
C	4.441722	0.410342	0.464665
C	3.943233	-1.804460	-0.425086
C	5.773538	0.065366	0.564648
C	5.283954	-2.127628	-0.336878
C	6.198896	-1.202786	0.163817
H	1.584941	3.663459	0.032896
H	-2.729234	3.572022	-0.085646
H	4.110374	1.389348	0.784989
H	3.249951	-2.524787	-0.831658
H	6.486877	0.779078	0.958536
H	5.620488	-3.104551	-0.662186
H	7.246879	-1.469083	0.241304
H	0.232504	-2.682206	-0.178279
H	-1.439257	5.466241	0.005400
Cu	-3.213810	-1.238241	0.046953
O	-5.178189	-1.068568	0.313240
H	-5.620446	-1.864324	-0.007597
O	-3.208343	-3.323639	0.278973
H	-4.009647	-3.662342	-0.138380
H	-3.318987	-3.529285	1.214831
H	-5.525594	-0.345296	-0.223884
H	-3.670184	1.526247	-0.141103

SCF Energy: -1303.24277751
Sum of electronic and zero-point Energies= -1302.966592
Sum of electronic and thermal Energies= -1302.944640
Sum of electronic and thermal Enthalpies= -1302.943695
Sum of electronic and thermal Free Energies= -1303.016234

==> ./AOX-II/mono/c4c5/HAT/c3 <==

O	1.716697	1.253114	0.143108
O	0.804831	-2.091911	0.109937
O	-2.949592	1.228917	-0.905876
O	-1.399831	-1.022730	-1.096214
O	-0.717780	5.185567	0.543075
C	-0.654001	1.135433	-0.377647
C	0.473582	1.859966	0.051376
C	1.944216	-0.011801	-0.005524
C	-0.540753	-0.272187	-0.618898
C	0.749443	-0.916173	-0.164726
C	3.296308	-0.434622	0.014099
C	-1.861095	1.879652	-0.496727
C	0.475878	3.193102	0.352077
C	-0.742679	3.884604	0.238133
C	-1.897185	3.232860	-0.177999
C	4.303545	0.493499	0.384155
C	3.664177	-1.749311	-0.365501
C	5.623116	0.105712	0.399249
C	4.994413	-2.115227	-0.358976
C	5.969975	-1.197080	0.027201
H	1.381831	3.698699	0.657493
H	-2.830729	3.778773	-0.264061
H	4.026927	1.498321	0.674852
H	2.915997	-2.458796	-0.685095
H	6.391380	0.807977	0.697650
H	5.278191	-3.115688	-0.660500
H	7.012239	-1.494931	0.034301
H	-1.589583	5.584703	0.429072
Cu	-2.982306	-1.683735	0.003156
O	-4.898416	-1.697457	0.873023
H	-5.024482	-2.508783	1.375468
O	-1.788876	-3.465820	0.688114
H	-1.816161	-3.557881	1.644878
H	-0.893996	-3.150603	0.496751
H	-5.582566	-1.723922	0.196092
H	-3.712076	1.822423	-0.931502

SCF Energy: -1302.80391439
Sum of electronic and zero-point Energies= -1302.543872
Sum of electronic and thermal Energies= -1302.520018
Sum of electronic and thermal Enthalpies= -1302.519074
Sum of electronic and thermal Free Energies= -1302.598725

==> ./AOX-II/mono/c4c5/HAT/h2o <==

O	1.746139	1.210178	-0.107566
O	1.222770	-2.282022	-0.479385
O	-2.921655	0.660907	-0.662310
O	-1.253976	-1.431125	-0.655004
O	-1.007999	4.946025	0.219256
C	-0.600185	0.859250	-0.368356
C	0.490833	1.709622	-0.150320
C	2.044918	-0.077554	-0.204584
C	-0.375686	-0.542645	-0.476733
C	0.991351	-0.995851	-0.391863
C	3.442543	-0.380120	-0.006385
C	-1.879520	1.466201	-0.389404
C	0.366642	3.072801	0.037885
C	-0.917633	3.624556	0.032719
C	-2.044218	2.819737	-0.159982
C	4.252957	0.569883	0.649728
C	4.021108	-1.581625	-0.460549
C	5.595094	0.316970	0.854227
C	5.370175	-1.816506	-0.259537
C	6.157828	-0.876322	0.400564
H	1.239840	3.689258	0.205145
H	-3.036738	3.256536	-0.170746
H	3.815890	1.491471	1.011641
H	3.427749	-2.312250	-0.989584
H	6.207327	1.046247	1.371340
H	5.811768	-2.736759	-0.622887
H	7.211900	-1.071989	0.562224
H	0.364003	-2.742284	-0.545311
H	-1.928389	5.237719	0.212977
Cu	-3.055167	-1.297422	0.180875
O	-4.769543	-0.949963	0.806264
H	-5.115342	-1.770521	1.172817
O	-3.050542	-3.335644	0.470572
H	-3.954845	-3.642145	0.324026
H	-2.889926	-3.496964	1.409293
H	-3.772200	1.119693	-0.565379

SCF Energy: -1302.74498148
Sum of electronic and zero-point Energies= -1302.482504
Sum of electronic and thermal Energies= -1302.460659
Sum of electronic and thermal Enthalpies= -1302.459715
Sum of electronic and thermal Free Energies= -1302.532549

==> ./AOX-II/mono/c4c5/HAT/c7 <==

O	1.844670	1.203191	0.006037
O	1.180817	-2.203654	0.609271
O	-2.848626	0.861626	0.775867
O	-1.225910	-1.348041	0.711952
O	-0.825801	4.991540	-0.331991
C	-0.533254	0.937422	0.350982
C	0.591635	1.740735	0.073605
C	2.118088	-0.067750	0.166361

C	-0.362782	-0.460492	0.501107
C	1.006596	-0.940591	0.431864
C	3.491793	-0.429218	-0.010234
C	-1.805552	1.628884	0.430844
C	0.523038	3.082656	-0.156010
C	-0.771704	3.766248	-0.122121
C	-1.919833	2.963501	0.175042
C	4.384601	0.551897	-0.508878
C	3.990555	-1.714916	0.305284
C	5.716703	0.252121	-0.688307
C	5.330173	-1.996950	0.124758
C	6.193584	-1.022475	-0.372637
H	1.417862	3.650303	-0.378201
H	-2.888047	3.446313	0.240413
H	4.015533	1.536832	-0.760918
H	3.341198	-2.478364	0.702953
H	6.391499	1.004831	-1.076969
H	5.707253	-2.980971	0.374526
H	7.242743	-1.254784	-0.515548
Cu	-3.155273	-1.284280	-0.120056
O	-5.161802	-0.812608	-0.611315
H	-5.492940	-1.411239	-1.288990
O	-3.048887	-3.351745	-0.831366
H	-3.938085	-3.661350	-1.031742
H	-2.755255	-3.909951	-0.104029
H	-5.192211	0.059925	-1.017834
H	-3.670600	1.372988	0.761125
H	0.286432	-2.599321	0.739217

SCF Energy: -1302.76452637
Sum of electronic and zero-point Energies= -1302.505298
Sum of electronic and thermal Energies= -1302.481535
Sum of electronic and thermal Enthalpies= -1302.480591
Sum of electronic and thermal Free Energies= -1302.559342

==> ./AOX-II/mono/c4c5/HAT/c5 <==

O	1.801479	1.190438	-0.042938
O	1.372639	-2.329701	-0.320976
O	-2.835852	0.590323	-0.781904
O	-1.139584	-1.556842	-0.441660
O	-1.042467	4.862668	0.148206
C	-0.525516	0.753125	-0.324048
C	0.532350	1.631704	-0.121443
C	2.128572	-0.097081	-0.127624
C	-0.282400	-0.646216	-0.342762
C	1.105483	-1.044189	-0.266142
C	3.544610	-0.363920	0.025789
C	-1.858962	1.311779	-0.431154
C	0.353000	3.000882	0.032100
C	-0.949539	3.535515	-0.009493
C	-2.040427	2.706274	-0.190403
C	4.341669	0.555868	0.731627
C	4.142805	-1.502863	-0.540964
C	5.698617	0.330647	0.877602
C	5.505882	-1.708307	-0.401505
C	6.284482	-0.800111	0.310600
H	1.206433	3.643203	0.209738
H	-3.044074	3.111259	-0.245928
H	3.887904	1.431428	1.178721
H	3.553942	-2.206223	-1.112098
H	6.303309	1.035708	1.435951
H	5.963473	-2.579610	-0.855233
H	7.349063	-0.972159	0.423657
H	-1.962383	5.152722	0.106085
Cu	-3.146571	-1.196649	0.056669
O	-5.034580	-0.642206	0.804522
H	-5.654361	-1.358045	0.623980
O	-3.390683	-3.240530	0.537734
H	-4.320225	-3.399024	0.737078
H	-2.929878	-3.422614	1.364329
H	-5.356098	0.102227	0.283370
H	0.522090	-2.807894	-0.350002

SCF Energy: -1302.78754777
Sum of electronic and zero-point Energies= -1302.526664
Sum of electronic and thermal Energies= -1302.503912
Sum of electronic and thermal Enthalpies= -1302.502968
Sum of electronic and thermal Free Energies= -1302.578069

==> ./AOX-II/mono/c4c5 <==

O	1.868833	1.201654	-0.061949
O	1.160148	-2.313978	-0.193055
O	-2.898630	0.909065	-0.201799
O	-1.242119	-1.361058	-0.206887
O	-0.654986	5.104569	-0.011359
C	-0.516815	0.948507	-0.132758
C	0.640845	1.755200	-0.076292
C	2.077828	-0.130575	-0.105482
C	-0.341961	-0.469596	-0.175690
C	1.005792	-0.974800	-0.173928
C	3.492535	-0.495204	-0.018835
C	-1.759006	1.638853	-0.142564
C	0.606354	3.139237	-0.034410
C	-0.631507	3.760705	-0.050155
C	-1.817034	3.012022	-0.104499
C	4.377290	0.344842	0.672501
C	3.986008	-1.664059	-0.613516
C	5.719611	0.011777	0.780700

C	5.333117	-1.984122	-0.509639
C	6.201919	-1.153561	0.191124
H	1.524172	3.710247	0.007720
H	-2.778678	3.514522	-0.116277
H	4.008159	1.251107	1.137415
H	3.324800	-2.312929	-1.171418
H	6.390836	0.663254	1.329241
H	5.704716	-2.886877	-0.981663
H	7.251772	-1.412089	0.276382
H	0.268591	-2.696964	-0.187739
H	-1.563029	5.428733	-0.034291
Cu	-3.163596	-1.246276	0.066685
O	-5.149302	-1.123600	0.359134
H	-5.575466	-1.927332	0.037862
O	-3.161811	-3.347038	0.305594
H	-3.959949	-3.691565	-0.112071
H	-3.273142	-3.550188	1.241613
H	-5.520010	-0.406604	-0.169852
H	-3.685416	1.466421	-0.147548

SCF Energy: -1303.45609231
Sum of electronic and zero-point Energies= -1303.180694
Sum of electronic and thermal Energies= -1303.158563
Sum of electronic and thermal Enthalpies= -1303.157619
Sum of electronic and thermal Free Energies= -1303.230860

==> ./AOX-II/mono/c4c5/RAF/c3 <==

O	1.665736	1.311928	0.154616
O	0.813775	-1.871629	-1.038576
O	-2.965628	1.681243	-1.004279
O	-1.417891	-0.589626	-1.512380
O	-0.497112	5.395486	0.688138
C	-0.690384	1.410136	-0.438169
C	0.468553	2.020908	0.060894
C	1.815261	0.047833	-0.045310
C	-0.628927	0.025360	-0.820674
C	0.556508	-0.791577	-0.233572
C	3.155968	-0.433148	0.003057
C	-1.832543	2.236240	-0.552220
C	0.561875	3.333566	0.429380
C	-0.598633	4.115170	0.313608
C	-1.783501	3.570964	-0.171138
C	4.214250	0.506057	-0.039435
C	3.453810	-1.810883	0.111157
C	5.522282	0.074822	0.003520
C	4.769650	-2.224439	0.175364
C	5.800789	-1.288709	0.112606
H	1.492807	3.755935	0.781969
H	-2.673311	4.186576	-0.249954
H	3.993019	1.561098	-0.133085
H	2.660649	-2.542091	0.172588
H	6.330849	0.793450	-0.049334
H	4.996776	-3.278758	0.273242
H	6.831207	-1.623506	0.149928
H	-1.337579	5.856674	0.572868
Cu	-3.040880	-2.246302	0.406195
O	-3.898031	-0.450028	0.793994
H	-4.856740	-0.500120	0.727048
O	-2.139272	-4.024211	0.012598
H	-2.677646	-4.566049	-0.573566
H	-2.055712	-4.543465	0.819198
H	-3.631930	0.202063	0.127459
H	-3.669850	2.341971	-1.048288
H	-0.022196	-2.093960	-1.482085
O	0.109635	-1.128778	1.058154
H	0.725737	-1.755937	1.459838

SCF Energy: -1379.25419958
Sum of electronic and zero-point Energies= -1378.965577
Sum of electronic and thermal Energies= -1378.940087
Sum of electronic and thermal Enthalpies= -1378.939143
Sum of electronic and thermal Free Energies= -1379.023486

==> ./AOX-II/mono/c4c5/RAF/c4a <==

O	0.266195	1.405044	0.627931
O	1.370784	-0.679820	-2.115154
O	-3.666849	0.811581	-1.977117
O	-1.316003	-0.697425	-2.410515
O	-3.807049	0.835695	2.743758
C	-1.654423	1.123326	-0.808328
C	-1.060601	1.221622	0.563148
C	1.108526	0.803323	-0.278478
C	-0.835433	0.039692	-1.570620
C	0.588175	0.081765	-1.309917
C	2.511965	0.974423	0.078283
C	-3.142194	0.848771	-0.782827
C	-1.774893	1.138881	1.695646
C	-3.181452	0.900798	1.603164
C	-3.848301	0.733427	0.379790
C	2.864308	1.242709	1.410384
C	3.522994	0.890963	-0.891151
C	4.195372	1.408768	1.762594
C	4.850631	1.068999	-0.530581
C	5.192116	1.322447	0.794856
H	-1.307027	1.209760	2.668154
H	-4.919483	0.567945	0.362165
H	2.095576	1.311927	2.169649
H	3.271400	0.709223	-1.926925

H	4.454910	1.606928	2.796482
H	5.621923	1.013650	-1.290574
H	6.231993	1.456208	1.072125
H	-4.756091	0.668007	2.632487
Cu	0.160110	-2.591512	0.495809
O	-1.749310	-2.329238	1.099211
H	-2.235374	-3.159784	1.059605
O	2.044189	-2.779592	-0.219053
H	2.686520	-2.494569	0.439192
H	2.111753	-2.128463	-0.934956
H	-2.210889	-1.741637	0.490117
H	-4.631042	0.708481	-1.951226
H	0.788379	-1.113661	-2.757533
O	-1.411366	2.361406	-1.441980
H	-1.711216	2.319138	-2.359651

SCF Energy: -1379.23090194
Sum of electronic and zero-point Energies= -1378.941125
Sum of electronic and thermal Energies= -1378.916953
Sum of electronic and thermal Enthalpies= -1378.916009
Sum of electronic and thermal Free Energies= -1378.994874

==> ./AOX-II/mono/c4c5/RAF/c4 <==

O	-1.917044	1.149243	-0.028038
O	-1.006164	-2.226057	0.081082
O	2.817102	1.338061	0.490694
O	1.195039	-0.969793	-0.624959
O	0.221593	5.285094	-0.281180
C	0.472183	1.152656	0.249451
C	-0.716698	1.828485	0.020122
C	-2.065902	-0.145135	0.040915
C	0.505540	-0.353368	0.366796
C	-0.874967	-0.950743	0.179129
C	-3.421559	-0.613155	-0.013589
C	1.628692	1.947590	0.293711
C	-0.837676	3.196921	-0.161057
C	0.328609	3.948279	-0.103698
C	1.554231	3.329200	0.130455
C	-4.414350	0.275712	-0.490552
C	-3.805117	-1.902780	0.418943
C	-5.731194	-0.124996	-0.559349
C	-5.132880	-2.281675	0.362490
C	-6.095364	-1.403500	-0.131117
H	-1.803398	3.650699	-0.339216
H	2.462504	3.921983	0.175602
H	-4.131569	1.263276	-0.830222
H	-3.079085	-2.586970	0.830144
H	-6.481121	0.553437	-0.947672
H	-5.422544	-3.265163	0.711965
H	-7.133279	-1.712516	-0.179366
H	1.090273	5.698498	-0.225115
Cu	3.098025	-1.244384	-0.366779
O	5.175599	-1.216283	-0.124206
H	5.464589	-2.046162	0.270342
O	3.061346	-3.493054	-0.372265
H	3.965497	-3.789318	-0.519187
H	2.578536	-3.796778	-1.147733
H	5.422479	-0.538948	0.514447
H	3.533675	1.983064	0.469399
H	-0.135788	-2.670025	0.086474
O	0.983557	-0.666595	1.665896
H	1.216992	-1.603797	1.694648

SCF Energy: -1379.17864395
Sum of electronic and zero-point Energies= -1378.891167
Sum of electronic and thermal Energies= -1378.867185
Sum of electronic and thermal Enthalpies= -1378.866241
Sum of electronic and thermal Free Energies= -1378.942392

==> ./AOX-II/mono/c4c5/RAF/c1p <==

O	1.589448	1.121286	0.176405
O	0.271784	-2.115650	-0.551339
O	-3.056660	1.826739	-0.658049
O	-1.919184	-0.642153	-0.799556
O	-0.121471	5.426842	0.413360
C	-0.759471	1.378144	-0.264322
C	0.508717	1.913037	0.062480
C	1.555689	-0.222026	-0.022171
C	-0.882882	-0.026903	-0.481545
C	0.350938	-0.829664	-0.340707
C	2.827412	-0.851778	0.125198
C	-1.841898	2.306430	-0.351407
C	0.727917	3.258119	0.292290
C	-0.355349	4.122403	0.192486
C	-1.636155	3.651374	-0.125039
C	4.045229	0.037056	0.210671
C	2.988964	-2.225702	0.279111
C	5.341572	-0.656418	0.400066
C	4.243839	-2.765551	0.515318
C	5.428701	-1.990892	0.588056
H	1.716510	3.620843	0.539873
H	-2.468195	4.343769	-0.198127
H	3.905839	0.598336	1.154058
H	2.141189	-2.889689	0.240139
H	6.219154	-0.019951	0.393755
H	4.317934	-3.839180	0.648321
H	6.380885	-2.477773	0.753661
H	-0.932700	5.942197	0.325712

Cu -3.332613 -1.439793 0.360025
O -4.761947 -2.289416 1.534754
H -4.908575 -3.211053 1.297748
O -1.767279 -3.564533 -1.529684
H -2.649716 -3.361162 -1.201790
H -1.610902 -4.474791 -1.260358
H -5.614185 -1.862537 1.397578
H -3.695837 2.549483 -0.693333
H -0.627290 -2.418384 -0.852690
O 4.122361 1.001580 -0.815134
H 4.355912 0.550543 -1.634236

SCF Energy: -1379.22932722
Sum of electronic and zero-point Energies= -1378.941699
Sum of electronic and thermal Energies= -1378.916399
Sum of electronic and thermal Enthalpies= -1378.915455
Sum of electronic and thermal Free Energies= -1378.996997

==> ./AOX-II/mono/c4c5/RAF/c6p <==
O 1.511367 1.564824 -0.086152
O 0.958058 -1.899108 0.614985
O -3.223273 1.185862 0.454746
O -1.537135 -1.003225 0.632077
O -1.128032 5.363711 -0.477914
C -0.864413 1.261012 0.177266
C 0.264112 2.080938 -0.057211
C 1.738673 0.236328 0.062785
C -0.668995 -0.145424 0.388944
C 0.719285 -0.628144 0.332424
C 3.154708 -0.094875 0.053429
C -2.129092 1.922989 0.203404
C 0.186678 3.445150 -0.276118
C -1.067578 4.037126 -0.259473
C -2.221734 3.281957 -0.018835
C 4.042926 0.663355 0.799942
C 3.722281 -1.109766 -0.854519
C 5.387483 0.314156 0.881765
C 5.103654 -1.523857 -0.615183
C 5.922147 -0.781529 0.186408
H 1.082156 4.025074 -0.455353
H -3.192520 3.766765 -0.000112
H 3.667273 1.487892 1.391703
H 6.034948 0.895514 1.527849
H 5.469414 -2.375149 -1.178748
H 6.962931 -1.050749 0.309391
H -2.041343 5.672370 -0.449473
Cu -3.324679 -1.632062 0.009583
O -5.062665 -2.452923 -0.637947
H -4.961712 -3.396114 -0.802933
O -0.839678 -3.963335 0.421334
H -1.743208 -3.645894 0.316481
H -0.863403 -4.493956 1.223110
H -5.316211 -2.079885 -1.488582
H -4.008367 1.746685 0.438915
H 0.135032 -2.444865 0.599838
H 3.986519 -0.321134 -1.633987
O 2.846095 -2.020264 -1.411527
H 3.265457 -2.417602 -2.182990

SCF Energy: -1379.22343664
Sum of electronic and zero-point Energies= -1378.938532
Sum of electronic and thermal Energies= -1378.912569
Sum of electronic and thermal Enthalpies= -1378.911625
Sum of electronic and thermal Free Energies= -1378.994787

==> ./AOX-II/mono/c4c5/RAF/c7 <==
O -1.764939 1.114931 -0.065861
O -1.272074 -2.327396 0.696459
O 2.890299 0.599856 1.012414
O 1.162219 -1.498776 0.960110
O 1.199094 4.124593 -1.378986
C 0.585327 0.779233 0.446947
C -0.507114 1.608018 0.059240
C -2.072638 -0.163613 0.158457
C 0.321825 -0.611750 0.661155
C -1.048376 -1.037667 0.510085
C -3.477584 -0.467556 -0.035506
C 1.888385 1.392787 0.593401
C -0.376531 2.927966 -0.204574
C 0.947086 3.611632 -0.094280
C 2.055899 2.708215 0.333300
C -4.272556 0.433736 -0.768709
C -4.073868 -1.621327 0.503932
C -5.617432 0.177544 -0.968135
C -5.425052 -1.859638 0.308835
C -6.198655 -0.968680 -0.429509
H -1.231187 3.523038 -0.502629
H 3.035442 3.166149 0.438228
H -3.826872 1.324448 -1.192271
H -3.493026 -2.316138 1.092166
H -6.216010 0.872846 -1.545229
H -5.877076 -2.745648 0.739317
H -7.253839 -1.165164 -0.583672
H 2.141647 4.309269 -1.474200
Cu 2.827073 -1.784094 -0.170933
O 4.604616 -1.250014 -1.219707
H 5.172684 -2.020871 -1.318251
O 2.816627 -4.008245 -0.544308

H	3.726838	-4.271583	-0.712560
H	2.362767	-4.182971	-1.374703
H	5.117237	-0.636263	-0.683627
H	3.714985	1.100659	1.048698
H	-0.407229	-2.726711	0.915530
O	0.748748	4.635453	0.848210
H	1.599517	4.931043	1.195765

SCF Energy: -1379.18963689
Sum of electronic and zero-point Energies= -1378.903590
Sum of electronic and thermal Energies= -1378.877550
Sum of electronic and thermal Enthalpies= -1378.876606
Sum of electronic and thermal Free Energies= -1378.960522

==> ./AOX-II/mono/c4c5/RAF/c5p <==

O	1.372716	1.549694	0.070000
O	1.020006	-1.936747	-0.723148
O	-3.278740	0.907743	-0.788961
O	-1.452781	-1.178397	-0.861022
O	-1.523902	5.167394	0.443250
C	-0.956292	1.123386	-0.351083
C	0.100819	1.995223	-0.011892
C	1.714367	0.261706	-0.171279
C	-0.651969	-0.258497	-0.585037
C	0.752411	-0.642442	-0.494128
C	3.151800	0.025660	-0.003450
C	-2.254685	1.701973	-0.432643
C	-0.076502	3.342030	0.256654
C	-1.363072	3.855288	0.180843
C	-2.449321	3.041885	-0.162914
C	3.955825	1.015222	0.604604
C	3.762771	-1.151122	-0.391418
C	5.324419	0.858649	0.824205
C	5.200709	-1.305217	-0.287975
C	5.946565	-0.306109	0.459377
H	0.767113	3.968609	0.513913
H	-3.445929	3.465995	-0.228419
H	3.491094	1.939979	0.921414
H	3.219707	-1.958201	-0.862651
H	5.879950	1.646083	1.315757
H	7.004796	-0.473811	0.624894
H	-2.450423	5.419005	0.351811
Cu	-3.212432	-1.776688	0.007123
O	-5.243755	-2.252435	0.279659
H	-5.307433	-3.079586	0.767962
O	-2.305493	-3.116933	1.790658
H	-3.051653	-3.615809	2.136187
H	-2.058118	-2.534259	2.514610
H	-5.678930	-2.430889	-0.559910
H	-4.098473	1.417021	-0.795845
H	0.158392	-2.355619	-0.901872
H	5.382115	-0.790034	-1.299408
O	5.637814	-2.616337	-0.319056
H	6.594308	-2.632202	-0.439248

SCF Energy: -1379.22048544
Sum of electronic and zero-point Energies= -1378.936623
Sum of electronic and thermal Energies= -1378.910044
Sum of electronic and thermal Enthalpies= -1378.909100
Sum of electronic and thermal Free Energies= -1378.993489

==> ./AOX-II/mono/c4c5/RAF/c5 <==

O	1.920134	1.128835	-0.148926
O	1.346749	-2.330468	0.553734
O	-2.581980	0.863458	-0.548110
O	-1.102800	-1.402063	0.736764
O	-0.654549	4.976302	-0.473482
C	-0.398979	0.837271	0.293201
C	0.683083	1.626021	-0.033397
C	2.193029	-0.177744	0.041819
C	-0.208822	-0.559206	0.477341
C	1.153681	-1.031144	0.347263
C	3.611659	-0.494215	-0.074281
C	-1.757506	1.483277	0.425851
C	0.575502	3.002153	-0.280812
C	-0.662117	3.659805	-0.207748
C	-1.777786	2.947383	0.141243
C	4.560411	0.515862	0.153278
C	4.053249	-1.779352	-0.423552
C	5.914555	0.241212	0.046835
C	5.411185	-2.040970	-0.537660
C	6.344830	-1.037225	-0.299198
H	1.469821	3.560507	-0.532853
H	-2.745181	3.434927	0.224769
H	4.234459	1.511696	0.427143
H	3.341411	-2.567008	-0.626098
H	6.636868	1.027266	0.235910
H	5.740058	-3.035084	-0.818745
H	7.404927	-1.249464	-0.384172
H	-1.537609	5.352605	-0.373499
Cu	-3.000858	-1.383905	-0.166391
O	-4.916179	-1.054477	-1.083765
H	-5.295855	-1.898430	-1.349357
O	-3.321165	-3.586754	0.074439
H	-4.164852	-3.794892	-0.339309
H	-3.450939	-3.802356	1.003292
H	-4.812388	-0.563736	-1.905424
H	-3.441105	1.309573	-0.584480

H 0.473697 -2.711326 0.760867
O -2.196775 1.230756 1.730290
H -2.951741 1.795469 1.938641

SCF Energy: -1379.20443504
Sum of electronic and zero-point Energies= -1378.917818
Sum of electronic and thermal Energies= -1378.892288
Sum of electronic and thermal Enthalpies= -1378.891344
Sum of electronic and thermal Free Energies= -1378.972818

==> ./AOX-II/mono/c4c5/RAF/c8 <==

O	1.811649	0.884233	-0.183509
O	0.526440	-2.433314	0.193600
O	-2.799568	1.512148	0.318463
O	-1.700879	-0.999761	0.240508
O	0.101210	5.204664	-0.059402
C	-0.521660	1.085774	0.020095
C	0.735386	1.616865	-0.139743
C	1.801881	-0.480426	-0.070824
C	-0.649696	-0.355284	0.110126
C	0.596509	-1.110046	0.067158
C	3.147044	-1.051703	-0.076116
C	-1.617499	2.023193	0.138262
C	1.010310	3.070035	-0.371111
C	-0.180249	3.933299	-0.072214
C	-1.432153	3.410036	0.100040
C	4.238961	-0.250616	0.292659
C	3.377963	-2.385330	-0.442876
C	5.522589	-0.775732	0.306822
C	4.667632	-2.899611	-0.432647
C	5.742594	-2.102024	-0.054279
H	-2.285997	4.061826	0.246391
H	4.080811	0.781829	0.579688
H	2.556178	-3.017032	-0.748466
H	6.354278	-0.146361	0.603509
H	4.830839	-3.931094	-0.724790
H	6.747117	-2.510606	-0.042133
H	-0.688444	5.750994	0.076552
Cu	-3.660694	-1.204644	-0.060251
O	-5.625357	-1.549448	-0.386668
H	-5.849307	-2.473596	-0.234715
O	-1.733383	-4.006741	0.397222
H	-1.759499	-4.468317	1.240427
H	-2.528389	-3.462780	0.395667
H	-5.852485	-1.390036	-1.308873
H	-3.486352	2.192085	0.403781
H	-0.403180	-2.744899	0.294740
H	1.128495	3.151351	-1.466238
O	2.172945	3.473840	0.287028
H	2.653925	4.085781	-0.278315

SCF Energy: -1379.25970451
Sum of electronic and zero-point Energies= -1378.971539
Sum of electronic and thermal Energies= -1378.945682
Sum of electronic and thermal Enthalpies= -1378.944738
Sum of electronic and thermal Free Energies= -1379.028564

==> ./AOX-II/mono/c4c5/RAF/c2 <==

O	-1.509751	1.505477	-0.180876
O	-1.248154	-1.686656	1.280305
O	3.125212	0.845673	0.804236
O	1.273160	-1.150677	1.134298
O	1.505107	4.938733	-1.008391
C	0.820380	1.105374	0.379881
C	-0.229900	1.948965	-0.086297
C	-2.006056	0.501653	0.687747
C	0.517452	-0.214524	0.797574
C	-0.920121	-0.532620	0.908180
C	-3.229386	-0.100317	0.023310
C	2.147563	1.636848	0.358632
C	0.008858	3.218527	-0.541721
C	1.328696	3.696720	-0.552370
C	2.394035	2.914535	-0.104836
C	-3.276456	-0.239227	-1.364315
C	-4.284196	-0.566493	0.806367
C	-4.386195	-0.821494	-1.963835
C	-5.393281	-1.145434	0.199954
C	-5.446351	-1.273261	-1.183900
H	-0.802628	3.837658	-0.900004
H	3.405198	3.306193	-0.111909
H	-2.453985	0.112623	-1.975418
H	-4.245081	-0.476033	1.885609
H	-4.422234	-0.920243	-3.043026
H	-6.215645	-1.497427	0.812724
H	-6.312453	-1.725549	-1.654529
H	2.438165	5.187811	-0.988291
Cu	2.739703	-1.862710	-0.055036
O	4.618926	-2.154323	-0.998237
H	4.743098	-3.090579	-1.185103
O	1.600974	-3.639350	-0.797639
H	2.179701	-4.139742	-1.381388
H	0.883515	-3.347557	-1.368563
H	5.343823	-1.924726	-0.408182
H	3.977569	1.296297	0.737430
H	-0.425874	-2.218565	1.430250
O	-2.215133	1.003600	1.965187
H	-3.017519	1.543104	1.969155

SCF Energy: -1379.23823728

Sum of electronic and zero-point Energies= -1378.951462

Sum of electronic and thermal Energies= -1378.926681

Sum of electronic and thermal Enthalpies= -1378.925737

Sum of electronic and thermal Free Energies= -1379.005653

==> ./AOX-II/mono/c4c5/RAF/c2p <==

O	1.617046	1.139241	-0.098118
O	0.484349	-2.219099	-0.533075
O	-3.112559	1.477581	-0.611131
O	-1.798899	-0.932269	-0.681509
O	-0.374706	5.325695	0.040525
C	-0.770901	1.209560	-0.358578
C	0.474770	1.849464	-0.166159
C	1.670059	-0.218449	-0.195185
C	-0.802140	-0.212329	-0.489563
C	0.496838	-0.918588	-0.412560
C	2.989427	-0.743070	-0.045821
C	-1.919183	2.057342	-0.419541
C	0.616190	3.216846	-0.034871
C	-0.531172	3.998978	-0.094059
C	-1.794225	3.425339	-0.286913
C	4.104002	0.205781	0.320292
C	3.320623	-2.065341	-0.332871
C	5.438514	-0.395272	0.505957
C	4.630496	-2.508081	-0.229351
C	5.701943	-1.681179	0.180904
H	1.592733	3.658834	0.112818
H	-2.676237	4.055192	-0.336344
H	4.249446	0.763093	-0.636592
H	2.562907	-2.766199	-0.642085
H	6.207034	0.272631	0.877153
H	4.835731	-3.545767	-0.467531
H	6.699319	-2.090687	0.270811
H	-1.225723	5.778207	-0.000943
Cu	-3.453186	-1.325411	0.361631
O	-5.039625	-1.948545	1.471350
H	-5.105495	-2.909056	1.480173
O	-1.626983	-3.876205	-0.831960
H	-2.433885	-3.555469	-0.414619
H	-1.408413	-4.681564	-0.353291
H	-5.866585	-1.649969	1.078574
H	-3.809872	2.145160	-0.620780
H	-0.430839	-2.595320	-0.645224
O	3.854767	1.097473	1.376248
H	3.173699	1.720847	1.101843

SCF Energy: -1379.23179270

Sum of electronic and zero-point Energies= -1378.944891

Sum of electronic and thermal Energies= -1378.919357

Sum of electronic and thermal Enthalpies= -1378.918412

Sum of electronic and thermal Free Energies= -1379.000660

==> ./AOX-II/mono/c4c5/RAF/c6 <==

O	1.806692	1.141710	-0.189203
O	1.440138	-2.398768	0.360028
O	-2.809226	0.591949	0.490266
O	-1.057383	-1.615057	0.407383
O	-0.976795	4.835560	-0.244290
C	-0.508026	0.689153	0.018710
C	0.570118	1.592419	-0.167975
C	2.159878	-0.176169	-0.028093
C	-0.219742	-0.711855	0.208323
C	1.176940	-1.098331	0.170073
C	3.605516	-0.370857	-0.064753
C	-1.781670	1.255126	0.097810
C	0.452168	2.990198	-0.267161
C	-0.791823	3.540123	-0.251156
C	-2.004495	2.664992	-0.350118
C	4.456983	0.702354	0.240969
C	4.167510	-1.610538	-0.403481
C	5.833118	0.532784	0.222162
C	5.546389	-1.767293	-0.428284
C	6.383115	-0.701653	-0.111907
H	1.343827	3.601348	-0.290305
H	-2.138566	2.540205	-1.442106
H	4.039289	1.666038	0.504875
H	3.532592	-2.445888	-0.663273
H	6.478213	1.368075	0.470789
H	5.968074	-2.728601	-0.699877
H	7.459641	-0.831802	-0.127386
H	-1.918047	5.046170	-0.123134
Cu	-2.813502	-2.064882	-0.484977
O	-4.572544	-2.573948	-1.376781
H	-4.929310	-3.379409	-0.988105
O	-2.672596	-1.550744	2.930345
H	-3.206393	-0.753266	2.885465
H	-2.201638	-1.556752	2.087557
H	-4.436538	-2.788757	-2.305511
H	-3.587961	1.182761	0.542161
H	0.584117	-2.837562	0.500953
O	-3.142899	3.218965	0.228932
H	-3.794730	3.418986	-0.451916

SCF Energy: -1379.25510810

Sum of electronic and zero-point Energies= -1378.966965

Sum of electronic and thermal Energies= -1378.941149
Sum of electronic and thermal Enthalpies= -1378.940205
Sum of electronic and thermal Free Energies= -1379.024106

==> ./AOX-II/mono/c4c5/RAF/c8a <==

O	-1.558686	1.211187	-0.135057
O	-1.031309	-2.168570	1.016556
O	3.080445	0.874739	0.415661
O	1.413965	-1.203348	1.183614
O	0.957167	4.961084	-0.874240
C	0.731859	1.004545	0.570868
C	-0.503076	1.865241	0.557230
C	-1.833032	-0.079645	0.201294
C	0.512390	-0.377914	0.882258
C	-0.841544	-0.863478	0.740886
C	-3.189483	-0.484391	-0.137324
C	1.956403	1.578816	0.272338
C	-0.362173	3.216244	-0.057503
C	0.859704	3.710232	-0.395194
C	2.000704	2.894340	-0.225748
C	-3.892624	0.232653	-1.122133
C	-3.826294	-1.561427	0.502247
C	-5.181784	-0.134291	-1.471714
C	-5.123318	-1.909863	0.156781
C	-5.802593	-1.205453	-0.833404
H	-1.260916	3.817729	-0.133386
H	2.970697	3.301904	-0.500615
H	-3.417897	1.066518	-1.623264
H	-3.319125	-2.108235	1.284233
H	-5.705669	0.417888	-2.243803
H	-5.608184	-2.734123	0.667556
H	-6.814236	-1.487115	-1.104147
H	1.872600	5.190484	-1.075139
Cu	2.848844	-1.668980	-0.195833
O	4.558324	-1.523732	-1.447152
H	4.854531	-2.404457	-1.698678
O	2.165777	-3.759732	-0.388293
H	2.267246	-4.073760	-1.292211
H	1.213982	-3.745130	-0.241480
H	5.289564	-1.157399	-0.938974
H	3.844205	1.335295	0.042666
H	-0.171392	-2.518184	1.305022
O	-0.847485	2.045613	1.906224
H	-1.568305	2.687424	1.967572

SCF Energy: -1379.18246137
Sum of electronic and zero-point Energies= -1378.896441
Sum of electronic and thermal Energies= -1378.870540
Sum of electronic and thermal Enthalpies= -1378.869596
Sum of electronic and thermal Free Energies= -1378.952308

==> ./AOX-II/mono/c4c5/RAF/c4p <==

O	1.411032	1.375166	0.119655
O	0.779706	-2.069103	-0.635569
O	-3.264876	1.047253	-0.776847
O	-1.588862	-1.150826	-0.840657
O	-1.230040	5.171394	0.485867
C	-0.944267	1.109178	-0.314578
C	0.174315	1.902282	0.036054
C	1.687492	0.062328	-0.114640
C	-0.745896	-0.282949	-0.538192
C	0.639605	-0.784550	-0.416919
C	3.078720	-0.252999	-0.038460
C	-2.202842	1.776567	-0.406435
C	0.083110	3.253376	0.308427
C	-1.167084	3.855433	0.224552
C	-2.308146	3.123774	-0.128213
C	4.005462	0.805783	0.220365
C	3.566165	-1.587964	-0.189285
C	5.336055	0.566383	0.263676
C	4.896203	-1.837876	-0.141413
C	5.890316	-0.750530	-0.079568
H	0.964035	3.823879	0.570555
H	-3.271166	3.618156	-0.201735
H	3.634766	1.803063	0.404886
H	2.876988	-2.408580	-0.302196
H	6.039684	1.362203	0.478629
H	5.271312	-2.851768	-0.227207
H	6.120461	-0.603086	-1.165799
H	-2.130867	5.499532	0.378914
Cu	-3.369459	-1.610859	0.080199
O	-5.392421	-1.491594	0.735763
H	-5.697804	-2.374869	0.965823
O	-2.759715	-3.760699	0.729608
H	-3.530121	-4.185877	1.118035
H	-2.123145	-3.721916	1.449735
H	-5.968400	-1.217163	0.015341
H	-4.057537	1.598188	-0.794861
H	-0.118126	-2.409364	-0.838291
O	7.072768	-1.026603	0.625114
H	7.548249	-1.726813	0.166728

SCF Energy: -1379.22963329
Sum of electronic and zero-point Energies= -1378.944198
Sum of electronic and thermal Energies= -1378.917730
Sum of electronic and thermal Enthalpies= -1378.916786

Sum of electronic and thermal Free Energies= -1379.001269

==> ./AOX-II/mono/c4c5/RAF/c3p <==

O	1.588039	1.194985	-0.117099
O	0.698908	-2.236081	-0.711880
O	-3.156045	1.199053	-0.664059
O	-1.652380	-1.133190	-0.756292
O	-0.733280	5.209150	0.215834
C	-0.801389	1.093292	-0.388874
C	0.388545	1.816313	-0.152157
C	1.720518	-0.136445	-0.318488
C	-0.713382	-0.327554	-0.572249
C	0.625159	-0.905767	-0.543293
C	3.112842	-0.584819	-0.210572
C	-2.006708	1.851776	-0.421792
C	0.423299	3.185161	0.051925
C	-0.778089	3.877442	0.017691
C	-1.989202	3.217813	-0.220675
C	4.010554	0.138323	0.547694
C	3.571519	-1.755900	-0.848250
C	5.410931	-0.229867	0.588058
C	4.889825	-2.205124	-0.745234
C	5.794434	-1.510346	0.013387
H	1.362234	3.692688	0.228760
H	-2.916244	3.781053	-0.254543
H	3.721252	1.041183	1.071403
H	2.881573	-2.328639	-1.453086
H	5.645189	0.353354	-0.373611
H	5.182715	-3.117675	-1.247149
H	6.824126	-1.831330	0.122913
H	-1.620044	5.584563	0.168388
Cu	-3.443534	-1.375809	0.217961
O	-5.324592	-1.000345	1.098862
H	-5.940262	-1.711259	0.893795
O	-3.421677	-3.777913	0.615233
H	-4.343339	-3.992391	0.786893
H	-2.988838	-3.915996	1.462849
H	-5.717043	-0.215877	0.702978
H	-3.897298	1.816515	-0.634154
H	-0.219603	-2.541157	-0.820001
O	6.110755	0.288746	1.660767
H	7.057107	0.182418	1.509742

SCF Energy: -1379.22009159
Sum of electronic and zero-point Energies= -1378.936048
Sum of electronic and thermal Energies= -1378.909344
Sum of electronic and thermal Enthalpies= -1378.908400
Sum of electronic and thermal Free Energies= -1378.993865

==> ./AOX-II/mono/c4c5/vEA <==

O	1.868833	1.201654	-0.061949
O	1.160148	-2.313978	-0.193055
O	-2.898630	0.909065	-0.201799
O	-1.242119	-1.361058	-0.206887
O	-0.654986	5.104569	-0.011359
C	-0.516815	0.948507	-0.132758
C	0.640845	1.755200	-0.076292
C	2.077828	-0.130575	-0.105482
C	-0.341961	-0.469596	-0.175690
C	1.005792	-0.974800	-0.173928
C	3.492535	-0.495204	-0.018835
C	-1.759006	1.638853	-0.142564
C	0.606354	3.139237	-0.034410
C	-0.631507	3.760705	-0.050155
C	-1.817034	3.012022	-0.104499
C	4.377290	0.344842	0.672501
C	3.986008	-1.664059	-0.613516
C	5.719611	0.011777	0.780700
C	5.333117	-1.984122	-0.509639
C	6.201919	-1.153561	0.191124
H	1.524172	3.710247	0.007720
H	-2.778678	3.514522	-0.116277
H	4.008159	1.251107	1.137415
H	3.324800	-2.312929	-1.171418
H	6.390836	0.663254	1.329241
H	5.704716	-2.886877	-0.981663
H	7.251772	-1.412089	0.276382
H	0.268591	-2.696964	-0.187739
H	-1.563029	5.428733	-0.034291
Cu	-3.163596	-1.246276	0.066685
O	-5.149302	-1.123600	0.359134
H	-5.575466	-1.927332	0.037862
O	-3.161811	-3.347038	0.305594
H	-3.959949	-3.691565	-0.112071
H	-3.273142	-3.550188	1.241613
H	-5.520010	-0.406604	-0.169852
H	-3.685416	1.466421	-0.147548

SCF Energy: -1303.61354453

==> ./AOX-II/mono/c4c5/vIP <==

O	1.868833	1.201654	-0.061949
O	1.160148	-2.313978	-0.193055
O	-2.898630	0.909065	-0.201799
O	-1.242119	-1.361058	-0.206887
O	-0.654986	5.104569	-0.011359
C	-0.516815	0.948507	-0.132758
C	0.640845	1.755200	-0.076292
C	2.077828	-0.130575	-0.105482
C	-0.341961	-0.469596	-0.175690

C	1.005792	-0.974800	-0.173928
C	3.492535	-0.495204	-0.018835
C	-1.759006	1.638853	-0.142564
C	0.606354	3.139237	-0.034410
C	-0.631507	3.760705	-0.050155
C	-1.817034	3.012022	-0.104499
C	4.377290	0.344842	0.672501
C	3.986008	-1.664059	-0.613516
C	5.719611	0.011777	0.780700
C	5.333117	-1.984122	-0.509639
C	6.201919	-1.153561	0.191124
H	1.524172	3.710247	0.007720
H	-2.778678	3.514522	-0.116277
H	4.008159	1.251107	1.137415
H	3.324800	-2.312929	-1.171418
H	6.390836	0.663254	1.329241
H	5.704716	-2.886877	-0.981663
H	7.251772	-1.412089	0.276382
H	0.268591	-2.696964	-0.187739
H	-1.563029	5.428733	-0.034291
Cu	-3.163596	-1.246276	0.066685
O	-5.149302	-1.123600	0.359134
H	-5.575466	-1.927332	0.037862
O	-3.161811	-3.347038	0.305594
H	-3.959949	-3.691565	-0.112071
H	-3.273142	-3.550188	1.241613
H	-5.520010	-0.406604	-0.169852
H	-3.685416	1.466421	-0.147548

SCF Energy: -1303.23467064
